# SARS-CoV-2 Genetic Diversity and Lineage Dynamics in Egypt during the First 18 Months of the Pandemic

**DOI:** 10.3390/v14091878

**Published:** 2022-08-25

**Authors:** Wael H. Roshdy, Mohamed K. Khalifa, James Emmanuel San, Houriiyah Tegally, Eduan Wilkinson, Shymaa Showky, Darren Patrick Martin, Monika Moir, Amel Naguib, Nancy Elguindy, Mokhtar R. Gomaa, Manal Fahim, Hanaa Abu Elsood, Amira Mohsen, Ramy Galal, Mohamed Hassany, Richard J. Lessells, Ahmed A. Al-Karmalawy, Rabeh EL-Shesheny, Ahmed M. Kandeil, Mohamed A. Ali, Tulio de Oliveira

**Affiliations:** 1 Central Public Health Laboratory, Ministry of Health and Population, Cairo 11613, Egypt; 2 Omicsense, Cairo 11799, Egypt; 3 KwaZulu-Natal Research Innovation and Sequencing Platform (KRISP), Nelson R Mandela School of Medicine, University of KwaZulu-Natal, Durban 4001, South Africa; 4 Centre for Epidemic Response and Innovation (CERI), School of Data Science and Computational Thinking, Stellenbosch University, Stellenbosch 7600, South Africa; 5 Institute of Infectious Disease and Molecular Medicine, University of Cape Town, Observatory, Cape Town 7700, South Africa; 6 Wellcome Centre for Infectious Diseases Research in Africa (CIDRI-Africa), University of Cape Town, Observatory, Cape Town 7700, South Africa; 7 Centre of Scientific Excellence for Influenza Viruses, National Research Centre, Dokki, Giza 12622, Egypt; 8 Department of Surveillance and Epidemiology, Ministry of Health and Population, Cairo 12622, Egypt; 9 World Health Organization, Egypt Country Office, Cairo 12622, Egypt; 10 Public Health Initiative, Cairo 11613, Egypt; 11 National Hepatology and Tropical Medicine Research Institute, Ministry of Health and Population, Cairo 11613, Egypt; 12 Department of Pharmaceutical Medicinal Chemistry, Faculty of Pharmacy, Horus University-Egypt, New Damietta 34518, Egypt; 13 Department of Global Health, University of Washington, Seattle, WA 98195, USA

**Keywords:** genomic epidemiology, SARS-CoV-2, Egypt

## Abstract

COVID-19 was first diagnosed in Egypt on 14 February 2020. By the end of November 2021, over 333,840 cases and 18,832 deaths had been reported. As part of the national genomic surveillance, 1027 SARS-CoV-2 near whole-genomes were generated and published by the end of July 2021. Here we describe the genomic epidemiology of SARS-CoV-2 in Egypt over this period using a subset of 976 high-quality Egyptian genomes analyzed together with a representative set of global sequences within a phylogenetic framework. A single lineage, C.36, introduced early in the pandemic was responsible for most of the cases in Egypt. Furthermore, to remain dominant in the face of mounting immunity from previous infections and vaccinations, this lineage acquired several mutations known to confer an adaptive advantage. These results highlight the value of continuous genomic surveillance in regions where VOCs are not predominant and the need for enforcement of public health measures to prevent expansion of the existing lineages.

## 1. Introduction

Over the first 18 months of the COVID-19 pandemic, several lineages of the SARS-CoV-2 characterized by diverse constellations of mutations emerged. Some of these lineages have manifested phenotypes of increased transmissibility, disease severity, and escape from neutralizing antibodies and have therefore been classified as variants of concern (VOC); e.g., Alpha, Beta, Gamma, Delta and most recently, Omicron [1]. Other lineages carrying mutational loads similar to those of VOCs, and which therefore might in the future present similar phenotypes to VOCs, have been classified as variants of interest (VOI), e.g., Eta, Iota, Kappa and Lambda. The World Health Organization (WHO) and the European Centre for Disease Prevention and Control (ECDCP) further designated a third category of variants: variants under monitoring (VUM), that, although not yet confirmed as an immediate exceptional risk, might pose future threats to pharmaceutical and non-pharmaceutical interventions devised to control or end the pandemic [2].

To date, studies to understand the impact of genomic variations on viral phenotypes have largely focused on VOCs and VOIs. Accordingly, stringent public health measures such as travel restrictions and lockdowns have been implemented at different times and in different regions of the world to control the spread of these variants. Considerably less attention has been given to understanding the emergence, local spread, and persistence of non-VOC or VOI lineages in the parts of the world where they emerged. This has favored the continued evolution of some of these lineages in the regions where they persist, evolution that, independently of that ongoing elsewhere, has, in some cases, yielded variants with improved transmission and/or immune evasion phenotypes.

The first case of COVID-19 in Egypt was reported on 14 February 2020, in a foreign national of undisclosed nationality [3]. On 13 March 2020, the first two SARS-CoV-2 genomes from Egypt were published [4]. By 18 March 2020, at least 256 cases and 7 deaths had been reported. As of 3 November 2021, 333,840 confirmed cases and 18,832 deaths (crude case-fatality rate 5.6 percent) have been reported [5], making Egypt, based on this metric at least, the most affected country in Africa after South Africa.

Here we describe the molecular epidemiology of SARS-CoV-2 in Egypt up to July 2021, focusing on the C.36 lineage that circulated within the country from early in the pandemic. This lineage evolved into sub-lineages C.36.1, C.36.3 and C.36.3.1, acquiring mutations known to confer fitness and pathogenic properties similar to those found in VOCs; with C.36.3 having since been classified as a VUM in June 2021. Our results highlight the importance of sustained surveillance and public health efforts to minimize the transmission, replication, and evolution of variants within the regions where they arise.

## 2. Materials and Methods

### 2.1. Ethics Statement

Ethical approval was obtained from the Ethics Committee of the National Research Centre, Giza, Egypt protocol number 14155, on 22 March 2020.

### 2.2. Epidemiological Data

Counts of daily cases and deaths analyzed were retrieved from the online data repository, Our World in Data (OWID) [6]. The effective reproductive number (Re) dataset was retrieved from the online repository, COVID-19-Re/dailyRe-Data [7,8]. Both epidemiological datasets analyzed were limited to data collected between 1 March 2020 and 15 July 2021 in line with the genomic dataset.

### 2.3. qPCR analyzed Samples

The study examined 1076 nasopharyngeal (NP) swab samples obtained from visa applicants, tourists, and patients in isolation throughout Egypt between 1 January 2020 and 31 July 2021. Briefly, samples were collected with flocked nasopharyngeal swabs and immersed in viral transport medium for delivery to local governorate laboratories for testing with SARS-CoV-2 PCR protocols. The chemagic 360 equipment (PerkinElmer Inc, Waltham, United States) was used to extract nucleic acid from the clinical samples. The local laboratories governorates performed the initial SARS-CoV-2 qPCR screening using the Viasure SARS-CoV-2 Real-Time PCR Detection Kit (Certest Biotec SL, Zaragoza, Spain). The Applied Biosystems QuantStudio 7 Flex Real-Time PCR Detection equipment with QuantStudio Real-Time PCR software v.1.3 (Thermo Fisher Scientific, Waltham, MA, USA) was used for RT-PCR. The samples were then sent to the central public health laboratory (CPHL) under optimal storage conditions. All accepted samples had a cycle threshold (CT) value less than 35. Positive SARS-CoV-2 samples were confirmed at CPHL using the Cobas 6800 system (Roche Holding AG, Basel, Switzerland).

### 2.4. Mutation Screening Assays

The C36.3 lineage was confirmed by the presence of amino acid deletion HV69-70 and substitution L452R in the Spike gene using a multiplex real-time RT-PCR assay and the VIASURE (Certest Biotec SL, Zaragoza, Spain) SARS-CoV-2 detection kit on the Applied Biosystems QuantStudio 7 Flex Real-Time PCR detection equipment and QuantStudio Real-Time PCR software v.1.3 (Thermo Fisher Scientific, Waltham, MA, USA). Additionally, Alpha variant mutations were confirmed using the SNPsig-Variplex SARS-CoV-2 SNP genotyping panel with primers and probes designed to detect 20I/501Y.V1 (B.1.1.7), 20H/501Y.V2 (B.1.351), 20J/501Y.V3 (P1), and 20C/S.452R. Briefly, the amplification profile was: 10 min at 55 °C, 2 min at 95 °C, and 45 cycles, each consisting of 10 s at 95 °C and 60 s at 60 °C. All the mentioned procedures were done according to the manufacturer’s instructions. Delta lineage mutations were identified using the Thermo Fisher Scientific (Thermo Fisher Scientific, Waltham, MA, USA) Applied Biosystems TaqMan SARS-CoV-2 Mutation Panel. Other SNP combinations, such as 501Y, 484K, and L452R and wild-types 501N, 484E, and 452L; deletion of amino acids H69 and V70, N501Y, E484K, and K417N; K417T, P681R, P681H, L452R, and K417N; and Q677H were identified with (Primerdesign Ltd., COVID-19 genesig, Chandler’s Ford, UK) following the manufacturer’s instructions.

### 2.5. SARS-CoV-2 Whole-Genome Sequencing

Ribosomal RNA was removed from previously extracted RNA using the ribo zero assay. This was followed by double stranded cDNA synthesis using the Truseq Stranded Total RNA (Illumina, San Diego, CA, USA) per the manufacturer’s conditions and sequenced on the Illumina MiSeq sequencing platform. Strain typing assays (NGS) were cross-validated using a set of 250 SARS-CoV-2-positive samples and 50 SARS-CoV-2-negative samples.

### 2.6. Genomic Analysis and Strain Typing

Raw short read sequence data was assembled and analyzed using Dragen for SARS-CoV-2 variant detection (for the Truseq assay). Adequate sequencing required coverage of strain-distinguishing areas of the open reading frame 1a (ORF1a), S, N, and ORF8 genes to a depth of at least 100×. All but two sequences in this series yielded adequate strain-typeable sequences, and no sample contained a mixed population of viruses. Lineages and clades were assigned using the dynamic lineage classification tool Phylogenetic Assignment of Named Global Outbreak LINeages (Pangolin) [9] and NextStrain [10].

### 2.7. Phylogenetic Analysis

A subset of available Egyptian SARS-CoV-2 sequences on the GISAID database were retrieved (*n* = 976; date of access: 9 August 2021) and analyzed against a globally representative dataset of SARS-CoV-2 isolates (*n* = 6561) to track and monitor the spatial temporal evolution of the virus. Global reference sequences were chosen based on their previous inclusion in the global and African phylogenetic builds of the NextStrain platform (https://nextstrain.org/ncov/gisaid/global; date of access: 9 August 2021). Additionally, due to the importance of the C.36 lineage and sub-lineages in Egypt we included all globally sampled C.36, and the sub-lineages on the GISAID database were also included (date of access: 9 August 2021). This sampling strategy ensures that sufficient global viral diversity was included in the downstream analysis.

Sequences were aligned using NextAlign [11] to obtain a good codon alignment of the sequences. A maximum likelihood tree topology was inferred from the resulting alignment in IQTREE 2 [12] using the General Time Reversible model of nucleotide substitution [13] and a total of 100 bootstrap replicates to infer support for branches in the resulting tree topology using Booster [14]. Firstly, the topology was assessed in TemPest [15] to remove any potential outlier sequences. The tree topology was then transformed into a dated tree topology where the branches correspond to units of calendar time using TreeTime [16] with a strict molecular clock assumption at a rate of 8 × 10^−4^ mutations/site/year. The dated SARS-CoV-2 phylogeny was used along with the associated metadata that was obtained from GISAID, to map discrete geographical locations to each of the tips and infer locations for the internal nodes. This was done using the Mugration package extension of TreeTime. A custom Python script was then used to count the number of discrete changes occurring as we transcended the topology from the root towards the tips. In essence, this provided a crude estimate of the number and timing of viral exchanges (import-export) between Egypt and the rest of the world. 

## 3. Results

### 3.1. Genomic Epidemiology of SARS-CoV-2 in Egypt

**Figure 1:**
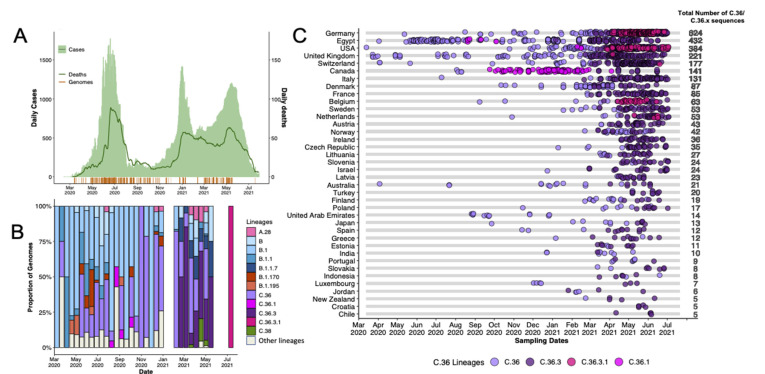
Epidemiological dynamics of the SARS-CoV-2 epidemic in Egypt. (**A**) Histogram showing the number of daily COVID-19 cases overlayed with a line plot of deaths: in the bottom, timing of the genomic sampling in Egypt. (**B**) Progressive distribution of the major SARS-CoV-2 lineages in Egypt. (**C**) Temporal sampling of sequences of the C.36 SARS-CoV-2 lineages globally in countries with at least 5 sequences (ordered by the total number of sequences). At the time of writing, there was only one sequence of lineage C.36.3.1.

Following the initial introduction, cases of SARS-CoV-2 quickly increased leading to the first wave. This wave lasted between April 2020 and July 2020 reaching its peak in May 2020 with over 1500 cases and just under 100 deaths recorded daily (Figure 1A). The wave was dominated by B-lineage viruses (Figure 1B).

The first wave was followed by a brief lull in case numbers between August and October 2020, then the cases began to rise again leading to a brief second wave that quickly progressed into a third wave, as of July 2021 (Figure 1A). The second wave was dominated by the C.36 lineage while the third wave was dominated by the C.36, C.36.3 and Alpha variants (Figure 1B,C). Figure 1C also shows a similar progression of the C.36 lineages globally. Outside of Egypt, the C.36.3 lineage has subsequently been detected in 37 countries around the world and the C.36.3.1 in six countries (Figure 1C).

### 3.2. Phylogenetic Analysis and Lineage Dynamics

By July 2021, 1027 SARS-CoV-2 near-full length genomes from Egypt had been submitted to the GISAID [17] database. Of these 1027 SARS-CoV-2 genome sequences downloaded from the GISAID database [17] on 31 July 2021, 976 genomes sampled between 08 March 2020 and 16 May 2021 were of good quality (genome coverage range 82.6–99.5%, with the collection date comprising at least the month and year) (Tables S1 and S3) and were associated with the relevant metadata. The majority of the genomes were sampled during the first and third waves of the pandemic in Egypt, which were also the most severe. Figure 1A shows a high correlation between cases, recorded case fatality, and genomes generated in Egypt during these periods. One major drawback of the associated metadata in the context of our analysis was the paucity of regional sampling information. At least 710 (73%) of the sequences were not allocated to any governorates. Two hundred and sixty-three (27%) genomes were sampled in Cairo, while the Kalyoubia and Faiyum governorates had only one and two sequences, respectively, assigned to them. It was therefore not possible to reconstruct the intra-Egypt viral dispersal history in a continuous space (continuous phylogeography) on these samples. 

**Figure 2:**
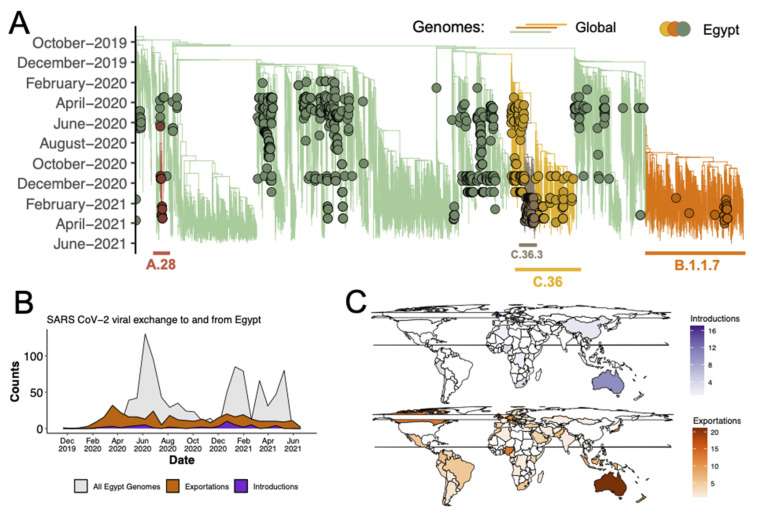
Phylogenetic reconstruction of the SARS-CoV-2 epidemic in Egypt. (**A**) Time-resolved maximum clade credibility phylogeny of the major lineages circulating in Egypt. (**B**) Area plot showing viral import-export as proportions of the sampled genomes. (**C**) Inferred locations of imports into and exports of SARS-CoV-2 out of Egypt. Sampling for molecular and genomic analyses across Egypt were sporadic across 2020 and 2021. Although genomic sequencing only detected C.36/C.36.3 in Cairo (GISAID Metadata), qPCR assays were able to detect these lineages in various other locations (Figure 3B) at a high proportion of the total numbers of samples tested with the qPCR assay (Figure 3A).

To contextualize the epidemic in Egypt, we therefore analyzed these sequences in a phylogenetic framework with a representative set of global sequences. The inferred phylogeny (Figure 2A) enabled us to estimate the number of viral imports and exports between Egypt and the rest of the world. Our analysis suggested that there were at least 58 unique introductions of SARS-CoV-2 to Egypt: 20 (34%) from Europe, 12 (20%) from Africa, 10 (17%) from Oceania, 9 (15%) from Asia and 7 (11%) from the Americas (Figure S1A), with the earliest introduction from Australia. At an individual country level, most of the viral introductions apparently emanated from Australia (*n* = 9, 16%) and the United Kingdom (*n* = 9, 16%). Viral imports peaked over December 2020 and January 2021, coinciding with the high tourism season. We identified a notably greater number of export events (at least 364) than introductions, mostly to Europe and the USA (Figure 2B,C and Figure S1). A genomic analysis of the sequences revealed the circulation of at least 36 distinct SARS-CoV-2 lineages spread across the three waves, which is in line with the number of introductions, i.e., some lineages were introduced multiple times.

The first wave, as is the case with most countries, was primarily dominated by lineage B viruses, i.e., the B.1 (*n* = 287, 32%) and B.1.1 (*n* = 57, 6%) PANGO lineages. Viruses belonging to these lineages carry the well-known D614G mutation that has been linked to increased transmissibility [18] but lack any of the other characteristic Spike mutations associated with VOCs or VOIs. Lineage A.28, the only A-lineage detected in Egypt was first sampled around August 2020. However, similar to other parts of the continent, the A.28 lineage did not take off, and by May 2021, no new sequences of the A.28 lineage were sampled in Egypt.

The only VOC detected in Egypt was Alpha (Figure 1B), with the first sequence being identified in March 2021. Interestingly, unlike the rapid rise of Alpha in many European countries throughout the first months of 2021, Egypt did not see a similar growth in the number of cases attributed to Alpha following its introduction (Figure 1B). Rather, the majority of cases were travel-associated, with minimal subsequent community transmission.

### 3.3. Introduction of the C.36 lineage in Egypt

**Figure 3:**
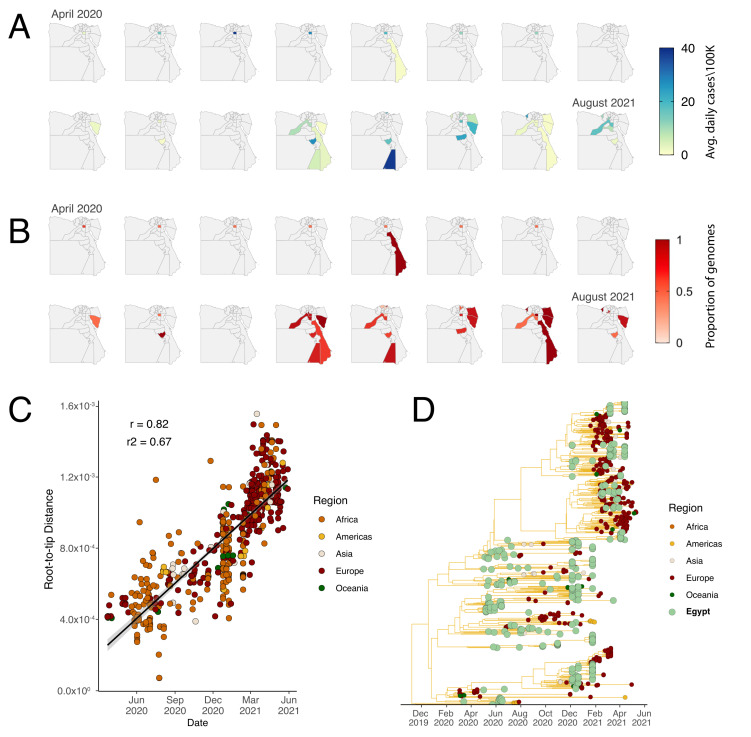
Average daily cases and distribution of the C.36 lineages in Egypt over time, and root-to-regression and phylogenetic placement of the Egypt C.36 genomes in the context of global C.36 sequences. (**A**) Prevalence maps following the progression in the daily number of cases, and (**B**) Proportions of C.36.3 lineage samples per governorate in Egypt confirmed by RT-PCR from April 2020 to July 2021. (**C**) Root-to-tip regression plot of the C.36 lineages. (**D**) Maximum clade credibility phylogenetic tree including all the global sequences of the C.36 lineages.

**Figure 4:**
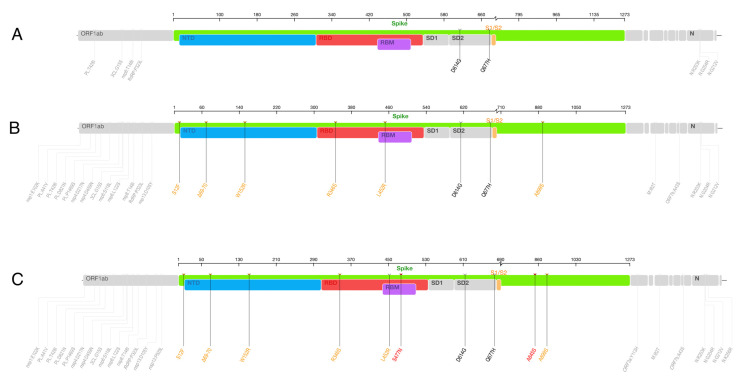
Genomic maps showing lineage defining mutations of the C.36 lineages. (**A**) Lineage defining mutations of the C.36 lineage, (**B**) lineage defining mutations of the C.36.3 sub-lineage, and (**C**) lineage defining mutations in the C.36.3.1 sub-lineage. Additional Spike gene mutations in the C.36.3 lineage (*n* = 6) are annotated in orange and mutations unique to C.36.3.1 (*n* = 2) in red.

The C.36 lineage was detected very early on in the epidemic in Egypt (in March 2020) and has since continued to circulate within the country at varying frequencies (Figure 1B and Figure 3D). Relative to B.1, the C.36 lineage is characterized by an additional mutation, Q677H in its Spike gene, upstream of the S1/S2 furin cleavage site (Figure 4A). The lineage persisted through the second and third waves during which it accounted for the majority of the recorded cases (Figure 1B).

### 3.4. Evolution and Spread of C.36 Sub-Lineages

As of August 2020, the C.36 lineage began to evolve into sub-lineages that included C.36.1, C.36.3, and C.36.3.1. The emergence of these sub-lineages occurred around the same time as the emergence of other known VOCs and was therefore suggestive of a similar shift in the selective environment in which SARS-CoV-2 was evolving in Egypt. 

Lineage C.36.3 which arose from approximately March 2021 through stepwise evolution rather than an evolutionary jump (Figure 2A) was particularly associated with a selective advantage (Figure 1B) and was responsible for the majority of cases in Egypt during the third wave. As our genomic data was largely limited to Cairo, to characterize the spread of C.36.3 across Egypt, we utilized real-time polymerase chain reaction (RT-PCR) data from 1076 nasopharyngeal swab samples with high quality demographic data. Of the 1076 samples analyzed, 462 samples (42.9%) belonged to the C.36.3 sub-lineage while only 35 samples (3.3%) belonged to the Alpha variant. Of the 462 samples, at least 33% were from Cairo, 13% from Sinai, 11.8% from Giza, 9.9% from Sohage, 9.1% from Aswan, 8.6% from Alexandria, 4.9% Dakhlia, and 4.7% from Luxor; the remaining 8% was spread across the remaining governorates (Figure 3A,B). Together, these results show the widespread community transmission of C.36.3 in all population centers of Egypt. An in-depth phylogenetic analysis suggested that the lineage evolved in a clock-like manner with Egypt as its potential origin (Figure 3C,D).

### 3.5. Mutational and Selection Analysis of the C.36.3 Lineage

The C.36.3 lineage accumulated an additional six Spike mutations and eleven non-Spike mutations over the core set of mutations of the C.36 lineage (Figure 4A,B). Notably, the Spike mutation L452R occurred in the receptor binding motif (RBM) as the R346S mutations in the RBD and the ∆HV69-70 deletion originally detected in the Alpha variant in the N-terminal domain (NTD). The lineage also acquired two additional mutations in the NTD, S12F and W152R. By July 2021, the C.36.3 lineage had evolved into the C.36.3.1 sub-lineage, adding two additional Spike mutations: S477N and A845S (Figure 4C). 

## 4. Discussion

In this study, we assessed the genomic epidemiology of SARS-CoV-2 in Egypt over the first 18 months of the pandemic. We show that the virus was introduced into Egypt multiple times, resulting in the identification of at least 36 unique viral lineages detected in the population. Continued genomic surveillance throughout 2020 and 2021 further illuminated the population dynamics of the SARS-CoV-2 lineages circulating in Egypt. Specifically, we show that the first wave of the pandemic in Egypt was largely dominated by lineage B viruses (particularly B.1), while the second and third waves were dominated by C-like lineages (especially C.36).

We observe that although the Beta variant dominated the second and third waves across the continent, there was no incidence of the variant in Egypt at the time of this study. This could possibly be attributed to under-sampling, compounded by limited viral exchanges with sub-Saharan countries, a known trend between countries in North Africa and those in sub-Saharan Africa [19].

The emergence of these sub-lineages occurred around the same time as the emergence of other known VOCs and is therefore suggestive of a similar shift in the selective environment in which SARS-CoV-2 was evolving in Egypt, i.e., from naïve susceptible to more resilient hosts, likely due to the mounting immunity from prior infections and later on the commencement of the vaccination [20].

Our results also show that, despite the comprehensive response plan devised by the Egyptian authorities that included various types of public health and social restriction measures to control the pandemic, the case numbers continued to rise. For example, on 19 March 2020, Egypt closed all airports and suspended air travel. Two days later, all social and religious gatherings were suspended, and curfews were imposed until the end of March. Restrictions were followed by campaigns to increase awareness amongst the public [21], and yet, the cases continued to rise.

Our findings show that, as of July 2021, Egypt had experienced three distinct epidemic waves. The first wave coincided with various religious and cultural celebrations in the holy month of Ramadan and the Fitr Islamic Holiday, as well as a season of traditional wedding celebrations and family gatherings characterized by little to no social distancing [3]. Events of this nature, also known as super-spreader events, have been shown to fuel epidemic growth. As such, the full extent of the epidemic during this period remains unknown, due to potential under-reporting as a result of limited testing and large numbers of asymptomatic cases. The results from seroprevalence studies suggest relatively high rates of community transmission. For example, Gomaa et al. (2021) [3] estimated the seroprevalence at 34.8% with a secondary attack rate of 89.8% among the study participants from 290 households between April and October 2020; while a further study on 4313 in-patients in Cairo estimated a seroprevalence at 29.8% with middle aged men, who comprise the largest segment of the Egyptian population at a higher risk of infection [22]. An even greater rate of seroprevalence (46.3%) was estimated among health care workers [23]. The second wave coincided with the resumption of schools and commencement of the cold weather season (October 2020–April 2021), both of which have been associated with a higher likelihood of virus transmission [24]. Similar to the first wave, the third wave was also associated with the start of religious and cultural celebrations. Of the three waves, the first had the highest case counts and associated deaths. The attenuated effects of the second and third waves could be attributed to prevailing immunity from infections in the first wave and the commencement of vaccination during the second wave.

Genomic epidemiology allows for the identification of emerging variants and enhances our understanding of how the mutations carried by individual virus lineages contribute to the relative adaptive advantages they display in relation to other viruses with which they cocirculate. This can in turn inform the implementation of effective pandemic control measures and the development of vaccines and therapeutics.

The C.36 lineage is characterized by S:Q677H. This mutation has also occurred independently in several other SARS-CoV-2 lineages and VUM Eta/B.1.525, suggestive of a host adaptation advantage [25]. Yet, despite its prevalence in Egypt, we observed only minimal viral export to neighboring countries such as Morocco, and found no evidence of regional spread to neighboring countries. This could be attributed to limited sampling among Egypt’s neighbors.

Its continued evolution and accumulation of several further adaptive mutations suggests a shift in the selection landscape likely due to natural and vaccine-induced immunity. The lineage acquired the Spike mutation L452R, also detected in various VOIs and VOCs (including Iota/B.1.526.1, Epsilon/B.1.427/B.1.429, and Delta/B.1.617.2 lineages) and occurs in the receptor binding motif (RBM): the portion of the Spike that mediates contact with ACE2 [26]. Occurrence of the L452R and R346S mutations in the RBD has been associated with reduction in the neutralizing susceptibility to several RBM binding class II monoclonal antibodies (mAbs), as well as convalescent and vaccine sera [27,28,29]. The sub-lineage also acquired the ∆HV69-70 deletion originally detected in the Alpha variant, which is associated with increased viral replication and infectivity [30,31]. Together, these mutations are likely to have conferred a significant adaptive advantage to the sub-lineage. Furthermore, it harbors two additional mutations in the N-terminal domain (NTD), S12F and W152R, which are potentially associated with an antigenic shift [32]. The S12F mutation has been hypothesized to affect cell interactions due to its surface exposure and proximity to the RBD [33]. It has particularly been associated with entry of the spike glycoprotein into the endoplasmic reticulum for viral folding and assembly [34,35], while results from in silico experiments suggest the W152R mutation may weaken interactions between the Spike and neutralizing antibodies [36]. The S477N mutation is known to increase the strength of ACE2 binding [37] while the biological consequences of the A845S mutation remains unknown. Although the Beta/B.1.351 variant which was first detected in South Africa [38] dominated the second waves of most African countries, at the time of writing it had not been detected in Egypt. This could possibly be attributed to under-sampling, compounded by limited viral exchanges with sub-Saharan countries, a known trend between countries in North Africa and those in sub-Saharan Africa [19]. Together, these findings highlight the need for sustained genomic surveillance and the monitoring of viral evolution to inform the public health measures that are intended to control the pandemic.

The emergence and rising to dominance of novel variants can be attributed to super-spreader events and viral evolution in immunocompromised hosts. In this study, we observed the alignment of epidemic waves with seasons of substantial social engagement further supporting this fact. Similar to many other countries in Africa, a substantial burden of chronic non-COVID-19 disease has been reported in Egypt [23,39], a factor leading to large segments of the population having reduced immunocompetence and which might therefore favor the emergence of novel variants [40]. Prioritization of the vaccine initiatives among the elderly and immunosuppressed groups is crucial to prevent the continued evolution of the lineages that are presently circulating within the country into novel variants.

The heterogeneous nature of the epidemic in Egypt also creates a favorable environment for recombination events that have the potential to produce viruses with enhanced adaptive and pathogenic properties. Such inter-lineage recombinants of SARS-CoV-2 with confirmed onward transmission have been reported in the UK, particularly from recombination with the Alpha variant that has also been detected in Egypt [41]. This demonstrates the potential of SARS-CoV-2 to evolve in similar settings and, therefore, the need for the continued close monitoring of recombination events in Egypt. Additionally, public health measures including rapid response to confirmed cases, contact tracing, and the enforcement of self-isolation are crucial to prevent further evolution of the virus.

The main limitations of this study were (i) that most of the analyzed genomic sequences were from a single Egyptian city (i.e., Cairo) and (ii) that the volume of sequencing was lower during the second and third waves than it was during the first wave. These two limitations greatly complicated the reconstruction of the viral transmission dynamics within the country. There is a need for a more robust surveillance strategy in Egypt and this needs to be met with sustainable funding to support genomic epidemiology initiatives and ensure that representative samples are collected across the country at regular time-intervals. A final important limitation of our study was that the epidemiological data needed to contextualize much of the genomic sequencing data was absent, for example, patient travel details that might have been useful to corroborate the inferred importation and exportation events were not generally available. Epidemiological data of this sort is vital because the slow evolutionary rate of the virus at the onset of the pandemic meant that genetic linkages between sequences in different countries could not be used to categorically prove that viral movements occurred between those countries.

In conclusion, the evolution of C.36 into various sub-lineages, some of global interest, reiterates a single important message: that no SARS-CoV-2 lineages should be ignored. Particularly, we have shown that if not controlled, non-VOC lineages can evolve rapidly within a single country to acquire adaptive mutations necessary to sustain community transmission and affect the prognosis of SARS-CoV-2 infections. This message is reinforced with the recent emergence of the Omicron variant suspected to have emerged from the cryptic circulation of B.1.1. lineage viruses from late 2020 in Southern Africa [42]. Furthermore, as Egypt is experiencing a fourth wave of infections, the continued epidemiological interaction of Egypt with the West as evidenced by the exportation events implied by our analyses (most of which were exports to Europe), highlights the need for a concerted united front against the pandemic between the rest of the world and Africa.

## Data Availability

All data used in this study are available on request from the corresponding author.

## Supplementary Materials

The following supporting information can be downloaded at: https://www.mdpi.com/article/10.3390/v14091878/s1, Figure S1. Import and export analysis of SARS-CoV-2 in Egypt. (A) Viral introductions into Egypt from other continents. (B) Viral exports from Egypt to other continents. Table S1: Nextclade report of all Egypt genomes (*n* = 976). Table S2: GISAID acknowledgements of all sequences from GISAID used in this analysis. Table S3: GISAID metadata for all Egyptian strains.
