# Supplementary material for: SARS-CoV-2 Genetic Diversity and Lineage Dynamics in Egypt during the First 18 Months of the Pandemic
Source: Viruses. 2022 Aug 25;14(9):1878. doi: 10.3390/v14091878 (PMC9502207; doi:10.3390/v14091878)
Supplement: Supplementary file 1 [file viruses-14-01878-s001.zip › Supplementary/FigureS1.pdf]

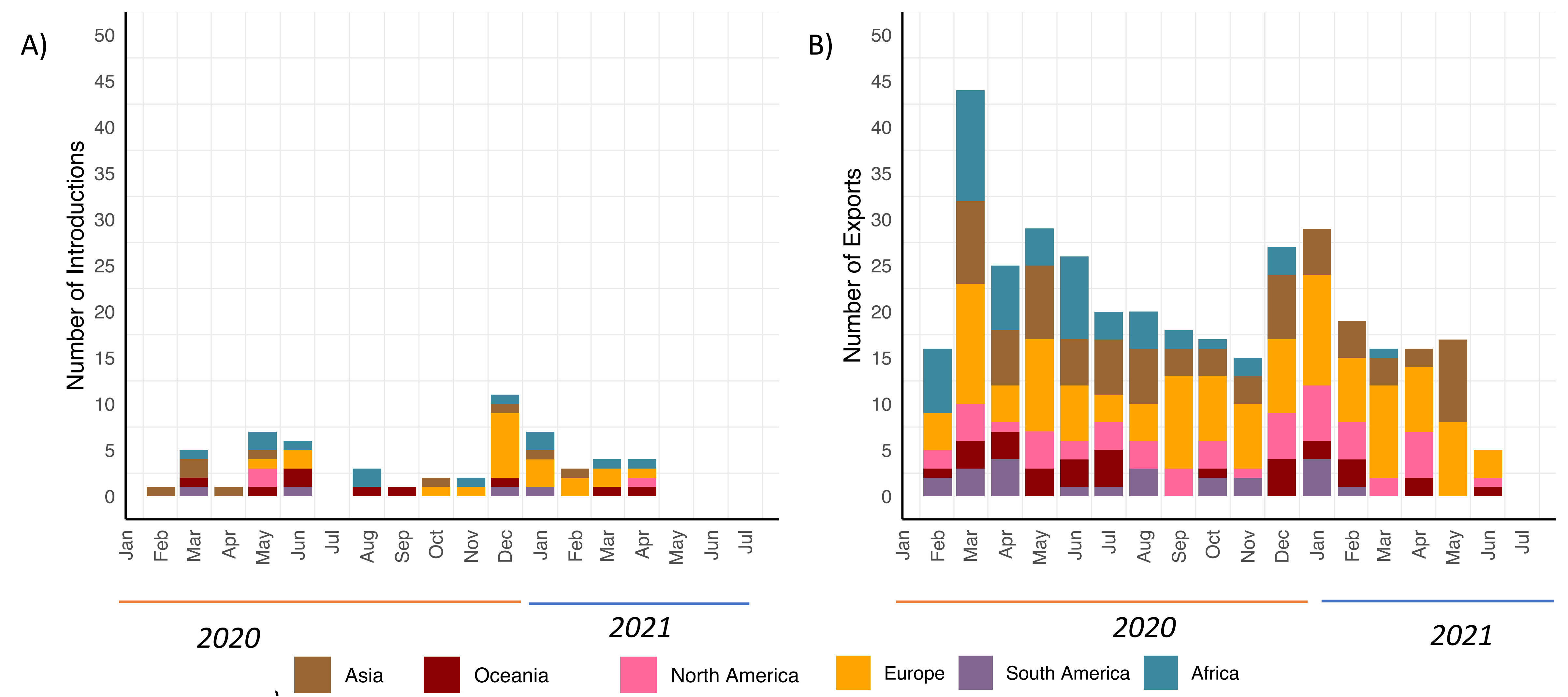
A)
B)
Number of Introductions
Number of Exports
0
5
10
15
20
25
30
35
40
45
50
Jan
Feb
Mar
Apr
May
Jun
Jul
Aug
Sep
Oct
Nov
Dec
2020
2021
Asia
Oceania
North America
Europe
South America
Africa
