# Supplementary material for: SARS-CoV-2 Genetic Diversity and Lineage Dynamics in Egypt during the First 18 Months of the Pandemic
Source: Viruses. 2022 Aug 25;14(9):1878. doi: 10.3390/v14091878 (PMC9502207; doi:10.3390/v14091878)
Supplement: Supplementary file 1 [file viruses-14-01878-s001.zip › Supplementary/TableS2.pdf]

We gratefully acknowledge the following Authors from the Originating laboratories responsible for obtaining the specimens, as well as the Submitting laboratories where the genome data were generated and shared via GISAID, on which this research is based.

All Submitters of data may be contacted directly via www.gisaid.org

Authors are sorted alphabetically.

Accession ID	Originating Laboratory	Submitting Laboratory	Authors
EPI_ISL_1167045	"Dr. Andrija Stampar" Teaching Institute of Public Health, Department of Clinical Microbiology	Istituto di Genomica Applicata	Davide Scaglione; Eleonora Paparelli; Fedrica Cattonaro; Gabriele Magris; Irena Jurman; Jasmina Vranes; Michele Morgante; Slobodanka Radovic; Vera Vendramin
EPI_ISL_993056, EPI_ISL_994661, EPI_ISL_996004	"Dr. Andrija Stampar" Teaching Institute of Public Health, Department of Clinical Microbiology	Institute of Applied Genomics	Federica Cattonaro; Jasmina Vranes; Michele Morgante
EPI_ISL_2438647	"National Center of Expertise" CSEC MH RK in Kostanay region	Reference laboratory for the control of viral infections	Aidar Usserbayev; Aknur Mutaliyeva; Andrey Komissarov; Artem Fadeev; Azamat Kenessov; Bekzhan Maikotov; Gaukhar Nussupbayeva; Madina Tleubergenova; Maria Pisareva; Nazym Tleumbetova
EPI_ISL_2438606	"National Center of Expertise" CSEC MH RK in Kyzylorda Region	Reference laboratory for the control of viral infections	Aidar Usserbayev; Aknur Mutaliyeva; Andrey Komissarov; Artem Fadeev; Azamat Kenessov; Bekzhan Maikotov; Gaukhar Nussupbayeva; Madina Tleubergenova; Maria Pisareva; Nazym Tleumbetova
EPI_ISL_2438655	"National Center of Expertise" CSEC MH RK in West Kazakhstan Region	Reference laboratory for the control of viral infections	Aidar Usserbayev; Aknur Mutaliyeva; Andrey Komissarov; Artem Fadeev; Azamat Kenessov; Bekzhan Maikotov; Gaukhar Nussupbayeva; Madina Tleubergenova; Maria Pisareva; Nazym Tleumbetova
EPI_ISL_2840043	"Sangius" LLP	Reference laboratory for the control of viral infections	Aidar Usserbayev; Aknur Mutaliyeva; Andrey Komissarov; Artem Fadeev; Azamat Kenessov; Bekzhan Maikotov; Gaukhar Nussupbayeva; Madina Tleubergenova; Maria Pisareva; Nazym Tleumbetova
EPI_ISL_632310	1-Laboratory of Microbiology, National Reference Lab, Charles Nicolle Hospital; 2-University of Tunis ElManar, Faculty of Medicine of Tunis, LR99ES09, Tunis, Tunisia	1-Clinical and Experimental Pharmacology Lab, LR16SP02, National Center of Pharmacovigilance, University of Tunis El Manar, Tunis, Tunisia. 2-Neurodegenerative diseases and psychiatric troubles, LR18SP03, Razi Hospital, University of Tunis El Manar, Tunis, Tunisia. 3- Ministry of Health, National Observatory of New and Emerging Diseases, 1006, Tunis, Tunisia	Alia Ben Kahla; Gaies Emna; Ilhem Boutiba-Ben Boubaker; Imen Kacem; Imen Mkada; Jalila Ben Khelil; Maher Kharrat; Mouna Ben Sassi; Mouna Safer; Nissaf Ben Alaya; Riadh Daghfous; Riadh Gouider.; Salma Abid; Sameh Trabelsi; Sana Ferjani; Soumaya Rammeh
EPI_ISL_635059, EPI_ISL_707698, EPI_ISL_733500, EPI_ISL_763065, EPI_ISL_794736	1-Laboratory of Microbiology, National Reference Lab, Charles Nicolle Hospital; 2-University of Tunis ElManar, Faculty of Medicine of Tunis, LR99ES09, Tunis, Tunisia	1-Clinical and Experimental Pharmacology Lab, LR16SP02, National Center of Pharmacovigilance, University of Tunis El Manar, Tunis, Tunisia. 2-Neurodegenerative diseases and psychiatric troubles, LR18SP03, Razi Hospital, University of Tunis El Manar, Tunis, Tunisia. 3- Ministry of Health, National Observatory of New and Emerging Diseases, 1006, Tunis, Tunisia	Alia Ben Kahla; Alia BenKahla; Asma Ferjani; Awatef El Moussi; Gaies Emna; Guedi Ali Barreh; Habiba Ben Romdhane; Hanen El Jebari; Ilhem Boutiba-Ben Boubaker; Imen Kacem; Imen Mkada; Ines Mdini; Jalila Ben Khelil; Maher Kharrat; Mouna Ben Sassi; Mouna Safer; Nissaf Ben Alaya; Riadh Daghfous; Riadh Gouider.; Rouaa Ben Othman; Salma Abid; Sameh Trabelsi; Sana Ferjani; Sarra Chamman; Souissi Amira; Soumaya Rammeh; Zaineb Hamzaoui
EPI_ISL_492072	1. ViroGenetics - BSL3 Laboratory of Virology, Małopolska Centre of Biotechnology, Jagiellonian University; 2. II Department of Internal Medicine, Faculty of Medicine, Jagiellonian University Medical College; 3. Narodowy Instytut Zdrowia Publicznego – Państwowy Zakład Higieny (NIZP-PZH)	1. ViroGenetics - BSL3 Laboratory of Virology, Małopolska Centre of Biotechnology, Jagiellonian University; 2. II Department of Internal Medicine, Faculty of Medicine, Jagiellonian University Medical College; 3. Narodowy Instytut Zdrowia Publicznego – Państwowy Zakład Higieny (NIZP-PZH).	Agnieszka Kołakowska-Kulesza; Aleksandra A. Zasada; Aleksandra Milewska; Ewelina Hallman-Szelińska; Katarzyna Owczarek; Katarzyna Pancer; Katarzyna Zacharczuk; Krzysztof Pyrć; Magdalena Rzeczkowska; Marek Sanak; Natalia Wolaniuk; Paweł P Łabaj; Tomasz Wołkowicz; Wojciech Branicki
EPI_ISL_877029	1.AO Universitaria 'S. Giovanni di Dio e Ruggi D'Aragona, Scuola Medica Salernitana' Hospital / 2.UOC di Virologia e Microbiologia, Università della Campania 'L. Vanvitelli' / 3.AO Universitaria 'Federico II' Napoli Hospital / 4.AORN 'San Giuseppe Moscati' Avellino Hospital / 5.AO 'San Pio - presidio G. Rummo' Benevento Hospital / 6.AO 'Sant'Anna e San Sebastiano' Caserta Hospital / 7.PO 'Maria Santissima Addolorata' Eboli Hospital / 8.Biogem Istituto di Ricerche Genetiche	1. Genome Research Center for Health (CRGS) / 2. Laboratory of Molecular Medicine and Genomics(LMMGe) / 3. Center for Research in Pure and Applied Mathematics (CRMPA)	Alessandro Weisz (Corresponding Author); Alessia Cossu; Aniello Gentile; Annamaria Salvati; Antonello Saccomanno; Arnolfo Petruzziello; Assunta Sellitto; Carlo Ferravante; Domenico Memoli; Domenico Palumbo; Elena Alexandrova; Emilia Vaccaro; Francesca Marciano; Francesca Rizzo (Corresponding Author); Gianluigi Franci; Giorgio Giurato (Corresponding Author); Giovanni Nassa; Giovanni Pecoraro; Giuseppe Fenza; Giuseppe Portella; Gregorio Goffredi; Ilaria Terenzi; Jessica Lamberti; Maddalena Schioppa; Maria Grazia Foti; Maria Landi; Marianna Scrima; Mariarosaria Ingino; Massimiliano Galdiero; Maurizio Fumi; Michele Caraglia; Michele Cennamo; Oriana Strianese; Pasquale Pagliano; Rita Greco; Roberta Tarallo; Sonia Amabile; Teresa Rocco; Valeria Mirici Cappa; Vincenzo Rocco; Viola Melone; Vittoria Letizia; Ylenia D'Agostino
EPI_ISL_1361596, EPI_ISL_1547392	1.AO Universitaria 'S. Giovanni di Dio e Ruggi D'Aragona, Scuola Medica Salernitana' Hospital / 2.UOC di Virologia e Microbiologia, Università della Campania 'L. Vanvitelli' / 3.AO Universitaria 'Federico II' Napoli Hospital / 4.AORN 'San Giuseppe Moscati' Avellino Hospital / 5.AO 'San Pio - presidio G. Rummo' Benevento Hospital / 6.AO 'Sant'Anna e San Sebastiano' Caserta Hospital / 7.PO 'Maria Santissima Addolorata' Eboli Hospital / 8.Biogem Istituto di Ricerche Genetiche / 9. U.O.C. di Genetica Medica e di Laboratorio A.O.R.N., Azienda Ospedaliera di Rilievo Nazionale Antonio Cardarelli, Napoli / 10. Centro di riferimento Oncologico della Basilicata (IRCCS-CROB), Rionero in Vulture (PZ) / 11. Presidio Ospedaliero di Agropoli, Agropoli (SA).	1. Genome Research Center for Health (CRGS) / 2. Laboratory of Molecular Medicine and Genomics(LMMGe) / 3. Center for Research in Pure and Applied Mathematics (CRMPA)	Alessandro Weisz; Alessandro Weisz (Corresponding Author); Alessia Cossu; Andreina Baj; Aniello Gentile; Annamaria Salvati; Antonello Saccomanno; Arnolfo Petruzziello; Assunta Sellitto; Caivano Antonella; Carlo Ferravante; D'Auria Fiorella; De Luca Luciana.; Domenico Memoli; Domenico Palumbo; Edmondo Adorisio; Elena Alexandrova; Emilia Vaccaro; Fausto Sessa; Francesca Marciano; Francesca Rizzo; Francesca Rizzo (Corresponding Author); Francesco Curcio; Gianluigi Franci; Giorgio Dirani; Giorgio Giurato; Giorgio Giurato (Corresponding Author); Giovanni Nassa; Giovanni Pecoraro; Giuseppe Fenza; Giuseppe Portella; Giusy Giordano; Gregorio Goffredi; Ilaria Terenzi; Jessica Lamberti; Maddalena Schioppa; Marcello Ametrano; Maria Grazia Foti; Maria Landi; Marianna Scrima; Mariano Fiorenza; Mariarosaria Ingino; Marina Tarsitano; Massimiliano Chetta; Massimiliano Galdiero; Maurizio Fumi; Michela Iacobellis; Michele Caraglia; Michele Cennamo; Morena D'Avenia; Oriana Strianese; Pasquale Pagliano; Rita Greco; Roberta Tarallo; Rosanna Piluscio; Silvia Zanoli; Simona Semprini; Sonia Amabile; Stefania Marzinotto; Teresa Rocco; Valeria Mirici Cappa; Vincenzo Rocco; Viola Melone; Vittoria Letizia; Vittorio Sambri; Ylenia D'Agostino
EPI_ISL_2140715	2 Military Hospital wc MAA	NHLS/UCT	Arash Iranzadeh; Bruna Galvao; Carolyn Williamson; Deelan Doolabh; Diana Hardie; Innocent Mudau; Kruger Marais; Lynn Tyers; Marvin Hsiao; Stephen Korsman
EPI_ISL_2547411	2 Military Hospital wc MAA	National Health Laboratory Service/University of Cape Town (NHLS/UCT)	Arash Iranzadeh; Bruna Galvao; Carolyn Williamson; Deelan Doolabh; Diana Hardie; Gert Marais; Innocent Mudau; Lynn Tyers; Marvin Hsiao; Stephen Korsman
EPI_ISL_2646244	ACM Bio	Cerba lab	Aude Lessenne; Bénédicte Roquebert; Emmanuel Lecorche; Kader Merah; Laura Verdurme; Patrice Herisson; Sabine Trombert-Paolantoni; Stéphanie Haïm-Boukobza; Thierry Collin
EPI_ISL_2621213	ADILAB	Universidad Nacional de Colombia - Laboratorio Genómico One Health	Andres F. Cardona-Rios; Carlos Franco-Muñoz; Carolina Muñoz-Arango; Celeny Ortiz; Daniel O. Maldonado-Perez; Diego A. Álvarez-Díaz; Hector Alejandro Ruiz-Moreno; Idabely Betancur Ortiz; Jorge E. Osorio; Juan P. Hernandez-Ortiz; Karl A Ciuoderis; Katherine Laiton-Donato; Laura Silvana Perez; Lina M. Hurtado; Marcela Mercado-Reyes; Maria Angélica Maya; Maria Stella López; Rita Almanza Payares; Sandra Ines Cano; Simón Villegas Velásquez
EPI_ISL_2617039, EPI_ISL_2617056	AFRICA_CDC - Angola (Ship 12)	CERI, Centre for Epidemic Response and Innvoation, Stellenbosch University and KRISP, KZN Research Innovation and Sequencing Platform, UKZN.	Afonso P; David K; Emmanuel SJ; Freitas RH; Giandhari J; Inglês L; Lutucuta S; Miranda J; Morais J; Mufinda M; Naidoo Y; Neto Z; Paulo A Carralero RR Paixão JP; Pereira A; Pillay S; Tegally H; Wilkinson E; de Oliveira T
EPI_ISL_918437, EPI_ISL_918438	AIID	Irish Coronavirus Sequencing Consortium-Teagasc Grange	Aljandro Abner Garcia Leon; Calum Walsh; Fiona Crispie; Gabriel Gonzalez; John Kenny; Matthew McCabe; Michael Carr; Patrick Mallon; Paul Cotter
EPI_ISL_2018254	AK State Public Health Lab, State Health Department	Centers for Disease Control and Prevention Division of Viral Diseases, Pathogen Discovery	Alison Laufer Halpin; Ben L. Rambo-Martin; Clinton R. Paden; Dakota Howard; Darlene Wagner; Dave Wentworth; Dhwani Batra; Jasmine Padilla; Justin Lee; Katie Dillon; Krista Queen; Kristen Knipe; Kristine Lacek; Mark Burroughs; Matthew Schmerer; Mili Sheth; Peter Cook; Sam Shepard; Sarah Nobles; Shoshona Le; Suxiang Tong; Vivien Dugan; Yvette Unoarumhi
EPI_ISL_2608418	AKUH Laboratories, Karachi, Pakistan	AKUH Laboratories, Karachi, Pakistan	Akbar Kanji; Asghar Nasir; Rumina Hasan; Safina A. Razzak; Sayed Ali Raza; Zahra Hasan
EPI_ISL_2383982	AL Dept. of Public Health Bureau of Clinical Laboratories	Centers for Disease Control and Prevention Division of Viral Diseases, Pathogen Discovery	Alison Laufer Halpin; Ben L. Rambo-Martin; Clinton R. Paden; Dakota Howard; Darlene Wagner; Dave Wentworth; Dhwani Batra; Jasmine Padilla; Justin Lee; Katie Dillon; Krista Queen; Kristen Knipe; Kristine Lacek; Mark Burroughs; Matthew Schmerer; Mili Sheth; Peter Cook; Sam Shepard; Sarah Nobles; Shoshona Le; Suxiang Tong; Vivien Dugan; Yvette Unoarumhi
EPI_ISL_2868624	ALBANY MEDICAL CENTER	Wadsworth Center, New York State Department of Health	Alexis Russell; Catharine Prussing; Daryl M. Lamson; Erasmus Schneider; Erica Lasek-Nesselquist; John Kelly; Jonathan Plitnick; Kirsten St. George; Matthew Shudt; Melissa A Leisner; Navjot Singh
EPI_ISL_2493041	AMPATH	CERI, Centre for Epidemic Response and Innvoation, Stellenbosch University and KRISP, KZN Research Innovation and Sequencing Platform, UKZN.	Emmanuel SJ; Giandhari J; Khan S; Lessells R; Mdlalose K; Naidoo Y; Pillay S; Ramphal U; Tegally H; Wilkinson E; York D; de Oliveira T
EPI_ISL_2086992, EPI_ISL_2087012, EPI_ISL_2087018	AMPATH LABORATORIES	National Institute for Communicable Diseases of the National Health Laboratory Service	Amoako DG; Bhiman JN; Ismail A; Mahlangu B; Mohale T; Ntuli N; Scheepers C
EPI_ISL_2658283	AREA DE SALUD ALAJUELITA	Inciensa, Instituto Costarricense de Investigación y Enseñanza en Nutrición y Salud	Adriana Godínez; Claudio Soto-Garita; Estela Cordero; Francisco Duarte; Hebleen Porras; José Luis Vargas; Mariela Gutiérrez & Joselyn Prado; Melany Calderón
EPI_ISL_3037812	AREA DE SALUD BAGACES	Inciensa, Instituto Costarricense de Investigación y Enseñanza en Nutrición y Salud	Adriana Godínez; Caterina Guzmán; Claudio Soto-Garita; Estela Cordero; Francisco Duarte; Hebleen Porras; José Luis Vargas; Mariela Gutiérrez Joselyn Prado; Melany Calderón; Nazareth Ruiz & Adriana Bermúdez
EPI_ISL_3037817	AREA DE SALUD BELEN-FLORES - CLINICA DR. JORGE VOLIO	Inciensa, Instituto Costarricense de Investigación y Enseñanza en Nutrición y Salud	Adriana Godínez; Caterina Guzmán; Claudio Soto-Garita; Estela Cordero; Francisco Duarte; Hebleen Porras; José Luis Vargas; Mariela Gutiérrez Joselyn Prado; Melany Calderón; Nazareth Ruiz & Sandra Mora Leitón
EPI_ISL_2272981	AREA DE SALUD CARRILLO	Inciensa, Instituto Costarricense de Investigación y Enseñanza en Nutrición y Salud	Adriana Godínez; Claudio Soto-Garita; Estela Cordero; Francisco Duarte; Hebleen Porras; Joselyn Prado & Adriana Bemúdez Espinoza; José Luis Vargas; Mariela Gutiérrez; Melany Calderón
EPI_ISL_2658271	AREA DE SALUD CHOMES - MONTEVERDE	Inciensa, Instituto Costarricense de Investigación y Enseñanza en Nutrición y Salud	Adriana Godínez; Claudio Soto-Garita; Estela Cordero; Francisco Duarte; Hebleen Porras; Joselyn Prado & Javier Zárate-León; José Luis Vargas; Mariela Gutiérrez; Melany Calderón

EPI_ISL_3037821	AREA DE SALUD COTO BRUS	Inciensa, Instituto Costarricense de Investigación y Enseñanza en Nutrición y Salud	Adriana Godínez; Caterina Guzmán; Claudio Soto-Garita; Estela Cordero; Francisco Duarte; Hebleen Porras; José Luis Vargas; Mariela Gutiérrez Joselyn Prado; Melany Calderón; Nazareth Ruiz & María Fernanda Matamoros
EPI_ISL_2502748	AREA DE SALUD DESAMPARADOS 2 (COOPESALUD)	Inciensa, Instituto Costarricense de InvestigaciOn y Enseñanza en NutriciOn y Salud	Adriana Godínez; Claudio Soto-Garita; Estela Cordero; Francisco Duarte; Hebleen Porras; Jose Luis Vargas; Joselyn Prado & Paola Sánchez-Traña; Mariela Gutierrez; Melany CalderOn
EPI_ISL_3026033	AREA DE SALUD Desamparados 1 - CLINICA DR. MARCIAL FALLAS [GRIFO ALTO/Desamparados/San Jose]	Inciensa, Instituto Costarricense de Investigación y Enseñanza en Nutrición y Salud	Adriana Godínez; Caterina Guzmán; Claudio Soto-Garita; Estela Cordero; Francisco Duarte; Hebleen Porras; Joselyn Prado; José Luis Vargas; Mariela Gutiérrez; Melany Calderón; Nazareth Ruíz
EPI_ISL_3026022	AREA DE SALUD EL GUARCO [EL GUARCO/CARTAGO]	Inciensa, Instituto Costarricense de Investigación y Enseñanza en Nutrición y Salud	Adriana Godínez; Caterina Guzmán; Claudio Soto-Garita; Estela Cordero; Francisco Duarte; Hebleen Porras; Joselyn Prado; José Luis Vargas; Mariela Gutiérrez; Melany Calderón; Nazareth Ruíz & Mónica Charpentier
EPI_ISL_3026027	AREA DE SALUD FORTUNA [San Carlos/ALAJUELA]	Inciensa, Instituto Costarricense de Investigación y Enseñanza en Nutrición y Salud	Adriana Godínez; Caterina Guzmán; Claudio Soto-Garita; Estela Cordero; Francisco Duarte; Hebleen Porras; Joselyn Prado; José Luis Vargas; Mariela Gutiérrez; Melany Calderón; Nazareth Ruíz & Carolina Arrieta
EPI_ISL_2828000	AREA DE SALUD GARABITO	Inciensa, Instituto Costarricense de Investigación y Enseñanza en Nutrición y Salud	Adriana Godínez; Claudio Soto-Garita; Estela Cordero; Francisco Duarte; Hebleen Porras; Joselyn Prado & María José Gómez; José Luis Vargas; Mariela Gutiérrez; Melany Calderón
EPI_ISL_2502755	AREA DE SALUD GOICOECHEA 2 - CLINICA DR. JIMENEZ NUÑEZ	Inciensa, Instituto Costarricense de InvestigaciOn y Enseñanza en NutriciOn y Salud	Adriana Godínez; Claudio Soto-Garita; Estela Cordero; Francisco Duarte; Hebleen Porras; Jose Luis Vargas; Joselyn Prado & Alberto Madrigal-Vega; Mariela Gutierrez; Melany CalderOn
EPI_ISL_2502736	AREA DE SALUD GUATUSO	Inciensa, Instituto Costarricense de InvestigaciOn y Enseñanza en NutriciOn y Salud	Adriana Godínez; Claudio Soto-Garita; Estela Cordero; Francisco Duarte; Hebleen Porras; Jose Luis Vargas; Joselyn Prado & Francisco ChacOn-Valverde; Mariela Gutierrez; Melany CalderOn
EPI_ISL_3026031	AREA DE SALUD Guatuso [Guatuso/ALAJUELA]	Inciensa, Instituto Costarricense de Investigación y Enseñanza en Nutrición y Salud	Adriana Godínez; Caterina Guzmán; Claudio Soto-Garita; Estela Cordero; Francisco Duarte; Hebleen Porras; Joselyn Prado; José Luis Vargas; Mariela Gutiérrez; Melany Calderón; Nazareth Ruíz & Francisco Chacón
EPI_ISL_2272966	AREA DE SALUD HATILLO - CLINICA DR. SOLON NUÑEZ	Inciensa, Instituto Costarricense de Investigación y Enseñanza en Nutrición y Salud	Adriana Godínez; Claudio Soto-Garita; Estela Cordero; Francisco Duarte; Hebleen Porras; Joselyn Prado & Roberto Brilla-Ferrer; José Luis Vargas; Mariela Gutiérrez; Melany Calderón
EPI_ISL_2502739	AREA DE SALUD MATA REDONDA -HOSPITAL -CLINICA DR. MORENO CAÑAS	Inciensa, Instituto Costarricense de InvestigaciOn y Enseñanza en NutriciOn y Salud	Adriana Godínez; Claudio Soto-Garita; Estela Cordero; Francisco Duarte; Hebleen Porras; Jose Luis Vargas; Joselyn Prado & Carolina Duarte; Mariela Gutierrez; Melany CalderOn
EPI_ISL_3037838	AREA DE SALUD MATA REDONDA-HOSPITAL - CLINICA DR. MORENO CAÑAS	Inciensa, Instituto Costarricense de Investigación y Enseñanza en Nutrición y Salud	& Nazareth Ruiz; Adriana Godínez; Caterina Guzmán; Claudio Soto-Garita; Estela Cordero; Francisco Duarte; Hebleen Porras; José Luis Vargas; Mariela Gutiérrez Joselyn Prado; Melany Calderón
EPI_ISL_2502720	AREA DE SALUD MORAVIA	Inciensa, Instituto Costarricense de InvestigaciOn y Enseñanza en NutriciOn y Salud	Adriana Godínez; Claudio Soto-Garita; Estela Cordero; Francisco Duarte; Hebleen Porras; Jose Luis Vargas; Joselyn Prado & Mariel LOpez; Mariela Gutierrez; Melany CalderOn
EPI_ISL_3037845	AREA DE SALUD PAQUERA	Inciensa, Instituto Costarricense de Investigación y Enseñanza en Nutrición y Salud	Adriana Godínez; Caterina Guzmán; Claudio Soto-Garita; Estela Cordero; Francisco Duarte; Hebleen Porras; José Luis Vargas; Mariela Gutiérrez Joselyn Prado; Melany Calderón; Nazareth Ruiz & Andre Moreno Carvajal
EPI_ISL_2502757	AREA DE SALUD PARAISO-CERVANTES	Inciensa, Instituto Costarricense de InvestigaciOn y Enseñanza en NutriciOn y Salud	Adriana Godínez; Claudio Soto-Garita; Estela Cordero; Francisco Duarte; Hebleen Porras; Jose Luis Vargas; Joselyn Prado & MOnica Charpentier; Mariela Gutierrez; Melany CalderOn
EPI_ISL_2502750	AREA DE SALUD PEREZ ZELEDON	Inciensa, Instituto Costarricense de InvestigaciOn y Enseñanza en NutriciOn y Salud	Adriana Godínez; Claudio Soto-Garita; Estela Cordero; Francisco Duarte; Hebleen Porras; Jose Luis Vargas; Joselyn Prado & Fabricio Aguilar; Mariela Gutierrez; Melany CalderOn
EPI_ISL_3037819	AREA DE SALUD PEREZ ZELEDON	Inciensa, Instituto Costarricense de Investigación y Enseñanza en Nutrición y Salud	Adriana Godínez; Caterina Guzmán; Claudio Soto-Garita; Estela Cordero; Francisco Duarte; Hebleen Porras; José Luis Vargas; Mariela Gutiérrez Joselyn Prado; Melany Calderón; Nazareth Ruiz & Yendry Ramírez
EPI_ISL_2658272, EPI_ISL_2658275	AREA DE SALUD SAN RAFAEL	Inciensa, Instituto Costarricense de Investigación y Enseñanza en Nutrición y Salud	Adriana Godínez; Claudio Soto-Garita; Estela Cordero; Francisco Duarte; Hebleen Porras; Joselyn Prado & Javier Zárate-León; José Luis Vargas; Mariela Gutiérrez; Melany Calderón
EPI_ISL_2828008	AREA DE SALUD SAN RAMON	Inciensa, Instituto Costarricense de Investigación y Enseñanza en Nutrición y Salud	Adriana Godínez; Claudio Soto-Garita; Estela Cordero; Francisco Duarte; Hebleen Porras; José Luis Vargas; Mariela Gutiérrez & Joselyn Prado; Melany Calderón
EPI_ISL_2272993	AREA DE SALUD SANTO DOMINGO	Inciensa, Instituto Costarricense de Investigación y Enseñanza en Nutrición y Salud	Adriana Godínez; Claudio Soto-Garita; Estela Cordero; Francisco Duarte; Hebleen Porras; Joselyn Prado & Margarita Lee-Lui; José Luis Vargas; Mariela Gutiérrez; Melany Calderón
EPI_ISL_2502744	AREA DE SALUD SIQUIRRES	Inciensa, Instituto Costarricense de InvestigaciOn y Enseñanza en NutriciOn y Salud	Adriana Godínez; Claudio Soto-Garita; Estela Cordero; Francisco Duarte; Hebleen Porras; Jose Luis Vargas; Joselyn Prado & Karla LOpez; Mariela Gutierrez; Melany CalderOn
EPI_ISL_2103384, EPI_ISL_2272958, EPI_ISL_2272977	AREA DE SALUD SIQUIRRES	Inciensa, Instituto Costarricense de Investigación y Enseñanza en Nutrición y Salud	Adriana Godínez; Caterina Guzmán; Claudio Soto-Garita; Estela Cordero; Francisco Duarte; Hebleen Porras; Joselyn Prado; Joselyn Prado & Ileana Chaves-Peraza; José Luis Vargas; Mariela Gutiérrez; Melany Calderón; Nazareth Ruiz & Ileana Chaves-Peraza
EPI_ISL_3037825	AREA DE SALUD TURRIALBA-JIMENEZ	Inciensa, Instituto Costarricense de Investigación y Enseñanza en Nutrición y Salud	Adriana Godínez; Caterina Guzmán; Claudio Soto-Garita; Estela Cordero; Francisco Duarte; Hebleen Porras; José Luis Vargas; Mariela Gutiérrez Joselyn Prado; Melany Calderón; Nazareth Ruiz & Mónica Charpentier
EPI_ISL_2502758	AREA DE SALUD ZAPOTE-CATEDRAL - CLINICA DR. CARLOS DURAN	Inciensa, Instituto Costarricense de InvestigaciOn y Enseñanza en NutriciOn y Salud	Adriana Godínez; Claudio Soto-Garita; Estela Cordero; Francisco Duarte; Hebleen Porras; Jose Luis Vargas; Joselyn Prado & Marco Cháves-Otárola; Mariela Gutierrez; Melany CalderOn
EPI_ISL_1067612	AREA DE SALUD ZAPOTE-CATEDRAL - CLINICA DR. CARLOS DURAN	Inciensa, Instituto Costarricense de Investigación y Enseñanza en Nutrición y Salud	Adriana Godínez; Caterina Guzmán; Claudio Soto-Garita; Estela Cordero; Francisco Duarte; Hebleen Porras; Melany Calderón; Nazareth Ruiz & Melissa Carazo-Gutiérrez
EPI_ISL_2695444	ARS Algarve - Laboratorio Laura Ayres	Instituto Nacional de Saude (INSA)	Borges et al
EPI_ISL_517616, EPI_ISL_518802	Academic Hospital Paramaribo	Erasmus Medical Center	Bas Oude Munnink; Dion Gajadin; Ed Ijzerman; Emmanuelle Munger; Gary Gummels; Ingrid Krishnadath; Lycke Woittiez; Marion Koopmans; Mireille Van de Veer; Princes Wongsowidjojo; Radjesh Ori; Rohma Banwari; Stephen Vreden
EPI_ISL_2833781	Adan Hospital	Kuwait Cancer Control Center	Mona Alateeqi; Shakir Bahzad
EPI_ISL_1445665, EPI_ISL_1650426, EPI_ISL_1689082, EPI_ISL_1702837, EPI_ISL_1737563, EPI_ISL_1835211, EPI_ISL_1837090, EPI_ISL_1837760, EPI_ISL_2040167, EPI_ISL_2040637, EPI_ISL_2040648, EPI_ISL_2040827, EPI_ISL_2041273, EPI_ISL_2042434, EPI_ISL_2090019, EPI_ISL_2090085, EPI_ISL_2146197, EPI_ISL_2148186, EPI_ISL_2148720, EPI_ISL_2148847, EPI_ISL_2150374, EPI_ISL_2158859, EPI_ISL_2159079, EPI_ISL_2159334, EPI_ISL_2180676, EPI_ISL_2187388, EPI_ISL_2201288, EPI_ISL_2202065, EPI_ISL_2203267, EPI_ISL_2243536, EPI_ISL_2280370, EPI_ISL_2368234, EPI_ISL_2370070, EPI_ISL_2421780, EPI_ISL_2489181, EPI_ISL_2489215, EPI_ISL_2528269, EPI_ISL_2600307, EPI_ISL_2687055, EPI_ISL_2687316, EPI_ISL_2687343, EPI_ISL_2709524			
see above	Aegis Sciences Corporation	Centers for Disease Control and Prevention Division of Viral Diseases, Pathogen Discovery	Adrian Paskey; Alec Vest; Benjamin Rambo-Martin; Christopher Gulvick; Clinton R. Paden; Cyndi Clark; Dakota Howard; Darlene Wagner; Dhwani Batra; Dillon Nall; Duncan MacCannell; Ethan Sanders; Holly Houdeshell; Jason Caravas; Kara Moser; Matthew Hardison; Matthew Schmerer; Ola Kvalvaag; Patrick Campbell; Peter W. Cook; Rob Case; Scott Sammons; Shatavia Morrison; Shaun Westlund; Vikramsinha Ghorpade; Yvette Unoarumhi
EPI_ISL_2617050	Africa_CDC - Malawi P4 S2	CERI, Centre for Epidemic Response and Innvoation, Stellenbosch University and KRISP, KZN Research Innovation and Sequencing Platform, UKZN.	Auld A; Chilima B; Chiwaula M; Emmanuel SJ; Giandhari J; Kaba M; Kampira E; Kasambara W; Kim L; Lessells R; Maida A; Mvula B; Mwangomba W; Naidoo Y; Panja L; Pillay S; Tegally H; Wadonda N; Wilkinson E; de Oliveira T
EPI_ISL_2617090	Africa_CDC - Malawi P6 S2	CERI, Centre for Epidemic Response and Innvoation, Stellenbosch University and KRISP, KZN Research Innovation and Sequencing Platform, UKZN.	Auld A; Chilima B; Chiwaula M; Emmanuel SJ; Giandhari J; Kaba M; Kampira E; Kasambara W; Kim L; Lessells R; Maida A; Mvula B; Mwangomba W; Naidoo Y; Panja L; Pillay S; Tegally H; Wadonda N; Wilkinson E; de Oliveira T
EPI_ISL_2360250, EPI_ISL_2360251	Afzalipoor Hospital	National Influenza Center	A Nejati; F Ajaminejad and T Mokhtari Azad; J Yavarian; K Sadeghi; N Ghavvami; NZ Shafiei Jandaghi; V Salimi
EPI_ISL_528935	Agenzia di Tutela della Salute di Bergamo	Istitituto Zooprofilattico Sperimentale dell'Abruzzo e Molise "G.Caporale"	Ancora M; Cammà C; Curini V; Di Domenico M; Di Pasquale A; Lorusso A; Mangone I; Marcacci M; Puglia I; Rinaldi A; Savini G.
EPI_ISL_500783, EPI_ISL_590952, EPI_ISL_775387, EPI_ISL_1820053, EPI_ISL_2333261	Akershus University Hospital, Department for Microbiology and Infectious Disease Control	Norwegian Institute of Public Health, Department of Virology	'Kathrine Stene-Johansen; Atiya R Ali; Debech Nadia; Engebretsen Serina Beate; Garcia Llorente Ignacio; Hilde Elshaug; Hilde Vollan; Jon Bråte; Kamilla Heddeland Instefjord; Karoline Bragstad; Kathrine Stene-Johansen; Line Victoria Moen; Marie Paulsen Madsen; Olav Hungnes; Pedersen Benedikte Nevjen; Rasmus Riis Kopperud
EPI_ISL_625613	Alameda County Public Health Lab	Chan-Zuckerberg Biohub	CZB Cliahub Consortium
EPI_ISL_1483588, EPI_ISL_2626698, EPI_ISL_2987166	Alaska State Virology Laboratory	Alaska State Virology Laboratory	Elva House; Jack Chen; Jacob Zidek; Lisa Smith; Ph.D.; Stephanie DeRonde
EPI_ISL_2163060, EPI_ISL_2169909	Alberta Precision Labs (APL)	Public Health Agency of Canada (PHAC) National Microbiology Laboratory	Buss; Croxen M; Deo A; Dieu P; E; Ferrato C; Gill K; Khan F; Koleva P; Li V; Lloyd C; Lynch T; Ma R; Murphy S; Pabbaraju K; Shokoples S; Thayer J; Tipples G; Whitehouse M; Wong A; Yu C; Zelyas N
EPI_ISL_2375924	Albow Gardens Clinic wc ALB	NHLS/UCT	Arash Iranzadeh; Bruna Galvao; Carolyn Williamson; Deelan Doolabh; Diana Hardie; Innocent Mudau; Kruger Marais; Lynn Tyers; Marvin Hsiao; Stephen Korsman
EPI_ISL_1385811	Alfa Diagnostica LLC	ONCOGENE LLC	ONCOGENE LLC
EPI_ISL_508174	All india institute of Medical Sciences Rishikesh	National Institute of Biomedical Genomics	Amit Mangla; Arindam Maitra; Deepjyoti Kalita; Ravi Kant; Saumitra Das
EPI_ISL_2375992	Alma CDC wc AHC	NHLS/UCT	Arash Iranzadeh; Bruna Galvao; Carolyn Williamson; Deelan Doolabh; Diana Hardie; Innocent Mudau; Kruger Marais; Lynn Tyers; Marvin Hsiao; Stephen Korsman
EPI_ISL_2854052	Ampang Hospital	Institute for Medical Research, Infectious Disease Research Centre, National Institutes of Health, Ministry of Health Malaysia	Anasir MI; Azizan MA; Kamel K; Mohd Zawawi Z; Ramly N; Robert F; Suppiah J; Thayan R
EPI_ISL_2727238	Ampath Greenacres Hospital	KRISP, KZn Research Innovation and Sequencing Platform	Giandhari Jennifer; Naidoo Yeshnee; Pillay Sureshnee; San James; Tegally Houriiyah; Tshabuila Derek; Wilkinson Eduan; Yajna Ramphal; de Oliveira Tulio
EPI_ISL_2178783, EPI_ISL_2178785, EPI_ISL_2178788, EPI_ISL_2178789, EPI_ISL_2178793, EPI_ISL_2178794, EPI_ISL_2193359			
see above	Ampath Laboratories	National Institute for Communicable Diseases of the National	Amoako DG; Bhiman JN; Glass A; Gottberg A; Mahlangu B; Marshall T; Mohale T; Ntuli N; Scheepers C; Viana R

		Health Laboratory Service	
EPI_ISL_1324593	Anteja laboratorija (UAB Diagnostikos laboratorija)	Vilnius University Hospital Santaros Klinikos, Center of Laboratory Medicine	Daniel Naumovas; Dovile Ezerskyte; Gytis Dudas; Ingrida Olendraite; Laimonas Griskevicius; Ligita Raugaite; Mindaugas Stoskus; Monika Katenaite; Rimvydas Norvilas
EPI_ISL_1483700	Area of Virology, Serology and Virology Division (SAVID), New South Wales Health Pathology Randwick	Area of Virology, Serology and Virology Division (SAVID), New South Wales Health Pathology Randwick	Au, J.; Bull, R.; Deveson, I.; Foster, C.; Jean, T.; Rawlinson, W.; Ruiz Silva, M.; Van Haal, S.
EPI_ISL_678312, EPI_ISL_678327, EPI_ISL_678328, EPI_ISL_707898, EPI_ISL_717702, EPI_ISL_1121974, EPI_ISL_1121975, EPI_ISL_1494718, EPI_ISL_1494722, EPI_ISL_1543926, EPI_ISL_1615594, EPI_ISL_1633353, EPI_ISL_2650011			
see above	Area of Virology, Serology and Virology Division (SAViD), New South Wales Health Pathology Randwick	Virology Research Laboratory; Area of Virology, Serology and Virology Division (SAViD), New South Wales Health Pathology Randwick	Au, J.; Bull, R.; Deveson, I.; Foster, C.; Rawlinson, W.; Ruiz Silva, M.; Van Hal, S.
EPI_ISL_2931537, EPI_ISL_2931586	Arkansas Public Health Laboratory, Arkansas Department of Health	University of Minnesota Genomics Center	Corbin Dirkx; Daryl M. Gohl; Jaquelyn Kuriger-Laber; John Garbe
EPI_ISL_1315065	Armed Forces Medical Research Laboratories and Blood Bank, Egypt	Department of Microbial Biotechnology, Genetic Engineering Division, National Research Centre	Ahmed Elsayed; Ahmed Taha; Ayman Farghaly; Mohamed Khalifa; Mohamed Shemis; Reham Dawood
EPI_ISL_1315064	Armed Forces Medical Research Laboratories and Blood Bank, Egypt	Department of Microbial Biotechnology, Genetic Engineering Division, National Research Centre,	Ahmed Elsayed; Ahmed Taha; Ayman Farghaly; Mohamed Khalifa; Mohamed Shemis; Reham Dawood
EPI_ISL_2849763	Artic Network workflow-EPI2ME labs	King Khalid Bin Abdul Aziz street, South Surra, Kuwait City	Anfal Al-Adwani; Ebaa Al-Awadhi; Hussain Safar; Nada Madi
EPI_ISL_2423528	Atlanta VA Medical Center	Genomics and Discovery, Respiratory Viruses Branch, Division of Viral Diseases, Centers for Disease Control and Prevention	Adam Retchless; Anna Kelleher; Anna Uehara; Brian Lynch; Clinton R. Paden; Dhwani Batra; Haibin Wang; Han Jia Justin Ng; Jasmine Padilla; Jing Zhang; Justin Lee; Krista Queen; Mark Burroughs; Mili Sheth; Morgan Davis; Peter Cook; Rachel Marine; Sarah Nobles; Suxiang Tong; Tara Coalter; Yan Li; Ying Tao
EPI_ISL_413490	Auckland Hospital	Institute of Environmental Science and Research (ESR)	Erasmus Smit; Gary McAuliffe; Joep de Ligt; Lauren Jelly; Matt Storey; Matthew Blakiston; Sally Roberts; Xiaoyun Ren
EPI_ISL_853962, EPI_ISL_853964, EPI_ISL_1671058, EPI_ISL_1840870, EPI_ISL_1840872, EPI_ISL_1840952, EPI_ISL_1840962, EPI_ISL_2137339, EPI_ISL_2232813, EPI_ISL_2232857, EPI_ISL_2232886, EPI_ISL_2232895, EPI_ISL_2324297, EPI_ISL_2324298, EPI_ISL_2324300, EPI_ISL_2427162, EPI_ISL_2485020, EPI_ISL_2485057, EPI_ISL_2485157, EPI_ISL_2616224, EPI_ISL_2616312, EPI_ISL_2616365, EPI_ISL_2657417, EPI_ISL_2757990, EPI_ISL_2757992, EPI_ISL_2758026, EPI_ISL_2887420, EPI_ISL_2887619, EPI_ISL_2887630, EPI_ISL_2985444, EPI_ISL_3039920, EPI_ISL_3039925			
see above	Austrian Agency for Health and Food Safety (AGES)	Bergthaler laboratory, CeMM Research Center for Molecular Medicine of the Austrian Academy of Sciences	Alexander Lercher; Alexandra Popa; Andreas Bergthaler; Anna Schedl; Bekir Erguner; Benedikt Agerer; Christoph Bock; Christoph Bock; Fabian Amman; Jakob-Wendelin Genger; Jan Laine; Lukas Endler; Maelle Le Moing; Martin Senekowitsch; Martin Senekowitsch; Matthew Thornton; Michael Schuster; Michael Schuster; Petr Triska; Thomas Penz
EPI_ISL_1670599, EPI_ISL_1919625	Azienda Ospedaliera Terni	Istituto Zooprofilattico Sperimentale dell'Abruzzo e Molise "G. Caporale"	Ancora M; Calistri P; Cammà C; Caporale M; Curini V; Delli Compagni E; Di Domenico M; Di Lollo Valeria; Di Pasquale A; Lorusso A; Mangone I; Marcacci M; Palumbo M; Puglia I; Rinaldi A; Savini G; Scaccetti A; Scialabba S
EPI_ISL_1222791, EPI_ISL_1489770	Azienda Sanitaria dell'Alto Adige Laboratorio Aziendale di Microbiologia e Virologia	Istituto di Genomica Applicata	Davide Scaglione; Eleonora Paparelli; Elisa Masi; Elisabetta Giacobazzi; Elisabetta Pagani; Gabriele Magris; Irena Jurman; Irene Bianconi; Michele Morgante; Stefanie Wieser; Vera Vendramin
EPI_ISL_467044	B.J. Medical College and Civil hospital	Gujarat Biotechnology Research Centre	; A M Kadri; Ankit Hinsu; Apurvasinh Puvar; Chaitanya Joshi; Dinesh Kumar; Harsh Bakshi; Janvi Raval; Kamlesh J Upadhyay; Komal Patel; Labdhi Pandya; Madhvi Joshi; Maharshi Pandya; Monika Gandhi; Nidhi Patel; Nirav Mungalpara; Nitin Savaliya; Pinal Trivedi; Pranay Shah; Pritesh Sabara; Priti Pandita; R D Dixit; Raghawendra Kumar; Snehal Bagatharia; Tejas Shah; Zarna Patel; Zuber Saiyed
EPI_ISL_2710311	BARC / Lancet	KRISP, KZN Research Innovation and Sequencing Platform	Giandhari Jennifer; Naidoo Yeshnee; Pillay Sureshnee; San James; Sisonke; Tegally Houriiyah; Tshabuila Derek; Wilkinson Eduan; Yajna Ramphal; de Oliveira Tulio
EPI_ISL_2842230	BBMP Urban	INSACOG-KA, NIMHANS	Ananthapadmanabha Kotambail; Anita S Desai; Anson Kunjumon George; Chetan G K; Chitra Pattabiraman; Darshan Sreenivas; Ellango Ramasamy; Gautham Arunachal Udupi; Mahesh Kumar.C.S; Sony Sharma; V Ravi
EPI_ISL_1169048	BBTKLPP Banjarbaru, Kalimantan Selatan	National Institute of Health Research and Development	Arie Ardiansyah Nugraha; Hana Apsari Pawestri; Hartanti Dian Ikawati; Kartika Dewi Puspa; Nelly Puspandari; Subangkit; Vivi Setiawaty
EPI_ISL_415577, EPI_ISL_418828, EPI_ISL_968754, EPI_ISL_974610, EPI_ISL_974630, EPI_ISL_974656, EPI_ISL_974661, EPI_ISL_975034, EPI_ISL_2499685, EPI_ISL_2522213, EPI_ISL_2530954			
see above	BCCDC Public Health Laboratory	BCCDC Public Health Laboratory	Ana Pacagnella; Choi; Corrinne Ng; Dan Fornika; Gilmour; Harrigan; Hoang; John Tyson; Kamelian; Kim Macdonald; Kimia Kamelian; Krajden; Lapointe; Lee; Levett; Li; Linda Hoang; Loman; Loretta Janz; Mel Krajden; Prystajecky; Prystajecky Natalie; Quick; Robert Azana; Robert Azana Terry Snutch; Sekirov; Shannon Russell; Snutch; Tyson
EPI_ISL_3032332, EPI_ISL_3032333	BIOFAST	Instituto Butantan	Antonio Jorge Martins; Claudia Renata dos Santos Barros; David Schlesinger; Debora Botequio Moretti; Dimas Tadeu Covas; Elaine Cristina Marqueze; Elaine Vieira Santos; Evandra Strazza Rodrigues; Heidge Fukumasu; Jayme Augusto de Souza-Neto; José Salvatore Leister Patané; Luiz Alcantara; Luiz Lehmann Coutinho; Maria Carolina Elias; Maurício Lacerda Nogueira; Rafael dos Santos Bezerra; Raul Machado Neto; Rejane Maria Tommasini Grotto; Ricardo Haddad; Sandra Coccuzzo Sampaio Vessoni; Simone Kashima; Svetoslav Nanev Slavov; Vincent Louis Viala
EPI_ISL_2933674	BIOMNIS LYON	CNR Virus des Infections Respiratoires - France SUD	Antonin Bal; Bruno Lina; Gregory Destras; Gwendolyne Burfin; Hadrien Regue; Laurence Josset; Martine Valette; Quentin Semanas
EPI_ISL_2141965	BIOR	Latvian Biomedical Research and Study Centre	Daina Pule; Davids Fridmanis; Elina Dimina; Guntars Zarins; Irena Meistere; Ivars Silamikelis; Janis Klovins; Janis Pjalkovskis; Jurijs Perevoscikovs; Kaspars Megnis; Laila Silamikele; Lauma Freimane; Laura Ansone; Liga Birzniece; Monta Ustinova; Nikita Zrelovs; Uga Dumpis; Una Krumina; Vita Rovite
EPI_ISL_859567, EPI_ISL_859573, EPI_ISL_859607, EPI_ISL_859642, EPI_ISL_859646, EPI_ISL_859668, EPI_ISL_859695, EPI_ISL_859735, EPI_ISL_859896, EPI_ISL_859900, EPI_ISL_859910, EPI_ISL_859951, EPI_ISL_860061, EPI_ISL_860068, EPI_ISL_860081			
see above	BTC, Khalifa University	BTC, Khalifa University	Al Safar et al
EPI_ISL_1263459	BTKLPP Ambon	National Institute of Health Research and Development	Arie Ardiansyah Nugraha; Hana Apsari Pawestri; Hartanti Dian Ikawati; Kartika Dewi Puspa; Krisna Pangesti; Nelly Puspandari; Subangkit; Vivi Setiawaty
EPI_ISL_2970354	Bacterial and Viral Diseases & Biotechnology and Bioinformatics, Armauer Hansen Research Institute	Bacterial and Viral Diseases & Biotechnology and Bioinformatics, Armauer Hansen Research Institute	A.A.; A.G.; Abdissa, A.; Abebe, M.; Adnew, B.; Alemayehu; Alemu, F.; Bayih; Beyene; D.A.; D.H.; G.T.; Gelanew, T.; Mihrete, A.; Mulu, A.; Negash; Seyoum, T.; Tefera
EPI_ISL_1159375	Bahman Hospital	Laboratory of Molecular Biology and Cancer Immunology,Lebanese University Public Health England	Fadi Abdel Sater; Steven Pullan
EPI_ISL_2544801	Balai Besar Penelitian dan Pengembangan dan Reservoir Penyakit, Salatiga	Genomik Solidaritas Indonesia Laboratorium	Alfin Mohammad Abdillah; Anindya Pradipta Susanto; Annisa Muthiah Sukirman; Anuraj Shankar; Ariel Pradipta; Budi Wiweko; Carissa Sintca Wijaya; Dhahlia Agustina Cahyono; Gracia Felias Enos Korompis; Joko Waluyo; Louisa Markus; Meutia Ayuputeri Kumaheri; Normastuti Adhini Tantyo; Pratiwi Sudarmono; Vania Gavrila Wikasa; Yudistira Aditya Rahmat
EPI_ISL_2343223	Banteay Meanchey Rapid Response Team	Virology Unit, Institut Pasteur du Cambodge	Cecile Troupin; Chau Darapheak; Chin Savuth; Erik A Karlsson; Jurre Y Siegers; Kraing Sidonn; Leakhena Pum; Ly Sovann; Veasna Duong; Yi Sengdoeurn
EPI_ISL_766645	Barnakuten	The Public Health Agency of Sweden	Department of Microbiology; The Public Health Agency of Sweden
EPI_ISL_833333	Batangas City Health Office	Research Institute for Tropical Medicine	Catalino Demetria; Daria Manalo; Edelwisa Mercado; Francisco Gerardo Polotan; Hannah Leah Morito; Inez Andrea Medado; John Leonard Chan; Kirstyn Brunker; Ma Angelica Tujan; Othoniel Jan Onza
EPI_ISL_2406459, EPI_ISL_2406460	Battambang Provincial Laboratory	Virology Unit, Institut Pasteur du Cambodge	Cecile Troupin; Chau Darapheak; Chin Savuth; Erik A Karlsson; Jurre Y Siegers; Kraing Sidonn; Leakhena Pum; Ly Sovann; Veasna Duong; Yi Sengdoeurn
EPI_ISL_1117377	Beijing Center for Disease Prevention and Control	Beijing Center for Disease Prevention and Control	Bing Lyu; Daitao Zhang; Fu Li; Lijuan Chen; Quanyi Wang; Shujuan Cui; Yang Pan; Zhaomin Feng; Zhichao Liang
EPI_ISL_529216	Beijing Institute of Microbiology and Epidemiology	Beijing Institute of Microbiology and Epidemiology	Cui, Y.; Fan; Guo, Y.; Hang; Hou, J.; Li, B.; Mi, Z.; Mu, J.; Qin, E.; Song; Teng; Wu, Y.; Xu, Z.; Yajun.; Yang, R.; Yong, Y.; Yue; Zhang, X.
EPI_ISL_509712	Belize Ministry of Health	Pathogen Discovery, Respiratory Viruses Branch, Division of Viral Diseases, Centers for Disease Control and Prevention	Anna Uehara; Clinton Paden; Haibin Wang; Jing Zhang; Krista Queen; Suxiang Tong; Yan Li; Ying Tao
EPI_ISL_780383, EPI_ISL_780384, EPI_ISL_780398	Bermuda Government Molecular Diagnostics Laboratory (MDL)	Respiratory Virus Unit, National Infection Service, Public Health England	Dr Ayoola Oyinloye (Bermuda); Dr Carika Weldon (Bermuda); PHE Covid Sequencing Team
EPI_ISL_2802860	BioMoLab	Microbiologia Molecular, Instituto SELADIS, Universidad Mayor de San Andrés	Aneth Vasquez Michel; Carmen Delgado Barrera; Oscar M. Rollano-Peñaloza; Sandra Miranda Sardon
EPI_ISL_2802858, EPI_ISL_2802859, EPI_ISL_2802863	BioMoLab	Molecular Genetics Laboratory, Instituto de Investigaciones Químicas, Universidad Mayor de San Andrés	Aneth Vasquez Michel; Carmen Delgado Barrera; Oscar M. Rollano-Peñaloza; Sandra Miranda Sardon
EPI_ISL_1502568, EPI_ISL_1502570, EPI_ISL_2107513, EPI_ISL_2107517	Biochemistry and Molecular Biology Department-Faculty of Medicine, Al-Quds University	Biochemistry and Molecular Biology Department-Faculty of Medicine, Al-Quds University	Al-Jawabreh, A.; Dumaidi, K.; Ereqat, S.; Naserddin, A.; Nasereddin, A.
EPI_ISL_1707927, EPI_ISL_2878346, EPI_ISL_2878383	Biogroup Bio Lam-LCD Saint-Denis	Department of Virology, Henri Mondor University Hospital, Assistance Publique Hôpitaux de Paris, Université Paris-Est Créteil, INSERM U955	Alexandre Soulier; Christophe Rodriguez; Elisabeth Trawinski; Guillaume Gricourt; Jean-Michel Pawlotsky; Melissa N'Debi; Slim Fourati; Vanessa Demontant
EPI_ISL_429995, EPI_ISL_430011, EPI_ISL_635778, EPI_ISL_635781, EPI_ISL_730270, EPI_ISL_730479, EPI_ISL_730558, EPI_ISL_730559, EPI_ISL_730562, EPI_ISL_755242, EPI_ISL_878477			
see above	Biolab Diagnostic Laboratories	Andersen lab at Scripps Research	Ahmad Tibi; Amid Abdelnour with SEARCH Alliance San Diego; Issa Abu-Dayyeh; Lama Hussein; Lina Mohammad; Zein Naber
EPI_ISL_977602, EPI_ISL_1406195, EPI_ISL_1406239, EPI_ISL_1822601, EPI_ISL_1823120, EPI_ISL_1823177, EPI_ISL_1823202, EPI_ISL_1824717, EPI_ISL_2105670, EPI_ISL_2105672, EPI_ISL_2105674, EPI_ISL_2617461, EPI_ISL_2617463, EPI_ISL_2658758, EPI_ISL_2658760, EPI_ISL_2658761, EPI_ISL_2658762, EPI_ISL_2693977, EPI_ISL_2693980, EPI_ISL_2868422			
see above	Biolab Diagnostic Laboratories	Biolab Diagnostic Laboratories	Ahmad Tibi; Amid Abdelnour; Badia Saddedin; Eiad Atwa; Issa Abu-Dayyeh; Lama Hussein; Shaima Ali; Shayma Ali
EPI_ISL_527007	Biological Prevention, Army	Biological Prevention, Army	A.E.; A.F.; A.M.; Ageez; B.E.; Elhoseiny; Gad; Harty; M.D.; M.F.; M.G.; Seadawy; Shabaan; Shamel
EPI_ISL_526975, EPI_ISL_526976, EPI_ISL_526977, EPI_ISL_526978, EPI_ISL_526979, EPI_ISL_526980, EPI_ISL_526981, EPI_ISL_526982, EPI_ISL_526983, EPI_ISL_526984, EPI_ISL_526985, EPI_ISL_526986, EPI_ISL_526987, EPI_ISL_526988, EPI_ISL_526989, EPI_ISL_526990, EPI_ISL_526991, EPI_ISL_526992, EPI_ISL_526993, EPI_ISL_526994, EPI_ISL_526995, EPI_ISL_526996, EPI_ISL_526997, EPI_ISL_526998, EPI_ISL_526999, EPI_ISL_527000, EPI_ISL_527001, EPI_ISL_527002, EPI_ISL_527003, EPI_ISL_527004, EPI_ISL_527005, EPI_ISL_527006			
see above	Biological prevention, army	Biological prevention, army	A.E.; A.F.; A.M.; Ageez; B.E.; Elhoseiny; Elhosieny; Gad; Harty; M.D.; M.F.; M.G.; Seadawy; Shabaan; Shamel
EPI_ISL_510526, EPI_ISL_510532	Biological prevention, army	Biological prevention, army	A.A.; A.F.; A.M. and Soliman; Ali; Amer; B.E.; B.S.; ELnabrawy; ElGohary; Elhoseiny; Elhoseny; Elnagdy; Elnakeeb; Gad; H.A.; Harty; Hassan; Kandeil; Karam; M.A.; M.D.; M.F.; M.G.; M.M. and Gad; Raouf; Seadawy; Shamel; T.A.; W.A.; Y.A.; k.E.
EPI_ISL_1524332, EPI_ISL_1524359, EPI_ISL_2931131, EPI_ISL_2931134, EPI_ISL_2931135, EPI_ISL_2931137, EPI_ISL_2931138			
see above	Biology Department, College of Science, Al Muthanna University and Public Health Laboratory, Al-Muthanna Health	Department of Virology, Faculty of Medicine, University of Helsinki, Helsinki, Finland	Alaa Hameed; Ali Jasim; Batool Kadham Salman; Hussein Alburkat; Hussein Riadh Kitab; Murad Munahi; Nihad Al-Rashedi; Olli Vapalahti; Tarja Sironen; Teemu Smura

	Directorate		
EPI_ISL_582030	Biology Department, College of Science, Al-Muthanna University	International Centre for Genetic Engineering and Biotechnology (ICGEB) and ARGO Open Lab Platform	Alessandro Marcello; Danilo Licastro; Nihad Al-Rashedi; Simeone Dal Monego; Sreejith Rajasekharan
EPI_ISL_1015369	Biology Lab, HIA PERCY	IRBA, 2MI	FOISSAUD V.; GORGE O.; NOLENT F.; SARILAR V.
EPI_ISL_907076, EPI_ISL_907077, EPI_ISL_907078, EPI_ISL_907079, EPI_ISL_907080, EPI_ISL_907081, EPI_ISL_907082, EPI_ISL_907083, EPI_ISL_907084, EPI_ISL_907085			
see above	Biology, MCL	Biology, MCL	A.F.; A.N.; Elhoseiny; Gad; M.D.; M.F.; M.G.; Seadawy; Shamel; Zekri
EPI_ISL_794634	Biology, MCL	Biology, MCL	A.F.; A.S.; B.S. and EL-Safty; EL-harty; EL-hosieny; Gad; M.D.; M.F.; M.G.; Seadawy; Shamel
EPI_ISL_1420697	Biomedical Research Foundation of the Academy of Athens (BRFAA)	Greek Genome Center, Biomedical Research Foundation of the Academy of Athens (BRFAA)	Dimitrios Thanos; Emmanouil Athanasiadis; Ioannis Vatsellas; Katerina Zoi; Theodoros Loupis
EPI_ISL_2401515	BioneXt Lab	Laboratoire national de sante, Microbiology, Microbial Genomics Platform	Anke Wienecke-Baldacchino; Catherine Ragimbeau; Fatu Djabi; Jessica Tapp; Lise Pignon; Raoul Salmon; Tamir Abdelrahman; Thibault Ferrandon
EPI_ISL_2878374, EPI_ISL_2984054	Biopole Antilles	Department of Virology, Henri Mondor University Hospital, Assistance Publique Hôpitaux de Paris, Université Paris-Est Créteil, INSERM U955	Alexandre Soulier; Christophe Rodriguez; Elisabeth Trawinski; Guillaume Gricourt; Jean-Michel Pawlotsky; Melissa N'Debi; Slim Fourati; Vanessa Demontant
EPI_ISL_1140703, EPI_ISL_1211107, EPI_ISL_1211289, EPI_ISL_1211353, EPI_ISL_1212796, EPI_ISL_1216676, EPI_ISL_1282001, EPI_ISL_1282133, EPI_ISL_1282295, EPI_ISL_1284692, EPI_ISL_1353663, EPI_ISL_1439255, EPI_ISL_1721811, EPI_ISL_2633951, EPI_ISL_2634081, EPI_ISL_2637135			
see above	Bioscientia Labor Wermsdorf	Robert Koch Institute	
EPI_ISL_1284823, EPI_ISL_1432947, EPI_ISL_1721133, EPI_ISL_1848523, EPI_ISL_1848543, EPI_ISL_2109502, EPI_ISL_2121932, EPI_ISL_2122022, EPI_ISL_2125493, EPI_ISL_2125560, EPI_ISL_2126008, EPI_ISL_2260272, EPI_ISL_2265701, EPI_ISL_2265849, EPI_ISL_2265853, EPI_ISL_2313390, EPI_ISL_2313636, EPI_ISL_2632395, EPI_ISL_2632447, EPI_ISL_2632460, EPI_ISL_2632554			
see above	Bioscientia MVZ Labor Karlsruhe GmbH	Robert Koch Institute	
EPI_ISL_1970307	Biotechnology Division, NCDC Delhi	NCDC Delhi, Biotechnology Division	Hema Gogia; Hemlata Lall; Kalaiarasan Ponnusamy; Mahesh S Dhar; Manoj K Singh; Meena Datta; Partha Rakshit; Preeti Madan; Priyanka Singh; Radhakrishnan V. S; Robin Marwal; Sandhya Kabra; Sujeet K Singh; Uma Sharma
EPI_ISL_745169	Bishop Lavis CDC wc BLP	National Health Laboratory Service (NHLS), Tygerberg	Bronwyn Kleinhans; Eduan Wilkindon; Gert van Zyl; Houriiyah Tegally; Kayla Delaney; Susan Engelbrecht; Tulio de Oliveira; Wolfgang Preiser
EPI_ISL_2380020, EPI_ISL_2608398	Borneo Medical Centre	Institute of Health and Community Medicine	Chan Chia Jui; Chua Hock Hin; David Perera; Ooi Mong How; Tonnii Sia Loong Loong; Wong Jyn Shan; Wong Kiing Aik
EPI_ISL_871872, EPI_ISL_968214, EPI_ISL_1516831, EPI_ISL_1516846, EPI_ISL_1516847, EPI_ISL_2233458, EPI_ISL_2233463, EPI_ISL_2372269, EPI_ISL_2372270, EPI_ISL_2372271, EPI_ISL_2372273, EPI_ISL_2372275, EPI_ISL_2372276, EPI_ISL_2372277, EPI_ISL_2372280, EPI_ISL_2372283, EPI_ISL_2372286, EPI_ISL_2372287, EPI_ISL_2372294, EPI_ISL_2372297, EPI_ISL_2372299, EPI_ISL_2372305, EPI_ISL_2386153, EPI_ISL_2386154, EPI_ISL_2504067, EPI_ISL_2504068, EPI_ISL_2504069, EPI_ISL_2504072, EPI_ISL_2504074, EPI_ISL_2504075, EPI_ISL_2504077, EPI_ISL_2504080, EPI_ISL_2504082, EPI_ISL_2504084, EPI_ISL_2504087, EPI_ISL_2566206, EPI_ISL_2566207, EPI_ISL_2566208, EPI_ISL_2566209, EPI_ISL_2648202, EPI_ISL_2820337, EPI_ISL_2820338, EPI_ISL_2820339, EPI_ISL_2820340, EPI_ISL_2820341, EPI_ISL_2820342, EPI_ISL_2820343, EPI_ISL_2820345, EPI_ISL_2820347, EPI_ISL_2820348, EPI_ISL_2820349, EPI_ISL_2820350, EPI_ISL_2820352, EPI_ISL_2820353, EPI_ISL_2820354, EPI_ISL_2820355, EPI_ISL_2820357, EPI_ISL_2820358, EPI_ISL_2820359, EPI_ISL_2820360, EPI_ISL_2820361, EPI_ISL_2820362, EPI_ISL_2820364, EPI_ISL_2820365, EPI_ISL_2820366, EPI_ISL_2820367, EPI_ISL_2820368, EPI_ISL_2820370, EPI_ISL_2820371, EPI_ISL_2820373, EPI_ISL_2820374, EPI_ISL_2820418, EPI_ISL_2820419, EPI_ISL_2820427, EPI_ISL_2820429, EPI_ISL_2820435, EPI_ISL_2820436, EPI_ISL_2820438, EPI_ISL_2820439, EPI_ISL_2820440, EPI_ISL_2820442, EPI_ISL_2820444, EPI_ISL_2820445, EPI_ISL_2820453, EPI_ISL_2820455, EPI_ISL_2820460, EPI_ISL_2820461, EPI_ISL_2868330, EPI_ISL_2868335, EPI_ISL_2868338, EPI_ISL_2868339, EPI_ISL_2868348, EPI_ISL_2868351, EPI_ISL_2868359, EPI_ISL_2868361, EPI_ISL_2868362, EPI_ISL_2868363, EPI_ISL_2868373, EPI_ISL_2868378, EPI_ISL_2868387, EPI_ISL_2868388, EPI_ISL_2868397, EPI_ISL_2868398, EPI_ISL_2868402, EPI_ISL_2868406, EPI_ISL_2931798, EPI_ISL_2931799, EPI_ISL_2931800, EPI_ISL_2931801, EPI_ISL_2931803, EPI_ISL_2931805, EPI_ISL_2931807, EPI_ISL_2931808, EPI_ISL_2931809, EPI_ISL_2931810, EPI_ISL_2931811, EPI_ISL_2931812, EPI_ISL_2931813, EPI_ISL_2931814, EPI_ISL_2931815, EPI_ISL_2931816, EPI_ISL_2931817, EPI_ISL_2931819, EPI_ISL_2931820, EPI_ISL_2931822, EPI_ISL_2931823, EPI_ISL_2931824, EPI_ISL_2931825, EPI_ISL_2931826, EPI_ISL_2931830, EPI_ISL_2931834, EPI_ISL_2931835, EPI_ISL_2931836			
see above	Botswana Harvard HIV Reference Laboratory	Botswana Harvard HIV Reference Laboratory	Boitumelo Zuze; Botshelo Radibe; David Lawrence; Dorcas Maruapula; Godfrey Simoonga; Irvin Kgetse; Joseph; Joseph Makhema; Kelebeletse K. Mokobela; Keoratile Ntshambiwa; Kereng Mphoyakgosi; Kgomotso Moruisi; Kwana Lechiile; Legodile Kooepile; Lindani Bochena; Madisa Mine; Makhema; Modisa Motswaledi; Mosepele Mosepele; Motlalepule L. Pone; Ontlametse T. Bareng; Pamela Smith-Lawrence; Roger Shapiro; Shahin Lockman; Sikhulile Moyo; Sikhulile Wonderful T. Choga; Simani Gaseitsiwe; Thongbotho Mphoyakgosi; Wonderful Choga; Wonderful T. Choga
EPI_ISL_1001001, EPI_ISL_1034758, EPI_ISL_1034760	Bundeswehr Institute of Microbiology	Bundeswehr Institute of Microbiology	Alexandra Rehn; Enrico Georgi; Malena Bestehorn-Willmann; Markus Antwerpen; Mathias Walter; Mike Pillukat; Roman Wölfel; Sabine Zange
EPI_ISL_1834008	Bundeswehrkrankenhaus Berlin	Bundeswehr Institute of Microbiology	Alexandra Rehn; Enrico Georgi; Malena Bestehorn-Willmann; Markus Antwerpen; Mathias Walter; Mike Pillukat; Roman Wölfel; Sabine Zange
EPI_ISL_2779327	Bungoma County Referral Hospital	USAMRD-A, Basic Science Laboratory	Alan Lemtudo; Beth Mutai; Brian Andika; Carol Kifude; Clement Masakwe; Eric Muthanje; Esther Omuseni; Faith Sigei; Gathii Kimita; George Awinda; John Waitumbi; Josphat Nyataya; Rachel Githii; Rehema Liyai; Stephen Ochola
EPI_ISL_2779387, EPI_ISL_3031380	Busia County Referral Hospital	USAMRD-A, Basic Science Laboratory	Alan Lemtudo; Beth Mutai; Brian Andika; Carol Kifude; Clement Masakwe; Eric Muthanje; Esther Omuseni; Faith Sigei; Gathii Kimita; George Awinda; John Waitumbi; Josphat Nyataya; Rachel Githii; Rehema Liyai; Stephen Ochola
EPI_ISL_2779289	Busia border point	USAMRD-A, Basic Science Laboratory	Alan Lemtudo; Beth Mutai; Brian Andika; Carol Kifude; Clement Masakwe; Eric Muthanje; Esther Omuseni; Faith Sigei; Gathii Kimita; George Awinda; John Waitumbi; Josphat Nyataya; Rachel Githii; Rehema Liyai; Stephen Ochola
EPI_ISL_1371903, EPI_ISL_2289766	C H DE LA POLYNESIE FRANCAISE	CNR Virus des Infections Respiratoires - France SUD	Antonin Bal; Bruno Lina; Bruno Simon; Gregory Destras; Gwendolyne Burfin; Hadrien Regue; Laurence Josset; Martine Valette; Quentin Semanas
EPI_ISL_2674322	CARVAJAL LABORATORIOS	Instituto Nacional de Salud- Dirección de Investigación en Salud Pública	Carlos Franco-Muñoz; Carmen Osorio; Diana Malo; Diego A. Álvarez-Díaz; Diego Andrés Prada; Gerardo Santamaría; Hector Alejandro Ruiz-Moreno; Jhonnatan Reales-González; Jorge Rivera; Juan Camilo Martinez; Julian Naizaque; Katherine Laiton-Donato; Lisseth Pardo; Magdalena Wiesner; Marcela Mercado-Reyes; Maria T. Herrera-Sepúlveda; Marta Lopez Blanco; Martha Lucia Ospina Martinez; Paola Rojas; Sergio Gomez; Sheryll Corchuelo; Ángela Alarcon Cruz
EPI_ISL_2657865	CARVAJALA LABORATORIO IPS	Instituto Nacional de Salud- Dirección de Investigación en Salud Pública	Carlos Franco-Muñoz; Carmen Osorio; Diana Malo; Diego A. Álvarez-Díaz; Diego Andrés Prada; Gerardo Santamaría; Hector Alejandro Ruiz-Moreno; Jhonnatan Reales-González; Jorge Rivera; Juan Camilo Martinez; Julian Naizaque; Katherine Laiton-Donato; Lisseth Pardo; Magdalena Wiesner; Marcela Mercado-Reyes; Maria T. Herrera-Sepúlveda; Marta Lopez Blanco; Martha Lucia Ospina Martinez; Paola Rojas; Sergio Gomez; Sheryll Corchuelo; Ángela Alarcon Cruz
EPI_ISL_2825738	CDPH VBL	California Department of Public Health	CDPH-COVIDNet; UCLA Technology Center for Genomics & Bioinformatics
EPI_ISL_605780, EPI_ISL_605781, EPI_ISL_605782	CEIRS Data Processing and Coordinating Center, St. Jude Center of Excellence for Influenza Research and Surveillance (CEIRS)	CEIRS Data Processing and Coordinating Center, St. Jude Center of Excellence for Influenza Research and Surveillance (CEIRS)	A.E.; Ali; El-Guindy; El-Sayes, M.; El-Shesheny, R.; El-Taweel, A.; Gomaa, M.; Kamel; Kandeil, A.; Kayali, G.; Kayed; Kutkat, O.; M.A.; M.N.; Mahmoud; Mahrous, N.; Moatasim, Y.; Mostafa, A.; N.M.; Naguib, A.; Roshdy; S.H.; Shehata, M.; Showky, S.; W.H.; Webby, R.
EPI_ISL_2471228, EPI_ISL_3043914	CENTOGENE Frankfurt Laboratory: Niederlassung Industriepark Höchst	Robert Koch Institute	
EPI_ISL_1695254	CENTRE HOSPITALIER DU HAUT BUGEY	CNR Virus des Infections Respiratoires - France SUD	Antonin Bal; Bruno Lina; Bruno Simon; Gregory Destras; Gwendolyne Burfin; Hadrien Regue; Laurence Josset; Martine Valette; Quentin Semanas
EPI_ISL_2373694	CENTRE HOSPITALIER ST JOSEPH ST LUC	CNR Virus des Infections Respiratoires - France SUD	Antonin Bal; Bruno Lina; Bruno Simon; Gregory Destras; Gwendolyne Burfin; Hadrien Regue; Laurence Josset; Martine Valette; Quentin Semanas
EPI_ISL_644252	CEPHR / Mater Hospital	Irish Coronavirus Sequencing Consortium - National Virus Reference Laboratory	Alejandro Abner Garcia Leon; Gabriel Gonzalez; Michael Carr; Patrick Mallon
EPI_ISL_2562031, EPI_ISL_2562045	CH Princesse Grace	Cerba lab	Aude Lessenne; Bénédicte Roquebert; Emmanuel Lecorche; Kader Merah; Laura Verdurme; Patrice Herisson; Sabine Trombert-Paolantoni; Stéphanie Haïm-Boukobza; Thierry Collin
EPI_ISL_2933198	CHR LA REUNION FELIX GUYON	CNR Virus des Infections Respiratoires - France SUD	Antonin Bal; Bruno Lina; Gregory Destras; Gwendolyne Burfin; Hadrien Regue; Laurence Josset; Martine Valette; Quentin Semanas
EPI_ISL_2694661, EPI_ISL_2694662, EPI_ISL_2709915, EPI_ISL_2709935	CHRIS HANI BARAGWANATH	National Institute for Communicable Diseases of the National Health Laboratory Service	Amoako DG; Bhiman JN; Everatt J; Ismail A; Mahlangu B; Mnguni A; Mohale T; Ntuli N; Scheepers C
EPI_ISL_2273025, EPI_ISL_2273036, EPI_ISL_2273053, EPI_ISL_2273054, EPI_ISL_2382241, EPI_ISL_2382243, EPI_ISL_2382682, EPI_ISL_2454635, EPI_ISL_2454649, EPI_ISL_2662632, EPI_ISL_2662644, EPI_ISL_2662645, EPI_ISL_2662650			
see above	CHRIS HANI BARAGWANATH LABORATORY	National Institute for Communicable Diseases of the National Health Laboratory Service	Amoako DG; Bhiman JN; Everatt J; Ismail A; Mahlangu B; Mnguni A; Mohale T; Ntuli N; Scheepers C
EPI_ISL_660356	CHU Clermont-Ferrand	CNR Virus des Infections Respiratoires - France SUD	Amélie Brebion; Antonin Bal; Audrey Mirand; Bruno Lina; Christel Regagnon; Christine Archimbaud; Cécile Henquell; Gregory Destras; Gwendolyne Burfin; Hadrien Règue; Hélène Chabrolles; Laurence Josset; Martine Chambon; Martine Valette; Maxime Bisseux; Patricia Combes; Quentin Semanas
EPI_ISL_2403233, EPI_ISL_2403237, EPI_ISL_2403240, EPI_ISL_2403242, EPI_ISL_2709247, EPI_ISL_2709252, EPI_ISL_3039524, EPI_ISL_3039535			
see above	CHU Pontchaillou	CHU Pontchaillou	DE TAYRAC Marie; DENOUAL Florent; ETCHEVERRY Amandine; FEBREAU Christine; GALIBERT Marie Dominique; GROLHIER Claire; JAGLINE Steven; PRONIER Charlotte; SASSI Mohamed; THIBAULT Vincent
EPI_ISL_593859, EPI_ISL_2351673	CHU Purpan - Laboratoire de Virologie - Institut Fédératif de Biologie	CHU Purpan - Laboratoire de Virologie - Institut Fédératif de Biologie	Boyer P.; Bulach T.; Carcenac R.; Donnadieu C.; Dubois M.; Harter A.; Izopet J.; Latour J.; Milhes M.; Nicot F.; Ranger N.; Salin G.; Tremeaux P.
EPI_ISL_2509992	CHUGA-IBP-laboratoire de Virologie	IBP-laboratoire de virologie	Alban Caporossi; Anne Signori -Schmuck; Anne-Karen Faure; Aurélie Truffot; Benjamin Nemoz; Hugo Jardin; Julien Andréani; Julien Lupo; Léa Ponderand; Pascal Poignard; Patrice Morand; Raphaële Germi; Sylvie Larrat
EPI_ISL_2249173	CHULN - H Santa Maria	Instituto Nacional de Saude (INSA) and Centro de Investigaçao em Biodiversidade e Recursos Geneticos (CIBIO), Universidade do Porto	Borges et al
EPI_ISL_1545239, EPI_ISL_2367418	CHUV	Laboratory of genomics and metagenomics	Claire Bertelli; Damien Jacot; Gilbert Greub; Sébastien Aeby; Trestan Pillonel
EPI_ISL_2683495, EPI_ISL_2683873	CICM, Bamako	Malaria Research and Training Center-Bamako	Abdoul Karim Sangare; Abdoulaye Djimde; Amadou Daou; Antoine Dara; Bourema Kouriba
EPI_ISL_2672214	CICM, Bamako	Malaria Research and Training Center-Parasito	Abdoul Karim Sangare; Abdoulaye Djimde; Amadou Daou; Antoine Dara; Bourema Kouriba
EPI_ISL_487463, EPI_ISL_487464	CICM-Mali	Bundeswehr Institut of Microbiology	Antwerpen; Bestehorn-Willmann; Dürr; Heitzer; Kouriba; Maiga; Quedraogo; Rehn; Sangaré; Sogodogo; Traoré; Walter; Wölfel; Zimmermann
EPI_ISL_2502731	CLINICA BIBLICA	Inciensa, Instituto Costarricense de InvestigaciOn y Enseñanza en NutriciOn y Salud	Adriana Godínez; Claudio Soto-Garita; Estela Cordero; Francisco Duarte; Hebleen Porras; Jose Luis Vargas; Joselyn Prado & Karla Gutierrez-González; Mariela Gutierrez; Melany CalderOn
EPI_ISL_1299497, EPI_ISL_1591100	CMA Dano	Centre Muraz	Abdoul-Salam Ouedraogo; Ange Badjo; Armel Poda; Arsène Somé; Arsène Zongo; Essia Belarbi; Fabian Leendertz; Firmin Kaboré; Grit Schubert; Jasmin Schlotterbeck; Soumeya Ouangraoua; Thérèse Kagone; Yacouba Sawadogo
EPI_ISL_2438608	COCR NEW TENGIZ Clinic	Reference laboratory for the control of viral infections	Aidar Usserbayev; Aknur Mutaliyeva; Andrey Komissarov; Artem Fadeev; Azamat Kenessov; Bekzhan Maikotov; Gaukhar Nussupbayeva; Madina Tleubergenova; Maria Pisareva; Nazym Tleumbetova

EPI_ISL_2484798	COMMUNITY TESTING CENTRE	Hong Kong Department of Health	Alan K.L. Tsang; Dominic N.C. Tsang; Edman T.K. Lam; Gannon C.K. Mak; Ken H.L. Ng; Peter C.W. Yip; Peter K.C. Cheng; Rickjason C.W. Chan
EPI_ISL_2227272, EPI_ISL_2227273	COVID-19 National Reference Laboratory, Pasteur Institute of Iran	Genetics Research Center, University of Social Welfare and Rehabilitation Sciences	Hossein Najmabadi; Hossein Najmabadi.; Kimia Kahrizi; Mahsa Tavakoli; Marzieh Mohseni; Mohammad Hassan Pouriayevali; Mostafa Salehi-Vaziri; Tahmineh Jalali; Zohreh Fattahi
EPI_ISL_1190767	CREMER(Centre de Recherches sur les Maladies Emergentes et Ré-émergentes)	TransVIHMI(Recherches Translationnelles sur le VIH et les Maladies Infectieuses)	Ahidjo Ayouba; Celestin Godwe; Christelle Butel; Dowbiss Meta Djomsi; Eitel Mpoudi Ngole; Eric Delaporte; Esemu Livo; Laetitia Serrano; Marcel Tongo; Marie Amougou; Martin Maidadi Foudi; Martine Peeters; Nicole Vidal; Rodrigue Kamga
EPI_ISL_636980	CS Xai Xai	KRISP, KZN Research Innovation and Sequencing Platform	Giandhari J; Ismael N; Nadia Sitoe; Nedio Mabunda; Paulo Arnaldo; Pillay S; Tegally H; Wilkinson E; de Oliveira T
EPI_ISL_910079	CSIR-Centre for Cellular and Molecular Biology	CSIR-Centre for Cellular and Molecular Biology	Archana Bharadwaj Siva; B Himasri; Blessy B John; Divya Tej Sowpati; Karthik Bharadwaj Tallapaka; Lamuk Zaveri; Namami Gaur; Payel Mukherjee; Pratheusa Maccha; Priya Singh; Purushotham Vodnala; Rakesh K Mishra; Sofia Banu; Tulasi Nagabandi; Viswagithe S L
EPI_ISL_1380396, EPI_ISL_1838393, EPI_ISL_1838400, EPI_ISL_1838452, EPI_ISL_1838522, EPI_ISL_2775002	CSIR-Centre for Cellular and Molecular Biology	CSIR-Centre for Cellular and Molecular Biology-INSACOG	Amareshwar Vodapalli; Ara Sreenivas; Archana Bharadwaj Siva; B Himasri; Blessy B John; Divya Tej Sowpati; Jandhyala Sai Krishna; Karthik Bharadwaj Tallapaka; Lamuk Zaveri; Namami Gaur; Onkar Kulkarni; Payel Mukherjee; Pratheusa Maccha; Priya Nurkurthy; Priya Singh; Purushotham Vodnala; Rakesh K Mishra; Sharath Chandra Thota; Shreekant Verma; Sofia Banu; Sumedha Avadhanula; Tulasi Nagabandi; Valli Nagalakshmi Undamatla; Vidhyadhari Methuku; Viswagithe S L
EPI_ISL_2754370	CURE	Institut Pasteur de Montevideo	Andres Lizasoain; Belén González; Cecilia Alonso; Daiana Mir; Emiliano Pereira; Gonzalo Bello; Ighor Arantes; Juan Zanetti; Lucia Bilbao; Luciana Griffero; Lucía Spangenberg; Mailen Arleo; Mariana Brandes; María José Benítez-Galeano; Matías Castells; Matías Salvo; Matías Victoria; Mauricio Méndez; Melissa Duquía; Natalia Rego; Natalia Reyes; Odhille Chappos; Pablo Smircich; Pía Techera; Rodney Colina; Tamara Fernández-Calero; Tania Possi; Verónica Noya
EPI_ISL_935791	Cadham Provincial laboratory	National Microbiology Laboratory (NML)	Anna Majer; Anneliese Landgraff; CanCOGeN's metadata curation team; Darian Hole; David Alexander; Elsie Grudeski; Gary Van Domselaar; Grace Seo; Jared Bullard; Jennifer Tanner; Kerry Dust; Kirsten Biggar; Madison Chapel; Morag Graham; Natalie Knox; Nathalie Bastien; Paul Van Caeseele; Philip Mabon; Public Health Agency of Canada CanCOGeN team; Rhiannon Huzarewich; Russell Mandes; Shari Tyson; Timothy Booth; Yan Li
EPI_ISL_413557	California Department of Public Health	Chiu Laboratory, University of California, San Francisco	Chao-Yang Pan; Debra A. Wadford; Hugo Guevara; Scot Federman; Wei Gu; Xianding Deng; and Charles Y. Chiu
EPI_ISL_1817730	Calvinia RSC Clinic	NHLS/UCT	Arash Iranzadeh; Bruna Galvao; Carolyn Williamson; Deelan Doolabh; Diana Hardie; Innocent Mudau; Kruger Marais; Lynn Tyers; Marvin Hsiao; Stephen Korsman
EPI_ISL_2547359, EPI_ISL_2547361	Calvinia RSC Clinic	National Health Laboratory Service/University of Cape Town (NHLS/UCT)	Arash Iranzadeh; Bruna Galvao; Carolyn Williamson; Deelan Doolabh; Diana Hardie; Gert Marais; Innocent Mudau; Lynn Tyers; Marvin Hsiao; Stephen Korsman
EPI_ISL_1096139, EPI_ISL_1096140	Cambodian National Public Health Laboratory, National Institute of Public Health	Virology Unit, Institut Pasteur du Cambodge	Chau Darapheak; Chin Savuth; Erik A Karlsson; Kraing Sidonn; Ly Sovann; Sokhoun Yann; Veasna Duong; Yi Sengdoeurn
EPI_ISL_576371, EPI_ISL_576372, EPI_ISL_576373, EPI_ISL_857315, EPI_ISL_857316, EPI_ISL_857317, EPI_ISL_857318, EPI_ISL_857319, EPI_ISL_857320, EPI_ISL_857321, EPI_ISL_857322, EPI_ISL_857323, EPI_ISL_857324, EPI_ISL_857325, EPI_ISL_857326, EPI_ISL_857327, EPI_ISL_857328, EPI_ISL_857329, EPI_ISL_857330, EPI_ISL_857331, EPI_ISL_857332, EPI_ISL_857333, EPI_ISL_857334, EPI_ISL_857335, EPI_ISL_857336, EPI_ISL_857337, EPI_ISL_857338, EPI_ISL_857339, EPI_ISL_857340, EPI_ISL_857341, EPI_ISL_857342, EPI_ISL_857343, EPI_ISL_857344, EPI_ISL_857345, EPI_ISL_857346, EPI_ISL_857347, EPI_ISL_862783, EPI_ISL_862784, EPI_ISL_862785, EPI_ISL_862786, EPI_ISL_862787, EPI_ISL_862788, EPI_ISL_862789, EPI_ISL_862790, EPI_ISL_862791, EPI_ISL_862792, EPI_ISL_862793, EPI_ISL_862794, EPI_ISL_862795, EPI_ISL_862796, EPI_ISL_862797, EPI_ISL_862798, EPI_ISL_862799, EPI_ISL_862800, EPI_ISL_862801, EPI_ISL_862802, EPI_ISL_862803, EPI_ISL_862804, EPI_ISL_862805, EPI_ISL_862806, EPI_ISL_862807, EPI_ISL_862808, EPI_ISL_862809, EPI_ISL_862810, EPI_ISL_862811, EPI_ISL_862812, EPI_ISL_862813, EPI_ISL_890195, EPI_ISL_890196, EPI_ISL_890197, EPI_ISL_890198, EPI_ISL_890199, EPI_ISL_890200, EPI_ISL_890201, EPI_ISL_890202, EPI_ISL_890203, EPI_ISL_890204, EPI_ISL_890205, EPI_ISL_890206, EPI_ISL_890207, EPI_ISL_890208, EPI_ISL_890209, EPI_ISL_890210, EPI_ISL_890211, EPI_ISL_890212, EPI_ISL_890213, EPI_ISL_890214, EPI_ISL_890215, EPI_ISL_890216, EPI_ISL_890217, EPI_ISL_890218, EPI_ISL_890219, EPI_ISL_890220, EPI_ISL_890221, EPI_ISL_890222, EPI_ISL_890223, EPI_ISL_890224, EPI_ISL_890225, EPI_ISL_890226, EPI_ISL_890227, EPI_ISL_890228, EPI_ISL_890229, EPI_ISL_890230, EPI_ISL_890231, EPI_ISL_907086, EPI_ISL_907087, EPI_ISL_907088, EPI_ISL_907089, EPI_ISL_907090, EPI_ISL_907091, EPI_ISL_907092, EPI_ISL_907093, EPI_ISL_907094, EPI_ISL_907095, EPI_ISL_907096, EPI_ISL_907097, EPI_ISL_907098, EPI_ISL_907099, EPI_ISL_907100, EPI_ISL_907101, EPI_ISL_907102, EPI_ISL_907103, EPI_ISL_907104, EPI_ISL_907105, EPI_ISL_907106, EPI_ISL_907107, EPI_ISL_907108, EPI_ISL_907109, EPI_ISL_907110, EPI_ISL_907111, EPI_ISL_907112, EPI_ISL_907113, EPI_ISL_907114, EPI_ISL_907115, EPI_ISL_907116, EPI_ISL_907117, EPI_ISL_907118			
see above	Cancer Biology Department, National Cancer Institute	Cancer Biology Department, National Cancer Institute	A.A.; A.E.; A.N.; Abouelhoda, M.; Ahmed; Bahnassy; Elhosieny; F.W.; Gad; H.K.; Hafez; Hamdy; M.G.; M.M.; M.S.; O.S.; Sedawy; Soliman; Soliman, L.; Zekri
EPI_ISL_1040916	Cancer Biology Department, National Cancer Institute	Cancer Biology Department, National Cancer Institute	A.A.; A.E.; A.N.; Ahmed; Bahnassy; Elhosieny; F.W.; Gad; H.K.; Hafez; Hamdy; L. and Abouelhoda, M.; M.G.; M.M.; M.S.; O.S.; Sedawy; Soliman; Zekri
EPI_ISL_3021345, EPI_ISL_3021415	Cantacuzino National Military-Medical Institute, Viral Respiratory Infections Laboratory	Cantacuzino Institute Virology	Carmen Cherciu; Luiza Ustea; Mihaela Lazar; Mihaela Oprea; Nicoleta Paraschiv; Sorin Dinu
EPI_ISL_548138, EPI_ISL_548140, EPI_ISL_579063, EPI_ISL_579066, EPI_ISL_579070, EPI_ISL_579086, EPI_ISL_579090, EPI_ISL_579426, EPI_ISL_579463, EPI_ISL_579496, EPI_ISL_622788, EPI_ISL_622789, EPI_ISL_622795, EPI_ISL_622799, EPI_ISL_622800, EPI_ISL_622810, EPI_ISL_622827, EPI_ISL_661260, EPI_ISL_682296, EPI_ISL_755638, EPI_ISL_843198, EPI_ISL_1016881, EPI_ISL_1082255, EPI_ISL_1904855, EPI_ISL_1967890, EPI_ISL_2406488, EPI_ISL_2811947, EPI_ISL_2811950, EPI_ISL_2811951, EPI_ISL_2811952, EPI_ISL_2811953, EPI_ISL_2811958			
see above	Canterbury Health Laboratories	Institute of Environmental Science and Research (ESR)	Anja Werno; Antje van der Linden; Arlo Upton; Chris Mansell; David Hammer; Dragana Drinkovic; Erasmus Smit; Gary McAuliffe; Hana Sofia Andersson; Hermes Perez; James Ussher; Jill Sherwood; Jing Wang; Joep de Ligt; Josh Freeman; Julia Howard; Juliet Elvy; Lauren Jelly; Mary DeAlmeida; Matt Blakiston; Matt Storey; Matthew Rogers; Max Bloomfield; Michael Addidle; Michelle Balm; Muhammad Faisal; Nikki Freed; Olin Silander; Olivia Stroeven; Paula scholes; Rachel Boyle; Sally Roberts; SallyAnn Harbison; Sarah Jefferies; Sharmini Muttaiyah; Susan Lin; Susan Morpeth; Susan Taylor; Timothy Blackmore; Vani Sathyendran; Veronica Playle; Virginia Hope; Xiaoyun Ren
EPI_ISL_977538, EPI_ISL_977583	Caribbean Public Health Agency	Carrington Lab, Department of PreClinical Sciences	Adesh Ramsubhag; Arianne Brown-Jordan; Avery Hinds; Chinna Chinnadurai; Christine V. F. Carrington; Christopher Oura; Dr. Sharon Belmar-George; Dr. Sharon Belmar-George Avery Hinds; Gabriel Escobar; Jaya Jayaraman; Jerome Foster; Karla Georges; Marsha Ivey; Naresh Nandram; Nikita S. D. Sahadeo; Nuno Faria; Oliver Pybus; Rahul Naidu; Rajini Haraksingh; Risha Singh; Sarah Hill; Stanley Giddings; SueMin Nathaniel; Vernie Ramkissoon
EPI_ISL_977659	Caribbean Public Health Agency	Carrington Lab, Department of PreClinical Sciences, Building 36, First Floor Biochemistry Unit, Faculty of Medical Sciences, The University of the West Indies	Adesh Ramsubhag; Arianne Brown-Jordan; Avery Hinds; Chinna Chinnadurai; Christine V. F. Carrington; Christopher Oura; Gabriel Escobar; Jaya Jayaraman; Jerome Foster; Karla Georges; Kenneth George; Marsha Ivey; Naresh Nandram; Nikita S. D. Sahadeo; Nuno Faria; Oliver Pybus; Rahul Naidu; Rajini Haraksingh; Risha Singh; Sarah Hill; Stanley Giddings; SueMin Nathaniel; Vernie Ramkissoon
EPI_ISL_872194, EPI_ISL_1591250, EPI_ISL_1591351, EPI_ISL_1591518, EPI_ISL_1591718	Caribbean Public Health Agency	Carrington Lab, Department of PreClinical Sciences, Faculty of Medical Sciences, The University of the West Indies	Adesh Ramsubhag; Arianne Brown-Jordan; Avery Hinds; Chinna Chinnadurai; Christine V. F. Carrington; Christopher Oura; Gabriel Escobar; Jaya Jayaraman; Jerome Foster; Karla Georges; Marsha Ivey; Naresh Nandram; Nikita S. D. Sahadeo; Nuno Faria; Oliver Pybus; Rahul Naidu; Rajini Haraksingh; Risha Singh; Roshan Parasram; Sarah Hill; Stanley Giddings; SueMin Nathaniel; Vernie Ramkissoon
EPI_ISL_872193	Caribbean Public Health Agency	Carrington Lab, Department ofBuilding 36, First Floor Biochemistry Unit, Faculty of Medical Sciences, The University of the West Indies	Adesh Ramsubhag; Arianne Brown-Jordan; Avery Hinds; Chinna Chinnadurai; Christine V. F. Carrington; Christopher Oura; Gabriel Escobar; Jaya Jayaraman; Jerome Foster; Karla Georges; Marsha Ivey; Naresh Nandram; Nikita S. D. Sahadeo; Nuno Faria; Oliver Pybus; Rahul Naidu; Rajini Haraksingh; Risha Singh; Sarah Hill; Stanley Giddings; SueMin Nathaniel; Vernie Ramkissoon
EPI_ISL_459858	Center for Genome Regulation (CRG)	Center for Mathematical Modeling and Center for Genome Regulation. Santiago, Chile	Allende ML; Gaete A; González M.; Maass A; Palma R; Travisany D; Urra C; Varas M
EPI_ISL_1594295, EPI_ISL_1594306	Center for Laboratory Medicine St. Gallen	Center for Laboratory Medicine St. Gallen	Yannick Gerth
EPI_ISL_2835623, EPI_ISL_2835624, EPI_ISL_2835634, EPI_ISL_2835635, EPI_ISL_2835636, EPI_ISL_2835637, EPI_ISL_2835640			
see above	Center for Medical and Sanitary Research (CERMES)	CDC Atlanta	Adamou Lagare; Dhwani Batra; Justin Lee
EPI_ISL_853839	Center for Virology, Medical University of Vienna	Bergthaler laboratory, CeMM Research Center for Molecular Medicine of the Austrian Academy of Sciences	Alexander Lercher; Alexandra Popa; Andreas Bergthaler; Anna Schedl; Benedikt Agerer; Christoph Bock; Jakob-Wendelin Genger; Jan Laine; Lukas Endler; Martin Senekowitsch; Michael Schuster; Thomas Penz
EPI_ISL_2385405	Center of Hygiene and Epidemiology in Belgorod Region	WHO National Influenza Centre Russian Federation	Alexey Masharsky; Andrey Komissarov; Artem Fadeev; Daria Danilenko; Dmitry Lioznov; Elena Nabieva; Georgii Bazykin; Kirill Varchenko; Ksenia Safina; Kseniya Komissarova; Maria Baturova; Maria Pisareva; Maria Timofeeva; Mikhail Bakaev; Nikita Yolshin; Oula Masour; Tamila Musaeva; Veronika Eder
EPI_ISL_1346779	Center of Medical Microbiology, Virology, and Hospital Hygiene, University of Duesseldorf	Center of Medical Microbiology, Virology, and Hospital Hygiene, University of Duesseldorf	Alexander Dilthey; Andreas Walker; Daniel Strelow; Jessica Nicolai; Jörg Timm; Klaus Pfeffer; Lisanna Hülse; Malte Kohns Vasconcelos; Maximilian Damagnez; Nadine Lübke; Tobias Wienemann; Torsten Houwaart
EPI_ISL_2227328, EPI_ISL_2227329, EPI_ISL_2227330, EPI_ISL_2227331, EPI_ISL_2227332, EPI_ISL_2227333, EPI_ISL_2227334, EPI_ISL_2227335, EPI_ISL_2227336, EPI_ISL_2227337, EPI_ISL_2227338, EPI_ISL_2227339, EPI_ISL_2227340, EPI_ISL_2227341, EPI_ISL_2227342, EPI_ISL_2227343, EPI_ISL_2227344, EPI_ISL_2227345, EPI_ISL_2227346, EPI_ISL_2227347, EPI_ISL_2227348, EPI_ISL_2227349, EPI_ISL_2227350, EPI_ISL_2232252, EPI_ISL_2232253, EPI_ISL_2232254, EPI_ISL_2232255, EPI_ISL_2232256, EPI_ISL_2232257, EPI_ISL_2232258, EPI_ISL_2232259, EPI_ISL_2232260, EPI_ISL_2232261, EPI_ISL_2232262, EPI_ISL_2232263, EPI_ISL_2232264, EPI_ISL_2232265, EPI_ISL_2232266, EPI_ISL_2232267, EPI_ISL_2232268, EPI_ISL_2232269, EPI_ISL_2232270, EPI_ISL_2232271, EPI_ISL_2232272, EPI_ISL_2232273, EPI_ISL_2232274, EPI_ISL_2232275, EPI_ISL_2232276, EPI_ISL_2232277, EPI_ISL_2232278, EPI_ISL_2232279, EPI_ISL_2232280, EPI_ISL_2232281, EPI_ISL_2232282, EPI_ISL_2232283, EPI_ISL_2232284, EPI_ISL_2232285, EPI_ISL_2232286, EPI_ISL_2232287, EPI_ISL_2232288, EPI_ISL_2232289, EPI_ISL_2232290, EPI_ISL_2232291, EPI_ISL_2232292, EPI_ISL_2232293, EPI_ISL_2232294, EPI_ISL_2232295, EPI_ISL_2232296, EPI_ISL_2232297, EPI_ISL_2232298, EPI_ISL_2232299, EPI_ISL_2232300, EPI_ISL_2232301, EPI_ISL_2232302, EPI_ISL_2232303, EPI_ISL_2232304, EPI_ISL_2232305, EPI_ISL_2232306, EPI_ISL_2232307, EPI_ISL_2232308, EPI_ISL_2232309, EPI_ISL_2232310, EPI_ISL_2232311, EPI_ISL_2232312, EPI_ISL_2232313, EPI_ISL_2232314, EPI_ISL_2232315, EPI_ISL_2232316, EPI_ISL_2232317, EPI_ISL_2232318, EPI_ISL_2232319, EPI_ISL_2232320, EPI_ISL_2232321, EPI_ISL_2232322, EPI_ISL_2232323, EPI_ISL_2232324, EPI_ISL_2232325, EPI_ISL_2232326, EPI_ISL_2232327, EPI_ISL_2232328, EPI_ISL_2232329, EPI_ISL_2232330, EPI_ISL_2232331, EPI_ISL_2232332, EPI_ISL_2232333, EPI_ISL_2232334, EPI_ISL_2232335, EPI_ISL_2232336, EPI_ISL_2232337, EPI_ISL_2232338, EPI_ISL_2232339, EPI_ISL_2232340, EPI_ISL_2232341, EPI_ISL_2232342, EPI_ISL_2232343, EPI_ISL_2232344, EPI_ISL_2232345, EPI_ISL_2232346, EPI_ISL_2232347, EPI_ISL_2232348, EPI_ISL_2232349, EPI_ISL_2232350, EPI_ISL_2232351, EPI_ISL_2232352, EPI_ISL_2232353, EPI_ISL_2232354, EPI_ISL_2232355, EPI_ISL_2232356, EPI_ISL_2232357, EPI_ISL_2232358, EPI_ISL_2232359, EPI_ISL_2232360, EPI_ISL_2232361, EPI_ISL_2232362, EPI_ISL_2232363, EPI_ISL_2232364, EPI_ISL_2232365, EPI_ISL_2232366, EPI_ISL_2232367, EPI_ISL_2232368, EPI_ISL_2232369, EPI_ISL_2232370, EPI_ISL_2232371, EPI_ISL_2232372, EPI_ISL_2232373, EPI_ISL_2232374, EPI_ISL_2232375, EPI_ISL_2232376, EPI_ISL_2232377, EPI_ISL_2232378, EPI_ISL_2232379, EPI_ISL_2232380, EPI_ISL_2232381, EPI_ISL_2232382, EPI_ISL_2232383, EPI_ISL_2232384, EPI_ISL_2232385, EPI_ISL_2232386, EPI_ISL_2232387, EPI_ISL_2232388, EPI_ISL_2232389, EPI_ISL_2232390, EPI_ISL_2232391, EPI_ISL_2232392, EPI_ISL_2232393, EPI_ISL_2232394, EPI_ISL_2232395, EPI_ISL_2232396, EPI_ISL_2232397, EPI_ISL_2232398, EPI_ISL_2232399, EPI_ISL_2232400, EPI_ISL_2232401, EPI_ISL_2232402, EPI_ISL_2232403, EPI_ISL_2232404, EPI_ISL_2232405, EPI_ISL_2232406, EPI_ISL_2232407, EPI_ISL_2380050, EPI_ISL_2380051, EPI_ISL_2380052, EPI_ISL_2380053, EPI_ISL_2380054, EPI_ISL_2380055, EPI_ISL_2380056, EPI_ISL_2380057, EPI_ISL_2380058, EPI_ISL_2380059, EPI_ISL_2380060, EPI_ISL_2380061, EPI_ISL_2380062, EPI_ISL_2380063, EPI_ISL_2380064, EPI_ISL_2380065, EPI_ISL_2380066, EPI_ISL_2380067, EPI_ISL_2380068, EPI_ISL_2380069, EPI_ISL_2380070, EPI_ISL_2380071, EPI_ISL_2380072, EPI_ISL_2380073, EPI_ISL_2380075, EPI_ISL_2380076, EPI_ISL_2380077, EPI_ISL_2380078, EPI_ISL_2380079, EPI_ISL_2380080, EPI_ISL_2380081, EPI_ISL_2380082, EPI_ISL_2380083, EPI_ISL_2380084, EPI_ISL_2380085, EPI_ISL_2380086, EPI_ISL_2380087, EPI_ISL_2380088, EPI_ISL_2380089, EPI_ISL_2380090, EPI_ISL_2380091, EPI_ISL_2380092, EPI_ISL_2380093, EPI_ISL_2380094, EPI_ISL_2380095, EPI_ISL_2380096, EPI_ISL_2380097, EPI_ISL_2380098			
see above	Center of Scientific Excellence for Influenza Viruses (CSEIV), National Research Centre	Center of Scientific Excellence for Influenza Viruses (CSEIV), National Research Centre	Ahmed E Kayed; Ahmed El-Taweel; Ahmed Kandeil; Ahmed Mostafa; Amal Naguib; Ghazi Kayali; M K Khalifa; Mahmoud Shehata; Mina Kamel; Mohamed Ahmed Ali; Mohamed El Sayes; Mohamed Hassany; Mokhtar Gomaa; Nancy M. El Guindy; Noura M Abo Shama; Omnia Kutkat; Rabeh El-Shesheny; Richard Webby; Sara Mahmoud; Shymaa S Ahmed; Wael Roshdy; Yassmin Moatasim
EPI_ISL_430820	Center of Scientific Excellence for Influenza Viruses, National Research Centre (NRC), Egypt.	Center of Scientific Excellence for Influenza Viruses, National Research Centre (NRC), Egypt.	Abo Shama; Ahmed E Kayed; Ahmed El-Taweel; Ahmed Kandeil; Ahmed Mostafa; Amal Naguib; M Noura; Mahmoud Shehata; Mina Kamel; Mohamed Ahmed Ali; Mohamed El Sayes; Mokhtar Gomaa; Nancy M. El Guindy; Omnia Kutkat; Rabeh El-Shesheny; Sara Mahmoud; Shymaa Showky Ahmed; Wael Roshdy; Yassmin Moatasim
EPI_ISL_430819	Center of Scientific Excellence for Influenza Viruses,National Research Centre (NRC), Egypt.	Center of Scientific Excellence for Influenza Viruses,National Research Centre (NRC), Egypt.	Abo Shama; Ahmed E Kayed; Ahmed El-Taweel; Ahmed Kandeil; Ahmed Mostafa; Amal Naguib; M Noura; Mahmoud Shehata; Mina Kamel; Mohamed Ahmed Ali; Mohamed El Sayes; Mokhtar Gomaa; Nancy M. El Guindy; Omnia Kutkat; Rabeh El-Shesheny; Sara Mahmoud; Shymaa Showky Ahmed; Wael Roshdy; Yassmin Moatasim
EPI_ISL_406031	Centers for Disease Control, R.O.C. (Taiwan)	Centers for Disease Control, R.O.C. (Taiwan)	Ji-Rong Yang; Jung-Jung Mu; Ming-Tsan Liu; Shu-Ying Li; Yu-Chi Lin
EPI_ISL_815257, EPI_ISL_815368	Centogene	Centogene	Krishna Kumar Kandaswamy; Peter Bauer; Vivi Hue-Trang Lieu
EPI_ISL_2123097	Centogene; Dr. Bauer Laboratoriums GmbH	Robert Koch Institute	
EPI_ISL_2834910, EPI_ISL_2834911, EPI_ISL_2834914, EPI_ISL_2834915	Central Health Laboratory	Central Health Laboratory ,Victoria Hospital, Candos,Ministry of Health and Wellness, Mauritius	Bahadoor BS; Jannoo N; Manraj SS; Mathur H; Ramuth M; Sonoo J; Sujeewon C
EPI_ISL_2834925, EPI_ISL_2834926, EPI_ISL_2834927, EPI_ISL_2834928	Central Health Laboratory/Airport Health Laboratory	Central Health Laboratory ,Victoria Hospital, Candos,Ministry of Health and Wellness, Mauritius	Bahadoor BS; Jannoo N; Manraj SS; Mathur H; Patoo M; Ramuth M; Sonoo J; Sujeewon C

EPI_ISL_794593, EPI_ISL_794594, EPI_ISL_794595, EPI_ISL_794596, EPI_ISL_794597, EPI_ISL_794598, EPI_ISL_794599, EPI_ISL_794600, EPI_ISL_794601, EPI_ISL_794602, EPI_ISL_794603, EPI_ISL_794604, EPI_ISL_2313046, EPI_ISL_2313047, EPI_ISL_2313048, EPI_ISL_2313049, EPI_ISL_2313050, EPI_ISL_2313051, EPI_ISL_2313052, EPI_ISL_2313053, EPI_ISL_2313054, EPI_ISL_2313055, EPI_ISL_2313056, EPI_ISL_2313057, EPI_ISL_2313058, EPI_ISL_2313059, EPI_ISL_2313060, EPI_ISL_2313061, EPI_ISL_2313062, EPI_ISL_2313063, EPI_ISL_2313064, EPI_ISL_2313065, EPI_ISL_2313066, EPI_ISL_2313067, EPI_ISL_2313068, EPI_ISL_2313069			
see above	Central Laboratories, Egyptian Ministry of Health and Population	Central Laboratories, Egyptian Ministry of Health and Population	A.E.; Ali; El Guindy; El Sayes; El Taweel, A.; El-Shesheny; El-Shesheny, R.; Gomaa, M.; Hassany, M.; Kamel; Kandeil, A.; Kayali, G.; Kayed; Khalifa; Kutkat, O.; M.A.; M.K.; M.N.; Mahmoud; Mahrous, N.; Moatasim, Y.; Mostafa, A.; N.M.; Naguib, A.; R.E.; Roshdy; S.H.; Saleh, M.; Shawky, S.; Shehata, M.; W.H.
EPI_ISL_2502381, EPI_ISL_2502387, EPI_ISL_2502393, EPI_ISL_2502402, EPI_ISL_2502426, EPI_ISL_2502427, EPI_ISL_2502430, EPI_ISL_2502435, EPI_ISL_2502438, EPI_ISL_2502441			
see above	Central Laboratory, Bureau of Public Health (BOG) and Academic Hospital Paramaribo	Erasmus Medical Center	Bas B Oude Munnink; Cherise Beek; Consuella Partowidjojo; Dion Gajadin; Ed PF IJzerman; Emmanuelle Munger; Gary Gummels; Ingrid SK Krishnadath; Lycke Woittiez; Marion PG Koopmans; Mireille Van de Veer; Phyllis Pinas; Princes Wongsowidjojo; Radjesh Ori; Ranisha Doerbalie; Rohma Banwari; Soeradj Harkisoen; Stephen Vreden; Tilotmadebie Ramlal; Verne Nanhoe
EPI_ISL_1822763, EPI_ISL_1822784	Central Medical Laboratory	Texas Children's Microbiome Center	Adrianna Maliga; Francis Morey; Gerhaldine Morazan; Jennifer K. Spinler; Kristy O. Murray; Melissa Diaz-Musa; Miguel Saldana; Russell I Manzanero; Ruth Ann Luna; Sarah M. Gunter; Shannon E. Ronca
EPI_ISL_529032	Central Molecular Microbiology Laboratory and Next Generation Sequencing Reference Laboratory, Clinical and Chemical Pathology Department, Faculty of Medicine, CAIRO UNIVERSITY	Next Generation Sequencing Reference Laboratory, Faculty of Medicine, CAIRO UNIVERSITY and The Center for Genome and Microbiome Research, Faculty of Pharmacy, CAIRO UNIVERSITY	May Abdelfattah; May Sherif Soliman; Ramy Karam Aziz
EPI_ISL_529031	Central Molecular Microbiology Laboratory, Clinical and Chemical Pathology Department, Faculty of Medicine, CAIRO UNIVERSITY	Next Generation Sequencing Reference Laboratory, Faculty of Medicine, Cairo University and The Center for Genome and Microbiome Research, Faculty of Pharmacy, CAIRO UNIVERSITY	May Abdelfattah; May Sherif Soliman; Ramy Karam Aziz
EPI_ISL_2464001	Central Public Health Lab, National Public Health Organization	Central Public Health Lab, National Public Health Organization	Gregory Spanakos et al; Kyriaki Tryfinopoulou
EPI_ISL_794605, EPI_ISL_794606, EPI_ISL_794607, EPI_ISL_794608	Central Public Health Laboratories, Egyptian Ministry of Health and Population	Central Public Health Laboratories, Egyptian Ministry of Health and Population	A.E.; Ali; El Guindy; El Sayes; El Taweel, A.; El-Shesheny, R.; Gomaa, M.; Kamel; Kandeil, A.; Kayali, G.; Kayed; Khalifa; Kutkat, O.; M.A.; M.K.; M.N.; Mahmoud; Mahrous, N.; Moatasim, Y.; Mostafa, A.; N.M.; Naguib, A.; Roshdy; S.H.; Saleh, M.; Shawky, S.; Shehata, M.; W.H.
EPI_ISL_693471, EPI_ISL_693473, EPI_ISL_693474, EPI_ISL_693477, EPI_ISL_693478, EPI_ISL_693481, EPI_ISL_693482, EPI_ISL_693483, EPI_ISL_1307643, EPI_ISL_1307660, EPI_ISL_1322329, EPI_ISL_1322330			
see above	Central Public Health Laboratory	National Public Health Laboratory, National Centre for Infectious Diseases	Esorom Daoni; Lin Cui; Raymond Tzer Pin Lin; Sophie Octavia; Theresa Palou; Tze Minn Mak; Zhenyang Zhou
EPI_ISL_978512	Central Public Health Laboratory - LACEN -Bahia, Salvador, Brazil	Central Public Health Laboratory - LACEN -Bahia, Salvador, Brazil	Arabela Leal; Breno Dominguez; Felicidade Pereira; Jaqueline Gomes; Luciana Oliveira; Luiz Alcantara; Marcela Gómez; Marta Giovanetti; Patrícia Cajado; Stephane Tosta; Vagner Fonseca; Vanessa Nardy
EPI_ISL_3045139, EPI_ISL_3045144, EPI_ISL_3045167, EPI_ISL_3045179, EPI_ISL_3045180, EPI_ISL_3045220	Central Public Health Laboratory, National Public Health Organization	Central Public Health Laboratory, National Public Health Organization	A.Katsoulidou et al; G.Spanakos; Kyriaki Tryfinopoulou
EPI_ISL_2232157	Central Public Health Laboratory, National Public Health Organization	Greek Genome Center, Biomedical Research Foundation of the Academy of Athens (BRFAA)	Dimitrios Thanos; Emmanouil Athanasiadis; Giannis Vatsellas; Katerina Zoi; Kyriaki Tryfinopoulou; Theodoros Loupis
EPI_ISL_2786923	Central Virology Laboratory, Ministry of Health	Central Virology Laboratory, Ministry of Health	Amos Adler; Efrat Bucris; Ella Mendelson; Michal Mandelboim; Moran Shwartswort-Cohen; Neta S. Zuckerman; Noam Protter; Oran Erster; Orna Mor; Saar Burstein
EPI_ISL_2141877	Centrala Laboratorija	Latvian Biomedical Research and Study Centre	Davids Fridmanis; Elina Dimina; Guntars Zarins; Ivars Silamikelis; Jana Osite; Janis Klovins; Janis Pjalkovskis; Jurijs Perevoscikovs; Kaspars Megnis; Laila Silamikele; Lauma Freimane; Laura Ansone; Liga Birzniece; Marta Priedite; Monta Ustinova; Nikita Zrelovs; Uga Dumpis; Una Krumina; Vita Rovite
EPI_ISL_961838, EPI_ISL_1312520	Centrala laboratorija	Latvian Biomedical Research and Study Centre	Davids Fridmanis; Davids Fridmanis; Guntars Zarins; Ivars Silamikelis; Ivars Silamikelis; Jana Osite; Janis Klovins; Janis Pjalkovskis; Janis Klovins; Jurijs Perevoscikovs; Kaspars Megnis; Kaspars Megnis; Laila Silamikele; Laila Silamikele; Lauma Freimane; Lauma Freimane; Laura Ansone; Laura Ansone; Liga Birzniece; Liga Birzniece; Marta Priedite; Monta Ustinova; Monta Ustinova; Nikita Zrelovs; Nikita Zrelovs; Uga Dumpis; Uga Dumpis; Una Krumina; Vita Rovite; Vita Rovite
EPI_ISL_1357603	Centre For Biotechnology Research And Development	THE AFRICA GENOMICS CENTRE AND CONSULTANCY	Adede Hawi; Cecilia Waruhiu; Damaris Matoke-Muhia; George Michuki; John Njuguna; Lilian Kanjau; Ravena Mubichi
EPI_ISL_414625	Centre Hospitalier Régional Universitaire de Nantes Laboratoire de Virologie	National Reference Center for Viruses of Respiratory Infections, Institut Pasteur, Paris	Angela Brisebarre; Flora Donati Vincent Enouf; Marianne Coste-Burel; Marion Barbet; Maud Vanpeene; Méline Bizard; Mélnie Albert; Sylvie Behillil; Sylvie van der Werf
EPI_ISL_1001044, EPI_ISL_1001045, EPI_ISL_1001050, EPI_ISL_1001051, EPI_ISL_1001053, EPI_ISL_1001059, EPI_ISL_1001062, EPI_ISL_1001064, EPI_ISL_1001068, EPI_ISL_1790120, EPI_ISL_1790131			
see above	Centre Pasteur du Cameroun	Institut Pasteur de Dakar	Carniel Elisabeth; Dia Ndongo; Diagne Moussa Moïse; Diallo Amadou; Diop Mamadou; Faye Ousmane; Loucoubar Cheikh; Ndiaye Ndack; Njouom Richard; Sall Amadou Alpha; Sankhe Safietou
EPI_ISL_788936, EPI_ISL_788938	Centre de Recherche et de Formation en Infectiologie Guinée	TransVIHMI, IRD/INSERM/Monpellier University	Abdoul Karim SOUMAH; Abdoulaye TOURE; Ahidjo AYOUBA; Alimou CAMARA; Alpha Kabinet KEITA; Bouna Yatassaye; Christelle BUTEL; Eric DELAPORTE; Jean-louis MONEMOU; Joel KOIVOGUI; Kaba KOUROUMA; Laetitia SERRANO; Mamadou Bhoye KEITA; Mamadou Saliou BAH; Mamadou Saliou SOW; Mandiou DIAKITE; Martine PEETERS; Moriba POVOGUI; Penda Malhado DIALLO; Sakoba KEITA
EPI_ISL_1615655, EPI_ISL_1913075, EPI_ISL_1913078, EPI_ISL_1913080, EPI_ISL_2097218, EPI_ISL_2097219, EPI_ISL_2097220, EPI_ISL_2097221, EPI_ISL_2097223, EPI_ISL_2097228, EPI_ISL_2097229, EPI_ISL_2097230, EPI_ISL_2097231, EPI_ISL_2156795, EPI_ISL_2156822, EPI_ISL_2424136, EPI_ISL_2424137, EPI_ISL_2424157, EPI_ISL_2424158, EPI_ISL_2424159, EPI_ISL_2424160, EPI_ISL_2424161, EPI_ISL_2424162, EPI_ISL_2424163, EPI_ISL_2434970, EPI_ISL_2434971, EPI_ISL_2434974, EPI_ISL_2434975, EPI_ISL_2434979, EPI_ISL_2434980, EPI_ISL_2442203, EPI_ISL_2442276, EPI_ISL_2442277, EPI_ISL_2442279, EPI_ISL_2442338, EPI_ISL_2442363, EPI_ISL_2442383, EPI_ISL_2442384			
see above	Centre de Recherches Médicales de Lambaréné (CERMEL)	Centre de Recherches Médicales de Lambaréné (CERMEL)	Anicet Mouity Matoumba; Armel MINTSA NDONG; Bertrand Lell and Ayola Akim Adegnika; Bénédicte NDEBOKO; Georgelin NGUEMA ONDO; Georgelin Nguema Ondo; Gédéon Prince Manouana; Jean Bernard LEKANA-DOUKI; Jean Bernard Lekana-Douki; Joël-Fleury DJOBA SIAWAYA; Joël-Fleury Djoba Siawaya; Michel Ngonga Dikongo; Moustapha Nzamba Maloum; Rodrigue BIKANGUI; Rodrigue Bikangui; Sam O'neilla Oye Bingono; Samira ZOA ASSOUMOU; Samira Zoa Assoumou; Srinivas reddy Pallerla; Steffen Borrmann; Thirumalaisamy P. Velavan
EPI_ISL_978545, EPI_ISL_978548, EPI_ISL_2968046, EPI_ISL_2968064, EPI_ISL_2968065, EPI_ISL_2968069	Centre de Virologie des Maladies infectueuses Tropicales	Functional Genomic Platform UATRS-biology, CNRST	; Abdelilah LARAQUI; Abderrazzak Rfaki; Ahmed REGGAD; Charifa DRISSI TOUZANI; Elmostafa BENAISSA; Elmostafa EL FAHIME; Farida HILALI; Hemlali Mouhssine; Hicham EL ANNAZ; Idriss-Amine LAHLOU; Ikram BNOUYAHIA; Khalid ENNIBI; Marouane MELLOUL; Mly Abdelaziz ELALAOUI; Mostafa ELOUENNASS; Nadia EL MRIMAR; Nadia TOUIL; Nadia Touil; Rachid ABI; Rida TAGAJDID; Safae ELKOCHRI; Sanaa ALAOUI-Amine; Tahar BAJJOU; Yassine SEKHSOKH; Youssef AKHOUAD; Zohour KASMY
EPI_ISL_471456	Centre de Virologie des Maladies Tropicales	Functional Genomic Platform/Service Analyses Biologique/UATRS/ Centre National Pour la Recherche Scientifique Et Technique (CNRST)	Abdelillah LARAQUI; Abdelkader LAATIRIS; Ahmed REGGAD; Elmostafa EL FAHIME; Farida HILALI; Hicham EL ANNAZ; Idriss-Amine LAHLOU; Khalid ENNIBI; Marouane MELLOUL; Mly Abdelaziz ELALAOUI; Mostafa ELOUENNASS; Nadia TOUIL; Rachid ABI; Rida TAGAJDID; Safae ELKOCHRI; Sanaa ALAOUI-Amine; Tahar BAJJOU; Yassine SEKHSOKH; Youssef AKHOUAD; Zohour KASMY
EPI_ISL_792550, EPI_ISL_1233067, EPI_ISL_1717082, EPI_ISL_1717092, EPI_ISL_1717093, EPI_ISL_1970356, EPI_ISL_1970358, EPI_ISL_1970371, EPI_ISL_1970374, EPI_ISL_1970399, EPI_ISL_1970410, EPI_ISL_1970413, EPI_ISL_2481412, EPI_ISL_2481418, EPI_ISL_2481425, EPI_ISL_2803170, EPI_ISL_2803176, EPI_ISL_2803179, EPI_ISL_2803193, EPI_ISL_2803199, EPI_ISL_2803251, EPI_ISL_2803298			
see above	Centre for Dengue Research and AICBU, Department of Immunology and Molecular Medicine	Centre for Dengue Research and AICBU, Department of Immunology and Molecular Medicine	Chandima Jeewandara; Deshan Madhusanka; Deshni Jayathilaka; Dinuka Ariyaratne; Diyanath Ranasinghe; Gathsaurie Neelika Malavige; Laksiri Gomes; Tibutius Thanesh Pramanayagam
EPI_ISL_668448, EPI_ISL_668449, EPI_ISL_668452, EPI_ISL_668455	Centre for Dengue Research, Department of Immunology and Molecular Medicine	Centre for Dengue Research, Department of Immunology and Molecular Medicine	Chandima Jeewandara; Deshni Jayathilaka; Dinuka Ariyaratne; Diyanath Ranasinghe; Gathsaurie Neelika Malavige; Laksiri Gomes
EPI_ISL_602565	Centre for Dengue Research, Department of Immunology and Molecular Medicine	Centre for Dengue Research, Department of Immunology and Molecular Medicine,	Chandima Jeewandara; Deshni Jayathilaka; Dinuka Ariyaratne; Dinuka Guruge; Diyanath Ranasinghe; Gathsaurie Neelika Malavige; Laksiri Gomes; Ruwan Wiajayamuni
EPI_ISL_679977, EPI_ISL_763605, EPI_ISL_841936	Centre for Enzyme Innovation, University of Portsmouth / Translational Research Laboratory, Portsmouth Hospitals NHS Trust	COVID-19 Genomics UK (COG-UK) Consortium	Allyson Lloyd; Angela Beckett; Anoop Chauhan; Ethan Butcher; Garry Scarlett; Kelly Bicknell; Robert Impey; Samuel Robson; Sarah Wyllie; Scott Elliott; Sharon Glaysher; Yann Bourgeois
EPI_ISL_654794	Centre for Human Virology & Genomics, Nigerian Institute of Medical Research	Centre for Human Virology & Genomics, Nigerian Institute of Medical Research	Shaibu, J.
EPI_ISL_2399460, EPI_ISL_2399462, EPI_ISL_2502019	Centre for Human Virology and Genomics, Microbiology Department, Nigerian Institute of Medical Research	Central Research Laboratory, Nigerian Institute of Medical Research	Ayorinde Babatunde James; Azuka Patrick Okwuraiwe; Babatunde Lawal Salako; Bamidele Iwalokun; Chika Kingsley Onwuamah; Grace Oni; Joseph Ojonugwa Shaibu; Josiah Ayoola Isong; Joy Ayoola; Muinah Adenike Fowora; Ngozi Mirabel Otuonye; Nyam Itse Yusuf; Olufemi Samuel Amoo; Phasha-Muchemenye Mmatshepho; Rahaman A. Ahmed; Rosemary Ajuma Audu; Sharon Abimbola; Sola Ajibaye; Uyi Emokpae; Yusuf Jimoh
EPI_ISL_413550	Centre for Human and Zoonotic Virology (CHAZVY), College of Medicine University of Lagos/Lagos University Teaching Hospital (LUTH), part of the Laboratory Network of the Nigeria Centre for Disease Control (NCDC)	African Centre of Excellence for Genomics of Infectious Diseases (ACEGID), Redeemer's University, Ede, Osun State, Nigeria	Ajogbasile F.V.; Folarin O.A.; Ihekweazu C. Happi C.T.; Kayode A.; Oguzie J.; Oluniyi P.E.
EPI_ISL_407893, EPI_ISL_413594	Centre for Infectious Diseases and Microbiology Laboratory Services	NSW Health Pathology - Institute of Clinical Pathology and Medical Research; Westmead Hospital; University of Sydney	; A; Alicia; Carter I; Chen SC; Eden J-S; Gall; Gray K; Holmes EC; Kok J and Dwyer DE for the 2019-nCoV Study Group; Kok J and Dwyer DE for the 2019-nCoV Study Group*; Lam C; M; Maddocks S; O'Sullivan MV; Rahman H; Rockett R; Sintchenko V; Timms; V
EPI_ISL_2842788	Centro Medico ABC	Instituto Nacional de Medicina Genomica	Cedro-Tanda A; Cesar Lara; Daniel Aguirre; Escobar-Arrazola; Gonzalez-Barrera D; Herrera-Montalvo LA.; Hidalgo-Miranda A; Jacomine Reyes-Carrasco; M; Mendoza-Vargas A; Munguia-Garza P; Ramirez-Vega O; Rangel-DeLeon D; Reyes-Grajeda JP; Roxana Trejo-González
EPI_ISL_837557, EPI_ISL_837559, EPI_ISL_837564, EPI_ISL_837565, EPI_ISL_837569, EPI_ISL_837572, EPI_ISL_837573, EPI_ISL_837577, EPI_ISL_837578			
see above	Centro Nacional de Enfermedades Tropicales (CENETROP)	Laboratory of Respiratory Viruses and Measles, Oswaldo Cruz Institute, FIOCRUZ	Ana Carolina Mendonca; Ana Carolina Paixao; Cinthia Avila; Fernando Motta; Luciana Appolinario; Marilda Siqueira on behalf of the Fiocruz COVID-19 Genomic Surveillance Network; Paola Resende; Roxana Loayza
EPI_ISL_2274031, EPI_ISL_2274037, EPI_ISL_2274038	Centro Nacional de Enfermidades Tropicales (CENETROP)	Laboratory of Respiratory Viruses and Measles, Oswaldo Cruz Institute, FIOCRUZ	Alice Sampaio Rocha; Ana Carolina Mendonca; Ana Carolina Paixao; Cinthia Avila; Elisa Cavalcante Pereira; Fernando Motta; Luciana Appolinario; Marilda Siqueira on behalf of the Fiocruz COVID-19 Genomic Surveillance Network; Paola Resende; Renata Serrano Lopes; Roxana Loayza; Taina Venas

EPI_ISL_1531863, EPI_ISL_2231524	Centro de Diagnostico COVID-19 UABC Tijuana	Andersen lab at Scripps Research	German Ibarra; Jonathan Vincent Baena; Jorge Luis Jimenez Niebla; Manuel Sanchez Alavez; Oscar Efren Zazueta Fierro; SEARCH Alliance San Diego with Idanya Rubi Serafin Higuera
EPI_ISL_635531	Centro de Diagn√≥stico COVID-19 UABC Tijuana	Andersen lab at Scripps Research	Germán Ibarra; Jonathan Vincent Baena; Jorge Luis Jiménez Niebla; Manuel Sánchez Alavez; Oscar Efrén Zazueta Fierro; SEARCH Alliance San Diego with Idanya Rubí Serafín Higuera
EPI_ISL_2339876, EPI_ISL_2339903, EPI_ISL_2339907	Centro de Estudio de Enfermedades Autoinmunes (CREA), Universidad del Rosario, Bogota, Colombia	Centro de Investigaciones en Microbiología y Biotecnología-UR (CIMBIUR), Facultad de Ciencias Naturales, Universidad del Rosario, Bogotá, Colombia	Carolina Ramírez-Santana; Gustavo Salguero; Juan David Ramírez; Juan Esteban Gallo; Juan-Manuel Anaya; Luz H. Patiño; Marina Muñoz; Nathalia Ballesteros; Sergio Castañeda
EPI_ISL_2031726	Centro de Innovación en Vigilancia Epidemiológica (CiVE), Institut Pasteur Montevideo, Uruguay	Centro de Innovación en Vigilancia Epidemiológica (CiVE), Institut Pasteur Montevideo, Uruguay	Alicia Costábile; Alvaro Fajardo; Andrés Lizosaín; Belén González; Bernardina Rivera; Cecilia Alonso; Cecilia Salazar; Gonzalo Moratorio; Gregorio Iraola; Henry Albornoz; Ignacio Ferrés; Inés Bellini; Juan Zanetti; Julio Medina; Lucia Bilbao; Luciana Griffero; Lucía Spangenberg; Ma Noel Bentancor; Ma Pía Techera; Mailen Arleo; Martina Alonso; María José Benítez; Matías Maidana; Mauricio Méndez; Melissa Duquía; Mercedes Paz; Natalia Rego; Natalia Reyes; Odhille Chappos; Paula Perbolianachis; Pilar Moreno; Rodney Colina; Rodrigo Arce; Tamara Fernández; Tania Possi
EPI_ISL_941116, EPI_ISL_941123, EPI_ISL_941139	Centro de Investigaciones en Microbiología y Biotecnología-UR (CIMBIUR), Facultad de Ciencias Naturales, Universidad del Rosario, Bogotá, Colombia	Centro de Investigaciones en Microbiología y Biotecnología-UR (CIMBIUR), Facultad de Ciencias Naturales, Universidad del Rosario, Bogotá, Colombia Icahn School of Medicine at Mount Sinai, New York, USA	Adriana van de Guchte; Alberto Paniz-Mondolfi; Alejandro Feged-Rivadeneira; Ana S. Gonzalez-Reiche; Andrés Angel; Carolina Flórez; Carolina Hernández; Emilia Mia Sordillo; Felipe González-Casabianca; Hala Alejel Alshammary; Harm van Bakel; Iván Carroll; Jaime Cascante; Jayeeta Dutta; Juan David Ramírez; Luz Helena Patiño; Marina Muñoz; Matthew M. Hernandez; Mauricio Santos-Vega; Mónica Palma-Cuero; Nathalia Ballesteros; Sergio Gomez; Viviana Simon; Zenab Khan
EPI_ISL_941982	Centro de Investigaciones en Microbiología y Biotecnología-UR (CIMBIUR), Facultad de Ciencias Naturales, Universidad del Rosario, Bogotá, Colombia	Centro de Investigaciones en Microbiología y Biotecnología-UR (CIMBIUR), Facultad de Ciencias Naturales, Universidad del Rosario, Bogotá, Colombia Instituto Nacional de Salud, Bogotá, Colombia Icahn School of Medicine at Mount Sinai, New York, USA	Adriana van de Guchte; Alberto Paniz-Mondolfi; Ana S. Gonzalez-Reiche; Carolina Flórez; Carolina Hernández; Emilia Mia Sordillo; Hala Alejel Alshammary; Harm van Bakel; Jayeeta Dutta; Juan David Ramírez; Luz Helena Patiño; Marina Muñoz; Matthew M. Hernandez; Nathalia Ballesteros; Sergio Gomez; Viviana Simon; Zenab Khan
EPI_ISL_491941	Centro de Investigaciones, Universidad de Especialidades Espíritu Santo	Institute of Microbiology, Universidad San Francisco de Quito	Belén Prado-Vivar; Bernardo Gutiérrez; Derly Andrade; Edith Lopez; Fernando Espinoza; Gabriel Morey; Gabriel Trueba; Jose Pedro Barberan; Juan Carlos Fernandez; Juan José Guadalupe; Michelle Grunauer; Monica Becerra-Wong; Patricio Rojas-Silva; Paúl Cárdenas; Ruben Armas; Sully Márquez; Verónica Barragán
EPI_ISL_2442098, EPI_ISL_2442113	Centro de Investigação em Saúde de Manhiça (CISM) & ISGlobal, Institut de Salut Global de Barcelona	Instituto de Biomedicina de València - CSIC	Alfredo Mayor; Arsenia Massinga; Inacio Mandomando; Irving Cancino; Iñaki Comas; Manoli Torres Puente; Santiago Jiménez-Serrano
EPI_ISL_450523	Centrālā Laboratorija	Latvian Biomedical Research and Study Centre	Ivars Silamiķelis; Jana Osīte; Jānis Kloviņš; Kaspars Megnis; Marta Priedīte; Monta Ustinova; Stella Lapiņa; Uga Dumpis; Vita Rovīte; Ņikita Zrelovs
EPI_ISL_2798876, EPI_ISL_2798895, EPI_ISL_2798922, EPI_ISL_2798996, EPI_ISL_2799000, EPI_ISL_2799099, EPI_ISL_2799215, EPI_ISL_2799803			
see above	Centrālā Laboratorija, SIA	Riga East University Hospital, National Microbiology Reference Laboratory; Eurofins Genomics Europe Sequencing GmbH	Arzu Algulieva; Diāna Dušacka; Dārta Pūpola; Ilva Pole; Jana Osīte; Jevgenijs Bodrenko; Jūlija Čevere; Reinis Vangravs; Reinis Zeltmatis; Sergejs Ņikišins; Stella Lapiņa; Ģirts Šķenders
EPI_ISL_486412, EPI_ISL_492988	Centrālā laboratorija	Latvian Biomedical Research and Study Centre	Ivars Silamiķelis; Jana Osīte; Jānis Kloviņš; Kaspars Megnis; Marta Priedīte; Monta Ustinova; Stella Lapiņa; Uga Dumpis; Vita Rovīte; Ņikita Zrelovs
EPI_ISL_861489	Cerballiance Côte d'Azur	CERBA LAB	Adjtoutah Z; Mangiarotti T; Prots L.
EPI_ISL_2646262	Cerballiance-IDF	Cerba lab	Aude Lessenne; Bénédicte Roquebert; Emmanuel Lecorche; Kader Merah; Laura Verdurme; Patrice Herisson; Sabine Trombert-Paolantoni; Stéphanie Haïm-Boukobza; Thierry Collin
EPI_ISL_2504131, EPI_ISL_2504133, EPI_ISL_2504138, EPI_ISL_2504139, EPI_ISL_2504141, EPI_ISL_2504142	Chantal Biya International Reference Centre (CIRCB)	International Centre for Genetic Engineering and Biotechnology (ICGEB) and ARGO Open Lab for Genome Sequencing	Alessandro Marcello; Alexis Ndjolo; Celine Nkenfou; Collins Chenwi; Danilo Licastro; Desire Takou; Emanuele Orsini; Grace Angong Beloumou; Joseph Fokam; Ndine Fainguem; Simeone Dal Monego
EPI_ISL_753955, EPI_ISL_754011	Charité Universitätsmedizin Berlin, Institut für Virologie/Labor Berlin	Charité Universitätsmedizin Berlin, Institut für Virologie	Barbara Mühlemann; Christian Drosten; Julia Schneider; Jörn Beheim-Schwarzbach; Talitha Veith; Terry Jones; Victor M Corman
EPI_ISL_2987446	Chattogram Maa-O-Shishu Hospital / Disease Biology & Molecular Epidemiology Research Lab, University of Chittagong	Virology Laboratory, International Centre for Diarrhoeal Disease Research, Bangladesh (ICDDR,B)	Adnan Mannan; Fahim Reza; H.M. Hamidullah Mehedi; Md. Abdur Rob; Md. Minhazul Hoque; Mohammad Enayet Hossain; Mojnu Miah; Mustafizur Rahman; Nahid Sultana; Rajdeep Biswas; Rashedul Hasan; Sanjoy Biswas
EPI_ISL_735497	Chattogram Veterinary and Animal Sciences University	Central Biological Research Laboratory and Department of Biochemistry and Molecular Biology	H. M. Abdullah Al Masud; Imam Hossen; Md. Arif Hossain; Md. Imranul Hoq; Md. Khondakar Raziur Rahman; Md. Omer Faruq; Mohammad Omar Faruque; Robiul Hasan Bhuiyan; Sajib Rudra; Shanta Paul
EPI_ISL_1211989	Chemisches und Veterinäruntersuchungsamt Ostwestfalen-Lippe	Robert Koch Institute	
EPI_ISL_419297, EPI_ISL_419298, EPI_ISL_419309	Chiba Prefectural Institute of Public Health	Pathogen Genomics Center, National Institute of Infectious Diseases	Hajime Kamiya; Kentaro Itokawa; Makoto Kuroda; Masakatsu Taira; Masanori Hashino; Motoi Suzuki; Rina Tanaka; Tsuyoshi Sekizuka; Yushi Hachisu
EPI_ISL_700332, EPI_ISL_906087, EPI_ISL_1360422, EPI_ISL_1634454, EPI_ISL_1634455, EPI_ISL_2446012, EPI_ISL_2692860, EPI_ISL_2692865, EPI_ISL_2692876			
see above	Child Health Research Foundation	Child Health Research Foundation	Afroza Akter Tanni; Arif Mohammad Tanmoy; CHRF Bangladesh Genomics Team; Md Hafizur Rahman; Roly Malaker; Samir K Saha; Senjuti Saha; Sharmistha Goswami; Syed Muktadir Al Sium
EPI_ISL_2445988	Chittagong General Hospital	Child Health Research Foundation	Adnan Mannan; CHRF Bangladesh Genomics Team; H. M. Hamidullah Mehedi; Md. Abdur Rob; Md. Minhazul Hoque; Rajdeep Biswas
EPI_ISL_1970348	Chongqing International Travel Health Care Center	Chongqing Municipal Center for Disease Control and Prevention	Dong Wang; Haiyan Wen; Hua Ling; Lan Zhou; Mingyue Wang; Rong Rong; Sheng Ye; Shuang Chen; Wenge Tang; Yun Tang; Zhangping Tan; Zhen Yu
EPI_ISL_1970347	Chongqing Yuzhong District Center For Disease Control And Prevention	Chongqing Municipal Center for Disease Control and Prevention	Hua Ling; Jiaqi Li; Mingyue Wang; Rong Rong; Sheng Ye; Shuang Chen; Wenge Tang; Yingbing Zhou; Yun Tang; Yuyue Luo; Zhangping Tan; Zhen Yu
EPI_ISL_3038908	Chulabhorn Hospital	National Institute of Health, Department of Medical Sciences, Ministry of Public Health, Thailand	; Natchaya Khiadsang; Nuttida Thongpramul; Pakorn Piromtong; Pilailuk Okada; Ratana Tacharoenmuang; Siripaporn Phuygun; Sittiporn Parnmen; Sunthareeya Waicharoen; Thanutsapa Thanadachakul; Warawan Wongboot; sirikanda wimol
EPI_ISL_2248847, EPI_ISL_2248852, EPI_ISL_2248853, EPI_ISL_2445744	Cintramedica	Instituto Nacional de Saude (INSA)	Borges et al
EPI_ISL_1722998, EPI_ISL_1844396	Clemenshospital - Alexianer Clemenshospital MÃ¼nster	Robert Koch Institute	
EPI_ISL_1363787	Clin & Gen Lab	Microbiologia Molecular, Instituto SELADIS, Universidad Mayor de San Andrés	Aneth Vasquez Michel; Oscar M. Rollano-Peñaloza
EPI_ISL_1250842	Clin & Gen Lab	Molecular Genetics Laboratory, Instituto de Investigaciones Químicas, Universidad Mayor de San Andrés	Aneth Vasquez Michel; Oscar M. Rollano-Peñaloza
EPI_ISL_2987572	Clinic of infective diseases	Laboratory of virology and molecular diagnostics, Institute of Public Health	Boshevska G; Janchevska E.; Kuzmanovska M
EPI_ISL_462450, EPI_ISL_462464	Clinical Center, University of Sarajevo	Charite Universitatsmedizin Berlin, Institute of Virology	Almedina Hadzihasanovic-Moro; Amela Dedeic-Ljubovic; Barbara Muehlemann; Christian Drosten; Irma Salimovic-Besic; Jorn Beheim-Schwarzbach; Julia Schneider; Selma Mutevelic; Suzana Arapcic; Talitha Veith; Terry Jones; Victor M Corman
EPI_ISL_1300651, EPI_ISL_2938102, EPI_ISL_2938104, EPI_ISL_2938106, EPI_ISL_2938118	Clinical Center, University of Sarajevo; Unit for Clinical Microbiology	Clinical Center, University of Sarajevo; Unit for Clinical Microbiology	Amela Dedeić-Ljubović; Edina Zahirović; Golubinka Boshevska; Irma Salimović-Bešić; Maja Kuzmanovska; Sandra Vegar-Zubović; Sebija Izetbegović; Suzana Arapčić
EPI_ISL_2652206, EPI_ISL_2652209, EPI_ISL_3021385, EPI_ISL_3021390	Clinical Emergency County Hospital, Craiova	Cantacuzino Institute Virology	Carmen Cherciu; Luiza Ustea; Mihaela Lazar; Mihaela Oprea; Nicoleta Paraschiv; Sorin Dinu
EPI_ISL_516431, EPI_ISL_678259	Clinical Hospital - Shtip	Research Center for Genetic Engineering and Biotechnology "Georgi D. Efremov" , Macedonian Academy of Sciences and Arts	RCGEB - MASA
EPI_ISL_3020158	Clinical Hospital of Infectious Diseases „Sfanta Parascheva" Iasi	National Institute of Infectious Diseases-Prof. Dr. Matei Bals Molecular Diagnostics Laboratory	Corina Casangiu; Dan Otelea; Leontina Banica; Marius Surleac; Petre Milu; Robert Hohan; Simona Paraschiv
EPI_ISL_447454	Clinical Microbiology Laboratory, Sheba Medical Center	Stern Lab	Stern Lab
EPI_ISL_968146, EPI_ISL_1334559	Clinical Molecular Microbiology Laboratory, UNC Hospital	Dirk Dittmer	Andrea McDaniel-Harper; Angelica Juarez; Ashwini Roy-Chaudhury; Billy Fisher; Blossom Damania.; Brent A. Eason; Cameroon Grant; Carolina Caro-Vegas; Cecilia Thompson; Danielle Chappell; Dirk Dittmer; Evelyn Hoffman; Jason Smedberg; Jason Wong; Justin T. Landis; Linda Pluta; Melissa B. Miller; Patricio Cano; Razia Moorad; Ryan McNamara; Shawn Hawken
EPI_ISL_2535703	Clinical Research Centre Hospital Sibu	Institute of Health and Community Medicine	Chan Chia Jui; Chua Hock Hin; David Perera; Ooi Mong How; Tonnii Sia Loong Loong; Wong Jyn Shan; Wong Kiing Aik
EPI_ISL_2608412	Clinical Research Centre, Sarawak General Hospital	Institute of Health and Community Medicine	Chan Chia Jui; Chua Hock Hin; David Perera; Ooi Mong How; Tonnii Sia Loong Loong; Wong Jyn Shan; Wong Kiing Aik
EPI_ISL_581842, EPI_ISL_581843, EPI_ISL_830804, EPI_ISL_930860, EPI_ISL_2102098, EPI_ISL_2270056, EPI_ISL_2768083			

see above	Clinical Virology	Clinical Bacteriology	Adrian Egli; Alexander Gensch; Alfredo Mari; Christian Nickel; Fanny Wegner; Hans Hirsch; Hans Pargger; Helena MB Seth-Smith; Julia Bielicki; Karoline Leuzinger; Kirstine K. Soegaard; Madlen Stange; Manuel Battegay; Martin Siegemund; Michael Osthoff; Michael Schweitzer; Myrta Brunner; Rita Schneider-SliFemalea; Rita Schneider-Sliwa; Roland Bingisser; Sarah Tschudin-Sutter; Simon Fuchs; Stefano Bassetti; Tim Roloff
EPI_ISL_2035560	Clinical Virology	Clinical Virology	Anissa Chouikha; Henda Triki; Kais Ghedira; Mariem Gdoura; Sondos Haddad; Wasfi Fares
EPI_ISL_605804	Clinical Virology Laboratory, Institute of Liver and Biliary Sciences	ILBS - IGIB	Abhishek Padhi; Ekta Gupta; Jaswinder Singh Maras; Reshu Agarwal; Sheetalnath Rooge; Shridhar Sivasubbu; Shvetank Sharma; Vinod Scaria
EPI_ISL_2370909	Clinical and Basic Functional Sciences, Department of Microbiology	Greek Genome Center, Biomedical Research Foundation of the Academy of Athens (BRFAA)	Dimitrios Thanos; Emmanouil Athanasiadis; Giannis Vatsellas; Katerina Zoi; Konstantina Gartzonika; Theodoros Loupis
EPI_ISL_1397826	Columbia University Irving Medical Center	Wadsworth Center, New York State Department of Health	Alexis Russel; Daryl M. Lamson; Erasmus Schneider; Erica Lasek-Nesselquist; John Kelly; Jonathan Plitnick; Kirsten St. George; Matthew Shudt; Melissa A Leisner; Navjot Singh
EPI_ISL_2383896	Commonwealth Healthcare Center	Centers for Disease Control and Prevention Division of Viral Diseases, Pathogen Discovery	Alison Laufer Halpin; Ben L. Rambo-Martin; Clinton R. Paden; Dakota Howard; Darlene Wagner; Dave Wentworth; Dhwani Batra; Jasmine Padilla; Justin Lee; Katie Dillon; Krista Queen; Kristen Knipe; Kristine Lacek; Mark Burroughs; Matthew Schmerer; Mili Sheth; Peter Cook; Sam Shepard; Sarah Nobles; Shoshona Le; Suxiang Tong; Vivien Dugan; Yvette Unoarumhi
EPI_ISL_632271, EPI_ISL_632285, EPI_ISL_682300, EPI_ISL_1660433, EPI_ISL_1660444, EPI_ISL_1660449, EPI_ISL_1660464, EPI_ISL_1660470, EPI_ISL_2466680, EPI_ISL_2622084, EPI_ISL_2663322, EPI_ISL_2663323, EPI_ISL_2715285, EPI_ISL_2715288, EPI_ISL_2715289, EPI_ISL_2812541, EPI_ISL_2932623, EPI_ISL_2932641, EPI_ISL_2932642, EPI_ISL_2932655, EPI_ISL_2932663, EPI_ISL_2932666, EPI_ISL_2932669, EPI_ISL_2932673, EPI_ISL_2932678, EPI_ISL_2992091			
see above	Communicable Disease Laboratory, Public Health Directorate	Communicable Disease Laboratory, Public Health Directorate	AlAbbas, Z.; AlHujairi, Z.; AlTaif, Z.; AlWasti, H.; Almoamen, G.; Altaif, Z.; Alwasti, H.; Marhoon, A.
EPI_ISL_2375921	Concordia Clinic	NHLS/UCT	Arash Iranzadeh; Bruna Galvao; Carolyn Williamson; Deelan Doolabh; Diana Hardie; Innocent Mudau; Kruger Marais; Lynn Tyers; Marvin Hsiao; Stephen Korsman
EPI_ISL_454110	ControlVet	Instituto Nacional de Saude (INSA)	Borges et al
EPI_ISL_2636156	Corona-Testzentrum ifp Institut für Produktqualität GmbH	Robert Koch Institute	
EPI_ISL_3014093, EPI_ISL_3014108, EPI_ISL_3014126	Cotugno	TIGEM	Antonio Grimaldi Patrizia Annunziata Francesco Panariello Biancamaria Pierri Claudia Tiberio Teresa Giuliano Valentina Bouche Chiara Colantuono Maria Concetta Cuomo Denise Di Concilio Lucio Di Filippo Anna Manfredi Marcello Salvi Antonio Limone Luigi Atripaldi Pellegrino Cerino Andrea Ballabio Davide Cacchiarelli
EPI_ISL_1084757	Croatian Institute of Public Health	Croatian Institute of Public Health	Irena Tabain; Ivana Ferenčak
EPI_ISL_1695973	DC Public Health Lab/ Dept. of Forensic Sciences	Centers for Disease Control and Prevention Division of Viral Diseases, Pathogen Discovery	Alison Laufer Halpin; Ben L. Rambo-Martin; Clinton R. Paden; Dakota Howard; Darlene Wagner; Dave Wentworth; Dhwani Batra; Jasmine Padilla; Justin Lee; Katie Dillon; Krista Queen; Kristen Knipe; Kristine Lacek; Mark Burroughs; Matthew Schmerer; Mili Sheth; Peter Cook; Sam Shepard; Sarah Nobles; Shoshona Le; Suxiang Tong; Vivien Dugan; Yvette Unoarumhi
EPI_ISL_1094341	DC Public Health Lab/ Dept. of Forensic Sciences	Respiratory Viruses Branch, Division of Viral Diseases, Centers for Disease Control and Prevention	Anna Montmayeur; Anna Uehara; Ben L. Rambo-Martin; Clinton R. Paden; Dhwani Batra; Haibin Wang; Jasmine Padilla; Jing Zhang; Justin Lee; Krista Queen; Lori Rowe; Mark Burroughs; Mili Sheth; Peter W. Cook; Rachel Marine; Sarah Nobles; Suxiang Tong; Yan Li; Ying Tao
EPI_ISL_2779433	DFMH	USAMRD-A, Basic Science Laboratory	Alan Lemtudo; Beth Mutai; Brian Andika; Carol Kifude; Clement Masakwe; Eric Muthanje; Esther Omuseni; Faith Sigei; Gathii Kimita; George Awinda; John Waitumbi; Josphat Nyataya; Rachel Githii; Rehema Liyai; Stephen Ochola
EPI_ISL_2342561, EPI_ISL_2342562, EPI_ISL_2342563	DNA Laboratories Sdn Bhd	Malaysia Genome Institute	Azrin Ahmad; Enizza Kasim; Irni Suhayu Sapian; Mohd Faizal Abu Bakar; Mohd Noor Mat Isa; Nor Azfa Johari.; Nurhezreen Md Iqbal; Shamsidar Sopie; Siti Noraini Othman; Wong Yong Wee; Yusuf Muhammad Noor
EPI_ISL_2983222, EPI_ISL_2983224, EPI_ISL_2987211, EPI_ISL_2987214	DNA Labs	National Reference Laboratory, Nigeria Centre for Disease Control	Anthony Ahumibe; Catherine Okoi; Chimaobi Chukwu; Dr Chikwe Ihekweazu; Dr Ndodo Nnaemeka; Dr Omoare Adesuyi; Nwando Mba; Olusola Anuoluwapo Akanbi
EPI_ISL_683829	DOHMH Central Harlem	New York City Public Health Laboratory	Jade Wang; et al.
EPI_ISL_823949	DOHMH Jamaica	New York City Public Health Laboratory	Jade Wang; et al.
EPI_ISL_857252	DOHMH Morrisania	New York City Public Health Laboratory	Jade Wang; et al.
EPI_ISL_745438	DOHMH PHL	New York City Public Health Laboratory	Jade Wang; et al.
EPI_ISL_1181670, EPI_ISL_1511145	DPHL	Delaware Public Health Lab	Gregory Hovan; Rebecca Savage
EPI_ISL_2593764, EPI_ISL_2595726, EPI_ISL_2595860, EPI_ISL_2596341, EPI_ISL_2596803, EPI_ISL_2597228, EPI_ISL_2597229, EPI_ISL_2597312, EPI_ISL_2612300			
see above	DSMRC	DSMRC	Khine Zaw Oo; Ko Ko Lwin; Ko Ko Win; Kyee Myint; Nay Myo Aung; Pai Khant Kyaw; Phyo Kyaw Aung; Thet Wai Oo; Zaw Win Htun
EPI_ISL_2289557	DYOMEDEA NEOLAB	CNR Virus des Infections Respiratoires - France SUD	Antonin Bal; Bruno Lina; Bruno Simon; Gregory Destras; Gwendolyne Burfin; Hadrien Regue; Laurence Josset; Martine Valette; Quentin Semanas
EPI_ISL_2894008	DYOMEDEA-LABORATOIRE DE LA SAUVEGARDE	CNR Virus des Infections Respiratoires - France SUD	Antonin Bal; Bruno Lina; Gregory Destras; Gwendolyne Burfin; Hadrien Regue; Laurence Josset; Martine Valette; Quentin Semanas
EPI_ISL_416542, EPI_ISL_416543	Dasman Diabetes Institute	Dasman Diabetes Institute	Ebaa AlOzairi; Fahd Al-Mulla; Motasem Melhem; Qais Al-Duwairi; Rasheeba Iqbal; Sara Al-Qabandi; Sumi John
EPI_ISL_416541	Dasman Diabetes Institute and Virology Laboratory Ministry of Health	Dasman Diabetes Institute	Ebaa AlOzairi; Fahd Al-Mulla; Motasem Melhem; Qais Al-Duwairi; Rasheeba Iqbal; Sara Al-Qabandi; Sumi John
EPI_ISL_2140727	De Rust (Blommenek) Clinic wc DRC	NHLS/UCT	Arash Iranzadeh; Bruna Galvao; Carolyn Williamson; Deelan Doolabh; Diana Hardie; Innocent Mudau; Kruger Marais; Lynn Tyers; Marvin Hsiao; Stephen Korsman
EPI_ISL_2372406, EPI_ISL_2648206, EPI_ISL_2648207, EPI_ISL_2648208, EPI_ISL_2648209, EPI_ISL_2648211, EPI_ISL_2648213, EPI_ISL_2648214, EPI_ISL_2648215, EPI_ISL_2648217, EPI_ISL_2648218, EPI_ISL_2648219, EPI_ISL_2648220			
see above	Debswana Orapa Mine Hospital Laboratory	Botswana Harvard HIV Reference Laboratory	Boitumelo Zuze; Botshelo Radibe; Dorcas Maruapula; Joseph Makhema; Keoratile Ntshambiwa; Kgomotso Moruisi; Koketso Maotwe; Legodile Kooepile; Lesedi Magama; Madisa Mine; Mosepele Mosepele; Mpo Molapisi; Ontlametse T. Bareng; Pamela Smith-Lawrence; Roger Shapiro; Shahin Lockman; Sikhulile Moyo; Simani Gaseitsiwe; Thongbotho Mphoyakgosi; Wonderful T. Choga
EPI_ISL_812293	Delaware Public Health Lab	Delaware Public Health Lab	Gregory Hovan
EPI_ISL_693691, EPI_ISL_693730	Delaware Public Health Laboratory	Delaware Public Health Laboratory	Gregory Hovan
EPI_ISL_2432518, EPI_ISL_2432922, EPI_ISL_2432953	Demy Health	National Reference Laboratory, Nigeria Centre for Disease Control	Anthony Ahumibe; Catherine Okoi; Chimaobi Chukwu; Dr Chikwe Ihekweazu; Dr Ndodo Nnaemeka; Dr Omoare Adesuyi; Grace Esebanmen; Naidoo Dhamari; Nwando Mba; Olusola Anuoluwapo Akanbi
EPI_ISL_430796	Departamento de Biología y genética molecular, IACA Laboratorios.	Área de Secuenciación del Laboratorio de Virología del Hospital de Niños Dr. Ricardo Gutierrez on behalf of 'Proyecto Argentino Interinstitucional de genomica de SARS-CoV-2' (PAIS Consortium)	A; AS; E; Goya; LE; Lusso; MI; MS; Masciovecchio MV; Mistchenko; Nabaes Jodar; Natale; S; Streitenberger ER; Suárez; Tittarelli; Valinotto; Viegas, M.
EPI_ISL_949238	Departamento de Microbiología, CDB, Hospital Clínic, Barcelona	SeqCOVID-SPAIN consortium/IBV(CSIC)	Aida Peiró and SeqCOVID-SPAIN consortium; Andrea Vergara; Elisa Rubio; Jéssica Navero; Mikel Martínez
EPI_ISL_1340753, EPI_ISL_1340755, EPI_ISL_1340756, EPI_ISL_1340758	Departamento de Virologia, Laboratorio Central de Salud Pública, Avenida Venezuela y Teniente Escurra, Asunción, Paraguay	Laboratory of Respiratory Viruses and Measles, Oswaldo Cruz Institute, FIOCRUZ	Alice Sampaio Rocha; Ana Carolina Mendonca; Ana Carolina Paixao; Cynthia Vazquez; Fernando Motta; Luciana Appolinario; Marilda Siqueira on behalf of the Fiocruz COVID-19 Genomic Surveillance Network; Paola Resende; Renata Serrano Lopes
EPI_ISL_516923	Department for Molecular Diagnostics, Centre for Medical Microbiology, Institute of Public Health of Montenegro	Charité Universitätsmedizin Berlin, Institut für Virologie	Barbara Muehlemann; Christian Drosten; Julia Schneider; Jörn Beheim-Schwarzbach; Marija Govedarica and Danijela Vujošević; Talitha Veith; Terry Jones; Victor M Corman
EPI_ISL_471529, EPI_ISL_477169, EPI_ISL_754180, EPI_ISL_1914665, EPI_ISL_1914938, EPI_ISL_1921859, EPI_ISL_3204236, EPI_ISL_3204239, EPI_ISL_3204245			
see above	Department for Virology, Molecular Biology and Genome Research, R. G. Lugar Center for Public Health Research, National Center for Disease Control and Public Health (NCDC) of Georgia.	Department for Virology, Molecular Biology and Genome Research, R. G. Lugar Center for Public Health Research, National Center for Disease Control and Public Health (NCDC) of Georgia.	Adam Kotorashvili; Amiran Gamkrelidze.; Ana Papkiauri; Ann Machablishvili; Anna Kasradze; Davit Tsaguria; Ekaterine Khmaladze; Ekaterine Zangaladze; Ekaterine Zhghenti; Giorgi Gogoladze; Giorgi Tomashvili; Gvantsa Brachveli; Gvantsa Chanturia; Irma Burjanadze; Ketevan Sidamonidze; Khatuna Zakhashvili; Lela Sabadze; Lela Urushadze; Magda Dgebuadze; Maia Alkhazashvili; Mari Gavashelidze; Mariam Zakalashvili; Marine Murtskhvaladze; Meri Pantsulaia; Nato Kotaria; Nino Berishvili; Paata Imnadze; Roena Sukhiashvili; Salome Javashvili; Tamar Jashiashvili; Tata Imnadze; Tea Tevdoradze
EPI_ISL_1642768	Department fÃ¼r Labormedizin Abteilung III Bereich Molekulare Diagnostik UniversitÃ¤tsklinikum Halle(Saale)	Robert Koch Institute	
EPI_ISL_2723562, EPI_ISL_2896229	Department of Acute Infectious Diseases Control and Prevention, Yunnan Provincial Center for Disease Control and Prevention	Department of Acute Infectious Diseases Control and Prevention, Yunnan Provincial Center for Disease Control and Prevention	Jienan Zhou; Meiling Zhang; Senquan Jia; Xiaoqing Fu; Zhaosheng Liu
EPI_ISL_2429651, EPI_ISL_2665812, EPI_ISL_2666405, EPI_ISL_2667413, EPI_ISL_2669906, EPI_ISL_2670493, EPI_ISL_2670876, EPI_ISL_2671330, EPI_ISL_2680019, EPI_ISL_2684126, EPI_ISL_2819459, EPI_ISL_2854368, EPI_ISL_2854578, EPI_ISL_2862744, EPI_ISL_2971522, EPI_ISL_2992466, EPI_ISL_2992648, EPI_ISL_3029549			
see above	Department of Bacteria, Parasites and Fungi, Statens Serum Institut, Copenhagen, Denmark	Statens Serum Institut Bioinformatics and Microbial Genomics	Danish Covid-19 Genome Consortium
EPI_ISL_515082	Department of Biochemistry, Cell and Molecular Biology	WACCBIP, University of Ghana	A.K.; Adu, B.; Amenga-Etego; Ampofo, W.; Amuzu; Anang; Arjarquah, A.; Asante, I.; Awandare; Bediako, Y.; Boatemaa, L.; Bonney, E.; Bonney, K.; C.M.; D.S.; Eshun, M.; G.A.; G.B.; J.K.; J.M.; Kotey, E.; Kumordjie, S.; Kyei; L.N.; Magnussen, V.; Morang'a; Mutungi; Ngoi; Quashie, P.; Tei-Maya, F.
EPI_ISL_884839, EPI_ISL_884841, EPI_ISL_884856	Department of Biochemistry, Cell and Molecular Biology, West African Centre for Cell Biology of Infectious Pathogens (WACCBIP), University of Ghana	Department of Biochemistry, Cell and Molecular Biology, West African Centre for Cell Biology of Infectious Pathogens (WACCBIP), University of Ghana	A.-K.; A.B.; Abass; Akoriyea; Amenga-Etego; Amoako, E.; Amuzu; Awandare; Bediako, Y.; Boakye; C.M.; D.S.; Diallo; G.A.; J.M.; Kibinge, N.; Kumi-Ansah, F.; L.N.; Magnussen, V.; Mohammed, A.; Morang'a; Ngoi; O.D.; Odoom, T.; Quashie, P.; S.K.; Tapela, K.; Tei-Maya, F.
EPI_ISL_421195, EPI_ISL_540509, EPI_ISL_678471, EPI_ISL_737311, EPI_ISL_1708595, EPI_ISL_1826581, EPI_ISL_1826750			
see above	Department of Clinical Microbiology	GIGA Medical Genomics	Axelle Chaslain; Bouchra Boujemla; Cécile Meex; Céline Fombellida-Lopez; Keith Durkin; Maria Artesi; Marie-Pierre Hayette; Nathalie Renotte; Pierrette Melin; Raphaël Boreux; Sébastien Bontems; Vincent Bours; Vincent Bours.

EPI_ISL_419216	Department of Clinical Pathology, Pamela Youde Nethersole Eastern Hospital	Department of Health Technology and Informatics, Faculty of Health and Social Science, The Hong Kong Polytechnic University	Alan Ka-Lun WU; Alex Yat-Man HO; Barry Kin-Chung WONG; David Ho-Keung SHUM; Eugene Yuk-Keung TSO; Gilman Kit-Hang SIU; Hiu-Yin LAO; Kam-Tong YIP; Kenneth Siu-Sing LEUNG; Kingsley King-Gee TAM; Kit-Man SIN; Kitty Sau-Chun FUNG; Kwok-Cheung LUNG; Lam-Kwong LEE; Man-Chun CHAN; Ming-Pan CHOI; Miranda Chong-Yee YAU; Raymond Wai-To LIU; Sandy Ka-Yee CHAU; Shea Ping YIP; Tak-Lun QUE; Timothy Ting-Leung NG; Wai-Shing LEUNG; Wing Cheong YAM; Wing-Kin TO; Yuk-Yung NG
EPI_ISL_1731082	Department of Genetics and Department of Microbiology, University Hospital Motol	Department of Microbiology, University Hospital Motol	Ales Briksi; Katerina Chuda; Katerina Pehlikova; Malgorzata Libik; Milan Macek; Ondrej Cinek; Pavel Drevinek; Petr Broz; Petr Hubacek; Zdenek Kepka
EPI_ISL_1280115	Department of Genetics, Medirex	Laboratory of Genomics and Bioinformatics, Comenius University Science Park	Anna Gičová; Diana Rusňáková; Gabriel Minárik; Jaroslav Budiš; Miroslav Böhmer; Renáta Lukačková; Tatiana Sedláčková; Tomáš Szemes; Werner Krampl
EPI_ISL_1121891	Department of Health & Mental Hygiene Crown Heights	New York City Public Health Laboratory	Jade Wang; et al.
EPI_ISL_1018120, EPI_ISL_1018293, EPI_ISL_1018326, EPI_ISL_2710858, EPI_ISL_2713166, EPI_ISL_2713181, EPI_ISL_2713213, EPI_ISL_2713247, EPI_ISL_2713256, EPI_ISL_2713258, EPI_ISL_2713309, EPI_ISL_2723759, EPI_ISL_2723767, EPI_ISL_2723768, EPI_ISL_2860793, EPI_ISL_3039610			
see above	Department of Health Technology and Informatics, The Hong Kong Polytechnic University	Department of Health Technology and Informatics, The Hong Kong Polytechnic University	Alan Ka-Lun Wu; Alex Yat-Man Ho; Barry Kin-Chung Wong; Chloe Toi-Mei Chan; David Ho-Keung Shum; Denise Sze-Hang Wong; Gilman Kit-Hang Siu; Hiu-Yin Lao; Hoi-Ching Jim; Ivan Tak-Fai Wong; Jake Siu-Lun Leung; Kam-Tong Yip; Kenneth Siu-Sing Leung; Kingsley King-Gee Tam; Kitty Sau-Chun Fung; Kristine Luk; Lam-Kwong Lee; Miranda Chong-Yee Yau; Sandy Ka-Yee Chau; Shea Ping Yip; Tak-Lun Que; Timothy Ting-Leung Ng; Wing Cheong Yam; Wing-Hei Lo; Wing-Kin To; Yvette Wai-Man Lai
EPI_ISL_408430	Department of Infectious and Tropical Diseases, Bichat Claude Bernard Hospital, Paris	National Reference Center for Viruses of Respiratory Infections, Institut Pasteur, Paris	Angela Brisebarre; Flora Donati; Marion Barbet; Maud Vanpeene; Mélanie Albert; Méline Bizard; Sylvie Behillil; Sylvie van der Werf; Vincent Enouf; Xavier Lescure; Yazdan Yazdanpanah
EPI_ISL_413019	Department of Internal Medicine, Triemli Hospital	Institute of Medical Virology, University of Zurich	Alexandra Trkola; Andrea Zbinden; Fiona Steiner; Gerhard Eich; Jon Huder; Jürg Böni; Maryam Zaheri; Michael Huber; Patrick Redli; Riccarda Capaul; Stefan Schmutz; Verena Kufner
EPI_ISL_1965488, EPI_ISL_2137132	Department of Laboratory Medicine, Division of Clinical Virology, University of Medicine, Vienna	Bergthaler laboratory, CeMM Research Center for Molecular Medicine of the Austrian Academy of Sciences	Andreas Bergthaler; Anna Schedl; Bekir Erguner; Benedikt Agerer; Christoph Bock; Fabian Amman; Jan Laine; Lukas Endler; Maelle Le Moing; Martin Senekowitsch; Michael Schuster; Petr Triska; Thomas Penz
EPI_ISL_422411, EPI_ISL_422417, EPI_ISL_447614, EPI_ISL_1667474	Department of Laboratory Medicine, National Taiwan University Hospital	Microbial Genomics Core Lab, National Taiwan University Centers of Genomic and Precision Medicine	Chiao-Ling Li; Pei-Jer Chen; Shan-Chwen Chang; Shiou-Hwei Yeh; Sui-Yuan Chang; Ya-Yun Lai; You-Yu Lin
EPI_ISL_476795, EPI_ISL_538436, EPI_ISL_538458, EPI_ISL_648689, EPI_ISL_648761	Department of Laboratory Medicine, Tan Tock Seng Hospital	Department of Laboratory Medicine, Tan Tock Seng Hospital	Barkham TMS; Chen YYC; Li C; Lim JX; Maurer-Stroh S; Nagarajan N; Sessions OM; Tang WY; Zair X
EPI_ISL_1599170, EPI_ISL_1820321, EPI_ISL_2138809	Department of Medical Microbiology - section Molde, Molde Hospital	Norwegian Institute of Public Health, Department of Virology	Atiya R Ali; Debech Nadia; Engebretsen Serina Beate; Garcia Llorente Ignacio; Hilde Elshaug; Hilde Vollan; Jon Bråte; Kamilla Heddeland Instefjord; Karoline Bragstad; Kathrine Stene-Johansen; Marie Paulsen Madsen; Olav Hungnes; Pedersen Benedikte Nevjen; Rasmus Riis Kopperud
EPI_ISL_861719	Department of Medical Microbiology, Hospital Pengajar Universiti Putra Malaysia	Malaysia Genome Institute	Avisha Richards; Azrin Ahmad; Enizza Kasim; Hui-Yee Chee; Irni Suhayu Sapian; Mohd Faizal Abu Bakar; Mohd Noor Mat Isa; Muhammad MI; Narcisse Joseph; Nor Azfa Johari; Nor Zahrin Hasran; Nurhezreen Md Iqbal; Shamsidar Sopie; Siti Noraini Othman; Syafinaz Amin-Nordin; Yusuf Muhammad Noor
EPI_ISL_1192188, EPI_ISL_1820301, EPI_ISL_1919387, EPI_ISL_1989268, EPI_ISL_2142789, EPI_ISL_2225820, EPI_ISL_2225844, EPI_ISL_2225890, EPI_ISL_2225891, EPI_ISL_2225993, EPI_ISL_2333513, EPI_ISL_2333514, EPI_ISL_2549126, EPI_ISL_2612273, EPI_ISL_2927874, EPI_ISL_3152014, EPI_ISL_3152024			
see above	Department of Medical Microbiology, St. Olavs hospital	Norwegian Institute of Public Health, Department of Virology	'Kathrine Stene-Johansen; Atiya R Ali; Debech Nadia; Engebretsen Serina Beate; Garcia Llorente Ignacio; Hilde Elshaug; Hilde Vollan; Jon Bråte; Kamilla Heddeland Instefjord; Karoline Bragstad; Kathrine Stene-Johansen; Line Victoria Moen; Marie Paulsen Madsen; Olav Hungnes; Pedersen Benedikte Nevjen; Rasmus Riis Kopperud
EPI_ISL_506999	Department of Medical Microbiology, University Malaya Medical Centre	Department of Medical Microbiology, Faculty of Medicine, University of Malaya	I-Ching SAM; Jennifer Chong; University Malaya Medical Centre COVID Team; Yoke Fun CHAN; Yoong Min CHONG
EPI_ISL_512844	Department of Medical Research	DMR_Myanmar	Aung Kyaw Kyaw; Aung Zaw Latt; Hlaing Myat Thu; Hnin Ohnmar Soe; Htin Lin; Kay Thi Aye; Lai Lai San; Myat Htut Nyunt; Nan Aye Thida Oo; Ni Ni Zaw; Phyu Win Ei; Su Mon Win; Theingi Win Myat; Wah Wah Aung; Yi Yi Kyaw; Zaw Than Htun
EPI_ISL_2547429	Department of Medical Virology, School of Medicine Ahvaz Jundishapur University of Medical sciences	Genetics Research Center, University of Social Welfare and Rehabilitation Sciences	Ahmad Tavakoli; Azarakhsh Azaran; Farid yousefi; Hossein Najmabadi.; Kimia Kahrizi; Maryam Beheshtian; Marzieh Mohseni; Mohammad Farahmand; Mohsen Savaie; Seyed Mohammad Jazayeri; Zohreh Fattahi
EPI_ISL_437442	Department of MicroBiology, Government Medical College, Surat	Gujarat Biotechnology Research Centre	Akanksha Verma; Amit Kanani; Ankit Hinsu; Apurvasinh Puvar; Bhavesh Modi; Binita Aring; Chaitanya Joshi; Dinesh Kumar; Dipa Kinariwala; Dipeshwari Shewale; Disha Patel; Gaurishankar Shrimali; Geeta Vaghela; Janvi Raval; Kairavi Joshi; Kamlesh J Upadhyay; Madhvi Joshi; Maharshi Pandya; Monika Gandhi; Neeta Khandelwal; Nidhi Sood; Nitin Savaliya; Pinal Trivedi; Pranay Shah; Pritesh Sabara; R D Dixit; Raghawendra Kumar; Ramesh Pandit; Snehal Bagatharia; Sonia Barve; Tejas Shah; Zuber Saiyed
EPI_ISL_1273103	Department of Microbial Biotechnology, Genetic Engineering Division, National Research Centre	Department of Microbial Biotechnology, Genetic Engineering Division, National Research Centre	A.E. and Shemis, M.; A.G.; Dawood; Farghaly, A.; Fikry; Khalifa; M.K.; R.M.; Taha
EPI_ISL_1745697	Department of Microbiology, Faculty of Medicine and Dentistry, Palacky University and University Hospital Olomouc	Institute of Molecular and Translational Medicine / Laboratory of Experimental Medicine, Faculty of Medicine and Dentistry, Palacky University	Barbora Blumová; Hana Jaworek; Marián Hajdúch; Pavel Sauer; Rastislav Slavkovský; Tomáš Pospíšil; Vladimíra Koudeláková
EPI_ISL_2833646, EPI_ISL_2833648, EPI_ISL_2833649, EPI_ISL_2833650, EPI_ISL_2833651, EPI_ISL_2833652, EPI_ISL_2833653, EPI_ISL_2833654, EPI_ISL_2833655, EPI_ISL_2833657, EPI_ISL_2833658, EPI_ISL_2833659, EPI_ISL_2833661, EPI_ISL_2833663, EPI_ISL_2833664, EPI_ISL_2833665, EPI_ISL_2833666			
see above	Department of Microbiology, National Institute for Public Health of Kosova	Charité Universitätsmedizin Berlin, Institut für Virologie	Aferdita Hyseni; Barbara Mühlemann; Blendi Jerliu; Christian Drosten; Donjeta Hajdari; Julia Schneider; Jörn Beheim-Schwarzbach; Nazmi Mehmeti; Pranvera Abazi; Talitha Veith; Terry Jones; Victor M Corman; Xhevat Jakupi; Zana Deva
EPI_ISL_1027643, EPI_ISL_1027652, EPI_ISL_1027653	Department of Microbiology, National Institute for Public Health of Kosova	Charité Universitätsmedizin Berlin, Institut für Virologie	Barbara Mühlemann; Christian Drosten; Donjeta Hajdari; Julia Schneider; Jörn Beheim-Schwarzbach; Talitha Veith; Terry Jones; Victor M Corman; Xhevat Jakupi; Zana Deva
EPI_ISL_1034592, EPI_ISL_1034598, EPI_ISL_1034716, EPI_ISL_2423558	Department of Microbiology, The University of Hong Kong	Department of Microbiology, The University of Hong Kong	Kelvin K.W. To; Kwok-Yung Yuen
EPI_ISL_1117809, EPI_ISL_1840981	Department of Microbiology, University Innsbruck	Bergthaler laboratory, CeMM Research Center for Molecular Medicine of the Austrian Academy of Sciences	Andreas Bergthaler; Anna Schedl; Bekir Erguner; Benedikt Agerer; Christoph Bock; Fabian Amman; Jan Laine; Lukas Endler; Maelle Le Moing; Martin Senekowitsch; Michael Schuster; Petr Triska; Thomas Penz
EPI_ISL_463744, EPI_ISL_463745, EPI_ISL_1164626, EPI_ISL_1164627, EPI_ISL_1164628, EPI_ISL_1164649, EPI_ISL_1164662, EPI_ISL_1164664, EPI_ISL_1164671			
see above	Department of Molecular Virology, Cyprus Institute of Neurology and Genetics	Department of Molecular Virology, Cyprus Institute of Neurology and Genetics	Anastasis Oulas; Andreas Hadjisavvas; Christina Christodoulou; Christina Tryfonos; Dana Koptides; Denise Alexandrou; George Krashias; George Spyrou; Jan Richter; Maria Loizidou; Mihalis Panayiotidis; Olga Kalakouta; Pavlos Fanis; Stavros Bashiardes
EPI_ISL_2692906	Department of Pathology, School of Medicine, Imam Hospital Complex, Tehran University of Medical Sciences, Tehran, Iran	Genetics Research Center, University of Social Welfare and Rehabilitation Sciences	Ali Jafarpour; Alireza Abdollahi; Azam Ghaziasadi; Azar Hadadi; Hossein Najmabadi.; Khadijeh Jalalvand; Kimia Kahrizi; Marzieh Mohseni; Reza Najafipour; Saber Soltani; Seyed Mohammad Jazayeri; Seyedeh elham Mortazavi; Zohreh Fattahi
EPI_ISL_448062	Department of Pathology, University of Cambridge	COVID-19 Genomics UK (COG-UK) Consortium	Aminu S. Jahun; Anna Yakovleva; Charlotte J. Houldcroft; Fahad A Khokhar; Grant Hall; Ian Goodfellow; Laura G Caller; Luke W Meredith; M. Estée Török; Martin D. Curran; Myra Hosmillo; Sarah L. Caddy; Theresa Feltwell; William L. Hamilton
EPI_ISL_439557, EPI_ISL_439647, EPI_ISL_459182, EPI_ISL_459193, EPI_ISL_459217, EPI_ISL_459427, EPI_ISL_459489, EPI_ISL_459495			
see above	Department of Pathology, University of Cambridge	Wellcome Sanger Institute for the COVID-19 Genomics UK (COG-UK) consortium	Alex Alderton; Aminu S. Jahun; Anna Yakovleva; Charlotte J. Houldcroft; Cordelia Langford; David K. Jackson; Dominic Kwiatkowski; Ewan Harrison; Fahad A Khokhar; Grant Hall; Ian Goodfellow; Ian Johnston; John Sillitoe on behalf of the Wellcome Sanger Institute COVID-19 Surveillance Team (http://www.sanger.ac.uk/covid-team); Laura G Caller; Luke W Meredith; M. Estée Török; Martin D. Curran; Myra Hosmillo; Roberto Amato; Sarah L. Caddy; Sonia Goncalves; Theresa Feltwell; William L. Hamilton; and Alex Alderton
EPI_ISL_1657104, EPI_ISL_1657117, EPI_ISL_1969986, EPI_ISL_1969990, EPI_ISL_2099878, EPI_ISL_2099884, EPI_ISL_2190107			
see above	Department of Public Health Bucharest	National Institute of Infectious Diseases-Prof. Dr. Matei Bals Molecular Diagnostics Laboratory	Andreea Tudor; Corina Casangiu; Dan Otelea; Leontina Banica; Marius Surleac; Ovidiu Vlaicu; Simona Paraschiv
EPI_ISL_2687986	Department of Public Health Buzau	National Institute of Infectious Diseases-Prof. Dr. Matei Bals Molecular Diagnostics Laboratory	Corina Casangiu; Dan Otelea; Leontina Banica; Marius Surleac; Petre Milu; Robert Hohan; Simona Paraschiv
EPI_ISL_2687992, EPI_ISL_3045846	Department of Public Health Constanta	National Institute of Infectious Diseases-Prof. Dr. Matei Bals Molecular Diagnostics Laboratory	Corina Casangiu; Dan Otelea; Leontina Banica; Marius Surleac; Petre Milu; Robert Hohan; Simona Paraschiv
EPI_ISL_3045850, EPI_ISL_3045852, EPI_ISL_3045853, EPI_ISL_3045867, EPI_ISL_3045876	Department of Public Health Iasi	National Institute of Infectious Diseases-Prof. Dr. Matei Bals Molecular Diagnostics Laboratory	Corina Casangiu; Dan Otelea; Leontina Banica; Marius Surleac; Petre Milu; Robert Hohan; Simona Paraschiv
EPI_ISL_1677895, EPI_ISL_1795690, EPI_ISL_1795878	Department of Public Health Microbiology Ljubljana, National Laboratory for Health, Environment and Food	Department for Public Health Microbiology Ljubljana, National Laboratory for Health, Environment and Food	José Gonçalves; Katarina Prosenc; Martin Bosilj; Metka Paragi; Natasa Berginc; Tom Koritnik
EPI_ISL_582513, EPI_ISL_979973	Department of Respiratory and other Viral Infections of L.V.Gromashevsky Institute of Epidemiology & Infectious Diseases NAMS of Ukraine	Department of Respiratory and other Viral Infections of L.V.Gromashevsky Institute of Epidemiology & Infectious Diseases NAMS of Ukraine, JSC "Farmak"	Alla Mironenko; Andriy Goy; Ihor Kravchuk; Larysa Radchenko; Ludmyla Bolotova; Nataliia Teteriuk
EPI_ISL_1385784, EPI_ISL_1385786, EPI_ISL_1385791, EPI_ISL_1385794, EPI_ISL_1398039, EPI_ISL_1406394, EPI_ISL_1710506, EPI_ISL_1711673, EPI_ISL_1732172, EPI_ISL_1969995, EPI_ISL_2313112, EPI_ISL_2434781, EPI_ISL_2434982, EPI_ISL_2438547, EPI_ISL_2757735, EPI_ISL_2757736, EPI_ISL_2757738, EPI_ISL_2757739, EPI_ISL_2757740, EPI_ISL_2757743, EPI_ISL_2757756, EPI_ISL_2757758, EPI_ISL_2757759, EPI_ISL_2757761, EPI_ISL_2757762, EPI_ISL_2894974, EPI_ISL_2894982			
see above	Department of Virology	Department of Virology	Aamer Ikram; Abdul Ahad; Adnan Haider; Ammar Amjad; Austin Leach; Joel Montgomery; John Klena; Ketan Patel; Massab Umair; Melissa Mobley; Muhammad Ammar; Muhammad Salman; Nazish Badar; Qasim Ali; Sana Tamim; Shannon Whitmer; Syed Adnan Haider; Zaira Rehman
EPI_ISL_732649, EPI_ISL_738136, EPI_ISL_759798, EPI_ISL_759830, EPI_ISL_1218648, EPI_ISL_1595768, EPI_ISL_2604473, EPI_ISL_2608455, EPI_ISL_2608457, EPI_ISL_2608770, EPI_ISL_2608977, EPI_ISL_2609443, EPI_ISL_3014476, EPI_ISL_3014729, EPI_ISL_3014888, EPI_ISL_3015049			
see above	Department of Virology and Immunology, University of	Department of Virology, Faculty of Medicine, University of	Essi Korhonen; Fathiah Zakham; Hanna Jarva; Hanna Liimatainen; Hannimari Kallio-Kokko; Harri Kangas; Hussein Alburkat; Jenni Virtanen; Maija Lappalainen; Maija Suvanto; Mert Erdin; Olli Vapalahti; Pekka Ellonen; Phuoc Truong; Ravi Kant; Sari Hannula; Satu

	Helsinki and Helsinki University Hospital, Huslab Finland	Helsinki, Helsinki, Finland	Kurkela; Teemu Smura; fathiah Zakham
EPI_ISL_2896259	Department of Virology, Molecular Biology and Genome Research	Department of Virology, Molecular Biology and Genome Research	Kotaria, N.; Kotorashvili, A.; Murtskhvaladze, M.
EPI_ISL_855567, EPI_ISL_855570	Department of Virology, Principal Military Hospital of Instruction of Tunis	Bundeswehr Institute of Microbiology	Habiba Naija; Kilian Stoecker; Malena Bestehorn-Willmann; Markus H. Antwerpen; Mathias C. Walter; Roman Wölfel & Mohamed Ben Moussa; Simone Eckstein; Susann Handrick
EPI_ISL_757286	Department of Virology, Public Health Laboratories Division	Department of Virology, Public Health Laboratories Division	Aamer Ikram; Massab Umair; Muhammad Salman
EPI_ISL_2254131	Department of Virology, School of Medicine, Shiraz University of Medical Sciences, Shiraz, Iran	Genetics Research Center, University of Social Welfare and Rehabilitation Sciences	Hossein Najmabadi; Kimia Kahrizi; Marzieh Mohseni; Moattari A; Moghadami M; Zohreh Fattahi
EPI_ISL_926067, EPI_ISL_927136, EPI_ISL_928108, EPI_ISL_928152, EPI_ISL_930199, EPI_ISL_1022501, EPI_ISL_1022644, EPI_ISL_1022682, EPI_ISL_1022684, EPI_ISL_1065948, EPI_ISL_1125881, EPI_ISL_1126064, EPI_ISL_1126279, EPI_ISL_1858552, EPI_ISL_1859527, EPI_ISL_1859734, EPI_ISL_1860060, EPI_ISL_1860341, EPI_ISL_1862355, EPI_ISL_1862402, EPI_ISL_1862888, EPI_ISL_1863717, EPI_ISL_1864758, EPI_ISL_1865226, EPI_ISL_1865320, EPI_ISL_1866574, EPI_ISL_1867960, EPI_ISL_1868550, EPI_ISL_1868837, EPI_ISL_1869258, EPI_ISL_1871039, EPI_ISL_1871224, EPI_ISL_1871274, EPI_ISL_1871949, EPI_ISL_1872826, EPI_ISL_1872870, EPI_ISL_1873858, EPI_ISL_1874487, EPI_ISL_1874779, EPI_ISL_1875598, EPI_ISL_1875866, EPI_ISL_1876066, EPI_ISL_1876442, EPI_ISL_1876721, EPI_ISL_1877285, EPI_ISL_1877995, EPI_ISL_1878462, EPI_ISL_1879001, EPI_ISL_1881008, EPI_ISL_1881989, EPI_ISL_1882010, EPI_ISL_1882472, EPI_ISL_1882982, EPI_ISL_1883563, EPI_ISL_1883815, EPI_ISL_1884113, EPI_ISL_1886538, EPI_ISL_1887618, EPI_ISL_1888054, EPI_ISL_1888124, EPI_ISL_1890115, EPI_ISL_1891575, EPI_ISL_1891764, EPI_ISL_1893809, EPI_ISL_1894309, EPI_ISL_1895070, EPI_ISL_1896329, EPI_ISL_2023813, EPI_ISL_2025423, EPI_ISL_2028832, EPI_ISL_2175777, EPI_ISL_2176487, EPI_ISL_2300915			
see above	Department of Virus and Microbiological Special Diagnostics, Statens Serum Institut, Copenhagen, Denmark	Aalborg University	Danish Covid-19 Genome Consortium
EPI_ISL_669126, EPI_ISL_669629, EPI_ISL_670863, EPI_ISL_757623, EPI_ISL_792954	Department of Virus and Microbiological Special Diagnostics, Statens Serum Institut, Copenhagen, Denmark	Albertsen Lab, Department of Chemistry and Bioscience, Aalborg University, Denmark	Danish Covid-19 Genome Consortium
EPI_ISL_437631	Department of Virus and Microbiological Special Diagnostics, Statens Serum Institut, Copenhagen, Denmark, Artillerivej 5, 2300 Copenahgen S	Albertsen lab, Department of Chemistry and Bioscience, Aalborg University, Denmark	Rasmus Kirkegaard
EPI_ISL_614409, EPI_ISL_616443, EPI_ISL_616724, EPI_ISL_617431, EPI_ISL_618152, EPI_ISL_618309, EPI_ISL_618562, EPI_ISL_619869, EPI_ISL_620017, EPI_ISL_621663			
see above	Department of Virus and Microbiological Special Diagnostics, Statens Serum Institut, Denmark	Albertsen lab, Department of Chemistry and Bioscience, Aalborg University, Denmark	Danish Covid-19 Genome Consortia
EPI_ISL_1919409, EPI_ISL_2400054, EPI_ISL_2491413	Dept. of Medical Microbiology, Stavanger University Hospital, Helse Stavanger HF	Norwegian Institute of Public Health, Department of Virology	Atiya R Ali; Debech Nadia; Engebretsen Serina Beate; Garcia Llorente Ignacio; Hilde Elshaug; Hilde Vollan; Jon Bråte; Kamilla Heddeland Instefjord; Karoline Bragstad; Kathrine Stene-Johansen; Line Victoria Moen; Marie Paulsen Madsen; Olav Hungnes; Pedersen Benedikte Nevjen; Rasmus Riis Kopperud
EPI_ISL_1118120, EPI_ISL_1118121	Dept. of Microbiology and Infection Control, Akershus University Hospital HF	Dept. of Microbiology and Infection Control, Akershus University Hospital HF	Alexander Hesselberg Løvestad; Hege Vangstein Aamot
EPI_ISL_410532	Dept. of Pathology, National Institute of Infectious Diseases	Pathogen Genomics Center, National Institute of Infectious Diseases	Harutaka Katano; Hideki Hasegawa; Kazuya Shirato; Makoto Kuroda; Makoto Takeda; Motoi Suzuki; Naganori Nao; Shutoku Matsuyama; Tadaki Suzuki; Takaji Wakita; Tsuyoshi Sekizuka
EPI_ISL_774872	Designated Reference Institute for Chemical Measurements (DRICM)	DNA SOLUTION LTD.	Abdul Khaleque; Abu Sufian; Hasan Ul Haider; Jannatun Naima; Kazi Nadim Hasan; MSM Chowdhury; Mala Khan; Mamudul Hasan Razu; Md. Imran Khan; Mizanur Rahman; Mohammad Fazle Alam Rabbi
EPI_ISL_2171041, EPI_ISL_2171267	Dhulikhel Hospital, Kathmandu University Hospital	Molecular and Genomics Research Lab, Dhulikhel Hospital, Kathmandu University Hospital	Dipesh Tamrakar; Manu Vanaerschot; Meghnath Dhimal; Navin Adhikari; Nishan Katuwal; Pradip Gyanwali; Rajeev Shrestha; Saroj Bhattarai; Surendra Kumar Madhup
EPI_ISL_2101016, EPI_ISL_2101017	Diagnostic Laboratory-Microbiology	Biomedical Laboratory-2	; A. Dharshan De Silva; Dilini Nakawita; Dilini Nakkawita; Dumitha Govindapala; Harshi Abeygoonawardena; Himali S. Jayasinghearachchi; Pawani Gamage; Priyamali Jayasekera; Thamarasi Senaratne; Upeksha Kulasekera
EPI_ISL_1447336, EPI_ISL_1533051, EPI_ISL_1533073	Diagnostic and Research Center of Infectious Diseases, Medical Faculty, Andalas University	Diagnostic and Research Center of Infectious Diseases, Medical Faculty, Andalas University	Andani Eka Putra; Ayu Novita Trisnawati; Dede Rahman Agustian; Desmawati; Dessy Arisanty; Fauzul Azhim; Gestina Aliska; Ikhwan R. Sudji; Juane Plantika Menra; Linosefa; Mutia Lailani; Nia Ayuni Putri; Nita Afriani; SM Rezvi; Sekar Asri Tresnaningtyas; Siskalil Fahma; Syafrizayanti; Syandrez Prima Putra; Yolani Syaputri
EPI_ISL_2636693, EPI_ISL_2762335, EPI_ISL_2762339, EPI_ISL_2957236, EPI_ISL_2957275	Diagnosticum - Labor Neukirchen	Robert Koch Institute	
EPI_ISL_2657185	Diagnostyka	1. Tricity SARS-CoV-2 sequencing consortium: University of Gdansk, Medical University of Gdansk, Vaxican Ltd., Invicta Ltd. 2. National Institute of Public Health - National Institute of Hygiene, Warsaw, Poland	Celina Cybulska; Karolina Gackowska; Katarzyna Groth; Katarzyna Zacharczuk; Krystyna Bienkowska Szewczyk; Lukasz Rabalski; Maciej Grzybek; Maciej Kosinski; Magdalena Nowakowska; Marcin Lubocki; Małgorzata Sadkowska-Todys; Tomasz Wołkowicz
EPI_ISL_2319055, EPI_ISL_2937950, EPI_ISL_2937967, EPI_ISL_3133633	Diagnostyka. Laboratoria Medyczne.	1. ViroGenetics - BSL3 Laboratory of Virology, Małopolska Centre of Biotechnology, Jagiellonian University; 2. genXone SA, Research & Development Laboratory	Aleksandra Gidlewicz; Anna Brylak; Brylak A; Drweska-Matelska N; Gidlewicz A; Grabowski J; Gromowski, T.; Grzegorz Nowicki; Jakub Grabowski; Januszczak S; Karol Szeszko; Kaszuba M; Kowalski, M.; Krych L; Labaj; Maciej Sykulski; Mazur-Panasiuk, N.; Michał Kaszuba; Natalia Drweska-Matelska; Nowicki G; P.P.; Pyrc, K.; Sykulski M; Sylwia Januszczak; Szeszko K; Szulc, P.; Wydmanski W.; Wydmanski, W.; Łukasz Krych
EPI_ISL_1752833	Diagnostyka. Laboratoria Medyczne.	1. ViroGenetics - BSL3 Laboratory of Virology, Małopolska Centre of Biotechnology, Jagiellonian University; 2. genXone SA, Research & Development Laboratory	Brylak A; Drweska-Matelska N; Gidlewicz A; Grabowski J; Gromowski, T.; Januszczak S; Kaszuba M; Kowalski, M.; Krych L; Labaj; Mazur-Panasiuk, N.; Nowicki G; P.P.; Pyrc, K.; Sykulski M; Szeszko K; Szulc, P.; Wydmanski W.
EPI_ISL_2597974	Dianalabs SA	Genesupport	Geraldine Jost; Katia Jaton; Nadia Liassine; Tanguy ARAUD
EPI_ISL_1855939	Dinas Kesehatan Kota Bogor	Indonesian Research Center for Veterinary Science / BBalitvet	Diana Nurjanah; Fadjry Djufry; Harimurti Nuradji; Ilham Chaidir; Ni Luh Putu Indi Dharmayanti; Risa Indriani; Sri Nowo Retno
EPI_ISL_747337, EPI_ISL_747419, EPI_ISL_760064, EPI_ISL_1063655, EPI_ISL_1063656, EPI_ISL_2161069, EPI_ISL_2161113, EPI_ISL_2161137, EPI_ISL_2332342, EPI_ISL_2361092, EPI_ISL_2361095, EPI_ISL_2361097, EPI_ISL_2361167, EPI_ISL_2464346, EPI_ISL_2464347, EPI_ISL_2710485, EPI_ISL_2710499, EPI_ISL_2754407, EPI_ISL_2754473, EPI_ISL_2754619, EPI_ISL_2967308, EPI_ISL_2967332, EPI_ISL_2967375, EPI_ISL_2967572, EPI_ISL_3026379, EPI_ISL_3026450, EPI_ISL_3026451, EPI_ISL_3026459, EPI_ISL_3026473, EPI_ISL_3026476, EPI_ISL_3026508, EPI_ISL_3026560, EPI_ISL_3026673, EPI_ISL_3026704, EPI_ISL_3026742, EPI_ISL_3026834			
see above	Division of Emerging Infectious Diseases, Bureau of Infectious Diseases Diagnosis Control, Korea Disease Control and Prevention Agency	Division of Emerging Infectious Diseases, Bureau of Infectious Diseases Diagnosis Control, Korea Disease Control and Prevention Agency	Ae Kyung Park; Chae Young Lee; Chaeyoung Lee; Eun-Jin Kim; Heui Man Kim; Il-Hwan Kim; Jeong-Ah Kim; Jeong-Min Kim; Jin Sun No; Namjoo Lee; Sang Hee Woo
EPI_ISL_2695860, EPI_ISL_2695862, EPI_ISL_2695874, EPI_ISL_2876313, EPI_ISL_2876320, EPI_ISL_2876336, EPI_ISL_2876382, EPI_ISL_3032493, EPI_ISL_3032496, EPI_ISL_3032506, EPI_ISL_3032513, EPI_ISL_3032515, EPI_ISL_3032516			
see above	Division of Medical Virology, National Health Laboratory Service (NHLS), Tygerberg Hospital / Stellenbosch University	Division of Medical Virology, National Health Laboratory Service (NHLS), Tygerberg Hospital / Stellenbosch University	Bronwyn Kleinhans; Gert van Zyl; Kamela Mahlakwane; San Emmanual James; Susan Engelbrecht; Tania Stander; Tongai Maponga; Tulio de Oliveira; Wolfgang Preiser
EPI_ISL_1591415, EPI_ISL_1591427	Division of Medical Virology, National Health Laboratory Service (NHLS), Tygerberg Hospital / Stellenbosch University	Division of Medical Virology, Stellenbosch University and NHLS Tygerberg Hospital	Bronwyn Kleinhans; Gert van Zyl; Susan Engelbrecht; Wolfgang Preiser
EPI_ISL_412869, EPI_ISL_426173, EPI_ISL_497962, EPI_ISL_506987, EPI_ISL_510627	Division of Viral Diseases, Center for Laboratory Control of Infectious Diseases, Korea Centers for Diseases Control and Prevention	Division of Viral Diseases, Center for Laboratory Control of Infectious Diseases, Korea Centers for Diseases Control and Prevention	Daesang Lee; Dong Hyun Song; Heui Man Kim; Hye-Jun Jo; Jeong-Min Kim; Jun-Sub Kim; Junhyeong Jang; Mi-Seon Kim; Myung Guk Han; Namjoo Lee; Sang Hee Woo; Sehee Park; Seong Tae Jeong; Yoon-Seok Chung
EPI_ISL_2521795	Dr S raju, Director of public heath and preventive medicine	inStem NCBS - INSACOG	Uma Ramakrishnan Dasaradhi Palakodeti Aswin SaiNarain
EPI_ISL_1055411	Dr. Leonard A. Miller Centre for Health Services	National Microbiology Laboratory (NML)	Adel Malek; Anna Majer; Anneliese Landgraff; CanCOGeN's metadata curation team; Darian Hole; Elsie Grudeski; Gary Van Domselaar; George Zahariadis; Grace Seo; Jennifer Tanner; Kerri Smith; Kirsten Biggar; Laura Gilbert; Madison Chapel; Morag Graham; Natalie Knox; Nathalie Bastien; Philip Mabon; Public Health Agency of Canada CanCOGeN team; Rhiannon Huzarewich; Robert Needle; Russell Mandes; Shari Tyson; Timothy Booth; Yan Li; Yang Yu
EPI_ISL_461385, EPI_ISL_632354, EPI_ISL_801407, EPI_ISL_2483471, EPI_ISL_2860624	Dutch COVID-19 response team	Erasmus Medical Center	Anne van der Linden; Annemiek van der Eijk; Aura Timen; Bas Oude Munnink; Claudia Schapendonk; Corien Swaan; Corine GeurtsvanKessel; David Nieuwenhuijse; Emmanuelle Munger; Irina Chestakova; Jeroen van Kampen; Jolanda Voermans; Madelief Mollers; Manon Haverkate; Marion Koopmans; Marjan Boter; Mark Pronk; Mart Stein; Pascal Lexmond; Reina Sikkema; Richard Molenkamp; Sandra Kengne Kamga Mobou; Stefan van Nieuwkoop; Theo Bestebroer; on behalf of the Dutch national COVID-19 respo; on behalf of the Dutch national COVID-19 response team.
EPI_ISL_547447, EPI_ISL_547449, EPI_ISL_547451, EPI_ISL_636492, EPI_ISL_636519, EPI_ISL_824273, EPI_ISL_1014558, EPI_ISL_1014573, EPI_ISL_1014646, EPI_ISL_1035245, EPI_ISL_1232248, EPI_ISL_1232305, EPI_ISL_1232602, EPI_ISL_1288137, EPI_ISL_1371227, EPI_ISL_1457590, EPI_ISL_1521321, EPI_ISL_1521326, EPI_ISL_1522167, EPI_ISL_1522202, EPI_ISL_1522223, EPI_ISL_1596268, EPI_ISL_1597320, EPI_ISL_1597411, EPI_ISL_1703535, EPI_ISL_1704912, EPI_ISL_1704914, EPI_ISL_1704921, EPI_ISL_1704945, EPI_ISL_1704948, EPI_ISL_1704954, EPI_ISL_1704959, EPI_ISL_1704990, EPI_ISL_1705155, EPI_ISL_1705178, EPI_ISL_1705190, EPI_ISL_1705242, EPI_ISL_1705251, EPI_ISL_1705268, EPI_ISL_1705285, EPI_ISL_1705293, EPI_ISL_1705301, EPI_ISL_1705568, EPI_ISL_1705864, EPI_ISL_1705904, EPI_ISL_1705929, EPI_ISL_1792360, EPI_ISL_1792363, EPI_ISL_1792862, EPI_ISL_1792863, EPI_ISL_1792864, EPI_ISL_1792865, EPI_ISL_1792868, EPI_ISL_1961869, EPI_ISL_1961897, EPI_ISL_1961912, EPI_ISL_1961920, EPI_ISL_1962066, EPI_ISL_1962160, EPI_ISL_1962166, EPI_ISL_1962181, EPI_ISL_1962712, EPI_ISL_1962940, EPI_ISL_1962944, EPI_ISL_1962971, EPI_ISL_2093518, EPI_ISL_2093534, EPI_ISL_2093545, EPI_ISL_2093608, EPI_ISL_2093659, EPI_ISL_2093759, EPI_ISL_2093798, EPI_ISL_2093885, EPI_ISL_2094043, EPI_ISL_2094340, EPI_ISL_2094396, EPI_ISL_2218611, EPI_ISL_2218731, EPI_ISL_2219699, EPI_ISL_2220430, EPI_ISL_2220456, EPI_ISL_2220523, EPI_ISL_2220549, EPI_ISL_2302309, EPI_ISL_2302360, EPI_ISL_2302965, EPI_ISL_2302971, EPI_ISL_2303552, EPI_ISL_2303931, EPI_ISL_2303978, EPI_ISL_2405457, EPI_ISL_2405458, EPI_ISL_2405505, EPI_ISL_2405516, EPI_ISL_2405518, EPI_ISL_2405526, EPI_ISL_2405538, EPI_ISL_2405550, EPI_ISL_2405561, EPI_ISL_2405570, EPI_ISL_2405573, EPI_ISL_2405584, EPI_ISL_2405585, EPI_ISL_2405594, EPI_ISL_2405596, EPI_ISL_2405597, EPI_ISL_2405608, EPI_ISL_2405609, EPI_ISL_2405620, EPI_ISL_2406008, EPI_ISL_2406319, EPI_ISL_2406352, EPI_ISL_2474249, EPI_ISL_2475174, EPI_ISL_2475191, EPI_ISL_2475363, EPI_ISL_2475518, EPI_ISL_2475682, EPI_ISL_2475716, EPI_ISL_2475722, EPI_ISL_2475732, EPI_ISL_2476242, EPI_ISL_2476276, EPI_ISL_2476311, EPI_ISL_2476312, EPI_ISL_2476313, EPI_ISL_2609665, EPI_ISL_2610116, EPI_ISL_2610186, EPI_ISL_2610234, EPI_ISL_2610471, EPI_ISL_2610546, EPI_ISL_2610658, EPI_ISL_2610674, EPI_ISL_2610690, EPI_ISL_2610721, EPI_ISL_2610722, EPI_ISL_2610775, EPI_ISL_2672255, EPI_ISL_2672409, EPI_ISL_2672546, EPI_ISL_2673234, EPI_ISL_2673239, EPI_ISL_2673240, EPI_ISL_2673274, EPI_ISL_2673295, EPI_ISL_2787916, EPI_ISL_2787926, EPI_ISL_2788186, EPI_ISL_2788368, EPI_ISL_2862759, EPI_ISL_2862935, EPI_ISL_2862937, EPI_ISL_2862941, EPI_ISL_2981152, EPI_ISL_2981385, EPI_ISL_2981482, EPI_ISL_2981711, EPI_ISL_2981868, EPI_ISL_2981896, EPI_ISL_2981901			
see above	Dutch COVID-19 response team	National Institute for Public Health and the Environment (RIVM)	Adam Meijer; AnneMarie van den Brandt; Annelies Kroneman; Bas van der Veer; Chantal Reusken; Dennis Schmitz; Dirk Eggink; Euníce Then; Florian Zwagemaker; Harry Vennema; James Groot; Jeroen Cremer; Jolienke Hardeman; Karim Hajji; Kim Freriks; Linda van de Nes; Lisa Wijsman; Lynn Aarts; Melissa van Tuil; Robert Kohl; Ryanne Jaarsma; Sanne Bos; Sharon van den Brink; Sjoerd Kuiling; on behalf of the national COVID-19 response team
EPI_ISL_2140728	Dysselsdorp Clinic wc DDC	NHLS/UCT	Arash Iranzadeh; Bruna Galvao; Carolyn Williamson; Deelan Doolabh; Diana Hardie; Innocent Mudau; Kruger Marais; Lynn Tyers; Marvin Hsiao; Stephen Korsman
EPI_ISL_2246957	Dział Diagnostyki Laboratoryjnej Wojewódzki Szpital	1. Academic Center for Pathomorphological and Genetic-	Anetta Sulewska; Jacek Nikliński; Janusz Dzięcioł; Joanna Kiśluk; Katarzyna Zacharczuk; Konrad Raczkowski; Magdalena Nowakowska; Małgorzata Sadkowska-Todys; Piotr Karabowicz; Piotr Majewski; Przemysław Biecek. Joanna Reszeć; Radosław Charkiewicz;

	Specjalistyczny im.J.Gromkowskiego	Molecular Diagnostics ltd, Bialystok, Poland 2. National Institute of Public Health - National Institute of Hygiene, Warsaw, Poland	Tomasz Wołkowicz
EPI_ISL_421656	E. Gulbja Laboratorija	Latvian Biomedical Research and Study Centre	Dmitrijs Perminovs; Ivars Silamiķelis; Jānis Kloviņš; Kaspars Megnis; Mikus Gavars; Monta Ustinova; Uga Dumpis; Vita Rovīte; Ņikita Zrelovs
EPI_ISL_1312417, EPI_ISL_1312435, EPI_ISL_1312683, EPI_ISL_1312689, EPI_ISL_1321930	E. Gulbja laboratorija	Latvian Biomedical Research and Study Centre	Davids Fridmanis; Dmitrijs Perminovs; Elina Dimina; Guntars Zarins; Ivars Silamikelis; Janis Klovins; Janis Pjalkovskis; Jurijs Perevoscikovs; Kaspars Megnis; Laila Silamikele; Lauma Freimane; Laura Ansone; Liga Birzniece; Mikus Gavars; Monta Ustinova; Nikita Zrelovs; Uga Dumpis; Una Krumina; Vita Rovite
EPI_ISL_1582980	E.S.E. HOSPITAL SAN JOSE DE MAICAO	Instituto Nacional de Salud- Dirección de Investigación en Salud Pública	Carlos Franco-Muñoz; Carmen Osorio; Diana Malo; Diego A. Álvarez-Díaz; Diego Andrés Prada; Gerardo Santamaría; Hector Alejandro Ruiz-Moreno; Jhonnatan Reales-González; Juan Camilo Martinez; Julian Naizaque; Katherine Laiton-Donato; Lisseth Pardo; Magdalena Wiesner; Marcela Mercado-Reyes; Maria T. Herrera-Sepúlveda; Marta Lopez Blanco; Martha Lucia Ospina Martinez; Paola Rojas; Sergio Gomez; Sheryll Corchuelo; Ángela Alarcon Cruz
EPI_ISL_1915793, EPI_ISL_1972358	EHA Clinics	National Reference Laboratory, Nigeria Centre for Disease Control	Anthony Ahumibe; Catherine Okoi; Chimaobi Chukwu; Dr Chikwe Ihekweazu; Dr Ndodo Nnaemeka; Dr Omoare Adesuyi; Grace Esebanmen; Naidoo Dhamari; Nwando Mba; Olusola Anuoluwapo Akanbi
EPI_ISL_2987317, EPI_ISL_2987318, EPI_ISL_2987335	ERMELO LABORATORY	National Institute for Communicable Diseases of the National Health Laboratory Service	Amoako DG; Bhiman JN; Everatt J; Ismail A; Mahlangu B; Mnguni A; Mohale T; Ntuli N; Scheepers C
EPI_ISL_2370457, EPI_ISL_2370853	ETHNIKO KENTRO AIMODOSIAS E.KE.A.	Greek Genome Center, Biomedical Research Foundation of the Academy of Athens (BRFAA)	Dimitrios Thanos; Efthimia Petinaki; Emmanouil Athanasiadis; Giannis Vatsellas; Katerina Zoi; Kostas Stamoulis; Theodoros Loupis
EPI_ISL_1811773, EPI_ISL_1811814	EXCITE Lab	Andersen lab at Scripps Research	Alexandre Bolze; Alice Summerfield; Celena Andrade; Charlotte Rivera-Garcia; David Becker; Efren Sandoval; Elizabeth Cirulli; Francisco Tanudjaja; Geraint Levan; James Lu + SEARCH; Jason Nguyen; Jimmy Ramirez; Kelly Schiabor Barrett; Magnus Isaksson; Marc Laurent; Nicole L Washington; Ryan Cho; Sherry Wang; Simon White; Tyler Cassens; William Lee
EPI_ISL_593513	Eastern Ontario Regional Laboratory Association	McMaster University	Ahmed Draia; Andrew G. McArthur; Emily Panousis; Hooman Derakhshani; Jalees Nasir; Leanne Mortimer; Robert Slinger
EPI_ISL_2477989, EPI_ISL_2478875, EPI_ISL_2479322, EPI_ISL_2939133, EPI_ISL_2940521, EPI_ISL_2940651, EPI_ISL_2940877, EPI_ISL_2940897			
see above	Edmonton Provincial Lab	Public Health Agency of Canada (PHAC) National Microbiology Laboratory	Buss; Croxen M; Deo A; Dieu P; E; Ferrato C; Gill K; Khan F; Koleva P; Li V; Lloyd C; Lynch T; Ma R; Murphy S; Pabbaraju K; Shokoples S; Thayer J; Tipples G; Whitehouse M; Wong A; Yu C; Zelyas N
EPI_ISL_468044, EPI_ISL_468045, EPI_ISL_468046, EPI_ISL_468047, EPI_ISL_468048, EPI_ISL_468049, EPI_ISL_468050, EPI_ISL_468051, EPI_ISL_468052, EPI_ISL_468053, EPI_ISL_468054, EPI_ISL_468055, EPI_ISL_468056, EPI_ISL_468057, EPI_ISL_468058, EPI_ISL_468059, EPI_ISL_468060, EPI_ISL_468061, EPI_ISL_468062, EPI_ISL_475722, EPI_ISL_475723, EPI_ISL_475724, EPI_ISL_477161, EPI_ISL_478672, EPI_ISL_479686, EPI_ISL_479687, EPI_ISL_479688, EPI_ISL_479689, EPI_ISL_479690, EPI_ISL_479691, EPI_ISL_479692, EPI_ISL_479693, EPI_ISL_479694, EPI_ISL_479695, EPI_ISL_479696, EPI_ISL_479697, EPI_ISL_479698, EPI_ISL_479699, EPI_ISL_479700, EPI_ISL_479701, EPI_ISL_479702, EPI_ISL_479703, EPI_ISL_479704, EPI_ISL_479705, EPI_ISL_479706, EPI_ISL_479707, EPI_ISL_479708, EPI_ISL_479709, EPI_ISL_479710, EPI_ISL_479711, EPI_ISL_479712, EPI_ISL_479713, EPI_ISL_479714, EPI_ISL_479715, EPI_ISL_479716, EPI_ISL_479717, EPI_ISL_479718, EPI_ISL_479719, EPI_ISL_479720, EPI_ISL_479721, EPI_ISL_479722, EPI_ISL_479723, EPI_ISL_479724, EPI_ISL_479725, EPI_ISL_479726, EPI_ISL_479727, EPI_ISL_479728, EPI_ISL_479729, EPI_ISL_479730, EPI_ISL_479731, EPI_ISL_479732, EPI_ISL_479733, EPI_ISL_479734, EPI_ISL_479735, EPI_ISL_524426, EPI_ISL_524427, EPI_ISL_529141, EPI_ISL_529142, EPI_ISL_529143, EPI_ISL_529144, EPI_ISL_529145			
see above	Egyptian National Cancer Institute (ENCI)	Egyptian National Cancer Institute (ENCI)	A.A.; A.N.; Abdel Rahman N; Abdel Rahman N.; Abdelhamid, W.; Abouelhoda; Abouelhoda, M.; Ahmed; Ali; Ali, M.; Amer; Bahnassy; Elkhateeb; Elsissy; Ezzelarab; Gad, A.; H.K.; Hafez; Hamdy; Hassan; Hassan, W.; K.E.; Khattab; M.A.; M.H.; M.M.; M.S.; Mahmoud; Mohamed; O.S.; Raouf, A.; S.M.; Samir, M.; Soliman; W.A.; Zekri
EPI_ISL_469275	Egyptian National Cancer Institute (ENCI)	Human Genome Center	A.A.; Abdel Rahman N; Abdelhamid, W.; Abouelhoda; Ahmed; Ali, M.; Amer; Bahnassy; Elkhateeb; Elsissy; Ezzelarab; Gad, A.; H.K.; Hafez; Hamdy; Hassan, W.; K.E.; M.H.; M.M.; M.S.; Mohamed; O.S.; Raouf, A.; S.M.; Samir, M.; Soliman; Zekri
EPI_ISL_2029260	Ekstralab Tuzla	Alea Genetic Centre	Adis Kandic; Dino Pecar; Enis Kandic; Lana Salihefendic; Nusret Butkovic; Rijad Konjhodzic
EPI_ISL_1969960, EPI_ISL_1969963	Emergency County Hospital Targu Mures	National Institute of Infectious Diseases-Prof. Dr. Matei Bals Molecular Diagnostics Laboratory	Andreea Tudor; Corina Casangiu; Dan Otelea; Leontina Banica; Marius Surleac; Ovidiu Vlaicu; Simona Paraschiv
EPI_ISL_754751	Emory Molecular Diagnostics Laboratory, Emory Healthcare	Piantadosi Lab, Emory Department of Pathology	Ahmed Babiker; Anne Piantadosi
EPI_ISL_2321508	Emory University	Centers for Disease Control and Prevention Division of Viral Diseases, Pathogen Discovery	Alison Laufer Halpin; Ben L. Rambo-Martin; Clinton R. Paden; Dakota Howard; Darlene Wagner; Dave Wentworth; Dhwani Batra; Jasmine Padilla; Justin Lee; Katie Dillon; Krista Queen; Kristen Knipe; Kristine Lacek; Mark Burroughs; Matthew Schmerer; Mili Sheth; Peter Cook; Sam Shepard; Sarah Nobles; Shoshona Le; Suxiang Tong; Vivien Dugan; Yvette Unoarumhi
EPI_ISL_2438624	Enbekshikazakh multidisciplinary interdistrict hospital	Reference laboratory for the control of viral infections	Aidar Usserbayev; Aknur Mutaliyeva; Andrey Komissarov; Artem Fadeev; Azamat Kenessov; Bekzhan Maikotov; Gaukhar Nussupbayeva; Madina Tleubergenova; Maria Pisareva; Nazym Tleumbetova
EPI_ISL_2086267	Enthabeni Lancet Laboratories	KRISP, KZN Research Innovation and Sequencing Platform	Emmanuel SJ; Giandhari J; Khan S; Lessells R; Mdlalose K; Naidoo Y; Pillay S; Ramphal U; Tegally H; Wilkinson E; York D; de Oliveira T
EPI_ISL_2112529	Epidemiology of Microbial Diseases, Yale School of Public Health	Epidemiology of Microbial Diseases, Yale School of Public Health	A.E.; A.F.; Alpert, T.; Breban; Brito; C.B.; C.C.; Fauver; Grubaugh; I.M.; Iwasaki, A.; J.E.; J.R.; Kalinich; Landry; Lucas, C.; M.E.; M.I.; M.L.; N.D.; Ott; Petrone; Rothman; Vogels; Watkins
EPI_ISL_1898554, EPI_ISL_2241494, EPI_ISL_2241623	Ethiopian Biotechnology Institute (EBTI)	International Centre for Genetic Engineering and Biotechnology (ICGEB) and ARGO Open Lab for Genome Sequencing	Alessandro Marcello; Danilo Licastro; Emanuele Orsini; Getnet Hailu; Hailu Dadi; Kassahun Tesfaye; Keyru Tuki; Kominist Asmamaw; Molalegne Bitew; Simeone Dal Monego; Yakob Gebregziabher Tsegay
EPI_ISL_877391	Eurofins Diatherix	Hudsonalpha Genome Sequencing Center	Ada Stewart; Jane Grimwood; Jenell Webber; Jennifer Cart; John Lovell; Joshua Stough; Leslie Malone; Lori H. Handley; Melissa Williams; Stefan Brzezinski; Teresa Jones; and Jeremy Schmutz
EPI_ISL_1570090, EPI_ISL_1571260, EPI_ISL_1571269, EPI_ISL_1846037, EPI_ISL_1846048, EPI_ISL_1846115, EPI_ISL_1846176, EPI_ISL_1846201, EPI_ISL_2259578			
see above	Eurofins LifeCodexx GmbH	Robert Koch Institute	
EPI_ISL_1571477, EPI_ISL_2110761, EPI_ISL_2110762, EPI_ISL_2316402, EPI_ISL_2316415, EPI_ISL_2845449	Eurofins MVZ Labor Gelsenkirchen	Robert Koch Institute	
EPI_ISL_2803756	Everight Diagnostic Laboratory	National Reference Laboratory, Nigeria Centre for Disease Control	Anthony Ahumibe; Catherine Okoi; Chimaobi Chukwu; Dr Chikwe Ihekweazu; Dr Ndodo Nnaemeka; Dr Omoare Adesuyi; Grace Esebanmen; Nwando Mba; Olusola Anuoluwapo Akanbi
EPI_ISL_2803754	Everight Diagnostic Laboratory	National Reference Laboratory, Nigeria Centre for Disease Control Durumi III, Gaduwa, Abuja, Nigeria	Anthony Ahumibe; Catherine Okoi; Chimaobi Chukwu; Dr Chikwe Ihekweazu; Dr Ndodo Nnaemeka; Dr Omoare Adesuyi; Grace Esebanmen; Nwando Mba; Olusola Anuoluwapo Akanbi
EPI_ISL_1712303	FSBI «NATIONAL MEDICAL RESEARCH CENTER FOR OBSTETRICS, GYNECOLOGY AND PERINATOLOGY NAMED AFTER ACADEMICIAN V.I.KULAKOV» MINISTRY OF HEALTHCARE OF THE RUSSIAN FEDERATION	Center for Precision Genome Editing and Genetic Technologies for Biomedicine, Pirogov Medical University, Moscow, Russian Federation	Anastasia Shut; Andrey Krivoy; Dmitriy Korostin; Margarita Korzhanova; Vera Belova; Yegor Botsmanov
EPI_ISL_1491576, EPI_ISL_1538420, EPI_ISL_1538429	Faculty of Medicine Vajira Hospital	COVID-19 Network Investigations (CONI) Alliance	Ampan Vimonvattana; Anan Manomaipiboon; Angkana Huang; Anthony R. Jones; Arporn Wangwiwatsin; Bhakbhoom Panthan; Chayanit Phutthanu; Chonticha Klungtong; Duangkamon Loesbanluechai; Ekawat Pasomsub; Elizabeth Batty; Insee Sensorn; Jakravoot Maneerit; Janjira Thaipadungpanit; Khajohn Joonlasak; Kingkan Rakmanee; Krittikorn Kumpornsin; Namfon Kotanan; Stefan Fernandez; Sunisa Dongphooyao; Thanat Chookajorn; Theerarat Kochakarn; Tonsan Hansirisathit; Treewat Watthanachockchai; Uraporn Phumisantiphong; Wasun Chantratita; Wipawee Thongsopa; Wudtichai Manasatienkij
EPI_ISL_1273102	Faculty of Medicine, Al-Quds University	Faculty of Medicine, Al-Quds University	Al-Jawabreh, A.; Dumaidi, K.; Ereqat, S.; Nasereddin, A.
EPI_ISL_451966	Federal Budget Institution of Science, State Research Center for Applied Microbiology & Biotechnology	Federal Budget Institution of Science, State Research Center for Applied Microbiology & Biotechnology	Abaimova A; Bakhteeva I; Blagodatskikh S; Bogun A; Borzilov A; Chekan L; Chernysh S; Denisenko E; Dentovskaya S; Detushev K; Detusheva E; Dyatlov I; Firstova V; Frolov V; Fursov M; Fursova N; Galkina E; Gapelchenkova T; Goncharova J; Gorbatov A; Hlyntseva A; Ivanov S; Kalmantayev T; Kalmantayeva O; Kanashenko M; Kartsev N; Kartseva A; Khomyakov A; Khramov M; Kislichkina A; Kolchanova A; Koroleva-Ushakova A; Kosilova I; Krasilnikova E; Kuzin V; Kuzina E; Makarova M; Marin M; Novikova T; Platonov M; Podkopaev Y; Ryabko A; Shaikhutdinova R; Shemyakin I; Shishkina L; Silkina M; Sizova A; Skryabin Y; Slukin P; Slukina N; Solomentsev V; Solovieva A; Teymurazov M; Timofeev V; Titareva G; Trunyakova A; Tyurin E; Vagayskaya A; Zeninskaya N; Zhumakaev R
EPI_ISL_2507666, EPI_ISL_2507677, EPI_ISL_2508274	Fimlab Laboratoriot Oy Tampere	Expert Microbiology, National Institute for Health and Welfare	Carita Savolainen-Kopra; Erika Lindh; Haider al-Hello; Jani Halkilahti; Kirsi Liitsola; Niina Ikonen; Olli Vapalahti; Pekka Ellonen; Phuoc Truong; Päivi Laurila; Ravi Kant; Sari Hannula; Soile Blomqvist; Teemu Smura
EPI_ISL_509726, EPI_ISL_1030086, EPI_ISL_2008721, EPI_ISL_2008761	Florida Bureau of Public Health Laboratories	Florida Bureau of Public Health Laboratories	Jason Blanton; Sarah Schmedes
EPI_ISL_590899, EPI_ISL_708077	Foerde Hospital, Department of Microbiology	Norwegian Institute of Public Health, Department of Virology	Hilde Elshaug; Hilde Vollan; Kamilla Heddeland Instefjord; Karoline Bragstad; Kathrine Stene-Johansen; Marie Paulsen Madsen; Olav Hungnes; Rasmus Riis Kopperud
EPI_ISL_476139	Folkhalsomyndigheten	The Public Health Agency of Sweden	Anna Risberg; Anna-Malin Linde; Karin Tegmark-Wisell; Maria Lind Karlberg; Mattias Haukland; Olov Svartstrom; Oskar Karlsson Lindsjo; Petra Edquist; Reza Advani; Sandra Broddesson; Shamam Muradrasoli
EPI_ISL_2889833, EPI_ISL_2889839, EPI_ISL_2889841, EPI_ISL_2889844, EPI_ISL_2889846, EPI_ISL_2889847, EPI_ISL_2889848, EPI_ISL_2889849, EPI_ISL_2889850, EPI_ISL_2889854			
see above	Fondation Congolaise pour la recherche medicale (FCRM)	Fondation Congolaise pour la Recherche Médicale	Abel Lissom; Batchi-Bouyou Armel Landry; Francine Ntoumi; Jean Claude Djontu; Mfoutou Mapanguy Claujens Chastel; Thirumalaisamy P. Velavan
EPI_ISL_581455, EPI_ISL_581491, EPI_ISL_581492	Fondation Congolaise pour la recherche medicale (FCRM)	NGS Competence Center Tübingen, Institut für Medizinische Mikrobiologie und Hygiene, Universitätsklinikum Tübingen	Angel Angelov
EPI_ISL_2087052	Fondation Congolaise pour la recherche medicale (FCRM), Francine Ntoumi	Centre de Recherche sur les Maladies Infectieuses	Mfoutou Mapanguy Claujeans Chastel and Batchi-Bouyou Armel Landry; Prof. Dr. Thirumalaisamy P. Velavan; Prof. Francine Ntoumi
EPI_ISL_2964949, EPI_ISL_2964950, EPI_ISL_2964955, EPI_ISL_3040125, EPI_ISL_3040129, EPI_ISL_3040130, EPI_ISL_3040133, EPI_ISL_3040134, EPI_ISL_3040135, EPI_ISL_3040137			
see above	Fondation Congolaise pour la recherche medicale (FCRM), Francine Ntoumi	Fondation Congolaise pour la Recherche Médicale	Batchi-Bouyou Armel Landry; Dr. Abel Lissom; Dr. Jean Claude Djontu; Mfoutou Mapanguy Claujens Chastel; Prof. Dr. Thirumalaisamy P. Velavan; Prof. Francine Ntoumi
EPI_ISL_1671925, EPI_ISL_1840863, EPI_ISL_1854769,	Fondation Congolaise pour la recherche medicale (FCRM), Francine Ntoumi	Institute of Tropical Medicine	Mfoutou Mapanguy Claujeans Chastel and Batchi-Bouyou Armel Landry; Prof. Dr. Thirumalaisamy P. Velavan; Prof. Dr. Thirumalaisamy P. Velavan and Prof. Francine Ntoumi; Prof. Francine Ntoumi

EPI_ISL_1854789, EPI_ISL_1854794, EPI_ISL_1857281			
EPI_ISL_912358, EPI_ISL_912371, EPI_ISL_912394	Fondation Congolaise pour la recherche medicale (FCRM), Francine Ntoumi	NGS Competence Center Tuebingen, Institut für Medizinische Mikrobiologie und Hygiene, Universitaetsklinikum Tübingen	Angel Angelov
EPI_ISL_1666171, EPI_ISL_1992782, EPI_ISL_2038596, EPI_ISL_2525602, EPI_ISL_2785604, EPI_ISL_2785740, EPI_ISL_2928732			
see above	Fulgent Genetics	Centers for Disease Control and Prevention Division of Viral Diseases, Pathogen Discovery	Adrian Paskey; Becky Tsai; Benafsh Sapra; Benjamin Rambo-Martin; Christopher Gulvick; Clinton R. Paden; Dakota Howard; Darlene Wagner; Dhwani Batra; Doreen Ng; Duncan MacCannell; Harry Gao; James Xie; Jason Caravas; John Gao; Joseph Fierro; Kara Moser; Matthew Schmerer; Mickey Li; Peter W. Cook; Scott Sammons; Shatavia Morrison; Yan Meng; Yvette Unoarumhi
EPI_ISL_1026982, EPI_ISL_1027144, EPI_ISL_1068425	Fulgent Genetics	Fulgent Genetics	Becky Tsai; Benafsh Sapra; Doreen Ng; Harry Gao; James Xie; John Gao; Joseph Fierro; Mickey Li; Yan Meng
EPI_ISL_1182612	Fundação Ezequiel Dias (FUNED)	Coordenação Geral de Laboratórios de Saúde Pública (CGLAB/DAEVS/SVS/MS)	Vagner Fonseca; et al.
EPI_ISL_493362, EPI_ISL_2788586, EPI_ISL_2925776, EPI_ISL_3008324	Furst Medical Laboratory	Norwegian Institute of Public Health, Department of Virology	Atiya R Ali; Debech Nadia; Engebretsen Serina Beate; Garcia Llorente Ignacio; Hilde Elshaug; Hilde Vollan; Jon Bråte; Kamilla Heddeland Instefjord; Karoline Bragstad; Kathrine Stene-Johansen; Line Victoria Moen; Marie Paulsen Madsen; Olav Hungnes; Pedersen Benedikte Nevjen; Rasmus Riis Kopperud
EPI_ISL_1318590	GA Department of Public Health Laboratory	Centers for Disease Control and Prevention Division of Viral Diseases, Pathogen Discovery	Anna Montmayeur; Anna Uehara; Ben L. Rambo-Martin; Clinton R. Paden; Dhwani Batra; Haibin Wang; Jasmine Padilla; Jing Zhang; Justin Lee; Katie Dillon; Krista Queen; Kristen Knipe; Kristine Lacek; Lori Rowe; Mark Burroughs; Matthew Schmerer; Mili Sheth; Peter W. Cook; Rachel Marine; Sam Shepard; Sarah Nobles; Shoshona Le; Suxiang Tong; Yan Li; Ying Tao
EPI_ISL_1672509, EPI_ISL_2333929	GH A.CHENEVIER-H.MONDOR	Department of Virology, Henri Mondor University Hospital, Assistance Publique Hôpitaux de Paris, Université Paris-Est Créteil, INSERM U955	Alexandre Soulier; Christophe Rodriguez; Elisabeth Trawinski; Guillaume Gricourt; Jean-Michel Pawlotsky; Melissa N'Debi; Slim Fourati; Vanessa Demontant
EPI_ISL_2779388	GK Prison Kiambu	USAMRD-A, Basic Science Laboratory	Alan Lemtudo; Beth Mutai; Brian Andika; Carol Kifude; Clement Masakwe; Eric Muthanje; Esther Omuseni; Faith Sigei; Gathii Kimita; George Awinda; John Waitumbi; Josphat Nyataya; Rachel Githii; Rehema Liyai; Stephen Ochola
EPI_ISL_2779313, EPI_ISL_2779316	GK Prisons Dispensary Kapsabet	USAMRD-A, Basic Science Laboratory	Alan Lemtudo; Beth Mutai; Brian Andika; Carol Kifude; Clement Masakwe; Eric Muthanje; Esther Omuseni; Faith Sigei; Gathii Kimita; George Awinda; John Waitumbi; Josphat Nyataya; Rachel Githii; Rehema Liyai; Stephen Ochola
EPI_ISL_2864606	Ga East Hospital	UHAS COVID-19 Lab	John O. Gyapong and the UHAS COVID-19 Lab Team; Jones Gyamfi; Kwabena O. Duedu; Reuben Ayivor-Djanie
EPI_ISL_831977	Gavle klinisk mikrobiologi	The Public Health Agency of Sweden	Department of Microbiology; The Public Health Agency of Sweden
EPI_ISL_730577, EPI_ISL_984749	Gazi University Faculty of Medicine, Medical Virology Laboratory	Gazi University Faculty of Medicine, Medical Virology Laboratory	Erdem Şahin; Gülendam Bozdayı; Hager Muftah; Işıl Fidan; Kayhan Çağlar; Murat Dizbay; Selin Yiğit; Shaknoza Sarzhanova; Özlem Güzel Tunçcan
EPI_ISL_3026015, EPI_ISL_3026016, EPI_ISL_3026019, EPI_ISL_3026020	Geelong Centre for Emerging Infectious Diseases, Deakin University	Geelong Centre for Emerging Infectious Diseases, Deakin University	Alexandersen, S.; Bhatta; Chamings, A.; T.R.
EPI_ISL_2681409, EPI_ISL_2681439	General Hospital "8th of September"	Laboratory of virology and molecular diagnostics, Institute of Public Health	Boshevska G; Janchevska E.; Kuzmanovska M
EPI_ISL_933566, EPI_ISL_934314	General Hospital - Bitola	Research Center for Genetic Engineering and Biotechnology "Georgi D. Efremov" , Macedonian Academy of Sciences and Arts	RCGEB - MASA
EPI_ISL_933775	General Hospital - Ohrid	Research Center for Genetic Engineering and Biotechnology "Georgi D. Efremov" , Macedonian Academy of Sciences and Arts	RCGEB - MASA
EPI_ISL_677674	General Hospital - Prilep	Research Center for Genetic Engineering and Biotechnology "Georgi D. Efremov" , Macedonian Academy of Sciences and Arts	RCGEB - MASA
EPI_ISL_933602, EPI_ISL_934413	General Hospital - Shtip	Research Center for Genetic Engineering and Biotechnology "Georgi D. Efremov" , Macedonian Academy of Sciences and Arts	RCGEB - MASA
EPI_ISL_3020343	General Hospital Tetovo	Laboratory of virology and molecular diagnostics, Institute of Public Health	Boshevska G; Janchevska E.; Kuzmanovska M
EPI_ISL_406798	General Hospital of Central Theater Command of People's Liberation Army of China	BGI & Institute of Microbiology, Chinese Academy of Sciences & Shandong First Medical University & Shandong Academy of Medical Sciences & General Hospital of Central Theater Command of People's Liberation Army of China	Weifeng Shi and Zhenhong Hu; Weijun Chen; Yuhai Bi
EPI_ISL_746523, EPI_ISL_1167697, EPI_ISL_1167721, EPI_ISL_1167794, EPI_ISL_1167814, EPI_ISL_1167878, EPI_ISL_1167922, EPI_ISL_1167925, EPI_ISL_1300477, EPI_ISL_1321468, EPI_ISL_1321493, EPI_ISL_1321508, EPI_ISL_1534569, EPI_ISL_1534583, EPI_ISL_1541011, EPI_ISL_1541012, EPI_ISL_1541035, EPI_ISL_1633372, EPI_ISL_1633383, EPI_ISL_1633418, EPI_ISL_1633500, EPI_ISL_2009203, EPI_ISL_2009256, EPI_ISL_2009293, EPI_ISL_2009637, EPI_ISL_2391042, EPI_ISL_2391174, EPI_ISL_2391176, EPI_ISL_2391269, EPI_ISL_2391355, EPI_ISL_2391412, EPI_ISL_2391442, EPI_ISL_2543475, EPI_ISL_2543539, EPI_ISL_2543664, EPI_ISL_2557275, EPI_ISL_2597292, EPI_ISL_2659168, EPI_ISL_2659197, EPI_ISL_2756220, EPI_ISL_2756329, EPI_ISL_2756330, EPI_ISL_2756336, EPI_ISL_2756337, EPI_ISL_2892690, EPI_ISL_2892741, EPI_ISL_2892797, EPI_ISL_2894889, EPI_ISL_2896177			
see above	Genetica Molecular and Subdepartamento de Virologia ISP Chile	Instituto de Salud Publica de Chile	Andres Castillo; Barbara Parra; Constanza Campano; Gisselle Barra; Jaime Lagos; Javier Tognarelli; Jorge Fernandez; Karen Orostica; Loredana Arata; Patricia Bustos; Rodrigo Fasce; Soledad Ulloa
EPI_ISL_480245, EPI_ISL_735371	Genomic Laboratory (GLAB) (Conjoint lab of Health Directorate of Istanbul and Istanbul Technical University)	Genomic Laboratory (GLAB), Istanbul Technical University	Arzu Irvem; Ayse Serra Ozel; Betsi Kose; Betsi Köse; Bugra Agaoglu; Elifnaz Çelik; Gizem Alkurt; Gizem Dinler Doganay; Ilker Karacan; Jale Yildiz; Levent Doganay; Mehtap Aydin; Mehtap Aydın; Nihat Bugra Agaoglu; Nilsun Altunal; Nisan Denizce Can; Ozlem Akgun Dogan; Payam Zolfagharian; Tugba Kizilboga Akgun; Yasemin Kendir Demirkol
EPI_ISL_1673680	Genomic Research Lab, BCSIR	Genomic Research Lab, BCSIR	Abu Sayeed Mohammad Mahmud; Barna Goswami; Eshrar Osman; Iffat Jahan; Md. Ahasan Habib; Md. Murshed Hasan Sarkar; Md. Saddam Hossain; Md. Salim Khan; Mohammad Mohi Uddin; Mohammad Samir Uzzaman; Shahina Akter; Tanjina Akhter Banu
EPI_ISL_2862015	Genomic Research Laboratory, Bangabandhu Sheikh Mujib Medical University (BSMMU) Shahbag, Dhaka_1000	Genomic Research Laboratory, BSMMU	Arifa Akram; Laila Anjuman Banu; Md.Saydur Rahman; Md.Sharfuddin Ahmed; Sharadindu Kanti Sinha
EPI_ISL_632908	Genomic Sciences, Rehman Medical Institute	Genomic Sciences, Rehman Medical Institute	Afridi; Ali, J.; H. and Jehanzeb, V.; Haider; Jan; S.A.; Sabiha, B.; U.K.
EPI_ISL_812783, EPI_ISL_812784, EPI_ISL_812785, EPI_ISL_812786, EPI_ISL_812787, EPI_ISL_812788, EPI_ISL_812789, EPI_ISL_812790, EPI_ISL_812791, EPI_ISL_812792, EPI_ISL_812793, EPI_ISL_812794, EPI_ISL_812795, EPI_ISL_812796, EPI_ISL_812797, EPI_ISL_812798, EPI_ISL_812799, EPI_ISL_812800, EPI_ISL_812801, EPI_ISL_812802, EPI_ISL_812803, EPI_ISL_812804, EPI_ISL_812805, EPI_ISL_812806, EPI_ISL_812807, EPI_ISL_812808, EPI_ISL_812809, EPI_ISL_812810, EPI_ISL_812811, EPI_ISL_812812, EPI_ISL_812813, EPI_ISL_812814, EPI_ISL_812815, EPI_ISL_812816, EPI_ISL_812817, EPI_ISL_812818, EPI_ISL_812819, EPI_ISL_812820, EPI_ISL_812821, EPI_ISL_812822, EPI_ISL_812823, EPI_ISL_812824, EPI_ISL_812825, EPI_ISL_812826, EPI_ISL_812827, EPI_ISL_812828, EPI_ISL_812829, EPI_ISL_812830, EPI_ISL_812831, EPI_ISL_812832, EPI_ISL_812833, EPI_ISL_812834, EPI_ISL_812835, EPI_ISL_812836, EPI_ISL_812837, EPI_ISL_812838, EPI_ISL_812839, EPI_ISL_812840, EPI_ISL_812841, EPI_ISL_812842, EPI_ISL_812843, EPI_ISL_812844, EPI_ISL_812845, EPI_ISL_812846, EPI_ISL_812847, EPI_ISL_812848, EPI_ISL_812849, EPI_ISL_812850, EPI_ISL_812851, EPI_ISL_812852, EPI_ISL_812853, EPI_ISL_812854, EPI_ISL_812855, EPI_ISL_812856, EPI_ISL_812857, EPI_ISL_812858, EPI_ISL_812859, EPI_ISL_812860, EPI_ISL_812861, EPI_ISL_812862, EPI_ISL_812863, EPI_ISL_812864, EPI_ISL_812865, EPI_ISL_812866, EPI_ISL_812867, EPI_ISL_812868, EPI_ISL_812869, EPI_ISL_812870, EPI_ISL_812871, EPI_ISL_812872, EPI_ISL_2566467, EPI_ISL_2566468, EPI_ISL_2566469, EPI_ISL_2566470, EPI_ISL_2566471, EPI_ISL_2566472, EPI_ISL_2566473, EPI_ISL_2566474, EPI_ISL_2566475, EPI_ISL_2566476, EPI_ISL_2566477, EPI_ISL_2566478, EPI_ISL_2566479, EPI_ISL_2566480, EPI_ISL_2566481, EPI_ISL_2566482, EPI_ISL_2566483, EPI_ISL_2566484, EPI_ISL_2566485, EPI_ISL_2566486, EPI_ISL_2566487, EPI_ISL_2566488, EPI_ISL_2566489, EPI_ISL_2566490, EPI_ISL_2566491, EPI_ISL_2566492, EPI_ISL_2566493, EPI_ISL_2566494, EPI_ISL_2566495, EPI_ISL_2566496, EPI_ISL_2566497, EPI_ISL_2566498, EPI_ISL_2566499, EPI_ISL_2566500, EPI_ISL_2566501, EPI_ISL_2566502, EPI_ISL_2566503, EPI_ISL_2566504, EPI_ISL_2566505, EPI_ISL_2566506, EPI_ISL_2566507, EPI_ISL_2566508, EPI_ISL_2566509, EPI_ISL_2566510, EPI_ISL_2566511, EPI_ISL_2566512, EPI_ISL_2566513, EPI_ISL_2566514, EPI_ISL_2566515, EPI_ISL_2566516, EPI_ISL_2566517, EPI_ISL_2566518, EPI_ISL_2566519, EPI_ISL_2566520, EPI_ISL_2566521, EPI_ISL_2566522, EPI_ISL_2566523, EPI_ISL_2566524, EPI_ISL_2566525, EPI_ISL_2566526, EPI_ISL_2566527, EPI_ISL_2566528, EPI_ISL_2566529, EPI_ISL_2566530			
see above	Genomics Program, Children Cancer Hospital	Genomics Program, Children Cancer Hospital	Abdelaziz, H.; Abdo, I.; Abouelnaga, S.; Almeldin, A.; Amer, K.; Bakry, U.; Diab, A.; El-Shaqnqery, H.; El-Zayat, M.; ElHaddad, A.; ElHalafawy, A.; Elnaqeeb, M.; Farawela, H.; Farawyla, H.; Gomaa, C.; Hadad, A.; Halafawy, A.; Hammad, M.; Hassan, R.; Hassan, W.; Hatem, A.; Hossam, M.; Hussein, S.; Ismail, J.; Jalal, D.; Magdeldin, S.; Mansour, T.; Monuir, G.; Saaid, M.; Said, D.; Salah, H.; Samir, O.; Sayed, A.; Shalaby, L.; Soliman, M.; Soliman, S.; Yahia, A.; shalaby, L.
EPI_ISL_2510688	Genomics Program, Children Cancer Hospital	Genomics Program, Children Cancer Hospital	Abdelaziz, H.; Abdo, I.; Abouelnaga, S.; Almeldin, A.; Amer, K.; Diab, A.; El-Shaqnqery, H.; El-Zayat, M.; ElHaddad, A.; ElHalafawy, A.; Elnaqeeb, M.; Farawela, H.; Hammad, M.; Hassan, R.; Hassan, W.; Hatem, A.; Hossam, M.; Hussein, S.; Ismail, J.; Jalal, D.; Mansour, T.; O. and Sayed, A.; Saaid, M.; Said, D.; Salah, H.; Samir; Shalaby, L.; Soliman, S.; Yahia, A.
EPI_ISL_2434978	Genomik Solidaritas Indonesia Laboratorium	Genomik Solidaritas Indonesia Laboratorium	Alfin Mohammad Abdillah; Anindya Pradipta Susanto; Annisa Muthiah Sukirman; Ariel Pradipta; Carissa Sintca Wijaya; Dhahlia Agustina Cahyono; Gracia Felias Enos Korompis; Meutia Ayuputeri Kumaheri; Normastuti Adhini Tantyo; Vania Gavrila Wikasa
EPI_ISL_2140696, EPI_ISL_2802110	George Hospital wc GRH	NHLS/UCT	Arash Iranzadeh; Bruna Galvao; Carolyn Williamson; Deelan Doolabh; Diana Hardie; Gert Marais; Innocent Mudau; Kruger Marais; Lynn Tyers; Marvin Hsiao; Stephen Korsman
EPI_ISL_1744345, EPI_ISL_1744346, EPI_ISL_2248892, EPI_ISL_2340163, EPI_ISL_2340181	Germano de Sousa	Instituto Nacional de Saude (INSA)	Borges et al
EPI_ISL_2340334, EPI_ISL_2340355	Germano de Sousa	Instituto Nacional de Saude (INSA) and BioSystems & Integrative Sciences Institute (BioISI) Genomics Unit, FCUL	Borges et al
EPI_ISL_1853560	Germano de Sousa	Instituto Nacional de Saude (INSA) and Instituto Gulbenkian de Ciencia (IGC)	Borges et al
EPI_ISL_960672	Germano de sousa	Instituto Gulbenkian de Ciencia	Cathy Paulino; João Costa; João Sobral; Maria Costa; Ricardo Leite; Susana Ladeiro
EPI_ISL_933769	Gerontology Institute - Skopje	Research Center for Genetic Engineering and Biotechnology "Georgi D. Efremov" , Macedonian Academy of Sciences and Arts	RCGEB - MASA

EPI_ISL_890188	Gonoshasthaya-RNA Research Center, Gonoshasthaya-RNA Molecular Diagnostics and Research Center	Gonoshasthaya-RNA Research Center, Gonoshasthaya-RNA Molecular Diagnostics and Research Center	Adnan, N.; Ahmed; Akter, S.; Ali, T.; Azmuda, N.; Chaity, M.; Haq; Jahan, N.; Jamiruddin; Jamiruddin, M.; Khandker; Khondoker; Liza; M.A.; M.F.; M.R.; M.U.; Marzan, M.; Mou; Nahar, S.; Oishee, M.; S.M.; S.S.; Sharif, N.; Sharmin, S.; T.J.
EPI_ISL_774976	Gonoshasthya-RNA Molecular Diagnostic and Research Center	Gonoshasthya-RNA Molecular Diagnostic and Research Center	Firoz Ahmed; Maha Jamiruddin; Mahfuza Marzan; Md. Ahsanul Haq; Mohd. Raeed Jamiruddin; Mohib Ullah Khondoker; Mousumi Chaity; Mumtarin Jannat Oishee; Nafisa Azmuda; Nihad Adnan; Nowshin Jahan; Salma Akter; Sayeda Moriam Liza; Shahad Saif Khandker; Shahana Sharmin; Tamanna Ali; Taslin Jahan Mou
EPI_ISL_496860, EPI_ISL_1225352, EPI_ISL_1225354, EPI_ISL_1225513, EPI_ISL_1225516, EPI_ISL_1225521, EPI_ISL_1225563, EPI_ISL_1225574, EPI_ISL_1225577, EPI_ISL_1502846, EPI_ISL_1502915, EPI_ISL_1502937, EPI_ISL_1502983, EPI_ISL_1502997, EPI_ISL_1503020, EPI_ISL_1503114, EPI_ISL_1503116			
see above	Gorgas Memorial Laboratory of Health Studies	Gorgas Memorial Laboratory of Health Studies	Adriana Weeden; Alejandra Valoy; Alexander A Martinez; Alexander Martinez; Ambar Moreno; Anyuri Ortiz; Brechla Moreno; Castillo Jorge; Claudia Gonzalez; Claudia Gonzalez Sandra Lopez-Verges; Daniel Castillo; Danilo Franco; Davis Beltran; Dimelza Arauz; Elimelec Valdespino; Franco Danilo; Gonzalez Claudia; Gretel Vasquez; Ilka Guerra; Isela Guerrero; Jessica Gondola; Jim Chang; Juan Miguel Pascale; Layda Abrego; Leyda Abrego; Lisseth Saenz; Lopez-Verges Sandra; Mabel Martinez-Montero; Maria Chen-German; Marlene Castillo; Marlenne Castillo; Martinez Alexander; Melissa Gaitan; Moreno Ambar; Moreno Brechla; Oris Chavarria; Ortiz Alma; Rita Corrales; Rita Rodriguez; Sandra Lopez-Verges; Yamilka Diaz; Yaneth Pitti; Zumara Chaverra
EPI_ISL_1302710	Gravity Diagnostics	Kentucky State Public Health Lab	Joshua Tobias; Karim George; Matthew Johnson; Rachel Zinner; Rhonda Lucas; Stephanie Lunn; Vaneet Arora; William Grooms
EPI_ISL_2602113, EPI_ISL_2614003, EPI_ISL_2985763	Gravity Diagnostics, LLC	Gravity Diagnostics, LLC	Gravity Diagnostics
EPI_ISL_2301649, EPI_ISL_2301783, EPI_ISL_2369069, EPI_ISL_2371278	Greek Genome Center, Biomedical Research Foundation of the Academy of Athens (BRFAA)	Greek Genome Center, Biomedical Research Foundation of the Academy of Athens (BRFAA)	Dimitrios Thanos; Emmanouil Athanasiadis; Giannis Vatsellas; Katerina Zoi; Theodoros Loupis
EPI_ISL_1040654, EPI_ISL_1706573, EPI_ISL_2140691, EPI_ISL_2140704, EPI_ISL_2140712, EPI_ISL_2375973, EPI_ISL_2621054, EPI_ISL_2876955			
see above	Groote Schuur Hospital wc GSH	NHLS/UCT	Arash Iranzadeh; Bruna Galvao; Carolyn Williamson; Deelan Doolabh; Diana Hardie; Emmanuel SJ; Gert Marais; Innocent Mudau; Kruger Marais; Lynn Tyers; Marvin Hsiao; Stephen Korsman; Tegally H; de Oliveira T
EPI_ISL_2547418	Groote Schuur Hospital wc GSH	National Health Laboratory Service/University of Cape Town (NHLS/UCT)	Arash Iranzadeh; Bruna Galvao; Carolyn Williamson; Deelan Doolabh; Diana Hardie; Gert Marais; Innocent Mudau; Lynn Tyers; Marvin Hsiao; Stephen Korsman
EPI_ISL_699059	Group 42 (G42) Healthcare, Abu Dhabi, United Arab Emirates; Department of Health, The United Arab Emirates	G42 Healthcare	Ashish Koshy; Budoor Alqarni; Denghui Liu; Fang Chen; Hanif Khalak; Huanming Yang; Javier Quilez; Jian Wang; Junhua Li; Ke Liang; Long Lin; Mohammed Saifuddin Fasihuddin; Nan Qiao; Nawal Ahmed Mohamed Al Kaabi; Pauline Ogrodzki; Pei Wu; Peng Xiao; Pengjuan Liu; Rong Liu; Sally Mahmoud; Siyang Liu; Stephen S. Francis; Tao Ma; Vinay Kusuma; Walid Abbas Zaher; Weibin Liu; Wenjun He; Xavier Anton; Xin Jin; Xin Meng; Xinyu Huang; Xun Xu; Zhaorong Yuan
EPI_ISL_3033081	Grupo de Genómica y Bioinformática del Instituto de Investigación de la Cadena Láctea CONICET-INTA on behalf of 'Proyecto Argentino Interinstitucional de genomica de SARS-CoV-2' (PAIS Consortium)	Grupo de Genómica y Bioinformática del Instituto de Investigación de la Cadena Láctea CONICET-INTA on behalf of 'Proyecto Argentino Interinstitucional de genomica de SARS-CoV-2' (PAIS Consortium)	AF; Amadio; Eberhardt; Irazoqui; JM; MF
EPI_ISL_1824180, EPI_ISL_2339776, EPI_ISL_2339777, EPI_ISL_2339778, EPI_ISL_2339779, EPI_ISL_2339780, EPI_ISL_2339781, EPI_ISL_2339782, EPI_ISL_2339783, EPI_ISL_2339784, EPI_ISL_2339785, EPI_ISL_2339786, EPI_ISL_2339787, EPI_ISL_2339788, EPI_ISL_2339789, EPI_ISL_2339790, EPI_ISL_2339791, EPI_ISL_2339792, EPI_ISL_2339793, EPI_ISL_2339794, EPI_ISL_2603146			
see above	Grupo de Investigación en Enfermedades Tropicales del Ejército (GINETEJ), Laboratorio de Referencia e Investigación, Dirección de Sanidad Ejército, Bogotá, Colombia	Centro de Investigaciones en Microbiología y Biotecnología-UR (CIMBIUR), Facultad de Ciencias Naturales, Universidad del Rosario, Bogotá, Colombia	Camilo A. Correa-Cárdenas; Carolina Oliveros; Claudia Méndez; Elizabeth K. Márquez; Frank de los Santos Ortiz; Juan David Ramírez; Julie Pérez; Lorena Albarracín; Luz H. Patiño; Maria Clara Duque; Marina Muñoz; María Teresa Alvarado; Nathalia Ballesteros; Sergio Castañeda; Sergio Gutíerrez-Riveros; Yanira Romero; Zulma Cucunubá
EPI_ISL_1273073, EPI_ISL_1823585, EPI_ISL_2451659, EPI_ISL_2787627, EPI_ISL_2787642	Guam Public Health Laboratory	Centers for Disease Control and Prevention Division of Viral Diseases, Pathogen Discovery	Alison Laufer Halpin; Anna Montmayeur; Anna Uehara; Ben L. Rambo-Martin; Clinton R. Paden; Dakota Howard; Darlene Wagner; Dave Wentworth; Dhwani Batra; Haibin Wang; Jasmine Padilla; Jing Zhang; Justin Lee; Katie Dillon; Krista Queen; Kristen Knipe; Kristine Lacek; Lori Rowe; Mark Burroughs; Matthew Schmerer; Mili Sheth; Peter Cook; Peter W. Cook; Rachel Marine; Sam Shepard; Sarah Nobles; Shoshona Le; Suxiang Tong; Vivien Dugan; Yan Li; Ying Tao; Yvette Unoarumhi
EPI_ISL_509701	Guatemala Ministry of Public Health	Pathogen Discovery, Respiratory Viruses Branch, Division of Viral Diseases, Centers for Disease Control and Prevention	Anna Uehara; Clinton Paden; Haibin Wang; Jing Zhang; Krista Queen; Suxiang Tong; Yan Li; Ying Tao
EPI_ISL_547739, EPI_ISL_942722	Gundersen Molecular Diagnostics Laboratory	Kabara Cancer Research Institute	Craig S. Richmond; Paraic A. Kenny
EPI_ISL_1853561	H Fernando Fonseca	Instituto Nacional de Saude (INSA) and Institute of Biomedicine (iBiMed), Universidade de Aveiro	Borges et al
EPI_ISL_693535	H Vila Franca Xira	Instituto Nacional de Saude (INSA)	Borges et al
EPI_ISL_2695606, EPI_ISL_2709863	HELEN JOSEPH	National Institute for Communicable Diseases of the National Health Laboratory Service	Amoako DG; Bhiman JN; Everatt J; Ismail A; Mahlangu B; Mnguni A; Mohale T; Ntuli N; Scheepers C
EPI_ISL_2984814, EPI_ISL_2984893, EPI_ISL_3024595	HELEN JOSEPH LABORATORY	National Institute for Communicable Diseases of the National Health Laboratory Service	Amoako DG; Bhiman JN; Everatt J; Ismail A; Mahlangu B; Mnguni A; Mohale T; Ntuli N; Scheepers C
EPI_ISL_2385290, EPI_ISL_2523862, EPI_ISL_2899790	HELIX LLC	WHO National Influenza Centre Russian Federation	Andrey Komissarov; Artem Fadeev; Daria Danilenko; Dmitry Lioznov; Elena Nabieva; Georgii Bazykin; Kirill Varchenko; Ksenia Safina; Kseniya Komissarova; Maria Pisareva; Maria Timofeeva; Mikhail Bakaev; Nikita Yolshin; Oula Mansour; Oula Masour; Tamila Musaeva; Veronika Eder
EPI_ISL_1180745, EPI_ISL_1965453	HG Pharma GmbH	Bergthaler laboratory, CeMM Research Center for Molecular Medicine of the Austrian Academy of Sciences	Andreas Bergthaler; Anna Schedl; Bekir Erguner; Benedikt Agerer; Christoph Bock; Fabian Amman; Jan Laine; Lukas Endler; Maelle Le Moing; Martin Senekowitsch; Michael Schuster; Petr Triska; Thomas Penz
EPI_ISL_955236	HGR 1 VICENTE GUERRERO	BIOBANCO / COCTI	Arias C; Borja-Aburto VH; Grajales-Muñiz C; Grande R; Isa P; López S; Muñoz-Medina JE; Ochoa Carrera LA; Rojas-Mendoza T; Santacruz Tinoco CE; Sánchez A; Taboada B
EPI_ISL_1805649	HIC	Instituto Nacional de Salud- Dirección de Investigación en Salud Pública	Carlos Franco-Muñoz; Carmen Osorio; Diana Malo; Diego A. Álvarez-Díaz; Diego Andrés Prada; Gerardo Santamaría; Hector Alejandro Ruiz-Moreno; Jhonnatan Reales-González; Jorge Rivera; Juan Camilo Martinez; Julian Naizaque; Katherine Laiton-Donato; Lisseth Pardo; Magdalena Wiesner; Marcela Mercado-Reyes; Maria T. Herrera-Sepúlveda; Marta Lopez Blanco; Martha Lucia Ospina Martinez; Paola Rojas; Sergio Gomez; Sheryll Corchuelo; Ángela Alarcon Cruz
EPI_ISL_2484763, EPI_ISL_2484784, EPI_ISL_3019487, EPI_ISL_3019492	HOME QUARANTINE TASKFORCE	Hong Kong Department of Health	Alan K.L. Tsang; Dominic N.C. Tsang; Edman T.K. Lam; Gannon C.K. Mak; Ken H.L. Ng; Peter C.W. Yip; Peter K.C. Cheng; Rickjason C.W. Chan
EPI_ISL_1940037	HOSAHALLI	inStem NCBS - INSACOG	Uma Ramakrishnan Dasaradhi Palakodeti Aswin SaiNarain
EPI_ISL_2209295	HOSP MUN DE MOGI DAS CRUZES PREF WALDEMAR COSTA FILHO	Instituto Butantan / Mendelics	Antonio Jorge Martins; Claudia Renata dos Santos Barros; David Schlesinger; Debora Botequio Moretti; Dimas Tadeu Covas; Elaine Cristina Marqueze; Elaine Vieira Santos; Evandra Strazza Rodrigues; Heidge Fukumasu; Jayme Augusto de Souza-Neto; José Salvatore Leister Patané; Luiz Alcantara; Luiz Lehmann Coutinho; Maria Carolina Elias; Maurício Lacerda Nogueira; Rafael dos Santos Bezerra; Raul Machado Neto; Rejane Maria Tommasini Grotto; Ricardo Haddad; Sandra Coccuzzo Sampaio Vessoni; Simone Kashima; Svetoslav Nanev Slavov; Vincent Louis Viala
EPI_ISL_2272985	HOSPITAL CIUDAD NEILY	Inciensa, Instituto Costarricense de Investigación y Enseñanza en Nutrición y Salud	Adriana Godínez; Claudio Soto-Garita; Estela Cordero; Francisco Duarte; Hebleen Porras; Joselyn Prado & Raúl Zeledón-Mayorga; José Luis Vargas; Mariela Gutiérrez; Melany Calderón
EPI_ISL_2103379, EPI_ISL_2103412, EPI_ISL_2272972	HOSPITAL DR. FERNANDO ESCALANTE PRADILLA	Inciensa, Instituto Costarricense de Investigación y Enseñanza en Nutrición y Salud	Adriana Godínez; Caterina Guzmán; Claudio Soto-Garita; Estela Cordero; Francisco Duarte; Hebleen Porras; Joselyn Prado & María Fernanda Matamoros Ceciliano; José Luis Vargas; Mariela Gutiérrez; Mariela Gutiérrez. Joselyn Prado; Melany Calderón; Nazareth Ruiz & Joselyn Prado; Nazareth Ruiz & Lizzeth Blanco
EPI_ISL_2103402	HOSPITAL DR. MAX TERAN VALLS	Inciensa, Instituto Costarricense de Investigación y Enseñanza en Nutrición y Salud	Adriana Godínez; Caterina Guzmán; Claudio Soto-Garita; Estela Cordero; Francisco Duarte; Hebleen Porras; Joselyn Prado; José Luis Vargas; Mariela Gutiérrez; Melany Calderón; Nazareth Ruiz & María José Gómez-Umaña
EPI_ISL_3037832	HOSPITAL GUAPILES [POCOCI/LIMON]	Inciensa, Instituto Costarricense de Investigación y Enseñanza en Nutrición y Salud	Adriana Godínez; Caterina Guzmán; Claudio Soto-Garita; Estela Cordero; Francisco Duarte; Hebleen Porras; José Luis Vargas; Mariela Gutiérrez Joselyn Prado; Melany Calderón; Nazareth Ruiz & Cristian Pérez
EPI_ISL_2502729	HOSPITAL LA ANEXION	Inciensa, Instituto Costarricense de InvestigaciOn y Enseñanza en NutriciOn y Salud	Adriana Godínez; Claudio Soto-Garita; Estela Cordero; Francisco Duarte; Hebleen Porras; Jose Luis Vargas; Joselyn Prado & Ivanna Krize-Morún; Mariela Gutierrez; Melany CalderOn
EPI_ISL_3037842	HOSPITAL LA ANEXION [NICOYA/GUANACASTE]	Inciensa, Instituto Costarricense de Investigación y Enseñanza en Nutrición y Salud	Adriana Godínez; Caterina Guzmán; Claudio Soto-Garita; Estela Cordero; Francisco Duarte; Hebleen Porras; José Luis Vargas; Mariela Gutiérrez Joselyn Prado; Melany Calderón; Nazareth Ruiz & Arnoldo Zamora
EPI_ISL_2657682, EPI_ISL_3037844	HOSPITAL METROPOLITANO	Inciensa, Instituto Costarricense de Investigación y Enseñanza en Nutrición y Salud	Adriana Godínez; Caterina Guzmán; Claudio Soto-Garita; Estela Cordero; Francisco Duarte; Hebleen Porras; Joselyn Prado; José Luis Vargas; Mariela Gutiérrez; Mariela Gutiérrez Joselyn Prado; Melany Calderón; Nazareth Ruiz & Margarita Lee; Nazareth Ruiz & Margarita Lee-Lui
EPI_ISL_2502746	HOSPITAL MEXICO	Inciensa, Instituto Costarricense de InvestigaciOn y Enseñanza en NutriciOn y Salud	Adriana Godínez; Claudio Soto-Garita; Estela Cordero; Francisco Duarte; Hebleen Porras; Jose Luis Vargas; Joselyn Prado & Juan Vilallobos; Mariela Gutierrez; Melany CalderOn
EPI_ISL_1827515, EPI_ISL_2658274	HOSPITAL MONSEÑOR SANABRIA	Inciensa, Instituto Costarricense de Investigación y Enseñanza en Nutrición y Salud	Adriana Godínez; Claudio Soto-Garita; Estela Cordero; Francisco Duarte; Hebleen Porras; Joselyn Prado & Andrea Moreno-Carvajal; Joselyn Prado & Javier Zárate-León; José Luis Vargas; Mariela Gutiérrez; Melany Calderón
EPI_ISL_1261540	HOSPITAL RAFAEL MENDEZ	Instituto de Salud Carlos III	A. Monzón; E; F. Casas; I. Jiménez; I.Cascales; M. Sandonís; P. Zaballos; S. Cuesta; S. Iglesias-Caballero; S. Pozo; S. Varona; V. Camarero; Vázquez-Morón
EPI_ISL_2674346	HOSPITAL REGIONAL DE LA ORINOQUIA	Instituto Nacional de Salud- Dirección de Investigación en Salud Pública	Carlos Franco-Muñoz; Carmen Osorio; Diana Malo; Diego A. Álvarez-Díaz; Diego Andrés Prada; Gerardo Santamaría; Hector Alejandro Ruiz-Moreno; Jhonnatan Reales-González; Jorge Rivera; Juan Camilo Martinez; Julian Naizaque; Katherine Laiton-Donato; Lisseth Pardo; Magdalena Wiesner; Marcela Mercado-Reyes; Maria T. Herrera-Sepúlveda; Marta Lopez Blanco; Martha Lucia Ospina Martinez; Paola Rojas; Sergio Gomez; Sheryll Corchuelo; Ángela Alarcon Cruz
EPI_ISL_2502741	HOSPITAL SAN FRANCISCO DE ASIS	Inciensa, Instituto Costarricense de InvestigaciOn y Enseñanza en NutriciOn y Salud	Adriana Godínez; Claudio Soto-Garita; Estela Cordero; Francisco Duarte; Hebleen Porras; Jose Luis Vargas; Joselyn Prado & Adrián Fallas-Mora; Mariela Gutierrez; Melany CalderOn
EPI_ISL_2103406, EPI_ISL_2658278	HOSPITAL SAN FRANCISCO DE ASIS	Inciensa, Instituto Costarricense de Investigación y Enseñanza en Nutrición y Salud	Adriana Godínez; Caterina Guzmán; Claudio Soto-Garita; Estela Cordero; Francisco Duarte; Hebleen Porras; Joselyn Prado & Juan Carlos Villalobos-Ugalde; José Luis Vargas; Mariela Gutiérrez; Melany Calderón; Nazareth Ruiz & Joselyn Prado
EPI_ISL_2502753, EPI_ISL_2502760, EPI_ISL_2502761,	HOSPITAL SAN JUAN DE DIOS	Inciensa, Instituto Costarricense de InvestigaciOn y Enseñanza en NutriciOn y Salud	Adriana Godínez; Claudio Soto-Garita; Estela Cordero; Francisco Duarte; Hebleen Porras; Jose Luis Vargas; Joselyn Prado & Marco Cháves-Otárola; Mariela Gutierrez; Melany CalderOn

EPI_ISL_2502762			
EPI_ISL_682245, EPI_ISL_2657687, EPI_ISL_2827974	HOSPITAL SAN JUAN DE DIOS	Inciensa, Instituto Costarricense de Investigación y Enseñanza en Nutrición y Salud	Adriana Godinez & Melany Calderon; Adriana Godínez; Claudio Soto-Garita; Estela Cordero; Francisco Duarte; Hebleen Porras; Joselyn Prado & Marco Cháves-Otárola; Joselyn Prado & Paola Sánchez; José Luis Vargas; Mariela Gutiérrez; Melany Calderón
EPI_ISL_1510697, EPI_ISL_2331700	HOSPITAL UNIVERSITARIO INSULAR DE GRAN CANARIA	Instituto de Salud Carlos III	A. Monzón; ARACELI; CRISTOBAL; F. Casas; I. Jiménez; I.DEL ROSARIO QUINTANA; I.HERNANDEZ BETANCOR; M. Sandonís; P. Zaballos; S. Cuesta; S. Iglesias-Caballero; S. Pozo; S. Varona; V. Camarero; Vázquez-Morón
EPI_ISL_682265	HOSPITAL UPALA	Inciensa, Instituto Costarricense de Investigación y Enseñanza en Nutrición y Salud	Adriana Godinez & Melany Calderon; Claudio Soto-Garita; Estela Cordero; Francisco Duarte; Hebleen Porras
EPI_ISL_636975	HP Pemba	KRISP, KZN Research Innovation and Sequencing Platform	Giandhari J; Ismael N; Nadia Sitoe; Nedio Mabunda; Paulo Arnaldo; Pillay S; Tegally H; Wilkinson E; de Oliveira T
EPI_ISL_412971	HUS Diagnostiikkakeskus, Hallinto	Department of Virology Faculty of Medicine, Medicum University of Helsinki	Hannimari Kallio-Kokko; Olli Vapalahti; Suvi Kuivanen; Teemu Smura
EPI_ISL_2193710	Hackensack Medical Center	New York Genome Center	Andre Corvelo; Barry Kreiswirth; David Perlin; Dayna M. Oschwald; Jose Mediavilla; Kaelea Composto; Kar Chow; Liang Chen; Marcus Cunningham; Michael Zody; Samantha Fennessey; Tom Maniatis
EPI_ISL_2096776, EPI_ISL_2096885	Hadassah Medical Center Clinical Virology Laboratory, Hadassah Ein Kerem	Hadassah Hebrew University Viral Sequencing Group, Hadassah Hebrew University Medical Center	Dana G. Wolf; Esther Oiknine-Djian; Hadar Golan Berman; Mila Rivkin; Sheera Adar
EPI_ISL_615105	Halmstad klinisk mikrobiologi	The Public Health Agency of Sweden	Anna Risberg; Anna-Malin Linde; Karin Tegmark-Wisell; Maria Lind Karlberg; Mattias Haukland; Mia Brytting; Olov Svartstrom; Oskar Karlsson Lindsjo; Petra Edquist; Reza Advani; Sandra Broddesson
EPI_ISL_2333091	Haugesund Hospital, laboratory for Medical Microbiology	Norwegian Institute of Public Health, Department of Virology	Atiya R Ali; Debech Nadia; Engebretsen Serina Beate; Garcia Llorente Ignacio; Hilde Elshaug; Hilde Vollan; Jon Bråte; Kamilla Heddeland Instefjord; Karoline Bragstad; Kathrine Stene-Johansen; Line Victoria Moen; Marie Paulsen Madsen; Olav Hungnes; Pedersen Benedikte Nevjen; Rasmus Riis Kopperud
EPI_ISL_1318065, EPI_ISL_2333443	Haukeland University Hospital, Dept. of Microbiology	Norwegian Institute of Public Health, Department of Virology	'Kathrine Stene-Johansen; Atiya R Ali; Debech Nadia; Engebretsen Serina Beate; Garcia Llorente Ignacio; Hilde Elshaug; Hilde Vollan; Jon Bråte; Kamilla Heddeland Instefjord; Karoline Bragstad; Kathrine Stene-Johansen; Line Victoria Moen; Marie Paulsen Madsen; Olav Hungnes; Pedersen Benedikte Nevjen; Rasmus Riis Kopperud
EPI_ISL_2938119	Health Center Novi Grad Sarajevo	Clinical Center, University of Sarajevo; Unit for Clinical Microbiology	Amela Dedeić-Ljubović; Edina Zahirović; Irma Salimović-Bešić; Sandra Vegar-Zubović; Sebija Izetbegović
EPI_ISL_2938097	Health Center Velika Kladuša	Clinical Center, University of Sarajevo; Unit for Clinical Microbiology	Amela Dedeić-Ljubović; Edina Zahirović; Irma Salimović-Bešić; Sandra Vegar-Zubović; Sebija Izetbegović
EPI_ISL_2080427, EPI_ISL_2469286	Health Services Laboratories	Wellcome Sanger Institute for the COVID-19 Genomics UK (COG-UK) Consortium	Cordelia Langford; David K. Jackson; Dominic Kwiatkowski; Ewan Harrison; Health Services Laboratories and Alex Alderton; Ian Johnston; Jeffrey Barrett; John Sillitoe on behalf of the Wellcome Sanger Institute COVID-19 Surveillance Team; Roberto Amato; Sonia Goncalves
EPI_ISL_2004109, EPI_ISL_2004111, EPI_ISL_2689834, EPI_ISL_2712906	Health sciences Department, Universidad Técnica Particular de Loja	Institute of Microbiology, Universidad San Francisco de Quito	Belén Prado-Vivar; Bernardo Gutiérrez; Danilo Sosa; Fernanda Zurita; Fernando Serrano; Gabriel Trueba; Juan José Guadalupe; Katty Ojeda; Michelle Grunauer; Monica Becerra-Wong; Paola Dalgo; Patricio Rojas-Silva; Paúl Cárdenas; Rita Rodriguez; Rommel Guevara; Sully Márquez; Verónica Barragán
EPI_ISL_796015	Hebei Provincial Center for Disease Control and Prevention, Shijiazhuang, Hebei Province; National Institute for Viral Disease Control and Prevention, China CDC	Hebei Provincial Center for Disease Control and Prevention, Shijiazhuang, Hebei Province; National Institute for Viral Disease Control and Prevention, China CDC	George F. Gao; Nankun Liu; Qi Li; Shunxiang Qi; Wenbo Xu; Xiang Zhao; Yang Song
EPI_ISL_640018	Heidelberg Clinic wc HBC	NHLS/UCT	Arash Iranzadeh; Bruna Galvao; Carolyn Williamson; Deelan Doolabh; Diana Hardie; Innocent Mudau; Kruger Marais; Lynn Tyers; Marvin Hsiao; Stephen Korsman
EPI_ISL_700598, EPI_ISL_2877001	Heideveld Emergency Centre	NHLS/UCT	Arash Iranzadeh; Bruna Galvao; Carolyn Williamson; Deelan Doolabh; Diana Hardie; Gert Marais; Innocent Mudau; Kruger Marais; Lynn Tyers; Marvin Hsiao; Stephen Korsman
EPI_ISL_1088823	Helix / Illumina	Respiratory Viruses Branch, Division of Viral Diseases, Centers for Disease Control and Prevention	Alexandre Bolze; Ary Ascencio; Ben L. Rambo-Martin; Brad Sickler; Charlotte Rivera-Garcia; Christine Tran; Clinton R. Paden; Dakota Howard; David Becker; Dhwani Batra; Duncan MacCannell; Efren Sandoval; Eileen de Feo; Elizabeth Cirulli; Eric Allen; Geraint Levan; James Lu; Jan Antico; Jason Nguyen; Jimmy Ramirez; Jingtao Liu; Kelly Schiabor Barrett; Kim Gietzen; Magnus Isaksson; Marc Laurent; Matthew Tolentino; Nicole L. Washington; Peter W. Cook; Phil Febbo; Ryan Cho; Shannon Wickline; Sherry Wang; Simon White; Summer Galloway; Suxiang Tong; Tyler Cassens; William Lee
EPI_ISL_1269377, EPI_ISL_1270167, EPI_ISL_1443915, EPI_ISL_1924266, EPI_ISL_2159579, EPI_ISL_2268352, EPI_ISL_2269936, EPI_ISL_2269998, EPI_ISL_2320596, EPI_ISL_2598842			
see above	Helix/Illumina	Centers for Disease Control and Prevention Division of Viral Diseases, Pathogen Discovery	Adrian Paskey; Alexandre Bolze; Ary Ascencio; Ben L. Rambo-Martin; Benjamin Rambo-Martin; Brad Sickler; Charlotte Rivera-Garcia; Christine Tran; Christopher Gulvick; Clinton R. Paden; Dakota Howard; Darlene Wagner; David Becker; Dhwani Batra; Duncan MacCannell; Efren Sandoval; Eileen de Feo; Elizabeth Cirulli; Eric Allen; Geraint Levan; James Lu; Jan Antico; Jason Caravas; Jason Nguyen; Jimmy Ramirez; Jingtao Liu; Kara Moser; Kelly Schiabor Barrett; Kim Gietzen; Magnus Isaksson; Marc Laurent; Matthew Schmerer; Matthew Tolentino; Nicole L. Washington; Peter W. Cook; Phil Febbo; Ryan Cho; Scott Sammons; Shannon Wickline; Shatavia Morrison; Sherry Wang; Simon White; Summer Galloway; Suxiang Tong; Tyler Cassens; William Lee; Yvette Unoarumhi
EPI_ISL_1036688, EPI_ISL_1036697	Helix/Illumina	Respiratory Viruses Branch, Division of Viral Diseases, Centers for Disease Control and Prevention	Alexandre Bolze; Ary Ascencio; Ben L. Rambo-Martin; Brad Sickler; Charlotte Rivera-Garcia; Christine Tran; Clinton R. Paden; Dakota Howard; David Becker; Dhwani Batra; Duncan MacCannell; Efren Sandoval; Eileen de Feo; Elizabeth Cirulli; Eric Allen; Geraint Levan; James Lu; Jan Antico; Jason Nguyen; Jimmy Ramirez; Jingtao Liu; Kelly Schiabor Barrett; Kim Gietzen; Magnus Isaksson; Marc Laurent; Matthew Tolentino; Nicole L. Washington; Peter W. Cook; Phil Febbo; Ryan Cho; Shannon Wickline; Sherry Wang; Simon White; Summer Galloway; Suxiang Tong; Tyler Cassens; William Lee
EPI_ISL_2367588	Hellenic National Blood Transfusion Center - EKEA	Greek Genome Center, Biomedical Research Foundation of the Academy of Athens (BRFAA)	Dimitrios Thanos; Efthimia Petinaki; Emmanouil Athanasiadis; Giannis Vatsellas; Katerina Zoi; Kostas Stamoulis; Theodoros Loupis
EPI_ISL_2617410	Hellenic National Blood Transfusion Center - EKEA	Greek Genome Center, Biomedical Research Foundation of the Academy of Athens (BRFAA)	Dimitrios Thanos; Efthimia Petinaki; Emmanouil Athanasiadis; Giannis Vatsellas; Katerina Zoi; Kostas Stamoulis; Theodoros Loupis
EPI_ISL_501235	Hellenic Pasteur Institute, National Influenza Reference laboratory of Southern Greece & Unit of Bioinformatics and Applied Genomics	Hellenic Pasteur Institute, National Influenza Reference laboratory of Southern Greece & Unit of Bioinformatics and Applied Genomics	Andreas Mentis; Androniki Voulgari-Kokota; Antonios Kalliaropoulos; Aspasia Kontou; Athanasios Kossyvakis; Evangelidou Maria; Horefti Elina; Timokratis Karamitros; Vasiliki Pogka
EPI_ISL_430469	Hellenic Pasteur Institute, Public Health Laboratories	Hellenic Pasteur Institute, Public Health Laboratories, Unit of Bioinformatics and Applied Genomics	Andreas Mentis; Androniki Voulgari-Kokota; Antonios Kalliaropoulos; Aspasia Kontou; Athanasios Kossyvakis; Evangelidou Maria; Horefti Elina; Timokratis Karamitros; Vasiliki Pogka
EPI_ISL_450529	Hematology Laboratory, Section of Molecular Diagnostics, University Clinical Centre, Medical University of Gdansk	Department of Virology, Faculty of Medicine, University of Helsinki, Helsinki, Finland	Aneta Szulc; Maciej Grzybek; Marlena Robakowska; Olli Vapalahti; Teemu Smura
EPI_ISL_451654, EPI_ISL_906752	Hematology Laboratory, Section of Molecular Diagnostics, University Clinical Centre, Medical University of Gdansk	Laboratory of Recombinant Vaccines	Adam Sodol; Aneta Szulc; Boguslaw Szewczyk; Ewa Milosz; Krystyna Bienkowska-Szewczyk; Krzysztof Lewandowski; Lukasz Rabalski; Maciej Grzybek; Maciej Kosinski; Marlena Robakowska
EPI_ISL_700262	Hematopathology Laboratory, ACTREC, TMC	Hematopathology Laboratory, ACTREC, TMC	ACTREC; Hematopathology Laboratory
EPI_ISL_2140721	Hillside Clinic wc BFW	NHLS/UCT	Arash Iranzadeh; Bruna Galvao; Carolyn Williamson; Deelan Doolabh; Diana Hardie; Innocent Mudau; Kruger Marais; Lynn Tyers; Marvin Hsiao; Stephen Korsman
EPI_ISL_2812851, EPI_ISL_2828084, EPI_ISL_3015123	Histopath	NSW Health Pathology - Institute of Clinical Pathology and Medical Research; Westmead Hospital; University of Sydney	CIDM-PH et al.
EPI_ISL_2779330, EPI_ISL_2779346	Homabay County Referral Hospital	USAMRD-A, Basic Science Laboratory	Alan Lemtudo; Beth Mutai; Brian Andika; Carol Kifude; Clement Masakwe; Eric Muthanje; Esther Omuseni; Faith Sigei; Gathii Kimita; George Awinda; John Waitumbi; Josphat Nyataya; Rachel Githii; Rehema Liyai; Stephen Ochola
EPI_ISL_1257882	Hopital Général de Douala	Institut Pasteur de Dakar	Dia Ndongo; Diagne Moussa Moïse; Diallo Amadou; Diop Mamadou; Elisabeth Carniel; Faye Ousmane; Loucoubar Cheikh; Njouom Richard; Sall Amadou Alpha; Sankhe Safietou
EPI_ISL_1257892	Hopital Laquintinie de Douala	Institut Pasteur de Dakar	Dia Ndongo; Diagne Moussa Moïse; Diallo Amadou; Diop Mamadou; Elisabeth Carniel; Faye Ousmane; Loucoubar Cheikh; Njouom Richard; Sall Amadou Alpha; Sankhe Safietou
EPI_ISL_1821607, EPI_ISL_2029952, EPI_ISL_2029977, EPI_ISL_2188319, EPI_ISL_2188320, EPI_ISL_2293517, EPI_ISL_2374409, EPI_ISL_2462905, EPI_ISL_2462927, EPI_ISL_2464242, EPI_ISL_2628279, EPI_ISL_2757663, EPI_ISL_2757673, EPI_ISL_2757678, EPI_ISL_2790847, EPI_ISL_2835879, EPI_ISL_2835906, EPI_ISL_2835907, EPI_ISL_3031652, EPI_ISL_3031654, EPI_ISL_3031655			
see above	Hospital	National Reference Center for Viruses of Respiratory Infections, Institut Pasteur, Paris	Alexandra Ducancelle; Angela Brisebarre; Antoine Talarmin; Camille Capel; Ch De Cornouaille; Christophe Malabat; CléMence Guillaume; Corinne Maufrais; Damien Mornico; Didier Mattera; Emmanuelle Permal; Etienne Simon-Lorière; Frédéric Lemoine; Hub de Bioinformatique et Biostatistique; JéRôMe Durivault; L. Courdavault; Louise Lefrançois; LéA Pilorge; Marianne Coste-Burel; Marianne Burgard; Marion Barbet; Maud Vanpeene; Méline Bizard; Patricia Barbut; Sylvaine Bastian; Sylvie Behillil; Sylvie Van der Werf; Victoire Baillet; Vincent Enouf
EPI_ISL_1917304	Hospital Center Emile Mayrisch	Laboratoire national de sante, Microbiology, Microbial Genomics Platform	Anke Wienecke-Baldacchino; Catherine Ragimbeau; Cynthia Oxacelay; Fatu Djabi; Jessica Tapp; Lise Pignon; Raoul Salmon; Tamir Abdelrahman
EPI_ISL_770028	Hospital Dr. Raul Blanco Cervantes	Inciensa, Instituto Costarricense de Investigación y Enseñanza en Nutrición y Salud	Adriana Godínez; Claudio Soto-Garita; Estela Cordero; Francisco Duarte; Hebleen Porras; Melany Calderón & Mariel López
EPI_ISL_2965560, EPI_ISL_2965569, EPI_ISL_2965583, EPI_ISL_2965584	Hospital Español	Centro de Innovación en Vigilancia Epidemiológica (CiVE), Institut Pasteur Montevideo, Uruguay	Alicia Costábile; Alvaro Fajardo; Ana Moller; Andrés Lizasoain; Belén González; Bernardina Rivera; Cecilia Alonso; Cecilia Salazar; Gonzalo Bello; Gonzalo Moratorio; Gregorio Iraola; Henry Albornoz; Ignacio Ferrés; Javier Hurtado; Juan Zanetti; Julio Medina; Luciana Griffero; Lucía Spangenberg; Ma Noel Bentancor; Ma Pía Techera; Mailen Arleo; Martina Alonso; Matías Maidana; Mauricio Méndez; Melissa Duquía; Mercedes Paz; Natalia Rego; Natalia Reyes; Nicolas Nin; Odhille Chappos; Paula Perbolianachis; Pilar Moreno; Rodney Colina; Rodrigo Arce; Tamara Fernández-Calero; Tania Possi; Veronica Noya; Viviana Bortagaray
EPI_ISL_1647957, EPI_ISL_2483973	Hospital General Universitario Gregorio Marañón	Hospital General Universitario Gregorio Marañón	Cristina Rodriguez-Grande; Darío García de Viedma; Julia Suárez; Laura Pérez-Lago; Marta Herranz Martin; Patricia Muñoz; Pedro Sola Campoy; Pilar Catalán; Sergio Buenestado Serrano; Victor Manuel de la Cueva
EPI_ISL_467234, EPI_ISL_541903, EPI_ISL_780044, EPI_ISL_780047	Hospital General Universitario Gregorio Marañón	SeqCOVID-SPAIN consortium/IBV(CSIC)	Darío García de Viedma; Darío García de Viedma and SeqCOVID-SPAIN consortium; Jon Sicilia; Julia Suárez; Laura Pérez-Lago; Marta Herranz; Patricia Muñoz; Patricia Muñoz and SeqCOVID-SPAIN consortium; Pilar Catalán
EPI_ISL_2014355	Hospital Herrera Llerandi	Asociación de Salud Integral/Clínica Familiar Luis Ángel García	Ana S. Gonzalez-Reiche; Claudia Rangel; Danicela Mercado; Eduardo Arathoon; Hilda Ruiz; Luis Aguirre; Luis Rivas; Narda Medina; Oscar Bonilla; Osmar Gamboa.
EPI_ISL_769992	Hospital Metropolitano	Inciensa, Instituto Costarricense de Investigación y Enseñanza en Nutrición y Salud	Adriana Godínez; Claudio Soto-Garita; Estela Cordero; Francisco Duarte; Hebleen Porras; Melany Calderón & Margarita Lee Lui
EPI_ISL_480327	Hospital Nacional de Niños	Charité Virology-University of Costa Rica	Andrei Montero Bonilla; Andres Moreira-Soto; Cristian Pérez Corrales; Eugenia Corrales-Aguilar; Ignacio Postigo-Hidalgo; Jan Felix Drexler

EPI_ISL_471270	Hospital Oncológico Solca Núcleo de Quito	Institute of Microbiology, Universidad San Francisco de Quito	Belén Prado-Vivar; Bernardo Gutiérrez; Gabriel Trueba; Grace Salazar; Juan José Guadalupe; Marcos Di Stefano; Michelle Grunauer; Patricio Rojas-Silva; Paúl Cárdenas; Sully Márquez; Verónica Barragán
EPI_ISL_524463	Hospital Regional de Cotia	Instituto Adolfo Lutz, Interdiciplinary Procedures Center, Strategic Laboratory	Claudia Regina Gonçalves; Claudio Tavares Sacchi; Erica Valessa Ramos Gomes
EPI_ISL_491452	Hospital San Rafael de Alajuela	Inciensa, Instituto Costarricense de Investigación y Enseñanza en Nutrición y Salud	Adriana Godinez & Melany Calderon; Claudio Soto-Garita; Estela Cordero; Francisco Duarte; Hebleen Brenes
EPI_ISL_2982423	Hospital Universitari Arnau de Vilanova	Hospital Universitari Vall d'Hebron - Vall d'Hebron Institut de Recerca	Alejandra González-Sánchez; Andrés Antón; Ariadna Rando; Carla Castillo; Cristina Andrés; Damir Garcia-Cehic; Josep Quer; Juliana Esperalba; Karen García; Maria Carmen Martin; Maria Gema Codina; Maria Piñana; Rodrigo Vásquez; Tomàs Pumarola
EPI_ISL_2516574	Hospital Universitari Bellvitge	Microbiology Department	Aida Gonzalez-Diaz; Carmen Ardanuy; Jordi Camara; Jordi Niubó; Laura Calatayud; M Angeles Domínguez; Miguel Fernandez-Huerta; Sara Marti
EPI_ISL_3009987	Hospital Universitari Vall d'Hebron - Vall d'Hebron Institut de Recerca	Hospital Universitari Vall d'Hebron - Vall d'Hebron Institut de Recerca	Alejandra González-Sánchez; Andrés Antón; Ariadna Rando; Carla Castillo; Cristina Andrés; Damir Garcia-Cehic; Josep Quer; Juliana Esperalba; Karen García; Maria Carmen Martin; Maria Gema Codina; Maria Piñana; Rodrigo Vásquez; Tomàs Pumarola
EPI_ISL_691687	Hospital Universitario de Ceuta	Instituto de Salud Carlos III	A. Monzón; F. Casas; I. Hijano, S.; I. Jiménez; Iglesias-Caballero; M. Camarero; M. Cuesta; M. González-Esguevillas; M. Pozo; M. Zaballos; P. Jiménez; S. Juliá; S. Molinero Calamita; S. Varona
EPI_ISL_1470312	Hospital Universitario de Gran Canaria Dr. Negrín	SeqCOVID-SPAIN consortium/IBV(CSIC)	Ana Bordes Benítez and SeqCOVID-SPAIN consortium; Francisco J. Chamizo López; M. Carmen Pérez González
EPI_ISL_455314	Hospital Virgen del Rocio	Instituto de Salud Carlos III	A. Monzón; F. Casas; I; I. Jiménez; Iglesias-Caballero; J. Lepe; M. Camarero; M. Cuesta; M. González-Esguevillas; M. Molinero Calamita; M. Zaballos; P. Jiménez; S. Juliá; S. Pozo; S. Varona
EPI_ISL_671815	Hospital de la Santa Creu i Sant Pau. Servicio de Microbiología	SeqCOVID-SPAIN consortium/IBV(CSIC)	Elisenda Miró and SeqCOVID-SPAIN consortium; Ferran Navarro; Núria Rabella
EPI_ISL_458028	Hospital for Tropical Diseases	COVID-19 Network Investigations (CONI) Alliance	Akanitt Jittmittraphap; Angkana Huang; Anthony R. Jones; Arporn Wangwiwatsin; Bhakbhoom Panthan; Chonticha Klungtong; Ekawat Pasomsub; Elizabeth Batty; Insee Sensorn; Janjira Thaipadungpanit; Khajohn Joonsalak; Kingkan Rakmanee; Krittikorn Kumpornsin; Namfon Kotanan; Nantarat Chantawat; Pornsawan Leaungwutiwong; Stefan Fernandez; Thanat Chookajorn; Theerarat Kochakarn; Treewat Watthanachockchai; Wasun Chantratita; Weena Janwitthayanan; Wudtichai Manasatienkij
EPI_ISL_2788566, EPI_ISL_2788595	Hospital of Southern Norway - Kristiansand, Department of Medical Microbiology	Norwegian Institute of Public Health, Department of Virology	Atiya R Ali; Debech Nadia; Engebretsen Serina Beate; Garcia Llorente Ignacio; Hilde Elshaug; Hilde Vollan; Jon Bråte; Kamilla Heddeland Instefjord; Karoline Bragstad; Kathrine Stene-Johansen; Line Victoria Moen; Marie Paulsen Madsen; Olav Hungnes; Pedersen Benedikte Nevjen; Rasmus Riis Kopperud
EPI_ISL_1077280, EPI_ISL_2219818	Houston Methodist Hospital	Houston Methodist Hospital	Ilya J. Finkelstein; James J. Davis; Jessica Cambric; Jimmy Gollihar; Kristina Reppond; Layne Pruitt; Madison N. Shyer; Marcus Nguyen; Matthew Ojeda Saavedra; Paul A. Christensen; Prasanti Yerramilli; Randall J. Olsen; Robert Olson; Ryan Gadd; S. Wesley Long; Sishir Subedi; and James M. Musser
EPI_ISL_475525	Huddinge VC	The Public Health Agency of Sweden	Anna Risberg; Anna-Malin Linde; Karin Tegmark-Wisell; Maria Lind Karlberg; Mattias Haukland; Mia Brytting; Olov Svartstrom; Oskar Karlsson Lindsjo; Reza Advani; Sandra Broddesson
EPI_ISL_2466657	Human Genetic Research Center, Kawsar Biotech Company	Kawsar Biotech Company	Abbasalipour, M.; Azadmanesh, K.; Chamran Hospital, C.; Khosravi; M.A.; National Institute for Medical Research Development, N.; Zeinali, R.; Zeinali, S.
EPI_ISL_645082	Human Genome Variation Research Group, Malopolska Centre of Biotechnology	Human Genome Variation Research Group, Malopolska Centre of Biotechnology	Botwina, P.; Branicki, W.; Dabrowska, A.; Foremny, J.; Gromowski, T.; Klajmon, A.; Kopera, K.; Kowalski, M.; Labaj; Marszalek, K.; Owczarek, K.; P.P.; Pisarek, A.; Pospiech, E.; Pyrc, K.; Sanak, M.; Swadzba, J.; Szczepanski, A.
EPI_ISL_526224, EPI_ISL_526225, EPI_ISL_1041201	Hungarian Defence Forces Military Medical Centre	National Laboratory of Virology, Szentágothai Research Centre	Balázs Somogyi; Bálint Eszenyi; Csaba Pereszlényi; Endre Gábor Tóth; Ferenc Jakab; Gábor Kemenesi; Ágnes Balázs-Nagy
EPI_ISL_1296394	Hôpital Bichat Claude Bernard, Laboratoire de Virologie	IAME UMR1137 Inserm, Université de Paris, Hôpital Bichat	Alexandre Storto; Amélie Recoing; Antoine Bridier-Nahmias; Benoit Visseaux; Charlotte Charpentier; Diane Descamps; Gilles Collin; Lena Daniel; Mélanie Bertine; Nadhira Houhou-Fidouh; Quentin Le Hingrat; Siham Hamri
EPI_ISL_2637514	Hôpital Cochin	Department of Virology, Henri Mondor University Hospital, Assistance Publique Hôpitaux de Paris, Université Paris-Est Créteil, INSERM U955	Alexandre Soulier; Christophe Rodriguez; Elisabeth Trawinski; Guillaume Gricourt; Jean-Michel Pawlotsky; Melissa N'Debi; Slim Fourati; Vanessa Demontant
EPI_ISL_710532	Hôpital Fattouma-Bourguiba de Monastir	Laboratoire des Procédés de Criblage Moléculaire et Cellulaire-Centre de Biotechnologie de Sfax	A. and Masmoudi, S.; Abdelmoulah, F.; Abid, N.; Ajili, F.; Aouni, M.; Ben Ayed, I.; Bensaid, M.; Chtourou, A.; Elargoubi, A.; Fki-berrajah, L.; Gaaloul, I.; Gargouri, S.; Hammami, A.; Kamoun, S.; Karray Hakim, H.; Kharat, N.; Mastouri, M.; Mhalla, S.; Nabli, A.; Rebai; Smeti, I.; Souissi, A.; Stambouli, N.; Turki, M.
EPI_ISL_961579	Hôpital Georges L. Dumont	National Microbiology Laboratory (NML)	Anna Majer; Anneliese Landgraff; CanCOGeN's metadata curation team; Darian Hole; Elsie Grudeski; Gary Van Domselaar; Grace Seo; Guillaume Desnoyers; Jennifer Tanner; Kirsten Biggar; Madison Chapel; Morag Graham; Natalie Knox; Nathalie Bastien; Philip Mabon; Public Health Agency of Canada CanCOGeN team; Rhiannon Huzarewich; Richard Garceau; Russell Mandes; Shari Tyson; Timothy Booth; Yan Li
EPI_ISL_535716	Hôpital de Verdun	Laboratoire de santé publique du Québec	Guillaume Bourque; Ioannis Ragoussis; Jesse Shapiro; Mark Lathrop and Michel Roger on behalf of the CoVSeQ research group; Sandrine Moreira
EPI_ISL_2384050, EPI_ISL_2384052	IA State Hygienic Laboratory	Centers for Disease Control and Prevention Division of Viral Diseases, Pathogen Discovery	Alison Laufer Halpin; Ben L. Rambo-Martin; Clinton R. Paden; Dakota Howard; Darlene Wagner; Dave Wentworth; Dhwani Batra; Jasmine Padilla; Justin Lee; Katie Dillon; Krista Queen; Kristen Knipe; Kristine Lacek; Mark Burroughs; Matthew Schmerer; Mili Sheth; Peter Cook; Sam Shepard; Sarah Nobles; Shoshona Le; Suxiang Tong; Vivien Dugan; Yvette Unoarumhi
EPI_ISL_2840309	ID Bureau of Laboratories	Centers for Disease Control and Prevention Division of Viral Diseases, Pathogen Discovery	Alison Laufer Halpin; Ben L. Rambo-Martin; Clinton R. Paden; Dakota Howard; Darlene Wagner; Dave Wentworth; Dhwani Batra; Jasmine Padilla; Justin Lee; Katie Dillon; Krista Queen; Kristen Knipe; Kristine Lacek; Mark Burroughs; Matthew Schmerer; Mili Sheth; Peter Cook; Sam Shepard; Sarah Nobles; Shoshona Le; Suxiang Tong; Vivien Dugan; Yvette Unoarumhi
EPI_ISL_903753	ID Bureau of Laboratories	Genomics and Discovery, Respiratory Viruses Branch, Division of Viral Diseases, Centers for Disease Control and Prevention	Anna Montmayeur; Anna Uehara; Ben L. Rambo-Martin; Clinton R. Paden; Dhwani Batra; Haibin Wang; Jasmine Padilla; Jing Zhang; Justin Lee; Krista Queen; Lori Rowe; Mark Burroughs; Mili Sheth; Peter W. Cook; Rachel Marine; Sarah Nobles; Suxiang Tong; Yan Li; Ying Tao
EPI_ISL_2234876, EPI_ISL_2444806, EPI_ISL_2444813, EPI_ISL_2444814, EPI_ISL_2444815, EPI_ISL_2444817, EPI_ISL_2444819, EPI_ISL_2444820, EPI_ISL_2444821, EPI_ISL_2444822, EPI_ISL_2444823, EPI_ISL_2444825, EPI_ISL_2444827, EPI_ISL_2444829, EPI_ISL_2444830, EPI_ISL_2444834, EPI_ISL_2444836, EPI_ISL_2444838, EPI_ISL_2444839			
see above	IICS-UNA	IICS-UNA	Adriana Valenzuela; Alejandra Rojas; Chyntia Diaz; Eva Nara; Fatima Cardozo; Florencia del Puerto; Joel Ortiz; Jonas Fernandez; Laura Franco; Laura Mendoza; Leticia Rojas; Magaly Martinez; Maria Eugenia Galeano.
EPI_ISL_2758180	ILV Kärnten	Bergthaler laboratory, CeMM Research Center for Molecular Medicine of the Austrian Academy of Sciences	Andreas Bergthaler; Anna Schedl; Bekir Erguner; Benedikt Agerer; Christoph Bock; Fabian Amman; Jan Laine; Lukas Endler; Maelle Le Moing; Martin Senekowitsch; Matthew Thornton; Michael Schuster; Petr Triska; Thomas Penz
EPI_ISL_2827770, EPI_ISL_2827774, EPI_ISL_2827780, EPI_ISL_2827784	IMAT S.A.S.	Instituto Nacional de Salud- Dirección de Investigación en Salud Pública	Carlos Franco-Muñoz; Carmen Osorio; Diana Malo; Diego A. Álvarez-Díaz; Diego Andrés Prada; Gerardo Santamaría; Hector Alejandro Ruiz-Moreno; Jhonnatan Reales-González; Jorge Rivera; Juan Camilo Martinez; Julian Naizaque; Katherine Laiton-Donato; Lisseth Pardo; Magdalena Wiesner; Marcela Mercado-Reyes; Maria T. Herrera-Sepúlveda; Marta Lopez Blanco; Martha Lucia Ospina Martinez; Paola Rojas; Sergio Gomez; Sheryll Corchuelo; Ángela Alarcon Cruz
EPI_ISL_1439879, EPI_ISL_2845684	IMD - Labor Oderland	Robert Koch Institute	
EPI_ISL_1722245	IMD - Medizinisches Labor Rostock	Robert Koch Institute	
EPI_ISL_1721083	IMD Labor Frankfurt	Robert Koch Institute	
EPI_ISL_2657686	INCIENSA	Inciensa, Instituto Costarricense de Investigación y Enseñanza en Nutrición y Salud	Adriana Godínez; Claudio Soto-Garita; Estela Cordero; Francisco Duarte; Hebleen Porras; Joselyn Prado & Grettel Chanto-Chacón; José Luis Vargas; Mariela Gutiérrez; Melany Calderón
EPI_ISL_2319343	INDRE	Instituto Nacional de Medicina Genomica	Cedro-Tanda A; Garcia-Cardenas FJ; Gisela Barrera-Badillo; Gonzalez-Barrera D; Gonzalez-Woge MA; Herrera-Montalvo LA.; Hidalgo-Miranda A; Irma Lopez-Martinez; Jose Ernesto Ramirez González; Mendoza-Vargas A; Munguia-Garza P; Rangel-DeLeon D; Reyes-Grajeda JP
EPI_ISL_1577752, EPI_ISL_1577815, EPI_ISL_2628208, EPI_ISL_2628297, EPI_ISL_2628307	INHRR	Laboratorio de Virología Molecular	Aguilar M; Alarcon V; Carmen L Loureiro; CoViVen Group; D Angelo P; Delgado M; Domingo J Garzaro; Esmeralda Vizzi; Flor H Pujol; Garzaro D; Héctor R Rangel; Jaspe RC; José Luis Zambrano; Lieska Rodríguez; Loureiro CL; Mariana Hidalgo; Pierina D´Angelo; Pujol FH; Rangel HR; Rodriguez L; Rossana C Jaspe; Víctor Alarcón; Yoneira Sulbaran; Zambrano JL; Zoila Moros
EPI_ISL_2878585, EPI_ISL_2881318	INSACOG Surveillance	INSACOG at CSIR Institute of Genomics and Integrative Biology	INSACOG
EPI_ISL_2812628	INSACOG-MANIPUR	National Institute of Biomedical Genomics – INSACOG	Arindam Maitra; Khumanthem Ranjana; Nanaocha Sharma; Nidhan Kumar Biswas; R K Manglem Singh; Saumitra Das; Sreedhar Chinnaswamy
EPI_ISL_3019053	INSACOG-Sikkim	National Institute of Biomedical Genomics – INSACOG	Arindam Maitra; Kaden Zangmu Bhutia; Nidhan Kumar Biswas; Saumitra Das; Sreedhar Chinnaswamy Tshering Ongmu Bhutia; Tara D Sharma
EPI_ISL_2503286, EPI_ISL_2503354, EPI_ISL_2548404, EPI_ISL_2676269, EPI_ISL_3019399	INSACOG-WB	National Institute of Biomedical Genomics – INSACOG	Ajay Chakraborti; Arindam Maitra; Bhaswati Bandyopadhyay; Nidhan Kumar Biswas; Saumitra Das; Sreedhar Chinnaswamy; Tamal Ghosh
EPI_ISL_2672210, EPI_ISL_2672216, EPI_ISL_2672330, EPI_ISL_2672993	INSP, Bamako	Malaria Research and Training Center-Parasito	Abdoulaye Djimde; Amadou Daou; Antoine Dara; Demba Koita; Ibrehima Guindo
EPI_ISL_2484167	INSP-Bamako	Malaria Research and Training Center	Abdoulaye Djimde; Amadou Daou; Antoine Dara; Demba Koita; Ibrehima Guindo
EPI_ISL_826816, EPI_ISL_826825, EPI_ISL_826830, EPI_ISL_826834	INSPI-CRN DE INFLUENZA Y OTROS VIRUS RESPIRATORIOS	Instituto de Salud Publica de Chile	Alfredo Bruno; Andres Castillo; Barbara Parra; Domenica de Mora; Gisselle Barra; Jaime Lagos; Javier Tognarelli; Jimmy Garcez; Jorge Fernandez; Loredana Arata; Manuel Gonzalez; Martiza Olmedo; Michelle Paez; Patricia Bustos; Rodrigo Fasce; Solon Narvaez
EPI_ISL_2488721, EPI_ISL_2488741, EPI_ISL_2488743, EPI_ISL_2757586, EPI_ISL_2757591, EPI_ISL_2757595, EPI_ISL_2757601, EPI_ISL_2757646, EPI_ISL_2893553, EPI_ISL_2893554, EPI_ISL_2893592, EPI_ISL_2893605, EPI_ISL_2893608, EPI_ISL_2893620, EPI_ISL_2893664, EPI_ISL_2897542, EPI_ISL_2897546, EPI_ISL_2988508, EPI_ISL_2988528, EPI_ISL_2988546, EPI_ISL_2988550, EPI_ISL_2988562, EPI_ISL_3010424, EPI_ISL_3010431, EPI_ISL_3010445			
see above	INSPI-CRN DE INFLUENZA Y OTROS VIRUS RESPIRATORIOS	NIC-INSPI	Alfredo Bruno; Domenica de Mora.; Jimmy Garcés; Johanna Laines; Lizbeth Patiño; Manuel Gonzalez; Maritza Olmedo; Michelle Páez
EPI_ISL_940775	INSPI-CRN de Influenza y otros virus respiratorios	INSPI-Centro de Investigación Multidisciplinaria de la DTIDI	Alberto Orlando.; Alfredo Bruno; Andrés Carrazco-Montalvo; Doménica de Mora; Leandro Patiño; Manuel González; Maritza Olmedo; Mary Regato-Arrata; Melissa Zambrano; Orson Mestanza
EPI_ISL_1630269	IRESSEF	Abbott	Adbou Padane; Ambroise Ahouidi; Aminata Dia; Aminata Mboup; Ana Olivo; Anna julienne selbe Ndiaye; Barbara Harris; Cyrille Diedhiou; Gavin Cloherty; Mary Rodgers; Moustapha Mbow; Nafissatou Leye; Ndeye Diabou Diagne; Papa Alassane Diaw; Souleymane Mboup; Todd Meyer
EPI_ISL_2015097,	IU-Cerrahpasa, Cerrahpasa School of Medicine, COVID-19 Lab	IU-Cerrahpasa, Cerrahpasa School of Medicine, COVID-19 Lab	Kenan Midilli; Mert Kuskucu; Yesim Tuyji Tok

EPI_ISL_2015111			
EPI_ISL_2628209, EPI_ISL_2628302, EPI_ISL_2628303, EPI_ISL_2628304, EPI_ISL_2628305, EPI_ISL_2628306	IVIC	Laboratorio de Virología Molecular	Carmen L Loureiro; CoViVen Group; Domingo J Garzaro; Esmeralda Vizzi; Flor H Pujol; Héctor R Rangel; José Luis Zambrano; Lieska Rodríguez; Mariana Hidalgo; Pierina D´Angelo; Rossana C Jaspe; Víctor Alarcón; Yoneira Sulbaran; Zoila Moros
EPI_ISL_738121	IZSM-U.O.C. Virologia	Istituto Zooprofilattico Sperimentale del Mezzogiorno	Giovanna Fusco; Lorena Cardillo; Maurizio Viscardi
EPI_ISL_2350569	Ibaraki Prefectural Institute of Public Health	Ibaraki Prefectural Institute of Public Health	Keiko Goto; Kentaro Itokawa; Makoto Kuroda; Masanori Hashino; Rina Tanaka; Tsuyoshi Sekizuka
EPI_ISL_2834665, EPI_ISL_2934986	Idaho Bureau of Laboratories	Center for Global Health, University of New Mexico Health Sciences Center	Christopher Ball; Darrell Dinwiddie; Daryl Domman; Kurt Schwalm; Matthew Burns; Robert Voermans; Valerie Morley
EPI_ISL_1323469	Illinois Department of Public Health	Gagnon Lab, Southern Illinois University	Keith Gagnon
EPI_ISL_1018095, EPI_ISL_2001061, EPI_ISL_2001063, EPI_ISL_2001093, EPI_ISL_2508380	Immunology, Noguchi Memorial Institute for Medical Research	Immunology, Noguchi Memorial Institute for Medical Research	A.W.; Adu, B.; Adusei-Poku; Agbenyo; Agbodzi, B.; Ampofo; Appiah-Kubi, J.; Asare; Assane, H.; Attiku; Awunyo, S.; Bonney; Dagnran, A.; Dorkenoo, A.; Egyir, B.; Halatoko; Issa, Z.; J.K.; K.M.; K.O.; Kossi, K.; Kumordjie, S.; Lamboni, L.; Layibo, Y.; M.A.; Maman, I.; Mohktar, Q.; Odoom; Opoku Frempong, H.; Oteng, F.; Owusu-Nyantakyi, C.; P.O.; S.B.; Sadji; Salah, D.; Salou, M.; Uche; W.K.; Y.A.; Yeboah, C.
EPI_ISL_2881744	InDRE	Instituto Nacional de Medicina Genomica	Cedro-Tanda A; Garcia-Cardenas FJ; Gisela Barrera-Badillo; Gonzalez-Barrera D; Gonzalez-Woge MA; Herrera-Montalvo LA.; Hidalgo-Miranda A; Irma Lopez-Martinez; Jose Ernesto Ramirez González; Mendoza-Vargas A; Munguia-Garza P; Rangel-DeLeon D; Reyes-Grajeda JP
EPI_ISL_1517392, EPI_ISL_1517399, EPI_ISL_1517404, EPI_ISL_1517416	Inciensa, Instituto Costarricense de Investigación y Enseñanza en Nutrición y Salud	Inciensa, Instituto Costarricense de Investigación y Enseñanza en Nutrición y Salud	Cristian Peréz-Corrales; Cristian Peréz-Corrales & Valeria Peralta-Barquero; Valeria Peralta-Barquero & César Cerdas Quesada; Valeria Peralta-Barquero & Mauricio Bolaños; Valeria Peralta-Barquero & Patricia Salas
EPI_ISL_476704	Incubadora Venezolana de Ciencia, Venezuela	Incubadora Venezolana de Ciencia, Venezuela / Instituto Nacional de Salud, Bogotá, Colombia / Grupo de Investigaciones Microbiológicas-UR (GIMUR), Departamento de Biología, Facultad de Ciencias Naturales, Universidad del Rosario, Bogotá, Colombia / Icahn School of Medicine at Mount Sinai, New York, USA	Alberto Paniz-Mondolfi; Ana S. Gonzalez-Reiche; Angelica Rico; Aníbal A. Teherán; Carolina Florez; Carolina Hernández; Emilia Mia Sordillo; Esther C. Barros; Harm van Bakel; Jesús E. Jaimes; Juan David Ramírez; Lisseth Pardo; Lourdes Delgado; Luis Perez-Garcia; Marina Muñoz; Matthew M. Hernandez; Sergio Gomez; Viviana Simon
EPI_ISL_3000158	Infection Prevention & Control Unit	Central Virology laboratory, Ministry of Health	Asaf Biber; Danis Sofer; Efrat Bucris; Ella Mendelson; Gili Regev-Yochay; Ilana Tal; Michal Mandelboim; Miki Goldenfeld; Neta Zuckerman; Orna Mor; Sabrina Hason; Sharon Amit; Shiraz Gefen-Halevi
EPI_ISL_2693906	Infectious Disease and Tropical Medicine Research Center, Resistant Tuberculosis Institute, Zahedan University of Medical Sciences, Zahedan, Iran	Genetics Research Center, University of Social Welfare and Rehabilitation Sciences	Ali Jafarpour; Azam Ghaziasadi; Ebrahim Kord; Hossein Najmabadi.; Khadijeh Jalalvand; Kimia Kahrizi; Marzieh Mohseni; Seyed Mohammad Hashemi-Shahri; Seyed Mohammad Jazayeri; Seyedeh elham Mortazavi; Zohreh Fattahi
EPI_ISL_1969944	Infectious Diseases Hospital IASI	National Institute of Infectious Diseases-Prof. Dr. Matei Bals Molecular Diagnostics Laboratory	Andreea Tudor; Corina Casangiu; Dan Otelea; Leontina Banica; Marius Surleac; Ovidiu Vlaicu; Simona Paraschiv
EPI_ISL_856714, EPI_ISL_856715	Infectious Diseases Unit, Department of Internal Medicine, Azienda Ospedaliera-Universitaria di Padova	Laboratory of Infectious Diseases, Department of Biomedical and Clinical Sciences L. Sacco, University of Milan	Alessia Lai; Anna Maria Cattelan; Annalisa Bergna; Carla Della Ventura; Claudia Balotta; Davide Leoni; Gianguglielmo Zehender on behalf of SARS-CoV-2 ITALIAN RESEARCH ENTERPRISE-(SCIRE) Collaborative Group; Lolita Sasset; Massimo Galli
EPI_ISL_2107507, EPI_ISL_2151337	Infectious Diseases, King Faisal Hospital Research Center	Infectious Diseases, King Faisal Hospital Research Center	Al-Qahtani A, A.; Alahideb B, M.; Alhamlan F, S.; Almaghrabi R, S.; Alsanea M; Alsanea M, S.; Althawadi S, I.; Balavenkatesh Mani; Balavenkatesh Mani, M.; M. and UdayaRaja G, K.; Mutabagani M, S.; S. and Alahideb B, M.; UdayaRaja G, K.
EPI_ISL_2013699, EPI_ISL_2014214, EPI_ISL_2089063, EPI_ISL_2282745, EPI_ISL_2282747	Infinity Biologix	Centers for Disease Control and Prevention Division of Viral Diseases, Pathogen Discovery	Adrian Paskey; Benjamin Rambo-Martin; Chirayu Goswami; Christian Bixby; Christopher Gulvick; Clinton R. Paden; Dakota Howard; Darlene Wagner; Dhwani Batra; Duncan MacCannell; Jason Caravas; Jonathan Schultz; Kara Moser; Matthew Schmerer; Peter W. Cook; Robin Grimwood; Russ Hager; Scott Sammons; Shatavia Morrison; Yihe Wang; Yvette Unoarumhi
EPI_ISL_1989269	Innlandet Hospital Trust, Division Lillehammer, Department for Medical Microbiology	Norwegian Institute of Public Health, Department of Virology	Atiya R Ali; Debech Nadia; Engebretsen Serina Beate; Garcia Llorente Ignacio; Hilde Elshaug; Hilde Vollan; Jon Bråte; Kamilla Heddeland Instefjord; Karoline Bragstad; Kathrine Stene-Johansen; Marie Paulsen Madsen; Olav Hungnes; Pedersen Benedikte Nevjen; Rasmus Riis Kopperud
EPI_ISL_2281228, EPI_ISL_2281229, EPI_ISL_2281230, EPI_ISL_2281237, EPI_ISL_2281238, EPI_ISL_2281245, EPI_ISL_2281256			
see above	Insitut Pasteur Bangui	Institut Pasteur de Dakar	Amadou Alpha Sall; Amadou Diallo; Benjamin Selekon; Cheikh Loucoubar; Christian Malaka; Mamadou Diop; Marie-Astrid Vernet; Moussa Moïse Diagne; Ndack Ndiaye; Ndongo Dia; Ousmane Faye; Rivalyn Nakoune Yandoko; Safietou Sankhe; Sandra Garba Ouangole
EPI_ISL_2828655	Instituo Adolfo Lutz Central	Instituto Adolfo Lutz, Interdiciplinary Procedures Center, Strategic Laboratory	Caio Vinicius Dias Lopes; Claudia Regina Gonçalves; Claudio Tavares Sacchi; Erica Valessa Ramos Gomes; Karoline Rodrigues Campos
EPI_ISL_1907961, EPI_ISL_1908147, EPI_ISL_1908148, EPI_ISL_1908155, EPI_ISL_1908158, EPI_ISL_1908735, EPI_ISL_1908805, EPI_ISL_1908896, EPI_ISL_1908967, EPI_ISL_1909099, EPI_ISL_1909211, EPI_ISL_1909241, EPI_ISL_1909242, EPI_ISL_1909243, EPI_ISL_1909244, EPI_ISL_1909247, EPI_ISL_1909250, EPI_ISL_1909252, EPI_ISL_1913009, EPI_ISL_1913018, EPI_ISL_1913019, EPI_ISL_1913061, EPI_ISL_1913077, EPI_ISL_1913081, EPI_ISL_1913083			
see above	Institut National d'Hygiène	Laboratoire de Biotechnologie	Abdelomunim Essabbar; Fatima El Falaki; Fatima ElFalaki; Hicham Oumzil; Lahcen Belyamani and Azeddine Ibrahimi; Mohamde Rhajaoui; Mohamed Rhajaoui; Mouna Ouadghiri; Saaïd Amzazi; Tarik Aanniz
EPI_ISL_1433132, EPI_ISL_1435763, EPI_ISL_1436817	Institut National d'Hygiène	Unité Mixte Internationale TransVIHMI (UMI 233 IRD – U1175 INSERM - Université de Montpellier) IRD (Institut de recherche pour le développement)	Abla A. KONOU; Adodo SADJI; Ahidjo AYOUBA; Akoélé SILIADIN; Alassane OURO-MEDELI; Amivi EHLAN; Améyo DORKENOO; Anoumou DAGNRA; Christelle BUTEL; Déléma MABA; Eric DELAPORTE; Issaka Maman; Kokou TEGUENI; Laetitia SERRANO; Martine PEETERS; Messanh DOUFFAN; Mireille PRINCE-DAVID; Mounerou SALOU; Sidonie A.M.KAGNISSODE; Sika DOSSIM; Wembo A. HALATOKO
EPI_ISL_2111158	Institut National d'Hygiène Consortium National de Veille Génomique - Maroc	Laboratoire de Biotechnologie	Abdelomunim Essabbar; Fatima El Falaki; Hicham Oumzil; Lahcen Belyamani and Azeddine Ibrahimi; Mohamed Rhajaoui; Mouna Ouadghiri; Saaïd Amzazi; Tarik Aanniz
EPI_ISL_1415425, EPI_ISL_1416192, EPI_ISL_1443003	Institut National d'hygiène	"Unité Mixte Internationale TransVIHMI (UMI 233 IRD – U1175 INSERM - Université de Montpellier) IRD (Institut de recherche pour le développement)"	Abla A. KONOU; Adodo SADJI; Ahidjo AYOUBA; Akoélé SILIADIN; Alassane OURO-MEDELI; Amivi EHLAN; Améyo DORKENOO; Anoumou DAGNRA; Christelle BUTEL; Déléma MABA; Eric DELAPORTE; Issaka Maman; Kokou TEGUENI; Laetitia SERRANO; Martine PEETERS; Messanh DOUFFAN; Mireille PRINCE-DAVID; Mounerou SALOU; Sidonie A.M.KAGNISSODE; Sika DOSSIM; Wembo A. HALATOKO
EPI_ISL_1508958, EPI_ISL_1508960, EPI_ISL_1509101	Institut National d'hygiène	Unité Mixte Internationale TransVIHMI (UMI 233 IRD – U1175 INSERM - Université de Montpellier) IRD (Institut de recherche pour le développement)	Abla A. KONOU; Adodo SADJI; Ahidjo AYOUBA; Akoélé SILIADIN; Alassane OURO-MEDELI; Amivi EHLAN; Améyo DORKENOO; Anoumou DAGNRA; Christelle BUTEL; Déléma MABA; Eric DELAPORTE; Issaka Maman; Kokou TEGUENI; Koku AGBODEKA; Laetitia SERRANO; Martine PEETERS; Messanh DOUFFAN; Mireille PRINCE-DAVID; Mounerou SALOU; Sidonie A.M.KAGNISSODE; Sika DOSSIM; Syntyche DEVATCHAGNI; Wembo A. HALATOKO
EPI_ISL_1321604, EPI_ISL_1406144, EPI_ISL_2278010	Institut National d'Hygiène (INH)	Unité Mixte Internationale TransVIHMI (UMI 233 IRD – U1175 INSERM - Université de Montpellier)IRD (Institut de recherche pour le développement)	Abla A. KONOU; Adodo SADJI; Ahidjo AYOUBA; Akoélé SILIADIN; Alassane OURO-MEDELI; Amivi EHLAN; Améyo DORKENOO; Anoumou DAGNRA; Christelle BUTEL; Déléma MABA; Eric DELAPORTE; Issaka Maman; Kokou TEGUENI; Laetitia SERRANO; Martine PEETERS; Messanh DOUFFAN; Mireille PRINCE-DAVID; Mounerou SALOU; Sidonie A.M.KAGNISSODE; Sika DOSSIM; Wembo A. HALATOKO
EPI_ISL_418206, EPI_ISL_418210, EPI_ISL_418212, EPI_ISL_476562, EPI_ISL_480783, EPI_ISL_480789, EPI_ISL_485708, EPI_ISL_486869			
see above	Institut Pasteur Dakar	Institut Pasteur de Dakar	Amadou Alpha Sall; Amadou Alpha Sall.; Amadou Alpha sall; Mamadou Diop; Mamadou Malado Jallow; Mamadou diop; Marie Henriette Dior Ndione; Moussa Moise Diagne; Ndongo Dia; Ousmane Faye; Safietou Sanke
EPI_ISL_498239	Institut Pasteur de Dakar	Institut Pasteur de Dakar	Amadou Alpha Sall.; Mamadou Diop; Mamadou Malado Jallow; Marie Henriette Dior Ndione; Moussa Moise Diagne; Ndongo Dia; Ousmane Faye; Safietou Sankhe Mbengue
EPI_ISL_2245787, EPI_ISL_2245795, EPI_ISL_2245816, EPI_ISL_2245819, EPI_ISL_2245821, EPI_ISL_2245840, EPI_ISL_2245855, EPI_ISL_2245859, EPI_ISL_2245862, EPI_ISL_2245864, EPI_ISL_2245865, EPI_ISL_2245882, EPI_ISL_2245892, EPI_ISL_2245894, EPI_ISL_2245897, EPI_ISL_2245910, EPI_ISL_2245931, EPI_ISL_2245957, EPI_ISL_2245958, EPI_ISL_2245960, EPI_ISL_2245964, EPI_ISL_2245966, EPI_ISL_2245977			
see above	Institut Pasteur de Guinée	Institut Pateur de Dakar	Dia Ndongo; Diagne Moussa Moïse; Diallo Amadou; Diop Mamadou; Faye Ousmane; Grayo Solene; Loucoubar Cheikh; Maimouna Mbanne; Mbengue Safietou Sankhe; Mohamed Kane; Ndiaye Ndack; Sall Amadou Alpha; Tordo Noel
EPI_ISL_613421, EPI_ISL_613438, EPI_ISL_613441, EPI_ISL_613446, EPI_ISL_613455	Institut Pasteur de la Guadeloupe	Institut Pasteur de la Guadeloupe	Angela Brisebarre; Antoine Talarmin; Camille Capel; Cherina Fleming; Etienne Simon-Lorière; Marion Barbet; Maud Vanpeene; Méline Bizard; Radjin Steingrover; Stéphanie Guyomard; Sylvie Behillil; Sylvie van der Werf; Sébastien Breurec; Vincent Enouf
EPI_ISL_459971	Institut Pasteur du Maroc	Institut Pasteur du Maroc	Abdellah Faouzi; Anass Abbad; Anderrahmane Maaroufi; Angela Brisebarre; Camille Capel; Etienne Simon-Lorière; Jalal Nourlil; Latifa Anga; Marion Barbet; Maud Vanpeene; Mjid Eloualid; Méline Bizard; Sylvie Behillil; Sylvie van der Werf; Vincent Enouf
EPI_ISL_475820, EPI_ISL_1008302	Institut für Virologie am Department für Hygiene, Mikrobiologie und Public Health	Bergthaler laboratory, CeMM Research Center for Molecular Medicine of the Austrian Academy of Sciences	Alexander Lercher; Alexandra Popa; Andreas Bergthaler; Anna Schedl; Bekir Erguner; Benedikt Agerer; Christoph Bock; Daniela Schmid; Dorothee von Laer; Elisabeth Puchhammer-Stoeckl; Franz Allerberger; Gregor Hörmann; Guenter Weiss; Henrique Colaco; Jakob-Wendelin Genger; Jan Laine; Judith Aberle; Kinga Rigler-Hohenwarter; Lukas Endler; Maelle Le Moing; Manfred Nairz; Mark Smyth; Martin Senekowitsch; Michael Schuster; Peter Hufnagl; Rainer Gattringer; Stephan Aberle; Thomas Penz; Wegene Borena
EPI_ISL_955168	Institute for Biocides and Medical Ecology	Institute of microbiology and Immunology, Faculty of Medicine, University of Belgrade	Jankovic, M.; Jovanovic, T.; Knezevic, A.; Milicevic, O.; Sekler, M.; Tesovic, B.; Vidanovic, D.
EPI_ISL_1657085, EPI_ISL_1915108	Institute for Developing Science and Health Initiatives (ideSHi)	Institute for Developing Science and Health Initiatives (ideSHi)	Fidausi Qadri; Hassan Afrad; Sadia Rahman; Tahmina Shirin
EPI_ISL_1822604, EPI_ISL_1827861, EPI_ISL_1827862, EPI_ISL_1827865, EPI_ISL_1827890, EPI_ISL_1827915, EPI_ISL_1827932, EPI_ISL_1827945			
see above	Institute for Health Research, Epidemiological Surveillance and Training (IRESSEF)	Abbott Laboratories	Adbou Padane; Ambroise Ahouidi; Aminata Dia; Aminata Mboup; Ana Olivo; Anna julienne selbe Ndiaye; Barbara Harris; Cyrille Diedhiou; Gavin Cloherty; Mary Rodgers; Moustapha Mbow; Nafissatou Leye; Ndeye Diabou Diagne; Papa Alassane Diaw; Souleymane Mboup; Todd Meyer
EPI_ISL_574790	Institute for Infectious Diseases, University of Bern	Institute for Infectious Diseases, University of Bern	Alban Ramette; Christian Baumann; Cora Sägesser; Franziska Suter-Riniker; Michel C Koch; Miguel A Terrazos Miani; Peter Keller; Stephen L Leib
EPI_ISL_2758186	Institute for Laboratory Diagnostics and Microbiology, Klinikum Klagenfurt am Wörthersee	Bergthaler laboratory, CeMM Research Center for Molecular Medicine of the Austrian Academy of Sciences	Andreas Bergthaler; Anna Schedl; Bekir Erguner; Benedikt Agerer; Christoph Bock; Fabian Amman; Jan Laine; Lukas Endler; Maelle Le Moing; Martin Senekowitsch; Matthew Thornton; Michael Schuster; Petr Triska; Thomas Penz

EPI_ISL_490090, EPI_ISL_490096, EPI_ISL_490101, EPI_ISL_490103, EPI_ISL_644672, EPI_ISL_718289, EPI_ISL_718301, EPI_ISL_944102, EPI_ISL_1114744, EPI_ISL_1263458, EPI_ISL_1406244, EPI_ISL_1673342, EPI_ISL_1696746, EPI_ISL_1696754, EPI_ISL_1787317, EPI_ISL_2091000, EPI_ISL_2625633, EPI_ISL_2685838			
see above	Institute for Medical Research, Infectious Disease Research Centre, National Institutes of Health, Ministry of Health Malaysia	Institute for Medical Research, Infectious Disease Research Centre, National Institutes of Health, Ministry of Health Malaysia	Azizan MA; Kalyanasundram J; Kamel K; Mohd Zawawi Z; Mohd-Zawawi Z; Ramly N; Robert F; Suppiah J; Thayan R
EPI_ISL_2324270	Institute for Medical and Chemical Laboratory Diagnostics, Kepler Universitätsklinikum	Bergthaler laboratory, CeMM Research Center for Molecular Medicine of the Austrian Academy of Sciences	Andreas Bergthaler; Anna Schedl; Bekir Erguner; Benedikt Agerer; Christoph Bock; Fabian Amman; Jan Laine; Lukas Endler; Maelle Le Moing; Martin Senekowitsch; Michael Schuster; Petr Triska; Thomas Penz
EPI_ISL_812955, EPI_ISL_1491539, EPI_ISL_1511131	Institute for Urban Disease Control and Prevention	COVID-19 Network Investigations (CONI) Alliance	Amornmas Kongklieng; Anek Mungaomklang; Angkana Huang; Anthony R. Jones; Arporn Wangwiwatsin; Bhakbhoom Panthan; Chonticha Klungtong; Duangkamon Loesbanluechai; Ekawat Pasomsub; Elizabeth Batty; Insee Sensorn; Janjira Thaipadungpanit; Kamolthip Atsawawaranunt; Khajohn Joonlasak; Khajohn Joonsalak; Kingkan Rakmanee; Krittikorn Kumpornsin; Namfon Kotanan; Prayuth Kaewmalang; Pukkaporn Parnwijitkul; Stefan Fernandez; Thanat Chookajorn; Theerarat Kochakarn; Treewat Watthanachockchai; Vichan Pawun; Wasun Chantratita; Wudtichai Manasatienkij
EPI_ISL_626228	Institute for Virology, University Hospital Essen	Center of Medical Microbiology, Virology, and Hospital Hygiene, University of Duesseldorf	Alexander Dilthey; Andreas Walker; Daniel Strelow; Jessica Nicolai; Jörg Timm; Klaus Pfeffer; Lisanna Hülse; Malte Kohns Vasconcelos; Maximilian Damagnez; Nadine Lübke; Olympia E. Anastasiou; Tobias Wienemann; Torsten Houwaart; Ulf Dittmer
EPI_ISL_2137158	Institute for Water Quality and Resource Management, Technical University Vienna	Bergthaler laboratory, CeMM Research Center for Molecular Medicine of the Austrian Academy of Sciences	Andreas Bergthaler; Anna Schedl; Bekir Erguner; Benedikt Agerer; Christoph Bock; Fabian Amman; Jan Laine; Lukas Endler; Maelle Le Moing; Martin Senekowitsch; Michael Schuster; Petr Triska; Thomas Penz
EPI_ISL_1057041, EPI_ISL_1057098	Institute of Biocides and Medical Ecology, Belgarde, Serbia	Virology Department Institute of Microbiology and Immunology Faculty of Medicine University of Belgrade	Abazovic Dzihan; Banko Ana; Despot Dragana; Loncar Ana; Milicevic Ognjen; Miljanovic Danijela
EPI_ISL_891266	Institute of Biocides and Medical Ecology, Belgrade, Serbia	Virology department Institute of microbiology and immunology Faculty of Medicine University of Belgrade	Abazovic Dzihan; Banko Ana; Despot Dragana; Loncar Ana; Milicevic Ognjen; Miljanovic Danijela
EPI_ISL_2180456, EPI_ISL_2284894, EPI_ISL_2600368, EPI_ISL_2600369, EPI_ISL_2774039, EPI_ISL_2774043, EPI_ISL_2774074			
see above	Institute of Epidemiology, Disease Control and Research (IEDCR)	IEDCR-ideSHi-icddr,b	Firdausi Qadri; Hassan Afrad; Manjur Hossain Khan; Sadia Rahman; Tahmina Shirin
EPI_ISL_1938476	Institute of Epidemiology, Disease Control and Research (IEDCR)	Institute for Developing Science and Health Initiatives (ideSHi)	Fidausi Qadri; Hassan Afrad; Sadia Rahman; Tahmina Shirin
EPI_ISL_864573	Institute of Medical Microbiology and Hospital Hygiene	Institute of Medical Microbiology and Hospital Hygiene	Aljoscha Tersteegen; Prof. Dr. Achim Kaasch
EPI_ISL_1511107, EPI_ISL_1668835, EPI_ISL_1964492, EPI_ISL_2492324, EPI_ISL_2492327, EPI_ISL_2644269, EPI_ISL_2932442, EPI_ISL_2955269, EPI_ISL_2966919, EPI_ISL_3039361			
see above	Institute of Microbiology and Immunology, Faculty of Medicine, University of Ljubljana	Institute of Microbiology and Immunology, Faculty of Medicine, University of Ljubljana	Alen Suljič; Andraž Celar; Dominika Šturm; Doroteja Vlaj; Mario Poljak; Matic Brvar; Miša Korva; Patricija Pozvek; Samo Zakotnik; Tatjana Avšič – Županc; Tomaž Mark Zorec; Špela Pleh
EPI_ISL_417482, EPI_ISL_477016, EPI_ISL_491936, EPI_ISL_516650, EPI_ISL_516651, EPI_ISL_527813, EPI_ISL_539789, EPI_ISL_660530, EPI_ISL_697798, EPI_ISL_728202, EPI_ISL_824285, EPI_ISL_877557, EPI_ISL_1805654, EPI_ISL_1805657, EPI_ISL_1805711, EPI_ISL_1896678, EPI_ISL_1896691, EPI_ISL_1896695, EPI_ISL_2004102, EPI_ISL_2004105, EPI_ISL_2086711, EPI_ISL_2100425, EPI_ISL_2100438, EPI_ISL_2134873, EPI_ISL_2228102, EPI_ISL_2228105, EPI_ISL_2361460, EPI_ISL_2361462, EPI_ISL_2361472, EPI_ISL_2361476, EPI_ISL_2484966, EPI_ISL_2534327, EPI_ISL_2689843, EPI_ISL_2774510, EPI_ISL_2861153			
see above	Institute of Microbiology, Universidad San Francisco de Quito	Institute of Microbiology, Universidad San Francisco de Quito	; Alberto Velez; Andrea Macías; Belén Prado-Vivar; Bernardo Darquea; Bernardo Gutiérrez; Carla Torres; Carlos Guerrero; Carlos Mena; Carolina Pacheco; Christian Zambrano; DamarisSandoya; David Zuñiga; Diana Zambrano; Edison Chavez; Erika Muñoz; Ernesto Pazos; Eulalia Pazmiño; Fernada Zurita; Fernanda Zurita; Fernando Serrano; Francisco Ríos; Fredy Loor; Gabriel Trueba; Gabriela Zambrano; Guzmán Bernabéu Lorenzo; Hermelinda Paguay; Hugo Hernandéz; Hugo Hernández; Jorge Luis Velez; Jorge Montaño; Juan Carlos Zuñiga; Juan José Guadalupe; Juan Pablo Haro; Juan Zuñiga; Katalina Pacheco; Katherine Ojeda; Khurram Mahbbob; Ligia Briceño; Luis Flores; Luis Morales; Manuel Jaramillo; Manuel Jibaja; Mayra Beltrán; Melissa Ortega; Melva Morales; Michelle Grunauer; Milton Tobar; Miriam Ruiz; Monica Becerra-Wong; Monica Becerra-Wong Juan José Guadalupe; Nabih Dahik; Oscar Mena; Paola Dalgo; Patricio Reyes; Patricio Rojas-Silva; Patricio Vega-Mariño; Paúl Cárdenas; Prado-Vivar; Rafael Parra; Raiza Briceño; Ramiro Echeverría Tapia; Rommel Guevara; Sully Márquez; Tania Guayasamin; Tania Lluma; Tanya Guayasamin; Verónica Barragán; Victor Vinicio Román
EPI_ISL_1745684, EPI_ISL_1745694, EPI_ISL_1745705, EPI_ISL_2346373, EPI_ISL_2727514, EPI_ISL_3006762	Institute of Molecular and Translational Medicine / Laboratory of Experimental Medicine, Faculty of Medicine and Dentistry, Palacky University and University Hospital Olomouc	Institute of Molecular and Translational Medicine / Laboratory of Experimental Medicine, Faculty of Medicine and Dentistry, Palacky University	Barbora Blumová; Hana Dřímalová; Hana Jaworek; Marián Hajdúch; Rastislav Slavkovský; Tomáš Pospíšíl; Vladimíra Koudeláková
EPI_ISL_402123	Institute of Pathogen Biology, Chinese Academy of Medical Sciences & Peking Union Medical College	Institute of Pathogen Biology, Chinese Academy of Medical Sciences & Peking Union Medical College	Chao Wu; Jianwei Wang; Lili Ren; Qi Jin; Yiwei Liu; Zhiqiang Wu; Zichun Xiang
EPI_ISL_2932530, EPI_ISL_2932531, EPI_ISL_2986987	Institute of Public Health Kraljevo	Veterinary Specialized Institute Kraljevo	Afonso, C.; Banovic Djeri, B.; Djukic V, .; Jankovic, M.; Jovanovic, T.; Knezevic, A.; Petrovic, T.; Sekler, M.; Tesovic, B.; Vidanovic, D.; Volkening, J.
EPI_ISL_2657582	Institute of Public Health Šibenik-Knin County	Croatian Institute of Public Health	Irena Tabain; Ivana Ferenčak
EPI_ISL_1712568	Institute of Virology Department of Hygiene, Microbiology and Public Health at Innsbruck Medical University	Institute of Virology Department of Hygiene, Microbiology and Public Health at Innsbruck Medical University	Andre Volland; Barbara Falkensammer; David Bante; Dorothee von Laer; Heribert Stoiber; Janine Kimpel; Lisa Pipperger; Lydia Riepler; Wegene Borena
EPI_ISL_577740, EPI_ISL_577742, EPI_ISL_904001, EPI_ISL_1994596, EPI_ISL_2351249, EPI_ISL_2894457	Institute of Virology, Biomedical Research Center of the Slovak Academy of Sciences, Bratislava	Faculty of Natural Sciences, Comenius University, Bratislava	Boris Klempa; Brona Brejova; Broňa Brejová; Dominika Fričová; Edita Staroňová; Elena Tichá; Jozef Nosek; Juraj Kopacek; Juraj Kopáček; Kristina Borsova; Kristína Boršová; Lubomira Lukacikova; Martina Lickova; Martina Ličková; Martina Nebohacova; Martina Neboháčová; Monika Slavikova; Monika Sláviková; Sabina Fumacova Havlikova; Sabina Fumačová Havlíková; Tomas Vinar; Tomáš Vinař; Viktoria Cabanova; Viktoria Hodorova; Viktória Hodorová; Viktória Čabanová; Ľubomíra Lukáčiková
EPI_ISL_1654810, EPI_ISL_1654830	Institute of Virology, Vaccines and Sera "Torlak"	Institute of microbiology and Immunology, Faculty of Medicine, University of Belgrade	Jankovic, M.; Jovanovic, T.; Knezevic, A.; Milicevic, O.; Sekler, M.; Tesovic, B.; Vidanovic, D.
EPI_ISL_1017684, EPI_ISL_1017686, EPI_ISL_1017688	Institute of Virology, Vaccines and Sera ''Torlak''	Institute of microbiology and Immunology, Faculty of Medicine, University of Belgrade	Jankovic, M.; Jovanovic, T.; Knezevic, A.; Milicevic, O.; Sekler, M.; Tesovic, B.; Vidanovic, D.
EPI_ISL_833163	Instituto Adolfo Lutz - Regional de Marilia	Instituto Adolfo Lutz, Interdiciplinary Procedures Center, Strategic Laboratory	Claudia Regina Gonçalves; Claudio Tavares Sacchi; Erica Valessa Ramos Gomes; Karoline Rodrigues Campos
EPI_ISL_2614622	Instituto Adolfo Lutz - Regional de Presidente Prudente	Instituto Adolfo Lutz, Interdiciplinary Procedures Center, Strategic Laboratory	Caio Vinicius Dias Lopes; Claudia Regina Gonçalves; Claudio Tavares Sacchi; Erica Valessa Ramos Gomes; Karoline Rodrigues Campos; Leonardo Jose Tadeu de Araujo
EPI_ISL_984246	Instituto Adolfo Lutz Central	Instituto Adolfo Lutz, Interdiciplinary Procedures Center, Strategic Laboratory	Claudia Regina Gonçalves; Claudio Tavares Sacchi; Erica Valessa Ramos Gomes; Karoline Rodrigues Campos
EPI_ISL_2344311, EPI_ISL_2344420	Instituto Butantan	Instituto de Medicina Tropical de Sao Paulo	Brazil-UK Centre for Arbovirus Discovery Diagnosis Genomics and Epidemiology (CADDE) Genomic Network - Instituto de Medicina Tropical
EPI_ISL_491196, EPI_ISL_491207, EPI_ISL_491212	Instituto Gulbenkian de Ciência	Instituto Gulbenkian de Ciência	Cathy Paulino; Joao Sobral; João Costa; Ricardo Leite; Susana Ladeiro
EPI_ISL_2348751, EPI_ISL_2348766, EPI_ISL_2348777	Instituto Nacional De Investigación En Salud Pública-Crn De Influenza Y Otros Virus Respiratorios	NIC-Instituto Nacional de Investigación en Salud Pública	Alfredo Bruno; Domenica de Mora.; Jimmy Garcés; Johanna Laines; Lizbeth Patiño; Manuel Gonzalez; Maritza Olmedo; Michelle Páez
EPI_ISL_837720	Instituto Nacional de Enfermedades Respiratorias (INER)	Instituto Nacional de Enfermedades Respiratorias (INER)	Alejandra Hernández-Terán; Alma Rincón-Rubio; Celia Boukadida; Edgar Sevilla-Reyes; Eduardo Becerril-Vargas; Fidencio Mejía-Nepomuceno; Hector Esteban Paz-Juárez; Joel Armando Vázquez-Pérez; Jorge Salas-Hernández; José Arturo Martínez-Orozco; Margarita Matías-Florentino; Mario Mújica-Sánchez; Olivia Briceño; Santiago Ávila-Ríos
EPI_ISL_574294	Instituto Nacional de Investigacion en Salud Pública	Instituto Nacional de Investigación en Salud Pública	Alberto Orlando Narvaez; Alfredo Bruno; Andrés Carrasco; Denisse Portugal; Doménica de Mora Coloma; Leandro Patino Patino; Manuel Gonzalez; Orson Mestanza
EPI_ISL_491950	Instituto Nacional de Investigación en Salud Pública - INSPI	INSPI - Charité	Alberto Orlando; Alexandra Usiña; Alfredo Bruno Caicedo; Andres Moreira-Soto; Anna-Lena Sander; Denisses Portugal; Domenica de Mora Coloma; Jan Felix Drexler; Juan Carlos Zeballos; Manuel Gonzalez; Maritza Olmedo; Nina Krause; Silvia Salgado
EPI_ISL_2492549, EPI_ISL_2492604, EPI_ISL_2492663, EPI_ISL_2492696, EPI_ISL_2492728, EPI_ISL_2492780, EPI_ISL_2492786, EPI_ISL_2492787, EPI_ISL_2492788, EPI_ISL_2492789, EPI_ISL_2492799, EPI_ISL_2492803, EPI_ISL_2492818, EPI_ISL_2492822, EPI_ISL_2492846, EPI_ISL_2492847, EPI_ISL_2492882, EPI_ISL_2492915, EPI_ISL_2493087, EPI_ISL_2617133, EPI_ISL_2617181, EPI_ISL_2617182, EPI_ISL_2685951, EPI_ISL_2685957, EPI_ISL_2685960, EPI_ISL_2685973, EPI_ISL_2841653, EPI_ISL_2841654			
see above	Instituto Nacional de Investigação em Saúde	CERI, Centre for Epidemic Response and Innvoation, Stellenbosch University and KRISP, KZN Research Innovation and Sequencing Platform, UKZN.	Afonso P; David K; Emmanuel SJ; Freitas RH; Giandhari J; Inglês L; Lutucuta S; Miranda J; Morais J; Mufinda M; Naidoo Y; Neto Z; Paulo A Carralero RR Paixão JP; Pereira A; Pillay S; Tegally H; Wilkinson E; de Oliveira T
EPI_ISL_1347897, EPI_ISL_1545274, EPI_ISL_1545278, EPI_ISL_1545383, EPI_ISL_2494880, EPI_ISL_2494884, EPI_ISL_2494900, EPI_ISL_2609486, EPI_ISL_2609493, EPI_ISL_2609531, EPI_ISL_2609540, EPI_ISL_2609541, EPI_ISL_2609561			
see above	Instituto Nacional de Investigação em Saúde	KRISP, KZN Research Innovation and Sequencing Platform	Afonso P; David K; Emmanuel SJ; Freitas RH; Giandhari J; Inglês L; Lutucuta S; Miranda J; Morais J; Mufinda M; Naidoo Y; Neto Z; Paulo A Carralero RR Paixão JP; Pereira A; Pillay S; Tegally H; Wilkinson E; de Oliveira T
EPI_ISL_933521, EPI_ISL_1591655, EPI_ISL_1711328, EPI_ISL_2230812, EPI_ISL_2894314	Instituto Nacional de Medicina Genomica	Instituto Nacional de Medicina Genomica	Alcaraz N; Canseco Mendez JC; Cedro-Tanda A; Cisneros- Villanueva M; Cisneros-Villanueva M; Escobar-Arrazola; Garcia-Cardenas FJ; Gonzalez-Barrera D; Gonzalez-Woge MA; Herrera-Montalvo LA; Herrera-Montalvo LA.; Hidalgo-Miranda A; M; Mendoza-Vargas A; Miranda-Ortiz H; Munguia-Garza P; Peñaloza-Figueroa F; Ramirez-Vega O; Rangel-DeLeon D; Reyes-Grajeda JP; Rosas-Escobar P
EPI_ISL_2500953, EPI_ISL_2500971	Instituto Nacional de Salud	Instituto Nacional de Salud- Dirección de Investigación en Salud Pública	Carlos Franco-Muñoz; Carmen Osorio; Diana Malo; Diego A. Álvarez-Díaz; Diego Andrés Prada; Gerardo Santamaría; Hector Alejandro Ruiz-Moreno; Jhonnatan Reales-González; Jorge Rivera; Juan Camilo Martinez; Julian Naizaque; Katherine Laiton-Donato; Lisseth Pardo; Magdalena Wiesner; Marcela Mercado-Reyes; Maria T. Herrera-Sepúlveda; Marta Lopez Blanco; Martha Lucia Ospina Martinez; Paola Rojas; Sergio Gomez; Sheryll Corchuelo; Ángela Alarcon Cruz
EPI_ISL_791087, EPI_ISL_791096	Instituto Nacional de Salud - Unidad de Secuenciación y Análisis Genómico	Instituto Nacional de Salud - Dirección de Investigación en Salud Pública	Carlos Franco-Muñoz; Diego A. Álvarez-Díaz; Diego Andrés Prada; Gerardo Santamaría; Jonathan Reales; Julian Naizaque; Katherine Laiton-Donato; Magdalena Wiesner; Marcela Mercado-Reyes; Maria T. Herrera; Martha Lucia Ospina Martinez; Mauricio Pacheco-Montealegre; Paola Muñoz-Laiton; Sheryll Corchuelo

EPI_ISL_941955, EPI_ISL_941986	Instituto Nacional de Salud, Bogotá, Colombia	Centro de Investigaciones en Microbiología y Biotecnología-UR (CIMBIUR), Facultad de Ciencias Naturales, Universidad del Rosario, Bogotá, Colombia Instituto Nacional de Salud, Bogotá, Colombia Icahn School of Medicine at Mount Sinai, New York, USA	Adriana van de Guchte; Alberto Paniz-Mondolfi; Ana S. Gonzalez-Reiche; Carolina Flórez; Carolina Hernández; Emilia Mia Sordillo; Hala Alejel Alshammary; Harm van Bakel; Jayeeta Dutta; Juan David Ramírez; Luz Helena Patiño; Marina Muñoz; Matthew M. Hernandez; Nathalia Ballesteros; Sergio Gomez; Viviana Simon; Zenab Khan
EPI_ISL_447804	Instituto Nacional de Salud, Bogotá, Colombia	Grupo de Investigaciones Microbiológicas-UR (GIMUR), Departamento de Biología, Facultad de Ciencias Naturales, Universidad del Rosario, Bogotá, Colombia Instituto Nacional de Salud, Bogotá, Colombia Icahn School of Medicine at Mount Sinai, New York, USA	Adriana Castillo; Alberto Paniz-Mondolfi; Ana S. Gonzalez-Reiche; Angelica Rico; Aníbal A. Teherán; Carolina Florez; Carolina Hernandez; David Martínez; Emilia Mia Sordillo; Esther C. Barros; Harm van Bakel; Jesús E. Jaimes; Juan David Ramírez; Laura Vega; Lisseth Pardo; Marina Muñoz; Martha L. Ospina; Matthew M. Hernandez; Nathalia Ballesteros; Sergio Castañeda; Sergio Gomez; Viviana Simon
EPI_ISL_526967	Instituto Nacional de Salud, Bogotá, Colombia	Instituto Nacional de Salud, Bogotá, Colombia	Astrid C. Flórez; Carlos Andrés Durán; Carlos Franco-Muñoz; Carolina Ferro; Christian Julian Villabona-Arenas; Diana Marcela Walteros-Acero; Diego A. Álvarez-Díaz; Diego Andrés Prada; Franklin Prieto; Jonathan Reales; Jose A. Usme-Ciro; Katherine Laiton-Donato; Liz Villabona-Arenas; Marcela Mercado-Reyes; Martha Lucia Ospina Martinez; Mauricio Pacheco-Montealegre; Sussy Echeverria; Zulma M. Cucunubá
EPI_ISL_2617100, EPI_ISL_2617116, EPI_ISL_2688576, EPI_ISL_2688577, EPI_ISL_2688578	Instituto Nacional de Saude (INS), Mozambique	CERI, Centre for Epidemic Response and Innvoation, Stellenbosch University and KRISP, KZN Research Innovation and Sequencing Platform, UKZN.	Emmanuel S; Giandhari J; Nadia Sitoe; Naidoo Yeshnee; Nalia Ismael; Nedio Mabunda; Paulo Arnaldo; Pillay S; Tegally H; Tshabuila Derek; Wilkinson E; Yajna Ramphal; de Oliveira T
EPI_ISL_887419, EPI_ISL_887429, EPI_ISL_887433, EPI_ISL_887501, EPI_ISL_964947, EPI_ISL_1132836, EPI_ISL_1132840, EPI_ISL_2396914, EPI_ISL_2396917, EPI_ISL_2396929, EPI_ISL_2396934, EPI_ISL_2396950, EPI_ISL_2396994			
see above	Instituto Nacional de Saude (INS), Mozambique	KRISP, KZN Research Innovation and Sequencing Platform	Emmanuel S; Giandhari J; Nadia Sitoe; Nalia Ismael; Nedio Mabunda; Paulo Arnaldo; Pillay S; Tegally H; Wilkinson E; de Oliveira T
EPI_ISL_2843966	Instituto de Biotecnologia - UNESP-Botucatu-SP	Instituto de Biotecnologia - UNESP-Botucatu-SP	Cecília Artico Banho; Cíntia Bittar; Fábio Sossai Possebon; Guilherme Campos; Helena Lage Ferreira; Jorge A. Petroli Marchesi; João Pessoa Araújo Jr.; Leila Sabrina Ullmann; Lívia Sacchetto; Maisa C. Pereira Parra; Marília Moraes; Maurício L. Nogueira; Paula Rahal; Paulo Inacio da Costa
EPI_ISL_516625, EPI_ISL_576266, EPI_ISL_576274, EPI_ISL_658893	Instituto de Diagnostico y Referencia Epidemiologicos (INDRE)	Instituto de Diagnostico y Referencia Epidemiologicos (INDRE)	Abril Rodriguez-Maldonado; Adnan Araiza-Rodriguez; Claudia Wong-Arambula; Dayanira Arellano-Suarez; Edgar Mendieta-Condado; Ernesto Ramirez-Gonzalez; Ernesto Ramirez-Gonzalez.; Fabiola Garces-Ayala; Gisela Barrera-Badillo; Gisela Barrera-Badillo.; Irma Lopez-Martinez; Lucia Hernandez-Rivas; Natividad Cruz-Ortiz; Tatiana Nunez-Garcia
EPI_ISL_452141	Instituto de Diagnostico y Referencia Epidemiologicos (INDRE)	Instituto de diagnóstico y Referencia Epidemiologicos (INDRE)	Araiza-Rodriguez Adnan; Barrera-Badillo Gisela; Garces-Ayala Fabiola; Hernandez-Rivas Lucia; Lopez-Martinez Irma.; Mendieta-Condado Edgar; Ramirez-Gonzalez Ernesto; Rodriguez-Maldonado Abril; Wong-Arambula Claudia
EPI_ISL_1060682, EPI_ISL_1060743, EPI_ISL_1060756, EPI_ISL_1060760, EPI_ISL_1060762	Instituto de Diagnostico y Referencia Epidemiologicos (INDRE)_RNLSP	Instituto de Diagnostico y Referencia Epidemiologicos (INDRE)	Abril Rodriguez-Maldonado; Adnan Araiza-Rodriguez; Claudia Wong-Arambula; David Fragoso-Fonseca; Ernesto Ramirez-Gonzalez.; Fabiola Garces-Ayala; Gisela Barrera-Badillo; Irma Lopez-Martinez; Lucia Hernandez-Rivas; Mayra Jimenez-Morales; Nancy Munoz-Hernandez; Natividad Cruz-Ortiz; Sergio Rangel-Guerrero; Tatiana Nunez-Garcia
EPI_ISL_913933, EPI_ISL_913944, EPI_ISL_913959, EPI_ISL_1054977, EPI_ISL_1168563	Instituto de Diagnostico y Referencia Epidemiologicos INDRE_RNLSP	Instituto de Diagnostico y Referencia Epidemiologicos (INDRE)	Abril Rodriguez-Maldonado; Adnan Araiza-Rodriguez; Ariadna Medina-Benitez; Claudia Wong-Arambula; David Fragoso-Fonseca; Ernesto Ramirez-Gonzalez.; Fabiola Garces-Ayala; Gisela Barrera-Badillo; Irma Lopez-Martinez; Joaquin Quiroz-Mercado; Lucia Hernandez-Rivas; Mayra Jimenez-Morales; Nancy Munoz-Hernandez; Natividad Cruz-Ortiz; Sergio Rangel-Guerrero; Tatiana Nunez-Garcia; Vanessa Rivero-Arredondo
EPI_ISL_1301527, EPI_ISL_1301559, EPI_ISL_1301712	Instituto de Diagnostico y Referencia Epidemiologicos InDRE_RNLSP	Instituto de Biotecnología de la UNAM	Alejandra Hernández-Terán; Alejandro Sanchez-Flores; Alma Rincón-Rubio; Andrea Santos Coy-Arechavaleta; Authors from IBT; Blanca Taboada; Celia Boukadida; Clara Esperanza Santacruz-Tinoco; Edgar Mendieta-Condado; Eduardo Becerril-Vargas; Fidencio Mejía-Nepomuceno; Francisco Pulido; Gisela Barrera-Badillo; Gloria Vazquez; Hector Esteban Paz-Juárez; IMSS; InDRE and INER (in alphabetical order): Carlos F. Arias; Irma Lopez-Martinez; Jerome Jean Verleyen; Joel Armando Vázquez-Pérez; Jorge Salas-Hernández; José Arturo Martínez-Orozco; José Ernesto Ramírez-González; José Esteban Muñoz-Medina; Larissa Fernandes-Matano; Lucia Hernandez-Rivas; Luis Alberto Ochoa-Carrera; Margarita Matías-Florentino; Mario Mújica-Sánchez; Natividad Cruz-Ortiz; Pavel Isa; Ricardo Grande; Santiago Ávila-Ríos; Tatiana Nunez-Garcia; Teresita Rojas-Mendoza
EPI_ISL_2776172, EPI_ISL_2776174, EPI_ISL_2776175, EPI_ISL_2776177, EPI_ISL_2776178, EPI_ISL_2776179, EPI_ISL_2776182, EPI_ISL_2776197			
see above	Instituto de Medicina Tropical & Salud Global (IMTSAG)	Grubaugh Lab - Yale School of Public Health	Alejandaro Vallejo Degaudenzi; Anderson Brito; Annie Watkins; Chaney Kalinich; Chantal Vogels; Elisa Contreras; Esperanza Mendoza; Isabel Ott; Jessica Rothman; Joseph Fauver; Kendall Billig; Mallery Breban; Mary Petrone; Nathan Grubaugh; Robert Paulino-Ramirez; Tara Alpert; Tobias Koch; Victor Virgilio Calderon
EPI_ISL_3020245, EPI_ISL_3020248, EPI_ISL_3020257, EPI_ISL_3020269	Instituto de Medicina Tropical Alexander Von Humboldt, Sede Cusco	Laboratorio de Genómica Microbiana, Universidad Peruana Cayetano Heredia	Alejandra Dávila-Barclay; Diego Cuicapuza; Guillermo Salvatierra; Janet Huancachoque; Luis González; Pablo Tsukayama; Pedro E. Romero; Pool Marcos
EPI_ISL_729916	Instituto de Medicina Tropical, Universidad Nacional Toribio Rodríguez de Mendoza de Amazonas	Laboratorio de Genómica Microbiana, Universidad Peruana Cayetano Heredia	Alejandra Dávila-Barclay; Brenda Ayzanoa; Carla Montenegro; Cecilia Pajuelo; Janet Huancachoque; Luis González; Pablo Tsukayama; Pedro E. Romero; Pool Marcos; Rafael Tapia; Stella Chenet
EPI_ISL_1627995	Instytut Onkologii	1. National Institute of Public Health - National Institute of Hygiene; 2. Eurofins Genomics Europe Sequencing GmbH	ECDC COVID-19 WGS support team; Eurofins Genomics Europe Sequencing Team; Gierczyński Rafał; Sadkowska-Todys Małgorzata; Wołkowicz Tomasz; Zacharczuk Katarzyna
EPI_ISL_1652050, EPI_ISL_1652051, EPI_ISL_1652052, EPI_ISL_1652053, EPI_ISL_1652054, EPI_ISL_1652055	Integrated Biorepository of H3Africa Uganda – IBRH3AU	Molecular Biology Laboratory	Ashaba Fred Katabazi; Bernard Ssentalo Bagaya; David Patrick Kateete; Edgar Kigozi; Emmanuel Nasinghe; Eric Katagirya; Gerald Mboowa; Lwanga Newton; Misaki Wayengera; Moses Joloba; Moses Luutu; Nsubuga Gideon; Rogers Kamulegeya; Samuel Kirimunda; Sarah Stanley; Savannah Mwesigwa
EPI_ISL_1919645, EPI_ISL_1919646	Ipoh Public Health Laboratory (MKAI), Ministry of Health Malaysia	Institute for Medical Research, Infectious Disease Research Centre, National Institutes of Health, Ministry	Kamel K; Mohd Zawawi Z; Suppiah J; Thayan R
EPI_ISL_1919858, EPI_ISL_2650007, EPI_ISL_2815329, EPI_ISL_2815330	Ipoh Public Health Laboratory (MKAI), Ministry of Health Malaysia	Institute for Medical Research, Infectious Disease Research Centre, National Institutes of Health, Ministry of Health Malaysia	Azizan MA; Kamel K; Mohd Zawawi Z; Ramly N; Robert F; Suppiah J; Thayan R
EPI_ISL_2795660, EPI_ISL_3038900	Iran University of Medical Sciences, Tehran, Iran	Genetics Research Center, University of Social Welfare and Rehabilitation Sciences	Farah Bokharaei-Salim; Hossein Najmabadi.; Kimia Kahrizi; Marzieh Mohseni; Seyed Jalal Kiani; Tahereh Donyavi; Zohreh Fattahi
EPI_ISL_1697387	Iressef / LBV	Iressef Genomics lab	Abdou PADANE; Adjiratou Aissatou BA; Alioune TINE; Ambroise AHOUIDI; Aminata DIA; Aminata MBOUP; Anna Julienne Selbe NDIAYE; Assane DIENG; Astou Gaye GAYE; Barada CISSE; Birahim Piere NDIAYE; Gora LO; Khadim GUEYE; Makhtar Camara; Mengue FALL; Moustapha MBOW; Nafisatou LEYE; Ndeye Coumba Toure KANE; OUMY DIOP; Papa Alassane DIAW; Pauline SENE; SADA DIALLO; Serigne Mbaye Lo NDIAYE; Souleymane MBOUP; Yacine DIA
EPI_ISL_2620888, EPI_ISL_2620889, EPI_ISL_2873822, EPI_ISL_2873823, EPI_ISL_2873828, EPI_ISL_2873829, EPI_ISL_2873833, EPI_ISL_2873834, EPI_ISL_2873835, EPI_ISL_2887849, EPI_ISL_2887851, EPI_ISL_2887852, EPI_ISL_2887857			
see above	Iressef Genomics lab	IRESSEF	Abdou PADANE; Ambroise AHOUIDI; Aminata DIA; Aminata MBOUP; Astou Gaye GAYE; Barada CISSE; Birahim Piere NDIAYE; Diabou Diagne; Gora LO; Khadim GUEYE; Moustapha MBOW; Nafisatou LEYE; Ndeye Coumba Toure KANE; Papa Alassane DIAW; Samba Ndiour; Seni Ndiaye; Souleymane MBOUP; Yacine DIA
EPI_ISL_1167146	Iressef Genomics lab	L'institut de Recherche en Santé, de Surveillance Épidémiologique et de Formation (IRESSEF)	Abdou PADANE; Abdoulie KANTEH; Abdul Karim SESAY; Ambroise AHOUIDI; Aminata DIA; Aminata MBOUP; Astou Gaye GAYE; Barada CISSE; Birahim Piere NDIAYE; Gora LO; Khadim GUEYE; Moustapha MBOW; Nafisatou LEYE; Ndeye Coumba Toure KANE; Papa Alassane DIAW; Souleymane MBOUP; Yacine DIA
EPI_ISL_2779373	Isebania Sub County Hospital	USAMRD-A, Basic Science Laboratory	Alan Lemtudo; Beth Mutai; Brian Andika; Carol Kifude; Clement Masakwe; Eric Muthanje; Esther Omuseni; Faith Sigei; Gathii Kimita; George Awinda; John Waitumbi; Josphat Nyataya; Rachel Githii; Rehema Liyai; Stephen Ochola
EPI_ISL_419299, EPI_ISL_419300	Ishikawa Prefectural Institute of Public Health and Environmental Science	Pathogen Genomics Center, National Institute of Infectious Diseases	Eri Nariai; Hajime Kamiya; Kentaro Itokawa; Makoto Kuroda; Masanori Hashino; Motoi Suzuki; Rina Tanaka; Sanae Kuramoto; Tsuyoshi Sekizuka
EPI_ISL_2505234, EPI_ISL_2508151, EPI_ISL_2508154	Islab, Pohjois-Savon aluelaboratorio	Expert Microbiology, National Institute for Health and Welfare	Carita Savolainen-Kopra; Erika Lindh; Haider al-Hello; Jani Halkilahti; Kirsi Liitsola; Niina Ikonen; Olli Vapalahti; Pekka Ellonen; Phuoc Truong; Päivi Laurila; Ravi Kant; Sari Hannula; Soile Blomqvist; Teemu Smura
EPI_ISL_1240650	Israel Central Virology Laboratory	Israel National Consortium for SARS-CoV-2 sequencing	Assaf Rokney; Dana Bar-Ilan; David A. Zeevi; Efrat Dahan Bucris; Efrat Glick-Saar; Efrat Rorman; Ella Mendelson; Ephraim Fass; Eva Nachum; Gal Zizelski Valenci; Gideon Rechavi; Israel Nissan; Joseph Jaffe; Maya Davidovich Cohen; Michal Mandelboim; Mor Rubinstein; Neta Zuckerman; Omer Murik; Omri Nayshool; Oran Erster; Orna Mor; Tzvia Mann
EPI_ISL_474989, EPI_ISL_516889, EPI_ISL_516900, EPI_ISL_575334, EPI_ISL_649094, EPI_ISL_776862	Israel Central Virology laboratory	Israel Central Virology laboratory	Efrat Dahan Bucris; Ella Mendelson; Michal Mandelboim; Neta Zuckerman; Oran Erster; Orna Mor
EPI_ISL_944436, EPI_ISL_1073463, EPI_ISL_1209484, EPI_ISL_1209811, EPI_ISL_2084501, EPI_ISL_2085005, EPI_ISL_2085242, EPI_ISL_2085776, EPI_ISL_2086077, EPI_ISL_2182466, EPI_ISL_2183616, EPI_ISL_2183687, EPI_ISL_2636241			
see above	Israel Central Virology laboratory	Israel National Consortium for SARS-CoV-2 sequencing	Assaf Rokney; Dana Bar-Ilan; David A. Zeevi; Efrat Dahan Bucris; Efrat Glick-Saar; Efrat Rorman; Ella Mendelson; Ephraim Fass; Eva Nachum; Gal Zizelski Valenci; Gideon Rechavi; Israel Nissan; Joseph Jaffe; Maya Davidovich Cohen; Michal Mandelboim; Miranda Geva; Mor Rubinstein; Neta Zuckerman; Netanel Abu; Omer Murik; Omri Nayshool; Oran Erster; Orna Mor; Tzvia Mann
EPI_ISL_1670660	Istituto Zooprofilattico Sperimentale Umbria e Marche "Togo Rosati"	Istituto Zooprofilattico Sperimentale dell'Abruzzo e Molise "G. Caporale"	Ancora M; Biagetti M; Calistri P; Cammà C; Caporale M; Curini V; Delli Compagni E; Di Domenico M; Di Lollo Valeria; Di Pasquale A; Giammarioli M; Lorusso A; Mangone I; Marcacci M; Puglia I; Rinaldi A; Savini G; Scialabba S
EPI_ISL_837247, EPI_ISL_1039312, EPI_ISL_1759528, EPI_ISL_2227133,	Istituto Zooprofilattico Sperimentale del Mezzogiorno	TIGEM	Andrea Ballabio; Anna Manfredi; Antonio Grimaldi; Antonio Grimaldi Patrizia Annunziata Francesco Panariello Biancamaria Pierri Claudia Tiberio Teresa Giuliano Valentina Bouche Chiara Colantuono Maria Concetta Cuomo Denise Di Concilio Lucio Di Filippo Anna Manfredi Marcello Salvi Antonio Limone Luigi Atripaldi Pellegrino Cerino Andrea Ballabio Davide Cacchiarelli; Antonio Limone; Biancamaria Pierri; Chiara Colantuono; Davide Cacchiarelli.; Denise Di Concilio; Francesco Panariello; Lucio Di Filippo; Marcello Salvi; Maria Concetta Cuomo; Patrizia Annunziata; Pellegrino Cerino; Valentina Bouche

EPI_ISL_2455775			
EPI_ISL_1632906, EPI_ISL_2143715, EPI_ISL_2144052	Istituto Zooprofilattico Sperimentale del Mezzogiorno	Telethon Institute of Genetics and Medicine (TIGEM)	Antonio Grimaldi Patrizia Annunziata Francesco Panariello Biancamaria Pierri Claudia Tiberio Teresa Giuliano Valentina Bouche Chiara Colantuono Maria Concetta Cuomo Denise Di Concilio Lucio Di Filippo Anna Manfredi Marcello Salvi Antonio Limone Luigi Atripaldi Pellegrino Cerino Andrea Ballabio Davide Cacchiarelli
EPI_ISL_1390714	Istituto Zooprofilattico Sperimentale del Mezzogiorno - Azienda Ospedaliera "Pugliese Ciaccio" di Catanzaro	TIGEM	Andrea Ballabio; Anna Manfredi; Antonio Grimaldi; Antonio Limone Pasquale Minchella Luigi Atripaldi Pellegrino Cerino; Biancamaria Pierri Claudia Tiberio Valentina Bouche; Chiara Colantuono; Davide Cacchiarelli; Denise Di Concilio; Francesco Panariello; Lucio Di Filippo; Marcello Salvi; Maria Concetta Cuomo; Patrizia Annunziata
EPI_ISL_1298481	Ivano-Frankivsk CCH#1	The Institute of Molecular Biology and Genetics of NASU	M.Tukalo et al.
EPI_ISL_2272918	JIPMER Puducherry	inStem NCBS - INSACOG	Uma Ramakrishnan Dasaradhi Palakodeti Aswin SaiNarain
EPI_ISL_2844844	Jaber Quarantine	Virology Lab, Jaber Al Ahmad Al Sabah Hospital	Adil Afridi; Dr. Ebaa Al-Awadhi; Dr. Zahrah Buhamad; Haroon Masih
EPI_ISL_2984226, EPI_ISL_2984232, EPI_ISL_2984244	Jahra Hospital	Kuwait Cancer Control Center	Mona Alateeqi; Shakir Bahzad
EPI_ISL_450793	Jamaica Ministry of Health and Wellness	Pathogen Discovery, Respiratory Viruses Branch, Division of Viral Diseases, Centers for Disease Control and Prevention	Anna Montmayeur; Anna Uehara; Bettina Bankamp; Clinton R. Paden; Haibin Wang; Jing Zhang; Krista Queen; Rachel Marine; Suxiang Tong; Yan Li; Ying Tao; Zachary Weiner
EPI_ISL_779255, EPI_ISL_779261, EPI_ISL_779263, EPI_ISL_779281, EPI_ISL_779283, EPI_ISL_779284, EPI_ISL_2151345, EPI_ISL_2151346, EPI_ISL_2151365			
see above	Jamil-ur-Rahman Center for Genome Research, Dr. Panjwani Center for Molecular Medicine and Drug Research	Jamil-ur-Rahman Center for Genome Research, Dr. Panjwani Center for Molecular Medicine and Drug Research	A.H.; Ain, N.; Ansari, S.; Hashmi; I.A.; Irfan, M.; Khan; Khan, I.; Nisa, Z.; Rashid, M.; Shakeel, M.; Siddiqui, S.
EPI_ISL_2662827	Jamil-ur-Rahman Center for Genome Research, Dr. Panjwani Center for Molecular Medicine and Drug Research, International Center for Chemical and Biological Sciences, University of Karachi	Jamil-ur-Rahman Center for Genome Research, Dr. Panjwani Center for Molecular Medicine and Drug Research, International Center for Chemical and Biological Sciences, University of Karachi	Ain, Nu.; B.T.; I.A.; Iqbal, W.; Irfan, M.; Jahan, S.; Javed; Kakar, N.; Khan, S.; Nisa, Zu.; Rehman, Z.; S. and Khan; Sarwar, B.; Shakeel, M.; Siddiqi; Zehra, M.
EPI_ISL_2779319, EPI_ISL_2779320, EPI_ISL_3031392, EPI_ISL_3031395, EPI_ISL_3031423, EPI_ISL_3031426, EPI_ISL_3031427, EPI_ISL_3031429, EPI_ISL_3031432, EPI_ISL_3031433			
see above	Jaramogi Oginga Odinga Teaching and Referral Hospital	USAMRD-A, Basic Science Laboratory	Alan Lemtudo; Beth Mutai; Brian Andika; Carol Kifude; Clement Masakwe; Eric Muthanje; Esther Omuseni; Faith Sigei; Gathii Kimita; George Awinda; John Waitumbi; Josphat Nyataya; Rachel Githii; Rehema Liyai; Stephen Ochola
EPI_ISL_3039592	Jessa	Jessa	Berden et al. on behalf of the Jessa_cmdLab
EPI_ISL_2248928	Joaquim Chaves Saude	Instituto Nacional de Saude (INSA)	Borges et al
EPI_ISL_2885164	Johns Hopkins Hospital Department of Pathology	Johns Hopkins Hospital Department of Pathology	Adannaya Amadi; C. Paul Morris; Chun Huai Luo; Heba H. Mostafa; Matthew Schwartz; Nicholas Gallagher
EPI_ISL_2932611, EPI_ISL_2932615, EPI_ISL_2932618	Jordan University of Science Technology/ Irbid filed Hospital/ MOH	Princess Haya Biotechnology Center/ Jordan University of Science Technology	Areej Alquran; Emad Abu Yaqeen; Hazem Haddad; Maha Karam; Saied Jaradat; Shereen Issa; Suha Hasan
EPI_ISL_2271955	Joubert Clinic	National Institute for Communicable Diseases of the National Health Laboratory Service	Amoako DG; Bhiman JN; Ismail A; Mahlangu B; Mohale T; Ntuli N; Scheepers C
EPI_ISL_2779335	KCRH KEMRI-DEID Surveillance Site	USAMRD-A, Basic Science Laboratory	Alan Lemtudo; Beth Mutai; Brian Andika; Carol Kifude; Clement Masakwe; Eric Muthanje; Esther Omuseni; Faith Sigei; Gathii Kimita; George Awinda; John Waitumbi; Josphat Nyataya; Rachel Githii; Rehema Liyai; Stephen Ochola
EPI_ISL_2779337, EPI_ISL_2779455, EPI_ISL_2779456, EPI_ISL_2779547, EPI_ISL_3031383, EPI_ISL_3031385, EPI_ISL_3031407, EPI_ISL_3031413, EPI_ISL_3031417			
see above	KCRH KEMRI/DEID Surveillance Site	USAMRD-A, Basic Science Laboratory	Alan Lemtudo; Beth Mutai; Brian Andika; Carol Kifude; Clement Masakwe; Eric Muthanje; Esther Omuseni; Faith Sigei; Gathii Kimita; George Awinda; John Waitumbi; Josphat Nyataya; Rachel Githii; Rehema Liyai; Stephen Ochola
EPI_ISL_2348630	KEMRI	KEMRI & ICGEB	Alessandro Marcello; Emanuele Orsini; Eric A Lelo; Janet Majanja; Samwel L Symekher; Silvanos M Opanda; Wadegu Meshack; Wallace D Bulimo
EPI_ISL_2602986, EPI_ISL_2602988, EPI_ISL_2602989, EPI_ISL_2602990, EPI_ISL_2602991, EPI_ISL_2602992	KEMRI Center for Biotechnology Research and Development	KEMRI-Wellcome Trust Research Programme,Kilifi	; Githinji G.; Matoke D.; Mburu M.W.; Mohamed K.S.; Onyango C.; de Laurent Z.
EPI_ISL_2602781, EPI_ISL_2602794, EPI_ISL_2602797, EPI_ISL_2602799, EPI_ISL_2602807, EPI_ISL_2602814, EPI_ISL_2602816, EPI_ISL_2602817, EPI_ISL_2602822, EPI_ISL_2602838, EPI_ISL_2602856, EPI_ISL_2602877, EPI_ISL_2602891, EPI_ISL_2602918, EPI_ISL_2602919, EPI_ISL_2602920, EPI_ISL_2602923, EPI_ISL_2602925, EPI_ISL_2602927, EPI_ISL_2602930, EPI_ISL_2602932, EPI_ISL_2602933, EPI_ISL_2602938, EPI_ISL_2602944, EPI_ISL_2602945, EPI_ISL_2602946, EPI_ISL_2602947, EPI_ISL_2602949, EPI_ISL_2602951			
see above	KEMRI-CGHR	KEMRI-Wellcome Trust Research Programme,Kilifi	; Githinji G.; Matoke D.; Mburu M.W.; Mohamed K.S.; Onyango C.; de Laurent Z.
EPI_ISL_457866, EPI_ISL_457876, EPI_ISL_457906	KEMRI-CGMR-C	KEMRI-Wellcome Trust Research Programme/KEMRI-CGMR-C Kilifi	Githinji G. et al 2020
EPI_ISL_1440093, EPI_ISL_1440096, EPI_ISL_1440106, EPI_ISL_1440115, EPI_ISL_1440118, EPI_ISL_1440122, EPI_ISL_1440257, EPI_ISL_1440319, EPI_ISL_1610444, EPI_ISL_1610516, EPI_ISL_1610567, EPI_ISL_2602477, EPI_ISL_2602480, EPI_ISL_2602508, EPI_ISL_2602519, EPI_ISL_2602520, EPI_ISL_2602521, EPI_ISL_2602522, EPI_ISL_2602527, EPI_ISL_2602528, EPI_ISL_2602529, EPI_ISL_2602531, EPI_ISL_2602532, EPI_ISL_2602534, EPI_ISL_2602549, EPI_ISL_2602553, EPI_ISL_2602557, EPI_ISL_2602559, EPI_ISL_2602560, EPI_ISL_2602566, EPI_ISL_2602569, EPI_ISL_2602570, EPI_ISL_2602571, EPI_ISL_2602572, EPI_ISL_2602583, EPI_ISL_2602594, EPI_ISL_2602596, EPI_ISL_2602597, EPI_ISL_2602598, EPI_ISL_2602602, EPI_ISL_2602605, EPI_ISL_2602607, EPI_ISL_2602610, EPI_ISL_2602612, EPI_ISL_2602620, EPI_ISL_2602625, EPI_ISL_2602635, EPI_ISL_2602637, EPI_ISL_2602643, EPI_ISL_2602644, EPI_ISL_2602647, EPI_ISL_2602650, EPI_ISL_2602657, EPI_ISL_2602659, EPI_ISL_2602660, EPI_ISL_2602661, EPI_ISL_2602662, EPI_ISL_2602663, EPI_ISL_2602667, EPI_ISL_2602674, EPI_ISL_2602681, EPI_ISL_2602683, EPI_ISL_2602684, EPI_ISL_2602685, EPI_ISL_2602686, EPI_ISL_2602687, EPI_ISL_2602688, EPI_ISL_2602689, EPI_ISL_2602695, EPI_ISL_2602696, EPI_ISL_2602697, EPI_ISL_2602698, EPI_ISL_2602699, EPI_ISL_2602700, EPI_ISL_2602701, EPI_ISL_2602703, EPI_ISL_2602706, EPI_ISL_2602713, EPI_ISL_2602714, EPI_ISL_2602716, EPI_ISL_2602723, EPI_ISL_2602730, EPI_ISL_2602745, EPI_ISL_2602748, EPI_ISL_2602749, EPI_ISL_2602750, EPI_ISL_2602751, EPI_ISL_2602753, EPI_ISL_2602754, EPI_ISL_2602762, EPI_ISL_2602763, EPI_ISL_2602996, EPI_ISL_2603012, EPI_ISL_2603021, EPI_ISL_2603036, EPI_ISL_2603038, EPI_ISL_2603039, EPI_ISL_2603040, EPI_ISL_2603043, EPI_ISL_2603044, EPI_ISL_2603045, EPI_ISL_2603047, EPI_ISL_2603048, EPI_ISL_2603049, EPI_ISL_2603050, EPI_ISL_2603052, EPI_ISL_2603053, EPI_ISL_2603054, EPI_ISL_2603055, EPI_ISL_2603056, EPI_ISL_2603057, EPI_ISL_2603058, EPI_ISL_2603059, EPI_ISL_2603061, EPI_ISL_2603063, EPI_ISL_2603065, EPI_ISL_2603066, EPI_ISL_2603067, EPI_ISL_2603068, EPI_ISL_2603069, EPI_ISL_2603070, EPI_ISL_2603071, EPI_ISL_2603072, EPI_ISL_2603074, EPI_ISL_2603075, EPI_ISL_2603078, EPI_ISL_2603079, EPI_ISL_2603082, EPI_ISL_2603085, EPI_ISL_2603086, EPI_ISL_2603088, EPI_ISL_2603089, EPI_ISL_2603091, EPI_ISL_2603093, EPI_ISL_2603095, EPI_ISL_2603096, EPI_ISL_2603097, EPI_ISL_2603099, EPI_ISL_2603103, EPI_ISL_2603104, EPI_ISL_2603105, EPI_ISL_2603106, EPI_ISL_2603108, EPI_ISL_2603111, EPI_ISL_2603113, EPI_ISL_2603115, EPI_ISL_2603118, EPI_ISL_2603119, EPI_ISL_2603120, EPI_ISL_2603121			
see above	KEMRI-Wellcome Trust Research Programme,Kilifi	KEMRI-Wellcome Trust Research Programme,Kilifi	; Githinji G.; Matoke D.; Mburu M.W.; Mohamed K.S.; Onyango C.; Thiongo K.; de Laurent Z.; deLaurent Z.
EPI_ISL_568715, EPI_ISL_568863, EPI_ISL_806550, EPI_ISL_806561, EPI_ISL_806574, EPI_ISL_806617, EPI_ISL_806638, EPI_ISL_806643, EPI_ISL_806653, EPI_ISL_806667, EPI_ISL_806704, EPI_ISL_806705, EPI_ISL_855536, EPI_ISL_855537, EPI_ISL_855544, EPI_ISL_855548, EPI_ISL_968817, EPI_ISL_968822, EPI_ISL_968850, EPI_ISL_968922, EPI_ISL_968961, EPI_ISL_968992, EPI_ISL_968993, EPI_ISL_969014, EPI_ISL_969074, EPI_ISL_1039226, EPI_ISL_1039228			
see above	KEMRI-Wellcome Trust Research Programme/KEMRI-CGMR-C Kilifi	KEMRI-Wellcome Trust Research Programme/KEMRI-CGMR-C Kilifi	Githinji et al; Githinji et al 2020
EPI_ISL_2779322, EPI_ISL_2779323	KEMRI/DEID Surveillance Site (Isebani Sub-County Hospital)	USAMRD-A, Basic Science Laboratory	Alan Lemtudo; Beth Mutai; Brian Andika; Carol Kifude; Clement Masakwe; Eric Muthanje; Esther Omuseni; Faith Sigei; Gathii Kimita; George Awinda; John Waitumbi; Josphat Nyataya; Rachel Githii; Rehema Liyai; Stephen Ochola
EPI_ISL_3031404, EPI_ISL_3031405	KEMRI/DEID Surveillance Site (Kombewa Sub County Hospital)	USAMRD-A, Basic Science Laboratory	Alan Lemtudo; Beth Mutai; Brian Andika; Carol Kifude; Clement Masakwe; Eric Muthanje; Esther Omuseni; Faith Sigei; Gathii Kimita; George Awinda; John Waitumbi; Josphat Nyataya; Rachel Githii; Rehema Liyai; Stephen Ochola
EPI_ISL_2779325, EPI_ISL_2779348	KEMRI/DEID Surveillance Site (Lamu County Hospital)	USAMRD-A, Basic Science Laboratory	Alan Lemtudo; Beth Mutai; Brian Andika; Carol Kifude; Clement Masakwe; Eric Muthanje; Esther Omuseni; Faith Sigei; Gathii Kimita; George Awinda; John Waitumbi; Josphat Nyataya; Rachel Githii; Rehema Liyai; Stephen Ochola
EPI_ISL_2779362, EPI_ISL_2779376	KEMRI/DEID Surveillance Site (Mtongwe Navy Base Hospital)	USAMRD-A, Basic Science Laboratory	Alan Lemtudo; Beth Mutai; Brian Andika; Carol Kifude; Clement Masakwe; Eric Muthanje; Esther Omuseni; Faith Sigei; Gathii Kimita; George Awinda; John Waitumbi; Josphat Nyataya; Rachel Githii; Rehema Liyai; Stephen Ochola
EPI_ISL_2382194, EPI_ISL_2382195, EPI_ISL_2382197, EPI_ISL_2382198, EPI_ISL_2382200, EPI_ISL_2382201, EPI_ISL_2382202, EPI_ISL_2382203, EPI_ISL_2382204, EPI_ISL_2382205, EPI_ISL_2382209, EPI_ISL_2382210, EPI_ISL_2382214, EPI_ISL_2382217, EPI_ISL_2382702, EPI_ISL_2382710, EPI_ISL_2382715, EPI_ISL_2382720, EPI_ISL_2382725, EPI_ISL_2582845, EPI_ISL_2582848, EPI_ISL_2582868, EPI_ISL_2582870, EPI_ISL_2582872, EPI_ISL_2582885, EPI_ISL_2582963, EPI_ISL_2582977, EPI_ISL_2662665, EPI_ISL_2662678, EPI_ISL_2662685, EPI_ISL_2662699, EPI_ISL_2662700, EPI_ISL_2984802, EPI_ISL_2984803, EPI_ISL_2984804, EPI_ISL_2984805, EPI_ISL_2984806, EPI_ISL_2984807, EPI_ISL_2984808, EPI_ISL_2984809			
see above	KIMBERLEY LABORATORY	National Institute for Communicable Diseases of the National Health Laboratory Service	Amoako DG; Bhiman JN; Everatt J; Ismail A; Mahlangu B; Mnguni A; Mohale T; Ntuli N; Scheepers C
EPI_ISL_436684, EPI_ISL_455631	KRISP, KZN Research Innovation and Sequencing Platform	KRISP, KZN Research Innovation and Sequencing Platform	Chimukangara B; Deforche K; Giandhari J; Lessells R; Pillay S; Tegally H; Wilkinson E; de Oliveira T
EPI_ISL_2384202, EPI_ISL_2690054	KS Health and Environmental Laboratories	Centers for Disease Control and Prevention Division of Viral Diseases, Pathogen Discovery	Alison Laufer Halpin; Ben L. Rambo-Martin; Clinton R. Paden; Dakota Howard; Darlene Wagner; Dave Wentworth; Dhwani Batra; Jasmine Padilla; Justin Lee; Katie Dillon; Krista Queen; Kristen Knipe; Kristine Lacek; Mark Burroughs; Matthew Schmerer; Mili Sheth; Peter Cook; Sam Shepard; Sarah Nobles; Shoshona Le; Suxiang Tong; Vivien Dugan; Yvette Unoarumhi
EPI_ISL_1048849, EPI_ISL_2424511, EPI_ISL_2425042, EPI_ISL_2425316, EPI_ISL_2833359	KU Leuven, Rega Institute, Clinical and Epidemiological Virology	KU Leuven, Rega Institute, Clinical and Epidemiological Virology	Bert Vanmechelen; Joan Marti-Carerras; Piet Maes; Tony Wawina-Bokalanga
EPI_ISL_1446361	KY State Public Health Lab	Centers for Disease Control and Prevention Division of Viral Diseases, Pathogen Discovery	Alison Laufer Halpin; Ben L. Rambo-Martin; Clinton R. Paden; Dakota Howard; Darlene Wagner; Dave Wentworth; Dhwani Batra; Jasmine Padilla; Justin Lee; Katie Dillon; Krista Queen; Kristen Knipe; Kristine Lacek; Mark Burroughs; Matthew Schmerer; Mili Sheth; Peter Cook; Sam Shepard; Sarah Nobles; Shoshona Le; Suxiang Tong; Vivien Dugan; Yvette Unoarumhi
EPI_ISL_495421	Kafkas University, Faculty of Medicine, Department of Medical Microbiology	Kafkas University, Faculty of Medicine, Department of Medical Microbiology	Didem Ozgur; E. Ediz Tutuncu; Murat Karamese
EPI_ISL_1412522, EPI_ISL_2153683, EPI_ISL_2627909, EPI_ISL_2688192, EPI_ISL_2833876, EPI_ISL_2842088, EPI_ISL_3030691			
see above	Kansas Health and Environmental Lab	Kansas Health and Environmental Lab	Ben Olsen; Jonathan Barnell; Katherine Wiggins; Mike Grose; and Phil Adam
EPI_ISL_2438618	Karasai Central District Hospital	Reference laboratory for the control of viral infections	Aidar Usserbayev; Aknur Mutaliyeva; Andrey Komissarov; Artem Fadeev; Azamat Kenessov; Bekzhan Maikotov; Gaukhar Nussupbayeva; Madina Tleubergenova; Maria Pisareva; Nazym Tleumbetova
EPI_ISL_2365354, EPI_ISL_2365857, EPI_ISL_2835993	Kariminejad-Najmabadi Pathology & Genetics Center, Tehran, Iran	Genetics Research Center, University of Social Welfare and Rehabilitation Sciences	Hossein Najmabadi.; Kimia Kahrizi; Mahdieh Koshki; Maryam Azad; Marzieh Mohseni; Siavash Ghaderi; Zohreh Fattahi

EPI_ISL_475544, EPI_ISL_510835	Karolinska Universitetslaboratoriet	The Public Health Agency of Sweden	Anna Risberg; Anna-Malin Linde; Karin Tegmark-Wisell; Maria Lind Karlberg; Mattias Haukland; Mia Brytting; Olov Svartstrom; Oskar Karlsson Lindsjo; Petra Edquist; Reza Advani; Sandra Broddesson; Shaman Muradrasoli
EPI_ISL_2648222, EPI_ISL_2648231, EPI_ISL_2648235, EPI_ISL_2648238, EPI_ISL_2820291, EPI_ISL_2820294, EPI_ISL_2820302, EPI_ISL_2820303, EPI_ISL_2820309, EPI_ISL_2820310, EPI_ISL_2820316, EPI_ISL_2820322, EPI_ISL_2820329, EPI_ISL_2820330, EPI_ISL_2820381, EPI_ISL_2820385, EPI_ISL_2820396, EPI_ISL_2820402, EPI_ISL_2820411, EPI_ISL_2820412			
see above	Kasane Primary Hospital Laboratory	Botswana Harvard HIV Reference Laboratory	Agnes Karutwaeng; Boitumelo Zuze; Botshelo Radibe; Dorcas Maruapula; Joseph Makhema; Keoratile Ntshambiwa; Legodile Kooepile; Madisa Mine; Mosepele Mosepele; Ontlametse T. Bareng; Roger Shapiro; Rose Munyere; Shahin Lockman; Sikhulile Moyo; Simani Gaseitsiwe; Thongbotho Mphoyakgosi; Wonderful T. Choga
EPI_ISL_754063	Kathmandu University	Nepal Health Research Council	Meghnath Dhimal; Pradip Gyanwali
EPI_ISL_1859725	Kazan Federal University, Academic Consortium	FEDERAL RESEARCH AND CLINICAL CENTER OF PHYSICAL-CHEMICAL MEDICINE OF FEDERAL MEDICAL BIOLOGICAL AGENCY, Academic Consortium	Alexander Manolov; Alexander Pavlenko; Alexander Rumyantsev; Alexander Samoilov; Alexander Troitsky; Alexander Tulichev; Alexander Vasiliev; Alexey Volnukhin; Alla Petrova; Andrey Koritsky; Anna Blagonravova; Anna Varizhuk; Daria Boldyreva; Daria Kharlampieva; Daria Matyushkina; Dmitry Fedorov; Dmitry Pushkar; Ekaterina Moskaleva; Elena Galova; Elena Ilina; Elizaveta Orlova; Galina Trifonova; Gaukhar Yusubalieva; Ilshat Gafurov; Irina Shirokova; Konstantin Ivanov; Konstantin Kolontarev; Ksenia Klimina; Larisa Balykova; Lyubov Rychkova; Nadezhda Kolyshkina; Natalia Pozhenko; Oleg Ogarkov; Pavel Bashkirov; Polina Khromova; Polina Tikhonova; Ramiz Bakhtyev; Raushaniya Gaifullina; Sergey Rumyantsev; Svetlana Solovieva; Vadim Govorun; Valentin Manuvera; Varvara Musarova; Vasily Lazarev; Vladimir Baklaushev; Vladimir Rumyantsev; Vladimir Veselovsky; Vladislav Babenko; Vyacheslav Kuropatkin; Vyacheslav Sinkov; Yulia Galeeva; Yuri Udalov
EPI_ISL_1184116	Kentucky State Public Health Lab	Kentucky State Public Health Lab	Joshua Tobias; Karim George; Matthew Johnson; Rachel Zinner; Rhonda Lucas; Stephanie Lunn; Vaneet Arora; William Grooms
EPI_ISL_1239232, EPI_ISL_1239238, EPI_ISL_1239247	Kimberley Hospital, National Health Laboratory Services, Northern Cape, South Africa	National Institute for Communicable Diseases of the National Health Laboratory Service	Amoako DG; Bhiman JN; Ismail A; Mahlangu B; Mohale T; Ntuli N; Scheepers C
EPI_ISL_3031398	King'ong'o Dispensary	USAMRD-A, Basic Science Laboratory	Alan Lemtudo; Beth Mutai; Brian Andika; Carol Kifude; Clement Masakwe; Eric Muthanje; Esther Omuseni; Faith Sigei; Gathii Kimita; George Awinda; John Waitumbi; Josphat Nyataya; Rachel Githii; Rehema Liyai; Stephen Ochola
EPI_ISL_483550, EPI_ISL_483551	Kingdom of Bahrein Ministry of Health	Erasmus Medical Center	Amjad Ghanem Mohamed; Anne van der Linden; Bas Oude Munnink; Claudia Schapendonk; David Nieuwenhuijse; Ebrahim Shehad; Fatema; Hashmeya Al Wasti; Irina Chestakova; Marion Koopmans; Mark Pronk; Pascal Lexmond; Reina Sikkema; Richard Molenkamp; Stefan van Nieuwkoop; Theo Bestebroer; on behalf of the Dutch national COVID-19 response team.
EPI_ISL_1510532	Klaipeda University Hospital	Vilnius University Hospital Santaros Klinikos, Center of Laboratory Medicine	Daniel Naumovas; Dovile Ezerskyte; Gytis Dudas; Ingrida Olendraite; Laimonas Griskevicius; Ligita Raugaite; Mindaugas Stoskus; Monika Katenaite; Rimvydas Norvilas
EPI_ISL_2693464	Klinika za infektivne bolesti "Dr. Fran Mihaljević"	Hrvatski zavod za javno zdravstvo	Irena Tabain; Ivana Ferenčak
EPI_ISL_654892	Klinisk mikrobiologi	The Public Health Agency of Sweden	Anna Risberg; Anna-Malin Linde; Karin Tegmark-Wisell; Maria Lind Karlberg; Mattias Haukland; Mia Brytting; Olov Svartstrom; Oskar Karlsson Lindsjo; Petra Edquist; Reza Advani; Sandra Broddesson
EPI_ISL_548962	Klinisk mikrobiologi, Region Västerbotten	Unit for Biological Agents, Department for CBRN Defence and Security, Swedish Defence Research Agency	FOI Bioinformatics team
EPI_ISL_419296	Kochi Prefectural Institute of Public Health	Pathogen Genomics Center, National Institute of Infectious Diseases	Akihiko Tokaji; Hajime Kamiya; Kentaro Itokawa; Makoto Kuroda; Masanori Hashino; Motoi Suzuki; Rina Tanaka; Tsuyoshi Sekizuka
EPI_ISL_2815331	Kuala Lumpur General Hospital	Institute for Medical Research, Infectious Disease Research Centre, National Institutes of Health, Ministry of Health Malaysia	Azizan MA; Kamel K; Mohd Zawawi Z; Ramly N; Robert F; Suppiah J; Thayan R
EPI_ISL_2649991	Kudat Hospital	Institute for Medical Research, Infectious Disease Research Centre, National Institutes of Health, Ministry of Health Malaysia	Azizan MA; Kamel K; Mohd Zawawi Z; Ramly N; Robert F; Suppiah J; Thayan R
EPI_ISL_2689954, EPI_ISL_2689960	LA Office of Public Health Laboratories	Centers for Disease Control and Prevention Division of Viral Diseases, Pathogen Discovery	Alison Laufer Halpin; Ben L. Rambo-Martin; Clinton R. Paden; Dakota Howard; Darlene Wagner; Dave Wentworth; Dhwani Batra; Jasmine Padilla; Justin Lee; Katie Dillon; Krista Queen; Kristen Knipe; Kristine Lacek; Mark Burroughs; Matthew Schmerer; Mili Sheth; Peter Cook; Sam Shepard; Sarah Nobles; Shoshona Le; Suxiang Tong; Vivien Dugan; Yvette Unoarumhi
EPI_ISL_2779525	LAB-BMC	USAMRD-A, Basic Science Laboratory	Alan Lemtudo; Beth Mutai; Brian Andika; Carol Kifude; Clement Masakwe; Eric Muthanje; Esther Omuseni; Faith Sigei; Gathii Kimita; George Awinda; John Waitumbi; Josphat Nyataya; Rachel Githii; Rehema Liyai; Stephen Ochola
EPI_ISL_2893835	LABORATOIRE FORTE BIO ST PAUL LES DAX	CNR Virus des Infections Respiratoires - France SUD	Antonin Bal; Bruno Lina; Gregory Destras; Gwendolyne Burfin; Hadrien Regue; Laurence Josset; Martine Valette; Quentin Semanas
EPI_ISL_2278210	LABORATOIRE GCS INTER HOSPITALIER	CHU Purpan - Laboratoire de Virologie - Institut Fédératif de Biologie	Bulach T.; Donnadieu C.; Izopet J.; Latour J.; Milhes M.; Nicot F.; Ranger N.; Salin G.; Tremeaux P.
EPI_ISL_2676632	LABORATOIRE d'ANALYSES de BIOLOGIE MEDICALES	CNR Virus des Infections Respiratoires - France SUD	Antonin Bal; Bruno Lina; Gregory Destras; Gwendolyne Burfin; Hadrien Regue; Laurence Josset; Martine Valette; Quentin Semanas
EPI_ISL_2657871	LABORATORIO CLINICA IMBANACO	Instituto Nacional de Salud- Dirección de Investigación en Salud Pública	Carlos Franco-Muñoz; Carmen Osorio; Diana Malo; Diego A. Álvarez-Díaz; Diego Andrés Prada; Gerardo Santamaría; Hector Alejandro Ruiz-Moreno; Jhonnatan Reales-González; Jorge Rivera; Juan Camilo Martinez; Julian Naizaque; Katherine Laiton-Donato; Lisseth Pardo; Magdalena Wiesner; Marcela Mercado-Reyes; Maria T. Herrera-Sepúlveda; Marta Lopez Blanco; Martha Lucia Ospina Martinez; Paola Rojas; Sergio Gomez; Sheryll Corchuelo; Ángela Alarcon Cruz
EPI_ISL_2834727	LABORATORIO CLINICO HIGUERA ESCALANTE	Instituto Nacional de Salud- Dirección de Investigación en Salud Pública	Carlos Franco-Muñoz; Carmen Osorio; Diana Malo; Diego A. Álvarez-Díaz; Diego Andrés Prada; Gerardo Santamaría; Hector Alejandro Ruiz-Moreno; Jhonnatan Reales-González; Jorge Rivera; Juan Camilo Martinez; Julian Naizaque; Katherine Laiton-Donato; Lisseth Pardo; Magdalena Wiesner; Marcela Mercado-Reyes; Maria T. Herrera-Sepúlveda; Marta Lopez Blanco; Martha Lucia Ospina Martinez; Paola Rojas; Sergio Gomez; Sheryll Corchuelo; Ángela Alarcon Cruz
EPI_ISL_682275	LABORATORIOS LABIN	Inciensa, Instituto Costarricense de Investigación y Enseñanza en Nutrición y Salud	Adriana Godinez; Claudio Soto-Garita; Estela Cordero; Francisco Duarte; Hebleen Porras; Melany Calderon & Pei Ling Chan
EPI_ISL_2893755	LABOSUD	GenBio	Gwladys SEVRIN
EPI_ISL_717909	LACEN RJ - Noel Nutels	Bioinformatics Laboratory / LNCC	Alexandra L Gerber; Amilcar Tanuri; Ana Paula de C Guimarães; Ana Tereza R de Vasconcelos; Andréa Cony Cavalcanti; Carolina M Voloch; Claudia dos Santos Rodrigues; Cynthia C Cardoso; Diana Mariani; Luiz G P de Almeida; Otavio Bustrolini; Ronaldo da Silva F Jr; Terezinha M P P Castiñeira
EPI_ISL_2821315, EPI_ISL_2821319, EPI_ISL_3046170, EPI_ISL_3046275, EPI_ISL_3046313, EPI_ISL_3046320, EPI_ISL_3046326, EPI_ISL_3046345, EPI_ISL_3046346			
see above	LACEN/PE	WallauLab on behalf of Fiocruz COVID-19 Genomic Surveillance Network	Alexandre Freitas da Silva; Antonio Mauro Rezende; Cassia Docena; Constância Flávia Junqueira Ayres; Cássia Docena; Duschinka Ribeiro Duarte Guedes; Elisama Helvecio; Filipe Zimmer Dezordi; Gabriel Luz Wallau; Gustavo Barbosa de Lima; Lais Ceschini Machado; Larissa Krokovsky; Laís Ceschini Machado; Lilian Caroliny Amorim Silva; Marcelo Henrique dos Santos Paiva; Matheus Filgueira Bezerra; Sinval Pinto Brandão Filho
EPI_ISL_2463301, EPI_ISL_2463303	LANCET LABORATORY	National Institute for Communicable Diseases of the National Health Laboratory Service	Amoako DG; Bhiman JN; Everatt J; Glass A; Sisonke Team; Viana R
EPI_ISL_2433867, EPI_ISL_2433906	LANCET, LABORATORIES	National Institute for Communicable Diseases of the National Health Laboratory Service	Amoako DG; Bhiman JN; Ismail A; Mahlangu B; Mohale T; Ntuli N; Scheepers C
EPI_ISL_1935347	LBM CLUSES	CNR Virus des Infections Respiratoires - France SUD	Antonin Bal; Bruno Lina; Bruno Simon; Gregory Destras; Gwendolyne Burfin; Hadrien Regue; Laurence Josset; Martine Valette; Quentin Semanas
EPI_ISL_1788463	LBM DE LA SAUVEGARDE	CNR Virus des Infections Respiratoires - France SUD	Antonin Bal; Bruno Lina; Bruno Simon; Gregory Destras; Gwendolyne Burfin; Hadrien Regue; Laurence Josset; Martine Valette; Quentin Semanas
EPI_ISL_2827783, EPI_ISL_2827787	LDSP - CAQUETA HOSPITAL MALVINAS	Instituto Nacional de Salud- Dirección de Investigación en Salud Pública	Carlos Franco-Muñoz; Carmen Osorio; Diana Malo; Diego A. Álvarez-Díaz; Diego Andrés Prada; Gerardo Santamaría; Hector Alejandro Ruiz-Moreno; Jhonnatan Reales-González; Jorge Rivera; Juan Camilo Martinez; Julian Naizaque; Katherine Laiton-Donato; Lisseth Pardo; Magdalena Wiesner; Marcela Mercado-Reyes; Maria T. Herrera-Sepúlveda; Marta Lopez Blanco; Martha Lucia Ospina Martinez; Paola Rojas; Sergio Gomez; Sheryll Corchuelo; Ángela Alarcon Cruz
EPI_ISL_2674311	LDSP NARIÑO	Instituto Nacional de Salud- Dirección de Investigación en Salud Pública	Carlos Franco-Muñoz; Carmen Osorio; Diana Malo; Diego A. Álvarez-Díaz; Diego Andrés Prada; Gerardo Santamaría; Hector Alejandro Ruiz-Moreno; Jhonnatan Reales-González; Jorge Rivera; Juan Camilo Martinez; Julian Naizaque; Katherine Laiton-Donato; Lisseth Pardo; Magdalena Wiesner; Marcela Mercado-Reyes; Maria T. Herrera-Sepúlveda; Marta Lopez Blanco; Martha Lucia Ospina Martinez; Paola Rojas; Sergio Gomez; Sheryll Corchuelo; Ángela Alarcon Cruz
EPI_ISL_2894632	LDSP QUINDIO	Instituto Nacional de Salud- Dirección de Investigación en Salud Pública	Carlos Franco-Muñoz; Carmen Osorio; Diana Malo; Diego A. Álvarez-Díaz; Diego Andrés Prada; Gerardo Santamaría; Hector Alejandro Ruiz-Moreno; Jhonnatan Reales-González; Jorge Rivera; Juan Camilo Martinez; Julian Naizaque; Katherine Laiton-Donato; Lisseth Pardo; Magdalena Wiesner; Marcela Mercado-Reyes; Maria T. Herrera-Sepúlveda; Marta Lopez Blanco; Martha Lucia Ospina Martinez; Paola Rojas; Sergio Gomez; Sheryll Corchuelo; Ángela Alarcon Cruz
EPI_ISL_3007486	LDSP VILLAVICENCIO - CLINICA PRIMAVERA DE VILLAVICENCIO	Instituto Nacional de Salud- Dirección de Investigación en Salud Pública	Carlos Franco-Muñoz; Carmen Osorio; Diana Malo; Diego A. Álvarez-Díaz; Diego Andrés Prada; Gerardo Santamaría; Hector Alejandro Ruiz-Moreno; Jhonnatan Reales-González; Jorge Rivera; Juan Camilo Martinez; Julian Naizaque; Katherine Laiton-Donato; Lisseth Pardo; Magdalena Wiesner; Marcela Mercado-Reyes; Maria T. Herrera-Sepúlveda; Marta Lopez Blanco; Martha Lucia Ospina Martinez; Paola Rojas; Sergio Gomez; Sheryll Corchuelo; Ángela Alarcon Cruz
EPI_ISL_3024517, EPI_ISL_3024525	LEBOWAKGOMO LABORATORY	National Institute for Communicable Diseases of the National Health Laboratory Service	Amoako DG; Bhiman JN; Everatt J; Ismail A; Mahlangu B; Mnguni A; Mohale T; Ntuli N; Scheepers C
EPI_ISL_2495924, EPI_ISL_2937905	LESP Aguascalientes	Instituto de Diagnostico y Referencia Epidemiologicos (INDRE)	Abril Rodriguez-Maldonado; Ariadna Medina-Benitez; Claudia Wong-Arambula; Ernesto Ramirez-Gonzalez.; Gisela Barrera-Badillo; Irma Lopez-Martinez; Joaquin Quiroz-Mercado; Lucia Hernandez-Rivas; Maribel Gonzalez-Villa; Natividad Cruz-Ortiz; Sergio Rangel-Guerrero; Tatiana Nunez-Garcia; Vanessa Rivero-Arredondo
EPI_ISL_2296064, EPI_ISL_3033354	LESP Baja California	Instituto de Diagnostico y Referencia Epidemiologicos (INDRE)	Abril Rodriguez-Maldonado; Ariadna Medina-Benitez; Claudia Wong-Arambula; Ernesto Ramirez-Gonzalez.; Gisela Barrera-Badillo; Irma Lopez-Martinez; Joaquin Quiroz-Mercado; Lucia Hernandez-Rivas; Maribel Gonzalez-Villa; Natividad Cruz-Ortiz; Sergio Rangel-Guerrero; Tatiana Nunez-Garcia; Vanessa Rivero-Arredondo
EPI_ISL_1651893, EPI_ISL_1821182, EPI_ISL_2533818	LESP Baja California Sur	Instituto de Diagnostico y Referencia Epidemiologicos (INDRE)	Abril Rodriguez-Maldonado; Ariadna Medina-Benitez; Claudia Wong-Arambula; Ernesto Ramirez-Gonzalez.; Gisela Barrera-Badillo; Irma Lopez-Martinez; Joaquin Quiroz-Mercado; Lucia Hernandez-Rivas; Natividad Cruz-Ortiz; Sergio Rangel-Guerrero; Tatiana Nunez-Garcia; Vanessa Rivero-Arredondo
EPI_ISL_2094509, EPI_ISL_2094511, EPI_ISL_2533816, EPI_ISL_2559361	LESP Campeche	Instituto de Diagnostico y Referencia Epidemiologicos (INDRE)	Abril Rodriguez-Maldonado; Ariadna Medina-Benitez; Claudia Wong-Arambula; Ernesto Ramirez-Gonzalez.; Gisela Barrera-Badillo; Irma Lopez-Martinez; Joaquin Quiroz-Mercado; Lucia Hernandez-Rivas; Natividad Cruz-Ortiz; Sergio Rangel-Guerrero; Tatiana Nunez-Garcia; Vanessa Rivero-Arredondo
EPI_ISL_2492494	LESP Chiapas	Instituto de Diagnostico y Referencia Epidemiologicos (INDRE)	Abril Rodriguez-Maldonado; Ariadna Medina-Benitez; Claudia Wong-Arambula; Ernesto Ramirez-Gonzalez.; Gisela Barrera-Badillo; Irma Lopez-Martinez; Joaquin Quiroz-Mercado; Lucia Hernandez-Rivas; Natividad Cruz-Ortiz; Sergio Rangel-Guerrero; Tatiana Nunez-Garcia; Vanessa Rivero-Arredondo
EPI_ISL_2858979	LESP Chihuahua	Instituto de Diagnostico y Referencia Epidemiologicos (INDRE)	Abril Rodriguez-Maldonado; Ariadna Medina-Benitez; Claudia Wong-Arambula; Ernesto Ramirez-Gonzalez.; Gisela Barrera-Badillo; Irma Lopez-Martinez; Joaquin Quiroz-Mercado; Lucia Hernandez-Rivas; Maribel Gonzalez-Villa; Natividad Cruz-Ortiz; Sergio Rangel-Guerrero; Tatiana Nunez-Garcia; Vanessa Rivero-Arredondo
EPI_ISL_1651889, EPI_ISL_2674689, EPI_ISL_2688985	LESP Coahuila	Instituto de Diagnostico y Referencia Epidemiologicos (INDRE)	Abril Rodriguez-Maldonado; Ariadna Medina-Benitez; Claudia Wong-Arambula; Ernesto Ramirez-Gonzalez.; Gisela Barrera-Badillo; Irma Lopez-Martinez; Joaquin Quiroz-Mercado; Lucia Hernandez-Rivas; Natividad Cruz-Ortiz; Sergio Rangel-Guerrero; Tatiana Nunez-Garcia; Vanessa Rivero-Arredondo
EPI_ISL_2920686	LESP Colima	Instituto de Diagnostico y Referencia Epidemiologicos (INDRE)	Abril Rodriguez-Maldonado; Ariadna Medina-Benitez; Claudia Wong-Arambula; Ernesto Ramirez-Gonzalez.; Gisela Barrera-Badillo; Irma Lopez-Martinez; Joaquin Quiroz-Mercado; Lucia Hernandez-Rivas; Natividad Cruz-Ortiz; Sergio Rangel-Guerrero; Tatiana Nunez-Garcia; Vanessa Rivero-Arredondo
EPI_ISL_1661512, EPI_ISL_2296101, EPI_ISL_2476421	LESP Guanajuato	Instituto de Diagnostico y Referencia Epidemiologicos (INDRE)	Abril Rodriguez-Maldonado; Ariadna Medina-Benitez; Claudia Wong-Arambula; Ernesto Ramirez-Gonzalez.; Gisela Barrera-Badillo; Irma Lopez-Martinez; Joaquin Quiroz-Mercado; Lucia Hernandez-Rivas; Natividad Cruz-Ortiz; Sergio Rangel-Guerrero; Tatiana Nunez-Garcia; Vanessa Rivero-Arredondo

EPI_ISL_2663360, EPI_ISL_3033348	LESP Hidalgo	Instituto de Diagnostico y Referencia Epidemiologicos (INDRE)	Abril Rodriguez-Maldonado; Ariadna Medina-Benitez; Claudia Wong-Arambula; Ernesto Ramirez-Gonzalez.; Gisela Barrera-Badillo; Irma Lopez-Martinez; Joaquin Quiroz-Mercado; Lucia Hernandez-Rivas; Maribel Gonzalez-Villa; Natividad Cruz-Ortiz; Sergio Rangel-Guerrero; Tatiana Nunez-Garcia; Vanessa Rivero-Arredondo
EPI_ISL_1821200, EPI_ISL_2094505, EPI_ISL_2533767	LESP Jalisco	Instituto de Diagnostico y Referencia Epidemiologicos (INDRE)	Abril Rodriguez-Maldonado; Ariadna Medina-Benitez; Claudia Wong-Arambula; Ernesto Ramirez-Gonzalez.; Gisela Barrera-Badillo; Irma Lopez-Martinez; Joaquin Quiroz-Mercado; Lucia Hernandez-Rivas; Natividad Cruz-Ortiz; Sergio Rangel-Guerrero; Tatiana Nunez-Garcia; Vanessa Rivero-Arredondo
EPI_ISL_2246830, EPI_ISL_2340892, EPI_ISL_2340899, EPI_ISL_2340901, EPI_ISL_3033443, EPI_ISL_3033447, EPI_ISL_3046115, EPI_ISL_3046118			
see above	LESP Michoacan	Instituto de Diagnostico y Referencia Epidemiologicos (INDRE)	Abril Rodriguez-Maldonado; Ariadna Medina-Benitez; Claudia Wong-Arambula; Ernesto Ramirez-Gonzalez.; Gisela Barrera-Badillo; Irma Lopez-Martinez; Joaquin Quiroz-Mercado; Lucia Hernandez-Rivas; Maribel Gonzalez-Villa; Natividad Cruz-Ortiz; Sergio Rangel-Guerrero; Tatiana Nunez-Garcia; Vanessa Rivero-Arredondo
EPI_ISL_1651907, EPI_ISL_1821146, EPI_ISL_1857248, EPI_ISL_2495935	LESP Morelos	Instituto de Diagnostico y Referencia Epidemiologicos (INDRE)	Abril Rodriguez-Maldonado; Ariadna Medina-Benitez; Claudia Wong-Arambula; Ernesto Ramirez-Gonzalez.; Gisela Barrera-Badillo; Irma Lopez-Martinez; Joaquin Quiroz-Mercado; Lucia Hernandez-Rivas; Natividad Cruz-Ortiz; Sergio Rangel-Guerrero; Tatiana Nunez-Garcia; Vanessa Rivero-Arredondo
EPI_ISL_2158204	LESP Nayarit	Instituto de Diagnostico y Referencia Epidemiologicos (INDRE)	Abril Rodriguez-Maldonado; Ariadna Medina-Benitez; Claudia Wong-Arambula; Ernesto Ramirez-Gonzalez.; Gisela Barrera-Badillo; Irma Lopez-Martinez; Joaquin Quiroz-Mercado; Lucia Hernandez-Rivas; Natividad Cruz-Ortiz; Sergio Rangel-Guerrero; Tatiana Nunez-Garcia; Vanessa Rivero-Arredondo
EPI_ISL_1805485, EPI_ISL_2296059	LESP Oaxaca	Instituto de Diagnostico y Referencia Epidemiologicos (INDRE)	Abril Rodriguez-Maldonado; Ariadna Medina-Benitez; Claudia Wong-Arambula; Ernesto Ramirez-Gonzalez.; Gisela Barrera-Badillo; Irma Lopez-Martinez; Joaquin Quiroz-Mercado; Lucia Hernandez-Rivas; Natividad Cruz-Ortiz; Sergio Rangel-Guerrero; Tatiana Nunez-Garcia; Vanessa Rivero-Arredondo
EPI_ISL_2455897, EPI_ISL_2455901, EPI_ISL_2495928, EPI_ISL_2495953, EPI_ISL_2559289, EPI_ISL_2559309, EPI_ISL_2689824, EPI_ISL_3046095			
see above	LESP Queretaro	Instituto de Diagnostico y Referencia Epidemiologicos (INDRE)	Abril Rodriguez-Maldonado; Ariadna Medina-Benitez; Claudia Wong-Arambula; Ernesto Ramirez-Gonzalez.; Gisela Barrera-Badillo; Irma Lopez-Martinez; Joaquin Quiroz-Mercado; Lucia Hernandez-Rivas; Maribel Gonzalez-Villa; Natividad Cruz-Ortiz; Sergio Rangel-Guerrero; Tatiana Nunez-Garcia; Vanessa Rivero-Arredondo
EPI_ISL_2296087, EPI_ISL_2492495	LESP Quintana Roo	Instituto de Diagnostico y Referencia Epidemiologicos (INDRE)	Abril Rodriguez-Maldonado; Ariadna Medina-Benitez; Claudia Wong-Arambula; Ernesto Ramirez-Gonzalez.; Gisela Barrera-Badillo; Irma Lopez-Martinez; Joaquin Quiroz-Mercado; Lucia Hernandez-Rivas; Natividad Cruz-Ortiz; Sergio Rangel-Guerrero; Tatiana Nunez-Garcia; Vanessa Rivero-Arredondo
EPI_ISL_1821128, EPI_ISL_2663403, EPI_ISL_2689794	LESP Sonora	Instituto de Diagnostico y Referencia Epidemiologicos (INDRE)	Abril Rodriguez-Maldonado; Ariadna Medina-Benitez; Claudia Wong-Arambula; Ernesto Ramirez-Gonzalez.; Gisela Barrera-Badillo; Irma Lopez-Martinez; Joaquin Quiroz-Mercado; Lucia Hernandez-Rivas; Natividad Cruz-Ortiz; Sergio Rangel-Guerrero; Tatiana Nunez-Garcia; Vanessa Rivero-Arredondo
EPI_ISL_2920762, EPI_ISL_3033552	LESP Tabasco	Instituto de Diagnostico y Referencia Epidemiologicos (INDRE)	Abril Rodriguez-Maldonado; Ariadna Medina-Benitez; Claudia Wong-Arambula; Ernesto Ramirez-Gonzalez.; Gisela Barrera-Badillo; Irma Lopez-Martinez; Joaquin Quiroz-Mercado; Lucia Hernandez-Rivas; Maribel Gonzalez-Villa; Natividad Cruz-Ortiz; Sergio Rangel-Guerrero; Tatiana Nunez-Garcia; Vanessa Rivero-Arredondo
EPI_ISL_1821158, EPI_ISL_2246840, EPI_ISL_2455939, EPI_ISL_2455950	LESP Tamaulipas	Instituto de Diagnostico y Referencia Epidemiologicos (INDRE)	Abril Rodriguez-Maldonado; Ariadna Medina-Benitez; Claudia Wong-Arambula; Ernesto Ramirez-Gonzalez.; Gisela Barrera-Badillo; Irma Lopez-Martinez; Joaquin Quiroz-Mercado; Lucia Hernandez-Rivas; Natividad Cruz-Ortiz; Sergio Rangel-Guerrero; Tatiana Nunez-Garcia; Vanessa Rivero-Arredondo
EPI_ISL_2937845	LESP Tlaxcala	Instituto de Diagnostico y Referencia Epidemiologicos (INDRE)	Abril Rodriguez-Maldonado; Ariadna Medina-Benitez; Claudia Wong-Arambula; Ernesto Ramirez-Gonzalez.; Gisela Barrera-Badillo; Irma Lopez-Martinez; Joaquin Quiroz-Mercado; Lucia Hernandez-Rivas; Maribel Gonzalez-Villa; Natividad Cruz-Ortiz; Sergio Rangel-Guerrero; Tatiana Nunez-Garcia; Vanessa Rivero-Arredondo
EPI_ISL_1821163, EPI_ISL_2559279	LESP Veracruz	Instituto de Diagnostico y Referencia Epidemiologicos (INDRE)	Abril Rodriguez-Maldonado; Ariadna Medina-Benitez; Claudia Wong-Arambula; Ernesto Ramirez-Gonzalez.; Gisela Barrera-Badillo; Irma Lopez-Martinez; Joaquin Quiroz-Mercado; Lucia Hernandez-Rivas; Natividad Cruz-Ortiz; Sergio Rangel-Guerrero; Tatiana Nunez-Garcia; Vanessa Rivero-Arredondo
EPI_ISL_2533779, EPI_ISL_2545762, EPI_ISL_3033498	LESP Yucatan	Instituto de Diagnostico y Referencia Epidemiologicos (INDRE)	Abril Rodriguez-Maldonado; Ariadna Medina-Benitez; Claudia Wong-Arambula; Ernesto Ramirez-Gonzalez.; Gisela Barrera-Badillo; Irma Lopez-Martinez; Joaquin Quiroz-Mercado; Lucia Hernandez-Rivas; Maribel Gonzalez-Villa; Natividad Cruz-Ortiz; Sergio Rangel-Guerrero; Tatiana Nunez-Garcia; Vanessa Rivero-Arredondo
EPI_ISL_1752510	LHUB-ULB	LHUB-ULB	Charlotte Michel; Marie Hallin; Olivier Vandenberg
EPI_ISL_1524979, EPI_ISL_2943031	LHUB-ULB	Labo Klinische Biologie, UZA	Basil Britto Xavier; Christine Lammens; Herman Goossens; Ines Verbesselt; Jasmine Coppens; Kathleen Holemans; Marie Le Mercier; Veerle Matheeussen
EPI_ISL_1312503, EPI_ISL_1613434	LIC	Latvian Biomedical Research and Study Centre	Davids Fridmanis; Diana Dusacka; Elina Dimina; Guntars Zarins; Ivars Silamikelis; Janis Klovins; Janis Pjalkovskis; Jurijs Perevoscikovs; Kaspars Megnis; Laila Silamikele; Lauma Freimane; Laura Ansone; Liga Birzniece; Monta Ustinova; Nikita Zrelovs; Reinis Zeltmatis; Uga Dumpis; Una Krumina; Vita Rovite
EPI_ISL_482737, EPI_ISL_482740	LNR National Reference Laboratory, Mohammed VI University of Health Sciences	Medical Biotechnology Laboratory, Rabat Medical and Pharmacy School, Mohammed The Vth University in Rabat	Chakib NEJJARI; Houda BENRAHMA; Idrissa DIAWARA; Imane SMYEJ; Jalil El ATAR; Jalila RAHOUI; Lahcen BELYAMANI and Azeddine IBRAHIMI; Laila SBABOU; Loubna ALLAM; M.W. CHEMAO-ELFIHRI; Meriem LAAMARTI; Mouna OUADGHIRI; Rachid EL JAOUDI; Rachid MENTAG; Rokia LAAMARTI; Saaid AMZAZI; Souad KARTTI
EPI_ISL_889543, EPI_ISL_1913263	LSUHS Emerging Viral Threat Laboratory	Microbial Genome Sequencing Center	Alexander Mijalis; Andrew D. Yurochko; Camille F. Abshire; Christopher G. Kevil; Daniel J. Snyder; Gregory L. Ware; Jennifer L. Carroll; Jeremy P. Kamil; John A. Vanchiere; Maarten Van Diest; Martin J. Sapp; Mohammed N.A. Siddiquey; Rona S. Scott; Vaughn S. Cooper
EPI_ISL_475082, EPI_ISL_717592	Lab voor klinische biologie	Onderzoeksgroep Virologie	Bruno Verhasselt; Hans Nauwynck; Laurens Lambrechts; Linos Vandekerckhove; Marthe Pauwels; Nick Vereecke; Sebastiaan Theuns
EPI_ISL_775596	Lab. Biologi Molekular FKIK Unwar, Bali	National Institute of Health Research and Development	AA; Dewi; HA; HD; Ikawati; KD; KNA; N; Nugraha; Pangesti; Pawestri; Puspa; Puspandari; SR; Setiawaty; Soekarso; Subangkit; T; V
EPI_ISL_1239665	Lab. Biologii molekularnej USK	1. National Institute of Public Health - National Institute of Hygiene; 2. Eurofins Genomics Europe Sequencing GmbH	ECDC COVID-19 WGS support team; Eurofins Genomics Europe Sequencing Team; Gierczyński Rafał; Sadkowska-Todys Małgorzata; Wołkowicz Tomasz; Zacharczuk Katarzyna
EPI_ISL_2844508	Lab. Microbiologia e Virologia Cotugno A.O. dei Colli - Istituto Zooprofilattico Sperimentale del Mezzogiorno	TIGEM	Antonio Grimaldi Patrizia Annunziata Francesco Panariello Biancamaria Pierri Claudia Tiberio Teresa Giuliano Valentina Bouche Chiara Colantuono Maria Concetta Cuomo Denise Di Concilio Lucio Di Filippo Anna Manfredi Marcello Salvi Antonio Limone Luigi Atripaldi Pellegrino Cerino Andrea Ballabio Davide Cacchiarelli
EPI_ISL_1154601	LabKom - Labor Augsburg MVZ GmbH	Robert Koch Institute	
EPI_ISL_1642661, EPI_ISL_2260869, EPI_ISL_2314139	LabKom - Labor Hannover MVZ GmbH	Robert Koch Institute	
EPI_ISL_2260098	LabKom - Labor Mainz MVZ GmbH	Robert Koch Institute	
EPI_ISL_456211, EPI_ISL_456393, EPI_ISL_579106, EPI_ISL_579425, EPI_ISL_637092, EPI_ISL_661258, EPI_ISL_661261, EPI_ISL_661262, EPI_ISL_755631, EPI_ISL_794619, EPI_ISL_877212, EPI_ISL_1016858, EPI_ISL_1016867, EPI_ISL_1082265, EPI_ISL_1172031, EPI_ISL_1315312, EPI_ISL_1469088, EPI_ISL_1469093, EPI_ISL_1469097, EPI_ISL_1469107, EPI_ISL_1621303, EPI_ISL_1621304, EPI_ISL_1621309, EPI_ISL_1621314, EPI_ISL_1621316, EPI_ISL_1621319, EPI_ISL_1904850, EPI_ISL_1904853, EPI_ISL_1967901, EPI_ISL_1967902, EPI_ISL_2103202, EPI_ISL_2406489, EPI_ISL_2406490, EPI_ISL_2406495, EPI_ISL_2650017, EPI_ISL_2650021, EPI_ISL_2650022, EPI_ISL_2964924, EPI_ISL_2964927, EPI_ISL_2964930, EPI_ISL_2964932, EPI_ISL_2964935			
see above	LabPLUS	Institute of Environmental Science and Research (ESR)	Anja Werno; Antje van der Linden; Arlo Upton; Chris Mansell; David Hammer; Dragana Drinkovic; Erasmus Smit; Gary McAuliffe; Hana Sofia Andersson; Hermes Perez; James Ussher; Jill Sherwood; Jing Wang; Joep de Ligt; Josh Freeman; Julia Howard; Juliet Elvy; Lauren Jelly; Mary DeAlmeida; Matt Blakiston; Matt Storey; Matthew Rogers; Max Bloomfield; Michael Addidle; Michelle Balm; Muhammad Faisal; Nikki Freed; Olin Silander; Olivia Stroeven; Rachel Boyle; Sally Roberts; SallyAnn Harbison; Sarah Jefferies; Sharmini Muttaiyah; Susan Morpeth; Susan Taylor; Timothy Blackmore; Vani Sathyendran; Veronica Playle; Virginia Hope; Xiaoyun Ren
EPI_ISL_548013	LabTests	Institute of Environmental Science and Research (ESR)	Anja Werno; Antje van der Linden; Arlo Upton; Chris Mansell; David Hammer; Dragana Drinkovic; Erasmus Smit; Gary McAuliffe; Hana Sofia Andersson; Hermes Perez; James Ussher; Jill Sherwood; Jing Wang; Joep de Ligt; Josh Freeman; Julia Howard; Juliet Elvy; Lauren Jelly; Mary DeAlmeida; Matt Blakiston; Matt Storey; Matthew Rogers; Max Bloomfield; Michael Addidle; Michelle Balm; Muhammad Faisal; Nikki Freed; Olin Silander; Sally Roberts; Sarah Jefferies; Sharmini Muttaiyah; Susan Morpeth; Susan Taylor; Timothy Blackmore; Vani Sathyendran; Veronica Playle; Virginia Hope; Xiaoyun Ren
EPI_ISL_912603, EPI_ISL_1219949, EPI_ISL_1821587, EPI_ISL_2029615, EPI_ISL_2029621, EPI_ISL_2029888, EPI_ISL_2029936, EPI_ISL_2030060, EPI_ISL_2188487, EPI_ISL_2709261			
see above	Labo Analyses Med	National Reference Center for Viruses of Respiratory Infections, Institut Pasteur, Paris	Adamou Lagare; Amaury Vaysse; Angela Brisebarre; Brieuc Gestin; Camille Capel; Christophe Malabat; Corinne Maufrais; Durivault JéRôMe; Emmanuelle Permal; Etienne Simon-Lorière; Frédéric Lemoine; Gael Millot; Louise Lefrançois; Marion Barbet; Maud Vanpeene; Méline Bizard; Patricia Tamby; Sylvaine Bastian; Sylvie Behillil; Sylvie Van der Werf; Sylvie van der Werf; Vincent Enouf
EPI_ISL_2003054	Labolatorium Biologi Molekularnej USK	1. Academic Center for Pathomorphological and Genetic-Molecular Diagnostics ltd, Bialystok, Poland 2. National Institute of Public Health - National Institute of Hygiene, Warsaw, Poland	Anetta Sulewska; Jacek Nikliński; Janusz Dzięcioł; Joanna Kiśluk; Katarzyna Zacharczuk; Konrad Raczkowski; Magdalena Nowakowska; Małgorzata Sadkowska-Todys; Piotr Karabowicz; Piotr Majewski; Przemysław Biecek. Joanna Reszeć; Radosław Charkiewicz; Tomasz Wołkowicz
EPI_ISL_1746802	Labolatorium Biologi Molekularnej USK	1. National Institute of Public Health - National Institute of Hygiene; 2. Eurofins Genomics Europe Sequencing GmbH	ECDC COVID-19 WGS support team; Eurofins Genomics Europe Sequencing Team; Gierczyński Rafał; Sadkowska-Todys Małgorzata; Wołkowicz Tomasz; Zacharczuk Katarzyna
EPI_ISL_2897150	Labolatorium Biologi Molekularnej USK	1. Tricity SARS-CoV-2 sequencing consortium: University of Gdansk, Medical University of Gdansk, Vaxican Ltd., Invicta Ltd. 2. National Institute of Public Health - National Institute of Hygiene, Warsaw, Poland	Celina Cybulska; Karolina Gackowska; Katarzyna Groth; Katarzyna Zacharczuk; Krystyna Bienkowska Szewczyk; Lukasz Rabalski; Maciej Grzybek; Maciej Kosinski; Magdalena Nowakowska; Marcin Lubocki; Małgorzata Sadkowska-Todys; Tomasz Wołkowicz
EPI_ISL_2761448	Labor 28 MVZ GmbH	Robert Koch Institute	
EPI_ISL_2845726	Labor Becker & Kollegen (Standort München)	Robert Koch Institute	
EPI_ISL_2390079	Labor Blackholm MVZ	Robert Koch Institute	
EPI_ISL_1463910, EPI_ISL_1716024, EPI_ISL_1862119, EPI_ISL_1862322, EPI_ISL_2384709	Labor Dr. Fenner und Kollegen	Heinrich Pette Institute, Leibniz Institute for Experimental Virology	Adam Grundhoff; Alexis Robitaille; Johannes Knobloch; Martin Aepfelbacher; Nicole Fischer; Thomas Günther
EPI_ISL_2129685	Labor Dr. Heidrich & Kollegen MVZ GmbH Hamburg	Robert Koch Institute	
EPI_ISL_2125015	Labor Dr. Wisplinghoff - Koln	Robert Koch Institute	
EPI_ISL_1850332	Labor Dr. Wisplinghoff - KÃ¶ln	Robert Koch Institute	
EPI_ISL_1215851,	Labor Dr. Wisplinghoff - Köln	Robert Koch Institute	

EPI_ISL_2266929			
EPI_ISL_803955	Labor Dr. Wisplinghoff - Köln	Robert Koch Institute, Influenza and respiratory viruses FG17 & Bioinformatics MF1, Berlin, Germany	Aleksandar Radonic; Dr. R. Grosser; Marianne Wedde; Oliver Drechsel; Ralf Duerrwald; Rene Kmiecinski; Stefan Kroeger; Stephan Fuchs; Thorsten Wolff
EPI_ISL_3044981	Labor Mönchengladbach MVZ Dr. Stein + Kollegen GbR	Robert Koch Institute	
EPI_ISL_1140227, EPI_ISL_1140343, EPI_ISL_1140360, EPI_ISL_1285209	Labor ZOTZ|KLIMAS; MVZ Düsseldorf-Centrum	Robert Koch Institute	
EPI_ISL_775220	Laboratoire Biolife	Laboratoire de Biotechnologie	Afaf Alaoui; Amina Benouda; Bouchra Belfquih; Hanae Dakka; Lahcen belyamani; Mohamed Chenaoui; Mohammed Walid Chemao Elfihri; Mouna Ouadghiri; Otmane Touzani; Saaid Amzazi and Azeddine Ibrahimi; Tarik Aanniz
EPI_ISL_1508956, EPI_ISL_1575436, EPI_ISL_1575441, EPI_ISL_1675141	Laboratoire Biolim/FSS/UL	Unité Mixte Internationale TransVIHMI (UMI 233 IRD – U1175 INSERM - Université de Montpellier) IRD (Institut de recherche pour le développement)	Abla A. KONOU; Adodo SADJI; Ahidjo AYOUBA; Akoélé SILIADIN; Alassane OURO-MEDELI; Amivi EHLAN; Améyo DORKENOO; Anoumou DAGNRA; Christelle BUTEL; Déléma MABA; Eric DELAPORTE; Issaka Maman; Kokou TEGUENI; Koku AGBODEKA; Laetitia SERRANO; Martine PEETERS; Messanh DOUFFAN; Mireille PRINCE-DAVID; Mounerou SALOU; Sidonie A.M.KAGNISSODE; Sika DOSSIM; Syntyche DEVATCHAGNI; Wembo A. HALATOKO
EPI_ISL_2835003	Laboratoire Central Analyse Médicale mohammedia	Functional Genomic Platform_UATRS_CNRST_Rabat	Amina Barkat; Belayachi Lamiae; Elmostafa El fahime; Hajjaji Mohammed; Hasnae Benkirane; Hassan Aguenaou; Jaafar Heikel; Marouane Melloul; Mouhcine Gardoul; Mouhssine Hemlali; Nadia Touil; Oumaima Ait Si Mohammed; Sanaa Alaoui-Amine; Taha Chouati Omar Askander
EPI_ISL_1905079, EPI_ISL_2318037	Laboratoire Central de Virologie	Laboratoire de Biotechnologie	Abdelomunim Essabbar; Amal Zouaki; Ghizlane EL Amin; Hakima Kabbaj; Lahcen Belyamani and Azeddine Ibrahimi; Mouna Ouadghiri; Myriam Seffar; Naima El Hafidi; Saaïd Amzazi; Tarik Aanniz
EPI_ISL_2978186, EPI_ISL_2978187, EPI_ISL_2978188, EPI_ISL_2978191, EPI_ISL_2978195, EPI_ISL_2978196, EPI_ISL_2978200, EPI_ISL_2978201, EPI_ISL_2978203, EPI_ISL_2978206, EPI_ISL_2978211, EPI_ISL_2978223, EPI_ISL_2978224, EPI_ISL_2978225, EPI_ISL_2978226, EPI_ISL_2978236			
see above	Laboratoire National de Santé Publique, Brazzaville, République du Congo	Pathogen Genomics Lab, National Institute for Biomedical Research (INRB)	Amuri Aziza; Andrew Rambaut; Catherine Pratt; Eddy Kinganda-Lusamaki; Edith Nkwembe; Emmanuel Lokilo Lofiko; Fabien Roch Niama; Francisca Muyembe Mawete; Gabriel Kabamba; Ian Goodfellow; Jean Claude Makangara; Jean-Jacques Muyembe Tamfum; Josh Quick; Matthias Pauthner; Michael Wiley; Nick Loman; Placide Mbala-Kingebeni; Raphael Lumembe; Steve Ahuka-Mundeke; Trevor Bedford
EPI_ISL_459898	Laboratoire National de Sante, Microbiology, Virology	Laboratoire National de Sante, Microbiology, Epidemiology and Microbial Genomics	Anke Wienecke-Baldacchino; Catherine Ragimbeau; Guillaume Fournier; Jessica Tapp; Tamir Abdelrahman; Trung Nguyen Nguyen
EPI_ISL_1972306	Laboratoire National de Santé Publique du Cameroun	Pathogen Genomics Lab, National Institute for Biomedical Research (INRB)	Amuri Aziza; Andrew Rambaut; Catherine Pratt; Eddy Kinganda-Lusamaki; Edith Nkwembe; Emmanuel Lokilo Lofiko; Francisca Muyembe Mawete; Gabriel Kabamba; Ian Goodfellow; Jean Claude Makangara; Jean-Jacques Muyembe Tamfum; Josh Quick; Marie Claire Okomo; Matthias Pauthner; Michael Wiley; Nick Loman; Placide Mbala-Kingebeni; Raphael Lumembe; Steve Ahuka-Mundeke; Trevor Bedford
EPI_ISL_2157560, EPI_ISL_2492505, EPI_ISL_2492517, EPI_ISL_2492525	Laboratoire National de Santé Publique – LNSP (HAITI - LNSP)	Laboratory of Respiratory Viruses and Measles, Oswaldo Cruz Institute, FIOCRUZ	Alice Sampaio Rocha; Ana Carolina Mendonca; Anna Carolina Paixao; Elisa Cavalcante Pereira; Fernando Motta; Ito Journel; Jaques Boncy; Luciana Appolinario; Marilda Siqueira on behalf of the Fiocruz COVID-19 Genomic Surveillance Network; Paola Resende; Patrick Delly; Renata Serrano Lopes; Taina Venas
EPI_ISL_1760554	Laboratoire Professeur Daniel GAHOUMA (LPDG)	Centre de recherches médicales de Lambaréné (CERMEL)	Ayola A. Adegnika; Ayong Moure; Bertrand Lell; Bénédicte Ndeboko; Emilio Skarwan; Georgelin Nguema Ondo; Gédéon P. Manouana; Haruka Abe; Jiro Yasuda; Joel Fleury Djoba Siawaya; Rodrigue Bikangui; Rotimi Myrabelle Avome Houechenou; Samira Zoa-Assoumou; Yuri Ushijima
EPI_ISL_2373998	Laboratoire Virologie Saint Louis APHP	Laboratoire Virologie Saint Louis APHP	Constance Delauguerre; Jérome Le Goff; Linda Feghoul; Marie Laure Chaix; Marie Laure Néré; Maud Salmona; Severine Mercier Delarue; Sophia Achaibou
EPI_ISL_1103576, EPI_ISL_1810947, EPI_ISL_1896699, EPI_ISL_1904886	Laboratoire central de Virologie	Laboratoire de Biotechnologie	Abdelomunim Essabbar; Amal Zouaki; Ghizlane EL Amin; Hakima Kabbaj; Lahcen Belyamani and Azeddine Ibrahimi; Mouna Ouadghiri; Myriam Seffar; Saaïd Amzazi; Tarik Aanniz
EPI_ISL_2227202, EPI_ISL_2227203	Laboratoire d'analyse biomédicale du camp Ouezzin Coulibaly	Centre Muraz	Abdoul-Salam Ouedraogo; Amariane Koné; Armel Poda; Arsène Zongo; Essia Belarbi; Fabian Leendertz; Grit Schubert; Halidou Tinto; Soumeya Ouangraoua; Thérèse Kagoné; Yacouba Sawadogo; Zekiba Tarnagda
EPI_ISL_2820514	Laboratoire de Diagnostic, Centre de Soins 1, Caisse Nationale de Sécurité Sociale (CNSS) de Djibouti	IHU Méditerranée Infection	Anthony Levasseur; Christian A. Devaux.; Didier Raoult; Idil Salah abdillahi; Ikram Omar Osman; Iman Abdillahi Hassan; Jeremy Delerce; Linda Houhamdi; Ludivine Brechar; Mohamed Houmed Aboubaker; Philippe Colson; Pierre-Edouard Fournier; Zeinab Ali Waberi
EPI_ISL_2820520	Laboratoire de Diagnostic, Centre de Soins 1, Caisse Nationale de Sécurité Sociale (CNSS) de Djibouti,	IHU Méditerranée Infection.	Anthony Levasseur; Christian A. Devaux.; Didier Raoult; Idil Salah abdillahi; Ikram Omar Osman; Iman Abdillahi Hassan; Jeremy Delerce; Linda Houhamdi; Ludivine Brechar; Mohamed Houmed Aboubaker; Philippe Colson; Pierre-Edouard Fournier; Zeinab Ali Waberi
EPI_ISL_2820626	Laboratoire de Diagnostic, Centre de Soins 1, Caisse Nationale de Sécurité Sociale (CNSS) de Djibouti.	IHU Méditerranée Infection.	Anthony Levasseur; Christian A. Devaux.; Didier Raoult; Idil Salah abdillahi; Ikram Omar Osman; Iman Abdillahi Hassan; Jeremy Delerce; Linda Houhamdi; Ludivine Brechar; Mohamed Houmed Aboubaker; Philippe Colson; Pierre-Edouard Fournier; Zeinab Ali Waberi
EPI_ISL_660448, EPI_ISL_660452, EPI_ISL_660471, EPI_ISL_660477, EPI_ISL_660478, EPI_ISL_660479, EPI_ISL_660492, EPI_ISL_660499, EPI_ISL_660515, EPI_ISL_2142708, EPI_ISL_2142721, EPI_ISL_2142743			
see above	Laboratoire de Microbiologie CHU Souro Sanou	Centre Muraz	Abdoul-Salam Ouedraogo; Amariane Koné; Armel Poda; Arsène Zongo; Essia Belarbi; Fabian Leendertz; Grit Schubert; Halidou Tinto; Lassana Sangaré; Soumeya Ouangraoua; Thérèse Kagoné; Yacouba Sawadogo; Zekiba Tarnagda
EPI_ISL_2289108	Laboratoire de Microbiologie CHU Souro Sanou	Laboratoire bacteriologie virologie CHUSS	Abdoul-Salam Ouedraogo; Abdoulie Kanteh; Abdul Sesay; Annette Erhart; Armel Poda; François Kiemdé; Halidou Tinto; Mariama Kujabi; Yacouba Sawadogo
EPI_ISL_1116464, EPI_ISL_1116467, EPI_ISL_1116468, EPI_ISL_1118923	Laboratoire de Microbiologie- CHU Habib Bourguiba – Sfax	Laboratoire des Procédés de Criblage Moléculaire et Cellulaire-Centre de Biotechnologie de Sfax	A. and Masmoudi, S.; Abdelmoulah, F.; Abid, N.; Ajili, F.; Aouni, M.; Ben Ayed, I.; Bensaid, M.; Chtourou, A.; Elargoubi, A.; Fki-berrajah, L.; Gaaloul, I.; Gargouri, S.; Hammami, A.; Kamoun, S.; Karray Hakim, H.; Kharat, N.; Mastouri, M.; Mhalla, S.; Nabli, A.; Rebai; Smeti, I.; Souissi, A.; Stambouli, N.; Turki, M.
EPI_ISL_414600	Laboratoire de Virologie Institut de Virologie - INSERM U 1109 Hôpitaux Universitaires de Strasbourg	National Reference Center for Viruses of Respiratory Infections, Institut Pasteur, Paris	Angela Brisebarre; Flora Donati Vincent Enouf; Marion Barbet; Maud Vanpeene; Méline Bizard; Mélnie Albert; Samira Fafi-Kremer; Sylvie Behillil; Sylvie van der Werf
EPI_ISL_825711, EPI_ISL_890133, EPI_ISL_890145, EPI_ISL_890158, EPI_ISL_1366018, EPI_ISL_1379080, EPI_ISL_1400646, EPI_ISL_1400702, EPI_ISL_1400733, EPI_ISL_1403232, EPI_ISL_1403325, EPI_ISL_1403327, EPI_ISL_1523424, EPI_ISL_1534163, EPI_ISL_1696760, EPI_ISL_1696761, EPI_ISL_1696762, EPI_ISL_1696763, EPI_ISL_1696764, EPI_ISL_1696765, EPI_ISL_1696818, EPI_ISL_1696894, EPI_ISL_1696913, EPI_ISL_1697201, EPI_ISL_1697202, EPI_ISL_1697203, EPI_ISL_1697205, EPI_ISL_2007707, EPI_ISL_2007710, EPI_ISL_2293764, EPI_ISL_2293840, EPI_ISL_2293901, EPI_ISL_2388272, EPI_ISL_2390825, EPI_ISL_2390950, EPI_ISL_2390959, EPI_ISL_2821389, EPI_ISL_2821439, EPI_ISL_2821747, EPI_ISL_2822078, EPI_ISL_2822086, EPI_ISL_2822095, EPI_ISL_2822121, EPI_ISL_2822220, EPI_ISL_2822230, EPI_ISL_2822242, EPI_ISL_2822257, EPI_ISL_2822259, EPI_ISL_2824272, EPI_ISL_2824348, EPI_ISL_2824702, EPI_ISL_2824716, EPI_ISL_2824921, EPI_ISL_2824929, EPI_ISL_2970215, EPI_ISL_2970217, EPI_ISL_2970307, EPI_ISL_2970418, EPI_ISL_2970502, EPI_ISL_2970657, EPI_ISL_2975903, EPI_ISL_2976528, EPI_ISL_2976529, EPI_ISL_2976530, EPI_ISL_2976531, EPI_ISL_3144438			
see above	Laboratoire de santé publique du Québec	Laboratoire de santé publique du Québec	Guillaume Bourque; Ioannis Ragoussis; Jesse Shapiro; Mark Lathrop and Michel Roger on behalf of the CoVSeQ research group; Mark Lathrop and Michel Roger on behalf of the CoVSeQ research group (http://covseq.ca/researchgroup); Sandrine Moreira
EPI_ISL_476822, EPI_ISL_476824, EPI_ISL_476826, EPI_ISL_476829, EPI_ISL_476830, EPI_ISL_476831	Laboratoire des Fièvres Hémorragiques Virales du Benin	Charité-Universitätsmedizin Berlin	Anges; Drexler; Jan Felix; Moreira-Soto Andres; Sander Anna-Lena; Yadouleton
EPI_ISL_910672, EPI_ISL_910845, EPI_ISL_1383353	Laboratoire national de sante, Microbiology, Virology	Laboratoire national de sante, Microbiology, Microbial Genomics Platform	Anke Wienecke-Baldacchino; Catherine Ragimbeau; Fatu Djabi; Jessica Tapp; Lise Pignon; Raoul Salmon; Tamir Abdelrahman; Trung Nguyen Nguyen
EPI_ISL_739880, EPI_ISL_740129	Laboratoire national de santé, Microbiology, Virology	Laboratoire national de santé, Microbiology, Epidemiology and Microbial Genomics	Anke Wienecke-Baldacchino; Catherine Ragimbeau; Fatu Djabi; Jessica Tapp; Tamir Abdelrahman; Trung Nguyen Nguyen
EPI_ISL_739901, EPI_ISL_739947, EPI_ISL_740028, EPI_ISL_744139, EPI_ISL_744561, EPI_ISL_744958, EPI_ISL_771034			
see above	Laboratoire national de santé, Microbiology, Virology	Laboratoire national de santé, Microbiology, Microbial Genomics Platform	Anke Wienecke-Baldacchino; Catherine Ragimbeau; Fatu Djabi; Jessica Tapp; Lise Pignon; Raoul Salmon; Tamir Abdelrahman
EPI_ISL_1384323	Laboratoires d'analyses medicales - Ketterthill	Laboratoire national de sante, Microbiology, Microbial Genomics Platform	Anke Wienecke-Baldacchino; Caroline Scheiber; Catherine Ragimbeau; Fatu Djabi; Jessica Tapp; Lise Pignon; Raoul Salmon; Serge Vedy; Tamir Abdelrahman
EPI_ISL_2645438, EPI_ISL_2863801, EPI_ISL_2863816	Laboratorio Central de Saude Publica do Estado do Para (LACEN/PA)	Laboratory of Respiratory Viruses and Measles, Oswaldo Cruz Institute, FIOCRUZ	Agatha Cristinne Prudencio Soares; Alice Sampaio Rocha; Ana Carolina Mendonca; Anna Carolina Paixao; Elisa Cavalcante Pereira; Fernando Motta; Ighor Leonardo Arantes Gomes; Luciana Appolinario; Marilda Siqueira on behalf of the Fiocruz COVID-19 Genomic Surveillance Network; Paola Resende; Renata Serrano Lopes; Taina Venas; Valnete Andrade
EPI_ISL_981041	Laboratorio Central Sección Virología. Hospital Regional Artemides Zatti (Viedma, Rio Negro); Laboratorio MICROBIOM (General Roca, Río Negro); Hospital Zonal Dr. Ramón Carrillo (San Carlos De Bariloche, Río Negro)	Laboratorio Central Mg. Luis Alfredo Pianciola on behalf of 'Proyecto Argentino Interinstitucional de genomica de SARS-CoV-2' (PAIS Consortium)	Antonela De Fino; C Pintos; C Ziehm; Darío Fabián di Prátula; J Ousset; L Pianciola.; Liliana Fonseca; M Fernandez; M Mazzeo; M Nabaes; Marcela Nóbile; María Laura Álvarez; Patricia Valeria Blanco; Silvana Cecchi; Yesica Espasandin
EPI_ISL_1288281, EPI_ISL_1811413, EPI_ISL_1811433, EPI_ISL_1811479	Laboratorio Central de Epidemiología (LCE)	Instituto de Biotecnología de la UNAM	Alejandro Sanchez-Flores; Alfredo Herrera-Estrella; Alicia Ocaña-Mondragón; Angel Gustavo Salas-Lais; Bernardo Martínez-Miguel; Blanca Taboada; Brenda Irasema Maldonado-Meza; Carla Ivón Herrera-Najera; Carlos F. Arias; Celia Boukadida; Clara Esperanza Santacruz-Tinoco; Concepción Grajales-Muñiz; Consorcio Mexicano de Vigilancia Genómica (CoViGen-Mex). Authors (in alphabetical order): Julio Elias Alvarado-Yaah; Célida Duque-Molina; Fernando Fontove-Herrera; Francisco Pulido; Gloria Elena Espinosa-Ayala; Gloria Elena Espinoza-Ayala; Gloria María Molina-Salinas; Gloria Vazquez; Hector Esteban Paz-Juárez; Hector Montoya-Fuentes; Helen Haydee Fernanda Ramírez-Plascencia; Jorge Ivan Salinal-Nevarez; José Antonio Enciso-Moreno; José Esteban Muñoz-Medina; José de Jesús Nuñez-Contreras; Juan Bautista Chale-Dzul; Julio Elias Alvarado-Yaah; Luis Alberto Ochoa-Carrera; Margarita Matías-Florentino; María Guadalupe Santiago-Mauricio; María Guadalupe de Jesús Mireles-Rivera; Nelly Sélem-Mojica; Pavel Isa; Ricardo Grande; Santiago Ávila-Ríos; Victor Eduardo Garcia-Arias; Víctor Hugo Borja-Aburto
EPI_ISL_1301498, EPI_ISL_1302158	Laboratorio Central de Epidemiología IMSS	Instituto de Biotecnología de la UNAM	Alejandra Hernández-Terán; Alejandro Sanchez-Flores; Alma Rincón-Rubio; Andrea Santos Coy-Arechavaleta; Authors from IBT; Blanca Taboada; Celia Boukadida; Clara Esperanza Santacruz-Tinoco; Edgar Mendieta-Condado; Eduardo Becerril-Vargas; Fidencio Mejía-Nepomuceno; Francisco Pulido; Gisela Barrera-Badillo; Gloria Vazquez; Hector Esteban Paz-Juárez; IMSS; InDRE and INER (in alphabetical order): Carlos F. Arias; Irma Lopez-Martinez; Jerome Jean Verleyen; Joel Armando Vázquez-Pérez; Jorge Salas-Hernández; José Arturo Martínez-Orozco; José Ernesto Ramírez-González; José Esteban Muñoz-Medina; Larissa Fernandes-Matano; Lucia Hernandez-Rivas; Luis Alberto Ochoa-Carrera; Margarita Matías-Florentino; Mario Mújica-Sánchez; Natividad Cruz-Ortiz; Pavel Isa; Ricardo Grande; Santiago Ávila-Ríos; Tatiana Nunez-Garcia; Teresita Rojas-Mendoza
EPI_ISL_1492641, EPI_ISL_1492656, EPI_ISL_1492658, EPI_ISL_1492661, EPI_ISL_1492666, EPI_ISL_1492672, EPI_ISL_1492675, EPI_ISL_1492679			
see above	Laboratorio Central de Salud Publica de Paraguay	Laboratorio Central de Salud Publica de Paraguay	Andrea Gómez de la Fuente; Cynthia Vázquez; Flavia Aburjaile; Juan Torales; Luiz Carlos Junior Alcantara; Marta Giovanetti; María José Ortega; María Liz Gamarra; Shirley Villalba; Talita Adelino; Vagner Fonseca
EPI_ISL_2645411	Laboratorio Central de Saude Publica do Estado Maranhao (LACEN-MA)	Laboratory of Respiratory Viruses and Measles, Oswaldo Cruz Institute, FIOCRUZ	Alice Sampaio Rocha; Ana Carolina Mendonca; Anna Carolina Paixao; Elisa Cavalcante Pereira; Fernando Motta; Lidio Gonçalves Lima Neto; Luciana Appolinario; Marilda Siqueira on behalf of the Fiocruz COVID-19 Genomic Surveillance Network; Paola Resende; Renata Serrano Lopes; Taina Venas

EPI_ISL_2645396, EPI_ISL_2645667	Laboratorio Central de Saude Publica do Estado de Alagoas (LACEN/AL)	Laboratory of Respiratory Viruses and Measles, Oswaldo Cruz Institute, FIOCRUZ	Alice Sampaio Rocha; Ana Carolina Mendonca; Anderson Brandao Leite; Anna Carolina Paixao; Elisa Cavalcante Pereira; Fernando Motta; Luciana Appolinario; Marilda Siqueira on behalf of the Fiocruz COVID-19 Genomic Surveillance Network; Paola Resende; Renata Serrano Lopes; Taina Venas
EPI_ISL_2863619	Laboratorio Central de Saude Publica do Estado de Sergipe (LACEN/SE)	Laboratory of Respiratory Viruses and Measles, Oswaldo Cruz Institute, FIOCRUZ	Alice Sampaio Rocha; Ana Carolina Mendonca; Anna Carolina Paixao; Cliomar Alves dos Santos; Elisa Cavalcante Pereira; Fernando Motta; Luciana Appolinario; Marilda Siqueira on behalf of the Fiocruz COVID-19 Genomic Surveillance Network; Paola Resende; Renata Serrano Lopes; Tainá Moreira Martins Venas
EPI_ISL_3045555	Laboratorio Central de Saude Publica do Estado do Espirito Santo (LACEN/ES)	Laboratory of Respiratory Viruses and Measles, Oswaldo Cruz Institute, FIOCRUZ	Alice Sampaio Rocha; Ana Carolina Mendonca; Anna Carolina Paixao; Eilisa Cavalcante Pereira; Fernando Motta; Luciana Appolinario; Marilda Siqueira on behalf of the Fiocruz COVID-19 Genomic Surveillance Network; Paola Resende; Renata Serrano Lopes; Rodrigo Ribeiro Rodrigues; Taina Venas
EPI_ISL_2775494	Laboratorio Central de Saude Publica do Estado do Parana (Instituto de Biologia Molecular do Paraná (LAC)EN-PR)	Instituto Carlos Chagas - Fiocruz	Alessandra De Melo Aguiar; Andreia Akemi Suzukawa; Andréa Rodrigues Ávila; Bruno Dallagiovanna; Dalila Zanette; Eduardo Balsanelli; Emanuel Maltempi de Souza; Fabio Passetti; Fabricio Klerynton Marchini; Fábio de Oliveira Pedrosa; Guilherme Becker; Helisson Faoro; Hellen Geremias dos Santos; Irina Nastassja Riediger; Letusa Albrecht; Lucas Blanes; Luis Gustavo Morello; Lysangela Ronalte Alves; Maria do Carmo Debur; Mauro de Medeiros Oliveira; Michelle Orane Schemberger; Paola Cristina Resende; Sheila Cristina Nardeli; Tiago Gräf; Valter Antônio de Baura
EPI_ISL_2645467, EPI_ISL_2731458	Laboratorio Central de Saude Publica do Estado do Parana (LACEN/PR)	Laboratory of Respiratory Viruses and Measles, Oswaldo Cruz Institute, FIOCRUZ	Alice Sampaio Rocha; Ana Carolina Mendonca; Anna Carolina Paixao; Elisa Cavalcante Pereira; Fernando Motta; Irina Riediger; Luciana Appolinario; Marilda Siqueira on behalf of the Fiocruz COVID-19 Genomic Surveillance Network; Paola Resende; Renata Serrano Lopes; Taina Venas
EPI_ISL_792519	Laboratorio Central de la Ciudad de Santa Fe	Grupo de Genómica y Bioinformática del Instituto de Investigación de la Cadena Láctea CONICET-INTA on behalf of 'Proyecto Argentino Interinstitucional de genomica de SARS-CoV-2' (PAIS Consortium)	AF; Amadio; C; Eberhardt; G; Irazoqui; JM; MF; Mugna; Ojeda; Pastor; Rompato; V
EPI_ISL_2271696	Laboratorio Central, Ministerio de Salud Cordoba	Instituto de Patologia Vegetal (CIAP-INTA) on behalf of 'Proyecto Argentino Interinstitucional de genomica de SARS-CoV-2' (PAIS Consortium)	Barbas, G.; Castro, G.; Debat, HJ.; FD; Fernandez; M; M.B.; Marquez, N.; Pisano; Re, V.
EPI_ISL_792539, EPI_ISL_2940267, EPI_ISL_2940286	Laboratorio Central, Ministerio de Salud Córdoba	Instituto de Patologia Vegetal (CIAP-INTA) on behalf of 'Proyecto Argentino Interinstitucional de genomica de SARS-CoV-2' (PAIS Consortium)	Barbas, G.; Castro, G.; Debat, HJ.; FD; Fernández; M; M.B.; MB; Marquez, N.; Pisano; Re; Re, V.; V
EPI_ISL_424667	Laboratorio Estatal de Salud Publica del Estado de México	Instituto de Diagnóstico y Referencia Epidemiológicos	Adnan Araiza Rodríguez; Alejandro Sánchez; Alfredo Ponce de León Garduño; Blanca Taboada; Carlos F. Arias.; Carolina González Torres; Celia Boukadida; Cesar Raúl González Bonilla; Concepción Grajales Muñiz; Edgar Mendieta Condado; Eduardo Becerril Vargas; Fabiola Garcés Ayala; Fernando Ledesma Barrientos; Francisco Javier Gaytán Cervantes; Francisco Pulido; Gisela Barrera Badillo; Gloria Vázquez; Guillermo M. Ruiz-Palacios; Irma López Martínez; Joel Armando Vázquez Pérez; José Arturo Martínez Orozco; José Ernesto Ramírez González; José Esteban Muñoz Medina; Lucia Hernández Rivas; Luis Alberto García Andrade; Mario Mújica Sánchez; Pavel Isa; Pilar Ramos Cervantes; Ricardo Grande; Santiago Avila Ríos; Victor Hugo Borja Aburto; Violeta Ibarra Gonzalez
EPI_ISL_779189, EPI_ISL_779196	Laboratorio Estatal de Salud Pública de Nuevo León	Laboratorio de Infectología Molecular, Departamento de Bioquímica y Medicina Molecular,Facultad de Medicina - Universidad Autónoma de Nuevo León	Ana M. Rivas-Estilla; Consuelo Treviño-Garza; Daniel Arellanos-Soto; Else del Carmen García-García; Gloria A. Jasso-de-la-Peña; Kame A. Galán-Huerta; Manuel E. de-la-O-Cavazos; María F. Herrera-Saldivar; Natalia Martínez-Acuña; Roberto Montes-de-Oca; Samuel Buentello-Wong; Sonia A. Lozano-Sepúlveda
EPI_ISL_2825105, EPI_ISL_2825117, EPI_ISL_2825135, EPI_ISL_2826967, EPI_ISL_2826996, EPI_ISL_2827807, EPI_ISL_2827824, EPI_ISL_2827828, EPI_ISL_2827835, EPI_ISL_2827848			
see above	Laboratorio Nacional de Salud	Laboratorio Nacional de Salud	Gabriela García; Linda Mendoza
EPI_ISL_837551, EPI_ISL_837579, EPI_ISL_837589	Laboratorio Nacional de Salud	Laboratory of Respiratory Viruses and Measles, Oswaldo Cruz Institute, FIOCRUZ	Ana Carolina Mendonca; Anna Carolina Paixao; Cesar Roberto Conde Pereira; Claudia Estrada; Fernando Motta; Luciana Appolinario; Marilda Siqueira on behalf of the Fiocruz COVID-19 Genomic Surveillance Network; Paola Resende
EPI_ISL_2274010, EPI_ISL_3045384, EPI_ISL_3045410, EPI_ISL_3045422	Laboratorio Nacional de Salud Pública Dr. Defilló - LNSPDD	Laboratory of Respiratory Viruses and Measles, Oswaldo Cruz Institute, FIOCRUZ	Alice Sampaio Rocha; Ana Carolina Mendonca; Anna Carolina Paixao; Elisa Cavalcante Pereira; Fernando Motta; Grey Benoit Vasquez; Isaac Miguel Sanchez; Ivonne Imbert; Lucia de la Cruz; Luciana Appolinario; Marilda Siqueira on behalf of the Fiocruz COVID-19 Genomic Surveillance Network; Nurys de Castro; Paola Resende; Renata Serrano Lopes; Ronald Skewes; Taina Venas
EPI_ISL_2648244, EPI_ISL_2648250, EPI_ISL_2648251, EPI_ISL_2648252, EPI_ISL_2648256, EPI_ISL_2648260, EPI_ISL_2650523, EPI_ISL_2650524, EPI_ISL_2650529, EPI_ISL_2650533, EPI_ISL_2650534, EPI_ISL_2650535, EPI_ISL_2650537			
see above	Laboratorio Nacional de Vigilancia de la Salud – Sección de Virologia	Genomics and Proteomics Departament, Gorgas Memorial Institute For Health Studies	Alexander Martinez; Ambar Moreno; Claudia Díaz; Claudia Gonzalez; Elda Martínez; Jessica Gondola; Leyda Abrego; Marlene Castillo; Mitzi Castro; Nelly Amador; Oris Chavarria; Sandra Paola Paz; Sofia Carolina Alvarado
EPI_ISL_1091264	Laboratorio PGM	Laboratorio de Infectología Molecular, Departamento de Bioquímica y Medicina Molecular,Facultad de Medicina - Universidad Autónoma de Nuevo León	Ana M. Rivas-Estilla; Daniel Arellanos-Soto; Eduardo Garza-de-la-Peña; Gabriela Elizondo; Javier Ramos-Jimenez; Kame A. Galán-Huerta; María F. Herrera-Saldivar; Natalia Martínez-Acuña; Sonia A. Lozano-Sepúlveda
EPI_ISL_1396389	Laboratorio Salud Publica Mendoza	Nodo de Secuenciación Tierra del Fuego - Hospital Regional Ushuaia - Centro Austral De Investigaciones Científicas - Universidad Nacional De Tierra Del Fuego on behalf of 'Proyecto Argentino Interinstitucional de genomica de SARS-CoV-2' (PAIS Consortium)	Aguirre Carolina; Alberto Carena; Alejandro Ezequiel Rojas; Andrea Falaschi; Belén Ortiz; Bosio Lia; Carlos Espul; Cristian Garay; Cristina Fernanda Nardi; Fernando Gallego; Fernando Giuliani; Ivan Dario Gramundi; Luciana Martinez; Maria Belen Peralta Roca; Patricia Robledo; Santiago Guillermo Ceballos; Silvia Zerrer; Silvina Denita
EPI_ISL_2462063	Laboratorio de Biologia Molecular, Hospital San Pedro Claver	Microbiologia Molecular, Instituto SELADIS, Universidad Mayor de San Andrés	Aneth Vasquez Michel; Carmen Delgado Barrera; Oscar M. Rollano-Peñaloza; Sandra Miranda Sardon
EPI_ISL_2600378	Laboratorio de Biologia Molecular, Hospital San Pedro Claver	Molecular Genetics Laboratory, Instituto de Investigaciones Químicas, Universidad Mayor de San Andrés	Aneth Vasquez Michel; Carmen Delgado Barrera; Oscar M. Rollano-Peñaloza; Sandra Miranda Sardon
EPI_ISL_1278284	Laboratorio de Biologia Molecular, SEDES-Potosi	Molecular Genetics Laboratory, Instituto de Investigaciones Químicas, Universidad Mayor de San Andrés	Aneth Vasquez Michel; Oscar M. Rollano-Peñaloza
EPI_ISL_457941, EPI_ISL_457956, EPI_ISL_457965	Laboratorio de Biología Molecular Asociación Española Primera en Salud	Departments of Pathology and Medicine, New York University School of Medicine	Adriana Heguy; Christian Marier; Gael Westby; Gonzalo Manrique; Maria Noel Zubillaga; Maria Victoria Elizondo; Matthew T Maurano; Paul Zappile
EPI_ISL_2427504, EPI_ISL_2427581, EPI_ISL_2427601, EPI_ISL_2427614, EPI_ISL_2427669, EPI_ISL_2427683, EPI_ISL_2427694, EPI_ISL_2427702, EPI_ISL_2427705, EPI_ISL_2427711, EPI_ISL_2427714, EPI_ISL_2427724, EPI_ISL_2427733, EPI_ISL_2427751, EPI_ISL_2427752, EPI_ISL_2427768, EPI_ISL_2427769, EPI_ISL_2427770, EPI_ISL_2427772, EPI_ISL_2427774, EPI_ISL_2427775, EPI_ISL_2427777, EPI_ISL_2427779			
see above	Laboratorio de Biología Molecular Médica Uruguaya	Departments of Pathology and Medicine, New York University School of Medicine	Adriana Heguy; Cecilia Sorhouet; Christian Marier; Dacia Dimartino; Gonzalo Manrique; Maria Cristina Mogdasy; Maria Noel Zubillaga; Maria Victoria Elizondo; Paul Zappile
EPI_ISL_626549	Laboratorio de Biología Molecular, Facultad de Medicina, Universidad de Atacama, Copiapo, Chile/ FONDAP CRG, Universidad Andrés Bello, Santiago, Chile	Center for Mathematical Modeling and Center for Genome Regulation. Santiago, Chile	Allende ML; Bastias M; Castro E; Echeverría C; González M; M; Maass A; Manríquez R; Meneses C.; Montecino; Orellana A; Sanhueza D; Travisany D
EPI_ISL_1068097	Laboratorio de Ecologia de Doencas Transmissiveis na Amazonia, Instituto Leonidas e Maria Deane - Fiocruz Amazonia	Laboratorio de Ecologia de Doencas Transmissiveis na Amazonia, Instituto Leonidas e Maria Deane - Fiocruz Amazonia	André Corado; Debora Duarte; Felipe Naveca on behalf of the Fiocruz COVID-19 Genomic Surveillance Network; Fernanda Nascimento; George Silva; Karina Pessoa; Luciana Gonçalves; Maria Júlia Brandão; Matilde Mejía; Michele Jesus; Valdinete Nascimento; Victor Souza; Ágatha Costa
EPI_ISL_1629797	Laboratorio de Genómica Microbiana, Universidad Peruana Cayetano Heredia	Laboratorio de Genómica Microbiana, Universidad Peruana Cayetano Heredia	Alejandra Dávila-Barclay; Diego Cuicapuza; Guillermo Salvatierra; Janet Huancachoque; Lenin Maturrano; Luis González; Pablo Tsukayama; Pedro E. Romero; Pool Marcos
EPI_ISL_779182, EPI_ISL_961785	Laboratorio de Infectología, Servicio de Infectología, Hospital Universitario Dr. José Eleuterio González - Universidad Autónoma de Nuevo León	Laboratorio de Infectología Molecular, Departamento de Bioquímica y Medicina Molecular,Facultad de Medicina - Universidad Autónoma de Nuevo León	Adrian Camacho-Ortiz; Ana M. Rivas-Estilla; Daniel Arellanos-Soto; Eduardo Perez-Alba; Elvira Garza-González; Kame A. Galán-Huerta; Laura Nuzzolo-Shihadeh; María F. Herrera-Saldivar; Natalia Martínez-Acuña; Paola Bocanegra-Ibarias; Samantha M. Flores-Treviño; Sonia A. Lozano-Sepúlveda
EPI_ISL_953404	Laboratorio de Investigaciones de Baney	"Swiss Tropical and Public Health Institute"	"Carlos Cortes; Bonifacio Manguire Nlavo; Claudia Daubenberger; Diosdado Odjama Nseng Ada; Elizabeth Nyakarungu; Guillermo Garcia; Maximilian Mpina; Mitoha Ondo O Ayekaba; Philip Wonder Phiri"; Salome Hosch; Tobias Schindler
EPI_ISL_1672556, EPI_ISL_2002672, EPI_ISL_2002686	Laboratorio de Investigaciones de Baney	Swiss Tropical and Public Health Institute	Bonifacio Manguire Nlavo; Carlos Cortes; Claudia Daubenberger; Diosdado Odjama Nseng Ada; Elizabeth Nyakarungu; Guillermo Garcia; Maximilian Mpina; Mitoha Ondo O Ayekaba; Philip Wonder Phiri; Philipp Wagner; Salome Hosch; Tobias Schindler; Yahya Maidane
EPI_ISL_648307, EPI_ISL_648318, EPI_ISL_648341, EPI_ISL_648350, EPI_ISL_648356, EPI_ISL_648372, EPI_ISL_648379, EPI_ISL_649171, EPI_ISL_649172			
see above	Laboratorio de Investigaciones de Baney	University Hospital Basel, Clinical Bacteriology	Adrian Egli; Alfredo Mari; Bonifacio Manguire Nlavo; Carlos Cortes; Claudia Daubenberger; Diosdado Odjama Nseng Ada; Elizabeth Nyakarungu; Guillermo Garcia; Helena Seth-Smith; Madlen Stange; Maximilian Mpina; Mitoha Ondo O Ayekaba; Philip Wonder Phiri; Salome Hosch; Tim Roloff; Tobias Schindler
EPI_ISL_2854117, EPI_ISL_2854145	Laboratorio de Medicina Molecular, Universidad de Magallanes	Centro Asistencial Docente y de Investigacion, Universidad de Magallanes	Constanza Ceroni; Diego Alvarez; Jacqueline Aldridge; Jorge González; Marcelo Navarrete; Roberto Uribe-Paredes
EPI_ISL_2462066	Laboratorio de Referencia Departamental en Inmunología, Sedes-Pando	Molecular Genetics Laboratory, Instituto de Investigaciones Químicas, Universidad Mayor de San Andrés	Aneth Vasquez Michel; Carmen Delgado Barrera; Oscar M. Rollano-Peñaloza; Sandra Miranda Sardon
EPI_ISL_524473	Laboratorio de Referencia Nacional de Virus Respiratorio. Centro Nacional de Salud Publica. Instituto Nacional de Salud Peru.	Laboratorio de Referencia Nacional de Biotecnología y Biología Molecular. Centro Nacional de Salud Publica. Instituto Nacional de Salud Peru.	Carlos Padilla Rojas; Henri Bailon Calderon; Johanna Balbuena Torres; Karolyn Vega Chozo; Marco Galarza Perez; Maribel Huaringa Nuñez; Nancy Rojas Serrano.; Omar Caceres Rey; Priscila Lope Pari
EPI_ISL_1575438	Laboratorio de Referencia Nacional de Virus Respiratorio. Instituto Nacional de Salud Peru	Laboratorio de Referencia Nacional de Biotecnología y Biología Molecular. Instituto Nacional de Salud Peru	Carlos Padilla Rojas; Henri Bailon Calderon; Johanna Balbuena Torrez; Karolyn Vega Chozo; Luis Barcena; Marco Galarza Perez; Maribel Huaringa Nuñez; Nancy Rojas Serrano; Omar Caceres Rey; Priscila Lope Pari
EPI_ISL_491429, EPI_ISL_1532220	Laboratorio de Referencia Nacional de Virus Respiratorio. Instituto Nacional de Salud Perú	Laboratorio de Referencia Nacional de Biotecnología y Biología Molecular. Instituto Nacional de Salud Perú	Carlos Padilla Rojas; Henri Bailon Calderon; Johanna Balbuena Torrez; Karolyn Vega Chozo; Luis Barcena; Marco Galarza Perez; Maribel Huaringa Nuñez; Nancy Rojas Serrano; Omar Caceres Rey; Priscila Lope Pari
EPI_ISL_1111143, EPI_ISL_1111223, EPI_ISL_1111249, EPI_ISL_1111480,	Laboratorio de Referencia Nacional de Virus Respiratorio. Instituto Nacional de Salud Perú	Laboratorio de Referencia Nacional de Enteropatógenos. Instituto Nacional de Salud del Perú	Fiorella Orellana Peralta; Iris Silva Molina; Junior Caro Castro; Ronnie Gavilan Chavez; Veronica Hurtado Vela; Willi Quino Sifuentes

EPI_ISL_1111501			
EPI_ISL_729872, EPI_ISL_729879, EPI_ISL_812456	Laboratorio de Referencia Nacional de Virus Respiratorios, Instituto Nacional de Salud Peru	Laboratorio de Genómica Microbiana, Universidad Peruana Cayetano Heredia	Alejandra Dávila-Barclay; Brenda Ayzanoa; Camila Castillo-Vilcahuamán; Guillermo Salvatierra; Janet Huancachoque; Luis González; Marco Galarza; Nancy Rojas; Oscar Escalante; Pablo Tsukayama; Pedro E. Romero; Pool Marcos; Priscila Lope
EPI_ISL_2536706, EPI_ISL_2536739, EPI_ISL_2536758, EPI_ISL_2536790, EPI_ISL_2536791, EPI_ISL_2536794, EPI_ISL_2536840, EPI_ISL_2536845, EPI_ISL_2921339, EPI_ISL_2921345, EPI_ISL_2921359, EPI_ISL_2921372, EPI_ISL_2921373, EPI_ISL_2921385, EPI_ISL_2921427, EPI_ISL_2921474, EPI_ISL_3023412, EPI_ISL_3023495, EPI_ISL_3023554, EPI_ISL_3023587, EPI_ISL_3023618			
see above	Laboratorio de Referencial Nacional de Virus Respiratorios	Laboratorio de Referencial Nacional de Virus Respiratorios	Carlos Padilla Rojas; Henri Bailon Calderon; Iris Silva Molina; Joseph Huayra Niquen; Lely Solari Zerpa; Luis Barcena Flores; Marco Galarza Perez; Nancy Rojas Serrano; Omar Caceres Rey; Orson Mestanza Millones; Priscila Lope Pari; Sandra Morales Ruiz; Steve Acedo Lazo; Veronica Hurtado Vela
EPI_ISL_1738785, EPI_ISL_1738789	Laboratorio de Salud Pública Bogota	Gencore - Universidad de los Andes	Alejandro Gomez; Ana Maria Palacio; David Gonzalez; Gabriela Delgado; Johana Hernandez; Luisa Sacristan; Marcela Guevara; Silvia Restrepo
EPI_ISL_717827	Laboratorio de Virologia Molecular / UFRJ	Bioinformatics Laboratory / LNCC	Alexandra L Gerber; Amilcar Tanuri; Ana Paula de C Guimarães; Ana Tereza R de Vasconcelos; Andréa Cony Cavalcanti; Carolina M Voloch; Claudia dos Santos Rodrigues; Cynthia C Cardoso; Diana Mariani; Luiz G P de Almeida; Otavio Bustrolini; Ronaldo da Silva F Jr; Terezinha M P P Castiñeira
EPI_ISL_792399	Laboratorio de Virología del Hospital de Niños Dr. Ricardo Gutierrez	Biocódices SA. on behalf of 'Proyecto Argentino Interinstitucional de genomica de SARS-CoV-2' (PAIS Consortium)	Acevedo; Alexay; Alvarez Lopez; Berros JM; C; Dopazo H; E; Gravis; Jacques; ME; Mistchenko; O; S; Zubrzycki J
EPI_ISL_1396198, EPI_ISL_1396199, EPI_ISL_2007487	Laboratorio de Virología del Hospital de Niños Dr. Ricardo Gutierrez	Área de Secuenciación del Laboratorio de Virología del Hospital de Niños Dr. Ricardo Gutierrez on behalf of 'Proyecto Argentino Interinstitucional de genomica de SARS-CoV-2' (PAIS Consortium)	A; Acevedo; Acuña; Alexay; Alvarez Lopez; Barreda Frank; C; D; E; G; Goya; Grandis; Jacques; LE; Labarta; Lusso; M; ME; MI; Medina; Mistchenko; N; Nabaes Jodar; Natale; O; S; Streitenberger; Thomas; Valinotto; Viegas, M.; Villegas
EPI_ISL_1760549	Laboratorio di Microbiologia	Laboratorio di Microbiologia	Martinetti Lucchini Gladys; Valeria Spina
EPI_ISL_1239601	Laboratorium Badawczo-Rozwojowe Vitagenum	1. National Institute of Public Health - National Institute of Hygiene; 2. Eurofins Genomics Europe Sequencing GmbH	ECDC COVID-19 WGS support team; Eurofins Genomics Europe Sequencing Team; Gierczyński Rafał; Sadkowska-Todys Małgorzata; Wołkowicz Tomasz; Zacharczuk Katarzyna
EPI_ISL_882960	Laboratorium Diagnostyi Mikrobiologicznej z Pracownią Prątka Gruźlicy SPSzW im. Jana Bożego w Lublinie	National Institute of Public Health - National Institute of Hygiene	Gawor Jan; Wołkowicz Tomasz; Zacharczuk Katarzyna
EPI_ISL_1891882	Laboratorium Mikrobiologiczne Dolnośląskie Centrum Transplantacji Komórkowych z Krajowym Bankiem Dawców Szpiku	1. Academic Center for Pathomorphological and Genetic-Molecular Diagnostics ltd, Bialystok, Poland 2. National Institute of Public Health - National Institute of Hygiene, Warsaw, Poland	Anetta Sulewska; Jacek Nikliński; Janusz Dzięcioł; Joanna Kiśluk; Katarzyna Zacharczuk; Konrad Raczkowski; Magdalena Nowakowska; Małgorzata Sadkowska-Todys; Piotr Karabowicz; Piotr Majewski; Przemysław Biecek. Joanna Reszeć; Radosław Charkiewicz; Tomasz Wołkowicz
EPI_ISL_1612196, EPI_ISL_1681431, EPI_ISL_1681649, EPI_ISL_1681687, EPI_ISL_1685076, EPI_ISL_1799239, EPI_ISL_1800114, EPI_ISL_1802363, EPI_ISL_1802595, EPI_ISL_1926483, EPI_ISL_1928869, EPI_ISL_1931457, EPI_ISL_1931706, EPI_ISL_2045663, EPI_ISL_2184581, EPI_ISL_2305197, EPI_ISL_2305215, EPI_ISL_2305316, EPI_ISL_2305435, EPI_ISL_2305757, EPI_ISL_2306269, EPI_ISL_2398872, EPI_ISL_2398939, EPI_ISL_2480633, EPI_ISL_2481359, EPI_ISL_2481449, EPI_ISL_2481857, EPI_ISL_2611208, EPI_ISL_2611644, EPI_ISL_2872882, EPI_ISL_2872931, EPI_ISL_2927056, EPI_ISL_3017270			
see above	Laboratory Corporation of America	Centers for Disease Control and Prevention Division of Viral Diseases, Pathogen Discovery	Adrian Paskey; Amanda Douglas; Amanda Suchanek; Andrea Throop; Ayla Burns; Benjamin Rambo-Martin; Bobbi Croy; Brian Krueger; Brian Norvell; Christopher Gulvick; Christos Petropoulos; Clinton R. Paden; Craig Lukasik; Dakota Howard; Darlene Wagner; Debbie Boles; Dhwani Batra; Duncan MacCannell; Eyad Almasri; Goran Stevovic; Howard Engler; Hrushikesh Deshmukh; Jake Humphrey; Jana Schroth; Jason Caravas; Joe Voshell; John Pruitt; Jonathan Meltzer; Jonathan Williams; Kara Moser; Kimberly Wagner; Lax Iyer; Lisa Pfefferle; Lyndon Tilson; Manoj Jain; Marcia Eisenberg; Mary Ann Cristobal; Mary Williamson; Matthew Robinson; Matthew Schmerer; Michael Levandoski; Mike Sapeta; Mindy Nye; Minoo Agarwal; Mohan Kolli; Nuthawin Charoensri; Oren Cohen; Peter W. Cook; Prashant Gupta; Qian Zeng; Rama Ghatti; Scott Parker; Scott Ryan; Scott Sammons; Shatavia Morrison; Stanley Letovsky; Steven Ragan; Suresh Babu Selvaraju; Susan Countryman; Susan Hicks; Suzanne Dale; Thomas Urban; Tim Kuphal; Tricia Zwiefelhofer; Vincent Drouillon; Yvette Unoarumhi
EPI_ISL_1021332, EPI_ISL_1026027, EPI_ISL_1029613	Laboratory Corporation of America	Respiratory Viruses Branch, Division of Viral Diseases, Centers for Disease Control and Prevention	Ben L. Rambo-Martin; Clinton R. Paden; Dakota Howard; Dhwani Batra; Duncan MacCannell; Peter W. Cook; Suxiang Tong
EPI_ISL_582530, EPI_ISL_3102050	Laboratory Diagnostic, Veterinary Specialized Institute Kraljevo	Laboratory Diagnostic, Veterinary Specialized Institute Kraljevo	Afonso, C.; Banovic Djeri, B.; Djukic, V.; Jankovic, M.; Jovanovic, T.; Knezevic, A.; Petrovic, T.; Sekler, M.; Tesovic, B.; Vidanovic, D.; Volkening, J.
EPI_ISL_754904, EPI_ISL_754906	Laboratory Diagnostics and Clinical Immunology of Developmental Age, Medical University of Warsaw	genXone SA, Research & Development Laboratory; The Faculty of Mathematics, Informatics and Mechanics of the University of Warsaw	Anna Gambin; Grzegorz Nowicki; Jakub Grabowski; Maciej Sykulski; Michał Kaszuba; Monika Mańkowska-Woźniak; Natalia Drwęska-Matelska; Urszula Demkow; Łukasz Krych
EPI_ISL_667809	Laboratory Medicine	Department of Laboratory Medicine, Lin-Kou Chang Gung Memorial Hospital, Taoyuan, Taiwan	Cheng-Hsun Chiu; Cheng-Ta Yang; Chung-Guei Huang; Guang-Wu Chen; Kuo-Chien Tsao; Kuo-Ming Lee; Mei-Jen Hsiao; Peng-Nien Huang; Po-Wei Huang; Shin-Ru Shih; Shu-Li Yang; Yi-Chun Liu; Yu-Nong Gong
EPI_ISL_2467938	Laboratory for HIV and opportunistic infections diagnosis The Republican Research and Practical Center for Epidemiology and Microbiology (RRPCEM)	Laboratory for HIV and opportunistic infections diagnosis The Republican Research and Practical Center for Epidemiology and Microbiology (RRPCEM	Alina Drozd; Artur Akhremchuk; Elena Gasich; Hanna Gudel; Katsiaryna Belyakova; Kirill Bulda; Leonid Valentovich; Nastassia Kabankova
EPI_ISL_933533, EPI_ISL_1138899, EPI_ISL_1653933, EPI_ISL_2080973	Laboratory for HIV and opportunistic infections diagnosis The Republican Research and Practical Center for Epidemiology and Microbiology (RRPCEM)	Laboratory for HIV and opportunistic infections diagnosis The Republican Research and Practical Center for Epidemiology and Microbiology (RRPCEM)	Alena Mikhalenka; Alexander Kilchevcky; Alina Drozd; Anatoly Krasko; Anna Gudel; Artur Akhremchuk; Elena Gasich; Katsiaryna Belyakova; Kirill Bulda; Leonid Valentovich; Leonid Valentovich; Nastassia Kabankova; Vladimir Gorbunov; Yauhen Sysaliatsin
EPI_ISL_471421	Laboratory for Respiratory Viruses, National Influenza Centre, Cantacuzino National Military-Medical Institute for Research and Development	Cantacuzino Institute	Luiza Ustea; Mihaela Lazar; Nicoleta Paraschiv; Tim Durfee
EPI_ISL_435045	Laboratory of Applied Genetics	RSE "National Center for Biotechnology"	Alexandr Shevtsov; Asylulan Amirgazin; Ilyas Akhmetollayev; Ruslan Kalendar; Viktoriya Lutsay; Yerlan Ramanculov
EPI_ISL_2832105, EPI_ISL_2832106	Laboratory of Genetics and Personalized Medicine, Zan Mitrev Clinic	Laboratory of Genetics and Personalized Medicine, Zan Mitrev Clinic	Kungulovski G. et al.
EPI_ISL_2349110	Laboratory of Immunohematology, Division of Hematology	Greek Genome Center, Biomedical Research Foundation of the Academy of Athens (BRFAA)	Athanasia Mouzaki; Dimitrios Thanos; Emmanouil Athanasiadis; Giannis Vatsellas; Katerina Zoi; Theodoros Loupis
EPI_ISL_2312569, EPI_ISL_2344050, EPI_ISL_2344165	Laboratory of Immunohematology, Division of Hematology	Greek Genome Center, Biomedical Research Foundation of the Academy of Athens (BRFAA)	Athanasia Mouzaki; Dimitrios Thanos; Emmanouil Athanasiadis; Giannis Vatsellas; Katerina Zoi; Theodoros Loupis
EPI_ISL_434456, EPI_ISL_437895	Laboratory of Microbiology, Medical School, National and Kapodistrian University of Athens	Laboratory of Biology, Department of Medicine, Democritus University of Thrace	Bampali, M.; Dovrolis, N.; Froukala, E.; Gatzidou, E.; Kassela K.; N. and Karakasiliotis, I.; Spanakis; Stavropoulou, A.; Tsakris, A.; Veletza, S.
EPI_ISL_654016, EPI_ISL_654017, EPI_ISL_2896981	Laboratory of Microbiology, National Reference Lab, Charles Nicolle Hospital; 2-University of Tunis ElManar, Faculty of Medicine of Tunis, LR99ES09, Tunis, Tunisia	1-Clinical and Experimental Pharmacology Lab, LR16SP02, National Center of Pharmacovigilance, University of Tunis El Manar, Tunis, Tunisia. 2-Neurodegenerative diseases and psychiatric troubles, LR18SP03, Razi Hospital, University of Tunis El Manar, Tunis, Tunisia. 3- Ministry of Health, National Observatory of New and Emerging Diseases, 1006, Tunis, Tunisia	Alia Ben Kahla; Alia Benkahla; Emna Gaies; Gaies Emna; Ilhem Boutiba-Ben Boubaker; Ilhem Boutiba-Ben Boubaker.; Imen Kacem; Imen Mkada; Ines Mdini; Jalila Ben Khelil; Maher Kharrat; Mouna Ben Sassi; Mouna Safer; Nissaf Ben Alaya; Riadh Daghfous; Riadh Gouider; Riadh Gouider.; Roua Ben Othman; Salma Abid; Sameh Trabelsi; Sana Ferjani; Sarra Chammam; Soumaya Rammeh
EPI_ISL_803119, EPI_ISL_2153433, EPI_ISL_2154331	Laboratory of Microbiology, National Reference Lab, Charles Nicolle Hospital; 2-University of Tunis ElManar, Faculty of Medicine of Tunis, LR99ES09, Tunis, Tunisia	Clinical and Experimental Pharmacology Lab, LR16SP02, National Center of Pharmacovigilance, University of Tunis El Manar, Tunis, Tunisia. 2-Neurodegenerative diseases and psychiatric troubles, LR18SP03, Razi Hospital, University of Tunis El Manar, Tunis, Tunisia. 3- Ministry of Health, National Observatory of New and Emerging Diseases, 1006, Tunis, Tunisia	Ahmed Fakhfakh; Alia BenKahla; Gaies Emna; Habiba Ben Romdhane; Hanen El Jebari; Ilhem Boutiba-Ben Boubaker; Ilhem Boutiba-Ben Boubaker.; Imen Kacem; Imen Mdini; Jalila Ben Khelil; Maher Kharrat; Manel Ben Sassi; Mouna Ben Sassi; Mouna Safer; Nissaf Ben Alaya; Raja Mahfoudh; Riadh Daghfous; Riadh Gouider; Riadh Gouider.; Roua Ben Othman; Salma Abid; Sameh Trabelsi; Sana Ferjani; Sarra Chamman; Souissi Amira; Taha Maatoug; Zaineb Hamzaoui
EPI_ISL_2893208, EPI_ISL_2893213, EPI_ISL_2893214, EPI_ISL_2893217, EPI_ISL_2893218, EPI_ISL_2893219, EPI_ISL_2893220			
see above	Laboratory of Molecular Biology and Cancer Immunology, Faculty of Sciences, Lebanese University	Microbial Pathogenomics Lab - LAU	Bassam Badran; Fadi Abdel Sater; Georgi Merhi; Hamad Hassan; Jad Koweyes; Rawan Makki; Sima tokajian
EPI_ISL_450746	Laboratory of Molecular Biology, Diagnostyka sp. z o.o.	Laboratory of Recombinant Vaccines	Anna Piotrowska-Mietelska; Boguslaw Szewczyk; Krystyna Bienkowska-Szewczyk; Lukasz Rabalski; Maciej Kosinski
EPI_ISL_2301773, EPI_ISL_2301776, EPI_ISL_2312615	Laboratory of Molecular Biology, Mamatsio General Hospital of Kozani	Greek Genome Center, Biomedical Research Foundation of the Academy of Athens (BRFAA)	Dimitrios Thanos; Emmanouil Athanasiadis; Giannis Vatsellas; Katerina Zoi; Konstantina Gartzonika; Theodoros Loupis
EPI_ISL_801607, EPI_ISL_801609	Laboratory of Molecular Virology, Pontificia Universidad Católica de Chile	MSHS Pathogen Surveillance Program	Adolfo Garcia-Sastre; Adriana van De Guchte; Ajay Obla; Ana Maria Contreras; Ana S. Gonzalez-Reiche; Bremy Alburquerque; Carlos Palma; Constanza Maldonado; Edward C. Holmes; Eileen Serrano; Erick Salinas; Hala Alshammary; Harm van Bakel; Jayeeta Dutta; Jorge Levican; Juan Soto; Leonardo I. Almonacid; M. Belen Leyton; Marcela Ferres; Matthew M. Hernandez; Melissa Smith; Rafael A. Medina.; Robert Sebra; Shwetha Hara Sridhar; Tamara García-Salum; Viviana Simon; Ying-Chih Wang; Zenab Khan
EPI_ISL_2557397	Laboratory of Respiratory Viruses and Measles, Oswaldo Cruz Institute, FIOCRUZ	Laboratory of Respiratory Viruses and Measles, Oswaldo Cruz Institute, FIOCRUZ	Alice Sampaio Rocha; Ana Carolina Mendonca; Anna Carolina Paixao; Elisa Cavalcante Pereira; Fernando Motta; Luciana Appolinario; Marilda Siqueira on behalf of the Fiocruz COVID-19 Genomic Surveillance Network; Paola Resende; Renata Serrano Lopes; Taina Venas
EPI_ISL_1082301, EPI_ISL_1082303	Laboratory of Virology of Federal Budget Health Care Institution "Center of Hygiene and Epidemiology in Arkhangelsk region"	Group of Genomics and Postgenomic Technologies of Central Research Institute of Epidemiology	Akimkin VG; Bulanenko VP; Cherkashina AS; Golubeva AG; Kaptelova VV; Kondrasheva LV; Korneenko EV; Ponomareva YA; Saenko SS; Samoilov AE; Semyina LV; Shishko LA; Speranskaya AS; Tivanova EV; Valdokhina AV
EPI_ISL_1341503, EPI_ISL_1489929	Laboratory of Virology, National center of expertise	RSE "National Center of Expertise" and RSE "National center for Biotechnology"	Abdaliyev Askar; Amirgazin Asylulan; Balykbaev Kanat; Kamalova Dinara; Ramankulov Erlan; Sharipova Saule; Shevtsov Alexandr; Tungushbayev Talgat

EPI_ISL_1191740	Laboratory of virology and molecular diagnostics	Laboratory of virology and molecular diagnostics	Boshevska Golubinka; Janchevska Elizabeta; Kuzmanovska Maja
EPI_ISL_2392161, EPI_ISL_2681400, EPI_ISL_2681403, EPI_ISL_2681404, EPI_ISL_2681431, EPI_ISL_2681436, EPI_ISL_2681438, EPI_ISL_2987553, EPI_ISL_2987557, EPI_ISL_2987571, EPI_ISL_2987574			
see above	Laboratory of virology and molecular diagnostics, Institute of Public Health	Laboratory of virology and molecular diagnostics, Institute of Public Health	Boshevska G; Janchevska E.; Kuzmanovska M
EPI_ISL_454585	Laboratory of virology, National Center of Expertise	Laboratory of molecular-genetic research, National Center for Expertise, Kazakhstan National Center for Biotechnology, Kazakhstan	; Abdaliyev Askar; Akhmetollayev Ilyas; Amirgazin Asylulan; Aushakhmetova Zabira; Kalendar Ruslan; Lutsay Viktoriya; Rakhmetova Akbota; Ramankulov Yerlan; Shevtsov Alexandr
EPI_ISL_1364615, EPI_ISL_1448018	Laboratory of virology, National center of expertise	RSE "National Center for Biotechnology" and RSE "National Center of Expertise"	Abdaliyev Askar; Amirgazin Asylulan; Balykbaev Kanat; Kamalova Dinara; Ramankulov Yerlan; Sharipova Saule; Shevtsov Alexandr; Tungushbayev Talgat
EPI_ISL_2696437, EPI_ISL_2696689	Laboratory, Gravity Diagnostics	Laboratory, Gravity Diagnostics	Diagnostics, G.
EPI_ISL_576119	Laboratory, The Bio Arte Limited	Laboratory, The Bio Arte Limited	Biazzo, M.; Madeddu, S.; Pinzauti, D.; Santoro, F.
EPI_ISL_2157506	Laboratório Central de Saude Publica do Estado do Paraná (LACEN/PR)	Laboratory of Respiratory Viruses and Measles, Oswaldo Cruz Institute, FIOCRUZ	Alice Sampaio Rocha; Ana Carolina Mendonca; Anna Carolina Paixao; Elisa Cavalcante Pereira; Fernando Motta; Irina Nastassja Riediger; Luciana Appolinario; Maria do Carmo Debur; Marilda Siqueira on behalf of the Fiocruz COVID-19 Genomic Surveillance Network; Paola Resende; Renata Serrano Lopes; Taina Venas
EPI_ISL_2777330, EPI_ISL_2777341, EPI_ISL_2777778, EPI_ISL_2777954	Laboratório Central de Saúde Pública do Amazonas - LACEN-AM	Laboratorio de Ecologia de Doencas Transmissiveis na Amazonia, Instituto Leonidas e Maria Deane - Fiocruz Amazonia	André Corado; Debora Duarte; Felipe Naveca; Fernanda Nascimento; George Silva; Karina Pessoa; Luciana Gonçalves; Maria Júlia Brandão; Matilde Mejía; Michele Jesus; Valdinete Nascimento; Victor Souza; Ágatha Costa
EPI_ISL_541387	Laboratório Central de Saúde Pública do Estado de Sergipe (LACEN-SE)	Laboratory of Respiratory Viruses and Measles, Oswaldo Cruz Institute, FIOCRUZ	Ana Carolina Mendonça; Anna Carolina Paixão; Clioma Santos; Fernando Motta; Jonathan Lopes; Luciana Appolinario; Marilda Siqueira on behalf of the Fiocruz COVID-19 Genomic Surveillance Network; Paola Resende
EPI_ISL_415128	Laboratório Central de Saúde Pública do Estado do Espírito Santo (LACEN-ES)	Laboratory of Respiratory Viruses and Measles, Oswaldo Cruz Institute, FIOCRUZ	Aline Mattos; Allison Fabri; Braulia Caetano; Cristiana Garcia; Fernando Motta; Joilson Xavier; Jonathan Lopes; Luciana Appolinario; Maria Nóbrega; Maria Ogrzewalska; Marilda Siqueira on behalf of the Fiocruz COVID-19 Genomic Surveillance Network; Milene Miranda; Paola Resende; Sunando Roy
EPI_ISL_729832	Laboratório Central de Saúde Pública do Estado do Rio Grande do Sul (LACEN-RS)	Laboratory of Respiratory Viruses and Measles, Oswaldo Cruz Institute, FIOCRUZ	Ana Carolina Mendonça; Anna Carolina Paixão; Fernando Motta; Luciana Appolinario; Marilda Siqueira on behalf of the Fiocruz COVID-19 Genomic Surveillance Network; Marilda Tereza Mar da Rosa; Paola Resende; Tatiana Schaffer Gregianini
EPI_ISL_2150977, EPI_ISL_2158597, EPI_ISL_2158598, EPI_ISL_2158601	Laboratório de Biologia Molecular Jean Piaget	MRCG at LSHTM, Genomics lab	Abdoulie Kanteh; Abdul Karim Sesay; Adul Candé; Aicha Balde; Aladje Balde; Bakary Sanyang; Bubacar Delgado Pinto Embaló; Dabiri Damilari; Edmira Maria da Costa; Erica Luis Maria Magalhães; Faatu Cassama; Mariama Kujabi; Milanca Agostinho Cá; Paulina Joãozinho da Costa Jarra Manneh; Rei José Pereira; Rui Inndi; Sainabou Laye Ndure; Simão Tchuda Bióté
EPI_ISL_2835169	Laboratório de Biotecnologia Aplicada (LBA) - Laboratório de Biologia Molecular - Hospital das Clínicas, Faculdade de Medicina de Botucatu, Departamento de Bioprocessos e Biotecnologia - Faculdade de Ciências Agronômicas, UNESP – Botucatu/SP	Laboratory of Respiratory Viruses and Measles, Oswaldo Cruz Institute, FIOCRUZ	Alice Sampaio Rocha; Ana Carolina Mendonca; Anna Carolina Paixao; Elisa Cavalcante Pereira; Felipe Allan da Silva Costa; Fernando Motta; Jayme Augusto de Souza Neto; Leonardo Nazario de Moraes; Luciana Appolinario; Marilda Siqueira on behalf of the Fiocruz COVID-19 Genomic Surveillance Network; Paola Resende; Patricia Akemi Assato; Rejane Maria Tommasini; Renata Serrano Lopes; Taina Venas
EPI_ISL_832012	Laboratório de Microbiologia Molecular - Universidade FEEVALE	Universidade Federal de Ciências da Saúde de Porto Alegre	Amanda de Menezes Mayer; Carla Andretta Moreira Neves; Claudia Elizabeth Thompson; Fernando Rosado Spilki; Gabriel Dickin Caldana; Gabriela Bettella Cybis; Lívia Kmetzsch; Patrícia Aline Gröhs Ferrareze; Ricardo Ariel Zimerman; Vinícius Bonetti Franceschi
EPI_ISL_1216912	Labormedizin Darmstadt	Robert Koch Institute	
EPI_ISL_1233630, EPI_ISL_1233663, EPI_ISL_1299921, EPI_ISL_1914638, EPI_ISL_1973556, EPI_ISL_2274472, EPI_ISL_2484522, EPI_ISL_2768045, EPI_ISL_3030090			
see above	Labormedizinisches Zentrum Dr Risch	Clinical Bacteriology	Adrian Egli; Alfredo Mari; Fanny Wegner; Hans Hirsch; Helena MB Seth-Smith; Julia Bielicki; Karoline Leuzinger; Lorenz Risch; Madlen Stange; Manuel Battegay; Martin Risch; Nadia Wohlwend; Tim Roloff
EPI_ISL_2677281, EPI_ISL_2677286	Labortorio Central de Saude Publica do Estado de Santa Catarina (LACEN/SC)	Laboratory of Respiratory Viruses and Measles, Oswaldo Cruz Institute, FIOCRUZ	Alice Sampaio Rocha; Ana Carolina Mendonca; Anna Carolina Paixao; Darcita Buerger Rovaris; Elisa Cavalcante Pereira; Fernando Motta; Luciana Appolinario; Marilda Siqueira on behalf of the Fiocruz COVID-19 Genomic Surveillance Network; Paola Resende; Renata Serrano Lopes; Sandra Bianchini Fernandes; Taina Venas
EPI_ISL_660133	Lancet	National Health Laboratory Service (NHLS), Tygerberg	Bronwyn Kleinhans; Davis M-A; Draper C; Eduan Wilkindon; Gert van Zyl; Houriiyah Tegally; Hsiao M; Kayla Delaney; Siegfried N; Susan Engelbrecht; Tulio de Oliveira; Williamson C; Wolfgang Preiser
EPI_ISL_2013052	Lancet Laboratories	National Institute for Communicable Diseases of the National Health Laboratory Service	Amoako DG; Bhiman JN; Glass A; Mahlangu B; Mohale T; Ntuli N; Scheepers C; Viana R
EPI_ISL_738336, EPI_ISL_812275, EPI_ISL_812281	Landstuhl Regional Medical Center	United States Air Force School of Aerospace Medicine	Amanda Javorina; Anthony Fries; Clarise Starr; Cole Anderson; Elizabeth Macias; Fritz Castillo; Jennifer Meyer; Sarah Purves; William Gruner
EPI_ISL_407079	Lapland Central Hospital	Department of Virology, University of Helsinki and Helsinki University Hospital, Helsinki, Finland	Hannimari Kallio-Kokko; Olli Vapalahti; Suvi Kuivanen; Teemu Smura
EPI_ISL_2811989	Laverty Pathology	NSW Health Pathology - Institute of Clinical Pathology and Medical Research; Westmead Hospital; University of Sydney	CIDM-PH et al.
EPI_ISL_2863684	Lboratorio Central de Saude Publica do Estado do Parana (LACEN/PR)	Laboratory of Respiratory Viruses and Measles, Oswaldo Cruz Institute, FIOCRUZ	Agatha Cristinne Prudencio; Alice Sampaio Rocha; Ana Carolina Mendonca; Anna Carolina Paixao; Elisa Cavalcante Pereira; Fernando Motta; Ighor Leonardo Arantes Gomes; Irina Riediger; Luciana Appolinario; Marilda Siqueira on behalf of the Fiocruz COVID-19 Genomic Surveillance Network; Paola Resende; Renata Serrano Lopes; Taina Venas
EPI_ISL_2779329	Life Care Hospital (Bungoma)	USAMRD-A, Basic Science Laboratory	Alan Lemtudo; Beth Mutai; Brian Andika; Carol Kifude; Clement Masakwe; Eric Muthanje; Esther Omuseni; Faith Sigei; Gathii Kimita; George Awinda; John Waitumbi; Josphat Nyataya; Rachel Githii; Rehema Liyai; Stephen Ochola
EPI_ISL_2136815	Life Sciences Center, Vilnius University	Institute of Biotechnology, Life Sciences Center, Vilnius University	Albertas Timinskas; Alma Gedvilaite; Danguole Ziogiene; Emilija Vasiliunaite; Milda Norkiene
EPI_ISL_1510207	Life Sciences Center, Vilnius University	Vilnius University Hospital Santaros Klinikos, Center of Laboratory Medicine	Daniel Naumovas; Dovile Ezerskyte; Gytis Dudas; Ingrida Olendraite; Laimonas Griskevicius; Ligita Raugaite; Mindaugas Stoskus; Monika Katenaite; Rimvydas Norvilas
EPI_ISL_701677, EPI_ISL_701777, EPI_ISL_703603, EPI_ISL_709416, EPI_ISL_720734, EPI_ISL_720848, EPI_ISL_720858, EPI_ISL_731623, EPI_ISL_800014, EPI_ISL_835513, EPI_ISL_1256078, EPI_ISL_1414488, EPI_ISL_1415461, EPI_ISL_1415755, EPI_ISL_1415960, EPI_ISL_1594716, EPI_ISL_1594784, EPI_ISL_1653511, EPI_ISL_1699106, EPI_ISL_2005299, EPI_ISL_2468924, EPI_ISL_2553223, EPI_ISL_2995319			
see above	Lighthouse Lab in Alderley Park	Wellcome Sanger Institute for the COVID-19 Genomics UK (COG-UK) Consortium	Cordelia Langford; David K. Jackson; Dominic Kwiatkowski; Ewan Harrison; Ian Johnston; Jacquelyn Wynn; Jeffrey Barrett; John Sillitoe on behalf of the Wellcome Sanger Institute COVID-19 Surveillance Team; Mairead Hyland; Roberto Amato; Sonia Goncalves; The Lighthouse Lab in Alderley Park and Alex Alderton
EPI_ISL_597618, EPI_ISL_597757, EPI_ISL_606128, EPI_ISL_611066, EPI_ISL_625220	Lighthouse Lab in Alderley Park	Wellcome Sanger Institute for the COVID-19 Genomics UK (COG-UK) consortium	Cordelia Langford; David K. Jackson; Dominic Kwiatkowski; Ewan Harrison; Ian Johnston; Jacquelyn Wynn; John Sillitoe on behalf of the Wellcome Sanger Institute COVID-19 Surveillance Team; John Sillitoe on behalf of the Wellcome Sanger Institute COVID-19 Surveillance Team (http://www.sanger.ac.uk/covid-team); Mairead Hyland; Roberto Amato; Sonia Goncalves; The Lighthouse Lab in Alderley Park and Alex Alderton
EPI_ISL_1263915, EPI_ISL_1275626, EPI_ISL_1275723, EPI_ISL_1275862, EPI_ISL_1276977, EPI_ISL_1316141, EPI_ISL_1316216, EPI_ISL_1316218, EPI_ISL_1327314, EPI_ISL_1327532, EPI_ISL_1327570, EPI_ISL_1329240, EPI_ISL_1332662, EPI_ISL_1332697, EPI_ISL_1409592, EPI_ISL_1409671, EPI_ISL_1467652, EPI_ISL_1473620, EPI_ISL_1538088, EPI_ISL_1584041, EPI_ISL_1652995, EPI_ISL_1719191, EPI_ISL_1831454			
see above	Lighthouse Lab in Cambridge	Wellcome Sanger Institute for the COVID-19 Genomics UK (COG-UK) Consortium	Cordelia Langford; David K. Jackson; Dominic Kwiatkowski; Ewan Harrison; Ian Johnston; Jeffrey Barrett; John Sillitoe on behalf of the Wellcome Sanger Institute COVID-19 Surveillance Team; Rob Howes; Roberto Amato; Sonia Goncalves; The Lighthouse Lab in Cambridge and Alex Alderton
EPI_ISL_608558	Lighthouse Lab in Cambridge	Wellcome Sanger Institute for the COVID-19 Genomics UK (COG-UK) consortium	Cordelia Langford; David K. Jackson; Dominic Kwiatkowski; Ewan Harrison; Ian Johnston; John Sillitoe on behalf of the Wellcome Sanger Institute COVID-19 Surveillance Team; Rob Howes; Roberto Amato; Sonia Goncalves; The Lighthouse Lab in Cambridge and Alex Alderton
EPI_ISL_720767, EPI_ISL_760686, EPI_ISL_760985, EPI_ISL_859475, EPI_ISL_989853, EPI_ISL_1084473, EPI_ISL_1333020, EPI_ISL_1333066, EPI_ISL_1333267, EPI_ISL_1333902, EPI_ISL_1413324, EPI_ISL_2960675			
see above	Lighthouse Lab in Glasgow	Wellcome Sanger Institute for the COVID-19 Genomics UK (COG-UK) Consortium	Anna Dominiczak and Alex Alderton; Carol Clugston; Cordelia Langford; David Gray; David K. Jackson; Dominic Kwiatkowski; Ewan Harrison; Harper VanSteenhouse; Ian Johnston; Jeffrey Barrett; John Sillitoe on behalf of the Wellcome Sanger Institute COVID-19 Surveillance Team; John Sillitoe on behalf of the Wellcome Sanger Institute COVID-19 Surveillance Team (http://www.sanger.ac.uk/covid-team); Roberto Amato; Sonia Goncalves; Yumi Kasai
EPI_ISL_530530, EPI_ISL_530548, EPI_ISL_532638, EPI_ISL_532883, EPI_ISL_533001, EPI_ISL_533038, EPI_ISL_533069			
see above	Lighthouse Lab in Glasgow	Wellcome Sanger Institute for the COVID-19 Genomics UK (COG-UK) consortium	Anna Dominiczak and Alex Alderton; Carol Clugston; Cordelia Langford; David Gray; David K. Jackson; Dominic Kwiatkowski; Ewan Harrison; Harper VanSteenhouse; Ian Johnston; John Sillitoe; Roberto Amato; Sonia Goncalves; Yumi Kasai
EPI_ISL_878751, EPI_ISL_1072347, EPI_ISL_1332568, EPI_ISL_1631640, EPI_ISL_1698027, EPI_ISL_1698076, EPI_ISL_2317665			
see above	Lighthouse Lab in Milton Keynes	Wellcome Sanger Institute for the COVID-19 Genomics UK (COG-UK) Consortium	Cordelia Langford; David K. Jackson; Dominic Kwiatkowski; Ewan Harrison; Ian Johnston; Jeffrey Barrett; John Sillitoe on behalf of the Wellcome Sanger Institute COVID-19 Surveillance Team; Roberto Amato; Sonia Goncalves; The Lighthouse Lab in Milton Keynes and Alex Alderton
EPI_ISL_552471, EPI_ISL_553021, EPI_ISL_553243, EPI_ISL_557188, EPI_ISL_606702, EPI_ISL_606838	Lighthouse Lab in Milton Keynes	Wellcome Sanger Institute for the COVID-19 Genomics UK (COG-UK) consortium	Cordelia Langford; David K. Jackson; Dominic Kwiatkowski; Ewan Harrison; Ian Johnston; John Sillitoe on behalf of the Wellcome Sanger Institute COVID-19 Surveillance Team; John Sillitoe on behalf of the Wellcome Sanger Institute COVID-19 Surveillance Team (http://www.sanger.ac.uk/covid-team); Roberto Amato; Sonia Goncalves; The Lighthouse Lab in Milton Keynes and Alex Alderton
EPI_ISL_2635692	Limbach - MVZ Humangenetik Ulm	Robert Koch Institute	
EPI_ISL_603088, EPI_ISL_603119, EPI_ISL_636840, EPI_ISL_636842, EPI_ISL_636870, EPI_ISL_636880, EPI_ISL_636881, EPI_ISL_636888			
see above	Lithuanian University of Health Sciences Hospital, Department of Laboratory Medicine	Lithuanian University of Health Sciences, Molecular cardiology lab.	Arnoldas Pautienius; Astra Vitkauskiene; Dovydas Gecys; Ingrida Olendraitė; Kamile Tamusauskaite; Laura Pareckaite; Lukas Zemaitis; Vaiva Lesauskaite
EPI_ISL_1502008	Lurie Children's Hospital of Chicago	Northwestern University - Ozer Lab	Egon A. Ozer; Judd F. Hultquist; Lacy M. Simons; Larry K. Kociolek; Michael G. Ison; Ramon Lorenzo-Redondo; Taylor J. Dean; William J. Muller; Xiaotian; Zheng

EPI_ISL_2827933, EPI_ISL_2827939, EPI_ISL_2984851, EPI_ISL_2984857, EPI_ISL_3024551, EPI_ISL_3024553, EPI_ISL_3024567, EPI_ISL_3024568			
see above	MANKWENG LABORATORY	National Institute for Communicable Diseases of the National Health Laboratory Service	Amoako DG; Bhiman JN; Everatt J; Ismail A; Mahlangu B; Mnguni A; Mohale T; Ntuli N; Scheepers C
EPI_ISL_1258302, EPI_ISL_1594077, EPI_ISL_2495486, EPI_ISL_2495574, EPI_ISL_2495647, EPI_ISL_2495669	MB-Cadham Provincial laboratory	National Microbiology Laboratory (NML)	Anna Majer; Anneliese Landgraff; CanCOGeN's metadata curation team; Darian Hole; David Alexander; Elsie Grudeski; Gary Van Domselaar; Grace Seo; Jared Bullard; Jennifer Tanner; Kerry Dust; Kirsten Biggar; Madison Chapel; Morag Graham; Natalie Knox; Nathalie Bastien; Paul Van Caeseele; Philip Mabon; Public Health Agency of Canada CanCOGeN team; Rhiannon Huzarewich; Russell Mandes; Shari Tyson; Timothy Booth; Yan Li
EPI_ISL_1152607, EPI_ISL_1435428	MDI Limbach Berlin GmbH	Robert Koch Institute	
EPI_ISL_2120501, EPI_ISL_2472756, EPI_ISL_2632870, EPI_ISL_2678707, EPI_ISL_2678719, EPI_ISL_2761877	MDI Limbach Berlin GmbH; MVZ Labor Berlin	Robert Koch Institute	
EPI_ISL_568978, EPI_ISL_569028, EPI_ISL_569395, EPI_ISL_569496, EPI_ISL_900072, EPI_ISL_900176, EPI_ISL_1020363, EPI_ISL_1694792			
see above	MEPHI, Aix Marseille University	MEPHI, Aix Marseille University	Anthony LEVASSEUR
EPI_ISL_2895751, EPI_ISL_2895753, EPI_ISL_2895755, EPI_ISL_2895756, EPI_ISL_2895757, EPI_ISL_2895759, EPI_ISL_2895760, EPI_ISL_2895763, EPI_ISL_2895764, EPI_ISL_2895765, EPI_ISL_2942282			
see above	MIDDELBURG LABORATORY	National Institute for Communicable Diseases of the National Health Laboratory Service	Amoako DG; Bhiman JN; Everatt J; Ismail A; Mahlangu B; Mnguni A; Mohale T; Ntuli N; Scheepers C
EPI_ISL_2566568	MKK Mühlenkreiskliniken, Johannes Wesling Klinikum Minden	Hannover Medical School, Institute of Virology	Lars Steinbrück
EPI_ISL_1226380	MO State Public Health Laboratory	Genomics and Discovery, Respiratory Viruses Branch, Division of Viral Diseases, Centers for Disease Control and Prevention	Anna Montmayeur; Anna Uehara; Ben L. Rambo-Martin; Clinton R. Paden; Dhwani Batra; Haibin Wang; Jasmine Padilla; Jing Zhang; Justin Lee; Katie Dillon; Krista Queen; Kristen Knipe; Kristine Lacek; Lori Rowe; Mark Burroughs; Matthew Schmerer; Mili Sheth; Peter W. Cook; Rachel Marine; Sam Shepard; Sarah Nobles; Shoshona Le; Suxiang Tong; Yan Li; Ying Tao
EPI_ISL_2556117, EPI_ISL_2557251	MOH - Jaber Al-Ahmad Hospital (Innovation Research Laboratory)	MOH - Jaber Al-Ahmad Hospital (Innovation Research Laboratory)	Mohammad Alghounaim; Salman Al-Sabah
EPI_ISL_2779435	MOH-K	USAMRD-A, Basic Science Laboratory	Alan Lemtudo; Beth Mutai; Brian Andika; Carol Kifude; Clement Masakwe; Eric Muthanje; Esther Omuseni; Faith Sigei; Gathii Kimita; George Awinda; John Waitumbi; Josphat Nyataya; Rachel Githii; Rehema Liyai; Stephen Ochola
EPI_ISL_2779526, EPI_ISL_2779528, EPI_ISL_2779534	MOH-Q	USAMRD-A, Basic Science Laboratory	Alan Lemtudo; Beth Mutai; Brian Andika; Carol Kifude; Clement Masakwe; Eric Muthanje; Esther Omuseni; Faith Sigei; Gathii Kimita; George Awinda; John Waitumbi; Josphat Nyataya; Rachel Githii; Rehema Liyai; Stephen Ochola
EPI_ISL_2346387, EPI_ISL_2346398, EPI_ISL_2346404, EPI_ISL_2346424, EPI_ISL_2346425, EPI_ISL_2346426, EPI_ISL_2346428, EPI_ISL_2346429, EPI_ISL_2346430, EPI_ISL_2346432, EPI_ISL_2346434, EPI_ISL_2346435			
see above	MRC/UVRI & LSHTM Uganda Research Unit, Central Public Health Laboratories	MRC/UVRI & LSHTM Uganda Research Unit, Central Public Health Laboratories	Dan Lule Bugembe; Isaac Sseeewanyana; Matthew Cotten; My V.T. Phan; Patrick Semanda; Pontiano Kaleebu; Susan Nabadda
EPI_ISL_1970565, EPI_ISL_1970566, EPI_ISL_1970567, EPI_ISL_1970568	MRC/UVRI & LSHTM Uganda Research Unit	MRC/UVRI & LSHTM Uganda Research Unit	Dan Lule Bugembe; Isaac Sseeewanyana; Matthew Cotten; My V.T. Phan; Patrick Semanda; Pontiano Kaleebu; Susan Nabadda
EPI_ISL_954236, EPI_ISL_954239, EPI_ISL_1469319, EPI_ISL_1469324, EPI_ISL_1469337, EPI_ISL_1469381, EPI_ISL_1469382, EPI_ISL_1469385			
see above	MRC/UVRI & LSHTM Uganda Research Unit	Where sequence data have been generated and submitted to GISAID	Dan Lule Bugembe; Isaac Sseeewanyana; Matthew Cotten; My V.T. Phan; Patrick Semanda; Pontiano Kaleebu; Susan Nabadda
EPI_ISL_2690446, EPI_ISL_2690447, EPI_ISL_2690448, EPI_ISL_2690449, EPI_ISL_2690450, EPI_ISL_2690451, EPI_ISL_2690452, EPI_ISL_2690456, EPI_ISL_2690457, EPI_ISL_2690458, EPI_ISL_2690459, EPI_ISL_2690460, EPI_ISL_2690461, EPI_ISL_2690462, EPI_ISL_2690463, EPI_ISL_2690485, EPI_ISL_2690496, EPI_ISL_2690500, EPI_ISL_2690513			
see above	MRC/UVRI & LSHTM Uganda Research Unit, Central Public Health Laboratories, Rakai Health Sciences Program	MRC/UVRI & LSHTM Uganda Research Unit, Central Public Health Laboratories	Dan Lule Bugembe; Hellen Nansumba; Isaac Sseeewanyana; Matthew Cotten; My V.T. Phan; Patrick Semanda; Pontiano Kaleebu; Susan Nabadda
EPI_ISL_2690464, EPI_ISL_2690465, EPI_ISL_2690466, EPI_ISL_2690467, EPI_ISL_2690468, EPI_ISL_2690469, EPI_ISL_2690470, EPI_ISL_2690471, EPI_ISL_2690472, EPI_ISL_2690473, EPI_ISL_2690474, EPI_ISL_2690475, EPI_ISL_2690476, EPI_ISL_2690477, EPI_ISL_2690478			
see above	MRC/UVRI & LSHTM Uganda Research Unit, Central Public Health Laboratories, Rakai Health Sciences Program	MRC/UVRI & LSHTM Uganda Research Unit, Rakai Health Sciences Program	Charles Ssuuna; Dan Lule Bugembe; Matthew Cotten; My V.T. Phan; Pontiano Kaleebu; Ronald Moses Galiwango; Steven J Reynolds
EPI_ISL_428857, EPI_ISL_471158, EPI_ISL_471160, EPI_ISL_471166, EPI_ISL_561027, EPI_ISL_561085, EPI_ISL_561218, EPI_ISL_1216072, EPI_ISL_1216077, EPI_ISL_1216118, EPI_ISL_1234526, EPI_ISL_1234531, EPI_ISL_1731549, EPI_ISL_1731557, EPI_ISL_2142749, EPI_ISL_2820697, EPI_ISL_2941560, EPI_ISL_2958626, EPI_ISL_2958629, EPI_ISL_2958631, EPI_ISL_2958632, EPI_ISL_2958636, EPI_ISL_2958637, EPI_ISL_2958638, EPI_ISL_2958639, EPI_ISL_2958644, EPI_ISL_2958645, EPI_ISL_2958646, EPI_ISL_2958647			
see above	MRCG at LSHTM Genomics lab	MRCG at LSHTM Genomics lab	Abdoulie Kanteh; Abdul Karim sesay; Bakary Sanyang; Dabiri Damilari; Damiri Damilari; Jarra Manneh; Mariama Kujabi; Sainabou laye Ndure; Sesay et al
EPI_ISL_903937	MS Public Health Laboratory	Genomics and Discovery, Respiratory Viruses Branch, Division of Viral Diseases, Centers for Disease Control and Prevention	Anna Montmayeur; Anna Uehara; Ben L. Rambo-Martin; Clinton R. Paden; Dhwani Batra; Haibin Wang; Jasmine Padilla; Jing Zhang; Justin Lee; Krista Queen; Lori Rowe; Mark Burroughs; Mili Sheth; Peter W. Cook; Rachel Marine; Sarah Nobles; Suxiang Tong; Yan Li; Ying Tao
EPI_ISL_2224388, EPI_ISL_2920904	MS Public Health Laboratory	University of Mississippi Medical Center, Molecular and Genomics Core Facility	Ashley C. Johnson; D. Ashley Robinson; Ithiel J. Frame; Krishna K. Ayyalasomayajula; Michael R. Garrett; Wenjie Wu
EPI_ISL_802148, EPI_ISL_1300812, EPI_ISL_1300813, EPI_ISL_1301126	MSHS Clinical Microbiology Laboratories	MSHS Pathogen Surveillance Program	Adolfo García-Sastre; Adriana van de Guchte; Ajay Obla; Alberto Paniz-Mondolfi; Ana S. Gonzalez-Reiche; Andrew Kasarskis; Angela Amoako; Ashley S. Salimbangon; Ashley Salimbangon; Betsaida Salom Melo; Bremy Alburquerque; Brianne Ciferri; Charles Gleason; Daniel Floda; Deena R. Altman; Denise Jurczyszak; Elena Hirsch; Emilia Mia Sordillo; Emily Ferreri; Gintaras Deikus; Giulio Kleiner; Gopi Patel; Hala Alshammary; Harm van Bakel; Irina Oussenko; Jayeeta Dutta; Juan Soto; Julia Matthews; Katherine Beach; Kathryn Twyman; Kayla Russo; Komal Srivastava; Levy Sominsky; Mahmoud Awawda; Marta Luksza; Matthew M. Hernandez; Melissa Gitman; Michael D. Nowak; Mitchell J. Sullivan; Nancy Francoeur; Rachel Chernet; Robert Sebra; Sarah Schaefer; Shelcie Fabre; Shwetha Hara Sridhar; Viviana Simon; Ying-Chih Wang; Zenab Khan
EPI_ISL_2690428	MSK Microbiology Lab	MSK Microbiology Lab	Esther Babady; Krupa Jani; Tracy McMillen
EPI_ISL_1722072, EPI_ISL_1722136	MVZ Labor Dr. Fenner und Kollegen (Standort Hamburg)	Robert Koch Institute	
EPI_ISL_2261223	MVZ Labor Dr. Quade & Kollegen GmbH	Robert Koch Institute	
EPI_ISL_2636786, EPI_ISL_3044511	MVZ Labor Krone GbR	Robert Koch Institute	
EPI_ISL_2314763	MVZ Medizinisches Labor Hannover GmbH	Robert Koch Institute	
EPI_ISL_1936102, EPI_ISL_1936103, EPI_ISL_1936104, EPI_ISL_1936105, EPI_ISL_1936106, EPI_ISL_1936107, EPI_ISL_1936108, EPI_ISL_1936109, EPI_ISL_1936110, EPI_ISL_1936111, EPI_ISL_1936112, EPI_ISL_1936113, EPI_ISL_1936114, EPI_ISL_1936115, EPI_ISL_1936116, EPI_ISL_1936117, EPI_ISL_1936118, EPI_ISL_1936119, EPI_ISL_1936120, EPI_ISL_1936121, EPI_ISL_1936122, EPI_ISL_1936123, EPI_ISL_1936124, EPI_ISL_1936125, EPI_ISL_1936126, EPI_ISL_1936127, EPI_ISL_1936128, EPI_ISL_1936129, EPI_ISL_1936130, EPI_ISL_1936131, EPI_ISL_1936132, EPI_ISL_1936133, EPI_ISL_1936134, EPI_ISL_1936135, EPI_ISL_1936136, EPI_ISL_1936137, EPI_ISL_1936138, EPI_ISL_1936139, EPI_ISL_1936140, EPI_ISL_1936141, EPI_ISL_1936142, EPI_ISL_1936143, EPI_ISL_1936144, EPI_ISL_1936145, EPI_ISL_1936194, EPI_ISL_1936195, EPI_ISL_1936196, EPI_ISL_1936197, EPI_ISL_1936198, EPI_ISL_1936199, EPI_ISL_1936200, EPI_ISL_1936201, EPI_ISL_1936202, EPI_ISL_1936203, EPI_ISL_1936204, EPI_ISL_1936205, EPI_ISL_1936206, EPI_ISL_1936207, EPI_ISL_1936208, EPI_ISL_1936209, EPI_ISL_1936210, EPI_ISL_1936211, EPI_ISL_1936212, EPI_ISL_1936213, EPI_ISL_1936214, EPI_ISL_1936215, EPI_ISL_1936216, EPI_ISL_1936217, EPI_ISL_1936218, EPI_ISL_1936219, EPI_ISL_1936220, EPI_ISL_1936221, EPI_ISL_1936222, EPI_ISL_1936223, EPI_ISL_1936224, EPI_ISL_1936225, EPI_ISL_1936226, EPI_ISL_1936227, EPI_ISL_1936228, EPI_ISL_1936229, EPI_ISL_1936230, EPI_ISL_1936231, EPI_ISL_1936232, EPI_ISL_1936233, EPI_ISL_1936234, EPI_ISL_1936235, EPI_ISL_1936236, EPI_ISL_1936237, EPI_ISL_1936240, EPI_ISL_1936241, EPI_ISL_1936242, EPI_ISL_1936243, EPI_ISL_1936244, EPI_ISL_1936245, EPI_ISL_1936246, EPI_ISL_1936247, EPI_ISL_1936248, EPI_ISL_1936249, EPI_ISL_1936250, EPI_ISL_1936251, EPI_ISL_1936252, EPI_ISL_1936253, EPI_ISL_1936254, EPI_ISL_1936255, EPI_ISL_1936256, EPI_ISL_1936257, EPI_ISL_1936258, EPI_ISL_1936259, EPI_ISL_1936260, EPI_ISL_1936261, EPI_ISL_1936262, EPI_ISL_1936263, EPI_ISL_1936264, EPI_ISL_1936265, EPI_ISL_1936266, EPI_ISL_1936267, EPI_ISL_1936268, EPI_ISL_1936269, EPI_ISL_1936270, EPI_ISL_1936271, EPI_ISL_1936272, EPI_ISL_1936273, EPI_ISL_1936274, EPI_ISL_1936275, EPI_ISL_1936276, EPI_ISL_1936277, EPI_ISL_1936278, EPI_ISL_1936279, EPI_ISL_1936280, EPI_ISL_1936281, EPI_ISL_1936282, EPI_ISL_1936283, EPI_ISL_1936284, EPI_ISL_1936285, EPI_ISL_1936286, EPI_ISL_1936287, EPI_ISL_1936288, EPI_ISL_1936289, EPI_ISL_1936290, EPI_ISL_1936291, EPI_ISL_1936292, EPI_ISL_1936293, EPI_ISL_1936294, EPI_ISL_1936296, EPI_ISL_1936297, EPI_ISL_1936298, EPI_ISL_1936299, EPI_ISL_1936300, EPI_ISL_1936301, EPI_ISL_1936302, EPI_ISL_1936303, EPI_ISL_1936304, EPI_ISL_1936305, EPI_ISL_1936306, EPI_ISL_1936307, EPI_ISL_1936308, EPI_ISL_1936309, EPI_ISL_1936310, EPI_ISL_1936311, EPI_ISL_1936312, EPI_ISL_1936313, EPI_ISL_1936314, EPI_ISL_1936315, EPI_ISL_1936316, EPI_ISL_1936317, EPI_ISL_1936318, EPI_ISL_1936319, EPI_ISL_1936320, EPI_ISL_1936321, EPI_ISL_1936322, EPI_ISL_1936323, EPI_ISL_1936324, EPI_ISL_1936325, EPI_ISL_1936326, EPI_ISL_1936327, EPI_ISL_1936328, EPI_ISL_1936329, EPI_ISL_1936330, EPI_ISL_1936331, EPI_ISL_1936332, EPI_ISL_1936333, EPI_ISL_1936334, EPI_ISL_1936335, EPI_ISL_1936336, EPI_ISL_1936337, EPI_ISL_1936338, EPI_ISL_1936339, EPI_ISL_1936340, EPI_ISL_1936341, EPI_ISL_1936342, EPI_ISL_1936343, EPI_ISL_1936344, EPI_ISL_1936345, EPI_ISL_1936346, EPI_ISL_1936347, EPI_ISL_1936348, EPI_ISL_1936349, EPI_ISL_1936350, EPI_ISL_1936351, EPI_ISL_1936352, EPI_ISL_1936353, EPI_ISL_1936354, EPI_ISL_1936355, EPI_ISL_1936356, EPI_ISL_1936357, EPI_ISL_1936358, EPI_ISL_1936359, EPI_ISL_1936360, EPI_ISL_1936361, EPI_ISL_1936362, EPI_ISL_1936363, EPI_ISL_1936364, EPI_ISL_1936365, EPI_ISL_1936366, EPI_ISL_1936367, EPI_ISL_1936368, EPI_ISL_1936369, EPI_ISL_1936370, EPI_ISL_1936371, EPI_ISL_1936372, EPI_ISL_1936373, EPI_ISL_1936374, EPI_ISL_1969078, EPI_ISL_1969079, EPI_ISL_1969080, EPI_ISL_1969081			
see above	Main Chemical Laboratories Egypt Army	Main Chemical Laboratories Egypt Army	Abdullah Salama; AbedElrahman Zekri; Ahmed Gad; Bassem Elharty; Laila Elsawy; Mervat Hassan; Mohamed Abdel-Monem; Mohamed El-Esawi; Mohamed Seadawy; Mohamed Shamel; Mostfa Elhoseiny; Mostfa Yakout; Ola Elroby; Sabah Ahmed; Sherine Helmy
EPI_ISL_2647352, EPI_ISL_2875445	Mako Medical	Centers for Disease Control and Prevention Division of Viral Diseases, Pathogen Discovery	Adrian Paskey; Benjamin Rambo-Martin; Christopher Gulvick; Clinton R. Paden; Dakota Howard; Darlene Wagner; Dhwani Batra; Duncan MacCannell; Jason Caravas; Kara Moser; Lauren Moon; Matthew Schmerer; Matthew Tugwell; Peter W. Cook; Scott Sammons; Shatavia Morrison; Yvette Unoarumhi
EPI_ISL_582124	Malaysia Genome Institute	Malaysia Genome Institute	Azrin Ahmad; Enizza Kasim; Irni Suhayu Sapian; Mohd Faizal Abu Bakar; Mohd Noor Mat Isa; Nor Azfa Johari; Nurhezreen Md Iqbal; Shahrul Hisham Zainal Ariffin; Shamsidar Sopie; Siti Noraini Othman; Yusuf Muhammad Noor
EPI_ISL_1827700	Manzana	National Institute for Communicable Diseases of the National Health Laboratory Service	Amoako DG; Bhiman JN; Ismail A; Mahlangu B; Maphalala GP; Mohale T; Ntuli N; Scheepers C
EPI_ISL_2373638	Mapmygenome	CSIR-Centre for Cellular and Molecular Biology - INSACOG	Amareshwar Vodapalli; Ara Sreenivas; B Himasri; Divya Tej Sowpati; Karthik Bharadwaj Tallapaka; Lamuk Zaveri; Onkar Kulkarni; Rakesh K Mishra; Sharath Chandra Thota; Shreekant Verma; Sofia Banu; Viswagithe S L
EPI_ISL_1910606, EPI_ISL_1910607, EPI_ISL_1910617, EPI_ISL_1913025	Mashrek Medical Diagnostic Center	Microbial Pathogenomics Lab - LAU	Georgi Merhi; Jad Koweyes; Sima Tokajian; Tamara Salloum
EPI_ISL_692915	Massachusetts State Public Health Laboratory	Massachusetts State Public Health Laboratory	Andrew Lang; Glen Gallagher; Sandra Smole; Timelia Fink

EPI_ISL_2547344	Matjieskloof Clinic	National Health Laboratory Service/University of Cape Town (NHLS/UCT)	Arash Iranzadeh; Bruna Galvao; Carolyn Williamson; Deelan Doolabh; Diana Hardie; Gert Marais; Innocent Mudau; Lynn Tyers; Marvin Hsiao; Stephen Korsman
EPI_ISL_1196004	Mbabane PHU	National Institute for Communicable Diseases of the National Health Laboratory Service	Amoako DG; Bhiman JN; Ismail A; Mahlangu B; Maphalala GP; Mohale T; Ntuli N; Scheepers C
EPI_ISL_1722079, EPI_ISL_2110393, EPI_ISL_2260594	Med. Labor Prof. Schenk Dr. Ansorge & Kollegen	Robert Koch Institute	
EPI_ISL_456378	MedLab Central Ltd	Institute of Environmental Science and Research (ESR)	Anja Werno; Antje van der Linden; Arlo Upton; Chris Mansell; David Hammer; Dragana Drinkovic; Erasmus Smit; Gary McAuliffe; Hana Sofia Andersson; James Ussher; Jill Sherwood; Joep de Ligt; Josh Freeman; Julia Howard; Juliet Elvy; Lauren Jelly; Mary DeAlmeida; Matt Blakiston; Matt Storey; Matthew Rogers; Max Bloomfield; Michael Addidle; Michelle Balm; Sally Roberts; Sarah Jefferies; Sharmini Muttaiyah; Susan Morpeth; Susan Taylor; Timothy Blackmore; Vani Sathyendran; Veronica Playle; Virginia Hope; Xiaoyun Ren
EPI_ISL_475745, EPI_ISL_475746, EPI_ISL_475747, EPI_ISL_475748, EPI_ISL_475749, EPI_ISL_475750, EPI_ISL_475751, EPI_ISL_475752, EPI_ISL_475753, EPI_ISL_482759, EPI_ISL_482760, EPI_ISL_482761, EPI_ISL_482762, EPI_ISL_482763, EPI_ISL_482764, EPI_ISL_482765, EPI_ISL_482766, EPI_ISL_482767, EPI_ISL_482768, EPI_ISL_482769, EPI_ISL_482770, EPI_ISL_482771, EPI_ISL_482772, EPI_ISL_482773, EPI_ISL_482774, EPI_ISL_482775, EPI_ISL_483035, EPI_ISL_483036, EPI_ISL_483037, EPI_ISL_483038, EPI_ISL_1098839, EPI_ISL_1109484, EPI_ISL_1109485, EPI_ISL_1109486, EPI_ISL_1109624, EPI_ISL_1109625, EPI_ISL_1109626, EPI_ISL_1109627, EPI_ISL_1109628, EPI_ISL_1109629, EPI_ISL_1109630, EPI_ISL_1141525, EPI_ISL_1165078, EPI_ISL_1165079, EPI_ISL_1165080, EPI_ISL_1165082, EPI_ISL_1165083, EPI_ISL_1165087, EPI_ISL_1167189, EPI_ISL_1167193, EPI_ISL_1586895			
see above	Medical Ain Shams Research Institute (MASRI), Ain Shams University	Medical Ain Shams Research Institute (MASRI), Ain Shams University	Ahmad Moustafa; Ashraf Omar; Aya Mohamed; Fatma Ebied; Ghada Ismael; Hagar Elshora; Hala Hafez; Hesham Elghazaly; Hoda Abd Elsatar; Hoda Ezz Elarab; Iman Foda; Mahmoud Elmeitini; Mahmoud Elmeteini; Manal Hamdy Elsaid; Mohamed Elhadidi; Osama Mansour; Osama Mansour.; Reham Kassab; Reham Mamdouh; Samia Abdo; Samia Abdou Girgis; Sara Elnakeep; Sara Hassan Agwa; Shaimaa Moustafa; Shimaa Moustafa
EPI_ISL_873164, EPI_ISL_2107508, EPI_ISL_2107509, EPI_ISL_2107510, EPI_ISL_2107511, EPI_ISL_2107512, EPI_ISL_2107524			
see above	Medical Laboratory Sciences, Arab American University	Medical Laboratory Sciences, Arab American University	Al-Jawabreh, A.; Al-Jawabreh, H.; Dumaidi, K.; Dumaidi, k.; Ereqat, S.; Nasereddin, A.
EPI_ISL_882646	Medical Research Center, Faculty of Medicine, Syarif Hidayatullah State Islamic University Jakarta	Medical Research Center, Faculty of Medicine, Syarif Hidayatullah State Islamic University Jakarta	Chris Adhiyanto; Endah Wulandari; Fika Ekayanti; Flori Ratna Sari; Hari Hendarto; Laifa Hendarmin; Zeti Harriyati
EPI_ISL_581457	Medizinische Klinik Innere Medizin I, Universitätsklinikum Tübingen	NGS Competence Center Tübingen, Institut für Medizinische Mikrobiologie und Hygiene, Universitätsklinikum Tübingen	Angel Angelov
EPI_ISL_1726791	Medizinisches Labor Wahl LÃ¼denscheid	Robert Koch Institute	
EPI_ISL_1722630	Medizinisches Zentrallabor Altenburg GmbH & Co.KG	Robert Koch Institute	
EPI_ISL_1158449	Michigan Department of Health and Human Services, Bureau of Laboratories	Michigan Department of Health and Human Services, Bureau of Laboratories	Blankenship HM; Riner D; Soehnlen MK
EPI_ISL_426477	Microbial Genomics Laboratory, Institut Pasteur Montevideo	Microbial Genomics Laboratory, Institut Pasteur Montevideo, Uruguay	Cecilia Salazar; Florencia Díaz-Viraqué; Gonzalo Moratorio; Gregorio Iraola; Marianoel Pereira; Pilar Moreno
EPI_ISL_480346	Microbial Genomics Laboratory, Institut Pasteur de Montevideo	Microbial Genomics Laboratory, Institut Pasteur de Montevideo	Cecilia Salazar; Gonzalo Moratorio; Gregorio Iraola; Ignacio Ferrés; Marianoel Pereira; Pilar Moreno
EPI_ISL_519427	Microbiological Diagnostic Unit - Public Health Laboratory (MDU-PHL)	MDU-PHL	Sait, M.; Schultz M.; Seemann T.; Sherry, N.
EPI_ISL_427149, EPI_ISL_430668	Microbiological Diagnostic Unit Public Health Laboratory	Microbiological Diagnostic Unit Public Health Laboratory	Sait, M.; Schultz M.; Seemann T.; Sherry, N.
EPI_ISL_513925	Microbiology & Bioinformatics and Biostatistics, Kohat University of Science and Technology (Pakistan) & Shanghai Jiao Tong University (China)	Microbiology & Bioinformatics and Biostatistics, Kohat University of Science and Technology (Pakistan) & Shanghai Jiao Tong University (China)	A.S.; Ali, S.; D.Q.; Khan; M.T.; Muhammad; N. and Wei; T.A.
EPI_ISL_2136858	Microbiology Department, Laboratori Clínic Metropolitana Nord. Hospital Universitari Germans Trias i Pujol	Can Ruti SARS-CoV-2 Sequencing Hub (HUGTiP/IrsiCaixa/IGTP)	Alba Sánchez; Anna Not; Antoni E Bordoy; Bonaventura Clotet; Cristina Casañ; Cristina Esteban; Francesc Catala-Moll; Gemma Clara; Ignacio Blanco; Marc Noguera-Julian; Maria Casadellà; Mariona Parera; Mercedes Guerrero; Montserrat Giménez; Pere-Joan Cardona; Pilar Armengol; Roger Paredes; Verónica Saludes; and Elisa Martró on behalf of the Can Ruti SARS-CoV-2 Sequencing Hub.
EPI_ISL_2931852	Microbiology Department. Complexo Hospitalario Universitario de Vigo	Microbiology Department. Complexo Hospitalario Universitario de Vigo	Alfaya N; Alonso I; Alvarez M; Cabrera JJ; Carballo R; Cores O; Cortizo S; Davina C; Martinez L; Mediero G; Perez S; Potel C; Regueiro B; Rey S; Vasallo FJ; del-Campo V
EPI_ISL_1303252	Microbiology Division, South Carolina Department of Health and Environmental Control	Microbiology Division, South Carolina Department of Health and Environmental Control	Flores, H.; Freeman, J.
EPI_ISL_2688489	Microbiology Laboratory, Attikon University Hospital, Athens	Central National Laboratory ,Public Health Organization	N.Siafakas; S.Pournaras et al
EPI_ISL_1416330	Microbiology and Virology Laboratory, "Policlinico Riuniti, Azienda Ospedaliero Universitaria, Foggia"	Microbiology and Virology Laboratory, "Policlinico Riuniti, Azienda Ospedaliero Universitaria, Foggia"	Annamaria D'Aprile; Daniela Pisanelli; Donatella Cedola; Fabio Arena; Loris Micelli; Maria Pia Patrizio; Maria Rosaria Lipsi; Maurizio Margaglione and Rosella De Nittis.; Settimia Altamura; Tiziana Rollo; Valeria Delli Carri
EPI_ISL_1064063	Microbiology and Virology Unit, Azienda Ospedale Padova, Padova, Italy	Department of Molecular Medicine, Computational Medicine Group, Univeresity of Padova, Padova, Italy	Andrea Crisanti; Andrea Spitaleri; Claudia Del Vecchio; Daniela Maria Cirillo; Dejan Lazarevic; Elisa Franchin; Enrico Lavezzo; Fabio Simeoni; Federico Bianca; Francesca Saluzzo; Francesco Onelia; Giovanni Lorenzin; Giovanni Tonon; Laura Manuto; Marco Grazioli; Stefano Toppo
EPI_ISL_3136161	Microbiology laboratory, National Center for Global Health and Medicine Hospital	Pathogen Genomics Center, National Institute of Infectious Diseases	Harutaka Katano; Kentaro Itokawa; Makoto Kuroda; Masanori Hashino; Norio Ohmagari; Rina Tanaka; Shinji Saito; Shun Iida; Tadaki Suzuki; Tsuyoshi Sekizuka; Yuichiro Hirata
EPI_ISL_2348487	Middle East Institute of Health University Hospital	Microbial Pathogenomics Lab - LAU	Edmond Abboud; Georgi Merhi; Jad Koweyes; Sima Tokajian
EPI_ISL_548032, EPI_ISL_548103, EPI_ISL_548145, EPI_ISL_579102, EPI_ISL_794624, EPI_ISL_1016845, EPI_ISL_1082258, EPI_ISL_1469089, EPI_ISL_1621293, EPI_ISL_1621326, EPI_ISL_1967893, EPI_ISL_1967898, EPI_ISL_1967900, EPI_ISL_2406492, EPI_ISL_2650019, EPI_ISL_2650024, EPI_ISL_2811945, EPI_ISL_2811946, EPI_ISL_2811954, EPI_ISL_2811956, EPI_ISL_2964937			
see above	Middlemore Hospital	Institute of Environmental Science and Research (ESR)	Anja Werno; Antje van der Linden; Arlo Upton; Chris Mansell; David Hammer; Dragana Drinkovic; Erasmus Smit; Gary McAuliffe; Hana Sofia Andersson; Hermes Perez; James Ussher; Jill Sherwood; Jing Wang; Joep de Ligt; Josh Freeman; Julia Howard; Juliet Elvy; Lauren Jelly; Mary DeAlmeida; Matt Blakiston; Matt Storey; Matthew Rogers; Max Bloomfield; Michael Addidle; Michelle Balm; Muhammad Faisal; Nikki Freed; Olin Silander; Olivia Stroeven; Paula scholes; Rachel Boyle; Sally Roberts; SallyAnn Harbison; Sarah Jefferies; Sharmini Muttaiyah; Susan Lin; Susan Morpeth; Susan Taylor; Timothy Blackmore; Vani Sathyendran; Veronica Playle; Virginia Hope; Xiaoyun Ren
EPI_ISL_718146, EPI_ISL_2367475, EPI_ISL_2535605, EPI_ISL_2535657, EPI_ISL_2535659	Ministry of Health Hospitals	Institute of Health and Community Medicine	Chan Chia Jui; Chua Hock Hin; David Perera; Ooi Mong How; Tonnii Sia Loong Loong; Wong Jyn Shan; Wong Kiing Aik
EPI_ISL_428713, EPI_ISL_814079, EPI_ISL_1020467, EPI_ISL_1117613, EPI_ISL_2158034, EPI_ISL_2158057, EPI_ISL_2158066, EPI_ISL_2158074, EPI_ISL_2232628, EPI_ISL_2232646, EPI_ISL_2232672, EPI_ISL_2232673, EPI_ISL_2232674, EPI_ISL_2232675, EPI_ISL_2232676, EPI_ISL_2232695, EPI_ISL_2363590, EPI_ISL_2363596, EPI_ISL_2363632, EPI_ISL_2363736, EPI_ISL_2403280, EPI_ISL_2958500, EPI_ISL_2984650, EPI_ISL_2984684, EPI_ISL_2984763, EPI_ISL_2987762, EPI_ISL_2987829, EPI_ISL_2987841, EPI_ISL_2987847, EPI_ISL_2987930, EPI_ISL_2987933, EPI_ISL_2988072, EPI_ISL_3032772, EPI_ISL_3032778, EPI_ISL_3063381, EPI_ISL_3063382, EPI_ISL_3066742, EPI_ISL_3066746			
see above	Ministry of Health Turkey	Ministry of Health Turkey	Ayşe Başak Altaş; Fatma Bayrakdar; Gulay Korukluoglu; Gülay Korukluoğlu; Selçuk Kılıç; Suleyman Yalcin; Süleyman Yalcin; Yasemin Cosgun; Yasemin Coşgun
EPI_ISL_2846023	Ministry of Health, Jaber Al-Ahmad Hospital	Virology Unit, Department of Microbiology, Faculty of Medicine, Kuwait University	Anfal Al-Adwani; Ebaa Al-Awadhi; Hussain Safar; Nada Madi
EPI_ISL_1712690, EPI_ISL_1712705, EPI_ISL_1713194, EPI_ISL_1713231, EPI_ISL_1713276, EPI_ISL_1713546, EPI_ISL_1713637, EPI_ISL_2274279, EPI_ISL_2274319, EPI_ISL_2274351, EPI_ISL_2274354, EPI_ISL_2274358, EPI_ISL_2274390, EPI_ISL_2408295, EPI_ISL_2408310, EPI_ISL_2408353, EPI_ISL_2408365, EPI_ISL_2842881, EPI_ISL_2842914, EPI_ISL_2843042, EPI_ISL_2843050, EPI_ISL_2843081, EPI_ISL_2843089, EPI_ISL_2843116, EPI_ISL_2843135, EPI_ISL_2843139, EPI_ISL_2843146, EPI_ISL_2843149, EPI_ISL_2843237, EPI_ISL_2843241, EPI_ISL_2843246, EPI_ISL_2843259, EPI_ISL_2843273, EPI_ISL_2843280, EPI_ISL_2843284, EPI_ISL_2843285			
see above	Ministry of Public Health / Hamad Medical Corporation	Biomedical Research Center (BRC), Qatar University / Qatar Genome Project (QGP)	Asmaa A. Al-Thani. MOPH and HMC: Abdullatif Al-Khal; Asmaa A. Al-Thani. MOPH and HMC: Abdullatif Al-Khal; BRC: Fatiha M. Benslimane; Chadi Saad; Dana Al-Batesh; Dina Elgakhlab QGP: Fatima H. Al-Kuwari; Dina Elgakhlab QGP: Fatima H. Al-Kuwari; Einas A. E. Al-Kuwari; Hadi M. Yassine; Hamad E. Al-Romaihi; Hamda Alromaihi; Heba A. Al-Khatib; Mashael A. Al-Bader; Mohammed Al-Thani; Muna A. S. Al-Maslamani; Oal Al-Jamal; Peter V. Coyle; Reham A. El-Kahlout. QBB: Tasneem Al-Hamad; Reham A. El-Kahlout. QBB: Tasneem Al-Hamad; Roberto Bertollini; Salih Al-Marri
EPI_ISL_1714165, EPI_ISL_1714185, EPI_ISL_1714328, EPI_ISL_1714331, EPI_ISL_1714468, EPI_ISL_1714531, EPI_ISL_1714557			
see above	Ministry of Public Health / Hamad Medical Corporation	Weill Cornell Medical College - Qatar (WCM-Q), Genomics Core Laboratory / Qatar Genome Project (QGP)	Chadi Saad MOPH and HMC: Abdullatif Al-Khal; Dina Elgakhlab; Einas A. E. Al-Kuwari; Hamad E. Al-Romaihi; Hamda Alromaihi; Joel A Malek. QGP: Fatima H. Al-Kuwari; Laith Abu-Raddad; Mashael A. Al-Bader; Meryem Bensaad; Mohammed Al-Thani; Muna A. S. Al-Maslamani; Peter V. Coyle; Reham A. El-Kahlout. QBB: Tasneem Al-Hamad; Roberto Bertollini; Salih Al-Marri; Shameem Younuskunju; WCMQ: Ayeda A. Ahmed; Yasmin Mohamoud
EPI_ISL_708485, EPI_ISL_1760680, EPI_ISL_2015217, EPI_ISL_2443802, EPI_ISL_2536893	Minnesota Department of Health, Public Health Laboratory	Minnesota Department of Health, Public Health Laboratory	Alexandra Lorentz; Jacob Garfin; Matt Plumb; and Xiong Wang
EPI_ISL_1362768, EPI_ISL_1392816, EPI_ISL_2035597	Missouri State Public Health Laboratory	Missouri State Public Health Laboratory	Ashley New; Joshua Barry; Matthew Sinn
EPI_ISL_2140698	Mitchells Plain Hospital wc MPH	NHLS/UCT	Arash Iranzadeh; Bruna Galvao; Carolyn Williamson; Deelan Doolabh; Diana Hardie; Innocent Mudau; Kruger Marais; Lynn Tyers; Marvin Hsiao; Stephen Korsman
EPI_ISL_2861095, EPI_ISL_2928345	Mobile County Health Department	Synergy Laboratories	Megan Cornwell
EPI_ISL_435127, EPI_ISL_463740, EPI_ISL_520723	Mohammed Bin Rashid University of Medicine and Health Sciences	Al Jalila Genomics Center	Abdulmajeed Alkhaja; Abiola Catherine Senok; Ahmad Abou Tayoun; Alawi Alsheikh-Ali; Divinlal Harilal; Hamda Khansaheb; Hanan Al Suwaidi; Mohammed Uddin; Norbert Nowotny; Qutayba Hamid; Rabih Halwani; Rifat Hamoudi; Rupa Murthy Varghese; Sathishkumar Ramaswamy; Tom Loney; Zulfa Omar Deesi
EPI_ISL_2101058, EPI_ISL_2101204	Molecular Biology Laboratory, Faculty Medicine and Health Sciences, Warmadewa University	Eijkman Institute for Molecular Biology, National Agency for Research and Innovation; Molecular Biology Laboratory, Faculty Medicine and Health Sciences, Warmadewa University	Amin Soebandrio; Edison Johar; Erly Sintya; Frilasita A Yudhaputri; Hidayat Trimarsanto; Iskandar Adnan; Khin Saw Myint; Lidwina Priliani; Lydia V. Panggalo; Muhammad Rezki Rasyak; Safarina G Malik; Sri Masyeni; Sukma Oktavianthi; Willy Agustine
EPI_ISL_2095819	Molecular Diagnostics Mater Dei Hospital Pathology Department Malta	Molecular Diagnostics Mater Dei Hospital Pathology Department Malta	C Cilia; G Zahra; L Grech; R Borg

EPI_ISL_2006673, EPI_ISL_2101345, EPI_ISL_2101350, EPI_ISL_2101358, EPI_ISL_2102068, EPI_ISL_2102070, EPI_ISL_2105875, EPI_ISL_2105881, EPI_ISL_2105939, EPI_ISL_2113984, EPI_ISL_2114905, EPI_ISL_2399438, EPI_ISL_2399457, EPI_ISL_2399611, EPI_ISL_2716425, EPI_ISL_2716474, EPI_ISL_2718538, EPI_ISL_2854661, EPI_ISL_2970370, EPI_ISL_2970563, EPI_ISL_2970965, EPI_ISL_2973300, EPI_ISL_2988654, EPI_ISL_2988658			
see above	Molecular Diagnostics Pathology Department Mater Dei Hospital Malta	Molecular Diagnostics Pathology Department Mater Dei Hospital Malta	C Cilia; G Zahra; L Grech; M Briffa; R Borg
EPI_ISL_681690, EPI_ISL_681700	Molecular Medicine Laboratory, University of Magallanes	Centro Asistencial Docente y de Investigacion, Universidad de Magallanes	Diego Alvarez; Hermy Alvarez; Jacqueline Aldridge; Jorge González; Marcelo Navarrete; Marco Montes de Oca; Roberto Uribe-Paredes
EPI_ISL_895793, EPI_ISL_895834	Molecular biology division, Institute of Clinical Biochemistry and Diagnostics, Charles University, Faculty of Medicine in Hradec Králové and University Hospital Hradec Králové	Molecular biology division, Institute of Clinical Biochemistry and Diagnostics, Charles University, Faculty of Medicine in Hradec Králové and University Hospital Hradec Králové	Helena Kovaříková; Ivana Baranová; Jitka Novotná; Kateřina Hrochová; Kateřina Pehlíková; Petr Brož; Tereza Baťková; Vladimír Palička. Cooperation project with BioVendor-R&D and bioinformatics company BIOXSYS s r.o.
EPI_ISL_2031957	Molecular diagnostic laboratory of Federal Budget Institution of Science "Central Research Institute of Epidemiology" of The Federal Service on Customers' Rights Protection and Human Well-being Surveillance	Group of Genomics and Postgenomic Technologies of Central Research Institute of Epidemilology	Akimkin V.G.; Kaptelova V.V.; Kondrasheva L.Y.; Korneenko E.V.; Nadtoka M.I.; Saenko S.S.; Samojlov A.E.; Shipulina O.Y.; Sinicyn S.O.; Smirnova Y.S.; Speranskaya A.S.; Tivanova E.V.
EPI_ISL_2626244	Molecular diagnostic laboratory of Federal Budget Institution of Science "Central Research Institute of Epidemiology" of The Federal Service on Customers' Rights Protection and Human Well-being Surveillance	Group of Genomics and Postgenomic Technologies of Central Research Institute of Epidemilology	Akimkin V.G.; Cherkashina A.S.; Kaptelova V.V.; Kondrasheva L.Y.; Korneenko E.V.; Nadtoka M.I.; Saenko S.S.; Samoilov A.E.; Sinitsyn S.O.; Smirnova Y.S.; Speranskaya A.S.; Tivanova E.V.
EPI_ISL_1365029, EPI_ISL_1365031, EPI_ISL_1367691, EPI_ISL_1663669, EPI_ISL_1663671	Molecular diagnostic unit for viral haemorrhagic fevers and emerging viruses, Bouaké CHU Laboratory	Molecular diagnostic unit for viral haemorrhagic fevers and emerging viruses, Bouaké CHU Laboratory	Adjaratou Traoré; Bamba Fatoumata Touré; Chantal Akoua-Koffi; Coulibaly Mbegnan; Diané Bamourou; Essia Belarbi; Etilé Anoh; Fabian Leendertz; Grit Schubert; Kra Ouffoué; Monemo Pacome; Oby Wayoro; Safiatou Karidioula; Soundélé Maïté
EPI_ISL_614348, EPI_ISL_614364, EPI_ISL_614382, EPI_ISL_614391, EPI_ISL_681834, EPI_ISL_682058, EPI_ISL_1662579, EPI_ISL_1662582, EPI_ISL_1662584			
see above	Molecular diagnostic unit for viral haemorrhagic fevers and emerging viruses, Bouaké CHU Laboratory	Project group Epidemology of Highly Pathogenic Microorganisms, Robert Koch-Institute	Adjaratou Traoré; Bamba Fatoumata Touré; Chantal Akoua-Koffi; Coulibaly Mbegnan; Diané Bamourou; Essia Belarbi; Etilé Anoh; Fabian Leendertz; Grit Schubert; Kra Ouffoué; Monemo Pacome; Safiatou Karidioula; Soundélé Maïté
EPI_ISL_1881945	Molekylær Medicinsk Afdeling, Aarhus University Hospital, Aarhus, Denmark	Aalborg University	Danish Covid-19 Genome Consortium
EPI_ISL_2350967, EPI_ISL_2351003	Mongkutwattana General Hospital	Division of Genomic Medicine and Innovation support,Department of Medical Sciences, Ministry of Public Health, Thailand	Archawin Rojanawiwat; Jirapha Pakdee; Natthakul Bunneang; Nuanjun Wichukchinda; Penpitcha Thawong; Pilailuk Akkapaiboon Okada; Pundharika Piboonsiri; Surakameth Mahasirimongkol; Waritta Sawaengdee
EPI_ISL_889366	Motol University Hospital	Institute of Applied Biotechnologies a.s.	Kateřina Kvapilová; Martin Kašný; Ondřej Brzoň; Pavel Dřevínek; Petr Klempt; Petr Kvapil
EPI_ISL_3031397	Mount Kenya Hospital	USAMRD-A, Basic Science Laboratory	Alan Lemtudo; Beth Mutai; Brian Andika; Carol Kifude; Clement Masakwe; Eric Muthanje; Esther Omuseni; Faith Sigei; Gathii Kimita; George Awinda; John Waitumbi; Josphat Nyataya; Rachel Githii; Rehema Liyai; Stephen Ochola
EPI_ISL_1534267	Mowbray Maternity Hospital wc MMH	NHLS/UCT	Arash Iranzadeh; Bruna Galvao; Carolyn Williamson; Deelan Doolabh; Diana Hardie; Innocent Mudau; Kruger Marais; Lynn Tyers; Marvin Hsiao; Stephen Korsman
EPI_ISL_2429125, EPI_ISL_2429128	Mubarak Al-Kabeer Hospital	Virology Unit, Department of Microbiology, Faculty of Medicine, Kuwait University	Anfal Al-Adwani; Hussain Safar; Nada Madi
EPI_ISL_2899875, EPI_ISL_2964432, EPI_ISL_2964451	N.F. Gamaleya Research Center for Epidemiology and Microbiology	WHO National Influenza Centre Russian Federation	Alexander Gintsburg; Alexey Shchetinin; Alina Odintsova; Andrei Botikov; Andrei Pochtovyi; Andrei Siniavin; Andrey Komissarov; Anna Kovyrshina; Artem Fadeev; Artem Tkachuk; Daria Danilenko; Denis Kleymenov; Denis Logunov; Dmitry Lioznov; Dmitry Shcheblyakov; Elena Mazunina; Elena Nabieva; Elena Shidlovskaya; Elizaveta Divisenko; Evgeniia Bykonia; Georgii Bazykin; Inna Dolzhikova; Kirill Varchenko; Ksenia Safina; Kseniya Komissarova; Liubov Popova; Ludmila Vasilchenko; Maria Nikiforova; Maria Pisareva; Mikhail Bakaev; Nadezhda Kuznetsova; Nikita Yolshin; Oula Mansour; Tamila Musaeva; Veronika Eder; Vladimir Gushchin
EPI_ISL_2382733	NAMRU-6	Genomics and Discovery, Respiratory Viruses Branch, Division of Viral Diseases, Centers for Disease Control and Prevention	Adam Retchless; Anna Kelleher; Anna Uehara; Brian Lynch; Clinton R. Paden; Dhwani Batra; Haibin Wang; Han Jia Justin Ng; Jasmine Padilla; Jing Zhang; Justin Lee; Krista Queen; Mark Burroughs; Mili Sheth; Morgan Davis; Peter Cook; Rachel Marine; Sarah Nobles; Suxiang Tong; Tara Coalter; Yan Li; Ying Tao
EPI_ISL_2550520	NB-Hôpital Georges L. Dumont	National Microbiology Laboratory (NML)	Anna Majer; Anneliese Landgraff; CanCOGeN's metadata curation team; Darian Hole; Elsie Grudeski; Gary Van Domselaar; Grace Seo; Guillaume Desnoyers; Jennifer Tanner; Kirsten Biggar; Madison Chapel; Morag Graham; Natalie Knox; Nathalie Bastien; Philip Mabon; Public Health Agency of Canada CanCOGeN team; Rhiannon Huzarewich; Richard Garceau; Russell Mandes; Shari Tyson; Timothy Booth; Yan Li
EPI_ISL_2710271, EPI_ISL_2726856	NHLS Charlotte Maxeke Johannesburg Academic Hospital and the University of the Witwatersrand	KRISP, KZN Research Innovation and Sequencing Platform	Bulelani Manene; Florette Treurnicht; Giandhari Jennifer; Kathleen Subramoney; Naidoo Yeshnee; Pillay Sureshnee; San James; Tegally Houriiyah; Tshabuila Derek; Wilkinson Eduan; Yajna Ramphal; de Oliveira Tulio
EPI_ISL_912481, EPI_ISL_1818856, EPI_ISL_1818891, EPI_ISL_2162307, EPI_ISL_2162308, EPI_ISL_2162309, EPI_ISL_2162311, EPI_ISL_2162320, EPI_ISL_2162335, EPI_ISL_2162339, EPI_ISL_2162340, EPI_ISL_2162341, EPI_ISL_2162346, EPI_ISL_2162347, EPI_ISL_2162351, EPI_ISL_2162354, EPI_ISL_2162357, EPI_ISL_2162358, EPI_ISL_2162359, EPI_ISL_2162360, EPI_ISL_2162361, EPI_ISL_2162362, EPI_ISL_2162365, EPI_ISL_2162366, EPI_ISL_2162371, EPI_ISL_2162378			
see above	NHLS Universitas Academic	UFS Virology	D Goedhals; Emmanuel Ogunbayo; MM Nyaga; MT Mogotsi; P Nthiga; PA Bester; T de Oliveira
EPI_ISL_467513, EPI_ISL_518043, EPI_ISL_529765, EPI_ISL_660605, EPI_ISL_660660, EPI_ISL_736971	NHLS-IALCH	KRISP, KZN Research Innovation and Sequencing Platform	Chimukangara B; ChimukangaraB; Giandhari J; Khan S; Lessells R; Mdlalose K; Pillay S; Tegally H; Wilkinson E; York D; de Oliveira T
EPI_ISL_860607	NHLS-IALCH	KRISP, KZn Research Innovation and Sequencing Platform	Giandhari J; Khan S; Lessells R; Mdlalose K; Pillay S; Tegally H; Wilkinson E; York D; de Oliveira T
EPI_ISL_2693039, EPI_ISL_2693050, EPI_ISL_2693075, EPI_ISL_2693084, EPI_ISL_2693093	NHLS_VIRO	KRISP, KZN Research Innovation and Sequencing Platform	Giandhari Jennifer; Naidoo Yeshnee; Pillay Sureshnee; San James; Tegally Houriiyah; Tshabuila Derek; Wilkinson Eduan; Yajna Ramphal; de Oliveira Tulio
EPI_ISL_2494387, EPI_ISL_2494397, EPI_ISL_2494427, EPI_ISL_2494465, EPI_ISL_2494505, EPI_ISL_2494506, EPI_ISL_2494511, EPI_ISL_2494541, EPI_ISL_2494569, EPI_ISL_2494618, EPI_ISL_2494619, EPI_ISL_2494625, EPI_ISL_2727380, EPI_ISL_2727391, EPI_ISL_2727393, EPI_ISL_2727394			
see above	NHLS_VIRO	KRISP, KZn Research Innovation and Sequencing Platform	Baillie Vicky; Giandhari Jennifer; Madhi Shabir; Naidoo Yeshnee; Pillay Sureshnee; San James; Tegally Houriiyah; Tshabuila Derek; Wilkinson Eduan; Yajna Ramphal; de Oliveira Tulio; du Plessis Jeanine
EPI_ISL_2693824, EPI_ISL_2693825, EPI_ISL_2693828, EPI_ISL_2693832, EPI_ISL_2693835, EPI_ISL_2693860, EPI_ISL_2693863, EPI_ISL_2693864, EPI_ISL_2693865, EPI_ISL_2693899, EPI_ISL_2893253, EPI_ISL_2893257, EPI_ISL_2893259, EPI_ISL_2893260, EPI_ISL_2893268, EPI_ISL_2893284, EPI_ISL_2893296			
see above	NHLS_VIRO Eastern Cape	KRISP, KZN Research Innovation and Sequencing Platform	Emmanuel SJ; Giandhari J; Giandhari Jennifer; Khan S; Laguda-Akingba O; Lessells R; Mdlalose K; Naidoo Yeshnee; Pillay S; Pillay Sureshnee; San James; Tegally H; Tegally Houriiyah; Tshabuila Derek; Wilkinson E; Wilkinson Eduan; Yajna Ramphal; York D; de Oliveira T; de Oliveira Tulio
EPI_ISL_2727175, EPI_ISL_2727179, EPI_ISL_2727180, EPI_ISL_2727185, EPI_ISL_2727186, EPI_ISL_2727189, EPI_ISL_2727193, EPI_ISL_2727194, EPI_ISL_2727195, EPI_ISL_2727196, EPI_ISL_2727197, EPI_ISL_2727198, EPI_ISL_2727216, EPI_ISL_2727217, EPI_ISL_2955454, EPI_ISL_2955464, EPI_ISL_2955466, EPI_ISL_2955472, EPI_ISL_2955474, EPI_ISL_2955475, EPI_ISL_2955479, EPI_ISL_2955484, EPI_ISL_2955487, EPI_ISL_2955488, EPI_ISL_2955490, EPI_ISL_2955497, EPI_ISL_2955499			
see above	NHLS_VIRO Eastern Cape	KRISP, KZn Research Innovation and Sequencing Platform	Emmanuel SJ; Giandhari J; Khan S; Laguda-Akingba O; Lessells R; Mdlalose K; Pillay S; Tegally H; Wilkinson E; York D; de Oliveira T
EPI_ISL_766863, EPI_ISL_766864, EPI_ISL_766865, EPI_ISL_766869, EPI_ISL_766873, EPI_ISL_766874, EPI_ISL_766875			
see above	NIC Viral Respiratory Unit - Institut Pasteur of Algeria	National Reference Center for Viruses of Respiratory Infections, Institut Pasteur, Paris	Angela Brisebarre; Etienne Simon-Lorière; Fawzi Derrar; Flora Donati; Marion Barbet; Maud Vanpeene; Mélanie Albert; Méline Bizard; Sylvie Behillil; Sylvie van der Werf; Vincent Enouf
EPI_ISL_577717	NIV Influenza	NIV Influenza	NIV; Potdar; Pune; Varsha on behalf of National Influenza Centre
EPI_ISL_2161796, EPI_ISL_2484617	NL-Dr. Leonard A. Miller Centre for Health Services	National Microbiology Laboratory (NML)	Adel Malek; Anna Majer; Anneliese Landgraff; CanCOGeN's metadata curation team; Darian Hole; Elsie Grudeski; Gary Van Domselaar; George Zahariadis; Grace Seo; Jennifer Tanner; Kerri Smith; Kirsten Biggar; Laura Gilbert; Madison Chapel; Morag Graham; Natalie Knox; Nathalie Bastien; Philip Mabon; Public Health Agency of Canada CanCOGeN team; Rhiannon Huzarewich; Robert Needle; Russell Mandes; Shari Tyson; Timothy Booth; Yan Li; Yang Yu
EPI_ISL_960304	NLZOH, Laboratory for Virology	NLZOH, Laboratory for Virology	Cesare Camma (IZSAM); Erik Alm (ECDC); Katarina Prosenc (Laboratory for Virology)
EPI_ISL_1579896, EPI_ISL_1628463, EPI_ISL_1654970	NMVRVI	National Public Health Surveillance Laboratory	Ana Steponkiene; Arnoldas Pautienius; Astra Vitkauskiene; Danas Baksa; Dovydas Gecys; Ingrida Olendraite; Jelena Razmuk; Kamile Tamusauskaite; Laura Pareckaite; Lukas Vasionis; Lukas Zemaitis; Migle Gabrielaite; Svajune Muralyte; Vaiva Lesauskaite
EPI_ISL_1016162	NORTHWELL HEALTH LABORATORIES	Wadsworth Center, New York State Department of Health	Alexis Russel; Daryl M. Lamson; Erasmus Schneider; Erica Lasek-Nesselquist; John Kelly; Jonathan Plitnick; Kirsten St. George; Matthew Shudt; Melissa A Leisner; Navjot Singh
EPI_ISL_416742	NRL for Influenza, Centrum Epidemiology and Microbiology of National Institute of Public Health, Czech Republic	Charite Universitaetsmedizin Berlin, Institute of Virology	Akexander Nagy; Barbara Muehlemann; Christian Drosten; Dusan Trnka; Helena Jirincova; Jaromira Vecerova; Jörn Beheim-Schwarzbach; Julia Schneider; Ludmila Novakova; Talitha Veith; Terry Jones; Victor M Corman
EPI_ISL_1272388, EPI_ISL_1272389, EPI_ISL_1272390, EPI_ISL_1272391, EPI_ISL_2162047, EPI_ISL_2162051	NS-QEII Health Sciences Centre	National Microbiology Laboratory (NML)	Anna Majer; Anneliese Landgraff; CanCOGeN's metadata curation team; Dan Gaston; Darian Hole; Elsie Grudeski; Gary Van Domselaar; Grace Seo; Janice Pettipas; Jason LeBlanc; Jennifer Tanner; Kirsten Biggar; Madison Chapel; Morag Graham; Natalie Knox; Nathalie Bastien; Philip Mabon; Public Health Agency of Canada CanCOGeN team; Rhiannon Huzarewich; Russell Mandes; Shari Tyson; Timothy Booth; Todd Hatchette; Yan Li
EPI_ISL_3046227	NUPIT/UFPE	WallauLab on behalf of Fiocruz COVID-19 Genomic Surveillance Network	Alexandre Freitas da Silva; Cassia Docena; Constância Flávia Junqueira Ayres; Filipe Zimmer Dezordi; Gabriel Luz Wallau; Gustavo Barbosa de Lima; Lais Ceschini Machado; Lilian Caroliny Amorim Silva; Maira Galdino da Rocha Pitta; Marcelo Henrique dos Santos Paiva; Matheus Filgueira Bezerra; Michelly Cristiny Pereira; Rômulo Pessoa e Silva; Sinval Pinto Brandão Filho
EPI_ISL_903727	NV State Public Health Laboratory	Genomics and Discovery, Respiratory Viruses Branch, Division of Viral Diseases, Centers for Disease Control and Prevention	Anna Montmayeur; Anna Uehara; Ben L. Rambo-Martin; Clinton R. Paden; Dhwani Batra; Haibin Wang; Jasmine Padilla; Jing Zhang; Justin Lee; Krista Queen; Lori Rowe; Mark Burroughs; Mili Sheth; Peter W. Cook; Rachel Marine; Sarah Nobles; Suxiang Tong; Yan Li; Ying Tao

EPI_ISL_1226660, EPI_ISL_1226661	NYC Pandemic Response Lab	Wadsworth Center, New York State Department of Health	Alexis Russel; Daryl M. Lamson; Erasmus Schneider; Erica Lasek-Nesselquist; John Kelly; Jonathan Plitnick; Kirsten St. George; Matthew Shudt; Melissa A Leisner; Navjot Singh
EPI_ISL_1922797	NYU Langone Health	Departments of Pathology and Medicine, New York University School of Medicine	Adriana Heguy; Christian Marier; Dacia Dimartino; Emily Guzman; Gael Westby; Guiqing Wang; Paolo Cotzia; Paul Zappile; Peter Meyn; Sitharam Ramaswami; Yutong Zhang
EPI_ISL_1960297, EPI_ISL_2694492	Nacionalinis maisto ir veterinarijos rizikos vertinimo institutas	National Public Health Surveillance Laboratory	Ana Steponkiene; Danas Baksa; Jelena Razmuk; Lukas Vasionis; Lukas Zemaitis; Migle Gabrielaite; Svajune Muralyte
EPI_ISL_2798341, EPI_ISL_2800233, EPI_ISL_2800265, EPI_ISL_2800271	Nacionālais medicīnas serviss - laboratorija, SIA	Riga East University Hospital, National Microbiology Reference Laboratory; Eurofins Genomics Europe Sequencing GmbH	Arzu Algulieva; Diāna Dušacka; Dārta Pūpola; Ilva Pole; Inita Balta; Jevgenijs Bodrenko; Jūlija Čevere; Natalja Mikena; Reinis Vangravs; Reinis Zeltmatis; Sergejs Ņikišins; Ģirts Šķenders
EPI_ISL_523962, EPI_ISL_523966	National Agency for Public Health, Republic of Moldova	Charite Universitatsmedizin Berlin, Institute of Virology	Ala Halacu; Barbara Mühlemann; Barbara Mühlemann; Christian Drosten; Elizabeta Jancheska; Golubinka Bosevska; Joern Beheim-Schwarzbach; Jörn Beheim-Schwarzbach; Julia Schneider; Maja Kuzmanovska; Mariana Apostol; Talitha Veith; Terry Jones; Victor M Corman
EPI_ISL_2894992, EPI_ISL_2894993, EPI_ISL_2894994, EPI_ISL_2894995, EPI_ISL_2894996	National Agency for Public Health, Republic of Moldova	Charité Universitätsmedizin Berlin, Institut für Virologie	Ala Halacu; Barbara Mühlemann; Christian Drosten; Julia Schneider; Julia Tesch; Jörn Beheim-Schwarzbach; Mariana Apostol; Talitha Veith; Terry Jones; Tobias Bleicker; Victor M Corman
EPI_ISL_1805651, EPI_ISL_1805740, EPI_ISL_1805741	National Center for Communicable Diseases (NCCD) National Influenza Center	National Center for Communicable Diseases (NCCD) National Influenza Center	Ankhbayar S; Battur L; Bayasgalan N; Darmaa B; Hideka M; Khishigmunkh Ch; Mina N; Naranzul Ts; Nymadawa P; Seiichiro F; Shinji W; Tsogzolmaa G
EPI_ISL_1805697	National Center for Communicable Diseases (NCCD) National Influenza Center	National Centre for Disease Control (NCDC) National Influenza Center	Ankhbayar S; Battur L; Bayasgalan N; Darmaa B; Hideka M; Khishigmunkh Ch; Mina N; Naranzul Ts; Nymadawa P; Seiichiro F; Shinji W; Tsogzolmaa G
EPI_ISL_1233442	National Center for Infectious and Parasitic Diseases (NCIPD)	National Center for Infectious and Parasitic Diseases (NCIPD)	Alexiev et al
EPI_ISL_1718293, EPI_ISL_1718294	National Center of Disease Control and Prevention of the Republic of Armenia	Institute of Molecular Biology NAS RA, Republic of Armenia, Department of Bioengineering, BioinformaticsInstitute and Molecular Biology IBMPh RAU, Republic of Armenia	Andranik Chavushyan; Arsen Arakelyan; Diana Avetyan; Gayane Melik-Pashayan; Gisane Khachatyan; Hovsep Ghazaryan; Maria Nikoghosyan; Nelli Muradyan; Roksana Zakharyan; Shushan Sargrsyan; Siras Hakobyan; Tamara Sirunyan; Zaven Karalyan
EPI_ISL_1854607, EPI_ISL_1854610, EPI_ISL_1854624, EPI_ISL_1854625, EPI_ISL_1854638, EPI_ISL_1854647	National Center of Disease Control and Prevention of the Republic of Armenia	UW Virology Lab	Alexander Greninger; Anahit Hovhannisyan; Andranik Chavushyan; Anna Khazaryan; Arsen Arakelyan; Diana Avetyan; Gisane Khachatyan; Hong Xie; Hovsep Ghazaryan; Keith R. Jerome; Lasata Shrestha; Lyudmila Niazyan; Maria Nikoghosyan; Meei-Li Huang; Michelle Lin; Nelli Muradyan; Pavitra Roychoudhury; Roksana Zakharyan; Shah Mohamed Bakhash; Siras Hakobyan; Tamara Sirunyan
EPI_ISL_454572	National Center of Expertise	National Center for Expertise, Kazakhstan National Center for Biotechnology, Kazakhstan	; Abdaliyev Askar; Akhmetollayev Ilyas; Amirgazin Asylulan; Aushakhmetova Zabira; Kalendar Ruslan; Lutsay Viktoriya; Rakhmetova Akbota; Ramankulov Yerlan; Shevtsov Alexandr
EPI_ISL_454571	National Center of Expertise	National Center for Expertise, National Center for Biotechnology, Kazakhstan	; Abdaliyev Askar; Akhmetollayev Ilyas; Amirgazin Asylulan; Aushakhmetova Zabira; Kalendar Ruslan; Lutsay Viktoriya; Rakhmetova Akbota; Ramankulov Yerlan; Shevtsov Alexandr
EPI_ISL_1786307, EPI_ISL_2081876, EPI_ISL_2081915, EPI_ISL_2081935, EPI_ISL_2099343, EPI_ISL_2099441, EPI_ISL_2099454, EPI_ISL_2180209, EPI_ISL_2379203, EPI_ISL_2621269, EPI_ISL_2621400, EPI_ISL_2621428, EPI_ISL_2621449, EPI_ISL_2648098, EPI_ISL_2648102, EPI_ISL_2841870, EPI_ISL_2841883, EPI_ISL_2841901, EPI_ISL_2841982, EPI_ISL_2967059, EPI_ISL_2967087, EPI_ISL_2967113, EPI_ISL_2967132, EPI_ISL_2967172			
see above	National Center of Infectious and Parasitic Diseases	National Center of Infectious and Parasitic Diseases	Alexiev; Alexiev et al; Dimitrova; Dobrinov; Donchev; Grigorova I.; Grigorova L.; Hristova; Ivanov; Kantardjiev; Korsun; Philipova; Stoitsova; Stoykov; Trifonova
EPI_ISL_2332763	National Centre For Cell Science	National Centre For Cell Science – INSACOG	Ajay Pillai; Dhiraj Paul; INSACOG Consortium team; Manoj Kumar Bhat; Mitali Inamdar; Mohak P Gujare; Shivang P. Bhanushali; Yogesh Shouche
EPI_ISL_2459215, EPI_ISL_2459216, EPI_ISL_2459690, EPI_ISL_2459936	National Centre for Disease Control (NCDC) Biotechnology Division, Delhi	NCDC Delhi, Biotechnology Division INSACOG	Hema Gogia; Hemlata Lall; Kalaiarasan Ponnusamy; Mahesh S Dhar; Manoj K Singh; Meena Datta; Partha Rakshit; Preeti Madan; Priyanka Singh; Radhakrishnan V. S; Robin Marwal; Sandhya Kabra; Sujeet K Singh; Uma Sharma
EPI_ISL_1661758	National Food and Veterinary Risk Assessment Institute	Vilnius University Hospital Santaros Klinikos, Center of Laboratory Medicine	Daniel Naumovas; Dovile Ezerskyte; Gytis Dudas; Ingrida Olendraite; Laimonas Griskevicius; Ligita Raugaite; Mindaugas Stoskus; Monika Katenaite; Rimvydas Norvilas
EPI_ISL_1909873	National Food and Veterinary Risk Assessment Institute (NMVRVI)	National Public Health Surveillance Laboratory	Ana Steponkiene; Danas Baksa; Jelena Razmuk; Lukas Vasionis; Lukas Zemaitis; Migle Gabrielaite; Svajune Muralyte
EPI_ISL_528584, EPI_ISL_528585	National Genomics Core-Center for DNA Fingerprinting and Diagnostics	National Genomics Core- Center for DNA Fingerprinting and Diagnostics (NGC-CDFD)- DBT's PAN-INDIA-1000 Genome consortium	Abhijeeth Singh Thakur; Ashwin Dalal; Bala Pratyusha; Bathula Siddardha; Debashish Mitra; Divya Vashisht; G Shashikanth; Heena Shah; Lavanya Banda; Mohammad Mudassir; Murali Dharan Bashyam; Nalini Raghunathan; R Harinarayanan; Rajeshree Sanyal; Rashna Bhandari; Surya Chodisetty; Vinay Donipadi; Vineesha Oddi
EPI_ISL_2492627, EPI_ISL_2492631, EPI_ISL_2492642, EPI_ISL_2492643, EPI_ISL_2492664, EPI_ISL_2492697, EPI_ISL_2492710, EPI_ISL_2493032, EPI_ISL_2493033, EPI_ISL_2493049, EPI_ISL_2493052, EPI_ISL_2493054, EPI_ISL_2493056, EPI_ISL_2493057, EPI_ISL_2686025			
see above	National HIV Reference Laboratory, Ministry of Health, Public Health Institute of Malawi	CERI, Centre for Epidemic Response and Innvoation, Stellenbosch University and KRISP, KZN Research Innovation and Sequencing Platform, UKZN.	Auld A; Chilima B; Chiwaula M; Emmanuel SJ; Giandhari J; Kaba M; Kampira E; Kasambara W; Kim L; Lessells R; Maida A; Mvula B; Mwangomba W; Naidoo Y; Panja L; Pillay S; Tegally H; Wadonda N; Wilkinson E; de Oliveira T
EPI_ISL_1407121, EPI_ISL_1407184, EPI_ISL_1407198, EPI_ISL_2494782, EPI_ISL_2494844, EPI_ISL_2494924	National HIV Reference Laboratory, Ministry of Health, Public Health Institute of Malawi	KRISP, KZN Research Innovation and Sequencing Platform	Auld A; Chilima B; Chiwaula M; Emmanuel SJ; Giandhari J; Kaba M; Kampira E; Kasambara W; Kim L; Lessells R; Maida A; Mvula B; Mwangomba W; Naidoo Y; Panja L; Pillay S; Tegally H; Wadonda N; Wilkinson E; de Oliveira T
EPI_ISL_560387, EPI_ISL_560388, EPI_ISL_1677703, EPI_ISL_1677707, EPI_ISL_1677716	National Health Laboratory	Botswana Institute for Technology Research and Innovation	Dineo Emang Tshiamo. Gape Nyepetsi; Dineo Emang Tshiamo. Tefelo Thela; Gape Nyepetsi; Kefentse Arnold Tumedi; Madisa Mine; Maitshwarelo Ignatius Matsheka; Malebogo Kebabonye; Thongbotho Mphoyakgosi
EPI_ISL_622942, EPI_ISL_622976, EPI_ISL_622980, EPI_ISL_622984, EPI_ISL_622989, EPI_ISL_622991	National Health Laboratory Service	National Institute for Communicable Diseases of the National Health Laboratory Service	Allam M; Bhiman JN; Ismail A; Khumalo Z; Kwenda S; Mnyameni F; Mohale T; Mtshali P; Subramoney K
EPI_ISL_944143, EPI_ISL_944163, EPI_ISL_1250455, EPI_ISL_1817756, EPI_ISL_1817763, EPI_ISL_1817766, EPI_ISL_1817767, EPI_ISL_1817859, EPI_ISL_2086228, EPI_ISL_2086229, EPI_ISL_2086238, EPI_ISL_2086243, EPI_ISL_2494834, EPI_ISL_2494948			
see above	National Health Laboratory Service, South Africa	KRISP, KZN Research Innovation and Sequencing Platform	Emmanuel SJ; Giandhari J; Khan S; Laguda-Akingba O; Lessells R; Maslo C; Mdlalose K; Naidoo Y; Pillay S; Ramphal U; Sitharam L; Tegally H; Wilkinson E; York D; de Oliveira T
EPI_ISL_2617196, EPI_ISL_2617201, EPI_ISL_2617210, EPI_ISL_2688538, EPI_ISL_2688543, EPI_ISL_2688549, EPI_ISL_2688550, EPI_ISL_2688553, EPI_ISL_2688563, EPI_ISL_2688564, EPI_ISL_2688573, EPI_ISL_2899689, EPI_ISL_2899695, EPI_ISL_2899725, EPI_ISL_2899740			
see above	National Health Laboratory Service, South Africa	KRISP, KZn Research Innovation and Sequencing Platform	Giandhari Jennifer; Naidoo Yeshnee; Pillay Sureshnee; San James; Tegally Houriiyah; Tshabuila Derek; Wilkinson Eduan; Yajna Ramphal; de Oliveira Tulio
EPI_ISL_456597, EPI_ISL_456598, EPI_ISL_456601, EPI_ISL_456605, EPI_ISL_456610	National Health Laboratory, Timor-Leste	Microbiological Diagnostic Unit Public Health Laboratory, The Peter Doherty Institute for Infection and Immunity	Antonia da Costa, E.; Baird, R.; Barreto, I.; Caly, L.; Canisia, D.; Dakh, F.; Dolores de Jesus da Costa, M.; Douglas, N.; Francis, J.; Freeman, K.; Jayanti Pereira Tilman, A.; Marr, I.; Sait, M.; Salles de Sousa, A.; Schultz, M.; Seemann, T.; Sherry, N.; Soares da Silva, E.; Wapling, J.; Ximenes, J.
EPI_ISL_2379748	National Heart Institute	Institute for Medical Research, Infectious Disease Research Centre, National Institutes of Health, Ministry of Health Malaysia	Azizan MA; Kamel K; Mohd Zawawi Z; Ramly N; Robert F; Suppiah J; Thayan R
EPI_ISL_2434158	National Hospital for Tropical Diseases	Oxford University Clinical Research Unit, Hanoi	H.Rogier van Doorn on behalf of the OUCRU COVID-19 research group; Le Van Duyet; Nguyen Thi Hong Thuong; Nguyen Thi Kim Chi; Nguyen Thi Tam; Nguyen Thu Trang; Pham Ngoc Thach; Phan Manh Cuong; Thomas Kesteman; Van Dinh Trang
EPI_ISL_2455479, EPI_ISL_2455488, EPI_ISL_2455494, EPI_ISL_2455495, EPI_ISL_2455496, EPI_ISL_2455497, EPI_ISL_2455498, EPI_ISL_2455500			
see above	National Hospital for Tropical Diseases	Oxford University Clinical Research Unit, Hanoi, Vietnam	H.Rogier van Doorn on behalf of the OUCRU COVID-19 research group; Le Van Duyet; Nguyen Thi Hong Thuong; Nguyen Thi Kim Chi; Nguyen Thi Tam; Nguyen Thu Trang; Pham Ngoc Thach; Phan Manh Cuong; Thomas Kesteman; Van Dinh Trang
EPI_ISL_511898	National Hospital of Tropical Diseases	Oxford University Clinical Research Unit, Hanoi, Vietnam	H. Rogier van Doorn; Le Nguyen Minh Hoa; Nguyen Thi Hong Thuong; Nguyen Thi Ngoc Diep; Nguyen Thi Tam; Nguyen Thu Trang; Pham Ngoc Thach; Van Dinh Trang; Vu Thi Ngoc Bich; on behalf of the OUCRU COVID-19 research group
EPI_ISL_2695785	National Influenza Center (NIC). Virology department. Institut National d'Hygiène.	National Influenza Center (NIC). Virology department. Institut National d'Hygiène.	Abderrahmane BIMOUHEN; Hassan IHAZMAD; Hicham OUMZIL; Samira BENKERROUM; Zakia REGRAGUI; fatima EL FALAKI and Mohamed RHAJAOUI
EPI_ISL_486889	National Influenza Center, Bahrain	National Influenza Center, Bahrain	AlWasti, H.; Altaif, Z.; Shehab, F.; Zaed, A.
EPI_ISL_862077, EPI_ISL_862078, EPI_ISL_1014676, EPI_ISL_1014681, EPI_ISL_1993547, EPI_ISL_1993552, EPI_ISL_1993554, EPI_ISL_1993557			
see above	National Influenza Center, Virology Department	National Influenza Center	A Nejati; F Ajaminejad; F Ajaminejad and T Mokhtari Azad; J Yavarian; K Sadeghi; N Ghavvami; N Ghavvami and T Mokhtari Azad; NZ Shafiei Jandaghi; V Salimi

EPI_ISL_2285849, EPI_ISL_2285850, EPI_ISL_2285852, EPI_ISL_2285853, EPI_ISL_2285855, EPI_ISL_2285864, EPI_ISL_2285869, EPI_ISL_2535795, EPI_ISL_2535796, EPI_ISL_2873841, EPI_ISL_2873845, EPI_ISL_2873850, EPI_ISL_2873858, EPI_ISL_2873860, EPI_ISL_2873865, EPI_ISL_2873870, EPI_ISL_2873872			
see above	National Influenza Centre	National Influenza Centre	; Benjamin B. Lindsey; Benjamin H. Foulkes; Dennis Laryea; Ernest Asiedu; Franklin Asiedu-Bekoe; Gordon Awandare; Ivy A. Asante; Joseph Oliver-Commey; Joyce Ngoi; Linda Boatemaa; Lorreta Kwasah; Mathew D. Parker; Michael Marks; Mildred Adusei-Poku; Sharon Hsu; Thushan I de Silva; William K. Ampofo
EPI_ISL_476838	National Influenza Centre for Northern Greece	National Influenza Centre for Northern Greece	Maria Christoforidi
EPI_ISL_410301	National Influenza Centre, National Public Health Laboratory, Kathmandu, Nepal	The University of Hong Kong	Alfonso J. Rodriguez-Morales; Anup Bastola; Basu Dev Pandey; Bibek Kumar Lal; Daniel Chu; Haogao Gu; Leo Poon; Malik Peiris; Ranjit Sah; Runa Jha
EPI_ISL_402125	National Institute for Communicable Disease Control and Prevention (ICDC) Chinese Center for Disease Control and Prevention (China CDC)	National Institute for Communicable Disease Control and Prevention (ICDC) Chinese Center for Disease Control and Prevention (China CDC)	Chen; Dai; F.-H.; Hu, Y.; J.-H.; J.-J.; J.-L. and Zhu; Liu, Y.; Pei; Q.-M.; She; Song; T.-Y.; Tao; Tian; Wang; Wang, W.; Wu, F.; Xu, L.; Y.-L.; Y.-M.; Y.-Y.; Y.-Z.; Yu, B.; Z.-G.; Z.-W.; Zhang; Zhao, S.; Zheng
EPI_ISL_450297, EPI_ISL_450299, EPI_ISL_450300, EPI_ISL_450301, EPI_ISL_450495, EPI_ISL_470878, EPI_ISL_490281, EPI_ISL_514417, EPI_ISL_514419, EPI_ISL_515177, EPI_ISL_622930, EPI_ISL_2433725, EPI_ISL_2433832, EPI_ISL_2433834, EPI_ISL_2444005, EPI_ISL_2444020, EPI_ISL_2662723			
see above	National Institute for Communicable Diseases of the National Health Laboratory Service	National Institute for Communicable Diseases of the National Health Laboratory Service	Allam M; Amoako DG; Bhiman JN; Everatt J; Ismail A; Khumalo Z; Kwenda S; Mahlangu B; Mnguni A; Mnyameni F; Mohale T; Mtshali P; Ntuli N; Scheepers C; Subramoney K; van Heusden P
EPI_ISL_1914886, EPI_ISL_1914931	National Institute for Communicable Diseases,National Health Laboratory Services, Gauteng, South Africa	National Institute for Communicable Diseases of the National Health Laboratory Service	Amoako DG; Bhiman JN; Ismail A; Mahlangu B; Mohale T; Ntuli N; Scheepers C
EPI_ISL_1911458	National Institute for Food and Veterinary Risk Assessment (NMVRVI)	National Public Health Surveillance Laboratory	Ana Steponkiene; Danas Baksa; Jelena Razmuk; Lukas Vasionis; Lukas Zemaitis; Migle Gabrielaite; Svajune Muralyte
EPI_ISL_469254	National Institute for Viral Disease Control and Prevention, China CDC	Institute of Viral Disease Control and Prevention, China CDC	Chun Huang; Dayan Wang; George Fu Gao; Guizhen Wu; Li Zhao; Lijuan Chen; Peihua Niu , Baoying Huang; Roujian Lu; Wenbo Xu; Wenjie Tan; Wenling Wang; Yubai Bi
EPI_ISL_591270, EPI_ISL_591276, EPI_ISL_850947, EPI_ISL_850949	National Institute for Viral Disease Control and Prevention, China CDC	National Institute for Viral Disease Control and Prevention, China CDC	Cao Chen; Dayan Wang; George F.Gao; Hong Wang; Huilai Ma; Ji Wang; Jingdong Song; Jun Han; Kai Nie; Ruqin Gao; Shiwen Wang; Weimin Zhou; Wenbo Xu; Wenjie Tan; Xiang Zhao; Yang Song; Yanhai Wang; Yao Meng; Yenan Feng; Yong Zhang; Yuchao Wu; Zhaoguo Wang; Zhixiao Chen
EPI_ISL_709542	National Institute of Blood Diseases (NIBD), Molecular Biology Lab	Genomics Lab NIBD	Aneeta Shahni; Arshi Naz; Gul Sufaida; Samina Naz Mukry; Sayed Ali Raza; Shariq Ahmed; Tahir Sultan Shamsi
EPI_ISL_2107094, EPI_ISL_2107095, EPI_ISL_2107098, EPI_ISL_2308255, EPI_ISL_2308256, EPI_ISL_2308259	National Institute of Health (NIH) - Federal Government of Somalia	African Centre of Excellence for Genomics of Infectious Diseases (ACEGID), Redeemer's University	A.T.; Abechi; Ajogbasile; Akano; C.A.; C.T.; Eromon; F.V.; Folarin, O.; Happi; I.B.; J.N.; J.U.; K.O.; Kayode; Nosamiefan, I.; Oguzie; Olawoye; Olumade; Oluniyi; P.E.; P.S.; T.J.; Ugwu; Uwanibe
EPI_ISL_833130, EPI_ISL_1915513	National Institute of Health Research and Development	National Institute of Health Research and Development	A; AA; Adam; Agustiningsih; Arie Ardiansyah Nugraha; F; Fauzul Muna; Febriyani; Febriyanti; HA; HD; Hana Apsari Pawestri; Hariastuti; Hartanti Dian Ikawati; Herman; Herna; Holy Arif Wibowo; IL; Ikawati; Indalao; Irene Lorinda Indalao; J; K; KD; KNA; Kartika Dewi Puspa; Kindi Adam; Kipuw; Krisna Nur Andriana Pangesti; Kurniawati; Muna; Mursinah; N; NI; NK; NL; Natalie Laurencia Kipuw; Nelly Puspandari; Ni Ketut Susilarini; Nike Susanti; Nikmah; Nugraha; Nurika Hariastuti; Pangesti; Pawestri; Puspa; Puspandari; R; Ramadhany; Reni Herman; Ririn Ramadhany; Rizki; Rukminiati; Setiawaty; Soekarso; Subangkit; Susanti; Susilarini; T; Tati Febriyanti; Triyani Soekarso; UA; Ully Alfi Nikmah; V; Vivi Setiawaty.; Wibowo; Y; Yuni Rukminiati
EPI_ISL_985395, EPI_ISL_1116455, EPI_ISL_1279947, EPI_ISL_1279951, EPI_ISL_1279953, EPI_ISL_1279966, EPI_ISL_1279972, EPI_ISL_2190103, EPI_ISL_2308251, EPI_ISL_2932503			
see above	National Institute of Infectious Diseases-Prof. Dr. Matei Bals Molecular Diagnostics Laboratory	National Institute of Infectious Diseases-Prof. Dr. Matei Bals Molecular Diagnostics Laboratory	Andreea Tudor; Corina Casangiu; Dan Otelea; Leontina Banica; Marius Surleac; Ovidiu Vlaicu; Petre Milu; Robert Hohan; Simona Paraschiv
EPI_ISL_943552, EPI_ISL_955172, EPI_ISL_2796523, EPI_ISL_2796527	National Institute of Laboratory Medicine and Referral Center	Genomic Research Lab, BCSIR	; A. K. M. Shamsuzzaman; A. K. M. Shamsuzzaman; Abu Sayeed Mohammad Mahmud; Arifa Akram; Asish Kumar Ghosh; Barna Goswami; Eshrar Osman; Iffat Jahan; Mahmuda Yeasmin; Md. Ahasan Habib; Md. Ahashan Habib; Md. Kamrul Islam; Md. Maruf Ahmed Molla; Md. Murshed Hasan Sarkar; Md. Saddam Hossain; Md. Salim Khan; Md. Salim Khan.; Mohammad Mohi Uddin; Mohammad Samir Uzzaman; Shahina Akter; Tanjina Akhtar Banu; Tanjina Akhter Banu; Tasnim Nafisa; Tasnim Nafisa
EPI_ISL_2323222, EPI_ISL_2323258, EPI_ISL_2380403	National Institute of Public Health	Charles University, Faculty of Science, BIOCEV, OMICS Genomics	Blanka Hamplová; Ingrid Poláková; Jana Šmahelová; Jiří Novák; Magdalena Jančářová; Ruth Tachezy; Sebastian Cristian Treitli; Vladimír Hampl; Zoltán Füssy; Štěpánka Hrdá
EPI_ISL_2709618, EPI_ISL_2709627	National Institute of Public Health	Institute of Molecular Genetics CAS	Jan Pačes; Jana Šáchová; Lucie Pfeiferová; Martin Zmuda; Michal Kolář; Miluše Hradilová; Ondřej Moravčík
EPI_ISL_2687993	National Institute of Public Health	National Institute of Infectious Diseases-Prof. Dr. Matei Bals Molecular Diagnostics Laboratory	Corina Casangiu; Dan Otelea; Leontina Banica; Marius Surleac; Petre Milu; Robert Hohan; Simona Paraschiv
EPI_ISL_1492241, EPI_ISL_2324674, EPI_ISL_2324821, EPI_ISL_2324940	National Institute of Public Health	National Institute of Public Health	Alexander Nagy; Dusan Trnka; Helena Jirincova; Jaromira Vecerova; Timotej Suri
EPI_ISL_1828716, EPI_ISL_1828718, EPI_ISL_1828724, EPI_ISL_1971075, EPI_ISL_2562079	National Institute of Public Health	State Veterinary Institute Prague	A; D; H; J; Jirincova; Nagy; Suri; T; Trnka; Vecerova
EPI_ISL_2928004, EPI_ISL_2928005, EPI_ISL_2928006, EPI_ISL_2928007, EPI_ISL_2928008, EPI_ISL_2928009, EPI_ISL_2928010, EPI_ISL_2928011, EPI_ISL_2928012			
see above	National Institute of Public Health Burundi, WHO Burundi, ASLM Burundi, MRC/UVRI & LSHTM Uganda Research Unit	MRC/UVRI & LSHTM Uganda Research Unit, National Institute of Public Health Burundi, WHO Burundi, ASLM Burundi	Alexis Niyomwungere; Anatole Nkeshimana; Blessing T. Marondera; Butoyi Pascal; Cassien Nduwimana; Dan Lule Bugembe; Eric Kezakarayagwa; Francine Kabatesi; Jean Baptiste Niyibigira; Jerome Ndaruhutse; Jerome Nkurunziza; Joseph Nyandwi; Kouadio Theodore Yao; Matthew Cotten; My V.T. Phan; Xavier Crespin
EPI_ISL_1335479, EPI_ISL_1335489	National Laboratory for Health, Environment and Food, OMM, Celje	CISLD (Clinical Institute of Special Laboratory Diagnostics), University Children's Hospital, University Medical Center Ljubljana	Ana Grom; Barbara Jenko Bizjan; Jernej Kovač; Katarina Kozmos; Marko Pokorn; Maruša Debeljak; Robert Šket; Tadej Battelino; Tine Tesovnik
EPI_ISL_1664811, EPI_ISL_2001884	National Laboratory for Health, Environment and Food, OMM, Celje	NLZOH (National Laboratory for Health, Environment and Food) / CISLD (Clinical Institute of Special Laboratory Diagnostics), University Children's Hospital, University Medical Center Ljubljana	Aleksander Kocuvan; Aleksander Mahnic; Alenka Štorman; Ana Grom; Barbara Jenko Bizjan; Daša Kavka / Jernej Kovač; Kaja Tominc; Katarina Kozmos; Maja Rupnik; Marko Pokorn; Maruša Debeljak; Mateja Borinc; Maša Jarčič; Nika Gobec; Robert Šket; Sandra Janezic; Tadej Battelino; Tine Tesovnik; Tjasa Žohar Čretnik
EPI_ISL_2789267, EPI_ISL_2983030	National Laboratory for Health, Environment and Food, OMM, Koper	NLZOH (National Laboratory for Health, Environment and Food) / CISLD (Clinical Institute of Special Laboratory Diagnostics), University Children's Hospital, University Medical Center Ljubljana	Aleksander Kocuvan; Aleksander Mahnic; Alenka Štorman; Ana Grom; Barbara Jenko Bizjan; Gašper Strugar; Kaja Tominc; Katarina Kozmos; Maja Rupnik; Marko Pokorn; Maruša Debeljak; Maša Jarčič; Mitja Rak / Jernej Kovač; Nika Gobec; Robert Šket; Sandra Janezic; Tadej Battelino; Tina Cvetković; Tine Tesovnik; Tjaša Žohar Čretnik
EPI_ISL_2162621	National Laboratory for Health, Environment and Food, OMM, Kranj	NLZOH (National Laboratory for Health, Environment and Food) / CISLD (Clinical Institute of Special Laboratory Diagnostics), University Children's Hospital, University Medical Center Ljubljana	Aleksander Kocuvan; Aleksander Mahnic; Alenka Štorman; Ana Grom; Barbara Jenko Bizjan; Kaja Tominc; Katarina Kozmos; Maja Rupnik; Marjana Petrevčič / Jernej Kovač; Marko Pokorn; Maruša Debeljak; Mateja Ravnik; Maša Jarčič; Monika Korošec; Nika Gobec; Robert Šket; Sandra Janezic; Tadej Battelino; Tine Tesovnik; Tjasa Žohar Čretnik
EPI_ISL_1111786, EPI_ISL_1111889	National Laboratory for Health, Environment and Food, OMM, Maribor	CISLD (Clinical Institute of Special Laboratory Diagnostics), University Children's Hospital, University Medical Center Ljubljana	Ana Grom; Barbara Jenko Bizjan; Jernej Kovač; Katarina Kozmos; Marko Pokorn; Maruša Debeljak; Robert Šket; Tadej Battelino; Tine Tesovnik
EPI_ISL_1664818, EPI_ISL_2162480, EPI_ISL_2162562, EPI_ISL_2162583, EPI_ISL_2463348, EPI_ISL_2463442, EPI_ISL_2789297, EPI_ISL_2982921			
see above	National Laboratory for Health, Environment and Food, OMM, Maribor	NLZOH (National Laboratory for Health, Environment and Food) / CISLD (Clinical Institute of Special Laboratory Diagnostics), University Children's Hospital, University Medical Center Ljubljana	Aleksander Kocuvan; Aleksander Mahnic; Alenka Štorman; Ana Grom; Andrej Golle / Jernej Kovač; Barbara Jenko Bizjan; Kaja Tominc; Katarina Kozmos; Maja Rupnik; Marko Pokorn; Maruša Debeljak; Maša Jarčič; Mojca Cimerman; Nika Gobec; Nika Volmajer; Robert Šket; Sandra Janezic; Tadej Battelino; Tine Tesovnik; Tjasa Žohar Čretnik; Tjaša Žohar Čretnik
EPI_ISL_2789283	National Laboratory for Health, Environment and Food, OMM, Novo mesto	NLZOH (National Laboratory for Health, Environment and Food) / CISLD (Clinical Institute of Special Laboratory Diagnostics), University Children's Hospital, University Medical Center Ljubljana	Aleksander Kocuvan; Aleksander Mahnic; Alenka Štorman; Ana Grom; Barbara Jenko Bizjan; David Cvetko; Kaja Tominc; Katarina Kozmos; Maja Rupnik; Marko Pokorn; Maruša Debeljak; Matjaž Retelj / Jernej Kovač; Maša Jarčič; Nika Gobec; Robert Šket; Sandra Janezic; Tadej Battelino; Tatjana Harlander; Tine Tesovnik; Tjaša Žohar Čretnik
EPI_ISL_512612, EPI_ISL_512613, EPI_ISL_512636	National Laboratory for Influenza/Virology reference laboratory, Public Health Center of the Ministry of Health of Ukraine	Respiratory Virus Unit, Microbiology Services Colindale, Public Health England	Dr. Iryna Demchyshyna; PHE Covid Sequencing Team
EPI_ISL_647980, EPI_ISL_1191831, EPI_ISL_1191921, EPI_ISL_1192066	National Microbiology Reference Laboratory	Quadram Institute Bioscience	Agnes Juru; Alexander Goredema; Ana-Victoria Gutierrez; Andrew J. Page; Andrew Tarupiwa; Barbra Murwira; Beuty Makamure; Charles Nyagupe; David Baker; Faustinos T Takawira; Gaetan Thilliez; Gemma Kay; Gibson Mhlanga; Hlanai Gumbo; Isaac Phiri; Justin O'Grady; Kenneth K Maeka; Leonardo de Oliveira Martins; Muchaneta Mugabe; Portia Manangazira; Raiva Simbi; Robert Kingsley; Sekesai Zinyowera; Tapfumanei Mashe; Tatenda Takawira; Thanh Le Viet
EPI_ISL_2492582, EPI_ISL_2492707, EPI_ISL_2492718, EPI_ISL_2492732, EPI_ISL_2492775, EPI_ISL_2492860, EPI_ISL_2492916			

see above	National Microbiology Reference Laboratory, Ministry of Health, Harare, Zimbabwe	CERI, Centre for Epidemic Response and Innvoation, Stellenbosch University and KRISP, KZN Research Innovation and Sequencing Platform, UKZN.	Agnes Juru; Air Comodor Dr J. Chimedza; Charles Nyagupe; Dr Raiva Simbi; Emmanuel SJ; Giandhari J; Hlanai Gumbo; Kenneth Maeka; Naidoo Y; Pillay S; Tapfumanei Mashe; Tatenda Takawira; Tegally H; Wilkinson E; de Oliveira T
EPI_ISL_1095620	National Public Health Center, COVID Laboratory	National Public Health Center, National Biosafety Laboratory	Bernadett Pályi; Dániel Déri; Judit Henczkó; Norbert Solymosi; Nóra Magyar; Zoltán Kis
EPI_ISL_2479956, EPI_ISL_2479957, EPI_ISL_2479972, EPI_ISL_2479975, EPI_ISL_2479981, EPI_ISL_2479985	National Public Health Laboartory	CSIR-Institute of Genomics and Integrative Biology	Jyoti Acharya; Lokbandhu Chaudhary; Priya Jha; Runa Jha; Suni Dangol
EPI_ISL_2674091, EPI_ISL_2674102	National Public Health Laboratory	National Public Health Laboratory/CSIR-Institute of Genomics and Integrative Biology	National Public Health Laboratory Team
EPI_ISL_845548, EPI_ISL_845549, EPI_ISL_845550, EPI_ISL_845551, EPI_ISL_845563	National Public Health Laboratory, Cameroon	African Centre of Excellence for Genomics of Infectious Diseases (ACEGID), Redeemer's University	Oluniyi P.E. et al
EPI_ISL_2450778, EPI_ISL_2450783, EPI_ISL_2450789, EPI_ISL_2450791	National Public Health Laboratory, Ministry of Health, Ministry of Health, Republic of South Sudan	South Sudan Ministry of Health, WHO South Sudan, MRC/UVRI & LSHTM Uganda Research Unit	Abe G. Abias; Dan Lule Bugembe; Dennis Kenyi Lodiongo; James Ayei; John Rumunu; Joseph Francis Wamala; Juma John HM; Lul Lojok Deng; Matthew Cotten; My V.T. Phan; Pontiano Kaleebu; Richard Lino Loro Lako; Sudhir Bunga
EPI_ISL_428822, EPI_ISL_428830, EPI_ISL_428841, EPI_ISL_435678, EPI_ISL_435686, EPI_ISL_462300, EPI_ISL_493408, EPI_ISL_498594, EPI_ISL_498599, EPI_ISL_516827, EPI_ISL_536427, EPI_ISL_536432, EPI_ISL_548976, EPI_ISL_574541, EPI_ISL_626631, EPI_ISL_626636, EPI_ISL_645124, EPI_ISL_693316, EPI_ISL_693328, EPI_ISL_754076, EPI_ISL_754080, EPI_ISL_1098834, EPI_ISL_1519384, EPI_ISL_1652095, EPI_ISL_1652199, EPI_ISL_1704809, EPI_ISL_1816946, EPI_ISL_1816953, EPI_ISL_1816955, EPI_ISL_1897609, EPI_ISL_1897620, EPI_ISL_2349711, EPI_ISL_2349779, EPI_ISL_2349819, EPI_ISL_2349865, EPI_ISL_2508703, EPI_ISL_2508993, EPI_ISL_2508995, EPI_ISL_2509024, EPI_ISL_2509025, EPI_ISL_2509027, EPI_ISL_2509028, EPI_ISL_2509091, EPI_ISL_2621909, EPI_ISL_2621948, EPI_ISL_2621955, EPI_ISL_2710081, EPI_ISL_2790332, EPI_ISL_2887792, EPI_ISL_2982247, EPI_ISL_2982249, EPI_ISL_3026034			
see above	National Public Health Laboratory, National Centre for Infectious Diseases	National Public Health Laboratory, National Centre for Infectious Diseases	Chavatte JM; Chavatte Jean-Marc; Cui L; Cui Lin; Grace Jie Yin Ngan; Katherine Ching; Lin Cui; Lin RTP; Lin Raymond Tzer Pin; Mak TM; Mak Tze Minn; Octavia S; Octavia Sophie; Raymond Tzer Pin Lin; Royce Ang; Sophie Octavia; Tze Minn Mak; Zhenyang Zhou; Zhou Z
EPI_ISL_2674074	National Public Health Laboratory/B.P Koirala Institute of Health Science	National Public Health Laboratory/CSIR-Institute of Genomics and Integrative Biology	National Public Health Laboratory Team /B.P Koirala Institute of Health Science COVID 19 PCR Team
EPI_ISL_2674069	National Public Health Laboratory/Palpa HospitalCOVID-19 Lab	National Public Health Laboratory/CSIR-Institute of Genomics and Integrative Biology	National Public Health Laboratory Team /Palpa HospitalCOVID-19 PCR Team
EPI_ISL_2674065	National Public Health Laboratory/Pokhara Academy of Health Sciences	National Public Health Laboratory/CSIR-Institute of Genomics and Integrative Biology	National Public Health Laboratory Team /Pokhara Academy of Health Sciences COVID 19 PCR Team
EPI_ISL_2674086	National Public Health Laboratory/Provincial Public Health Laboratory -2	National Public Health Laboratory/CSIR-Institute of Genomics and Integrative Biology	National Public Health Laboratory Team /Provincial Public Health Laboratory -2 Team
EPI_ISL_2674070	National Public Health Laboratory/Provincial Public Health Laboratory -Gandaki	National Public Health Laboratory/CSIR-Institute of Genomics and Integrative Biology	National Public Health Laboratory Team /Provincial Public Health Laboratory -Gandaki Team
EPI_ISL_2674108	National Public Health Laboratory/Provincial Public Health Laboratory -Lumbini	National Public Health Laboratory/CSIR-Institute of Genomics and Integrative Biology	National Public Health Laboratory Team /Provincial Public Health Laboratory -Lumbini Team
EPI_ISL_2349378	National Reference Centre for Retroviruses	Central Public Health Laboratory	Gkikas Magiorkinis et al
EPI_ISL_480301, EPI_ISL_480304	National Reference Laboratory "Influenza and acute respiratory diseases"	NRL-HIV	Ivailo Alexiev; Ivan Ivanov; Ivva Philipova
EPI_ISL_1273394, EPI_ISL_1273395, EPI_ISL_1273396, EPI_ISL_1273399, EPI_ISL_1273406, EPI_ISL_1273408	National Reference Laboratory - Ministry of Health Maseru Lesotho	National Institute for Communicable Diseases of the National Health Laboratory Service	Amoako DG; Banda R; Bhiman JN; Gorova V; Ismail A; Mahlangu B; Mathabo M; Mohale T; Mooko M; Ntuli N; Scheepers C
EPI_ISL_737200, EPI_ISL_737201	National Reference Laboratory, Nigeria Centre for Disease Control.	National Reference Laboratory, Nigeria Centre for Disease Control, Gaduwa,Abuja, Nigeria	Akinpelu Afolabi; Anthony Ahumibe; Catherine Okoi; Celestina Obiekea; Chimaobi Chukwu; Dr Adesuyi Omoare; Dr Chikwe Ihekweazu; Dr Chinwe Ochu; Dr Ndodo Nnaemeka; Dr Sikiru Badaru; Grace Esebanmen; Kingsley Njoku; Nwando Mba; Olusola Akanbi; Shirlee Wohl; oluwaseyi Ajegbe
EPI_ISL_435677, EPI_ISL_962877	National Virology Reference Laboratory	National Public Health Laboratory, National Centre for Infectious Diseases	Chavatte Jean-Marc; Cui Lin; Lin Cui; Lin Raymond Tzer Pin; Mak Tze Minn; Octavia Sophie; Raymond Tzer Pin Lin; Taib Surita; Tze Minn Mak; Zaini Zainun; Zhenyang Zhou
EPI_ISL_981252	National Virus Reference Laboratory	Irish Coronavirus Sequencing Consortium - Helixworks	Conor Crosbie; Nimesh Pinnamaneni; Sachin Chalapati
EPI_ISL_500592, EPI_ISL_501261, EPI_ISL_512102, EPI_ISL_525416, EPI_ISL_528457, EPI_ISL_578207, EPI_ISL_671885, EPI_ISL_671920, EPI_ISL_959858, EPI_ISL_1785368, EPI_ISL_1891139, EPI_ISL_1960680, EPI_ISL_1960901, EPI_ISL_1972834, EPI_ISL_2029170, EPI_ISL_2029356, EPI_ISL_2240983, EPI_ISL_2533387, EPI_ISL_2601258, EPI_ISL_2649721, EPI_ISL_2710562, EPI_ISL_2821177, EPI_ISL_2840952, EPI_ISL_2841044, EPI_ISL_2862431, EPI_ISL_2893050, EPI_ISL_2893164, EPI_ISL_2932096, EPI_ISL_3011232, EPI_ISL_3011525, EPI_ISL_3030768			
see above	National Virus Reference Laboratory	National Virus Reference Laboratory	Aditi Chaturvedi; Calum Walsh; Charlene Bennet; Charlene Bennett; Cillian F De Gascun; Daniel Hare; Fiona Crispie; Gabriel Gonzalez; Guerrino Macori; Jonathan Dean; Matthew McCabe; Michael Carr; Paul Cotter; Seamus Fanning; Suzie Coughlan; Zoe Yandle
EPI_ISL_1627088	National reference Laboratory, NCDC, Gaduwa, Abuja, Nigeria	National reference Laboratory, NCDC, Gaduwa, Abuja, Nigeria	Akinpelu Afolabi; Anthony Ahumibe; Badaru Sikiru; Catherine Okoi; Celestina Obiekea; Chimaobi Chukwu; Dr Chikwe Ihekweazu; Dr Grace Esebanmen; Dr Ndodo Nnaemeka; Dr Omoare Adesuyi; Kingsley Njoku; Nwando Mba; Olusola Anuoluwapo Akanbi
EPI_ISL_2533832, EPI_ISL_2533868, EPI_ISL_2533869, EPI_ISL_2533870, EPI_ISL_2533871, EPI_ISL_2533872, EPI_ISL_2533873, EPI_ISL_2533874, EPI_ISL_2533875, EPI_ISL_2533876, EPI_ISL_2533882, EPI_ISL_2858619, EPI_ISL_2858622, EPI_ISL_2858631, EPI_ISL_2858678, EPI_ISL_2858680, EPI_ISL_2858685, EPI_ISL_2860831, EPI_ISL_2860889			
see above	Naval Medical Research Unit No. 3	Naval Medical Research Center Biological Defense Research Directorate	Andrea E. Luquette; Andrew J. Bennett; Bishwo N. Adhikari; Catherine E. Arnold; Chaselynn M. Watters; Emily K. Stefanov; Francisco Malagon; Kyle A. Long; Logan J. Voegtly; Luis A. Estrella; Michael V. Deschenes; Regina Z. Cer; Stephen M. Eggan; and Kimberly A. Bishop-Lilly
EPI_ISL_2754090, EPI_ISL_2967911	Nebraska Public Health Laboratory	NPHL COVID-19 Response Team	NPHL COVID-19 Response Team
EPI_ISL_754068, EPI_ISL_754070	Nepal Korea Friendship Municipality Hospital	Nepal Health Research Council	Meghnath Dhimal; Pradip Gyanwali
EPI_ISL_515391, EPI_ISL_1483251, EPI_ISL_1483264, EPI_ISL_3021067	Nevada State Public Health Laboratory	Nevada State Public Health Laboratory	Andrew Gorzalski; Chris Laverdure; Cyprian Rossetto; David Jackson; Heather Kerwin; Joel R. Sevinsky; Mark Pandori; Natalie Crawford; Paul Hartley; Richard Tillett; Stephanie Van Hooser; Subhash C. Verma; and Mark Pandori
EPI_ISL_2202898	New Mexico Department of Health Scientific Laboratory Division	Los Alamos National Laboratory Bioscience Division	Alina Deshpande; Cheryl Gleasner; Chien-Chi Lo; Joseph Hicks; Kim McMurry; Michael Edwards; Migun Shakya; Patrick Chain; Twila Kunde
EPI_ISL_1660402, EPI_ISL_1660408, EPI_ISL_1660411, EPI_ISL_1697281, EPI_ISL_1756023, EPI_ISL_2107449, EPI_ISL_2107451, EPI_ISL_2107456, EPI_ISL_2107457, EPI_ISL_2107535, EPI_ISL_2811816			
see above	New South Wales Health Pathology Royal Prince Alfred Hospital	Microbiology RPAH	Au, J.; Bull, R.; Deveson, I.; Foster, C.; Rawlinson, W.; Ruiz Silva, M.; Van Hal, S.
EPI_ISL_1196008	Nhlangano Health Centre	National Institute for Communicable Diseases of the National Health Laboratory Service	Amoako DG; Bhiman JN; Ismail A; Mahlangu B; Maphalala GP; Mohale T; Ntuli N; Scheepers C
EPI_ISL_516934	Nicolae Testemitanu State University of Medicine and Pharmacy	International Centre for Genetic Engineering and Biotechnology (ICGEB) and ARGO Open Lab Platform for Genome Sequencing	Dal Monego S; Licastro D; Marcello A; Rajasekharan S; Ulinici M
EPI_ISL_1073639, EPI_ISL_1073640, EPI_ISL_1381746	Nigeria Centre For Disease Control	National reference Laboratory, NCDC, Gaduwa, Abuja	Anthony Ahumibe; Catherine Okoi; Celestina Obiekea; Chimaobi Chukwu; Dr Chikwe Ihekweazu; Dr Ndodo Nnaemeka; Dr Omoare Adesuyi; Grace Esebanmen; Naidoo Dhamari; Nwando Mba; Olusola Anuoluwapo Akanbi
EPI_ISL_2565890, EPI_ISL_2565894	Nigeria Centre for Disease Control (NCDC)	Africa Centre for Excellence for Genomics of Infectious Diseases (ACEGID), Redeemer's University	A.T.; Abechi; Ajogbasile; Akano; C.A.; C.T.; Eromon; F.V.; Folarin, O.; Happi; I.B.; J.N.; J.U.; K.O.; Kayode; Nosamiefan, I.; Oguzie; Olawoye; Olumade; Oluniyi; P.E.; P.S.; T.J.; Ugwu; Uwanibe
EPI_ISL_1970548	Nigeria Centre for Disease Control (NCDC)	African Centre for Excellence for Genomics of Infectious Diseases (ACEGID), Redeemer's University	A.T.; Abechi; Ajogbasile; Akano; C.A.; C.T.; Eromon; F.V.; Folarin, O.; Happi; I.B.; J.N.; J.U.; K.O.; Kayode; Oguzie; Olawoye; Olumade; Oluniyi; P.E.; P.S.; T.J.; Ugwu; Uwanibe
EPI_ISL_872604, EPI_ISL_872608, EPI_ISL_872610, EPI_ISL_906285, EPI_ISL_906297, EPI_ISL_941276, EPI_ISL_941277, EPI_ISL_941279, EPI_ISL_941285, EPI_ISL_1242022, EPI_ISL_1242023, EPI_ISL_1242025			
see above	Nigeria Centre for Disease Control (NCDC)	African Centre of Excellence for Genomics of Infectious Diseases (ACEGID), Redeemer's University	Oluniyi P.E. et al
EPI_ISL_455362, EPI_ISL_455412, EPI_ISL_487092, EPI_ISL_487098, EPI_ISL_487101, EPI_ISL_527872, EPI_ISL_527879, EPI_ISL_527881, EPI_ISL_527882, EPI_ISL_527884, EPI_ISL_527887, EPI_ISL_527895, EPI_ISL_527909, EPI_ISL_527914, EPI_ISL_729921, EPI_ISL_729922, EPI_ISL_729924, EPI_ISL_729925, EPI_ISL_729926, EPI_ISL_729929, EPI_ISL_729946, EPI_ISL_729951, EPI_ISL_729962, EPI_ISL_729968, EPI_ISL_729979, EPI_ISL_729982, EPI_ISL_729985, EPI_ISL_730004, EPI_ISL_730016, EPI_ISL_730023, EPI_ISL_730031, EPI_ISL_730043			
see above	Nigeria Centre for Disease Control (NCDC)	African Centre of Excellence for Genomics of Infectious Diseases (ACEGID), Redeemer's University, Ede, Osun State, Nigeria	Ajogbasile F.V.; Folarin O.A.; Happi C.T.; Ihekweazu C.; Kayode A.; Oguzie J.; Olawoye I.; Olumade T.; Oluniyi P.E.; Oluniyi P.E. et al; Uwanibe J.
EPI_ISL_2240754,	Nigeria Centre for Disease Control (NCDC)	African Centre of Excellence for Genomics of Infectious	A.T.; Abechi; Ajogbasile; Akano; C.A.; C.T.; Eromon; F.V.; Folarin, O.; Happi; I.B.; J.N.; J.U.; K.O.; Kayode; Nosamiefan, I.; Oguzie; Olawoye; Olumade; Oluniyi; P.E.; P.S.; T.J.; Ugwu; Uwanibe

EPI_ISL_2285324		Diseases (ACEGID), Redeemer's University	
EPI_ISL_1093445, EPI_ISL_1093475	Nigerian Centre for Disease Control (NCDC)	African Centre of Excellence for Genomics of Infectious Diseases (ACEGID), Redeemer's University	I.B.; Olawoye; et al
EPI_ISL_2230650	Noblis	Noblis	June Kang; Katharine Jennings; Lauren Brinkac; Lee F. Kolakowski; Shane Mitchell; Shiela Diepold; William M. Nelson
EPI_ISL_2645140	NordLab Kokkola	Expert Microbiology, National Institute for Health and Welfare	Carita Savolainen-Kopra; Erika Lindh; Haider al-Hello; Jani Halkilahti; Kirsi Liitsola; Niina Ikonen; Olli Vapalahti; Pekka Ellonen; Phuoc Truong; Päivi Laurila; Ravi Kant; Sari Hannula; Soile Blomqvist; Teemu Smura
EPI_ISL_2507749, EPI_ISL_2507872, EPI_ISL_2507873, EPI_ISL_2507875, EPI_ISL_2507876, EPI_ISL_2645062, EPI_ISL_2645166, EPI_ISL_3020668			
see above	NordLab Oulu	Expert Microbiology, National Institute for Health and Welfare	Carita Savolainen-Kopra; Erika Lindh; Haider al-Hello; Jani Halkilahti; Kirsi Liitsola; Niina Ikonen; Olli Vapalahti; Pekka Ellonen; Phuoc Truong; Päivi Laurila; Ravi Kant; Sari Hannula; Soile Blomqvist; Teemu Smura
EPI_ISL_1599388, EPI_ISL_2333076, EPI_ISL_2509363, EPI_ISL_2725101	Nordland Hospital - Bodo, Laboratory Department, Molecular Biology Unit	Norwegian Institute of Public Health, Department of Virology	'Kathrine Stene-Johansen; Atiya R Ali; Debech Nadia; Engebretsen Serina Beate; Garcia Llorente Ignacio; Hilde Elshaug; Hilde Vollan; Jon Bråte; Kamilla Heddeland Instefjord; Karoline Bragstad; Kathrine Stene-Johansen; Line Victoria Moen; Marie Paulsen Madsen; Olav Hungnes; Pedersen Benedikte Nevjen; Rasmus Riis Kopperud
EPI_ISL_534243	Norra Alvsborgs lanssjukhus	The Public Health Agency of Sweden	Anna Risberg; Anna-Malin Linde; Karin Tegmark-Wisell; Maria Lind Karlberg; Mattias Haukland; Mia Brytting; Olov Svartstrom; Oskar Karlsson Lindsjo; Petra Edquist; Reza Advani; Sandra Broddesson
EPI_ISL_2931369	North Lantau Hospital	Hong Kong Department of Health	Alan K.L. Tsang; Edman T.K. Lam; Ken H.L. Ng; Peter C.W. Yip; Rickjason C.W. Chan
EPI_ISL_456396, EPI_ISL_548060, EPI_ISL_579406, EPI_ISL_579407	North Shore Hospital	Institute of Environmental Science and Research (ESR)	Anja Werno; Antje van der Linden; Arlo Upton; Chris Mansell; David Hammer; Dragana Drinkovic; Erasmus Smit; Gary McAuliffe; Hana Sofia Andersson; Hermes Perez; James Ussher; Jill Sherwood; Jing Wang; Joep de Ligt; Josh Freeman; Julia Howard; Juliet Elvy; Lauren Jelly; Mary DeAlmeida; Matt Blakiston; Matt Storey; Matthew Rogers; Max Bloomfield; Michael Addidle; Michelle Balm; Muhammad Faisal; Nikki Freed; Olin Silander; Sally Roberts; Sarah Jefferies; Sharmini Muttaiyah; Susan Morpeth; Susan Taylor; Timothy Blackmore; Vani Sathyendran; Veronica Playle; Virginia Hope; Xiaoyun Ren
EPI_ISL_936968	Northwestern Memorial Hospital	Ozer Lab	Chad J. Achenbach; Egon A. Ozer; Judd F. Hultquist; Lacy M. Simons; Lawrence J. Jennings; Michael G. Ison; Ramon Lorenzo-Redondo
EPI_ISL_2788600	Norwegian Institute of Public Health, Department of Virology	Norwegian Institute of Public Health, Department of Virology	Atiya R Ali; Debech Nadia; Engebretsen Serina Beate; Garcia Llorente Ignacio; Hilde Elshaug; Hilde Vollan; Jon Bråte; Kamilla Heddeland Instefjord; Karoline Bragstad; Kathrine Stene-Johansen; Line Victoria Moen; Marie Paulsen Madsen; Olav Hungnes; Pedersen Benedikte Nevjen; Rasmus Riis Kopperud
EPI_ISL_735444, EPI_ISL_735448	Nucleic Acid Testing - Rwanda National Reference Laboratory	GIGA Medical Genomics	Bouchra Boujemla; Esperence Umumararungu; Jacob Souopgui; Keith Durkin; Léon Mutesa; Maria Artesi; Marie-Pierre Hayette; Patrick Tuyisenge; Robert Rutayisire; Sabin Nsanzimana; Swaibu Gatare; Sébastien Bontems; Vincent Bours; Yvan Butera
EPI_ISL_925896, EPI_ISL_925907, EPI_ISL_925910, EPI_ISL_930634, EPI_ISL_960228, EPI_ISL_960237, EPI_ISL_960284, EPI_ISL_960300, EPI_ISL_960301, EPI_ISL_960302, EPI_ISL_1301736, EPI_ISL_1301778, EPI_ISL_1302681, EPI_ISL_2362498, EPI_ISL_2362515, EPI_ISL_2362523, EPI_ISL_2362524, EPI_ISL_2362525, EPI_ISL_2362526, EPI_ISL_2365914, EPI_ISL_2521981, EPI_ISL_2521982, EPI_ISL_2521987, EPI_ISL_2521988, EPI_ISL_2521989, EPI_ISL_2521992, EPI_ISL_2521993, EPI_ISL_2521994, EPI_ISL_2521995, EPI_ISL_2521996, EPI_ISL_2521997, EPI_ISL_2522092, EPI_ISL_2522096, EPI_ISL_2811717, EPI_ISL_2811720, EPI_ISL_2811724, EPI_ISL_2820242, EPI_ISL_2828472, EPI_ISL_2828474, EPI_ISL_2828475, EPI_ISL_2828477, EPI_ISL_2828478, EPI_ISL_2828481, EPI_ISL_2828483, EPI_ISL_2828484, EPI_ISL_2828486, EPI_ISL_2828488, EPI_ISL_2828489, EPI_ISL_2828490, EPI_ISL_2828491, EPI_ISL_2828492, EPI_ISL_2828493, EPI_ISL_2828494, EPI_ISL_2828495, EPI_ISL_2828496, EPI_ISL_2828497, EPI_ISL_2828503, EPI_ISL_2828506, EPI_ISL_2828508, EPI_ISL_2828509, EPI_ISL_2828510, EPI_ISL_2828511, EPI_ISL_2828512, EPI_ISL_2828514, EPI_ISL_2828515, EPI_ISL_2863937, EPI_ISL_2863942, EPI_ISL_3012179, EPI_ISL_3012181, EPI_ISL_3012182, EPI_ISL_3012184, EPI_ISL_3012189, EPI_ISL_3012190, EPI_ISL_3012191, EPI_ISL_3012192, EPI_ISL_3012193, EPI_ISL_3012194, EPI_ISL_3012198, EPI_ISL_3012199, EPI_ISL_3012200, EPI_ISL_3012201, EPI_ISL_3012203, EPI_ISL_3012204, EPI_ISL_3012206, EPI_ISL_3012209, EPI_ISL_3012212, EPI_ISL_3012217, EPI_ISL_3012219, EPI_ISL_3012220, EPI_ISL_3012221, EPI_ISL_3012222, EPI_ISL_3012223, EPI_ISL_3012224, EPI_ISL_3012225, EPI_ISL_3012227, EPI_ISL_3012228, EPI_ISL_3012230, EPI_ISL_3012232, EPI_ISL_3012233, EPI_ISL_3012234, EPI_ISL_3021476, EPI_ISL_3021477, EPI_ISL_3021478			
see above	Nucleic Acid Testing, National Reference Laboratory	GIGA Medical Genomics	Bouchra Boujemla; Corinne Fasquelle; Esperence Umumararungu; Jacob Souopgui; Keith Durkin; Léon Mutesa; Maria Artesi; Marie-Pierre Hayette; Nathalie Renotte; Patrick Tuyisenge; Reuben Sindayiheba; Robert Rutayisire; Sabin Nsanzimana; Swaibu Gatare; Sébastien Bontems; Vincent Bours; Yvan Butera
EPI_ISL_2779341, EPI_ISL_2779342	Nyamira County Referral Hopital	USAMRD-A, Basic Science Laboratory	Alan Lemtudo; Beth Mutai; Brian Andika; Carol Kifude; Clement Masakwe; Eric Muthanje; Esther Omuseni; Faith Sigei; Gathii Kimita; George Awinda; John Waitumbi; Josphat Nyataya; Rachel Githii; Rehema Liyai; Stephen Ochola
EPI_ISL_2372331, EPI_ISL_2372338, EPI_ISL_2372377, EPI_ISL_2372402	Nyangabwe Hospital HIV Reference Laboratory	Botswana Harvard HIV Reference Laboratory	Boitumelo Zuze; Botshelo Radibe; Dorcas Maruapula; Joseph Makhema; Keoratile Ntshambiwa; Kgomotso Moruisi; Legodile Kooepile; Madisa Mine; Mosepele Mosepele; Ontlametse T. Bareng; Pamela Smith-Lawrence; Roger Shapiro; Shahin Lockman; Sikhulile Moyo; Simani Gaseitsiwe; Thongbotho Mphoyakgosi; Wonderful T. Choga
EPI_ISL_667345	OHSU Lab Services Molecular Microbiology Lab	Oregon SARS-CoV-2 Genome Sequencing Center	Alec J. Hirsch; Andrew C. Adey; Benjamin N. Bimber; Brendan L. O'Connell; Brian J. O'Roak; Daniel N. Streblow; Donna Hansel; Guang Fan; Ruth V. Nichols; Sally Grindstaff; William B. Messer
EPI_ISL_2545564	OKMI FN Brno	CEITEC MU	Bystry V.; Deissova T.; Dolejska M.; Machackova T.; Pardy F.; Zdrazilova Dubska L.
EPI_ISL_1366742, EPI_ISL_2372266	OUCRU	OUCRU	Guy Thwaites; Huynh Trung Trieu; Lam Minh Yen; Le Manh Hung; Le Nguyen Truc Nhu; Le Thi Thu Huong; Le Van Tan; Nghiem My Ngoc; Ngo Ngoc Quang Minh; Nguyen Thanh Dung; Nguyen Thanh Phong; Nguyen Thanh Truong; Nguyen Thi Han Ny; Nguyen Thi Thu Hong; Nguyen Thi thu Hong; Nguyen To Anh; Nguyen Tri Dung; Nguyen Van Vinh Chau; Tran Nguyen Hoang Tu; Tran Tan Thanh
EPI_ISL_2406461, EPI_ISL_2501032	Oddar Meanchey Rapid Response Team	Virology Unit, Institut Pasteur du Cambodge	Cecile Troupin; Chau Darapheak; Chin Savuth; Erik A Karlsson; Jurre Y Siegers; Kraing Sidonn; Leakhena Pum; Ly Sovann; Veasna Duong; Yi Sengdoeurn
EPI_ISL_1138869	Office of Diseases Prevention and Control Region 4 Saraburi	COVID-19 Network Investigations (CONI) Alliance	Angkana Huang; Anthony R. Jones; Arporn Wangwiwatsin; Bhakbhoom Panthan; Chonticha Klungtong; Duangkamon Loesbanluechai; Ekawat Pasomsub; Elizabeth Batty; Insee Sensorn; Janjira Thaipadungpanit; Jutikul Kaewmalakul; Khajohn Joonlasak; Kingkan Rakmanee; Krittikorn Kumpornsin; Namfon Kotanan; Nathamon Runnachot; Pakjira Rimdusit; Payon Pengyo; Praima Moonmuang; Sataporn Hatsadichart; Sirinapa Singthong; Siriporn Lakesukthom; Siriwan Yaemnimnual; Stefan Fernandez; Suttiruk Changchawai; Thanat Chookajorn; Theerarat Kochakarn; Treewat Watthanachockchai; Wasun Chantratita; Wonvimol Lemprasert; Wudtichai Manasatienkij
EPI_ISL_2588639	Ohio Department of Health Laboratory	Ohio Department of Health Laboratory	Allison Black; Brent Lee; Caitlin McDonnell; Eric Brandt; Erica Leasure; Glen McGillivary; Heather Blankenship; Holmes; Jade Mowery; Jennifer; Kelsey Florek; Keoni Omura; Kirtana Ramadugu; Quanta Brown; Stephanie Mccracken; Tyler Payne; and Tammy Bannerman
EPI_ISL_2547342, EPI_ISL_2547351, EPI_ISL_2547353	Okiep CHC	National Health Laboratory Service/University of Cape Town (NHLS/UCT)	Arash Iranzadeh; Bruna Galvao; Carolyn Williamson; Deelan Doolabh; Diana Hardie; Gert Marais; Innocent Mudau; Lynn Tyers; Marvin Hsiao; Stephen Korsman
EPI_ISL_492016	Oman-NIC	Department of Microbiology and Immunology-SQUH	Abdulla Balkhair; Ahlam Al-Amri; Aisha Al-Amri; Aisha Al-Busaidi; Amina Al Jardani; Fahad Zadjali; Fatma BaAlawi; Hamida AL Barwani; Hanan Al-kindi; Intisar Al-Shukri; Khulood Al-Mammary; Mohammed Al-Tobi; Samiha Al Kharusi; Samira Al-Maruqi; Zeyana AL-Dahmani
EPI_ISL_457701, EPI_ISL_457706	Oman-NIC	Oman-NIC	Abdulla Balkhair; Ahlam Al-Amri; Aisha Al-Amri; Aisha Al-Busaidi; Amina Al Jardani; Fahad Zadjali; Fatma BaAlawi; Hamida AL Barwani; Hanan Al-kindi; Intisar Al-Shukri; Khulood Al-Mammary; Mohammed Al-Tobi; Samiha Al Kharusi; Samira Al-Maruqi; Zeyana AL-Dahmani
EPI_ISL_2921171, EPI_ISL_2921174, EPI_ISL_2921181, EPI_ISL_2921182, EPI_ISL_2921186, EPI_ISL_2921187, EPI_ISL_2921188, EPI_ISL_2921200, EPI_ISL_2921201, EPI_ISL_2925607, EPI_ISL_2925610, EPI_ISL_2925612, EPI_ISL_2925622, EPI_ISL_2925623			
see above	Oman-NIC	Oman-National Influenza Center	Aisha Al-Busaidi; Amina Al Jardani; Bilal Hussain.; Fahad Al Balushi; Hanan Al Kindi; Intisar Al-Shukri; Intisar Al-Shukri.; Laila Al Balushi; Samiha Al Kharusi
EPI_ISL_1795050	Oman-NIC	Oman-National Influenza Center-Department of Microbiology and Immunology-SQUH	Abdulla Balkhair; Ahlam Al-Amri; Aisha Al-Amri; Aisha Al-Busaidi; Amina Al Jardani; Fahad Zadjali; Fatma BaAlawi; Hamida AL Barwani; Hanan Al-kindi; Intisar Al-Shukri; Khulood Al-Mammary; Mohammed Al-Tobi; Samiha Al Kharusi; Samira Al-Maruqi; Zeyana AL-Dahmani
EPI_ISL_1819076	Oman-NIC	Oman-National Influenza Center-Department of Microbiology and Immunology-SQUH	Abdulla Balkhair; Ahlam Al-Amri; Aisha Al-Amri; Aisha Al-Busaidi; Amina Al Jardani; Fahad Zadjali; Fatma BaAlawi; Hamida AL Barwani; Hanan Al-kindi; Intisar Al-Shukri; Khulood Al-Mammary; Mohammed Al-Tobi; Samiha Al Kharusi; Samira Al-Maruqi; Zeyana AL-Dahmani
EPI_ISL_525429, EPI_ISL_1532282, EPI_ISL_2692996	Oman-National Influenza Center	Biotechnology & OMICs Laboratory	; Abdul Latif Khan; Adil Al-Wahaibi; Adil Khan; Ahmed Al Harrasi; Ahmed Al-Harrasi; Ahmed Al-Rawahi; Ahmed N Al-Rawahi; Aisha Al-Amri; Amal Al-Maani; Amina Al-Jardani; Amina Al-Jardani.; Bilal Hussain; Hanan Al-Kindi; Intisar Al-Shukri; Sajjad Asaf; Samiha Al-Kharusi; Samira Al-Mahruqi; Samiya Al-Zadjali; Saqib Bilal; Seif Al-Abri
EPI_ISL_518845, EPI_ISL_518848	Oman-National Influenza Center	Biotechnology & OMICs Laboratory, Natural & Medical Sciences Research Center, University of Nizwa	Abdul Latif Khan; Adil Al-Wahaibi; Adil Khan; Ahlam Al-Amri; Ahmed Al-Harrasi; Ahmed Al-Rawahi; Aisha Al-Amri; Aisha Al-Busaidi; Amina Al-Jardani; Hanan Al-Kindi; Intisar Al-Shukri; Sajjad Asaf; Samiha Al-Kharusi; Samira Al-Mahruqi; Seif Al-Abri; Seif Al-Abri.
EPI_ISL_2820625	Omega Diagnostics at Mounes	Omega Diagnostics at Mounes	Cynthia Corley; Latira Haynes-Jacob; Maria Echeverria; Vivek Khare MD
EPI_ISL_2405157	Omics Sciences Laboratory	Omics Sciences Laboratory	Darlyn Amaya; Derly Andrade Molina; Gabriel Morey León; Juan Carlos Fernández Cadena; Rubén Armas González
EPI_ISL_569843	Omsk Research Institute of Natural Focal Infections	WHO National Influenza Centre Russian Federation	Aleksei Vasilenko; Andrey Komissarov; Artem Fadeev; Daria Nashatyreva; Ekaterina Gradoboeva; Ekaterina Savkina; Elena Poleshchuk; Valery Yakimenko
EPI_ISL_1739670, EPI_ISL_1739728, EPI_ISL_1739844, EPI_ISL_1739932, EPI_ISL_1739979, EPI_ISL_1740041, EPI_ISL_1740066, EPI_ISL_1740069, EPI_ISL_1740095, EPI_ISL_1740341, EPI_ISL_1740361, EPI_ISL_1740375, EPI_ISL_1740387, EPI_ISL_1740392, EPI_ISL_1740396, EPI_ISL_1740428, EPI_ISL_1740446			
see above	Ontario's COVID-19 Genomics Rapid Response Coalition	McMaster University	Ahmed Draia; Allison McGeer; Andrew G. McArthur; Angel Li; Emily Panousis; Hooman Derakhshani; Jalees Nasir; Kuganya Nirmalarajah; Michael Surette; Patryk Aftanas; Samira Mubareka
EPI_ISL_1546496, EPI_ISL_1546997, EPI_ISL_1979289	Originating lab: Wales Specialist Virology Centre Sequencing lab: Pathogen Genomics Unit	Public Health Wales Microbiology Cardiff Wales Specialist Virology Centre	Alec Birchley; Alexander Adams; Amy Gaskin; Angela Marchbank; Bree Gatica-Wilcox; Catherine Moore; Jason Coombes; Joanne Watkins; Joel Southgate; Johnathan Evans; Laura Gifford; Lauren Gilbert; Lee Graham; Malorie Perry; Matthew Bull; Nicole Pacchiarini; Sally Corden; Sara Kumziene-Summerhayes; Sara Rey; Sarah Taylor; Simon Cottrell; Sophie Jones; Tom Connor
EPI_ISL_417484, EPI_ISL_2491426	Oslo University Hospital, Department of Medical Microbiology	Norwegian Institute of Public Health, Department of Virology	Atiya R Ali; Debech Nadia; Engebretsen Serina Beate; Garcia Llorente Ignacio; Hilde Elshaug; Hilde Vollan; Jon Bråte; Kamilla Heddeland Instefjord; Karoline Bragstad; Kathrine Stene-Johansen; Line Victoria Moen; Marie Paulsen Madsen; Olav Hungnes; Pedersen Benedikte Nevjen; Rasmus Riis Kopperud
EPI_ISL_1820369, EPI_ISL_1820375, EPI_ISL_2225889, EPI_ISL_2426075, EPI_ISL_2491425, EPI_ISL_2925773	Ostfold Hospital Trust - Kalnes, Centre for Laboratory Medicine, Section for gene technology and infection serology	Norwegian Institute of Public Health, Department of Virology	Atiya R Ali; Debech Nadia; Engebretsen Serina Beate; Garcia Llorente Ignacio; Hilde Elshaug; Hilde Vollan; Jon Bråte; Kamilla Heddeland Instefjord; Karoline Bragstad; Kathrine Stene-Johansen; Line Victoria Moen; Marie Paulsen Madsen; Olav Hungnes; Pedersen Benedikte Nevjen; Rasmus Riis Kopperud
EPI_ISL_3031381, EPI_ISL_3031391, EPI_ISL_3031396, EPI_ISL_3031399	Othaya Sub-County Hospital	USAMRD-A, Basic Science Laboratory	Alan Lemtudo; Beth Mutai; Brian Andika; Carol Kifude; Clement Masakwe; Eric Muthanje; Esther Omuseni; Faith Sigei; Gathii Kimita; George Awinda; John Waitumbi; Josphat Nyataya; Rachel Githii; Rehema Liyai; Stephen Ochola
EPI_ISL_1534378	Oudtshoorn Hospital wc OUD	NHLS/UCT	Arash Iranzadeh; Bruna Galvao; Carolyn Williamson; Deelan Doolabh; Diana Hardie; Emmanuel SJ; Innocent Mudau; Kruger Marais; Lynn Tyers; Marvin Hsiao; Stephen Korsman; Tegally H; de Oliveira T
EPI_ISL_1273214	Oxford University Clinical Research Unit (OUCRU)	Oxford University Clinical Research Unit (OUCRU)	Guy Thwaites; Huynh Trung Trieu; Lam Minh Yen; Le Manh Hung; Le Nguyen Truc Nhu; Le Van Tan; Nghiem My Ngoc; Ngo Ngoc Quang Minh; Nguyen Thanh Dung; Nguyen Thanh Phong; Nguyen Thanh Truong; Nguyen Thi Thu Hong; Nguyen To Anh; Nguyen Tri Dung; Nguyen Van Vinh Chau; Tran Nguyen Hoang Tu; Tran Tan Thanh; le Thi Thu Huong
EPI_ISL_3078887	Oxford Viromics, NDM, University of Oxford; Oxford University Hospitals; Basingstoke and North Hampshire Hospital	COVID-19 Genomics UK (COG-UK) Consortium	Alex Mobbs; Amy Trebes; Anita Justice; Catrin Moore; Christophe Fraser; David Bonsall; David Buck; Emma Wise; George Macintyre; Jessica Lynch; John Todd; Mariateresa de Cesare; Matilde Mori; Monique Andersson; Nathan Moore; Nick Cortes; Robert Shaw; Stephen Kidd; Tanya Golubchik; Timothy Peto
EPI_ISL_2086976	PATH CARE	National Institute for Communicable Diseases of the National	Amoako DG; Bhiman JN; Ismail A; Mahlangu B; Mohale T; Ntuli N; Scheepers C

		Health Laboratory Service	
EPI_ISL_2695543, EPI_ISL_2709853, EPI_ISL_2709854, EPI_ISL_2709856	POLOKWANE MANKWENG HOSPITAL	National Institute for Communicable Diseases of the National Health Laboratory Service	Amoako DG; Bhiman JN; Everatt J; Ismail A; Mahlangu B; Mnguni A; Mohale T; Ntuli N; Scheepers C
EPI_ISL_2984863	PORT ELIZABETH LABOARTORY	National Institute for Communicable Diseases of the National Health Laboratory Service	Amoako DG; Bhiman JN; Everatt J; Ismail A; Mahlangu B; Mnguni A; Mohale T; Ntuli N; Scheepers C
EPI_ISL_2827902, EPI_ISL_2827903, EPI_ISL_2895717, EPI_ISL_2895720, EPI_ISL_2895726, EPI_ISL_2895727, EPI_ISL_2895735, EPI_ISL_2895741, EPI_ISL_2895744, EPI_ISL_2895748			
see above	PORT ELIZABETH LABORATORY	National Institute for Communicable Diseases of the National Health Laboratory Service	Amoako DG; Bhiman JN; Everatt J; Ismail A; Mahlangu B; Mnguni A; Mohale T; Ntuli N; Scheepers C
EPI_ISL_2984922, EPI_ISL_2984939	POTCHEFSTROOM	National Institute for Communicable Diseases of the National Health Laboratory Service	Amoako DG; Bhiman JN; Everatt J; Ismail A; Mahlangu B; Mnguni A; Mohale T; Ntuli N; Scheepers C
EPI_ISL_2384025	PR Public Health Lab	Centers for Disease Control and Prevention Division of Viral Diseases, Pathogen Discovery	Alison Laufer Halpin; Ben L. Rambo-Martin; Clinton R. Paden; Dakota Howard; Darlene Wagner; Dave Wentworth; Dhwani Batra; Jasmine Padilla; Justin Lee; Katie Dillon; Krista Queen; Kristen Knipe; Kristine Lacek; Mark Burroughs; Matthew Schmerer; Mili Sheth; Peter Cook; Sam Shepard; Sarah Nobles; Shoshona Le; Suxiang Tong; Vivien Dugan; Yvette Unoarumhi
EPI_ISL_2493252	PRONTO ATENDIMENTO DE VARGEM GRANDE PAULISTA	Instituto Butantan	Antonio Jorge Martins; Claudia Renata dos Santos Barros; David Schlesinger; Debora Botequio Moretti; Dimas Tadeu Covas; Elaine Cristina Marqueze; Elaine Vieira Santos; Evandra Strazza Rodrigues; Heidge Fukumasu; Jayme Augusto de Souza-Neto; José Salvatore Leister Patané; Luiz Alcantara; Luiz Lehmann Coutinho; Maria Carolina Elias; Maurício Lacerda Nogueira; Rafael dos Santos Bezerra; Raul Machado Neto; Rejane Maria Tommasini Grotto; Ricardo Haddad; Sandra Coccuzzo Sampaio Vessoni; Simone Kashima; Svetoslav Nanev Slavov; Vincent Louis Viala
EPI_ISL_2779535	PSTC	USAMRD-A, Basic Science Laboratory	Alan Lemtudo; Beth Mutai; Brian Andika; Carol Kifude; Clement Masakwe; Eric Muthanje; Esther Omuseni; Faith Sigei; Gathii Kimita; George Awinda; John Waitumbi; Josphat Nyataya; Rachel Githii; Rehema Liyai; Stephen Ochola
EPI_ISL_2876986	Pacaltsdorp Clinic wc PAC	NHLS/UCT	Arash Iranzadeh; Bruna Galvao; Carolyn Williamson; Deelan Doolabh; Diana Hardie; Gert Marais; Innocent Mudau; Lynn Tyers; Marvin Hsiao; Stephen Korsman
EPI_ISL_2241616, EPI_ISL_2241618, EPI_ISL_2241619, EPI_ISL_2504093, EPI_ISL_2504094, EPI_ISL_2504099, EPI_ISL_2504100, EPI_ISL_2504101, EPI_ISL_2504102, EPI_ISL_2504103, EPI_ISL_2504104, EPI_ISL_2504105, EPI_ISL_2504106, EPI_ISL_2504107, EPI_ISL_2504108, EPI_ISL_2504109, EPI_ISL_2504110, EPI_ISL_2504112, EPI_ISL_2504114, EPI_ISL_2504115, EPI_ISL_2504116, EPI_ISL_2504117, EPI_ISL_2509333, EPI_ISL_2509334			
see above	Palapye Primary Hospital Laboratory	Botswana Harvard HIV Reference Laboratory	Boitumelo Zuze; Botshelo Radibe; David Lawrence; Dorcas Maruapula; Joseph Makhema; Keoratile Ntshambiwa; Kwana Lechiile; Legodile Kooepile; Letsibogo Gaoraelwe; Madisa Mine; Modisa Motswaledi; Mosepele Mosepele; Ontlametse T. Bareng; Roger Shapiro; Shahin Lockman; Sikhulile Moyo; Sikhulile Wonderful T. Choga; Simani Gaseitsiwe; Thela Tefelo; Thongbotho Mphoyakgosi; Wonderful T. Choga
EPI_ISL_596509, EPI_ISL_596529, EPI_ISL_596530, EPI_ISL_596532, EPI_ISL_596544, EPI_ISL_596561	Palestinian Ministry of Health	Molecular Genetics Lab	Damien Richard; Dana Najjar; Francois Balloux; Hisham Darwish; Husam Sallam; Issa Shtayeh; Lucy van Dorp; Mahmoud Ruzayqat; Nouar Qutob; Osama Najjar; Zaidoun Salah
EPI_ISL_1098330, EPI_ISL_1173095, EPI_ISL_1543418, EPI_ISL_1543814, EPI_ISL_2633269, EPI_ISL_2634471, EPI_ISL_2634792, EPI_ISL_2716022, EPI_ISL_2923861			
see above	Pandemic Response Lab - NYC	Pandemic Response Lab, R&D	Cybill del Castillo; Dylan Law; Haiping Hao; Henry Lee; Isabel Fernandez Escapa; Jon Laurent; Katharine Nelson; Melissa Hopkins; Michael Hammerling; Pradeep Bugga; Shinyoung Clair Kang; Sol Rey; William Ward
EPI_ISL_2876996	Parkwood Clinic wc PRK	NHLS/UCT	Arash Iranzadeh; Bruna Galvao; Carolyn Williamson; Deelan Doolabh; Diana Hardie; Gert Marais; Innocent Mudau; Lynn Tyers; Marvin Hsiao; Stephen Korsman
EPI_ISL_2171131	Pasig City Children's Hospital - Child's Hope	Philippine Genome Center	Alethea R. de Guzman; Anna Ong-Lim; Arianne A. Zamora; Asia Louisa U. Chong; Benedict A. Maralit; Candice Francheska B. Tambaoan; Carlo M. Lapid; Celia Carlos; Devon Ray Pacial; Edsel Maurice Salvaña; El King D. Morado; Elcid Aaron R. Pangilinan; Eva Maria Cutiongco-de la Paz; Francis A. Tablizo; Irish Coleen A. Asin; Jaime C. Montoya; Jan Michael C. Yap; Jo-Hannah S. Llames; John Q. Wong; Joshua Gregor A. Dizon; Juan Antonio R. Magalang; Karol Sophia Agape R. Padilla; Kenneth M. Kim; Kris P. Punayan; Marc Edsel C. Ayes; Marc Jerrone R. Castro; Maria Rosario Singh-Vergeire and Cynthia P. Saloma; Maria Sofia L. Yangzon; Marissa Alejandria; Razel Nikka M. Hao; Rianna Patricia S. Cruz; Sheila Mae M. Araiza
EPI_ISL_2035941, EPI_ISL_2035946, EPI_ISL_2035947, EPI_ISL_2035949, EPI_ISL_2035988, EPI_ISL_2036077	Pasteur Institute - Laboratory of Clinical Virology	Pasteur Institute - Laboratory of Clinical Virology	Anissa Chouikha; Henda Triki; Kais Ghedira; Mariem Gdoura; Sondos Haddad; Wasfi Fares
EPI_ISL_2694596, EPI_ISL_2695826	PathCare, Cape Town	Division of Medical Virology, National Health Laboratory Service (NHLS), Tygerberg Hospital / Stellenbosch University	Bronwyn Kleinhans; Gert van Zyl; Houriiyah Tegally; Jean Maritz; Petra Raimond; San Emmanual James; San Emmanuel James; Susan Engelbrecht; Tania Stander; Tongai Maponga; Tulio de Oliveira; Wolfgang Preiser
EPI_ISL_456163, EPI_ISL_456215, EPI_ISL_456301, EPI_ISL_622806, EPI_ISL_755626	PathLab Bay of Plenty	Institute of Environmental Science and Research (ESR)	Anja Werno; Antje van der Linden; Arlo Upton; Chris Mansell; David Hammer; Dragana Drinkovic; Erasmus Smit; Gary McAuliffe; Hana Sofia Andersson; Hermes Perez; James Ussher; Jill Sherwood; Jing Wang; Joep de Ligt; Josh Freeman; Julia Howard; Juliet Elvy; Lauren Jelly; Mary DeAlmeida; Matt Blakiston; Matt Storey; Matthew Rogers; Max Bloomfield; Michael Addidle; Michelle Balm; Muhammad Faisal; Nikki Freed; Olin Silander; Sally Roberts; Sarah Jefferies; Sharmini Muttaiyah; Susan Morpeth; Susan Taylor; Timothy Blackmore; Vani Sathyendran; Veronica Playle; Virginia Hope; Xiaoyun Ren
EPI_ISL_420456, EPI_ISL_470851, EPI_ISL_2713075, EPI_ISL_2758809, EPI_ISL_2932263	PathWest Laboratory Medicine WA	PathWest Laboratory Medicine WA	Avram Levy; Chisha Sikazwe; David Smith and David Speers; Jurissa Lang; PathWest Laboratory Medicine WA Microbial Surveillance Unit
EPI_ISL_596691, EPI_ISL_596741, EPI_ISL_596743, EPI_ISL_596754, EPI_ISL_596770, EPI_ISL_596777, EPI_ISL_596837, EPI_ISL_605854, EPI_ISL_672628, EPI_ISL_672640, EPI_ISL_672646, EPI_ISL_708758, EPI_ISL_708794, EPI_ISL_794672, EPI_ISL_794686, EPI_ISL_794710, EPI_ISL_810967, EPI_ISL_1017678, EPI_ISL_1069391, EPI_ISL_1069392, EPI_ISL_1069393, EPI_ISL_1198802, EPI_ISL_1295933, EPI_ISL_1448427, EPI_ISL_1508992, EPI_ISL_1508993, EPI_ISL_1508994, EPI_ISL_1633676, EPI_ISL_1633681, EPI_ISL_1669117, EPI_ISL_1669120, EPI_ISL_1669123, EPI_ISL_1669125, EPI_ISL_1750965, EPI_ISL_1816917, EPI_ISL_1817558, EPI_ISL_1914666, EPI_ISL_1914667, EPI_ISL_1914668, EPI_ISL_1972908, EPI_ISL_1972909, EPI_ISL_1972911, EPI_ISL_1972912, EPI_ISL_2226229, EPI_ISL_2304306, EPI_ISL_2322898, EPI_ISL_2598297, EPI_ISL_2757655, EPI_ISL_2757656, EPI_ISL_2757657, EPI_ISL_2784344, EPI_ISL_2841828, EPI_ISL_2841829, EPI_ISL_2841830, EPI_ISL_2841831, EPI_ISL_2861270, EPI_ISL_2942965, EPI_ISL_2982303, EPI_ISL_3038899			
see above	PathWest Laboratory Medicine WA	PathWest Laboratory Medicine WA Microbial Surveillance Unit	PathWest Laboratory Medicine WA Microbial Surveillance Unit
EPI_ISL_2691707	PathWest Laboratory Medicine WA Microbial Surveillance Unit	PathWest Laboratory Medicine WA Microbial Surveillance Unit	Hospital Ave; Nedlands WA 6009; QEII Medical Centre
EPI_ISL_591403, EPI_ISL_591414, EPI_ISL_591426, EPI_ISL_667570, EPI_ISL_667586, EPI_ISL_667621, EPI_ISL_721585, EPI_ISL_779210, EPI_ISL_779237, EPI_ISL_803936, EPI_ISL_825399, EPI_ISL_833371			
see above	Pathogen Genomics Center, National Institute of Infectious Diseases	Pathogen Genomics Center, National Institute of Infectious Diseases	Kentaro Itokawa; Makoto Kuroda; Masanori Hashino; Rina Tanaka; Tsuyoshi Sekizuka
EPI_ISL_437463, EPI_ISL_437467, EPI_ISL_512962, EPI_ISL_513023, EPI_ISL_513152, EPI_ISL_678037, EPI_ISL_678151, EPI_ISL_678165			
see above	Pathogen Genomics Lab King Abdullah University of Science and Technology(KAUST)	Pathogen Genomics Lab King Abdullah University of Science and Technology(KAUST)	Abbas Al Mutair; Abdulaziz Alahmadi; Afrah Alsomali; Amanda; Amanda Ooi; Amit Kumar Subudhi; Anwar Hashem; Arnab Pain; Asim Khogeer; Awad Al-Omari; Fadwa Alofi; Fathia Ben Rached; Jumana Taha; Kahled Algithami; Luke; Luke Esau; Naif Almontashiri; Nashwa Al-khotani; Olga Douvropoulou; Raeece Naeem; Rahul P Salunke; Raushan Nugmanova; Samer Salih; Sara Mfarrej; Sharif Hala
EPI_ISL_593656	Pathology North - Hunter - NSW Health Pathology	NSW Health Pathology - Institute of Clinical Pathology and Medical Research; Westmead Hospital; University of Sydney	CIDM-PH et al.
EPI_ISL_407894, EPI_ISL_407896, EPI_ISL_410717	Pathology Queensland	Public Health Virology Laboratory	Alyssa Pyke; Amanda De Jong; Andrew Van Den Hurk; Ben Huang; Carmel Taylor; David Warrilow; Doris Genge; Elisabeth Gamez; Glen Hewitson; Ian Maxwell Mackay; Inga Sultana; Jamie McMahon; Jean Barcelon; Judy Northill; Mitchell Finger; Natalie Simpson; Neelima Nair; Peter Burtonclay; Peter Moore; Sarah Wheatley; Sean Moody; Sonja Hall-Mendelin; Timothy Gardam; and Frederick Moore.
EPI_ISL_490036	Pathology West - NSW Health Pathology	NSW Health Pathology - Institute of Clinical Pathology and Medical Research; Westmead Hospital; University of Sydney	CIDM-PH et al.
EPI_ISL_2876956	Pelican Park CDC wc PAX	NHLS/UCT	Arash Iranzadeh; Bruna Galvao; Carolyn Williamson; Deelan Doolabh; Diana Hardie; Gert Marais; Innocent Mudau; Lynn Tyers; Marvin Hsiao; Stephen Korsman
EPI_ISL_2859337	Philippine General Hospital (PGH)	Philippine Genome Center	Alethea R. de Guzman; Anna Ong-Lim; Arianne A. Zamora; Benedict A. Maralit; Carlo M. Lapid; Celia Carlos; Devon Ray Pacial; Diomedes A. Carino; Edsel Maurice Salvaña; El King D. Morado; Elcid Aaron R. Pangilinan; Eva Maria Cutiongco-de la Paz; Francis A. Tablizo; Henrietta Marie Rodriguez; Jaime C. Montoya; Jan Michael C. Yap; Jarvin E. Nipales; Jo-Hannah S. Llames; John Q. Wong; Joshua Gregor A. Dizon; Juan Antonio R. Magalang; Karol Sophia Agape R. Padilla; Kenneth M. Kim; Kris P. Punayan; Krisitna Patriz Dela Cruz; Lindsay Claire D.L. Carandang; Ma. Exanil Plantig; Marc Edsel C. Ayes; Maria Rosario Singh-Vergeire and Cynthia P. Saloma; Maria Sofia L. Yangzon; Marielle M Gamboa; Marissa Alejandria; Nina Francesca Bustamante; Razel Nikka M. Hao; Renato Jacinto Q. Mantaring; Rianna Patricia S. Cruz; Sheila Mae M. Araiza; Yvonne Valerie Austria; Zipporah Mariebelle R. Enriquez; Zyrel V. Mollejon
EPI_ISL_2155237, EPI_ISL_2859654	Philippine Red Cross - Port Area	Philippine Genome Center	Alethea R. de Guzman; Anna Ong-Lim; Arianne A. Zamora; Asia Louisa U. Chong; Benedict A. Maralit; Candice Francheska B. Tambaoan; Carlo M. Lapid; Celia Carlos; Devon Ray Pacial; Diomedes A. Carino; Edsel Maurice Salvaña; El King D. Morado; Elcid Aaron R. Pangilinan; Eva Maria Cutiongco-de la Paz; Francis A. Tablizo; Henrietta Marie Rodriguez; Irish Coleen A. Asin; Jaime C. Montoya; Jan Michael C. Yap; Jarvin E. Nipales; Jo-Hannah S. Llames; John Q. Wong; Joshua Gregor A. Dizon; Juan Antonio R. Magalang; Karol Sophia Agape R. Padilla; Kenneth M. Kim; Kris P. Punayan; Krisitna Patriz Dela Cruz; Lindsay Claire D.L. Carandang; Ma. Exanil Plantig; Marc Edsel C. Ayes; Marc Jerrone R. Castro; Maria Rosario Singh-Vergeire and Cynthia P. Saloma; Maria Sofia L. Yangzon; Marielle M Gamboa; Marissa Alejandria; Nina Francesca Bustamante; Razel Nikka M. Hao; Renato Jacinto Q. Mantaring; Rianna Patricia S. Cruz; Sheila Mae M. Araiza; Yvonne Valerie Austria; Zipporah Mariebelle R. Enriquez; Zyrel V. Mollejon
EPI_ISL_1164597	Plateforme de testing Namuroise	Plateforme de testing Namuroise	Degosserie Jonathan; Denis Olivier; Mullier François; Otto Gaetan
EPI_ISL_2483324, EPI_ISL_2714810, EPI_ISL_2988339	Platform BIS UZA/UAntwerpen	Labo Klinische Biologie, UZA	Basil Britto Xavier; Christine Lammens; Herman Goossens; Ines Verbesselt; Jasmine Coppens; Kathleen Holemans; Marie Le Mercier; Veerle Matheeussen
EPI_ISL_1120735	Platform BIS UZA/UAntwerpen	UAntwerp, Laboratory of Medical Microbiology	Basil Britto Xavier; Christine Lammens; Herman Goossens; Jasmine Coppens; Marie Le Mercier; Veerle Matheeussen
EPI_ISL_2550759	Port Dickson Hospital	Institute for Medical Research, Infectious Disease Research Centre, National Institutes of Health, Ministry of Health Malaysia	Azizan MA; Kamel K; Mohd Zawawi Z; Ramly N; Robert F; Suppiah J; Thayan R
EPI_ISL_2447801, EPI_ISL_2447809	Port Elizabeth Laboratory	National Institute for Communicable Diseases of the National Health Laboratory Service	Amoako DG; Bhiman JN; Ismail A; Mahlangu B; Mohale T; Ntuli N; Scheepers C
EPI_ISL_712075	Port Elizabeth Provincial Hospital, National Health Laboratory Services, Eastern Cape, South Africa	National Institute for Communicable Diseases of the National Health Laboratory Service	Allam M; Bhiman JN; Ismail A; Mahlangu B; Mohale T; Ntuli N
EPI_ISL_2779427, EPI_ISL_2779428, EPI_ISL_3031388, EPI_ISL_3031389, EPI_ISL_3031390, EPI_ISL_3031419, EPI_ISL_3031420			

see above	Port Health Clinic, Busia border point	USAMRD-A, Basic Science Laboratory	Alan Lemtudo; Beth Mutai; Brian Andika; Carol Kifude; Clement Masakwe; Eric Muthanje; Esther Omuseni; Faith Sigei; Gathii Kimita; George Awinda; John Waitumbi; Josphat Nyataya; Rachel Githii; Rehema Liyai; Stephen Ochola
EPI_ISL_2779496, EPI_ISL_2779500, EPI_ISL_2779507	Port Health Clinic, Malaba border point	USAMRD-A, Basic Science Laboratory	Alan Lemtudo; Beth Mutai; Brian Andika; Carol Kifude; Clement Masakwe; Eric Muthanje; Esther Omuseni; Faith Sigei; Gathii Kimita; George Awinda; John Waitumbi; Josphat Nyataya; Rachel Githii; Rehema Liyai; Stephen Ochola
EPI_ISL_529013	Presidio Ospedaliero "S.Filippo e Nicola"-Avezzano	Istituto Zooprofilattico Sperimentale dell'Abruzzo e Molise "G.Caporale"	Ancora M; Cammà C; Curini V; Di Domenico M; Di Pasquale A; Lorusso A; Mangone I; Marcacci M; Puglia I; Rinaldi A; Savini G.
EPI_ISL_1819200	Presidio di Brindisi Di Summa - Perrino	Istituto Zooprofilattico Sperimentale della Puglia e della Basilicata	Bianco A.; Capozzi L.; Del Sambro L.; Difato L.; Giannico A.; Parisi A.; Ridolfi D.; Santoro A.; Simone D.
EPI_ISL_2790410	Primary Health Care Celinac	Public Health Institute of Republic of Srpska	Branka Culibrk; Dijana Vukajlovic; Milica Celic; Pava Dimitrijevic; Stanka Tomic; Tatjana Markovic; Zeljka Sumic
EPI_ISL_2790402, EPI_ISL_2790404, EPI_ISL_2790406	Primary Health Care Laktasi	Public Health Institute of Republic of Srpska	Branka Culibrk; Dijana Vukajlovic; Milica Celic; Pava Dimitrijevic; Stanka Tomic; Tatjana Markovic; Zeljka Sumic
EPI_ISL_2790405	Primary Health Care Prijedor	Public Health Institute of Republic of Srpska	Branka Culibrk; Dijana Vukajlovic; Milica Celic; Pava Dimitrijevic; Stanka Tomic; Tatjana Markovic; Zeljka Sumic
EPI_ISL_417064	Prince of Wales Hospital	Hong Kong Department of Health	Alan K.L. Tsang; Dominic N.C. Tsang; Edman T.K. Lam; Peter C.W. Yip; Rickjason C.W. Chan
EPI_ISL_1402425, EPI_ISL_1402426	Private clinic of Biogen Med, Tashkent, Uzbekistan	Center of Genomics and bioinformatics, Bioinformatics laboratory	Abdurakhmon N Yusupov; Ibrokhim Y Abdurakhmonov.; Mirzakamol S Ayubov; Mukhammadjon H Mirzakhmedov; Shukhrat E Shermatov; Zabardast T Buriev
EPI_ISL_1092071	Protzer Lab	Protzer Lab, Laboratory for Functional Genome Analysis, Dept. Genomics, Gene Center of the LMU Munich	Alexander Graf; Dieter Hoffmann; Elisabeth Esser; Eva C.Schulte; Helmut Blum; Stefan Krebs; Till Bunse; Ulrike Protzer
EPI_ISL_1909922	Provincial Public Health Reference Laboratory	Provincial Public Health Reference Laboratory	ANDLEEB HANIF; Asim Altaf; Hassan Nawaz; Mariam Javed
EPI_ISL_2543563, EPI_ISL_2543615, EPI_ISL_2544508	Provincial Public Health Reference Laboratory (PPHRL) P&SHD	Provincial Public Health Reference Laboratory (PPHRL) P&SHD	ANDLEEB HANIF
EPI_ISL_1180679	Public Health Authority of the Slovak Republic	Bergthaler laboratory, CeMM Research Center for Molecular Medicine of the Austrian Academy of Sciences	Andreas Bergthaler; Anna Schedl; Bekir Erguner; Benedikt Agerer; Christoph Bock; Fabian Amman; Jan Laine; Lukas Endler; Maelle Le Moing; Martin Senekowitsch; Michael Schuster; Thomas Penz
EPI_ISL_1647661, EPI_ISL_1647749, EPI_ISL_1749617, EPI_ISL_1749618, EPI_ISL_2448839, EPI_ISL_2598620, EPI_ISL_2657889, EPI_ISL_2657919, EPI_ISL_2657920, EPI_ISL_2688039			
see above	Public Health Authority of the Slovak Republic	Laboratory of Genomics and Bioinformatics, Comenius University Science Park	Anna Gičová; Diana Rusňáková; Jakub Styk; Jaroslav Budiš; Miroslav Böhmer; Tatiana Sedláčková; Tomáš Szemes
EPI_ISL_2379751	Public Health Authority of the Slovak Republic	Public Health Authority of the Slovak Republic	Anna Gičová; Barbora Kotvasová; Elena Tichá; Lucia Ševčíková; Miroslav Böhmer; Pavol Mišenko; Terézia Vrabľová; Tomáš Szemes
EPI_ISL_2351129	Public Health Center 52 Samsennok	Division of Genomic Medicine and Innovation support,Department of Medical Sciences, Ministry of Public Health, Thailand	Archawin Rojanawiwat; Jirapha Pakdee; Natthakul Bunneang; Nuanjun Wichukchinda; Penpitcha Thawong; Pilailuk Akkapaiboon Okada; Pundharika Piboonsiri; Surakameth Mahasirimongkol; Waritta Sawaengdee
EPI_ISL_1112327, EPI_ISL_1112329, EPI_ISL_1112753	Public Health Center of Ukraine	Charité Universitätsmedizin Berlin, Institute of Virology	Barbara Mühlemann; Christian Drosten; Ihor Kuzin; Iryna Demchyshyna; Julia Schneider; Jörn Beheim-Schwarzbach; Liudmyla Chernenko; Roman Rodyna; Talitha Veith; Terry Jones; Victor M Corman
EPI_ISL_636973	Public Health Lab	Public Health Lab	Alwasti; H
EPI_ISL_775080, EPI_ISL_775157, EPI_ISL_854650, EPI_ISL_854721, EPI_ISL_875752, EPI_ISL_875764, EPI_ISL_875765, EPI_ISL_875768, EPI_ISL_875771, EPI_ISL_875848, EPI_ISL_875863, EPI_ISL_875864, EPI_ISL_925718, EPI_ISL_925769, EPI_ISL_925781, EPI_ISL_1112618, EPI_ISL_1112619, EPI_ISL_1112631, EPI_ISL_1112803, EPI_ISL_1112836, EPI_ISL_1170498, EPI_ISL_1170552, EPI_ISL_1170615, EPI_ISL_1170627, EPI_ISL_1170659, EPI_ISL_1170706, EPI_ISL_1170769, EPI_ISL_1170787, EPI_ISL_1170801, EPI_ISL_1170838, EPI_ISL_1170842, EPI_ISL_1183470, EPI_ISL_1183583, EPI_ISL_1230694, EPI_ISL_1230736, EPI_ISL_1230755, EPI_ISL_1230800, EPI_ISL_1270899, EPI_ISL_1270994, EPI_ISL_1271039, EPI_ISL_1271347, EPI_ISL_1271359, EPI_ISL_1271393, EPI_ISL_1271396, EPI_ISL_1271455, EPI_ISL_1271458, EPI_ISL_1271532, EPI_ISL_1271545, EPI_ISL_1271550, EPI_ISL_1271649, EPI_ISL_1271754, EPI_ISL_1271774, EPI_ISL_1271827, EPI_ISL_1271914, EPI_ISL_1335164, EPI_ISL_1368590, EPI_ISL_1478357, EPI_ISL_1478379, EPI_ISL_1478747, EPI_ISL_1559221, EPI_ISL_1559226, EPI_ISL_1559230, EPI_ISL_1559294, EPI_ISL_1559308, EPI_ISL_1742848, EPI_ISL_1743549, EPI_ISL_1896723, EPI_ISL_2011880, EPI_ISL_2011881, EPI_ISL_2012922, EPI_ISL_2141305, EPI_ISL_2141308, EPI_ISL_2141368, EPI_ISL_2141369, EPI_ISL_2141377, EPI_ISL_2250400, EPI_ISL_2250575, EPI_ISL_2251679, EPI_ISL_2252430, EPI_ISL_2364408, EPI_ISL_2452463, EPI_ISL_2922328, EPI_ISL_2922543			
see above	Public Health Ontario Laboratory	Public Health Ontario Laboratory	Aimin Li; Alireza Eshaghi; Andre Villegas; Ashleigh Sullivan; Christine Frantz; Dean Maxwell; Esha Joshi; Heather Rilkoff; Jared Simpson; Jennifer L Guthrie; Jonathan B Gubbay; Karthikeyan Sivaraman; Lawrence Heisler; Matthew Watson; Michael CY Li; Michael Laszloffy; Nahuel Fittipaldi; Philip Banh; Richard de Borja; Samir N Patel; Sandeep Nagra; Sandra Zittermann; Sarah Teatero; Vanessa G Allen; Yao Chen; Yogi Sundaravadanam
EPI_ISL_2860636, EPI_ISL_2860638, EPI_ISL_2860640, EPI_ISL_2860642, EPI_ISL_2860643, EPI_ISL_2860644, EPI_ISL_2860645, EPI_ISL_2860646, EPI_ISL_2860647			
see above	Public Health Reference Laboratory	Erasmus Medical Center	Anne van der Linden; Annemiek van der Eijk; Bas Oude Munnink; Corine GeurtsvanKessel; David Nieuwenhuijse; Emmanuelle Munger; Irina Chestakova; Marion Koopmans; Marjan Boter; Omar Elahmer-Abdulla Bashein- Rahma Algheriani-Ahlam Alarif; Reina Sikkema; Richard Molenkamp; on behalf of the Dutch national COVID-19 response team.
EPI_ISL_1910858	Public Health Virology-Forensic and Scientific Services	Public Health Virology-Forensic and Scientific Services	Son Nguyen
EPI_ISL_1495211	Public Health Authority of the Slovak Republic	Bergthaler laboratory, CeMM Research Center for Molecular Medicine of the Austrian Academy of Sciences	Andreas Bergthaler; Anna Schedl; Bekir Erguner; Benedikt Agerer; Christoph Bock; Fabian Amman; Jan Laine; Lukas Endler; Maelle Le Moing; Martin Senekowitsch; Michael Schuster; Petr Triska; Thomas Penz
EPI_ISL_2801898	Pursat Rapid Response Team	Virology Unit, Institut Pasteur du Cambodge	Cecile Troupin; Chau Darapheak; Chin Savuth; Erik A Karlsson; Jurre Y Siegers; Kraing Sidonn; Leakhena Pum; Ly Sovann; Veasna Duong; Yi Sengdoeurn
EPI_ISL_1055477, EPI_ISL_1055478, EPI_ISL_1055481, EPI_ISL_1055483, EPI_ISL_1055485, EPI_ISL_1055486, EPI_ISL_1055487, EPI_ISL_1055488, EPI_ISL_1055489, EPI_ISL_1055490, EPI_ISL_1055505, EPI_ISL_1055506, EPI_ISL_1055513, EPI_ISL_1055535, EPI_ISL_1055602, EPI_ISL_1055688			
see above	QEII Health Sciences Centre	National Microbiology Laboratory (NML)	Anna Majer; Anneliese Landgraff; CanCOGeN's metadata curation team; Dan Gaston; Darian Hole; Elsie Grudeski; Gary Van Domselaar; Grace Seo; Janice Pettipas; Jason LeBlanc; Jennifer Tanner; Kirsten Biggar; Madison Chapel; Morag Graham; Natalie Knox; Nathalie Bastien; Philip Mabon; Public Health Agency of Canada CanCOGeN team; Rhiannon Huzarewich; Russell Mandes; Shari Tyson; Timothy Booth; Todd Hatchette; Yan Li
EPI_ISL_493545, EPI_ISL_493546, EPI_ISL_2519670	Quadram Institute Bioscience	COVID-19 Genomics UK (COG-UK) Consortium	Alexander J Trotter; Alison E. Mather; Alp Aydin; Ana P. Tedim; Anastasia Kolyva; Andrew Bell; Andrew J. Page; Claire Stuart; Dave J. Baker; Gemma L. Kay; John Wain; Justin O'Grady; Leonardo de Oliveira Martins; Lizzie Meadows; Maria Diaz; Mark Webber; Muhammed Yasir; Nabil-Fareed Alikhan; Ngozi Elumogo; Nicholas M. Thomson; Rachael Stanley; Rachel Gilroy; Reenesh Prakash; Samir Dervisevic; Samuel Bloomfield; Steven Rudder; Thanh Le-Viet
EPI_ISL_2860172	Qualimed Hospital Sta. Rosa	Philippine Genome Center	Alethea R. de Guzman; Anna Ong-Lim; Arianne A. Zamora; Benedict A. Maralit; Carlo M. Lapid; Celia Carlos; Devon Ray Pacial; Diomedes A. Carino; Edsel Maurice Salvaña; El King D. Morado; Elcid Aaron R. Pangilinan; Eva Maria Cutiongco-de la Paz; Francis A. Tablizo; Henrietta Marie Rodriguez; Jaime C. Montoya; Jan Michael C. Yap; Jarvin E. Nipales; Jo-Hannah S. Llames; John Q. Wong; Joshua Gregor A. Dizon; Juan Antonio R. Magalang; Karol Sophia Agape R. Padilla; Kenneth M. Kim; Kris P. Punayan; Krisitna Patriz Dela Cruz; Lindsay Claire D.L. Carandang; Ma. Exanil Plantig; Marc Edsel C. Ayes; Maria Rosario Singh-Vergeire and Cynthia P. Saloma; Maria Sofia L. Yangzon; Marielle M Gamboa; Marissa Alejandria; Nina Francesca Bustamante; Razel Nikka M. Hao; Renato Jacinto Q. Mantaring; Rianna Patricia S. Cruz; Sheila Mae M. Araiza; Yvonne Valerie Austria; Zipporah Mariebelle R. Enriquez; Zyrel V. Mollejon
EPI_ISL_820573, EPI_ISL_820578, EPI_ISL_820607	Queens Medical Centre, Clinical Microbiology Department / DeepSeq Nottingham	COVID-19 Genomics UK (COG-UK) Consortium	Christopher Moore; Fei Sang; Gemma Clark; Hannah Howson-Wells; Johnny Debebe; Jonathan Ball; Joseph Chappell; Manjinder Khakh; Matthew Carlisle; Matthew Loose; Michelle M Lister; Nadine Holmes; Patrick McClure; Theocharis Tsoleridis; Vicki M Fleming; Victoria Wright; Wendy Smith
EPI_ISL_593616, EPI_ISL_593617, EPI_ISL_693269, EPI_ISL_693290, EPI_ISL_849686, EPI_ISL_849694, EPI_ISL_849754, EPI_ISL_1465893, EPI_ISL_1516885, EPI_ISL_1516886, EPI_ISL_1516888, EPI_ISL_1563670, EPI_ISL_1563678, EPI_ISL_1633659, EPI_ISL_1754858, EPI_ISL_1910859, EPI_ISL_1938308, EPI_ISL_2137034, EPI_ISL_2137035, EPI_ISL_2137036, EPI_ISL_2274244, EPI_ISL_2274245, EPI_ISL_2274246, EPI_ISL_2274248, EPI_ISL_2283730, EPI_ISL_2385970, EPI_ISL_2385974, EPI_ISL_2507086, EPI_ISL_2507088, EPI_ISL_2507089, EPI_ISL_2507095, EPI_ISL_2603820, EPI_ISL_2661522, EPI_ISL_2661525, EPI_ISL_2675252, EPI_ISL_2675253, EPI_ISL_2727637, EPI_ISL_2811889, EPI_ISL_2811892, EPI_ISL_2811904, EPI_ISL_2842840, EPI_ISL_2842841, EPI_ISL_2842842, EPI_ISL_2842843, EPI_ISL_2896248, EPI_ISL_2896249, EPI_ISL_2956008, EPI_ISL_2993362, EPI_ISL_2993401, EPI_ISL_2993405, EPI_ISL_2993415, EPI_ISL_2993422, EPI_ISL_2993425, EPI_ISL_2993426, EPI_ISL_2993427			
see above	Queensland Health Forensic and Scientific Services	Queensland Health Forensic and Scientific Services	Son Nguyen; Son Nguyen et al; Son Nguyen et al.
EPI_ISL_530239, EPI_ISL_530279	Queensland Health Forensic and Scientific Services, Public Health Virology	Public Health Virology Laboratory, Forensic and Scientific Services, Queensland Health	Son Nguyen et al
EPI_ISL_1424519	Queensland Medical Laboratories	Victorian Infectious Diseases Reference Laboratory (VIDRL) and the Melbourne Diagnostic Unit Public Health Laboratory (MDU-PHL)	N.L.; Palou, T.; Seemann, T.; Sherry; Vaccher, S.
EPI_ISL_494533, EPI_ISL_571261, EPI_ISL_571975, EPI_ISL_604114	Quest Diagnostics	Quest Diagnostics	Anderson; Anderson, B.; B.P.; D.F.; Gerasimova, A.; Grover, D.; Hua, M.; K.E.; Kagan; Lacbawan, F.; Liu Y.; Livingston; Owen, R.; R.M.; R.M. and Owen, R.; Rosenthal; S.H.; Shalhout
EPI_ISL_1220443, EPI_ISL_1253267, EPI_ISL_1648870, EPI_ISL_1668705, EPI_ISL_1753408, EPI_ISL_1762016, EPI_ISL_1818271, EPI_ISL_1924348, EPI_ISL_1989864, EPI_ISL_2047081, EPI_ISL_2143545, EPI_ISL_2143956, EPI_ISL_2180464, EPI_ISL_2247212, EPI_ISL_2247292, EPI_ISL_2371834, EPI_ISL_2371912, EPI_ISL_2397489, EPI_ISL_2451305, EPI_ISL_2649980, EPI_ISL_2652295, EPI_ISL_2652368, EPI_ISL_2869180, EPI_ISL_2874567, EPI_ISL_2874761, EPI_ISL_2875140, EPI_ISL_2876694, EPI_ISL_2876857, EPI_ISL_2930261			
see above	Quest Diagnostics Incorporated	Centers for Disease Control and Prevention Division of Viral Diseases, Pathogen Discovery	A. Gerasimova; A. Perez; Adrian Paskey; B. Anderson; Ben L. Rambo-Martin; Benjamin Rambo-Martin; Christopher Gulvick; Clinton R. Paden; Dakota Howard; Darlene Wagner; Dhwani Batra; Duncan MacCannell; F. Lacbawan; I. A. Shlyakhter; Jason Caravas; K.E. Livingston; Kara Moser; L.E. Bernstein; M. Hua; Matthew Schmerer; P. Tanpaiboon; Peter W. Cook; R. M. Kagan; R. Owen; R. V. Rolando; S. H. Rosenthal; Scott Sammons; Shatavia Morrison; Suxiang Tong; Y. Liu; Yvette Unoarumhi
EPI_ISL_1086247, EPI_ISL_1087772, EPI_ISL_1087817	Quest Diagnostics Incorporated	Respiratory Viruses Branch, Division of Viral Diseases, Centers for Disease Control and Prevention	A. Gerasimova; A. Perez; B. Anderson; Ben L. Rambo-Martin; Clinton R. Paden; Dakota Howard; Dhwani Batra; Duncan MacCannell; F. Lacbawan; I. A. Shlyakhter; K.E. Livingston; L.E. Bernstein; M. Hua; P. Tanpaiboon; Peter W. Cook; R. M. Kagan; R. Owen; R. V. Rolando; S. H. Rosenthal; Suxiang Tong; Y. Liu
EPI_ISL_1711855	RI State Health Laboratories	Centers for Disease Control and Prevention Division of Viral Diseases, Pathogen Discovery	Alison Laufer Halpin; Ben L. Rambo-Martin; Clinton R. Paden; Dakota Howard; Darlene Wagner; Dave Wentworth; Dhwani Batra; Jasmine Padilla; Justin Lee; Katie Dillon; Krista Queen; Kristen Knipe; Kristine Lacek; Mark Burroughs; Matthew Schmerer; Mili Sheth; Peter Cook; Sam Shepard; Sarah Nobles; Shoshona Le; Suxiang Tong; Vivien Dugan; Yvette Unoarumhi
EPI_ISL_2531885, EPI_ISL_2531926, EPI_ISL_2531934	RIIP	National Reference Center for Viruses of Respiratory Infections, Institut Pasteur, Paris	Angela Brisebarre; Camille Capel; Christophe Malabat; Corinne Maufrais; Etienne Simon-Lorière; Frédéric Lemoine; Louise Lefrançois; Marion Barbet; Maud Vanpeene; Méline Bizard; StéPhanie Guyomard-Rabenirina; Sylvie Behillil; Sylvie Van der Werf; Vincent Enouf
EPI_ISL_413581	RIVM	Erasmus Medical Center	Anne van der Linden; Annemiek van der Eijk; Aura Timen; Bas Oude Munnink; Claudia Schapendonk; Corien Swaan; Corine GeurtsvanKessel; David Nieuwenhuijse; Irina Chestakova; Jeroen van Kampen; Jolanda Voermans; Madelief Mollers; Manon Haverkate; Marion Koopmans; Mark Pronk; Mart Stein; Pascal Lexmond; Reina Sikkema; Richard Molenkamp; Sandra Kengne Kamga Mobou; on behalf of the Dutch national COVID-19 response team.
EPI_ISL_2086951, EPI_ISL_2273059, EPI_ISL_2273060, EPI_ISL_2273061, EPI_ISL_2273063, EPI_ISL_2273065, EPI_ISL_2273067, EPI_ISL_2273068, EPI_ISL_2273075, EPI_ISL_2273082, EPI_ISL_2273087, EPI_ISL_2442379, EPI_ISL_2444100, EPI_ISL_2444106, EPI_ISL_2454669, EPI_ISL_2454670, EPI_ISL_2454676, EPI_ISL_2454683, EPI_ISL_2454685, EPI_ISL_2454688, EPI_ISL_2454690, EPI_ISL_2454695, EPI_ISL_2582830, EPI_ISL_2582833, EPI_ISL_2695608, EPI_ISL_2695609, EPI_ISL_2695615, EPI_ISL_2695620, EPI_ISL_2695621, EPI_ISL_2695623, EPI_ISL_2695625, EPI_ISL_2695754, EPI_ISL_2695755, EPI_ISL_2695763, EPI_ISL_2695764, EPI_ISL_2695767, EPI_ISL_2695771			
see above	ROB FERREIRA LABORATORY	National Institute for Communicable Diseases of the National	Amoako DG; Bhiman JN; Everatt J; Ismail A; Mahlangu B; Mnguni A; Mohale T; Ntuli N; Scheepers C

		Health Laboratory Service	
EPI_ISL_3138836	RS Jantung Dan Pembuluh Darah Harapan Kita	National Institute of Health Research and Development	Arie Ardiansyah Nugraha; Fajar Nur Sulistiyohadi; Hana Apsari Pawestri; Hartanti Dian Ikawati; Kartika Dewi Puspa; Krisna Pangesti; Nelly Puspandari; Subangkit; Triyani Soekarso; Vivi Setiawaty
EPI_ISL_763064	RS Kasih Ibu, Surakarta	Universitas Sebelas Maret (UNS); Rumah Sakit UNS (RS-UNS); National Institute of Health Research and Development, Indonesian Ministry of Health.	Afif A Ghufron; Arie A Nugraha; Betty Suryawati; Dewi Puspa; Hana A Pawestri; Hartanti Dian Ika; Hartono; Kartika; Maryani; Revi G H Novika; Vivi Setiawaty.; Yulia Sari
EPI_ISL_2987618	RS Mata Cicendo Bandung	National Institute of Health Research and Development	Arie Ardiansyah Nugraha; Fajar Nur Sulistiyohadi; Hana Apsari Pawestri; Hartanti Dian Ikawati; Kartika Dewi Puspa; Krisna Pangesti; Nelly Puspandari; Subangkit; Triyani Soekarso; Vivi Setiawaty
EPI_ISL_574611	RS Premier Bintaro	Eijkman Institute for Molecular Biology, Ministry of Research and Technology/National Agency for Research and Innovation	Amin Soebandrio; David H Muljono; Edison Johar; Frilasita A Yudhaputri; Herawati Sudoyo; Hidayat Trimarsanto; Iskandar A Adnan; Khin Saw Myint; Safarina G Malik; Willy Agustine
EPI_ISL_2709370	RS Pusat Pertamina	Eijkman Institute for Molecular Biology, National Research and Innovation Agency	Amin Soebandrio; Edison Johar; Frilasita A Yudhaputri; Hidayat Trimarsanto; Iskandar Adnan; Khin Saw Myint; Lidwina Priliani; Lydia V. Panggalo; Muhammad Rezki Rasyak; Safarina G Malik; Sukma Oktavianthi; Willy Agustine
EPI_ISL_2262254, EPI_ISL_2262265	RS Umum Kartika Pulomas	National Institute of Health Research and Development	Arie Ardiansyah Nugraha; Hana Apsari Pawestri; Hartanti Dian Ikawati; Kartika Dewi Puspa; Krisna Pangesti; Nelly Puspandari; Subangkit; Triyani Soekarso; Vivi Setiawaty
EPI_ISL_906051	RSA UGM	Genetics Working Group (Pokja Genetik) Faculty of Medicine, Public Health and Nursing Universitas Gadjah Mada (FK-KMK UGM); Disease Investigation Center Wates Ministry of Agriculture Indonesia; Department of Microbiology FK-KMK UGM; Laboratorium Diagnostik Yayasan Tahija World Mosquito Program (WMP) Yogyakarta Center for Tropical Medicine FK-KMK UGM; Integrated Research center FK-KMK UGM; Department of Computer Science and Electronics FMIPA UGM	Afiahayati; Alvin Santoso Kalim; Dwi AA Nugrahaningsih; Dwiki Afandy; Edwin W. Daniwijaya; Eggi Arguni; Endah Supriyati; Gunadi; Hendra Wibawa; Kristy Iskandar; Ludhang P. Rizki; Marcellus; Mohamad S. Hakim; Nungki Anggorowati; Siswanto; Titik Nuryastuti; Tri Wibawa
EPI_ISL_454520	RSE "National Center for Biotechnology"	RSE "National Center for Biotechnology"	Akbota Rakhmetova; Alexandr Shevtsov; Askar Abdaliyev; Asylulan Amirgazin; Ilyas Akhmetollayev; Ruslan Kalendar; Viktoriya Lutsay; Yerlan Ramankulov; Zabira Aushakhmetova
EPI_ISL_947247	RSU Bunda Mulia	Eijkman Institute for Molecular Biology, Ministry of Research and Technology/National Agency for Research and Innovation	Amin Soebandrio; Edison Johar; Frilasita A Yudhaputri; Hidayat Trimarsanto; Iskandar Adnan; Khin Saw Myint; Lydia V. Panggalo; Safarina G Malik; Sukma Oktavianthi; Willy Agustine
EPI_ISL_947298	RSU Medirossa	Eijkman Institute for Molecular Biology, Ministry of Research and Technology/National Agency for Research and Innovation	Amin Soebandrio; Edison Johar; Frilasita A Yudhaputri; Hidayat Trimarsanto; Iskandar Adnan; Khin Saw Myint; Lydia V. Panggalo; Safarina G Malik; Sukma Oktavianthi; Willy Agustine
EPI_ISL_560991	RSUD Dr. Soetomo	Institute of Tropical Disease, Universitas Airlangga	Aldise M Nastri; Gatot Soegiarto; Jezzy R Dewantari; Joni Wahyuhadi; Kazufumi Shimizu; Krisnoadi Rahardjo; Laksmi Wulandari; Maria I Lusida; Mitsuhiro Nishimura; Resti Yudhawati; Retno A Setyoningrum; Rima R Prasetya; Soetjipto; Yasuko Mori; Yohko K Shimizu
EPI_ISL_610162, EPI_ISL_1005696	RSUD Dr. Tjitrowardojo	Genetics Working Group (Pokja Genetik) Faculty of Medicine, Public Health and Nursing Universitas Gadjah Mada (FK-KMK UGM); Disease Investigation Center Wates Ministry of Agriculture Indonesia; Department of Microbiology FK-KMK UGM; Laboratorium Diagnostik Yayasan Tahija World Mosquito Program (WMP) Yogyakarta Center for Tropical Medicine FK-KMK UGM; Integrated Research Center FK-KMK UGM; Department of Computer Science and Electronics FMIPA UGM; Balai Besar Teknik Kesehatan Lingkungan dan Pengendalian Penyakit (BBTKLPP) Yogyakarta	Afiahayati; Alvin Santoso Kalim; Arry Setyowati; Chusni Mubarakh; Dwi AA Nugrahaningsih; Edwin W. Daniwijaya; Eggi Arguni; Endah Supriyati; Gunadi; Havid Setyawan; Hendra Wibawa; Indaryati; Irene; Kristy Iskandar; Ludhang P. Rizki; Marcellus; Mohamad S. Hakim; Nungki Anggorowati; Siswanto; Susan Simanjaya; Titik Nuryastuti; Tri Wibawa; Try Nirmala Sari; Wuryanto
EPI_ISL_2854750	RSUD Dr. W.Z. Johanes kupang	National Institute of Health Research and Development	Arie Ardiansyah Nugraha; Hana Apsari Pawestri; Hartanti Dian Ikawati; Kartika Dewi Puspa; Krisna Pangesti; Nelly Puspandari; Subangkit; Triyani Soekarso; Vivi Setiawaty
EPI_ISL_2631472	RSUD Karawang	Indonesian Institute of Sciences (LIPI)/National Agency of Research and Innovation (BRIN) and National Institute of Health Research and Development (NIHRD)	Andri Wardiana; Herjuno A. Nugroho; Idris; Indriawati; Isa Nuryana; Listiana Oktavia; Puspita Lisdiyanti; Sri Swasthikawati; Subangkit; Sugiyono Saputra; Syam B. Iryanto; Vivi Setiawaty; Yayuk Sri Rahayu; Yuliawati
EPI_ISL_632937	RSUD Saptosari Gunung Kidul	Genetics Working Group (Pokja Genetik) Faculty of Medicine, Public Health and Nursing Universitas Gadjah Mada (FK-KMK UGM); Disease Investigation Center Wates Ministry of Agriculture Indonesia; Department of Microbiology FK-KMK UGM; Laboratorium Diagnostik Yayasan Tahija World Mosquito Program (WMP) Yogyakarta Center for Tropical Medicine FK-KMK UGM; Integrated Research Center FK-KMK UGM; Department of Computer Science and Electronics FMIPA UGM; Balai Besar Teknik Kesehatan Lingkungan dan Pengendalian Penyakit (BBTKLPP) Yogyakarta	Afiahayati; Audric Kenny Tedja; Dwi AA Nugrahaningsih; Edwin W. Daniwijaya; Eggi Arguni; Eko Darmawan; Endah Supriyati; Gunadi; Havid Setyawan; Hendra Wibawa; Indaryati; Irene; Kristy Iskandar; Ludhang P. Rizki; Marcellus; Maria Patricia Inggriani; Mohamad S. Hakim; Nungki Anggorowati; Siswanto; Titik Nuryastuti; Tri Wibawa
EPI_ISL_1824615	RSUD dr. Hasri Ainun Habibie Gorontalo	National Institute of Health Research and Development	Arie Ardiansyah Nugraha; Hana Apsari Pawestri; Hartanti Dian Ikawati; Kartika Dewi Puspa; Krisna Pangesti; Nelly Puspandari; Subangkit; Vivi Setiawaty
EPI_ISL_2824996	RSUP Dr. Hasan Sadikin Bandung	Genomik Solidaritas Indonesia Laboratorium	Alfin Mohammad Abdillah; Anna Christina Brazia; Annisa Muthiah Sukirman; Anuraj Shankar; Ariel Pradipta; Basti Andriyoko; Carissa Sintca Wijaya; Delita Prihatni; Dewi Kartika Turbawati; Dhahlia Agustina Cahyono; Gracia Felias Enos Korompis; Louisa Markus; Meutia Ayuputeri Kumaheri; Normastuti Adhini Tantyo; Reinhart Gabriel; Vania Gavrila Wikasa; Vivi Setyawati
EPI_ISL_766032	RSUP Dr. Sardjito	Eijkman Institute for Molecular Biology, Ministry of Research and Technology/National Agency for Research and Innovation	Amin Soebandrio; David H Muljono; Edison Johar; Frilasita A Yudhaputri; Hidayat Trimarsanto; Khin Saw Myint; Safarina G Malik
EPI_ISL_1824606	RSUP Sanglah	National Institute of Health Research and Development	Arie Ardiansyah Nugraha; Hana Apsari Pawestri; Hartanti Dian Ikawati; I Gede Ketut Sajinadiyasa; I Nengah Tony Rustawan; Kartika Dewi Puspa; Krisna Pangesti; Nelly Puspandari; Subangkit; Vivi Setiawaty
EPI_ISL_2827879, EPI_ISL_2827884, EPI_ISL_2827886, EPI_ISL_2895681, EPI_ISL_2895682, EPI_ISL_2895683, EPI_ISL_2895690, EPI_ISL_2895693			
see above	RUSTENBURG LABORATORY	National Institute for Communicable Diseases of the National Health Laboratory Service	Amoako DG; Bhiman JN; Everatt J; Ismail A; Mahlangu B; Mnguni A; Mohale T; Ntuli N; Scheepers C
EPI_ISL_450508, EPI_ISL_450515	Rafik Hariri University Hospital	Rafik Hariri University Hospital	Rita Feghali
EPI_ISL_2678109	Ramathibodi Chakri Naruebodindra Hospital	COVID-19 Network Investigations (CONI) Alliance	Angkana Huang; Anthony R. Jones; Arporn Wangwiwatsin; Bhakbhoom Panthan; Chonticha Klungtong; Duangkamon Loesbanluechai; Ekawat Pasomsub; Elizabeth Batty; Insee Sensorn; Janjira Thaipadungpanit; Khajohn Joonlasak; Kingkan Rakmanee; Krittikorn Kumpornsin; Namfon Kotanan; Stefan Fernandez; Thanat Chookajorn; Theerarat Kochakarn; Treewat Watthanachockchai; Wasun Chantratita; Wudtichai Manasatienkij
EPI_ISL_429168, EPI_ISL_2104753, EPI_ISL_2678105, EPI_ISL_2802837, EPI_ISL_2802852, EPI_ISL_2802853, EPI_ISL_2928525			
see above	Ramathibodi Hospital	COVID-19 Network Investigations (CONI) Alliance	Angkana Huang; Anthony R. Jones; Arporn Wangwiwatsin; Bhakbhoom Panthan; Chonticha Klungtong; Duangkamon Loesbanluechai; Ekawat Pasomsub; Elizabeth Batty; Insee Sensorn; Janjira Thaipadungpanit; Khajohn Joonlasak; Khajohn Joonsalak; Kingkan Rakmanee; Krittikorn Kumpornsin; Namfon Kotanan; Poramate Jiaranai; Stefan Fernandez; Thanat Chookajorn; Theerarat Kochakarn; Treewat Watthanachockchai; Wasun Chantratita; Wudtichai Manasatienkij
EPI_ISL_1275880, EPI_ISL_1275902	Randox Laboratories	Wellcome Sanger Institute for the COVID-19 Genomics UK (COG-UK) Consortium	Cordelia Langford; David K. Jackson; Dominic Kwiatkowski; Ewan Harrison; Ian Johnston; Jeffrey Barrett; John Sillitoe on behalf of the Wellcome Sanger Institute COVID-19 Surveillance Team; Randox Laboratories and Alex Alderton; Roberto Amato; Sonia Goncalves
EPI_ISL_2433337	Rattaphum Hospital	Division of Genomic Medicine and Innovation support,Department of Medical Sciences, Ministry of Public Health, Thailand	Archawin Rojanawiwat; Jirapha Pakdee; Natthakul Bunneang; Nuanjun Wichukchinda; Penpitcha Thawong; Pilailuk Akkapaiboon Okada; Pundharika Piboonsiri; Surakameth Mahasirimongkol; Waritta Sawaengdee
EPI_ISL_2157357	Red - Regional de Vigilancia Genómica del COVID-19	Laboratory of Respiratory Viruses and Measles, Oswaldo Cruz Institute, FIOCRUZ	Alice Sampaio Rocha; Ana Carolina Mendonca; Anna Carolina Paixao; Claudia Díaz; Eilisa Cavalcante Pereira; Fernando Motta; Luciana Appolinario; Marilda Siqueira on behalf of the Fiocruz COVID-19 Genomic Surveillance Network; Mitzi Castro; Paola Resende; Renata Serrano Lopes; Sandra Paola Paz; Taina Venas
EPI_ISL_1415406, EPI_ISL_1415407, EPI_ISL_1415412, EPI_ISL_1415413, EPI_ISL_1415419	Reference Laboratory of the Ministry of Health	Laboratory of Respiratory Viruses and Measles, Oswaldo Cruz Institute, FIOCRUZ	Alice Sampaio Rocha; Ana Carolina Mendonca; Anna Carolina Paixao; Fernando Motta; Indira Martins; Jessica Edwards; Luciana Appolinario; Marilda Siqueira on behalf of the Fiocruz COVID-19 Genomic Surveillance Network; Paola Resende; Renata Serrano Lopes
EPI_ISL_2982838, EPI_ISL_2982850, EPI_ISL_2982851, EPI_ISL_2982858, EPI_ISL_2982860, EPI_ISL_2982861, EPI_ISL_2982863			
see above	Reference Laboratory of the Ministry of Health Royal Victoria Gardens	Laboratory of Respiratory Viruses and Measles, Oswaldo Cruz Institute, FIOCRUZ	Agatha Cristinne Prudencio; Alice Sampaio Rocha; Ana Carolina Mendonca; Anna Carolina Paixao; Elisa Cavalcante Pereira; Fernando Motta; Ighor Leonardo Arantes Gomes; Indira Martins; Jessica Edwards; Luciana Appolinario; Marilda Siqueira on behalf of the Fiocruz COVID-19 Genomic Surveillance Network; Paola Resende; Renata Serrano Lopes; Taina Venas
EPI_ISL_2276073, EPI_ISL_2276076	Regional Authority of Public Health Trenčín	Veterinary institute in Zvolen Slovakia	Beňová K.; Bučková L.; Dirbáková Z.; Mojžiš M.; Mokryšová S.; Reizigová L.; Sujová S.; Tinák M.
EPI_ISL_768617	Regional Medical Sciences Center 5 Samut Songkhram	National Institute of Health, Department of Medical Sciences, Ministry of Public Health, Thailand	; Natchaya Khiadsang; Pakorn Piromtong; Pilailuk Okada; Ratana Tacharoenmuang; Siripaporn Phuygun; Sittiporn Parnmen; Sunthareeya Waicharoen; Thanutsapa Thanadachakul; Warawan Wongboot; sirikanda wimol
EPI_ISL_2877034	Regional Medical Sciences Center 8 Udonthani	National Institute of Health, Department of Medical Sciences, Ministry of Public Health, Thailand	; Natchaya Khiadsang; Nuttida Thongpramul; Pakorn Piromtong; Pilailuk Okada; Ratana Tacharoenmuang; Siripaporn Phuygun; Sittiporn Parnmen; Sunthareeya Waicharoen; Thanutsapa Thanadachakul; Warawan Wongboot; sirikanda wimol
EPI_ISL_708806, EPI_ISL_708807	Regional medical sciences center 6 chonburi	National Institute of Health, Department of Medical Sciences, Ministry of Public Health, Thailand	Malinee Chittaganpitch; Pakorn Piromtong; Pilailuk Okada; Siripaporn Phuygun; Sittiporn Parnmen; Sunthareeya Waicharoen; Thanutsapa Thanadachakul; Warawan Wongboot
EPI_ISL_2544703, EPI_ISL_2544707,	Research Center for Emerging Viral Infections, Chang Gung University, Taiwan	Research Center for Emerging Viral Infections, Chang Gung University, Taiwan	Carol Wang; Chung-Guei Huang; Hsiao-Chen Tu; Hui-Ying Weng; Hung-Yu Shu; Jason Su; Jora Lin; Kuo-Ming Lee; Po-Wei Huang; Pocky Lai; Shih-Feng Tsai; Shin-Ru Shih; Shu-Li Yang; Tsu-Lan Wu; Yu-Nong Gong; Yung-Feng Lin

EPI_ISL_2544708, EPI_ISL_2544710, EPI_ISL_2544711, EPI_ISL_2544713			
EPI_ISL_2740439, EPI_ISL_2740490, EPI_ISL_2740549, EPI_ISL_2741203, EPI_ISL_2741308, EPI_ISL_2741766, EPI_ISL_2741814, EPI_ISL_2742068, EPI_ISL_2748953, EPI_ISL_2748955, EPI_ISL_2749048, EPI_ISL_2749111, EPI_ISL_2749225, EPI_ISL_2904339, EPI_ISL_2904411			
see above	Respiratory Virus Unit, Microbiology Services Colindale, Public Health England	COVID-19 Genomics UK (COG-UK) Consortium	PHE Covid Sequencing Team
EPI_ISL_423103, EPI_ISL_423104, EPI_ISL_423207, EPI_ISL_464172	Respiratory Virus Unit, Microbiology Services Colindale, Public Health England	Respiratory Virus Unit, Microbiology Services Colindale, Public Health England	Angie Lackenby; Joanna Ellis; Jonathan Hubb; Kirstin Edwards; Leena Bhaw; Maria Zambon; Monica Galiano; Omolola Akinbami; PHE Covid Sequencing Team; Richard Myers; Shahjahan Miah; Steven Platt; Tiina Talts
EPI_ISL_747100	Respiratory Viruses Branch, Centers for Disease Control and Prevention	Respiratory Viruses Branch, Centers for Disease Control and Prevention	C.R.; Cook; Lee, J.; Li, Y.; Marine, R.; Montmayeur, A.; P.W.; Paden; Queen, K.; Sheth, M.; Tao, Y.; Tong, S.; Uehara, A.; Wang, H.
EPI_ISL_2603892	Rhode Island State Health Laboratory	Rhode Island State Health Laboratory	Ewa King; Kristin Carpenter-Azevedo; Richard C. Huard
EPI_ISL_2799553, EPI_ISL_2800292	Riga East University Hospital, National Microbiology Reference Laboratory	Riga East University Hospital, National Microbiology Reference Laboratory; Eurofins Genomics Europe Sequencing GmbH	Arzu Algulieva; Diāna Dušacka; Dārta Pūpola; Ilva Pole; Jevgenijs Bodrenko; Jūlija Čevere; Reinis Vangravs; Reinis Zeltmatis; Sergejs Ņikišins; Ģirts Šķenders
EPI_ISL_1239479	Rob Ferreira Hospital, National Health Laboratory Services, Mpumalanga, South Africa	National Institute for Communicable Diseases of the National Health Laboratory Service	Amoako DG; Bhiman JN; Ismail A; Mahlangu B; Mohale T; Ntuli N; Scheepers C
EPI_ISL_2447836, EPI_ISL_2447870	Rob Ferreira Laboratory	National Institute for Communicable Diseases of the National Health Laboratory Service	Amoako DG; Bhiman JN; Ismail A; Mahlangu B; Mohale T; Ntuli N; Scheepers C
EPI_ISL_419833, EPI_ISL_419835	Royal Darwin Hospital	Victorian Infectious Diseases Reference Laboratory and Microbiological Diagnostic Unit Public Health Laboratory, Doherty Institute	Caly L.; Druce J.; Meumann, E.; Sait, M.; Schultz M.; Seemann T.
EPI_ISL_521862, EPI_ISL_779406, EPI_ISL_779408, EPI_ISL_779409, EPI_ISL_779413, EPI_ISL_812423, EPI_ISL_2321158, EPI_ISL_2321160, EPI_ISL_2321161, EPI_ISL_2321163, EPI_ISL_2321166, EPI_ISL_2379269, EPI_ISL_2839562, EPI_ISL_2839563, EPI_ISL_2839567, EPI_ISL_2839568, EPI_ISL_2907544, EPI_ISL_2907545, EPI_ISL_2907546, EPI_ISL_3030412			
see above	Royal Darwin Hospital Pathology	MDU-PHL	Caly L.; Druce J.; M.L.; Meumann, E.; N.L.; Sait; Sait, M.; Schultz M.; Seemann T.; Sherry; Sherry, N.
EPI_ISL_2250194, EPI_ISL_2250196, EPI_ISL_2250198, EPI_ISL_2250204, EPI_ISL_2250206, EPI_ISL_2250209, EPI_ISL_2250214, EPI_ISL_2250216, EPI_ISL_2250217, EPI_ISL_2250219			
see above	Royal Darwin Hospital Pathology	Microbiological Diagnostic Unit Public Health Laboratory (MDU-PHL)	Caly L.; Druce J.; M.L.; Meumann, E.; N.L.; Sait; Seemann T.; Sherry
EPI_ISL_426898	Royal Darwin Hospital Pathology	Microbiological Diagnostic Unit Public Health Laboratory and Victorian Infectious Diseases Reference Laboratory, Doherty Institute	Caly L.; Druce J.; Meumann, E.; Sait, M.; Schultz M.; Seemann T.; Sherry, N.
EPI_ISL_430631	Royal Darwin Hospital Pathology	Microbiological Diagnostic Unit Public Health Laboratory and Victorian Infectious Diseases Reference Laboratory, The Peter Doherty Institute for Infection and Immunity	Caly L.; Druce J.; Meumann, E.; Sait, M.; Schultz M.; Seemann T.; Sherry, N.
EPI_ISL_577607	Royal Hobart Hospital	Royal Hobart Hospital	Cooley L.; van Haeften R.
EPI_ISL_522621, EPI_ISL_522739, EPI_ISL_522745	Royal Hobart Hospital Microbiology Department	MDU-PHL	Cooley L.; M.B.; Sait M.; Schultz; Seemann T.; Sherry N.; van Haeften R.
EPI_ISL_516806	Rumah Sakit PKU Gamping	Genetics Working Group (Pokja Genetik) Faculty of Medicine, Public Health and Nursing Universitas Gadjah Mada (FK-KMK UGM); Disease Investigation Center Wates Ministry of Agriculture Indonesia; Department of Microbiology FK-KMK UGM; Laboratorium Diagnostik Yayasan Tahija World Mosquito Program (WMP) Yogyakarta Center for Tropical Medicine FK-KMK UGM; Integrated Research center FK-KMK UGM; Department of Computer Science and Electronics FMIPA UGM	. Afiahayati; . Marcellus; . Siswanto; Ardorisye Saptaty Fornia; Dwi AA Nugrahaningsih; Edwin W. Daniwijaya; Eggi Arguni; Endah Supriyati; Gunadi; Hendra Wibawa; Kemala Athollah; Ludhang P. Rizki; Mohamad S. Hakim; Titik Nuryastuti; Tri Wibawa
EPI_ISL_735445	Rwanda National Reference Laboratory	GIGA Medical Genomics	Bouchra Boujemla; Esperence Umumararungu; Jacob Souopgui; Keith Durkin; Léon Mutesa; Maria Artesi; Marie-Pierre Hayette; Patrick Tuyisenge; Robert Rutayisire; Sabin Nsanzimana; Swaibu Gatare; Sébastien Bontems; Vincent Bours; Yvan Butera
EPI_ISL_1063900, EPI_ISL_1064168	Rwanda National Reference Laboratory	Rwanda National Reference Laboratory	Enatha Mukantwari; Umuringa Jeanne d'Arc
EPI_ISL_451127, EPI_ISL_451144, EPI_ISL_467989, EPI_ISL_468024, EPI_ISL_468039, EPI_ISL_508138, EPI_ISL_510544, EPI_ISL_513911, EPI_ISL_603124, EPI_ISL_603127, EPI_ISL_654795, EPI_ISL_654800, EPI_ISL_755568, EPI_ISL_812358, EPI_ISL_1704675, EPI_ISL_1704796, EPI_ISL_1704798, EPI_ISL_1704799, EPI_ISL_1704804, EPI_ISL_1706366, EPI_ISL_1706367			
see above	SA Pathology	SA Pathology	Chuan Kok Lim; Geoff Higgins; Ivan Bastian; Julien Soubrier; Karin Kassahn; Lex Leong; Mark Turra; Song Gao
EPI_ISL_1603794	SARS-CoV-2 Sequencing Castilla y Leon-Spain Consortium	SARS-CoV-2 Sequencing Castilla y Leon-Spain Consortium	Antonio Orduña-Domingo; Carlos Fuster Foz; Carmen Aldea-Mansilla; Carmen Gimeno Crespo; David Abad; Gregoria Megías Lobón; Jose María Eiros Bouza; Laura Sánchez de Prada; M. Isabel Fernandez-Natal; Marta Dominguez-Gil; Marta Hernandez; María Antonia García Castro; Mª Fe Brezmes-Valdivieso; Noelia Arenal Andrés; Silvia Rojo; Sonsoles Garcinuño Pérez
EPI_ISL_1046778, EPI_ISL_1198812, EPI_ISL_1927132, EPI_ISL_1927133, EPI_ISL_1927135, EPI_ISL_1927136, EPI_ISL_1927138, EPI_ISL_1927139, EPI_ISL_1927308, EPI_ISL_1927364, EPI_ISL_1927368, EPI_ISL_1927381, EPI_ISL_1927389, EPI_ISL_1927397, EPI_ISL_1927435, EPI_ISL_1927441, EPI_ISL_2131776, EPI_ISL_2131792, EPI_ISL_2447620, EPI_ISL_2447623, EPI_ISL_2447628, EPI_ISL_2447634, EPI_ISL_2447692, EPI_ISL_2621762, EPI_ISL_2674807, EPI_ISL_2828265, EPI_ISL_2828268, EPI_ISL_2930625, EPI_ISL_2933814			
see above	SARS-CoV-2 testing team, National Institute of Infectious Diseases	Pathogen Genomics Center, National Institute of Infectious Diseases	Chang-Kweng Lim; Eri Nakayama; Hazuka Y Furihata; Hidemasa Izumiya; Hiromizu Takahashi; Ken Miyazawa; Ken Shimuta; Ken-ichi Lee; Kentaro Itokawa; Koichi Ishikawa; Makoto Kuroda; Masanori Hashino; Masatomo Morita; Masumichi Saito; Minoru Nagi; Naomi Nojiri; Nobuo Koizumi; Nozomu Hanaoka; Rina Tanaka; Sana Uchikoba; Sayuri Seki; Shigeru Kusagawa; Shigeru Tajima; Shouji Yamamoto; Sunao Iyoda; Takashi Sakudoh; Tsuguto Fujimoto; Tsuyoshi Sekizuka
EPI_ISL_2324433, EPI_ISL_2567592, EPI_ISL_2828393	SC (UCO) Igiene e Sanità Pubblica, ASUGI, Trieste	ARGO Laboratorio Genomica ed Epigenomica	Barbone F; Braida C; Breda C; Busetti M; D'Agaro P; Dal Monego S; Degasperi M; Fontana F; Licastro D; Marcello A; Piscianz E; Segat L
EPI_ISL_2828450	SC (UCO) Igiene e Sanità Pubblica, ASUGI, Trieste and SC Microbiologia e Virologia, ASFO, Pordenone	ARGO Laboratorio Genomica ed Epigenomica	Barbone F; Breda C; D'Agaro P; Dal Monego S; De Rosa R; Degasperi M; Licastro D; Marcello A; Segat L
EPI_ISL_424841	SC Dept of Health and Env. Control-Bureau of Laboratories	Pathogen Discovery, Respiratory Viruses Branch, Division of Viral Diseases, Centers for Disease Control and Prevention	Alison S. Laufer Halpin; Anna Uehara; Christopher A. Elkins; Clinton R. Paden; Haibin Wang; Jing Zhang; Krista Queen; Mary S. Keckler; Rachel Marine; Suxiang Tong; Yan Li; Ying Tao
EPI_ISL_2482956	SCM B12	CNR Virus des Infections Respiratoires - France SUD	Antonin Bal; Bruno Lina; Bruno Simon; Gregory Destras; Gwendolyne Burfin; Hadrien Regue; Laurence Josset; Martine Valette; Quentin Semanas
EPI_ISL_2018218	SD Public Health Laboratory	Centers for Disease Control and Prevention Division of Viral Diseases, Pathogen Discovery	Alison Laufer Halpin; Ben L. Rambo-Martin; Clinton R. Paden; Dakota Howard; Darlene Wagner; Dave Wentworth; Dhwani Batra; Jasmine Padilla; Justin Lee; Katie Dillon; Krista Queen; Kristen Knipe; Kristine Lacek; Mark Burroughs; Matthew Schmerer; Mili Sheth; Peter Cook; Sam Shepard; Sarah Nobles; Shoshona Le; Suxiang Tong; Vivien Dugan; Yvette Unoarumhi
EPI_ISL_2934571, EPI_ISL_2966511	SE Kyiv CDC MHU	The Institute of Molecular Biology and Genetics of NASU	M.Tukalo et al.
EPI_ISL_1732275	SECAO CENTRO DE DIAGNOSTICO SECEDI	Instituto Butantan / Mendelics	Antonio Jorge Martins; Bianca Cechetto Carlos. Mendelics: Bibiana Santos; Claudia Renata dos Santos Barros; David Schlesinger. Hemocentro Ribeirão Preto: Simone Kashima; Debora Botequio Moretti. Centro de Genômica Funcional da ESALQ: Luiz Lehmann Coutinho; Dimas Tadeu Covas; Elaine Cristina Marqueze; Elaine Vieira dos Santos; Elisangela Chicaroni Mattos; Erika Freitas; Evandra Strazza Rodrigues; Felipe Allan da Silva da Costa; Flavia Aburjaile; Guilherme Targino Valente; Heidge Fukumasu. USP-Botucatu: Rejane Maria Tommasini Grotto; Instituto Butantan: Alexander Roberto Precioso; Jayme A. Souza-Neto; Jessika Cristina Chagas Lesbon; José Salvatore Leister Patané; João Paulo Kitajima; Luiz Carlos Junior de Alcantara; Maria Carolina Elias; Marta Giovanetti; Patricia Akemi Assato; Rafael dos Santos Bezerra; Raquel de Lello Rocha Campos Cassano. NGS Soluções Genômicas: Pilar Drummond Sampaio Corrêa Mariani. FZEA-USP Pirassununga: Mirele Daiana Poleti; Raul Machado Neto; Ricardo Augusto Brassaloti; Ricardo Haddad; Rodrigo Tocantins Calado.; Sandra Coccuzzo Sampaio; Svetoslav Nanev Slavov; Vagner Fonseca; Vincent Louis Viala
EPI_ISL_2934570, EPI_ISL_2934573, EPI_ISL_2934574, EPI_ISL_2966512	SECP MHU	The Institute of Molecular Biology and Genetics of NASU	M.Tukalo et al.
EPI_ISL_2938096	SECRETARIA MUNICIPAL DA SAUDE DE GUARATINGUETA LABORATÓRIO 01	Instituto Butantan	Antonio Jorge Martins; Claudia Renata dos Santos Barros; David Schlesinger; Debora Botequio Moretti; Dimas Tadeu Covas; Elaine Cristina Marqueze; Elaine Vieira Santos; Evandra Strazza Rodrigues; Heidge Fukumasu; Jayme Augusto de Souza-Neto; José Salvatore Leister Patané; Luiz Alcantara; Luiz Lehmann Coutinho; Maria Carolina Elias; Maurício Lacerda Nogueira; Rafael dos Santos Bezerra; Raul Machado Neto; Rejane Maria Tommasini Grotto; Ricardo Haddad; Sandra Coccuzzo Sampaio Vessoni; Simone Kashima; Svetoslav Nanev Slavov; Vincent Louis Viala
EPI_ISL_2878465	SELAS MEDILYS	Department of Virology, Henri Mondor University Hospital, Assistance Publique Hôpitaux de Paris, Université Paris-Est Créteil, INSERM U955	Alexandre Soulier; Christophe Rodriguez; Elisabeth Trawinski; Guillaume Gricourt; Jean-Michel Pawlotsky; Melissa N'Debi; Slim Fourati; Vanessa Demontant
EPI_ISL_2895385, EPI_ISL_2895396, EPI_ISL_2895404, EPI_ISL_2895415	SESARAM	Instituto Nacional de Saude (INSA)	Borges et al
EPI_ISL_2600810	SIESP CHIETI - DRIVE IN LANCIANO	Istituto Zooprofilattico Sperimentale dell'Abruzzo e Molise "G. Caporale"	Ancora M; Calistri P; Cammà C; Caporale M; Curini V; Delli Compagni E; Di Domenico M; Di Lollo Valeria; Di Pasquale A; Lorusso A; Mangone I; Marcacci M; Puglia I; Rinaldi A; Savini G; Scialabba S
EPI_ISL_2483577	SIESP DIPARTIMENTO DI PREVENZIONE TERAMO	Istituto Zooprofilattico Sperimentale dell'Abruzzo e Molise "G.	Ancora M; Calistri P; Cammà C; Caporale M; Curini V; Delli Compagni E; Di Domenico M; Di Lollo Valeria; Di Pasquale A; Lorusso A; Mangone I; Marcacci M; Puglia I; Rinaldi A; Savini G; Scialabba S

		Caporale”	
EPI_ISL_2828709	SISP ASL BAT	University of Bari Biomedical Sciences and Human Oncology	Maria Chironna
EPI_ISL_2562988, EPI_ISL_2564146, EPI_ISL_2565189, EPI_ISL_2587465, EPI_ISL_2587504, EPI_ISL_2883377, EPI_ISL_2889715, EPI_ISL_2889739			
see above	SK-Roy Romanow Provincial Laboratory	National Microbiology Laboratory (NML)	Alanna Senecal; Amanda Lang; Anna Majer; Anneliese Landgraff; CanCOGeN's metadata curation team; Darian Hole; Elsie Grudeski; Gary Van Domselaar; Grace Seo; Jennifer Tanner; Jessica Minion; Kara Loos; Keith MacKenzie; Kirsten Biggar; Madison Chapel; Meredith Faires; Morag Graham; Natalie Knox; Nathalie Bastien; Philip Mabon; Public Health Agency of Canada CanCOGeN team; Rachel DePaulo; Rhiannon Huzarewich; Russell Mandes; Ryan McDonald; Shari Tyson; Timothy Booth; Yan Li
EPI_ISL_1660718, EPI_ISL_1708577, EPI_ISL_2139795	SYNLAB	GIGA Medical Genomics	Bouchra Boujemla; Cécile Meex; Keith Durkin; Maria Artesi; Marie-Pierre Hayette; Nathalie Renotte; Pierrette Melin; Raphaël Boreux; Sébastien Bontems; Vincent Bours
EPI_ISL_2642051, EPI_ISL_2642061, EPI_ISL_2642070, EPI_ISL_2642080, EPI_ISL_2642086, EPI_ISL_2642087, EPI_ISL_2642090, EPI_ISL_2642091, EPI_ISL_2642092, EPI_ISL_2642119, EPI_ISL_2642127, EPI_ISL_2642139, EPI_ISL_2642152, EPI_ISL_2642154, EPI_ISL_2642155, EPI_ISL_2642157, EPI_ISL_2642180, EPI_ISL_2642186, EPI_ISL_2642187, EPI_ISL_2642188, EPI_ISL_2642194, EPI_ISL_2642198, EPI_ISL_2642199, EPI_ISL_2642228, EPI_ISL_2642244, EPI_ISL_2642302, EPI_ISL_2642339, EPI_ISL_2642342, EPI_ISL_2642362, EPI_ISL_2642372, EPI_ISL_2642376, EPI_ISL_2642391, EPI_ISL_2642418, EPI_ISL_2642429, EPI_ISL_2642433, EPI_ISL_2642439, EPI_ISL_2642447, EPI_ISL_2642448, EPI_ISL_2642449, EPI_ISL_2642450, EPI_ISL_2642452, EPI_ISL_2642453, EPI_ISL_2642454, EPI_ISL_2642510, EPI_ISL_2642518, EPI_ISL_2642578, EPI_ISL_2642659, EPI_ISL_2642660, EPI_ISL_2642661, EPI_ISL_2642768, EPI_ISL_2642923, EPI_ISL_2642945, EPI_ISL_2642955, EPI_ISL_2642958, EPI_ISL_2642959, EPI_ISL_2642960, EPI_ISL_2642961, EPI_ISL_2642962, EPI_ISL_2642966, EPI_ISL_2642967, EPI_ISL_2642968, EPI_ISL_2643032, EPI_ISL_2643033, EPI_ISL_2643039, EPI_ISL_2643042, EPI_ISL_2643073, EPI_ISL_2643085, EPI_ISL_2643086, EPI_ISL_2643088, EPI_ISL_2643092, EPI_ISL_2643093, EPI_ISL_2643110, EPI_ISL_2643113, EPI_ISL_2643163, EPI_ISL_2643169, EPI_ISL_2643178, EPI_ISL_2643179, EPI_ISL_2643186, EPI_ISL_2643187, EPI_ISL_2643190, EPI_ISL_2643191, EPI_ISL_2643197, EPI_ISL_2643212, EPI_ISL_2643258, EPI_ISL_2643263, EPI_ISL_2643299, EPI_ISL_2643379, EPI_ISL_2643385, EPI_ISL_2643396, EPI_ISL_2643409, EPI_ISL_2643441, EPI_ISL_2643470, EPI_ISL_2643544, EPI_ISL_2643648, EPI_ISL_2643653			
see above	SYNLAB Eesti OÜ	Department of Microbiology, Institute of Biomedicine and Translational Medicine, University of Tartu	Aare Abroi; Andrio Lahesaare; Arina Shablinskaja; Dagmar Hoidmets; Ene-Ly Jõgeda; Eveli Kallas; Heiki Niglas; Irja Lutsar; Kai Truusalu; Kaisa Truus; Katrin Kaarna; Kristi Huik; Liidia Dotsenko; Lili Azin Milani; Mari-Anne Härma; Mats Hansen; Meri Pauskar; Olga Sadikova; Paul Naaber; Radko Avi; Taavi Päll; Tuuli Reisberg; Ulvi Gerst Talas
EPI_ISL_1145357	SYNLAB Jena Oncoscreen	Robert Koch Institute	
EPI_ISL_1357516	SYNLAB Labor München Zentrum LMZ	Robert Koch Institute	
EPI_ISL_2262015	SYNLAB MVZ Leinfelden-Echterdingen	Robert Koch Institute	
EPI_ISL_2113906, EPI_ISL_2114481	SYNLAB MVZ Leverkusen	Robert Koch Institute	
EPI_ISL_1845492	SYNLAB MVZ Trier	Robert Koch Institute	
EPI_ISL_2389790	SYNLAB MVZ Weiden	Robert Koch Institute	
EPI_ISL_419301, EPI_ISL_419302, EPI_ISL_419303, EPI_ISL_419304	Saitama Prefectural Institute of Public Health	Pathogen Genomics Center, National Institute of Infectious Diseases	Hajime Kamiya; Kentaro Itokawa; Makoto Kuroda; Masanori Hashino; Michiyo Shinohara; Motoi Suzuki; Rina Tanaka; Tsuyoshi Kishimoto; Tsuyoshi Sekizuka
EPI_ISL_684147	Sakai City Institute of Public Health	Pathogen Genomics Center, National Institute of Infectious Diseases	Kentaro Itokawa; Makoto Kuroda; Masanori Hashino; Rina Tanaka; Tsuyoshi Sekizuka
EPI_ISL_2105815, EPI_ISL_2319293, EPI_ISL_2692637, EPI_ISL_2810090, EPI_ISL_2810116, EPI_ISL_2978450, EPI_ISL_2978587			
see above	Salud Digna	Instituto Nacional de Medicina Genomica	Abraham Campos-Romero; Cedro-Tanda A; Escobar-Arrazola; Gonzalez-Barrera D; Herrera-Montalvo LA.; Hidalgo-Miranda A; Luna-Ruiz Marco; M; Mendoza-Vargas A; Moreno-Camacho José Luis; Munguia-Garza P; Ramirez-Vega O; Rangel-DeLeon D; Reyes-Grajeda JP; Rodriguez-Gallegos Jorge
EPI_ISL_636198, EPI_ISL_667046, EPI_ISL_730115	San Diego County Public Health Laboratory	Andersen lab at Scripps Research	Brett Austin; Jovan Shephard; SEARCH Alliance San Diego with Tracy Basler
EPI_ISL_749155, EPI_ISL_749706, EPI_ISL_750174, EPI_ISL_2754033, EPI_ISL_2754203	Sanatorio Americano	Institut Pasteur de Montevideo	Ana Carolina Mendonça; Andres Lizasoain; Andrés Lizasoain; Belén González; Camila Simoes; Cecilia Alonso; Cecilia Salazar; Daiana Mir; Emiliano Pereira; Fernando López-Tort; Fernando Motta; Gonzalo Bello; Ighor Arantes; Ignacio Ferrés; Jose Sotelo; Juan Zanetti; Leticia Maya; Letícia Garay Martins; Lucia Bilbao; Luciana Appolinario; Luciana Griffero; Lucía Spangenberg; Lucía Spangenberg; Mailen Arleo; Mariana Brandes; Marilda Mendonça Siqueira; Marilda Tereza Mar da Rosa; María José Benitez-Galeano; Martín Graña; María José Benítez-Galeano; Matías Castells; Matías Victoria; Matías Castells; Matías Salvo; Matías Victoria; Mauricio Méndez; Melissa Duquía; Natalia Rego; Natalia Reyes; Odhille Chappos; Pablo Smircich; Paola Cristina Resende; Pía Techera; Rodney Colina; Tamara Fernandez-Calero; Tamara Fernández-Calero; Tania Possi; Tatiana Schäffer Gregianini; Verónica Noya; Verónica Noya; Yasser Vega
EPI_ISL_2547347, EPI_ISL_2547348	Sanddrift nc Clinic	National Health Laboratory Service/University of Cape Town (NHLS/UCT)	Arash Iranzadeh; Bruna Galvao; Carolyn Williamson; Deelan Doolabh; Diana Hardie; Gert Marais; Innocent Mudau; Lynn Tyers; Marvin Hsiao; Stephen Korsman
EPI_ISL_479800	Sapporo City Institute of Public Health	Pathogen Genomics Center, National Institute of Infectious Diseases	Asami Ohnishi; Hajime Kamiya; Kentaro Itokawa; Makoto Kuroda; Masanori Hashino; Motoi Suzuki; Rina Tanaka; Tsuyoshi Sekizuka
EPI_ISL_1971032	Sapporo City Institute of Public Health	Sapporo City Institute of Public Health	Asami Ohnishi; Kentaro Itokawa; Makoto Kuroda; Masanori Hashino; Masayuki Kikuchi; Rie Shimazaki; Rina Tanaka; Tsuyoshi Sekizuka; Yusuke Oguchi
EPI_ISL_1009123	School of Pharmacy	School of Pharmacy	Ahmed Kandeil; Ghazi Kayali; Mina Nabil Kamel; Mohamed A Ali; Rabeh El-Shesheny; Walid Abi Habib
EPI_ISL_661185	Scientific Veterinary Institute Novi Sad	Veterinary Specialized Institute "Kraljevo", Serbia	Afonso, C.; Banovic Djeri, B.; Jankovic, M.; Jovanovic, T.; Knezevic, A.; Petrovic, T.; Sekler, M.; Tesovic, B.; Vidanovic, D.; Volkening, J.
EPI_ISL_408977	Serology, Virology and OTDS Laboratories (SAViD), NSW Health Pathology Randwick	NSW Health Pathology - Institute of Clinical Pathology and Medical Research; Centre for Infectious Diseases and Microbiology Laboratory Services; Westmead Hospital; University of Sydney	Carter I; Chen SC; Eden J-S; Holmes EC; Kok J and Dwyer DE for the 2019-nCoV Study Group*; Maddocks S; O'Sullivan MV; Rahman H; Rawlinson W; Rockett R; Sintchenko V
EPI_ISL_1754133, EPI_ISL_2179780	Servicio Microbiología Hospital La Paz	Servicio Microbiología Hospital La Paz	Elie Dahdouh; Fernando Lázaro; Jesús Mingorance Cruz; Rubén Cáceres
EPI_ISL_856766, EPI_ISL_856774, EPI_ISL_2105574, EPI_ISL_2135170, EPI_ISL_2135699, EPI_ISL_2136027, EPI_ISL_2136086, EPI_ISL_2136141, EPI_ISL_2136143, EPI_ISL_2140067, EPI_ISL_2140071, EPI_ISL_2158708, EPI_ISL_2158745, EPI_ISL_2158754, EPI_ISL_2158755, EPI_ISL_2158761, EPI_ISL_2158762, EPI_ISL_2158766, EPI_ISL_2158773, EPI_ISL_2158774, EPI_ISL_2158779, EPI_ISL_2158815, EPI_ISL_2158817, EPI_ISL_2158830, EPI_ISL_2158850			
see above	Servicio Virosis Respiratorias-Departamento Virología-INEI	Instituto Nacional Enfermedades Infecciosas C.G.Malbran	Avaro M.; Baumeister E.; Benedetti E.; Campos J.; Cisterna D.; Dattero ME; Lorenzo F.; Molina V.; Perandones C.; Poklepovich T.; Pontoriero A.; Russo M.; Tuduri E.
EPI_ISL_2516849	Servicio de Microbiología Clínica (Complejo Hospitalario de Navarra, Pamplona)	Centro de Secuenciación NASERTIC	Ana Miqueleiz; Ana Navascués; Carmen Ezpeleta Baquedano
EPI_ISL_691645, EPI_ISL_691646, EPI_ISL_691648	Servicio de Microbiología, Hospital Universitario Son Espases	SeqCOVID-SPAIN consortium/IBV(CSIC)	Antonio Oliver and SeqCOVID-SPAIN consortium; Carla López-Causapé; Jordi Reina
EPI_ISL_419687	Servicio de Microbiología. Consorcio Hospital General Universitario de Valencia	Sequencing and Bioinformatics Service and Molecular Epidemiology Research Group. FISABIO-Public Health	Concepcion Gimeno; Fernando Gonzalez-Candelas; Giuseppe D'Auria; Griselda De Marco; Maria Alma Bracho; Maria Dolores Ocete; Neris Garcia-Gonzalez
EPI_ISL_2776001	Servicio de Microbiología. Hospital General Universitario de Castellón	SeqCOVID-SPAIN consortium/IBV(CSIC)	María Dolores Tirado Balaguer and SeqCOVID-SPAIN consortium; Rosario Moreno Muñoz
EPI_ISL_509615	Servicio de Microbiología. Hospital Universitario Donostia. OSI Donostialdea. Área de Enfermedades Infecciosas, Grupo de Infección Respiratoria y Resistencia Antimicrobiana. Instituto de Investigación Sanitaria Biodonostia	SeqCOVID-SPAIN consortium/IBV(CSIC)	Gustavo Cilla; Jose Maria Marimón and SeqCOVID-SPAIN consortium; Luis Piñeiro; Milagrosa Montes
EPI_ISL_452641	Servicio de Microbiología. Hospital Universitario Donostia. OSI Donostialdea. Área de Enfermedades Infecciosas, Grupo de Infección Respiratoria y Resistencia Antimicrobiana. Instituto de Investigación Sanitaria Biodonostia.	SeqCOVID-SPAIN consortium/IBV(CSIC)	Gustavo Cilla; Jose Maria Marimón and SeqCOVID-SPAIN consortium; Luis Piñeiro; Milagrosa Montes
EPI_ISL_710547	Sestre Milosrdnice University Hospital Center	Ruđer Boškovic Institute; Forensic Science Centre Ivan Vučetić; University of Zagreb Faculty of Science	Ana Livun; Antonela Blažeković; Boris Maček; Danilo Licastro; Dunja Glavaš; Fran Borovečki; Gordana Maravić Vlahoviček; Ivan Šamija; Ivana Ćelap; Josipa Skelin; Kristian Vlahoviček; Kristina Gotovac Jerečić; Lidija Cvetko-Krajinović; Lucija Markulin; Maja Kuzman; Marina Korolija; Mario Štefanović; Mirjana Domazet-Lošo; Paula Štancl; Robert Belužić; Rosa Karlić; Sanja Tadinac; Tomislav Domazet-Lošo; Vjekoslav Tomaić; Vladimir Krajinović; Željka Maćak Šafranko
EPI_ISL_2545290, EPI_ISL_2834380, EPI_ISL_2834393, EPI_ISL_2834506, EPI_ISL_3019862, EPI_ISL_3022075, EPI_ISL_3022076			
see above	Shamir Medical Center (Asaf Harofe)	Shamir Medical Center (Asaf Harofe)	Abu Hamad Ramzia; Adina Bar Chaim; Anna Vishnevsky; Chen Weiner; Netta Zuckerman; Nir Rainy; Patricia Benveniste-Lekovitz; Reut Sorek Abramovich; Yevgeni Yegorov
EPI_ISL_582125, EPI_ISL_582126	Sheikh Khalifa Medical City	Molecular Surveillance lab Sheikh Khalifa Medical City	Amirtharaj Francis; Hala Imambaccus; Hiba Saud; Sahar Almarzooqi; Sajeed Abdul; Stefan Weber
EPI_ISL_582611, EPI_ISL_582613, EPI_ISL_582617, EPI_ISL_582646, EPI_ISL_582648, EPI_ISL_582677, EPI_ISL_582679, EPI_ISL_582683			
see above	Sheikh Khalifa Medical City	Molecular/Surveillance lab Sheikh Khalifa Medical City	Amirtharaj Francis; Hala Imambaccus; Hiba Saud; Sahar Almarzooqi; Sajeed Abdul; Stefan Weber
EPI_ISL_2500849, EPI_ISL_2921027	Shenzhen Center for Disease Control and Prevention	National Institute for Viral Disease Control and Prevention, China CDC	Can Zhu; Jiancheng Chen; Kai Nie; Long Chen; Peihua Niu; Renli Zhang; Shaoyu Deng; Shaoyu Deng and Yaqing He; Weihua Wu; Xiang Zhao; Xiaoliang Xiao; Xinyi Wei; Yaqing He; Yenan Feng; Ying Sun; Yue Li
EPI_ISL_2086220, EPI_ISL_2086224, EPI_ISL_2086226	ShipMed Inc	KRISP, KZN Research Innovation and Sequencing Platform	Emmanuel SJ; Giandhari J; Lessells R; Naidoo Y; Pillay S; Ramphal U; Tegally H; Wilkinson E; de Oliveira T
EPI_ISL_2608422	Siem Reap Provinical Laboratory	Virology Unit, Institut Pasteur du Cambodge	Cecile Troupin; Chau Darapheak; Chin Savuth; Erik A Karlsson; Jurre Y Siegers; Kraing Sidonn; Leakhena Pum; Ly Sovann; Veasna Duong; Yi Sengdoeurn
EPI_ISL_500570, EPI_ISL_610154	Singapore General Hospital	Department of Microbiology	Chenhao Li; Chenhao Li; Karrie Ko; Karrie Ko; Kenneth Xin Long Chan; Kern Rei Chng; Kern Rei Chng; Kian Sing Chan; Kian Sing Chan; Kun Lee Lim; Kun Lee Lim; Lynette Oon; Lynette Oon; Niranjan Nagarajan; Niranjan Nagarajan; Nurdyana Abdul Rahman; Nurdyana Abdul Rahman; Sui Sin Goh
EPI_ISL_2470333	Sonic - Labor Dr. von Froreich GmbH	Robert Koch Institute	
EPI_ISL_1569062	Sonic - MVZ Medizinisches Labor Bremen GmbH	Robert Koch Institute	

EPI_ISL_2006881, EPI_ISL_2006899, EPI_ISL_2427243, EPI_ISL_2675217, EPI_ISL_3047978	South Dakota Public Health Laboratory	South Dakota Public Health Laboratory	Chris Carlson; Jacob Garfin and Chris Carlson
EPI_ISL_513377, EPI_ISL_593699, EPI_ISL_593726, EPI_ISL_629030, EPI_ISL_740868, EPI_ISL_768602, EPI_ISL_1904462, EPI_ISL_1904464, EPI_ISL_2462416, EPI_ISL_2462422, EPI_ISL_2650459, EPI_ISL_2650461			
see above	South Eastern Area Laboratory Services (SEALS)	NSW Health Pathology - Institute of Clinical Pathology and Medical Research; Westmead Hospital; University of Sydney	CIDM-PH et al.
EPI_ISL_2928014, EPI_ISL_2928016, EPI_ISL_2928017, EPI_ISL_2928018, EPI_ISL_2928019, EPI_ISL_2928020, EPI_ISL_2928021, EPI_ISL_2928023, EPI_ISL_2928025, EPI_ISL_2928026, EPI_ISL_2928027			
see above	South Sudan Ministry of Health, WHO South Sudan, MRC/UVRI & LSHTM Uganda Research Unit	MRC/UVRI & LSHTM Uganda Research Unit, South Sudan Ministry of Health, WHO South Sudan	Abe G. Abias; Dan Lule Bugembe; Dennis Kenyi Lodiongo; James Ayei; John Rumunu; Joseph Francis Wamala; Juma John HM; Lul Lojok Deng; Matthew Cotten; My V.T. Phan; Pontiano Kaleebu; Richard Lino Loro Lako; Sudhir Bunga
EPI_ISL_456232, EPI_ISL_456238, EPI_ISL_456297, EPI_ISL_579133	Southern Community Labs Dunedin	Institute of Environmental Science and Research (ESR)	Anja Werno; Antje van der Linden; Arlo Upton; Chris Mansell; David Hammer; Dragana Drinkovic; Erasmus Smit; Gary McAuliffe; Hana Sofia Andersson; Hermes Perez; James Ussher; Jill Sherwood; Jing Wang; Joep de Ligt; Josh Freeman; Julia Howard; Juliet Elvy; Lauren Jelly; Mary DeAlmeida; Matt Blakiston; Matt Storey; Matthew Rogers; Max Bloomfield; Michael Addidle; Michelle Balm; Muhammad Faisal; Nikki Freed; Olin Silander; Sally Roberts; Sarah Jefferies; Sharmini Muttaiyah; Susan Morpeth; Susan Taylor; Timothy Blackmore; Vani Sathyendran; Veronica Playle; Virginia Hope; Xiaoyun Ren
EPI_ISL_849739, EPI_ISL_849743	Special Operations Medical Research Division, Defence Services Medical Research Centre	Special Operations Medical Research Division, Defence Services Medical Research Centre	Aung; Htun; K.K.; K.Z.; Lwin; Myint, K.; N.M.; Oo; P.K.; Win; Z.W.; Zaw, T.
EPI_ISL_513399	St Vincent's Pathology (SydPath)	NSW Health Pathology - Institute of Clinical Pathology and Medical Research; Westmead Hospital; University of Sydney	CIDM-PH et al.
EPI_ISL_596672	St.Vincent's University Hospital	St.Vincent's University Hospital	Aisling Purcell; Gabriel Gonzalez; Guerrino Macori; Kirsten Schaffer; Lynda Fenelon; Mary Lucey; Niamh Mullane; Suzie Coughlan; Séamus Fanning; Una Sutton-Fitzpatrick
EPI_ISL_1495136	State Institution «Public Health Center of Ministry of Health of Ukraine»	Robert Koch Institute, ZBS1 Highly Pathogenic Viruses, Berlin, Germany	Andreas Nitsche; Annika Brinkmann; Iryna Demchyshyna; Janine Michel; Liudmyla Chernenko; Roman Rodyna; Steven Uddin
EPI_ISL_2779638, EPI_ISL_2779639	State Key Laboratory of Emerging Infectious Diseases, The University of Hong Kong	The University of Hong Kong	Huachen Zhu; Lifeng Li; Xianfan Wu; Yi Guan; Yiu Man Cheung
EPI_ISL_2662727	State Key Laboratory of Pathogen and Biosecurity, Beijing Institute of Microbiology and Epidemiology	State Key Laboratory of Pathogen and Biosecurity, Beijing Institute of Microbiology and Epidemiology	Campbell, A.; Campbell, J.; Gevao; Harding, D.; Lin, L.; P.F.; P.S.; Rogers, J.; Sahr; Wurie, I.; Zhang, J.
EPI_ISL_752613, EPI_ISL_753087, EPI_ISL_753170, EPI_ISL_1771478, EPI_ISL_2007889, EPI_ISL_2724554, EPI_ISL_2724734, EPI_ISL_3033249			
see above	State Laboratories Division, Hawaii State Department of Health	State Laboratories Division, Hawaii State Department of Health	Ayana Garnet; Drew Kuwazaki; Edward Desmond; Pamela O'Brien; Razvan Sultana; Sabrina Diemert
EPI_ISL_2378030, EPI_ISL_2378031, EPI_ISL_2378033, EPI_ISL_2378143, EPI_ISL_2378146, EPI_ISL_2378147, EPI_ISL_2800610			
see above	State Testing Facility	Altius Institute for Biomedical Research	Alex Isner; Alex Nguyen; Amanda Gale; Audra Johnson; Clem Green; Daniel Bates; Eric Thorland; Jacob Rodriguez; Jean Robinston; Jemma Nelson; Jessica Kunder; John Stamatoyannopoulos; Joshua Richards; Julia Wald; Kneshay Harper; Lauren Mitchell; Mark Frerker; Matt Hartman; Michael Buckley; Muhammad Halimun; Rebecca Bruders; Sadie Patraw; Sofia Olsson; Tobias Ragoczy
EPI_ISL_2685046	Statens Serum Institut Bioinformatics and Microbial Genomics	Statens Serum Institut Bioinformatics and Microbial Genomics	Danish Covid-19 Genome Consortium
EPI_ISL_2375915	Steinkopf PHC Clinic	NHLS/UCT	Arash Iranzadeh; Bruna Galvao; Carolyn Williamson; Deelan Doolabh; Diana Hardie; Innocent Mudau; Kruger Marais; Lynn Tyers; Marvin Hsiao; Stephen Korsman
EPI_ISL_640079, EPI_ISL_2375919	Stellenbosch Hospital wc STB	NHLS/UCT	Arash Iranzadeh; Bruna Galvao; Carolyn Williamson; Deelan Doolabh; Diana Hardie; Innocent Mudau; Kruger Marais; Lynn Tyers; Marvin Hsiao; Stephen Korsman
EPI_ISL_2921612	Sultan Haji Ahmad Shah Hospital	Institute for Medical Research, Infectious Disease Research Centre, National Institutes of Health, Ministry of Health Malaysia	Anasir MI; Azizan MA; Kamel K; Mohd Zawawi Z; Ramly N; Robert F; Suppiah J; Thayan R
EPI_ISL_2931923	Sultanah Fatimah Specialist Hospital, Muar	Institute for Medical Research, Infectious Disease Research Centre, National Institutes of Health, Ministry of Health Malaysia	Anasir MI; Azizan MA; Kamel K; Mohd Zawawi Z; Ramly N; Robert F; Suppiah J; Thayan R
EPI_ISL_2811879	Sultanah Nora Ismail Hospital, Batu Pahat	Institute for Medical Research, Infectious Disease Research Centre, National Institutes of Health, Ministry of Health Malaysia	Azizan MA; Kamel K; Mohd Zawawi Z; Ramly N; Robert F; Suppiah J; Thayan R
EPI_ISL_450747	Sunnybrook Health Sciences Centre	Department of Laboratory Medicine and Molecular Diagnostics, Sunnybrook Health Sciences Centre	Amogelang R. Raphenya; Andrew G. McArthur; Arinjay Banerjee; Bo Wang; Brian P. Alcock; Finlay Maguire; Hamza Mbareche; Hassaan Maan; Jalees A. Nasir; Julian A. Hiscox; Karen Mossman; Kendrick M. Smith; Muhannad Alruwaili; Natalie C. Knox; Patryk Aftanas; Robert A. Kozak; Samira Mubareka
EPI_ISL_2406466	Svay Rieng Rapid Response Team	Virology Unit, Institut Pasteur du Cambodge	Cecile Troupin; Chau Darapheak; Chin Savuth; Erik A Karlsson; Jurre Y Siegers; Kraing Sidonn; Leakhena Pum; Ly Sovann; Veasna Duong; Yi Sengdoeurn
EPI_ISL_1193078, EPI_ISL_1617530, EPI_ISL_1618454, EPI_ISL_1676225, EPI_ISL_1807444, EPI_ISL_1898458, EPI_ISL_1899265, EPI_ISL_1899290, EPI_ISL_1900277, EPI_ISL_2033384, EPI_ISL_2033792, EPI_ISL_2099115, EPI_ISL_2278930, EPI_ISL_2410253, EPI_ISL_2414900, EPI_ISL_2415990, EPI_ISL_2417386, EPI_ISL_2419171, EPI_ISL_2420110, EPI_ISL_2613350, EPI_ISL_2792809, EPI_ISL_2792949, EPI_ISL_2793678, EPI_ISL_2794645, EPI_ISL_2889039, EPI_ISL_2980627			
see above	Swedish national genomic surveillance program of SARS-CoV-2	The Public Health Agency of Sweden	Alma Brolund; Maria Lind Karlberg; Maximilian Riess; Swedish national genomic surveillance program of SARS-CoV-2
EPI_ISL_860745	Swiss National Reference Centre for Influenza	Swiss National Reference Centre for Influenza	Ana Rita Gonçalves Cabecinhas; Christoph Grünig; Florian Laubscher; Laurent Kaiser; Samuel Cordey
EPI_ISL_478718	Sydney South West Pathology Service (SSWPS) - Concord Repatriation General Hospital - NSW Health Pathology	NSW Health Pathology - Institute of Clinical Pathology and Medical Research; Westmead Hospital; University of Sydney	CIDM-PH et al.
EPI_ISL_2811986, EPI_ISL_2812836, EPI_ISL_2812857	Sydney South West Pathology Service (SSWPS) - Liverpool Hospital - NSW Health Pathology	NSW Health Pathology - Institute of Clinical Pathology and Medical Research; Westmead Hospital; University of Sydney	CIDM-PH et al.
EPI_ISL_767863, EPI_ISL_803111, EPI_ISL_2462428	Sydney South West Pathology Service (SSWPS) - Royal Prince Alfred Hospital - NSW Health Pathology	NSW Health Pathology - Institute of Clinical Pathology and Medical Research; Westmead Hospital; University of Sydney	CIDM-PH et al.
EPI_ISL_1319275, EPI_ISL_2107235, EPI_ISL_2107284, EPI_ISL_2546950, EPI_ISL_2547057, EPI_ISL_2788795, EPI_ISL_2788800, EPI_ISL_2788837			
see above	Synlab Eesti OÜ	1. Laboratory of Communicable Diseases (Estonia); 2. Eurofins Genomics Europe Sequencing GmbH	Liidia Dotsenko et al.
EPI_ISL_1655749, EPI_ISL_1655750, EPI_ISL_1656735	Synlab Haut de France	UMR 8199/1283 EGID	Derhourhi Mehdi; Mehdi Derhourhi
EPI_ISL_2897132	Szpital Uniwersytecki w Krakowie	1. Tricity SARS-CoV-2 sequencing consortium: University of Gdansk, Medical University of Gdansk, Vaxican Ltd., Invicta Ltd. 2. National Institute of Public Health - National Institute of Hygiene, Warsaw, Poland	Celina Cybulska; Karolina Gackowska; Katarzyna Groth; Katarzyna Zacharczuk; Krystyna Bienkowska Szewczyk; Lukasz Rabalski; Maciej Grzybek; Maciej Kosinski; Magdalena Nowakowska; Marcin Lubocki; Małgorzata Sadkowska-Todys; Tomasz Wołkowicz
EPI_ISL_2694620, EPI_ISL_2694702, EPI_ISL_2695504, EPI_ISL_2695517	TAMBO MEMORIAL	National Institute for Communicable Diseases of the National Health Laboratory Service	Amoako DG; Bhiman JN; Everatt J; Ismail A; Mahlangu B; Mnguni A; Mohale T; Ntuli N; Scheepers C
EPI_ISL_2662568, EPI_ISL_2662572, EPI_ISL_2662574, EPI_ISL_2709951, EPI_ISL_2709967, EPI_ISL_2709989, EPI_ISL_2827057, EPI_ISL_3024631			
see above	TAMBO MEMORIAL LABORATORY	National Institute for Communicable Diseases of the National Health Laboratory Service	Amoako DG; Bhiman JN; Everatt J; Ismail A; Mahlangu B; Mnguni A; Mohale T; Ntuli N; Scheepers C
EPI_ISL_914824	TAMIZAJE COMUNITARIO - PASO CANOAS	Inciensa, Instituto Costarricense de Investigación y Enseñanza en Nutrición y Salud	Adriana Godínez; Claudio Soto-Garita; Estela Cordero; Francisco Duarte; Hebleen Porras; Melany Calderón & Mariel López
EPI_ISL_1530791	TGen North	TGen North	Ashlyn Pfeiffer; Chris French; Darrin Lemmer; Dave Engelthaler; Hayley Yaglom; Heather Centner; Jolene Bowers; The Arizona COVID Genomics Union (ACGU)
EPI_ISL_426629, EPI_ISL_436105, EPI_ISL_447252, EPI_ISL_447593, EPI_ISL_457726, EPI_ISL_2693000, EPI_ISL_2693004, EPI_ISL_2693006			
see above	TSGH-CP molecular lab	TSGH-CP molecular lab	Cherng-Lih Perng; Chien-Wen Chen; Chih-Kai Chang; Feng-Yee Chang; Hsing-Yi Chung; Hung-Sheng Shang; Jung-Chung Lin; Kuo-Ming Yeh; Kuo-Sheng Hung; Ming-Jr JIAN; Ming-Jr Jian; Sheng-Kang Chiu; Shih-Hung Tsai; Tien-Yao Chang
EPI_ISL_2987237, EPI_ISL_2987242, EPI_ISL_2987249, EPI_ISL_2987322, EPI_ISL_2987328, EPI_ISL_2987337, EPI_ISL_2987343, EPI_ISL_2987344			
see above	TSHEPONG LABORATORY	National Institute for Communicable Diseases of the National Health Laboratory Service	Amoako DG; Bhiman JN; Everatt J; Ismail A; Mahlangu B; Mnguni A; Mohale T; Ntuli N; Scheepers C
EPI_ISL_2827906, EPI_ISL_2827908, EPI_ISL_2827911, EPI_ISL_2984879, EPI_ISL_2984882, EPI_ISL_2984913, EPI_ISL_2984921, EPI_ISL_2984926, EPI_ISL_2984930, EPI_ISL_2984938			
see above	TSHEPONG LABORATORY	National Institute for Communicable Diseases of the National Health Laboratory Service	Amoako DG; Bhiman JN; Everatt J; Ismail A; Mahlangu B; Mnguni A; Mohale T; Ntuli N; Scheepers C
EPI_ISL_2506492,	TYKS, Kliininen mikrobiologia	Expert Microbiology, National Institute for Health and Welfare	Carita Savolainen-Kopra; Erika Lindh; Haider al-Hello; Jani Halkilahti; Kirsi Liitsola; Niina Ikonen; Olli Vapalahti; Pekka Ellonen; Phuoc Truong; Päivi Laurila; Ravi Kant; Sari Hannula; Soile Blomqvist; Teemu Smura

EPI_ISL_2590709			
EPI_ISL_1972539	Tampin Hospital	Institute for Medical Research, Infectious Disease Research Centre, National Institutes of Health, Ministry of Health Malaysia	Azizan MA; Kamel K; Mohd Zawawi Z; Ramly N; Robert F; Suppiah J; Thayan R
EPI_ISL_911750	Tanjungpura University Hospital	Tanjungpura University Hospital	Ambar Rialita; Andriani; Delima Fajar Liana; Eka Ardiani Putri; Helmi Sastriawan; Mahyarudin; Muhammad Kahtan; Puji Astuti; Rini Andriani; Sofi Siti Shofiyah; Virhan Novianry; Wiwik Windarti
EPI_ISL_2611673, EPI_ISL_2651121, EPI_ISL_3019481, EPI_ISL_3019485	Temporary Specimen Collection Centre at the AsiaWorld-Expo	Hong Kong Department of Health	Alan K.L. Tsang; Dominic N.C. Tsang; Edman T.K. Lam; Ken H.L. Ng; Peter C.W. Yip; Rickjason C.W. Chan
EPI_ISL_934972	Thai Red Cross Emerging Infectious Diseases Health Science Centre, Chulalongkorn Hospital, Faculty of Medicine, Chulalongkorn University	Thai Red Cross Emerging Infectious Diseases Center and Faculty of Medicine, Chulalongkorn University	Apaporn Rodpan; Opass Putcharoen; Rome Buathong; Sininat Petcharat; Sopon Iamsirithaworn; Supaporn Wacharapluesadee; Thiravat Hemachudha; Weenassarin Ampoot; Yutthana Joyjinda
EPI_ISL_1490226, EPI_ISL_2230689, EPI_ISL_2230690, EPI_ISL_2230691, EPI_ISL_2230692, EPI_ISL_2230696, EPI_ISL_2230698, EPI_ISL_2230699, EPI_ISL_2478969, EPI_ISL_2500850, EPI_ISL_2544758, EPI_ISL_2545191, EPI_ISL_2545192, EPI_ISL_2545194, EPI_ISL_2545198			
see above	The Caribbean Public Health Agency	Carrington Lab, Department of PreClinical Sciences, Faculty of Medical Sciences, The University of the West Indies	Adesh Ramsubhag; Anushka Ramjag; Arianne Brown-Jordan; Avery Hinds; Ayoola Oyinloye; Chinna Chinnadurai; Christine V. F. Carrington; Christopher Oura; Gabriel Escobar; Jaya Jayaraman; Jerome Foster; Karla Georges; Kenneth George; Marsha Ivey; Naresh Nandram; Narine Singh; Nikita S. D. Sahadeo; Nuno Faria; Oliver Pybus; Rahul Naidu; Rajini Haraksingh; Risha Singh; Roshan Parasram; Sarah Hill; Shawn Charles; Stanley Giddings; SueMin Nathaniel; Vernie Ramkissoon
EPI_ISL_2631411	The Caribbean Public Health Agency	Carrington Lab, Department of PreClinical Sciences, Faculty of Medical Sciences, The University of the West Indies, St Augustine Campus	Anushka Ramjag; Arianne Brown-Jordan; Avery Hinds; Christine V. F. Carrington; Christopher Oura; Gabriel Escobar; Karla Georges; Naresh Nandram; Nikita S. D. Sahadeo; Nuno Faria; Oliver Pybus; Risha Singh; Roshan Parasram; Sarah Hill; SueMin Nathaniel; Vernie Ramkissoon
EPI_ISL_2557166, EPI_ISL_2565080, EPI_ISL_2621679, EPI_ISL_2621681, EPI_ISL_2621714, EPI_ISL_2621722, EPI_ISL_2626658, EPI_ISL_2626661, EPI_ISL_2626663			
see above	The Caribbean Public Health Agency	Carrington Lab, Department of Preclinical Sciences, Faculty of Medical Sciences, The University of the West Indies	Anushka Ramjag; Arianne Brown-Jordan; Avery Hinds; Christine V. F. Carrington; Christopher Oura; Gabriel Escobar; Jacquiline Bisesor-McKenzie; Karla Georges; Kenneth George; Naresh Nandram; Nikita S. D. Sahadeo; Nuno Faria; Oliver Pybus; Risha Singh; Roshan Parasram; Sarah Hill; Sharon Belmar-George; SueMin Nathaniel; Vernie Ramkissoon
EPI_ISL_2649790, EPI_ISL_2658366, EPI_ISL_2658367, EPI_ISL_2658376, EPI_ISL_2658405, EPI_ISL_2678129, EPI_ISL_2678143, EPI_ISL_2678145, EPI_ISL_2678146, EPI_ISL_2678161, EPI_ISL_2678163, EPI_ISL_2678165, EPI_ISL_2678166, EPI_ISL_2678168, EPI_ISL_2678169, EPI_ISL_2678171, EPI_ISL_2678172, EPI_ISL_2678180, EPI_ISL_2716565, EPI_ISL_2716569, EPI_ISL_2716570, EPI_ISL_2716574, EPI_ISL_2716576, EPI_ISL_2716577, EPI_ISL_2716580, EPI_ISL_2716583, EPI_ISL_2716594, EPI_ISL_2716595, EPI_ISL_2716602, EPI_ISL_2756569, EPI_ISL_2756571, EPI_ISL_2756578, EPI_ISL_2756579, EPI_ISL_2756580, EPI_ISL_2756581, EPI_ISL_2756582, EPI_ISL_2756585, EPI_ISL_2955588, EPI_ISL_2955590, EPI_ISL_2967979, EPI_ISL_2967980, EPI_ISL_2967981, EPI_ISL_2967982, EPI_ISL_2967983, EPI_ISL_2967984, EPI_ISL_2967985, EPI_ISL_2967986, EPI_ISL_2967991, EPI_ISL_2967992, EPI_ISL_2967993			
see above	The Caribbean Public Health Agency	Carrington Lab, Department of Preclinical Sciences, Faculty of Medical Sciences, The University of the West Indies, St Augustine Campus	Anushka Ramjag; Arianne Brown-Jordan; Avery Hinds; Christine V. F. Carrington; Christopher Oura; Gabriel Escobar; Hazel Laws; Karla Georges; Kenneth George; Naresh Nandram; Nikita S. D. Sahadeo; Nuno Faria; Oliver Pybus; Rhonda Sealey-Thomas; Risha Singh; Roshan Parasram; Sarah Hill; Sharon Belmar-George; Shawn Charles; Simone Keizer-Beache; SueMin Nathaniel; Vernie Ramkissoon
EPI_ISL_2860169	The Medical City	Philippine Genome Center	Alethea R. de Guzman; Anna Ong-Lim; Arianne A. Zamora; Benedict A. Maralit; Carlo M. Lapid; Celia Carlos; Devon Ray Pacial; Diomedes A. Carino; Edsel Maurice Salvaña; El King D. Morado; Elcid Aaron R. Pangilinan; Eva Maria Cutiongco-de la Paz; Francis A. Tablizo; Henrietta Marie Rodriguez; Jaime C. Montoya; Jan Michael C. Yap; Jarvin E. Nipales; Jo-Hannah S. Llames; John Q. Wong; Joshua Gregor A. Dizon; Juan Antonio R. Magalang; Karol Sophia Agape R. Padilla; Kenneth M. Kim; Kris P. Punayan; Krisitna Patriz Dela Cruz; Lindsay Claire D.L. Carandang; Ma. Exanil Plantig; Marc Edsel C. Ayes; Maria Rosario Singh-Vergeire and Cynthia P. Saloma; Maria Sofia L. Yangzon; Marielle M Gamboa; Marissa Alejandria; Nina Francesca Bustamante; Razel Nikka M. Hao; Renato Jacinto Q. Mantaring; Rianna Patricia S. Cruz; Sheila Mae M. Araiza; Yvonne Valerie Austria; Zipporah Mariebelle R. Enriquez; Zyrel V. Mollejon
EPI_ISL_577632, EPI_ISL_577633, EPI_ISL_792690, EPI_ISL_792699, EPI_ISL_850688	The National Institute of Public Health	State Veterinary Institute Prague	A; D; H; J; Jirincova; L; Nagy; Novakova; Trnka; Vecerova
EPI_ISL_489708	The National Institute of Public Health	The National Institute of Public Health and State Veterinary Institute Prague	A; D; H; J; Jirincova; L; Nagy; Novakova; Trnka; Vecerova
EPI_ISL_424583, EPI_ISL_1586190, EPI_ISL_1586350, EPI_ISL_1586370, EPI_ISL_1586520	The National University Hospital of Iceland	deCODE genetics	Agnar Helgason; Alma Moller; Arna B Agustsdottir; Arnaldur Gylfason; Asgeir Sigurdsson; Aslaug Jonasdottir; Berglind Eiriksdottir; Bjarni Thorbjornsson; Brynjar O Jensson; Daniel F Gudbjartsson; Droplaug N Magnusdottir; Elisabet E Gardarsdottir; Emil A Thorarensen; Gardar Sveinbjornsson; Gisli Masson; Gudmundur Georgsson; Gudmundur L Norddahl; Gudrun Sigmundsdottir; Hakon Jonsson; Hannes Eggertsson; Hilma Holm; Ingileif Jonsdottir; Jona Saemundsdottir; Kamilla S Josefsdottir; Kari Stefansson; Karl G Kristinsson; Kjartan R Gudmundsson; Kristin E Sveinsdottir; Kristin E Sveinsdottir; Louise le Roux; Maney Sveinsdottir; Olafia S Gretarsdottir; Olafia S Gretarsdottir; Olafur T Magnusson; Pall Melsted; Patrick Sulem; Run Fridriksdottir; Solvi Rognvaldsson; Thora R Gunnarsdottir; Thordur Kristjansson; Thorolfur Gudnason; Unnur Thorsteinsdottir
EPI_ISL_648135	The Public Health Agency of Sweden	The Public Health Agency of Sweden	Anna Risberg; Anna-Malin Linde; Karin Tegmark-Wisell; Maria Lind Karlberg; Mattias Haukland; Mia Brytting; Olov Svartstrom; Oskar Karlsson Lindsjo; Petra Edquist; Reza Advani; Sandra Broddesson
EPI_ISL_754229	The Republican Research and Practical Center for Epidemiology and Microbiology (RRPCEM)	WHO National Influenza Centre Russian Federation	Anatoly Krasko; Andrey Komissarov; Anna Ivanova; Artem Fadeev; Daria Danilenko; Dmitry Bazhenov; Dmitry Lioznov; Elena Gasich; Elena Nabieva; Georgii Bazykin; Kirill Bulda; Ksenia Safina; Kseniya Komissarova
EPI_ISL_2631429	The Trinidad and Tobago Public Health Laboratory	Carrington Lab, Department of PreClinical Sciences, Faculty of Medical Sciences, The University of the West Indies, St Augustine Campus	Anushka Ramjag; Arianne Brown-Jordan; Avery Hinds; Christine V. F. Carrington; Christopher Oura; Gabriel Escobar; Karla Georges; Naresh Nandram; Nikita S. D. Sahadeo; Nuno Faria; Oliver Pybus; Risha Singh; Roshan Parasram; Sarah Hill; SueMin Nathaniel; Vernie Ramkissoon
EPI_ISL_2658353	The Trinidad and Tobago Public Health Laboratory	Carrington Lab, Department of Preclinical Sciences, Faculty of Medical Sciences, The University of the West Indies, St Augustine Campus	Anushka Ramjag; Arianne Brown-Jordan; Avery Hinds; Christine V. F. Carrington; Christopher Oura; Gabriel Escobar; Karla Georges; Naresh Nandram; Nikita S. D. Sahadeo; Nuno Faria; Oliver Pybus; Risha Singh; Roshan Parasram; Sarah Hill; SueMin Nathaniel; Vernie Ramkissoon
EPI_ISL_2876985	Thembalethu CDC wc THC	NHLS/UCT	Arash Iranzadeh; Bruna Galvao; Carolyn Williamson; Deelan Doolabh; Diana Hardie; Gert Marais; Innocent Mudau; Lynn Tyers; Marvin Hsiao; Stephen Korsman
EPI_ISL_729274, EPI_ISL_729275, EPI_ISL_755695, EPI_ISL_755883, EPI_ISL_792087, EPI_ISL_961405	Toronto Invasive Bacterial Diseases Network	McMaster University	Ahmed Draia; Allison McGeer; Andrew G. McArthur; Angel Li; Emily Panousis; Hooman Derakhshani; Jalees Nasir; Kuganya Nirmalarajah; Michael Surette; Patryk Aftanas; Samira Mubareka
EPI_ISL_853223, EPI_ISL_853236, EPI_ISL_853247, EPI_ISL_853567, EPI_ISL_853586	Toronto Invasive Bacterial Diseases Network	Ontario Institute for Cancer Research	Allison McGeer; Angel Li; Bernard Lam; Ilinca Lungu; Jared T. Simpson; Kuganya Nirmalarajah; Lawrence E. Heisler; Lubaina Kothari; Michael Laszloffy; Patryk Aftanas; Paul Krzyzanowski; Richard de Borja; Samira Mubareka
EPI_ISL_2140718	Touwsranten Clinic wc TST	NHLS/UCT	Arash Iranzadeh; Bruna Galvao; Carolyn Williamson; Deelan Doolabh; Diana Hardie; Innocent Mudau; Kruger Marais; Lynn Tyers; Marvin Hsiao; Stephen Korsman
EPI_ISL_2779295	Trans Nzoia hospital	USAMRD-A, Basic Science Laboratory	Alan Lemtudo; Beth Mutai; Brian Andika; Carol Kifude; Clement Masakwe; Eric Muthanje; Esther Omuseni; Faith Sigei; Gathii Kimita; George Awinda; John Waitumbi; Josphat Nyataya; Rachel Githii; Rehema Liyai; Stephen Ochola
EPI_ISL_610265	TriCore Reference Laboratories	Center for Global Health, University of New Mexico Health Sciences Center	Darrell Dinwiddie; Daryl Domman; Joseph Hicks; Kurt Schwalm; Michael Edwards; Twila Kunde
EPI_ISL_717692, EPI_ISL_717697, EPI_ISL_756362, EPI_ISL_2230685, EPI_ISL_2230686	Trinidad Public Health Laboratory	Carrington Lab, Department of PreClinical Sciences, Faculty of Medical Sciences, The University of the West Indies	Adesh Ramsubhag; Arianne Brown-Jordan; Avery Hinds; Chinna Chinnadurai; Christine V. F. Carrington; Christopher Oura; Gabriel Escobar; Jaya Jayaraman; Jerome Foster; Karla Georges; Marsha Ivey; Naresh Nandram; Nikita S. D. Sahadeo; Nuno Faria; Oliver Pybus; Rahul Naidu; Rajini Haraksingh; Risha Singh; Roshan Parasram; Sarah Hill; Stanley Giddings; SueMin Nathaniel; Vernie Ramkissoon
EPI_ISL_2547369	Tshwaragano Hospital	National Health Laboratory Service/University of Cape Town (NHLS/UCT)	Arash Iranzadeh; Bruna Galvao; Carolyn Williamson; Deelan Doolabh; Diana Hardie; Gert Marais; Innocent Mudau; Lynn Tyers; Marvin Hsiao; Stephen Korsman
EPI_ISL_2802121	Tsineng Clinic	NHLS/UCT	Arash Iranzadeh; Bruna Galvao; Carolyn Williamson; Deelan Doolabh; Diana Hardie; Gert Marais; Innocent Mudau; Lynn Tyers; Marvin Hsiao; Stephen Korsman
EPI_ISL_1225161	Turkish Society of Internal Medicine	Gen Era Diagnostics Corporation Life Science Research and Molecular Diagnostics	Serhat Ünal
EPI_ISL_2625892	UAB Diagnostikos laboratorija	Institute of Biotechnology, Life Sciences Center, Vilnius University	Emilija Vasiliunaite__ Milda Norkiene__ Danguole Ziogiene__ Albertas Timinskas__ Alma Gedvilaite
EPI_ISL_2334699	UAB Diagnostikos laboratorija	National Public Health Surveillance Laboratory	Ana Steponkiene; Danas Baksa; Jelena Razmuk; Lukas Vasionis; Lukas Zemaitis; Migle Gabrielaite; Svajune Muralyte
EPI_ISL_2428034, EPI_ISL_2694419, EPI_ISL_2694447	UAB InMedica	National Public Health Surveillance Laboratory	Ana Steponkiene; Danas Baksa; Jelena Razmuk; Lukas Vasionis; Lukas Zemaitis; Migle Gabrielaite; Svajune Muralyte
EPI_ISL_1581485, EPI_ISL_2427828, EPI_ISL_2427854	UAB InMedica	Vilnius University Hospital Santaros Klinikos, Center of Laboratory Medicine	Daniel Naumovas; Dovile Ezerskyte; Gytis Dudas; Ingrida Olendraite; Laimonas Griskevicius; Ligita Raugaite; Mindaugas Stoskus; Monika Katenaite; Rimvydas Norvilas
EPI_ISL_2340539	UAB Medicina practica laboratorija	Lithuanian University of Health Sciences	Arnoldas Pautienius; Dovydas Gecys; Gediminas Alzbutas; Kamile Tamusauskaite; Lukas Zemaitis; Vaiva Lesauskaite
EPI_ISL_2082397, EPI_ISL_2694049	UAB Medicina practica laboratorija	National Public Health Surveillance Laboratory	Ana Steponkiene; Danas Baksa; Jelena Razmuk; Lukas Vasionis; Lukas Zemaitis; Migle Gabrielaite; Svajune Muralyte
EPI_ISL_2758730	UEL	IPEC Guarapuava	NAPI-Genômica (Novos Arranjo de Pesquisa e Inovação em Genômica): Ademar Dantas da Cunha Júnior Adriano Ferrasa Adriano Mondini Aldo Przybysz Alessandra Lourenço Cecchini Armani Alex Sandro Jorge Alexandra Ivo de Medeiros Alexandre Maller Aline Cristina Batista Rodrigues Johann Ana Lucia Ferreira Ana Marisa Fusco Almeida Anderson Joel Martino Andrade André Luís Laforga Vanzela Andrea Duarte Doetzer Andrea Name Colado Simao Andressa Pereira de Souza Anelisa Ramão Angelica Beate Winter Boldt Anna Hermínia Castro Gomes de Amorim Anna Silvia Penteado Setti da Rocha Antonio Camilo da Silva Filho Antonio Stabelini Neto Arthur Hirata Bertachi Barbara Mendes Paz Chao Betty Cristiane Kuhn Bruno Ambrozio Galindo Bruno Ribeiro Cruz Camilla Reginatto De Pierri Carla Fredrichsen Moya Araujo Carla Fredrichsen Moya Araujo Carlos Alberto Oliveira de Biagi Junior Carlos Augusto Nassar Carlos Eduardo Buss Carlos Gilberto Carlotti Junior Carlos Henrique Schneider Carolina Panis Carolina Weigert

			Galvão Caroline de Jesus Coelho Donha Caroline Guisantes de Salvo Toni Caryna Eurich Mazur Catiuscie Cabreira da Silva Tortorella Celso F. D. Doliveira Cesar Luiz Boguszewski Christiane Pienna Soares Chung Man Chin Claudia Moro Cleverson Busso Cristiane Cominetti Daiane Priscila Simão-Silva Dalila Luciola Zanette Daniel de Paula Daniel de Paula Daniel Rech Daniela Fiori Gradia Daniela Pretti da Cunha Tirapelli Daniela Viganó Zanoti Jeronymo Daniele Ukan Danielle Malheiros Ferreira Danielle Venturini Deborah Catharine de Assis Leite Deivid Calebe de Souza Dennis Armando Bertolini Edenir Inez Pamero Edna Maria Vissoci Reiche Edson Roberto Arpini Miguel Eduardo José de Almeida Araújo Eliana Carolina Vespero Eliandro Reis Tavares Elza Kimura Grimshaw Emanuel Maltempi de Souza Emanuele Cristina Gustani Buss Émerson Carraro Emiliana Cristina Melo ENILze Maria de Souza Fonseca Ribeiro Enilze Maria de Souza Fonseca Ribeiro Erika Izumi Erika Seki Kioshima Cotica Evani Marques Pereira Fabio Negretti Fábio Rodrigues Ferreira Seiva Felipe Dunin dos Santos Felipe Tuon Fernanda Andreia Rosa Fernanda Cestaro Prado Cortez Fernanda Ivanski Fernanda Maris Peria Flavia Regina Oliveira de Barros Franciele Aní Caovilla Follador Franciele Mara Lucca Zanardo Bohm Francinete Ramos Campos Fulviana Silva Nishiyama GABRIEL RIBEIRO CORDEIRO Gabriela Datsch Bennemann Gisele Santos de Oliveira Glaucio Valdameri Glauco Akelinghton Freire Vitiello Glauco Vieira Miranda Glaura Scantamburlo ALves Fernandes Guilherme Ferreira Silveira Gustavo Bianchini Porfírio Gustavo Lenci Marques Hélito Volpato Hildebrando Masshiro Nagai Huei Diana Lee Ilce Mara de Syllos Cólus Iris Rabinovich Israel Gomy Jackson Kawakami Jacques Duílio Brancher Jaime Luís Lopes Rocha Jaqueline Carvalho de Oliveira Jean Henrique da Silva Rodrigues Jean Leandro dos Santos Jeane Eliete Laguila Visentainer João Paulo Bianchi Ximenez Joaquim Manoel da Silva Jociani Ascari Joel Donazzolo Jorge Luis Maria Ruiz Jose Knopfholz José Luis da Conceição Silva José Sebastião dos Santos Joseane Carla Schabarum Juliana Cheleski Wiggers Juliana Mara Serpeloni Juliana Morini Küpper Cardoso Perseguini Karen Brajão de Oliveira Karin Braun Prado Karine Aparecida de Lima Katiany Rizzieri Caleffi Ferracioli Katiuscia de Oliveira Francisco Gabriel Kelvinson Fernandes Viana Larissa Beatriz Cossalter Larissa Danielle Bahls Pinto Laurival Antonio Vilas Boas Léia Carolina Lucio Líbero Mezzadri Neto Ligia Carla Faccin Galhardi Lirane Elize Defante Ferreto Luciana Furlaneto Maia Luciana Oliveira de Fariña Luciana Reis Azevedo Alanis Luciane Regina Cavalli Lucy Megumi Yamauchi Lioni Luis Paulo Gomes Mascarenhas Luis Paulo Gomes Mascarenhas Luís Paulo Mascarenhas Lupe Furtado Alle Lyvia Regina Biagi Silva Bertachi Mara Antonia Ramos Costa Mara L. Cordeiro Marcela Maria Birolim Marcelo Ricardo Vicari Marcia Edilaine Lopes Consolaro Marcia Holsbach Beltrame Marcia Regina Eches Perugini Marcos Abdo Arbex Marcos Pileggi MARCOS TADEU GRZELCZAK Marcus Peikriszwili Tartaruga Maria Angelica Ehara Watanabe Maria Antonia Ramos Costa Maria Claudia Gross Maria José Soares Mendes Giannini Maria Leandra Terencio Maria Lúcia Bonfleur Maria Luiza Guimarães de Oliveira Maria Luiza Petzl-Erler Mariana Abe Vicente Cavagnari Marina Kimiko Kadowaki Marise Fonseca dos Santos Marla Karine Amarante Maurício Turkiewicz Mauro Antonio Alves Castro Michel Rodrigo Zambrano Passarini Michele Potrich Michelle Orane Schemberger Milena Massumi Kozonoe Mônica Degraf Cavallin Monica Tereza Suldofski Mucio Luiz de Assis Cirino Nadia Graciele Krohn Najeh Maissar Khalil Nédia de Castilhos Ghisi Neide Tomimura Costa Neiva Leite Neyva Maria Lopes Romeiro Patrícia Amâncio da Rosa Patricia Dayane Carvalho Schaker Patricia Oehlmeyer Nassar Patrícia Savio de Araújo-Souza Patricia Silva Lucio Paulo Henrique Couto Souza Paulo Roberto Donadio Percy Nohama Quirino Alves de Lima Neto Rafael Deminice Rafael dos Santos Bezerra Raquel Alves dos Santos Renan Manozzo Galante Renata Ernlund Freitas de Macedo Rita de Cássia Garcia Simão Roberta Losi Guembarovski Roberto H. Herai Roberto Rosati Rodrigo Ferreira Rodrigo Rodrigues Matiello Rogério Neri Shinsato Rogério Pincela Mateus Rosane Aparecida Ribeiro Rosilene Fressatti Cardoso Rosilene Fressatti Cardoso Sandra Mara Guse Scós Venske Selene Elifio Esposito Sérgio Ossamu Ioshii Silvana Giuliatti Silvia Mara de Souza Halick Silvio Henrique Maia de Almeida Simone Neumann Wendt Spencer Luiz Marques Payão Stefan Wolanski Negrão Stephane Janaina de Moura Escobar Sueli Fumie Yamada Ogatta SUELI PERCIO QUINAIA Taciane Finatto Tatiana Mayumi Veiga Iriyoda Tayza Katelline Danilau Ostroski Tony Alexander Hild Valeria Valente Vanessa Nascimento Kozak Vanessa Santos Sotomaior Victor Breno Pedrosa Victoria Zeghbi Cochenski Borba Vivian Rotuno Moure Valdameri Wander Rogerio Pavanelli Weber Cláudio Francisco Nunes da Silva Willian Augusto de Melo Yohandra Reyes Torres
EPI_ISL_2812356, EPI_ISL_2812359, EPI_ISL_2812362, EPI_ISL_2812363, EPI_ISL_2812364, EPI_ISL_2812367, EPI_ISL_2812368, EPI_ISL_2812369, EPI_ISL_2812373, EPI_ISL_2812374, EPI_ISL_2812386, EPI_ISL_2812387, EPI_ISL_2812391, EPI_ISL_2812396, EPI_ISL_2812402, EPI_ISL_2812407, EPI_ISL_2812411, EPI_ISL_2812412, EPI_ISL_2812419, EPI_ISL_2812425, EPI_ISL_2812426, EPI_ISL_2812429, EPI_ISL_2841638			
see above	UFS Virology	KRISP, KZn Research Innovation and Sequencing Platform	Giandhari Jennifer; Naidoo Yeshnee; Pillay Sureshnee; San James; Tegally Houriiyah; Tshabuila Derek; Wilkinson Eduan; Yajna Ramphal; de Oliveira Tulio
EPI_ISL_628760	UHAS COVID-19 Lab	UHAS COVID-19 Lab	John O. Gyapong and the UHAS COVID-19 Lab Team; Jones Gyamfi; Kwabena O. Duedu; Reuben Ayivor-Djanie
EPI_ISL_747486, EPI_ISL_747487	ULSS 5 Polesana	Istituto Zooprofilattico Sperimentale delle Venezie	Adelaide Milani; Alessia Schivo; Alice Fusaro; Ambra Pastori; Annalisa Salviato; Antonia Ricci; Bianca Zecchin; Calogero Terregino; Erika Giorgia Quaranta; Isabella Monne
EPI_ISL_1664743	UMMC-Health	WHO National Influenza Centre Russian Federation	Andrey Komissarov; Anna Ivanova; Artem Fadeev; Daria Danilenko; Dmitry Lioznov; Elena Nabieva; Georgii Bazykin; Ksenia Safina; Kseniya Komissarova; Maria Pisareva; Maria Timofeeva; Mikhail Bakaev; Tamila Musaeva; Tatiana Platonova; Veronika Eder
EPI_ISL_2092861	UNC Charlotte COVID-19 Testing Lab	UNC Charlotte Environmental Monitoring Laboratory	Angelica Martins; Cynthia Gibas; Jannatul Ferdous; Jessica Schlueter; Kevin Lambirth; Visva Barua
EPI_ISL_2645959, EPI_ISL_2989027, EPI_ISL_2989037	UNILABS	Instituto Nacional de Saude (INSA)	Borges et al
EPI_ISL_2796010	UNILABS	Instituto Nacional de Saude (INSA) and Instituto Gulbenkian de Ciencia (IGC)	Borges et al
EPI_ISL_853342	UPMC Clinical Microbiology Laboratory	Microbial Genome Sequencing Center; Microbial Genomic Epidemiology Laboratory	Chinelo Ezeonwuku; Dan Snyder; Jane W. Marsh; Kady D. Waggle; Lee H. Harrison; Marissa P. Griffith; Mustapha M. Mustapha; Stephanie L. Mitchell; Vatsala R. Srinivasa; Vaughn S. Cooper
EPI_ISL_1137240, EPI_ISL_1732367	UPMC Clinical Microbiology Laboratory	Microbial Genome Sequencing Center; Microbial Genomic Epidemiology Laboratory, University of Pittsburgh	Daniel J. Snyder; Jane W. Marsh; Kady D. Waggle; Lee H. Harrison; Marissa P. Griffith; Stephanie L. Mitchell; Vatsala R. Srinivasa; Vaughn S. Cooper
EPI_ISL_983464, EPI_ISL_1227749, EPI_ISL_1227750, EPI_ISL_2278770	URMC LABS	Wadsworth Center, New York State Department of Health	Alexis Russel; Alexis Russell; Catharine Prussing; Daryl M. Lamson; Erasmus Schneider; Erica Lasek-Nesselquist; John Kelly; Jonathan Plitnick; Kirsten St. George; Matthew Shudt; Melissa A Leisner; Navjot Singh
EPI_ISL_2383566, EPI_ISL_2661466	US Air Force School of Aerospace Medicine	US Air Force School of Aerospace Medicine	Amanda Javorina; Anthony Fries; Carol Garrett; Clarise Starr; Cole Anderson; Elizabeth Macias; Fritz Castillo; Jennifer Meyer; Sarah Purves; William Gruner
EPI_ISL_436045	US VI Department of Health	Pathogen Discovery, Respiratory Viruses Branch, Division of Viral Diseases, Centers for Disease Control and Prevention	Anna Uehara; Bettina Bankamp; Clinton R. Paden; Haibin Wang; Jing Zhang; Krista Queen; Suxiang Tong; Yan Li; Ying Tao; Zachary Weiner
EPI_ISL_903891	UT-Unified State Labs: Public Health Utah DOH	Genomics and Discovery, Respiratory Viruses Branch, Division of Viral Diseases, Centers for Disease Control and Prevention	Anna Montmayeur; Anna Uehara; Ben L. Rambo-Martin; Clinton R. Paden; Dhwani Batra; Haibin Wang; Jasmine Padilla; Jing Zhang; Justin Lee; Krista Queen; Lori Rowe; Mark Burroughs; Mili Sheth; Peter W. Cook; Rachel Marine; Sarah Nobles; Suxiang Tong; Yan Li; Ying Tao
EPI_ISL_424229, EPI_ISL_1324125, EPI_ISL_1621123, EPI_ISL_1785784, EPI_ISL_2194129, EPI_ISL_2773932	UW Virology Lab	UW Virology Lab	Alexander Greninger; Hong Xie; Keith Jerome; Keith R Jerome; Lasata Shrestha; Margaret Mills; Meei-Li Huang; Michelle Lin; Nathan Breit; Noah Baker; Noah R. Baker; Patrick Mathias; Pavitra Roychoudhury; Ricardo Perez; Robert J. Livingston; Saraswathi Sathees; Sean Ellis; Shah Mohamed Bakhash; Tien V. Nguyen
EPI_ISL_734804, EPI_ISL_734899, EPI_ISL_735130	UZ Leuven, National Reference Laboratory for Coronaviruses, Laboratory Medicine, Leuven, Belgium	KU Leuven, Rega Institute, Clinical and Epidemiological Virology	Bert Vanmechelen; Joan Marti-Carerras; Piet Maes; Tony Wawina-Bokalanga
EPI_ISL_737958, EPI_ISL_738036	Uganda Central Public Health Lab and Uganda Virus Research Institute	MRC/UVRI & LSHTM Uganda Research Unit	Dan Lule Bugembe; Matthew Cotten; My V.T. Phan; Pontiano Kaleebu et al.
EPI_ISL_451189	Uganda Virus Research Institute	MRC/UVRI & LSHTM Uganda Research Unit	Beatrice Dhaala; Dan Lule Bugembe; Deogratius Ssemwanga; Henry Kyobe; Henry Mwebesa; Jane Aceng; John Kayiwa; Jonas Lexow; Julius Lutwama; Matthew Cotten; My V.T Phan; Phionah Tushabe; Pontiano Kaleebu; Stephen Balinandi
EPI_ISL_2828019	Unidad de Investigación Molecular (UNIMOL)	Instituto Nacional de Salud- Dirección de Investigación en Salud Pública	Carlos Franco-Muñoz; Carmen Osorio; Diana Malo; Diego A. Álvarez-Díaz; Diego Andrés Prada; Gerardo Santamaría; Hector Alejandro Ruiz-Moreno; Jhonnatan Reales-González; Jorge Rivera; Juan Camilo Martinez; Julian Naizaque; Katherine Laiton-Donato; Lisseth Pardo; Magdalena Wiesner; Marcela Mercado-Reyes; Maria T. Herrera-Sepúlveda; Marta Lopez Blanco; Martha Lucia Ospina Martinez; Paola Rojas; Sergio Gomez; Sheryll Corchuelo; Ángela Alarcon Cruz
EPI_ISL_1640127	Uniklinikum Carl Gustav Carus an der TU Dresden; Institut fÃ¼r Virologie	Robert Koch Institute	
EPI_ISL_549175, EPI_ISL_1317690, EPI_ISL_1490459, EPI_ISL_2509362, EPI_ISL_2509368, EPI_ISL_2509369, EPI_ISL_3031028			
see above	Unilabs Laboratory Medicine	Norwegian Institute of Public Health, Department of Virology	Atiya R Ali; Debech Nadia; Engebretsen Serina Beate; Garcia Llorente Ignacio; Hilde Elshaug; Hilde Synnøve Vollan; Hilde Vollan; Jon Bråte; Kamilla Heddeland Instefjord; Karoline Bragstad; Kathrine Stene-Johansen; Line Victoria Moen; Marie Paulsen Madsen; Olav Hungnes; Pedersen Benedikte Nevjen; Rasmus Riis Kopperud
EPI_ISL_1299863, EPI_ISL_1299864	Unit of lab surveillance of viral emerging diseases, National Lab of Influenza	Respiratory Virus Unit, National Infection Service, Public Health England	Iris Hasibra; PHE Covid Sequencing Team; Prof Albana Fico; Prof Silvia Bino
EPI_ISL_812548, EPI_ISL_831801	United States Air Force School of Aerospace Medicine	United States Air Force School of Aerospace Medicine	Amanda Javorina; Anthony Fries; Clarise Starr; Elizabeth Macias; Jennifer Meyer; Sarah Purves; William Gruner
EPI_ISL_569963, EPI_ISL_570008, EPI_ISL_2823743	Unity Health Toronto	Ontario Institute for Cancer Research	Bernard Lam; Felicia Vincelli; Ilinca Lungu; Jared T. Simpson; Jeremy Johns; Karel Boissinot; Larissa M. Matukas; Le Luu; Mark Downing; Paul Krzyzanowski; Philip Zuzarte; Ramzi Fattouh; Richard de Borja; Samira Mubareka; TIBDN; Trina Otterman; Wai Sum Siu; Yan Chen; Zhi Cui
EPI_ISL_888804	Univeristy of New Mexico Hospital	Center for Global Health, University of New Mexico Health Sciences Center	Darrell Dinwiddie; Daryl Domman; Jon Femling; Justin Bacca; Kurt Schwalm
EPI_ISL_3024078	Universidad Autonoma de Yucatan	New York Genome Center	Amy Baldwin; Andre Corvelo; Aviles-Gomez E; Ayora-Talavera G; Chan-Gasca M; Dayna M. Oschwald; Flores-Quintal F; Granja-Perez P; Lopez-Coral L.; Michael Zody; Samantha Fennessey; Tom Maniatis; Villanueva-Jorge S; Yam-Pool E
EPI_ISL_697796	Universidad Regional Amazonica IKIAM	Institute of Microbiology, Universidad San Francisco de Quito	Andrea Carrera; Belén Prado-Vivar; Bernardo Gutiérrez; Carolina Proaño-Bolaños; Fabian Aguilar; Gabriel Trueba; Giovanna Moran; Juan José Guadalupe; Katherine Apunte; Marcelo Ortiz; Michelle Grunauer; Monica Becerra-Wong; Nina Espinoza de los Monteros; Patricio Rojas-Silva; Paúl Cárdenas; Sonia Sislema; Sully Márquez; Verónica Barragán; Yeimy Rojas
EPI_ISL_1656146	Universidad Tecnica Particular de Loja	Institute of Microbiology, Universidad San Francisco de Quito	Belén Prado-Vivar; Bernardo Gutiérrez; David Zuñiga; Fernando Serrano; Gabriel Trueba; Juan José Guadalupe; Katherine Ojeda; Luis Flores; Melissa Ortega; Michelle Grunauer; Monica Becerra-Wong; Paola Dalgo; Patricio Rojas-Silva; Paúl Cárdenas; Raiza Briceño; Sully Márquez; Verónica Barragán
EPI_ISL_2123875	Universitatsklinikum Koln; Institut fur Virologie	Robert Koch Institute	
EPI_ISL_812967	University Clinical Research Center, University of Sciences	University Clinical Research Center, University of Sciences	A.A.; Bane, S.; Dao, S.; Diakite, M.; Diarra, B.; Doumbia, S.; Guindo, I.; Iknane; Kone, A.
EPI_ISL_2895002	University College Hospital, Ibadan	African Centre of Excellence for Genomics of Infectious Diseases (ACEGID), Redeemer's University, Nigeria	A.T.; Abechi; Ajogbasile; Akano; C.A.; C.T.; Eromon; F.V.; Folarin, O.; Fowotade, A.; Happi; I.B.; J.N.; J.U.; K.O.; Kayode; Nosamiefan, I.; Oguzie; Olawoye; Olumade; Oluniyi; P.E.; P.S.; T.J.; Ugwu; Uwanibe
EPI_ISL_741244, EPI_ISL_865865	University College London, Great Ormond Street Hospital for Children NHS Foundation Trust, Imperial College Healthcare NHS Trust	COVID-19 Genomics UK (COG-UK) Consortium	Alison Holmes; Charlotte Williams; Helena Tutill; Jacqueline Findlay; James Price; Judith Breuer; Julianne Brown; Kathryn Harris; Leysa Forrest; Mark Kristiansen; Paola Niola; Paola Resende Silva; Patricia Dyal; Paul Randell; Rachel Williams; Samuel Weeks; Sergi Castellano; Sunando Roy; Tony Brooks; Yasmin Panchbhaya
EPI_ISL_982819, EPI_ISL_982938, EPI_ISL_982943, EPI_ISL_982954, EPI_ISL_982981, EPI_ISL_983011, EPI_ISL_983187			
see above	University Health Network/Mount Sinai Hospital Department of Microbiology	Ontario Institute for Cancer Research	Aimee Paterson; Allison McGeer; Angel Liu; Bernard Lam; Cassandra Bergwerff; Ilinca Lungu; Jared T. Simpson; Javier Hernandez; Jeff Wrana; Jessica Bourke; Kin Chan; Kuganya Nirmalarajah; Laurence Pelletier; Lawrence E. Heisler; Lubaina Kothari; Marc Mazzulli; Marie-Ming Aynaud; Michael Laszloffy; Patryk Aftanas; Paul Krzyzanowski; Richard de Borja; Samira Mubareka; Seda Barutcu; Tony Mazzulli
EPI_ISL_710555, EPI_ISL_710559, EPI_ISL_710570	University Hospital Dubrava	Ruđer Bošković Institute; Forensic Science Centre Ivan Vučetić; University of Zagreb Faculty of Science	Ana Livun; Antonela Blažeković; Boris Maček; Danilo Licastro; Dunja Glavaš; Fran Borovečki; Fuad Ćosović; Gordana Maravić Vlahoviček; Ivan Šamija; Ivana Ćelap; Jasna Kašman; Josipa Skelin; Katarina Marija Tupek; Kristian Vlahoviček; Kristina Gotovac Jerečić; Lidija Cvetko-Krajinović; Lucija Basić; Lucija Markulin; Maja Kuzman; Marina Korolija; Mario Štefanović; Mirjana Domazet-Lošo; Paula Štancl; Petra Vrabec; Robert Belužić; Rosa Karlić; Sanja Tadinac; Senčica Pejša; Tomislav Domazet-Lošo; Valentina Đumlijan-Combaj; Vjekoslav Tomaić; Vladimir Krajinović; Željka Maček Šafranko

EPI_ISL_2790388	University Hospital Foca	Public Health Institute of Republic of Srpska	Branka Culibrk; Dijana Vukajlovic; Milica Celic; Pava Dimitrijevic; Stanka Tomic; Tatjana Markovic; Zeljka Sumic
EPI_ISL_681761	University Hospital Limerick	Irish Coronavirus Sequencing Consortium - Teagasc Moorepark	Amy Fitzpatrick; Calum Walsh; Carolyn Meaney; Elaine Lawton; Fiona Crispie; John Kenny; Patrick Stapleton; Paul Cotter
EPI_ISL_1254573, EPI_ISL_2357760	University Hospital of Northern Norway, Department for Microbiology and Infectious Disease Control	Norwegian Institute of Public Health, Department of Virology	Atiya R Ali; Debech Nadia; Engebretsen Serina Beate; Garcia Llorente Ignacio; Hilde Elshaug; Hilde Vollan; Jon Bråte; Kamilla Heddeland Instefjord; Karoline Bragstad; Kathrine Stene-Johansen; Line Victoria Moen; Marie Paulsen Madsen; Olav Hungnes; Pedersen Benedikte Nevjen; Rasmus Riis Kopperud
EPI_ISL_429207	University Hospitals of Geneva Laboratory of Virology	University Hospitals of Geneva Laboratory of Virology	Laubscher F.
EPI_ISL_897794	University Hospitals of Geneva, Laboratory of Virology	HUG, Laboratory of Virology and the Health2030 Genome Center	Ana Rita Goncalves; Deborah Penet; Emmanouil Dermitzakis; Henri Pegeot; Ioannis Xenarios; Keith Harshman; Laurent Kaiser; Lorenzo Cerutti; Melyssa Elies; Samuel Cordey
EPI_ISL_2384717	University Medical Center Hamburg Eppendorf	Heinrich Pette Institute, Leibniz Institute for Experimental Virology	Adam Grundhoff; Alexis Robitaille; Johannes Knobloch; Martin Aepfelbacher; Nicole Fischer; Thomas Günther
EPI_ISL_2931002	University of Arkansas for Medical Sciences	Center for Global Health, University of New Mexico Health Sciences Center	Catherine Kirkpatrick; Darrell Dinwiddie; Daryl Domman; David Ussery; Jennifer Laudadio; Joshua L. Kennedy; Kurt Schwalm; Susan Harley; Uday Chalwadi; Valerie Morley
EPI_ISL_1072985	University of Balamand	Microbial Genomics Lab, Lebanese American University, Byblos	Mira El Chaar; Sima Tokajian; Youssef Bassim
EPI_ISL_2100503	University of Health Sciences	Quadram Institute Bioscience	Alam Khan; Alexander J Trotter; Alison E. Mather; Almina Shafiq; Alp Aydin; Ana P. Tedim; Anastasia Kolyva; Andrew Bell; Andrew J. Page; Claire Stuart; Dave J. Baker; Gemma L. Kay; Javed Akram; John Wain; Justin O'Grady; Leonardo de Oliveira Martins; Lizzie Meadows; Maria Diaz; Mark Webber; Muhammad Bilal Sarwar; Muhammad Roman; Muhammed Yasir; Nabil-Fareed Alikhan; Nadeem Afzal; Ngozi Elumogo; Nicholas M. Thomson; Rachael Stanley; Rachel Gilroy; Reenesh Prakash; Samir Dervisevic; Samuel Bloomfield; Shah Jahan; Sidra-tul-muntaha; Steven Rudder; Thanh Le-Viet
EPI_ISL_2864611	University of Health and Allied Sciences (UHAS) COVID-19 Testing and Research Centre	University of Health and Allied Sciences (UHAS) COVID-19 Testing and Research Centre	John O. Gyapong and the UHAS COVID-19 Lab Team; Jones Gyamfi; Kwabena O. Duedu; Reuben Ayivor-Djanie
EPI_ISL_2500011, EPI_ISL_2728657	University of Liège COVID-19 testing center	GIGA Medical Genomics	Bouchra Boujemla; Claire Gourzonès; Cécile Meex; Fabrice Bureau; Keith Durkin; Laurent Gillet; Maria Artesi; Marie-Pierre Hayette; Nadine Cambisano; Nathalie Renotte; Olivier Ek; Sébastien Bontems; Vincent Bours; Wouter Coppieters
EPI_ISL_2242506, EPI_ISL_2243223	University of Maiduguri Teaching Hospital	International Centre for Genetic Engineering and Biotechnology (ICGEB) and ARGO Open Lab for Genome Sequencing	Alessandro Marcello; Bamidele S Oderinde; Danilo Licastro; Emanuele Orsini; Galadima Gadzama; Marycelin M Baba; Monilade Akinola; Simeone Dal Monego; Zara Wuduri
EPI_ISL_1118703	University of Michigan Clinical Microbiology Laboratory	Lauring Lab, University of Michigan, Department of Microbiology and Immunology	Valesano
EPI_ISL_955142, EPI_ISL_955144, EPI_ISL_955148, EPI_ISL_955170, EPI_ISL_1016690, EPI_ISL_1016822	University of Sarajevo, Veterinary Faculty, Laboratory for Molecular Diagnostic and Research Laboratory	University of Sarajevo, Veterinary Faculty, Laboratory for Molecular Diagnostic and Research Laboratory	Alic-Seho A.; Alić-Šeho A.; Goletic S.; Goletic T.; Goletić T.; Goletić Š.; Hodzic A.; Hodžić A.; Jazic A.; Jažić A.; Nicevic M.; Nicević M.; Sabic E.; Softic A.; Softić A.; Terzic I.; Terzić I.; Terzić I. Jažić A.; Šabić E.
EPI_ISL_476078, EPI_ISL_677765	University of Szeged, Institute of Clinical Microbiology	National Laboratory of Virology, Szentágothai Research Centre	Balázs Somogyi; Brigitta; Brigitta Zana; Endre Gábor Tóth; Ferenc Jakab; Gabriella Terhes; Gábor Kemenesi; Terhes Gabriella
EPI_ISL_485391, EPI_ISL_485395	University of Ulsan College of Medicine and Asan Medical Center	University of Ulsan College of Medicine and Asan Medical Center	Heungsup Sung; Jaewoong Lee; Jina Lee; Jiwon Jung; Jongsik Chun; Kihyun Lee; Kuenyoul Park; Kyu-Hwa Hur; Mauricio Chalita; Mi-Na Kim; Seok-Hwan Yoon; Sung-Han Kim; and Hae Kyung Lee
EPI_ISL_509950, EPI_ISL_1822080	University of Wisconsin-Madison AIDS Vaccine Research Laboratories	University of Wisconsin-Madison AIDS Vaccine Research Laboratories	Gage Moreno; Katarina Braun; et al. AIDS Vaccine Research Laboratories
EPI_ISL_977291, EPI_ISL_977342, EPI_ISL_977350, EPI_ISL_977357, EPI_ISL_977367, EPI_ISL_977373, EPI_ISL_977421, EPI_ISL_977422, EPI_ISL_2803362, EPI_ISL_2803437, EPI_ISL_2803449, EPI_ISL_2803512, EPI_ISL_2803529, EPI_ISL_2803584, EPI_ISL_2803629, EPI_ISL_2803630, EPI_ISL_2803631, EPI_ISL_2803632, EPI_ISL_2803635, EPI_ISL_2803636, EPI_ISL_2803638, EPI_ISL_2803639, EPI_ISL_2803645, EPI_ISL_2803646, EPI_ISL_2803648, EPI_ISL_2803666, EPI_ISL_2803668, EPI_ISL_2803673, EPI_ISL_2803674, EPI_ISL_2803685, EPI_ISL_2803687, EPI_ISL_2803693, EPI_ISL_2803694, EPI_ISL_2803698, EPI_ISL_2803701, EPI_ISL_2803702, EPI_ISL_2803709, EPI_ISL_2803714, EPI_ISL_2803716, EPI_ISL_2803718, EPI_ISL_2803729, EPI_ISL_2803730, EPI_ISL_2803732			
see above	University of Zambia, School of Veterinary Medicine	UNZAVET and PATH	Daniel Bridges; Mulenga Mwenda-Chimfwembe; Ngonda Saasa; ZNPHI and ZGSC
EPI_ISL_1721045	UniversitÃ¤tsklinikum des Saarlandes - Institut fÃ¼r Virologie	Robert Koch Institute	
EPI_ISL_2382023, EPI_ISL_2382028, EPI_ISL_2382031, EPI_ISL_2382032	Universität Innsbruck, Institut für Mikrobiologie	Bergthaler laboratory, CeMM Research Center for Molecular Medicine of the Austrian Academy of Sciences	Andreas Bergthaler; Anna Schedl; Bekir Erguner; Benedikt Agerer; Christoph Bock; Fabian Amman; Jan Laine; Lukas Endler; Maelle Le Moing; Martin Senekowitsch; Michael Schuster; Petr Triska; Thomas Penz
EPI_ISL_1286241	Universitätsklinikum Heidelberg	Robert Koch Institute	
EPI_ISL_628939, EPI_ISL_1313869, EPI_ISL_2279888, EPI_ISL_2290332, EPI_ISL_2290425, EPI_ISL_2679594, EPI_ISL_2856552, EPI_ISL_2857105			
see above	Utah Public Health Laboratory	Utah Public Health Laboratory	Erin L. Young; Erin Young; Kelly F. Oakeson; Kelly Oakeson; Olinto Linares-Perdomo; Tara Gallagher
EPI_ISL_2382667	VERMAAK	National Institute for Communicable Diseases of the National Health Laboratory Service	Amoako DG; Bhiman JN; Ismail A; Mahlangu B; Mohale T; Ntuli N; Scheepers C
EPI_ISL_2662532, EPI_ISL_2662536	VERMAAK LABORATORY	National Institute for Communicable Diseases of the National Health Laboratory Service	Amoako DG; Bhiman JN; Everatt J; Ismail A; Mahlangu B; Mnguni A; Mohale T; Ntuli N; Scheepers C
EPI_ISL_1094245	VT Dept. of Health Laboratory	Respiratory Viruses Branch, Division of Viral Diseases, Centers for Disease Control and Prevention	Anna Montmayeur; Anna Uehara; Ben L. Rambo-Martin; Clinton R. Paden; Dhwani Batra; Haibin Wang; Jasmine Padilla; Jing Zhang; Justin Lee; Krista Queen; Lori Rowe; Mark Burroughs; Mili Sheth; Peter W. Cook; Rachel Marine; Sarah Nobles; Suxiang Tong; Yan Li; Ying Tao
EPI_ISL_2494898	Vaccines and Infectious Diseases Analytics Research Unit (VIDA)	KRISP, KZN Research Innovation and Sequencing Platform	Baillie Vicky; Giandhari Jennifer; Madhi Shabir; Naidoo Yeshnee; Pillay Sureshnee; San James; Tegally Houriiyah; Wilkinson Eduan; de Oliveira Tulio; du Plessis Jeanine
EPI_ISL_2360774, EPI_ISL_2360878, EPI_ISL_2494391, EPI_ISL_2494501, EPI_ISL_2494557, EPI_ISL_2494585	Vaccines and Infectious Diseases Analytics Research Unit (VIDA)	KRISP, KZn Research Innovation and Sequencing Platform	Baillie Vicky; Giandhari Jennifer; Madhi Shabir; Naidoo Yeshnee; Pillay Sureshnee; San James; Tegally Houriiyah; Tshabuila Derek; Wilkinson Eduan; de Oliveira Tulio; du Plessis Jeanine
EPI_ISL_2521377, EPI_ISL_3016436	Valais Hospital, Central Institute	Valais Hospital, Central Institute	Alexis Dumoulin; Deborah Penet; Emmanouil Dermitzakis; Henri Pegeot; Ioannis Xenarios; Keith Harshman; Lorenzo Cerutti; Melyssa Elies
EPI_ISL_1373898	Vanderbilt University Medical Center	Pathogen Discovery, Respiratory Viruses Branch, Division of Viral Diseases, Centers for Disease Control and Prevention	Anna Kelleher; Anna Uehara; Brian Lynch; Clinton R. Paden; Haibin Wang; Han Jia Justin Ng; Jing Zhang; Krista Queen; Peter Cook; Suxiang Tong; Yan Li; Ying Tao
EPI_ISL_640096	Vanguard CHC wc VGC	NHLS/UCT	Arash Iranzadeh; Bruna Galvao; Carolyn Williamson; Deelan Doolabh; Diana Hardie; Innocent Mudau; Kruger Marais; Lynn Tyers; Marvin Hsiao; Stephen Korsman
EPI_ISL_2582736	Vermaak & Partners Laboratory	National Institute for Communicable Diseases of the National Health Laboratory Service	Amoako DG; Bhiman JN; Everatt J; Ismail A; Mahlangu B; Mnguni A; Mohale T; Ntuli N; Scheepers C
EPI_ISL_2001620	Vestfold Hospital, Toensberg Department of Microbiology	Norwegian Institute of Public Health, Department of Virology	Atiya R Ali; Debech Nadia; Engebretsen Serina Beate; Garcia Llorente Ignacio; Hilde Elshaug; Hilde Vollan; Jon Bråte; Kamilla Heddeland Instefjord; Karoline Bragstad; Kathrine Stene-Johansen; Marie Paulsen Madsen; Olav Hungnes; Pedersen Benedikte Nevjen; Rasmus Riis Kopperud
EPI_ISL_678490	Veterinary Specialized Institute "Sabac", Serbia	Veterinary Specialized Institute "Kraljevo", Serbia	Afonso, C.; Banovic Djeri, B.; Jankovic, M.; Jovanovic, T.; Knezevic, A.; Mrkovacki, S.; Petrovic, T.; Sekler, M.; Tesovic, B.; Vidanovic, D.; Volkening, J.
EPI_ISL_833517	Veterinary Specialized Instute "Nis"	Veterinary Specialized Institute "Kraljevo", Serbia	Afonso, C.; Banovic Djeri, B.; Jankovic, M.; Jovanovic, T.; Knezevic, A.; Manic, M.; Petrovic, M.; Petrovic, T.; Sekler, M.; Tesovic, B.; Vidanovic, D.; Volkening, J.
EPI_ISL_2227287	Veterinary institute in Zvolen, Pod Drahami 918, 960 86 Zvolen Slovakia	Veterinary institute in Zvolen, Pod Drahami 918, 960 86 Zvolen Slovakia	Dirbáková Z.; Mojžiš M.; Mokryšová S.; Sujová S.; Tinák M.
EPI_ISL_1040814, EPI_ISL_2140693, EPI_ISL_2375993	Victoria Hospital wc VHW	NHLS/UCT	Arash Iranzadeh; Bruna Galvao; Carolyn Williamson; Deelan Doolabh; Diana Hardie; Innocent Mudau; Kruger Marais; Lynn Tyers; Marvin Hsiao; Stephen Korsman
EPI_ISL_426857, EPI_ISL_456455, EPI_ISL_456565	Victorian Infectious Diseases Reference Laboratory (VIDRL)	Microbiological Diagnostic Unit Public Health Laboratory and Victorian Infectious Diseases Reference Laboratory, Doherty Institute	Caly L.; Druce J.; Sait, M.; Schultz M.; Seemann T.; Sherry, N.
EPI_ISL_521971, EPI_ISL_640784, EPI_ISL_779621, EPI_ISL_779622, EPI_ISL_877578, EPI_ISL_1913202, EPI_ISL_1913204, EPI_ISL_1913205, EPI_ISL_1913206, EPI_ISL_1913211, EPI_ISL_2920928, EPI_ISL_2920932, EPI_ISL_2920946, EPI_ISL_2920948, EPI_ISL_2920952, EPI_ISL_2920955, EPI_ISL_2920960, EPI_ISL_2920963, EPI_ISL_2920974, EPI_ISL_2920989, EPI_ISL_2920990, EPI_ISL_2920998, EPI_ISL_2978349, EPI_ISL_3030415			
see above	Victorian Infectious Diseases Reference Laboratory (VIDRL)	VIDRL and MDU-PHL	Caly L.; Druce J.; M.L.; N.L.; Sait; Sait, M.; Schultz M.; Schultz M.B.; Seemann T.; Sherry; Sherry, N.
EPI_ISL_416410, EPI_ISL_416411, EPI_ISL_419733, EPI_ISL_419735	Victorian Infectious Diseases Reference Laboratory (VIDRL)	Victorian Infectious Diseases Reference Laboratory and Microbiological Diagnostic Unit Public Health Laboratory, Doherty Institute	Caly L.; Druce J.; Sait, M.; Schultz M.; Seemann T.; Sherry, N.; Taiaroa, G.
EPI_ISL_2694573	Viesoji istaiga Respublikine Siauliu ligonine	National Public Health Surveillance Laboratory	Ana Steponkiene; Danas Baksa; Jelena Razmuk; Lukas Vasionis; Lukas Zemaitis; Migle Gabrielaite; Svajune Muralyte

EPI_ISL_2339638, EPI_ISL_2510724	Viesoji istaiga Vilniaus universiteto ligonine Santaros klinikos	Vilnius University Hospital Santaros Klinikos, Center of Laboratory Medicine	Daniel Naumovas; Dovile Ezerskyte; Gytis Dudas; Ingrida Olendraite; Laimonas Griskevicius; Ligita Raugaite; Mindaugas Stoskus; Monika Katenaite; Rimvydas Norvilas
EPI_ISL_2779343	Vihiga County referral hospital	USAMRD-A, Basic Science Laboratory	Alan Lemtudo; Beth Mutai; Brian Andika; Carol Kifude; Clement Masakwe; Eric Muthanje; Esther Omuseni; Faith Sigei; Gathii Kimita; George Awinda; John Waitumbi; Josphat Nyataya; Rachel Githii; Rehema Liyai; Stephen Ochola
EPI_ISL_2802125	Villiersdorp Clinic wc VDP	NHLS/UCT	Arash Iranzadeh; Bruna Galvao; Carolyn Williamson; Deelan Doolabh; Diana Hardie; Gert Marais; Innocent Mudau; Lynn Tyers; Marvin Hsiao; Stephen Korsman
EPI_ISL_904938, EPI_ISL_904952, EPI_ISL_904953	Vilnius University Hospital Santaros Klinikos, Vilnius University	Institute of Biotechnology, Life Sciences Center, Vilnius University	Albertas Timinskas; Alma Gedvilaite; Aurelija Zvirbliene; Daniel Naumovas; Emilija Vasiliunaite; Laimonas Griskevicius; Milda Norkiene
EPI_ISL_560398, EPI_ISL_560405	Vilnius University Hospital Santaros Klinikos, Vilnius University	Institute of Biotechnology, Life Sciences Center, Vilnius University and Thermo Fisher Scientific	Albertas Timinskas; Alma Gedvilaite; Aurelija Zvirbliene; Daniel Naumovas; Justinas Slikas; Laimonas Griskevicius; Ligita Jancioriene; Mindaugas Paulauskas
EPI_ISL_933832, EPI_ISL_933912, EPI_ISL_934070, EPI_ISL_1005262, EPI_ISL_1005284, EPI_ISL_1005354	Vilnius university hospital Santaros Klinikos, Center of Laboratory Medicine	Vilnius university hospital Santaros Klinikos, Center of Laboratory Medicine	Daniel Naumovas; Dovile Ezerskyte; Gytis Dudas; Ingrida Olendraite; Justinas Slikas; Laimonas Griskevicius; Rimvydas Norvilas
EPI_ISL_1658231, EPI_ISL_1914247, EPI_ISL_1914248	Viollier AG	Department of Biosystems Science and Engineering, ETH Zurich	Andrea Patrignani; Andreia Cabral de Gouvea; Catharine Aquino; Chaoran Chen; Christian Beisel; Christiane Beckmann; Christoph Noppen; David Dreifuss; Doris Popovic; Elodie Burcklen; Griffin White; Ina Nissen; Ivan Topolsky; Jay Tracy; Katharina Jahn; Lara Fuhrmann; Laura Neff; Lennart Opitz; Maria Domenica Moccia; Maurice Redondo; Mirjam Feldkamp; Natascha Santacroce; Niko Beerenwinkel; Noemie Santamaria de Souza; Olivier Kobel; Philipp Jablonski; Ralph Schlapbach; Rebecca Denes; Sarah Nadeau; Simon Gruter; Sophie Seidel; Tanja Stadler; Timothy Sykes
EPI_ISL_451864, EPI_ISL_486506, EPI_ISL_500907, EPI_ISL_693877, EPI_ISL_721718, EPI_ISL_721726, EPI_ISL_737488, EPI_ISL_737507, EPI_ISL_737516, EPI_ISL_737673, EPI_ISL_737925, EPI_ISL_737926, EPI_ISL_767993, EPI_ISL_899061, EPI_ISL_899109, EPI_ISL_899362, EPI_ISL_1001507, EPI_ISL_1002753, EPI_ISL_1002759, EPI_ISL_1002868, EPI_ISL_1003152, EPI_ISL_1003836, EPI_ISL_1004279, EPI_ISL_1059306, EPI_ISL_1119265, EPI_ISL_1119341, EPI_ISL_1119430, EPI_ISL_1119431, EPI_ISL_1130149, EPI_ISL_1130743, EPI_ISL_1130884, EPI_ISL_1195147, EPI_ISL_1195162, EPI_ISL_1195173, EPI_ISL_1259963, EPI_ISL_1260061, EPI_ISL_1260151, EPI_ISL_1260423, EPI_ISL_1406785, EPI_ISL_1407469, EPI_ISL_1408298, EPI_ISL_1408489, EPI_ISL_1408490, EPI_ISL_1408791, EPI_ISL_1496311, EPI_ISL_1597912, EPI_ISL_1750693, EPI_ISL_1750789, EPI_ISL_2152242, EPI_ISL_2211587, EPI_ISL_2307509, EPI_ISL_2308029, EPI_ISL_2375082, EPI_ISL_2375120, EPI_ISL_2662389, EPI_ISL_2820594, EPI_ISL_2820604, EPI_ISL_2938399, EPI_ISL_3007533			
see above	Viollier AG	Department of Biosystems Science and Engineering, ETH Zürich	Andrea Patrignani; Andrea Patrizia Salzmann; Andreia Cabral de Gouvea; Catharine Aquino; Chaoran Chen; Christian Beisel; Christiane Beckmann; Christoph Noppen; Daniel Ehrsam; David Dreifuss; Deborah Penet; Doris Popovic; Elodie Burcklen; Emmanouil Dermitzakis; Griffin White; Henri Pegeot; Henriette Kurth; Ina Nissen; Ioannis Xenarios; Isabel Stürmer; Ivan Topolsky; Jay Tracy; Katharina Jahn; Keith Harshman; Kim Philipp Jablonski; Lara Fuhrmann; Laura Neff; Lennart Opitz; Lorenzo Cerutti; Maria Domenica Moccia; Maurice Redondo; Mirjam Feldkamp; Natascha Santacroce; Niko Beerenwinkel; Noemie Santamaria de Souza; Olivier Kobel; Pedro Ferreira; Philipp Jablonski; Ralph Schlapbach; Rebecca Denes; Sarah Nadeau; Simon Grüter; Sophie Seidel; Susana Posada-Céspedes; Tanja Stadler; Timothy Sykes; Tobias Schär
EPI_ISL_417946, EPI_ISL_420841, EPI_ISL_513597, EPI_ISL_514125, EPI_ISL_527492, EPI_ISL_527532, EPI_ISL_527543, EPI_ISL_527547, EPI_ISL_591086, EPI_ISL_965122, EPI_ISL_965123, EPI_ISL_965124, EPI_ISL_1623757, EPI_ISL_1785567, EPI_ISL_2135837, EPI_ISL_2135838, EPI_ISL_2135840, EPI_ISL_2135841, EPI_ISL_2135843, EPI_ISL_2135844, EPI_ISL_2135845, EPI_ISL_2135846, EPI_ISL_2966635, EPI_ISL_2966637, EPI_ISL_2966638, EPI_ISL_2966639, EPI_ISL_2966640, EPI_ISL_2966644, EPI_ISL_2966646, EPI_ISL_2966650, EPI_ISL_2966653, EPI_ISL_2968537, EPI_ISL_2968539, EPI_ISL_2968551, EPI_ISL_2968556, EPI_ISL_2968557, EPI_ISL_2968689, EPI_ISL_2968690, EPI_ISL_2968691, EPI_ISL_2968701, EPI_ISL_2968706, EPI_ISL_2968711, EPI_ISL_2968714, EPI_ISL_2968715, EPI_ISL_2968718, EPI_ISL_2968721, EPI_ISL_2968723, EPI_ISL_2968726, EPI_ISL_2968728, EPI_ISL_2968730, EPI_ISL_2982483, EPI_ISL_2982501, EPI_ISL_2982504, EPI_ISL_2982568, EPI_ISL_2982622			
see above	Viral Respiratory Lab, National Institute for Biomedical Research (INRB)	Pathogen Sequencing Lab, National Institute for Biomedical Research (INRB)	Allison Black; Amuri Aziza; Andrew Rambaut; Catherine Pratt; Eddy Kinganda-Lusamaki; Edith Nkwembe; Emmanuel Lokilo Lofiko; Francisca Muyembe Mawete; Ian Goodfellow; James Hadfield; Jean Claude Makangara; Jean-Claude Makangara Cigolo; Jean-Jacques Muyembe Tamfum; Josh Quick; Kristian Andersen; Matthias Pauthner; Michael Wiley; Nick Loman; Placide Mbala-Kingebeni; Steve Ahuka-Mundeke; Trevor Bedford
EPI_ISL_528386	Viral vaccines, VSVRI- Veterinary serum and vaccine research institute	Viral vaccines, VSVRI- Veterinary serum and vaccine research institute	A.A.; M.A.; Saad; Saleh
EPI_ISL_2344484	Virginia Division of Consolidated Laboratory Services	Virginia Division of Consolidated Laboratory Services	Virginia DCLS
EPI_ISL_2694588	Virology Centre, School of public health, Tehran University of Medical Sciences, Tehran, Iran	Genetics Research Center, University of Social Welfare and Rehabilitation Sciences	Hossein Najmabadi.; Jila Yavarian; Khadijeh Jalalvand; Kimia Kahrizi; Marzieh Mohseni; Talat Mokhtari-Azad; Zohreh Fattahi
EPI_ISL_2499903, EPI_ISL_2499907, EPI_ISL_2499909, EPI_ISL_2499913, EPI_ISL_2499916, EPI_ISL_2499917, EPI_ISL_2549198, EPI_ISL_2549199, EPI_ISL_2657327, EPI_ISL_2657329, EPI_ISL_2657330, EPI_ISL_2657332, EPI_ISL_2657333, EPI_ISL_2657334, EPI_ISL_2657335, EPI_ISL_2657336, EPI_ISL_2657337, EPI_ISL_2657338, EPI_ISL_2657339, EPI_ISL_2657341, EPI_ISL_2657343, EPI_ISL_2657344, EPI_ISL_2657345, EPI_ISL_2657346, EPI_ISL_2657347, EPI_ISL_2657352, EPI_ISL_2657353, EPI_ISL_2657354, EPI_ISL_2657355, EPI_ISL_2657356			
see above	Virology Department, Central Health Laboratory	The Francis Crick Institute	Bahadoor BS; Crawford M; Daniels RS; Goldstone R; Harvey R; Manraj SS; Nicod J; Patel H; Ramuth M; Sonoo J
EPI_ISL_1104285	Virology Department, Royal Infirmary of Edinburgh, NHS Lothian / School of Biological Sciences, University of Edinburgh	COVID-19 Genomics UK (COG-UK) Consortium	Colquhoun R; Cotton S; Dewar R; Hill V; Jackson B; McCrone JT; McHugh M; O'Toole Á; Rambaut A; Rooke S; Scher E; Templeton K; Yu X
EPI_ISL_473793	Virology Department, Royal Infirmary of Edinburgh, NHS Lothian / School of Biological Sciences, University of Edinburgh / Institute of Genetics and Molecular Medicine, University of Edinburgh	COVID-19 Genomics UK (COG-UK) Consortium	Balcaza C; Colquhoun R; Dewar R; Gallagher M; Hill V; Jackson B; McCrone JT; McHugh M; O'Toole Á; Rambaut A; Rooke S; Scher E; Templeton K; Williams TC; Yu X
EPI_ISL_1191603, EPI_ISL_1191604, EPI_ISL_1191605, EPI_ISL_1827649, EPI_ISL_1827650, EPI_ISL_1827667, EPI_ISL_1827674, EPI_ISL_1827677, EPI_ISL_1827690			
see above	Virology Department, Victoria Hospital, Plaine-Wilhems, Mauritius	National Institute for Communicable Diseases of the National Health Laboratory Service	Allam M; Amoako DG; Baboo SB; Bhiman JN; Ismail A; Mahlangu B; Manraj SS; Mohale T; Ntuli N; Ramuth M; Scheepers C; Sonoo J
EPI_ISL_636982	Virology Lab, National Institute for Biomedical Research (INRB)	Project group Epidemology of Highly Pathogenic Microorganisms, Robert Koch-Institute	Eddy Kinganda-Lusamaki; Essia Belarbi; Fabian Leendertz; Gabriel Mbunsu; Grit Schubert; Jasmin Schlotterbeck; Jean-Jacques Muyembe Tamfum; Sheila Makiala; Steve Ahuka-Mundeke
EPI_ISL_2955336	Virology Laboratories	Erasmus Medical Center	Abd Moniem Ain shoka; Amel Nagiub; Anne van der Linden; Annemiek van der Eijk; Bas Oude Munnink; Corine GeurtsvanKessel; Dalia Ramadan; David Nieuwenhuijse; Emmanuelle Munger; Galal Mahmoud; Irina Chestakova; Marion Koopmans; Marjan Boter; Mohamed Hassany; Mohamed K. Khalifa; Nancy El Guindy; Ramy Galal; Reina Sikkema; Richard Molenkamp; Salma Sayed; Shymaa A. Showky; Wael H. Roshdy; on behalf of the Dutch national COVID-19 response team.
EPI_ISL_2960150	Virology Laboratories	Erasmus Medical Center Department of Virology	Abd Moniem Ain shoka; Amel Nagiub; Anne van der Linden; Annemiek van der Eijk; Bas Oude Munnink; Corine GeurtsvanKessel; Dalia Ramadan; David Nieuwenhuijse; Emmanuelle Munger; Galal Mahmoud; Irina Chestakova; Marion Koopmans; Marjan Boter; Mohamed Hassany; Mohamed K. Khalifa; Nancy El Guindy; Ramy Galal; Reina Sikkema; Richard Molenkamp; Salma Sayed; Shymaa A. Showky; Wael H. Roshdy; on behalf of the Dutch national COVID-19 response team.
EPI_ISL_1711654, EPI_ISL_1711656	Virology Laboratory of São Vicente	Institut Pasteur de Dakar	Dia Ndongo; Diagne Moussa Moïse; Diallo Amadou; Diop Mamadou; Faye Ousmane; Loucoubar Cheikh; Nadia Rodrigues; Ndiaye Ndack; Sall Amadou Alpha; Sanders Kevin; Sankhe Safietou; da Luz Lima Mendonça Maria
EPI_ISL_2361901	Virology Laboratory, International Centre for Diarrhoeal Disease Research, Bangladesh (ICDDR,B)	Virology Laboratory, International Centre for Diarrhoeal Disease Research, Bangladesh (ICDDR,B)	Md. Mahfuzur Rahman; Mohammad Enayet Hossain; Mohammed Ziaur Rahman; Mojnu Miah; Mustafizur Rahman; Rashedul Hasan
EPI_ISL_1855056	Virology Laboratory, Scientific Department, Army Medical Center	Virology Laboratory, Scientific Department, Army Medical Center	Anella Monte; Anna Anselmo; Antonella Fortunato; Filippo Molinari; Florigio Lista; Francesco Giordani; Giancarlo Petralito; Giandomenico Cerreto; Riccardo De Sanctis; Silvia Fillo; Vanessa Vera Fain
EPI_ISL_508863, EPI_ISL_625456, EPI_ISL_677634, EPI_ISL_677636, EPI_ISL_1660225, EPI_ISL_1660226, EPI_ISL_1660228, EPI_ISL_1660235, EPI_ISL_1660241, EPI_ISL_1660245, EPI_ISL_1660246, EPI_ISL_1660250, EPI_ISL_1660258, EPI_ISL_1660261, EPI_ISL_1660271, EPI_ISL_1660279, EPI_ISL_1660281, EPI_ISL_1660289, EPI_ISL_1660297, EPI_ISL_1660298, EPI_ISL_1660301, EPI_ISL_1660305, EPI_ISL_1660310, EPI_ISL_1660312, EPI_ISL_1660314, EPI_ISL_1660317, EPI_ISL_1660318, EPI_ISL_1660322, EPI_ISL_1660329, EPI_ISL_1660330			
see above	Virology Unit, Institut Pasteur de Madagascar	Virology Unit, Institut Pasteur de Madagascar	Angela Brisebarre; Camille Capel; Cara Brook; Cara E. Brook; Christian Ranaivoson; Christophe Malabat; Corinne Maufrais; Cristina M. Tato; Emmanuelle Permal; Etienne Simon-Lorière; Frédéric Lemoine; Helisoa Razafimanjato; Jean-Michel Heraud; Joseph L. DeRisi; Louise Lefrançois; Marion Barbet; Maud Vanpeene; Michelle Tan; Méline Bizard; Norosoa Razanajatovo; Philippe Dussart; Soa Fy Andriamandimby; Sylvie Behillil; Sylvie van der Werf; Tsiry Randriambolamanantsoa; Vida Ahyong; Vincent Enouf; Vololoniaina Raharinosy
EPI_ISL_918365, EPI_ISL_918370, EPI_ISL_1534532, EPI_ISL_1706599, EPI_ISL_2501048, EPI_ISL_2693949, EPI_ISL_2801925, EPI_ISL_3031525, EPI_ISL_3031530, EPI_ISL_3031544, EPI_ISL_3045601			
see above	Virology Unit, Institut Pasteur du Cambodge	Virology Unit, Institut Pasteur du Cambodge	Cecile Troupin; Chau Darapheak; Chin Savuth; Erik A Karlsson; Etienne Simon-Loriere; Jurre Y Siegers; Kraing Sidonn; Leakhena Pum; Ly Sovann; Sokhoun Yann; Sophoannadedh Rath; Teyputita Ou; Veasna Duong; Yi Sengdoeurn
EPI_ISL_411902	Virology Unit, Institut Pasteur du Cambodge.	Virology Unit, Institut Pasteur du Cambodge (Sequencing done by: Jessica E Manning/Jennifer A Bohl at Malaria and Vector Research Research Laboratory, National Institute of Allergy and Infectious Diseases and Vida Ahyong from Chan-Zuckerberg Biohub)	Erik A Karlsson; Jennifer A Bohl; Jessica E Manning.; Philippe Dussart; Veasna Duong; Vida Ahyong
EPI_ISL_416458	Virology laboratory Ministry of Health Kuwait sequenced at Dasman Diabetes Institute	Dasman Diabetes Institute	Ebaa alOzairi; Fahd Al-Mulla; Motasem Melhem; Qais Al-Duwairi; Rasheeba iqbal; Sara Alqabandi; Sumi John
EPI_ISL_708021	Virology, Universitätsklinikum des Saarlandes	Epigenetics, Saarland University	Jörn Walter; Kathrin Kattler; Markus Vogelgesang; Sascha Tierling; Sigrun Smola; Stefan Lohse
EPI_ISL_3050736	Virus Ecology, NIH	Virus Ecology, NIH	Banakis, S.; Bushmaker, C.; Bushmaker, T.; Chung, M.; Ghedin, E.; Holbrook; M.G.; Munster; Roder, A.; V.J.; Van Doremalen, N.; Wang, W.
EPI_ISL_2507964	Vita Laboratoriot Oy	Expert Microbiology, National Institute for Health and Welfare	Carita Savolainen-Kopra; Erika Lindh; Haider al-Hello; Jani Halkilahti; Kirsi Liitsola; Niina Ikonen; Olli Vapalahti; Pekka Ellonen; Phuoc Truong; Päivi Laurila; Ravi Kant; Sari Hannula; Soile Blomqvist; Teemu Smura
EPI_ISL_2547367	Vredendal North Clinic wc VDN	National Health Laboratory Service/University of Cape Town (NHLS/UCT)	Arash Iranzadeh; Bruna Galvao; Carolyn Williamson; Deelan Doolabh; Diana Hardie; Gert Marais; Innocent Mudau; Lynn Tyers; Marvin Hsiao; Stephen Korsman
EPI_ISL_2836853, EPI_ISL_2836857, EPI_ISL_2836861, EPI_ISL_2836864, EPI_ISL_2836868, EPI_ISL_2836869, EPI_ISL_2836871, EPI_ISL_2836872, EPI_ISL_2836875, EPI_ISL_2836880, EPI_ISL_2836883, EPI_ISL_2836885, EPI_ISL_2836886, EPI_ISL_2836887, EPI_ISL_2836890, EPI_ISL_2836897, EPI_ISL_2836898, EPI_ISL_2836899, EPI_ISL_2836901, EPI_ISL_2836905, EPI_ISL_2836907, EPI_ISL_2836908, EPI_ISL_2836909, EPI_ISL_2836910, EPI_ISL_2836911, EPI_ISL_2836912, EPI_ISL_2836913, EPI_ISL_2836918, EPI_ISL_2836919, EPI_ISL_2836920, EPI_ISL_2836921, EPI_ISL_2836922, EPI_ISL_2836924, EPI_ISL_2836925, EPI_ISL_2836930, EPI_ISL_2836932, EPI_ISL_2836933, EPI_ISL_2836934, EPI_ISL_2836936, EPI_ISL_2836938, EPI_ISL_2836942, EPI_ISL_2836944, EPI_ISL_2836945, EPI_ISL_2836946, EPI_ISL_2836947, EPI_ISL_2836952, EPI_ISL_2836953, EPI_ISL_2836954, EPI_ISL_2836955, EPI_ISL_2836956, EPI_ISL_2836957, EPI_ISL_2836958, EPI_ISL_2836961, EPI_ISL_2836962, EPI_ISL_2836963, EPI_ISL_2836965, EPI_ISL_2836966, EPI_ISL_2836968, EPI_ISL_2967997, EPI_ISL_2968002, EPI_ISL_2968004, EPI_ISL_2968009, EPI_ISL_2968010, EPI_ISL_2968011, EPI_ISL_2968012, EPI_ISL_2968015, EPI_ISL_2968016, EPI_ISL_2968021, EPI_ISL_2968023, EPI_ISL_2968025, EPI_ISL_2968030, EPI_ISL_2968031, EPI_ISL_2968034			
see above	WACCBIP, University of Ghana, Accra, Ghana	WACCBIP, University of Ghana, Volta Road, Legon-Accra, Ghana	Bright K. Yemi; Collins M. Morang'a; Deborah N. A. Mettle; Dominic S. Y. Amuzu; Dominic S.Y. Amuzu; Evelyn B. Quansah; Evelyn Y. Bonney; Frederick Tei-Maya; Israel Osei-Wusu; Ivy A. Asante; Joe K. Mutungi; John K. Odoom; Joseph H.K. Bonney; Joyce M. Ngoi; Lawrence Ofori-Boadu; Lucas N. Amenga-Etego and Gordon A. Awandare; Mildred Adusei-Poku; Nicaise T. Ndam; Oliver Commey; Peter K Quashie; Philip M. Soglo; Samirah Saiid; Samuel Armoo; Vincent Appiah; Violette V. M'cormack; William K. Ampofo; Yaw Bediako
EPI_ISL_733021, EPI_ISL_1372304, EPI_ISL_1797437, EPI_ISL_2385079, EPI_ISL_2523488, EPI_ISL_2816149	WHO National Influenza Centre Russian Federation	WHO National Influenza Centre Russian Federation	Andrey Komissarov; Anna Ivanova; Artem Fadeev; Daria Danilenko; Dmitry Bazhenov; Dmitry Lioznov; Elena Nabieva; Georgii Bazykin; Kirill Varchenko; Ksenia Safina; Kseniya Komissarova; Maria Pisareva; Maria Timofeeva; Mikhail Bakaev; Nikita Yolshin; Oula Mansour; Oula Masour; Tamila Musaeva; Veronika Eder
EPI_ISL_860802,	WHO/Minsk	Charité Universitätsmedizin Berlin, Institut für Virologie	Barbara Mühlemann; Christian Drosten; Julia Schneider; Julia Tesch; Jörn Beheim-Schwarzbach; Shmialiova Natallia; Sivets Natallia; Talitha Veith; Terry Jones; Tobias Bleicker; Victor M Corman

EPI_ISL_860805, EPI_ISL_860806, EPI_ISL_860809			
EPI_ISL_1627990	WSSE w Krakowie	1. National Institute of Public Health - National Institute of Hygiene; 2. Eurofins Genomics Europe Sequencing GmbH	ECDC COVID-19 WGS support team; Eurofins Genomics Europe Sequencing Team; Gierczyński Rafał; Sadkowska-Todys Małgorzata; Wołkowicz Tomasz; Zacharczuk Katarzyna
EPI_ISL_2365408, EPI_ISL_2365412	WWF Bayanga field laboratory	Robert Koch Institute	F. H. Leendertz; F. S. Niatou-Singa; M. Ulrich; S. Calvignac-Spencer; T. B. Tombolomako; T. Fuh-Neba; U. Vickos
EPI_ISL_2365414, EPI_ISL_2365415, EPI_ISL_2365416, EPI_ISL_2365417	WWF Bayanga field laboratory	WWF Bayanga field laboratory	F. H. Leendertz; F. S. Niatou-Singa; M. Ulrich; S. Calvignac-Spencer; T. B. Tombolomako; T. Fuh-Neba; U. Vickos
EPI_ISL_2987085	WY Public Health Laboratory	Centers for Disease Control and Prevention Division of Viral Diseases, Pathogen Discovery	Alison Laufer Halpin; Ben L. Rambo-Martin; Clinton R. Paden; Dakota Howard; Darlene Wagner; Dave Wentworth; Dhwani Batra; Jasmine Padilla; Justin Lee; Katie Dillon; Krista Queen; Kristen Knipe; Kristine Lacek; Mark Burroughs; Matthew Schmerer; Mili Sheth; Peter Cook; Sam Shepard; Sarah Nobles; Shoshona Le; Suxiang Tong; Vivien Dugan; Yvette Unoarumhi
EPI_ISL_754579	Wadsworth Center, New York State Department.of Health	Wadsworth Center, New York State Department.of Health	Alexis Russel; Daryl M. Lamson; Erasmus Schneider; Erica Lasek-Nesselquist; John Kelly; Jonathan Plitnick; Kirsten St. George; Matthew Shudt; Melissa A Leisner; Navjot Singh; Sara Griesemer
EPI_ISL_456190, EPI_ISL_536790, EPI_ISL_548064, EPI_ISL_548101	Waikato Hospital	Institute of Environmental Science and Research (ESR)	Anja Werno; Antje van der Linden; Arlo Upton; Chris Mansell; David Hammer; Dragana Drinkovic; Erasmus Smit; Gary McAuliffe; Hana Sofia Andersson; Hermes Perez; James Ussher; Jill Sherwood; Jing Wang; Joep de Ligt; Josh Freeman; Julia Howard; Juliet Elvy; Lauren Jelly; Mary DeAlmeida; Matt Blakiston; Matt Storey; Matthew Rogers; Max Bloomfield; Michael Addidle; Michelle Balm; Muhammad Faisal; Nikki Freed; Olin Silander; Sally Roberts; Sarah Jefferies; Sharmini Muttaiyah; Susan Morpeth; Susan Taylor; Timothy Blackmore; Vani Sathyendran; Veronica Playle; Virginia Hope; Xiaoyun Ren
EPI_ISL_1833203	Wales Specialist Virology Centre Sequencing lab: Pathogen Genomics Unit	Public Health Wales Microbiology Cardiff Wales Specialist Virology Centre	Alec Birchley; Alexander Adams; Amy Gaskin; Angela Marchbank; Bree Gatica-Wilcox; Catherine Moore; Jason Coombes; Joanne Watkins; Joel Southgate; Johnathan Evans; Laura Gifford; Lauren Gilbert; Lee Graham; Malorie Perry; Matthew Bull; Nicole Pacchiarini; Sally Corden; Sara Kumziene-Summerhayes; Sara Rey; Sarah Taylor; Simon Cottrell; Sophie Jones; Tom Connor
EPI_ISL_417160	Washington State Department of Health	Seattle Flu Study	Chu etl al
EPI_ISL_416538	Wellington Hospital	Institute of Environmental Science and Research (ESR)	New Zealand; Newtown; Riddiford Street; Wellington 6021; Wellington Hospital; Wellington SCL
EPI_ISL_456161, EPI_ISL_456185, EPI_ISL_456320	Wellington SCL	Institute of Environmental Science and Research (ESR)	Anja Werno; Antje van der Linden; Arlo Upton; Chris Mansell; David Hammer; Dragana Drinkovic; Erasmus Smit; Gary McAuliffe; Hana Sofia Andersson; James Ussher; Jill Sherwood; Joep de Ligt; Josh Freeman; Julia Howard; Juliet Elvy; Lauren Jelly; Mary DeAlmeida; Matt Blakiston; Matt Storey; Matthew Rogers; Max Bloomfield; Michael Addidle; Michelle Balm; Sally Roberts; Sarah Jefferies; Sharmini Muttaiyah; Susan Morpeth; Susan Taylor; Timothy Blackmore; Vani Sathyendran; Veronica Playle; Virginia Hope; Xiaoyun Ren
EPI_ISL_1016878, EPI_ISL_1250695, EPI_ISL_2964938, EPI_ISL_2964939	Wellington SCL (WN)	Institute of Environmental Science and Research (ESR)	Anja Werno; Antje van der Linden; Arlo Upton; Chris Mansell; David Hammer; Dragana Drinkovic; Erasmus Smit; Gary McAuliffe; Hana Sofia Andersson; Hermes Perez; James Ussher; Jill Sherwood; Jing Wang; Joep de Ligt; Josh Freeman; Julia Howard; Juliet Elvy; Lauren Jelly; Mary DeAlmeida; Matt Blakiston; Matt Storey; Matthew Rogers; Max Bloomfield; Michael Addidle; Michelle Balm; Muhammad Faisal; Nikki Freed; Olin Silander; Olivia Stroeven; Rachel Boyle; Sally Roberts; SallyAnn Harbison; Sarah Jefferies; Sharmini Muttaiyah; Susan Morpeth; Susan Taylor; Timothy Blackmore; Vani Sathyendran; Veronica Playle; Virginia Hope; Xiaoyun Ren
EPI_ISL_2422499, EPI_ISL_2422500, EPI_ISL_2422505, EPI_ISL_2422515, EPI_ISL_2422517, EPI_ISL_2422520, EPI_ISL_2422521, EPI_ISL_2422526, EPI_ISL_2422530, EPI_ISL_2422549, EPI_ISL_2422571, EPI_ISL_2422595, EPI_ISL_2422614, EPI_ISL_2422624			
see above	West African Centre for Cell Biology of Infectious Pathogen, University of Ghana, Legon	WACCBIP, University of Ghana, Volta Road, Legon, Accra	Collins M. Morang'a; Dominic S. Y. Amuzu; Dominic S.Y. Amuzu; Edward Danso Fenteng; Emmanuel Kudjo; Evelyn B. Quansah; Evelyn Y Bonney; Frederick Tei-Maya; Ivy A Asante; Joe K. Mutungi; John K. Odoom; Joseph HK Bonney; Joyce M. Ngoi; Lawrence Ofori-Boadu; Lucas N. Amenga-Etego and Gordon A Awandare; Lucas N. Amenga-Etego and Gordon A. Awandare; Mildred Adusei-Poku; Nicaise T. Ndam; Patrick Tetteh Ababio; Peter K Quashie; Peter K. Quashie; Philip M Soglo; Philip M. Soglo; Samirah Saiid; Theophilus Odoom; Vincent Appiah; Violet MCormack; Violette M'cormack; William K. Ampofo; Yaw Bediako
EPI_ISL_1255107, EPI_ISL_1255110, EPI_ISL_1255150, EPI_ISL_1255152, EPI_ISL_1255164, EPI_ISL_1255170, EPI_ISL_1255176, EPI_ISL_1255186, EPI_ISL_1255188, EPI_ISL_1255199, EPI_ISL_1255229, EPI_ISL_1255231, EPI_ISL_1255244, EPI_ISL_1255250			
see above	West African Centre for Cell Biology of Infectious Pathogens (WACCBIP), University of Ghana, Accra, Ghana	West African Centre for Cell Biology of Infectious Pathogens (WACCBIP), University of Ghana, Volta Road, Legon-Accra, Ghana	; Abdoulaye B Diallo; Abdul-Karim Abass; Aisha Mohammed; Benjamin Demah Nuertey; Collins M. Morang'a; Dam Kenneth Mibut; Dominic S.Y. Amuzu; Emmanuella Amoako4; Evelyn B. Quansah; Frederick Kumi-Ansah; Frederick Tei-Maya; Gordon A Awandare; Joyce M. Ngoi; Kesego Tapela; Lucas N. Amenga-Etego; Nelson Kibinge; Oliver D Boakye5; Peter K Quashie; Philip M. Soglo; Samirah Saiid; Samuel Kaba Akoriyea; Theophilus Odoom; Vanessa Magnussen; Vincent Appiah; Yaw Bediako
EPI_ISL_451397	West China Hospital of Sichuan University	State Key Laboratory of Biotherapy of Sichuan University	Baowen Du; Binwu Ying; Chao Tang; Chuan Chen; Hancheng Wei; Jia Geng; Jing-wen Lin; Lu Chen; Mingxia Yu; Minjin Wang; Weimin Li; Yongzhao Zhou
EPI_ISL_2382409	Wisma Atlet	National Institute of Health Research and Development	Arie Ardiansyah Nugraha; Hana Apsari Pawestri; Hartanti Dian Ikawati; Kartika Dewi Puspa; Krisna Pangesti; Nelly Puspandari; Subangkit; Triyani Soekarso; Vivi Setiawaty
EPI_ISL_1300721, EPI_ISL_1493351, EPI_ISL_1731710, EPI_ISL_2500299, EPI_ISL_2692941, EPI_ISL_2692972	Wojewodzka Stacja Sanitarno-Epidemiologiczna w Olsztynie, Laboratorium Badan Epidemiologiczno-Klinicznych	Wojewodzka Stacja Sanitarno-Epidemiologiczna w Olsztynie, Laboratorium Badan Epidemiologiczno-Klinicznych	Aleksandra Kobiatko; Barbara Dolinska; Emilia Tarabasz; Ewa Liszewska; Marta Lukian; Monika Czerminska; Patryk Bielecki; Paulina Rozycka; Sylwia Krzetowska; Tomasz Jakubczak
EPI_ISL_3009173	Wojewódzki Szpital Specjalistyczny Laboratorium Mikrobiologiczne	1. Academic Center for Pathomorphological and Genetic-Molecular Diagnostics ltd, Bialystok, Poland 2. National Institute of Public Health - National Institute of Hygiene, Warsaw, Poland	Anetta Sulewska; Jacek Nikliński; Janusz Dzięcioł; Joanna Kiśluk; Katarzyna Zacharczuk; Konrad Raczkowski; Magdalena Nowakowska; Małgorzata Sadkowska-Todys; Piotr Karabowicz; Piotr Majewski; Przemysław Biecek. Joanna Reszeć; Radosław Charkiewicz; Tomasz Wołkowicz
EPI_ISL_454905, EPI_ISL_454939, EPI_ISL_454982	Wuhan Chain Medical Labs (CMLabs)	State Key Laboratory of Biotherapy of Sichuan University	Baowen Du; Binwu Ying; Chao Tang; Chuan Chen; Hancheng Wei; Jia Geng; Jing-wen Lin; Lu Chen; Mingxia Yu; Minjin Wang; Weimin Li; Yongzhao Zhou
EPI_ISL_462931	Wyoming Public Health Laboratory	Center for Global Health, University of New Mexico Health Sciences Center	Cari Sloma; Darrell Dinwiddie; Daryl Domman; Kurt Schwalm; Noah Hull; Rob Christensen; Wanda Manley
EPI_ISL_1532099, EPI_ISL_2404707, EPI_ISL_2444758, EPI_ISL_2716283, EPI_ISL_2775587	Wyoming Public Health Laboratory	Wyoming Public Health Laboratory	Ashley Norberg; Brian Dominguez; Brittany Oher; Cari Sloma; Channing Weber; Chayse Rowley; Elliot Thomasson; Jim Mildenberger; Lynette Gumbleton; Marley Goetz; Noah Hull; Robert Petit; Sam Britz; Taylor Fearing; Wanda Manley; and Rob Christensen
EPI_ISL_1595852	Yunan Center for Disease Control and Prevention	National Institute for Viral Disease Control and Prevention, China CDC	Jienan Zhou; Meiling Zhang; Senquan Jia; Xiang Zhao; Xiaoqing Fu; Yenan Feng; Yuchao Wu; Zhixiao Chen
EPI_ISL_2727271	ZARV/NHLS, Department Medical Virology, University of Pretoria	KRISP, KZN Research Innovation and Sequencing Platform	Adriano Mendes; Amy Strydom; Emmanuel SJ; Giandhari J; Lessells R; Micheala Davids; Naidoo Y; Pillay S; Ramphal U; Sim Mayaphi and Marietjie Venter; Tegally H; Wilkinson E; de Oliveira T
EPI_ISL_2727350, EPI_ISL_2899747	ZARV/NHLS, Department Medical Virology, University of Pretoria	KRISP, KZn Research Innovation and Sequencing Platform	Adriano Mendes; Amy Strydom; Emmanuel SJ; Giandhari J; Lessells R; Micheala Davids; Naidoo Y; Pillay S; Ramphal U; Sim Mayaphi and Marietjie Venter; Tegally H; Wilkinson E; de Oliveira T
EPI_ISL_3039570	ZOL	Jessa	Berden et al. on behalf of the Jessa_cmdLab
EPI_ISL_666636, EPI_ISL_718079, EPI_ISL_718096	ZOTZ KLIMAS MVZ Düsseldorf-Centrum GbR ÜBAG für Labormedizin, Genetik, Zytologie, Pathologie	Center of Medical Microbiology, Virology, and Hospital Hygiene, University of Duesseldorf	Alexander Dilthey; Andreas Walker; Ashley-Jane Duplessis; Daniel Strelow; Jessica Nicolai; Jörg Timm; Katrin Hoffmann; Klaus Pfeffer; Lisanna Hülse; Malte Kohns Vasconcelos; Marek Korencak; Maximilian Damagnez; Nadine Lübke; Patrick Finzer; Rainer Zotz; Tobias Wienemann; Torsten Houwaart
EPI_ISL_2377264	Zakład Laboratoryjnej Diagnostyki Klinicznej USK Białystok	1. National Institute of Public Health - National Institute of Hygiene; 2. Eurofins Genomics Europe Sequencing GmbH	ECDC COVID-19 WGS support team; Eurofins Genomics Europe Sequencing Team; Gierczyński Rafał; Sadkowska-Todys Małgorzata; Wołkowicz Tomasz; Zacharczuk Katarzyna
EPI_ISL_2674012, EPI_ISL_2674013	Zavod za javno zdravstvo Šibensko- Kninske Županije	Hrvatski zavod za javno zdravstvo	Irena Tabain; Ivana Ferenčak
EPI_ISL_1911196, EPI_ISL_1911197, EPI_ISL_1911250	Zhejiang Provincial Center for Disease Control and Prevention Zhoushan Center for Disease Prevention and Contol	Zhejiang Province Center of Disease Control and prevention	Bing WU; Hongling Wang; Yanjun Zhang
EPI_ISL_2716621	aboratoire National de Référence pour les Fièvres Hémorragiques Virales, Centre Muraz	Centre Muraz	Ange Badjo; Arsène Zongo; Essia Belarbi; Fabian Leendertz; Grit Schubert; Jasmin Schlotterbeck; Saïdou Ouedraogo; Soumeya Ouangraoua; Thérèse Kagone
EPI_ISL_1727293, EPI_ISL_1727369	amedes MVZ Hannover	Robert Koch Institute	
EPI_ISL_1281348	amedes MVZ Jena	Robert Koch Institute	
EPI_ISL_1141025	amedes MVZ für Laboratoriumsmedizin Rhein/Ruhr	Robert Koch Institute	
EPI_ISL_828071, EPI_ISL_828410, EPI_ISL_829200	deCODE genetics	deCODE genetics	Agnar Helgason; Alma Moller; Arna B Agustsdottir; Arnaldur Gylfason; Asgeir Sigurdsson; Aslaug Jonasdottir; Berglind Eiriksdottir; Bjarni Thorbjornsson; Brynjar O Jensson; Daniel F Gudbjartsson; Droplaug N Magnusdottir; Elisabet E Gardarsdottir; Emil A Thorarensen; Gardar Sveinbjornsson; Gisli Masson; Gudmundur Georgsson; Gudmundur L Norddahl; Gudrun Sigmundsdottir; Hakon Jonsson; Hannes Eggertsson; Hilma Holm; Ingileif Jonsdottir; Jona Saemundsdottir; Kamilla S Josefsdottir; Kari Stefansson; Karl G Kristinsson; Kjartan R Gudmundsson; Kristin E Sveinsdottir; Louise le Roux; Maney Sveinsdottir; Olafia S Gretarsdottir; Olafur T Magnusson; Pall Melsted; Patrick Sulem; Run Fridriksdottir; Solvi Rognvaldsson; Thora R Gunnarsdottir; Thordur Kristjansson; Thorolfur Gudnason; Unnur Thorsteinsdottir
EPI_ISL_732911	genXone SA, Molecular Diagnostics Laboratory / NZOZ	genXone SA, Research & Development Laboratory	Grzegorz Nowicki; Jakub Grabowski; Maciej Sykulski; Michał Kaszuba; Monika Mańkowska-Woźniak; Natalia Drwęska-Matelska; Łukasz Krych
EPI_ISL_458286	unknown	Bundeswehr Institute of Microbiology	Antwerpen; Bestehorn-Willmann; Eckstein, S.; Handrick, S.; M.C.; M.H.; M.S.; Naija, H.; R. and Ben Moussa, M.; Rehn, A.; Stoecker, K.; Walter; Woelfel
EPI_ISL_463005, EPI_ISL_463006	unknown	Clinical virology	Fares, W.; Triki, H.
EPI_ISL_487270	unknown	Communicable Disease Laboratory, Public Health Directorate	AlTaif, Z.; AlWasti, H.; Shehab, F.; Zaed, A.
EPI_ISL_437612,	unknown	Faculty of Medicine	Buathong, R.; Bunprakob, S.; Ghai, S.; Joyjinda, Y.; Mungaomklang, A.; Petcharat, S.; Plipat; Prasithsirikul, W.; Rodpan, A.; Sirichan, N.; T. and Hemachudha, T.; Wacharapluesadee, S.

EPI_ISL_437616, EPI_ISL_437618, EPI_ISL_437622			
EPI_ISL_507012	unknown	Infectious Diseases Research, King Abdullah International Medical Research Center (KAIMRC)	Alghoribi; M.F.
EPI_ISL_476559	unknown	Laboratoire Sciences et Technologies de la Santé (STS) Institut Supérieur des Sciences de la Santé Université Hassan 1er, Settat, Morocco	Abderraouf Hilali; Amal Souiri; Hajar Lemriss; Jawad Bouzid; Mustapha Mouallif; Narjis Amar; Sanaâ Lemriss; Saâd EL Kabbaj; Touria Essayagh
EPI_ISL_483060	unknown	Microbiology, Canterbury Health Laboratories	Anderson, T.; Dilcher, M.
